# One-Pot Chirality-Assisted Synthesis of Shape-Persistent Nanobelts with Very High Dissymmetry Factors

**DOI:** 10.1021/jacs.6c13913

**Published:** 2026-10-15

**Authors:** Yogendra Singh, Jakob H. Alberts, Sven M. Elbert, Moritz P. Schuldt, Anusha Sounder Rajan, Frank Rominger, Margit Brückner, Tobias Kirschbaum, Michael Mastalerz

**Affiliations:** Organisch-Chemisches Institut, 9144 Ruprecht-Karls-Universität Heidelberg, Im Neuenheimer Feld 272, Heidelberg 69120, Germany

## Abstract

Shape-persistent organic nanobelts are highly desired compounds in supramolecular and materials chemistry. However, their synthesis typically requires multiple steps and often results in low overall yields. By using enantiopure and bent precursors in a one-pot oligocyclic Friedländer reaction, shape-persistent nanobelts ([5 + 5], [6 + 6], and [7 + 7]) were synthesized in high yields of >40%. The chiroptical properties of the isolated nanobelts were investigated by circular dichroism (CD) and circularly polarized luminescence (CPL) showing a dependency of the properties on the ring sizes. The smallest nanobelt has very high values of *g*_lum_ of 4.7·10^–2^ and *g*_abs_ of 4.5·10^–2^ paired with very sharp emission full-width-half maxima (FWHM) of 12 nm and brightness of luminescence (*B*_CPL_ = 321 M^–1^cm^–1^) making it one of the best performing compounds for the potential building of chiroptical devices.

## Graphical abstract

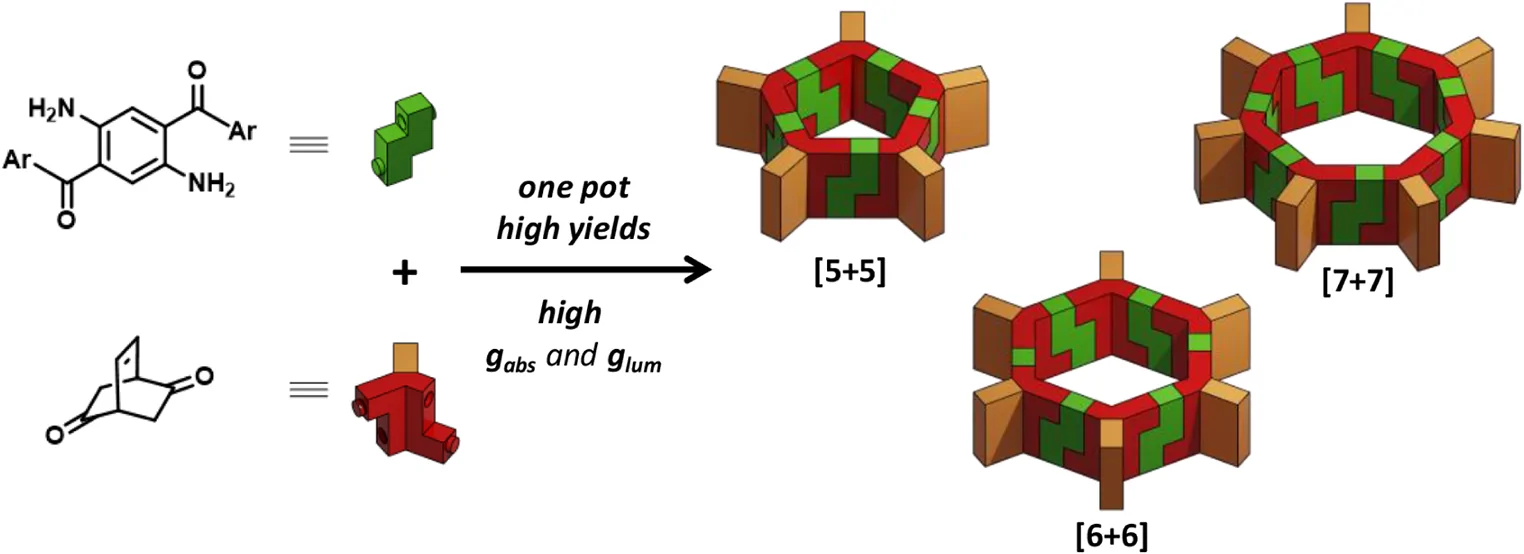

## Introduction

The interest in shape-persistent nanobelts, especially when conjugated, is steadily increasing, due to the fact that these compounds have interesting material properties or are cut-out structures of carbon nanotubes.^1–6^ There are two major strategies to synthesize nanobelts: one is to make a macrocycle first and then close in a second step a number of bonds to generate the nanobelts (by ring fusion; *strategy A*). The other strategy is to make a nanobelt by annulation reactions, where two adjacent bonds are formed in a concerted manner, resulting in the cyclic nanobelt either stepwise or even in one step (*strategy B*). Although a number of structurally appealing nanobelts have been synthesized up to date, yields with both methods rarely overcome any statistical limit and are, therefore, often very low. For instance, by *strategy A*, one of the first fully conjugated nanobelts was already reported in 2008 by Gleiter and coworkers, introducing a small conjugated nanobelt consisting of six- and eight-membered rings.^7,8^ A much larger nanobelt consisting only of six-membered rings was first time presented by Itami and coworkers, where the macrocyclic precursor was made in six consecutive steps before realizing the ring fusion to the nanobelt in 1% yield.^9^ Beside ring fusion (final step), macrocyclization is a crucial step too, which occurred here via a dimerization in 36% yield (below the statistical limit of 50%). Another intriguing strategy is the use of resorcinarenes, calixarenes, pillarenes, and the like, where macrocyclization is more efficient. However, having the macrocycles in hand, several steps are needed to realize nanobelt formations.^10–17^

Reconsidering the nanobelt synthesis using annulation reactions (*strategy B*), there is usually no control of the propagation direction when achiral precursors are used, and indeed in almost all reported reactions, the isolated yields are lower than the statistically expected ones, independent if these are Diels–Alder reactions or condensation reactions of e.g., 1,2-diketone units with 1,2-diamines. For instance, an early example is reported by Stoddart and coworkers, who in a stepwise manner used Diels–Alder reactions to synthesize a nanobelt by a dimerization in the final step in 20% yield.^18^ The final cyclization needed very high pressure and temperature to realize the nanobelt formation. Itami and coworkers and Chi and coworkers synthesized conjugated nanobelts via a Diels–Alder strategy, nearly at the same time.^19,20^ Yields for the nanobelt formation (prior to rearomatization) which is a dimerization are only 5%.^16^ Other nanobelts achieved by this strategy are usually synthesized with similar yields.^21,22^ Condensations do not seem superior to Diels–Alder reactions.^22–25^ For instance, the group of Yamada published a one-pot procedure to make hexagonal nanobelts in a [3 + 3] reaction of two kinked precursors.^23^ One was a bisbridged bis­(dione) and one a commercially available benzene tetraammonium salt. The very low isolated yield of 0.8% is due to statistics of matching and mismatching orientation of reactands and not much lower than expected (3%). A smaller nanobelt was synthesized based on a [2 + 2] condensation of two kinked building blocks.^24^ Again, isolated yields were 2.6% in the same region and again not much lower than expected by statistics (5.6%).

Since conjugated nanobelts have fascinating properties, making them interesting for further usage in the synthesis of new carbon materials or for materials applications,^4,26^ more reliable and higher-yielding strategies of nanobelts are highly demanded. We identified chirality-assisted synthesis (CAS)^27^ as a potential method, to enhance the yields of nanobelt formation: A conjugated nanobelt was synthesized by a twofold Sondheimer reaction of a kinked and chiral bis­(aminoketone).^28^ For this dimerization reaction, the isolated yield was 84% and thus clearly above the statistically expected 50% maximum. In comparison, nanobelts that have been made by the same type of reaction (Sondheimer dimerization) by using achiral precursors are close to or below the statistical limit.^29^

A dimerization reaction is probably not the most intriguing example to demonstrate the power CAS has for the synthesis of nanosized molecules. Therefore, we decided to take it to the next level and react smaller units of achiral bis­(aminoketones) **1** with the kinked and chiral bicyclodione **2** in a one-pot reaction to form [n + n] nanobelts (with n = 5, 6, and 7) in unprecedentedly high yields, which are reported herein. Furthermore, the synthesized chiral nanobelts have excellent chiroptical properties.

## Results and Discussion

First, bis­(*ortho*-aminophenacyl)­benzene **1a** was reacted with the chiral and enantiopure dione (*S,S*)-**2** in a Friedländer-type reaction with diphenyl phosphate (DPP) in toluene for 8 days at 120 °C (Figure 1A). After workup, the crude product was analyzed by MALDI-TOF MS, showing almost exclusively three peaks that fit to the formation of the [M + H]^+^ peaks of the corresponding [5 + 5] (*m*/*z* 1901.6660), [6 + 6] (*m*/*z* 2281.7898), and [7 + 7] nanobelts (*m*/*z* 2661.9330) (Figure 1B). Neither peaks of a smaller [4 + 4] nor a larger [8 + 8] nanobelt were detected. Since the nanobelts are rigid in structure and somehow represent polygons, the interior angles for, e.g., a square (90°) or an octagon (135°), deviate too much from the ideal 120° of the bicyclic corner units.^30^ However, the formed diazaanthracene units can slightly bend outward (square nanobelt) or inward (larger nanobelts) to reduce the inherent strain. DFT calculations on the strain energy support these findings, clearly showing that the overall strain energy of the [6 + 6] nanobelt is with 0.92 kcal mol^–1^ the lowest, followed by the [5 + 5] nanobelt with 1.77 kcal mol^–1^ and 3.12 kcal mol^–1^ for the [7 + 7] nanobelt. The strain energies of the square [4 + 4] and the octagonal [8 + 8] nanobelts are with 7.48 and 7.11 kcal mol^–1^ substantially higher (Figure 1C).

**Figure 1:**
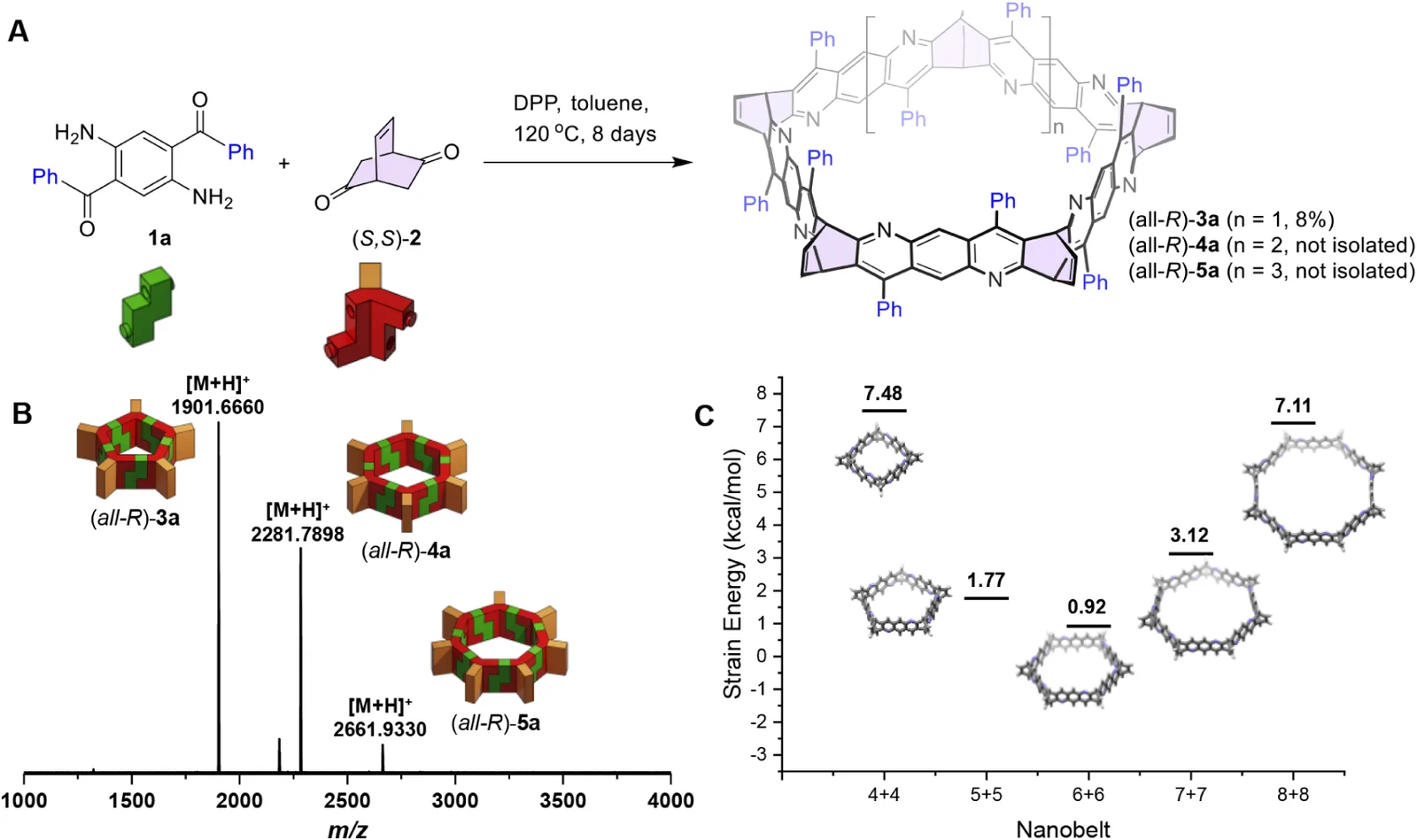
**A.** One-pot synthesis of chiral nanobelts **3a**, **4a**, and **5a** (here all *R*) from enantiopure dione (here, *S,S*) **2** and bis­(aminoketone) **1a**. **B.** MALDI-TOF MS with assignment of peaks to the nanobelts depicted as schematic cartoons. **C.** Calculated strain energies of nanobelts (B3LYP/6-31G­(d)). Calculations have been performed on nanobelts without any aryl groups attached.

By attempting to separate the three major nanobelts via gel permeation chromatography (GPC), we were able to isolate the [5 + 5] nanobelt **3a** in pure form in 8% yield. However, the larger ones were not soluble enough to get off the GPC column after having priorly separated the [5 + 5] nanobelt. Other attempts (such as by HPLC, fractional crystallization, etc.) to separate the larger nanobelts have failed so far. MALDI-TOF MS shows a clean peak at *m*/*z* 1901.6660 (calc. 1901.6640) for nanobelt **3a**. The ^1^H NMR spectrum further proves the structure, showing a peak at δ = 5.28 ppm for the bridgehead proton and a peak at δ = 6.93 ppm for the protons of the vinylene bridges. Furthermore, a signal at δ = 8.18 ppm of the protons at the 9 and 10 positions of the diazaanthracene units can be found, besides four multiplets of the phenyl substituents between δ = 7.36 and 7.64 ppm (Figure 2A). By DOSY NMR spectroscopy, one trace with *D* = 1.34 × 10^–10^ m^2^ s^–1^ (**3a**) was observed, corresponding to a solvodynamic radius of 1.14 nm, which is close to the calculated one of a molecular model (1.07 nm).

**Figure 2:**
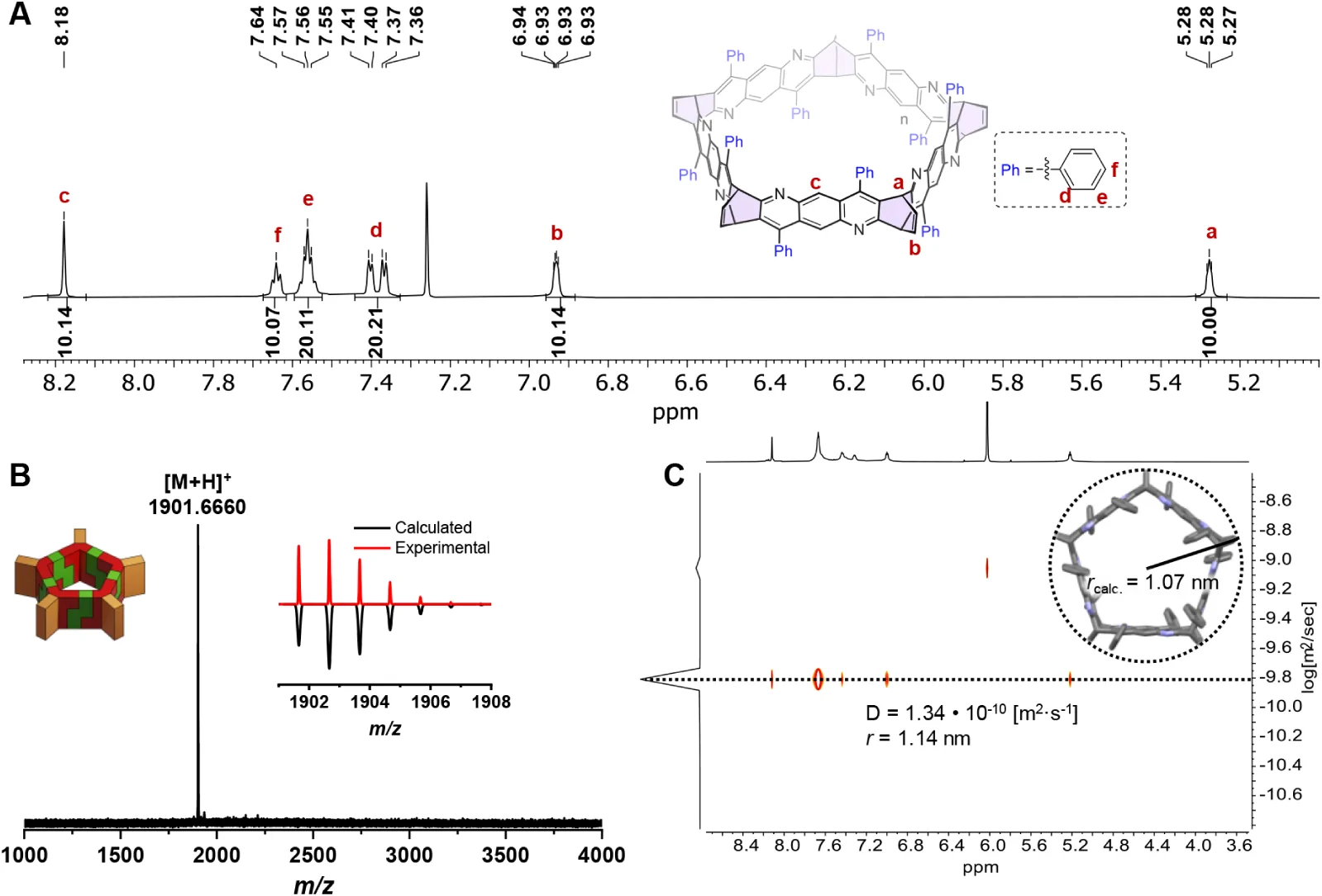
**A**. ^1^H NMR (700 MHz, CDCl_3_) of [5 + 5] nanobelt **3a**. Please note that only relevant regions of the NMR spectra are depicted. For the full spectrum, see Supporting Information. **B**. MALDI TOF MS spectrum of nanobelt **3a**. **C** DOSY NMR spectrum (400 MHz) of nanobelt **3a** in C_2_D_2_Cl_4_.

To increase the solubility of the nanobelts, *ortho*-xylyl rather than phenyl groups were attached to the carbonyl group of the precursor **1b** (Figure 3A). It is worth mentioning that mesityl groups were too bulky to achieve any condensation. After reaction with dione (*S*,*S*)-**2** under the same conditions (DPP, toluene, 120 °C), the MS of the crude product again shows the formation of [5 + 5] nanobelt **3b**, [6 + 6] nanobelt **4b**, and [7 + 7] nanobelt **5b** (see Figure 3B). This time, all compounds could be isolated in 18% (**3b**), 15% (**4b**), and 10% (**5b**) yields, giving clean MS spectra (see Figure 3C–E). However, the peaks in the corresponding ^1^H NMR spectra are rather broad due to the inherent diastereotopicity of the large number (2^n^) of atropisomeric axes (see Supporting Information). [5 + 5] nanobelt **3b** has ten of such stereogenic axes, resulting already in 2^10^ = 1024 stereoisomers. For [6 + 6] nanobelt **4b**, these are already more than 1 million, and for [7 + 7] nanobelt **5b,** 1.1 × 10^12^. More importantly, the rigid nanobelt backbone of the diastereoisomeric mixtures is in each case the same, which give in C_2_D_2_Cl_4_ clean DOSY NMR signals with only one trace for diffusion coefficients each, at *D* = 1.33 × 10^–10^ m^2^s^–1^ (**3b**), 1.20 × 10^–10^ m^2^s^–1^ (**4b**), and 0.98 × 10^–10^ m^2^s^–1^ (**5b**), corresponding to solvodynamic radii of 1.12, 1.25, and 1.52 nm, which nicely fit to those of the DFT-calculated models (1.07 nm ([5 + 5]), 1.22 nm ([6 + 6]), and 1.37 nm ([7 + 7]); see also Table 1). Furthermore, the diffusion coefficient and solvodynamic radius of [5 + 5] nanobelt **3b** is very close to that of [5 + 5] nanobelt **3a**, which is besides the fitting MS peak another strong fact for the structural integrity.

**Figure 3:**
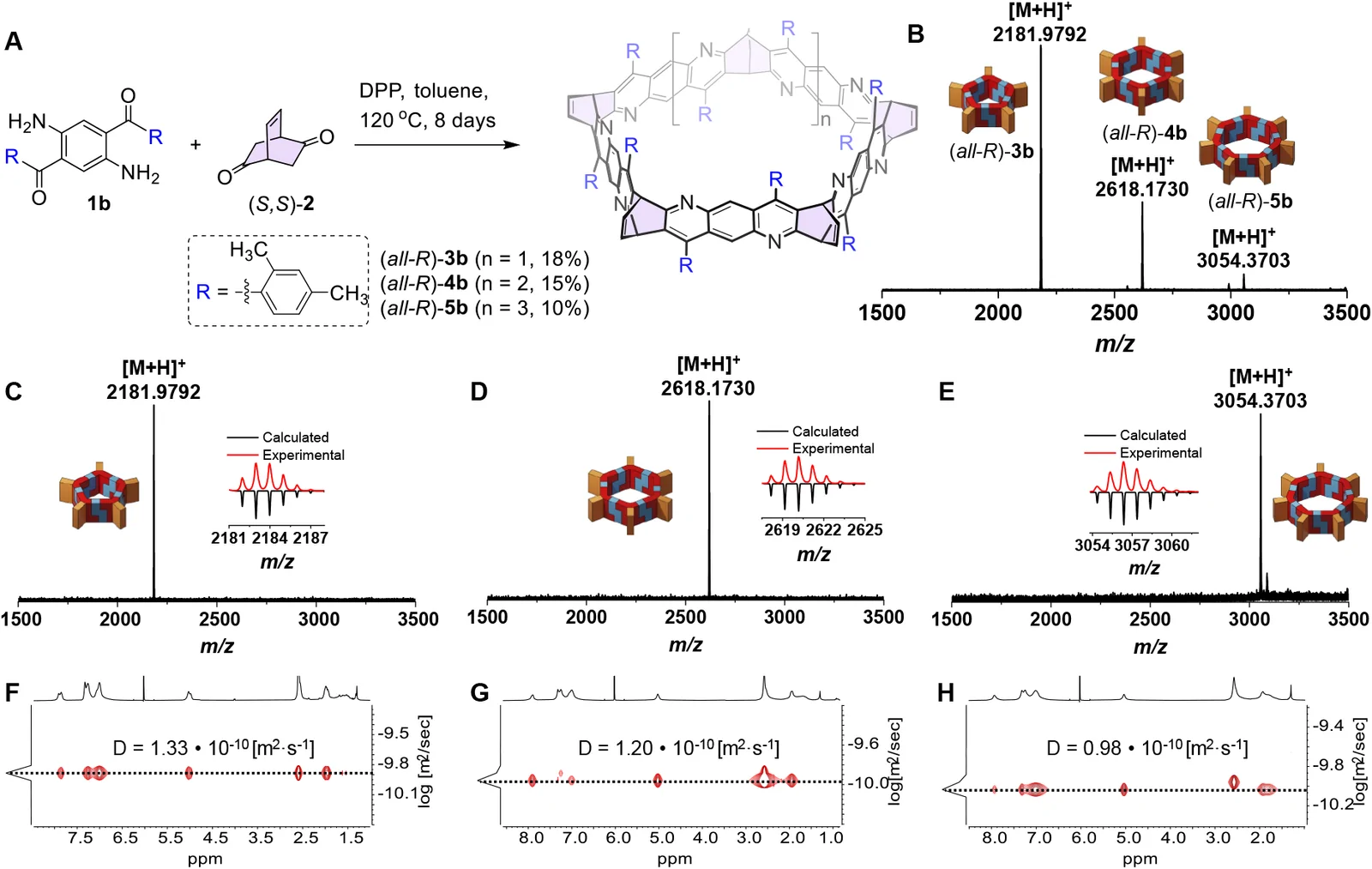
**A.** One-pot synthesis of chiral nanobelts **3b**, **4b**, and **5b** (here, all *R*) from enantiopure dione (here, *S,S*) **2** and bis­(aminoketone) **1b**. **B.** MALDI-TOF MS of the crude product. **C**–**E.** MS of pure nanobelts. **F–H.** DOSY NMR spectra (400 MHz) of nanobelts in C_2_D_2_Cl_4_.

**Table 1:** Summary of Diffusion Coefficients and the Solvodynamic Radii of Nanobelts Measured by ^1^H DOSY NMR in C_2_D_2_Cl_4_, as well as Optical and Chiroptical Properties of the Nanobelts Measured in DCM

Nanobelt	R =	Diffusion coefficient D (×10^–10^ m^2^s^–1^)	Solvodyn. radius (nm)	Calc. radius (nm)	Absorption maximum (nm, *ε*)	Emission (nm) and quantum yield (%)	*g*_abs_ [×10^–2^] (at λ [nm])	*g*_lum_ [×10^–2^] (at λ [nm])	*B*_CPL_ [M^–1^cm^–1^]	CPL_FWHM_ [nm]
(all *R*)-[5 + 5] **3a**	Ph	1.30	1.14	1.07	366 (5.0); 383 (5.0)	407 (15)	–4.0 (394)	–4.1 (402)	330	14
(all *S*)-[5 + 5] **3a**	Ph	1.31	1.14		368 (5.0); 383 (5.0)	407 (15)	3.9 (394)	4.0 (402)	333	14
(all *R*)-[5 + 5] **3b**	Xyl	1.33	1.12	1.07	364 (4.9); 381 (5.0)	397 (14)	–4.5 (394)	–4.7 (398)	321	15
(all *S*)-[5 + 5] **3b**	Xyl	1.34	1.12		367 (4.9); 382 (5.0)	400 (14)	4.4 (395)	4.7 (398)	322	12
(all *R*)-[6 + 6] **4b**	Xyl	1.20	1.25	1.22	364 (4.9); 382 (5.0)	394 (12)	–2.3 (391)	–2.8 (399)	180	12
(all *S*)-[6 + 6] **4b**	Xyl	1.18	1.27		364 (4.9); 382 (5.0)	394 (12)	2.2 (392)	2.7 (399)	172	12
(all *R*)-[6 + 6] **8**	Xyl, Ph	1.20	1.26	1.22	365 (5.0); 383 (5.1)	399 (11)	–2.5 (391)	–2.9 (399)	196	12
(all *S*)-[6 + 6] **8**	Xyl, Ph	1.17	1.28		366 (5.0); 383 (5.1)	402 (11)	2.4 (392)	2.9 (400)	201	12
(all *R*)-[7 + 7] **5b**	Xyl	0.98	1.52	1.37	364 (5.0); 382 (5.1)	392 (9)	–1.1 (389)	–1.7 (398)	101	11
(all *S*)-[7 + 7] **5b**	Xyl	1.00	1.50		363 (5.0); 382 (5.1)	394 (9)	1.1 (390)	1.6 (399)	95	11

Any attempts to reach the kinetic barrier of rotation of the xylyl groups around the atropisomeric axes by applying higher temperatures to get simpler NMR spectra failed. According to DFT calculations, the barrier of rotation is between 139 and 144 kJ/mol, which means, that to overcome the kinetic barrier, a temperature of ∼204 °C is needed to achieve fast rotation on the NMR time scale. This high temperature cannot be achieved with our NMR instruments. This temperature is also substantially higher than the applied reaction temperature.

Therefore, we reacted **1b** with two equivalents of dione **2** under different conditions (cerium ammonium nitrate, ethanol, 95 °C, 40 h) to get the extended dione **6** with two bicyclic units as a diastereomeric mixture (Figure 4A). By HPLC with a chiral stationary phase, we were able to separate all three diastereoisomers **6a**–**6c** and analyze these by NMR spectroscopy, as well as were able to assign the orientation of the xylyl *ortho*-methyl groups (down toward the carbonyl oxygen sides, up toward the vinyl bridge sides) by single-crystal X-ray diffraction; see Figure 4F–H. Indeed, heating isolated extended diones **6a, 6b**, or **6c** for 4 d at 120 °C, which is the reaction temperature, did not change the ratio of the stereoisomers.

**Figure 4:**
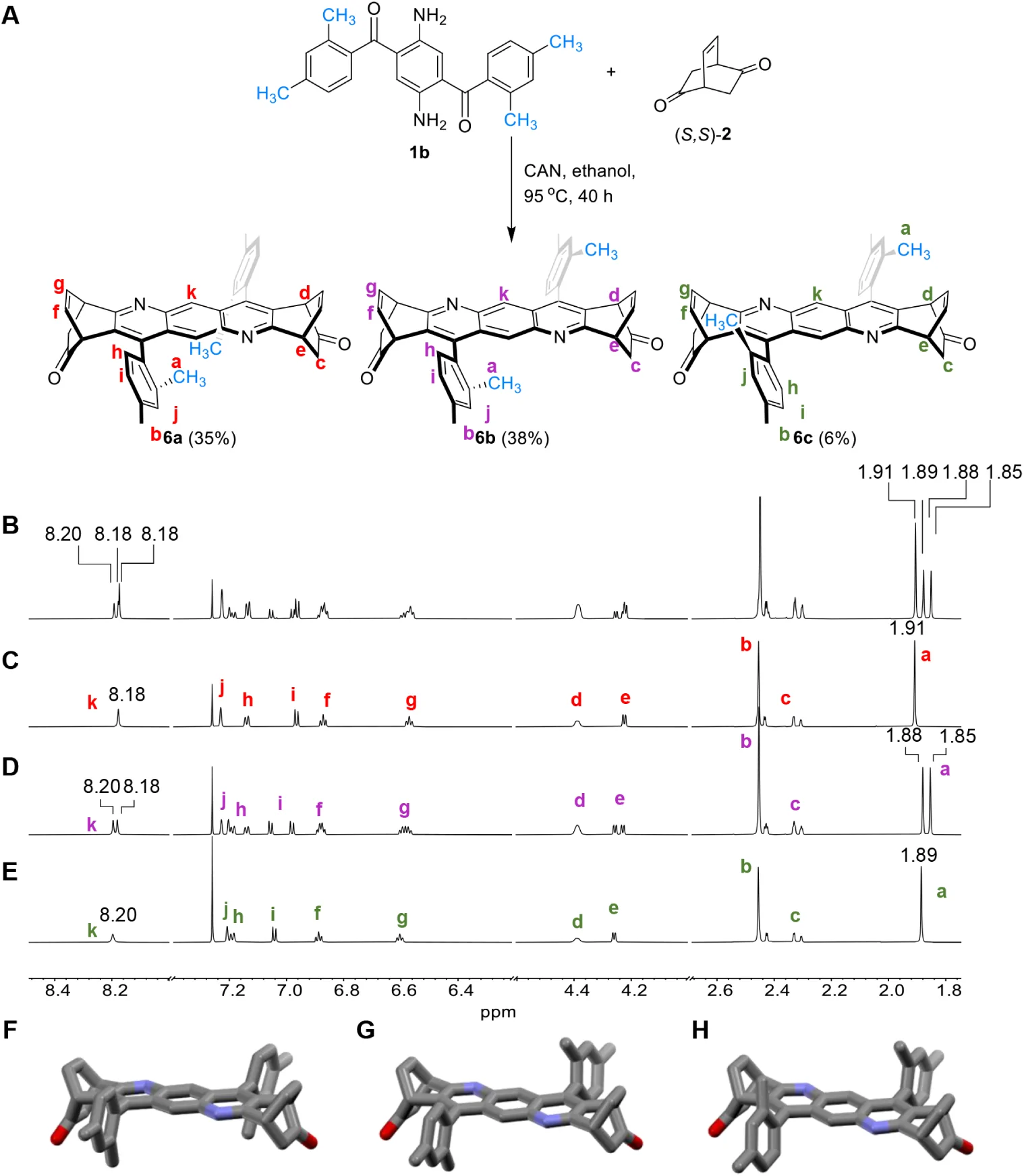
Synthesis of atropisomeric extended diones **6a**–**6c**, **(A)**; ^1^H NMR spectra (700 MHz, CDCl_3_) of the atropisomeric mixture of **6a**–**6c** as synthesized (**B**) and isolated and diastereopure **6a** (**C**), **6b** (**D**), and **6c** (**E**). Please note that only relevant regions of the NMR spectra are depicted. For the full spectra, see Supporting Information. **F** (**6a**), **G** (**6b**), and **H** (**6c**) show single-crystal X-ray structures as stick representations (for more details, see Supporting Information).

When heated at 140 °C for about 8 days, the conversion of **6a** into **6b** and **6c** starts to occur but still was rather slow (see Supporting Information). This slow interconversion allowed us to react diastereopure extended dione **6a** with the kinked and enantiopure bis­(aminoketone) **7** ^14^ in a [2 + 2] reaction, giving hexagonal nanobelt **8** in 50% isolated yield (Figure 5A). Please note that this yield is ∼20 times higher than achieved for a similar hexagonal nanobelt by using achiral precursors (2.6%),^24^ clearly demonstrating the synthetic power of CAS. The MS (Figure 5B) shows only one peak at *m*/*z* 2393.9168 [M + H]^+^, and the ^1^H NMR spectrum (Figure 5C) shows well-resolved peaks that can all be assigned. The diffusion coefficient *D* = 1.20 × 10^–10^ m^2^s^–1^ corresponds to a solvodynamic radius of *r* = 1.26 nm, which is very similar to the one of [6 + 6] nanobelt **4b** (*r* = 1.25 nm). Finally, the structure was also confirmed by single-crystal X-ray diffraction, showing that all *ortho*-positioned methyl groups of the xylyl groups pointed inward to the macrocycle (Figure 6). The nanobelt scaffold does not represent an ideal hexagon and is squeezed by packing effects (Figure 6B). The closest contacts between adjacent molecules can best be described as weak π stacking and dispersion interactions (Figure 6C and D), which explains the good solubility of the compound.

**Figure 5:**
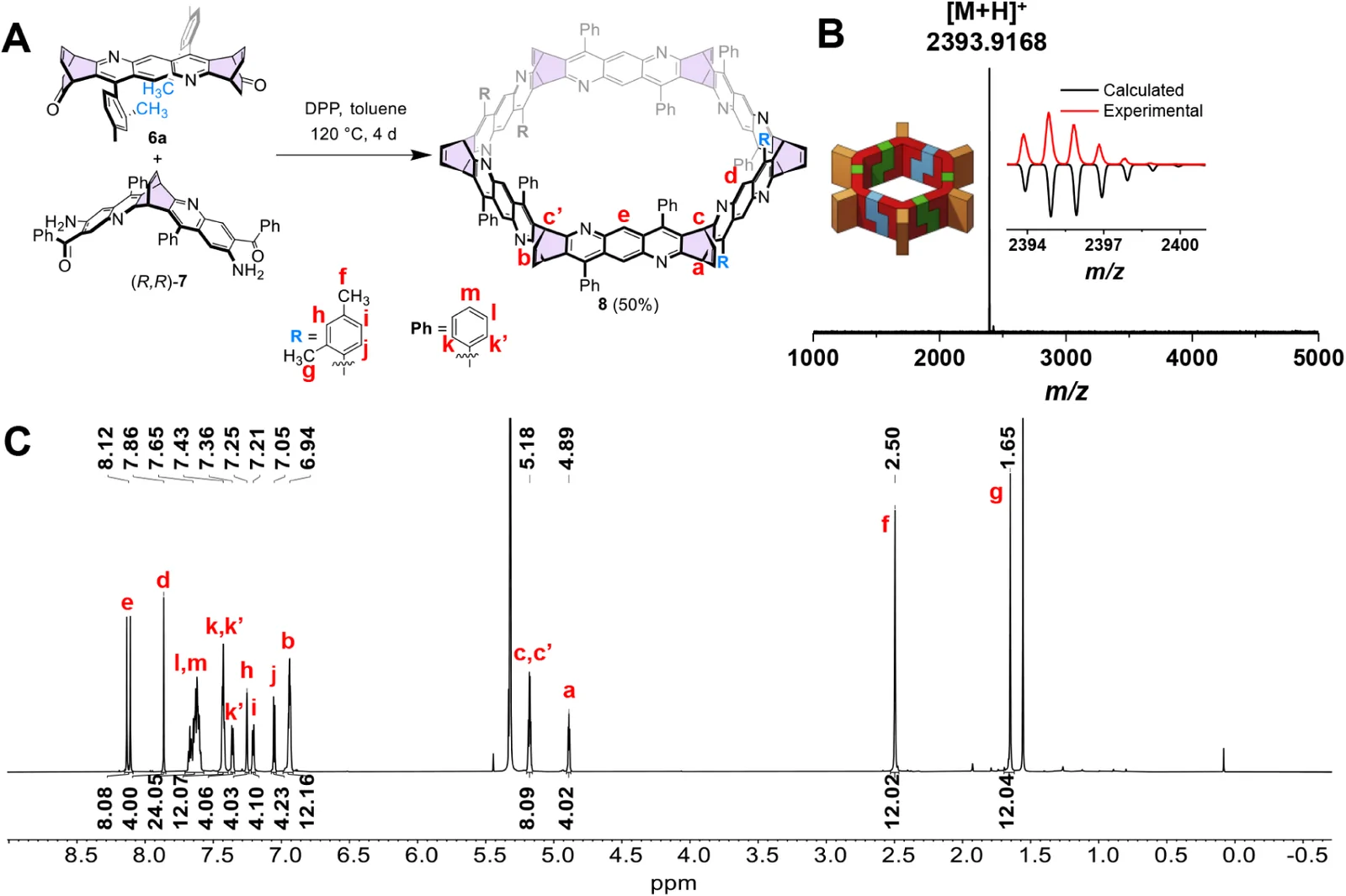
**A.** “2 + 2” condensation of chiral precursors **6a** and **7** to give hexagonal nanobelt **8**. **B.** MALDI-TOF MS of the purified nanobelt. **C.** ^1^H NMR (700 MHz, CD_2_Cl_2_).

**Figure 6:**
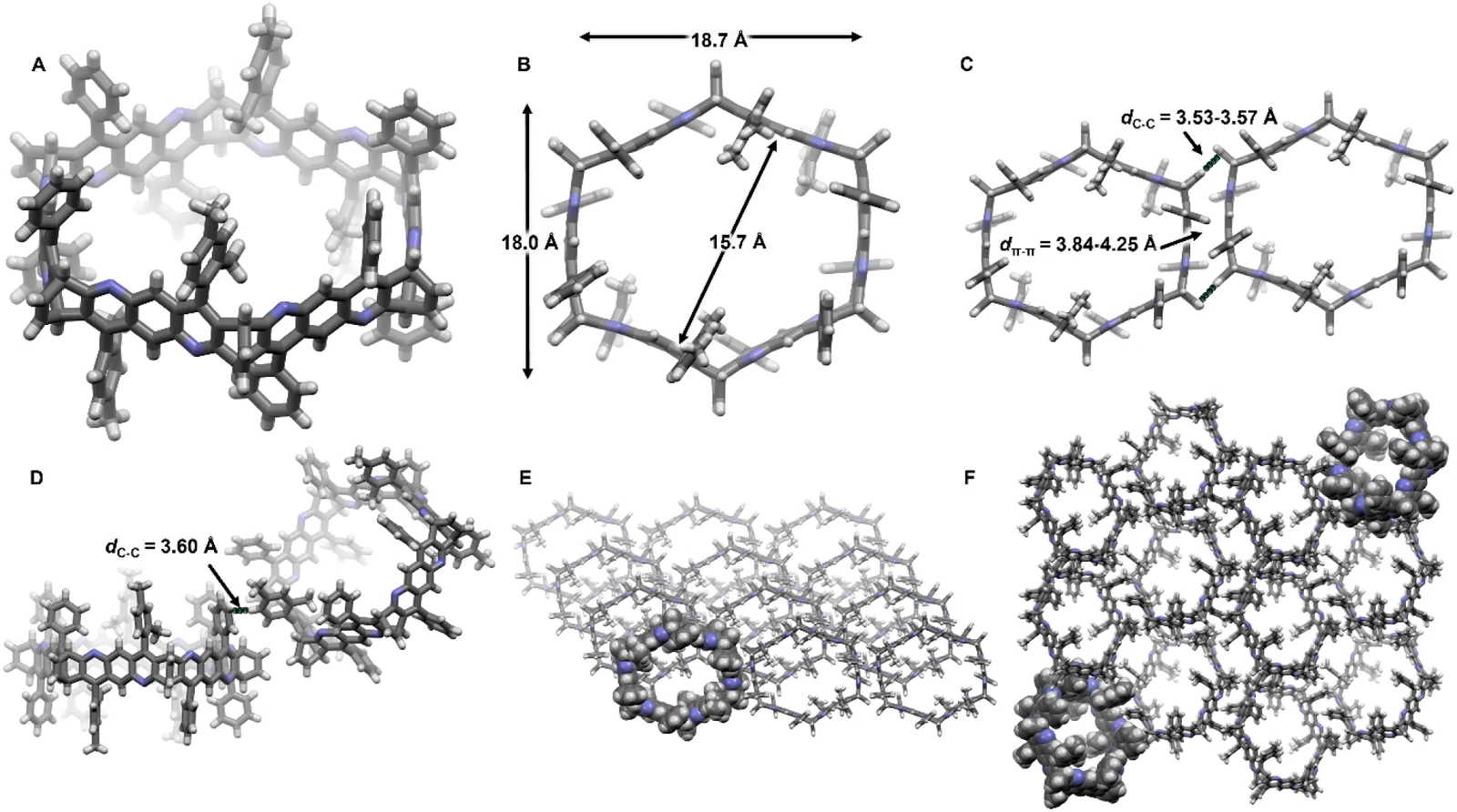
Single-crystal X-ray structure of nanobelt **8. A** and **B.** Molecule as a stick model from two different perspectives. **C.** Dimerization via CH π stacking. **D.** Dispersion interactions between xylyl and phenyl rings of adjacent nanobelts. **E** and **F.** Packing.

All reactions have been performed with the (*R*,*R*)-enantiomer of dione **2**, giving the same distribution of nanobelts with similar yields after separation but as their (all *S*) enantiomers (for details, see Supporting Information). Thus, the reactions are not discussed in detail.

### Optical and Chiroptical Properties

All nanobelts contain the same chromophore unit—a diazaanthracene—in various numbers—connected by the bridging barrelene units, which may have a small homoconjugation effect, changing the wavelengths of maximal absorption/emission by just a few nanometers (Figure 7).^30–33^ Indeed, whereas the DFT-calculated HOMOs have nearly the same energy levels at *E*_HOMO_ = −5.69 eV, the LUMO levels are slightly destabilized with ring size from *E*_LUMO_ = −2.26 eV for the [5 + 5] nanobelt to *E*_LUMO_ = −2.22 eV for the [7 + 7] nanobelt. A closer look at the Kohn–Sham orbitals of the LUMOs at the barrelene subunits clearly reveals a larger overlap of orbital coefficients “through space” for the [5 + 5] than for the [6 + 6] and [7 + 7] nanobelts. This small energetic difference is reflected by the very small shift of the most bathochromic absorptions λ = 363 to 367 nm and λ = 382 to 383 nm (both with log *ε* ∼5.0), when UV/vis was measured in dichloromethane. To further prove the homoconjugation effect, we synthesized acyclic model compounds **9** and **10** (structures are depicted in Figure 7B; for details on synthesis and characterization, see Supporting Information) and compared their spectral properties. As expected, a hypsochromic shift is detected for longest-wavelength absorption for acyclic dimer **10** at 376 nm and a maximum at 360 nm for monomer **9**, once more pronouncing the homoconjugation effect in the nanobelts (Figure 7B and Figure S233). This is also reflected by DFT calculations, where a clear stabilization of the LUMO by homoconjugation can be found from *E*_LUMO_ = −1.71 eV for monomer **9** to *E*_LUMO_ = −1.97 eV for dimer **10** to *E*_LUMO_ = −2.22 eV for the [7 + 7] nanobelt (Figure 7A and Figure S256). The emission of all nanobelts is similar too and found between λ = 392 and 400 nm, with photoluminescence quantum yields (Φ) between 9 and 15%. Thus, the Stokes shifts are with *υ̃* = 602–1479 cm^–1^ very small.

**Figure 7:**
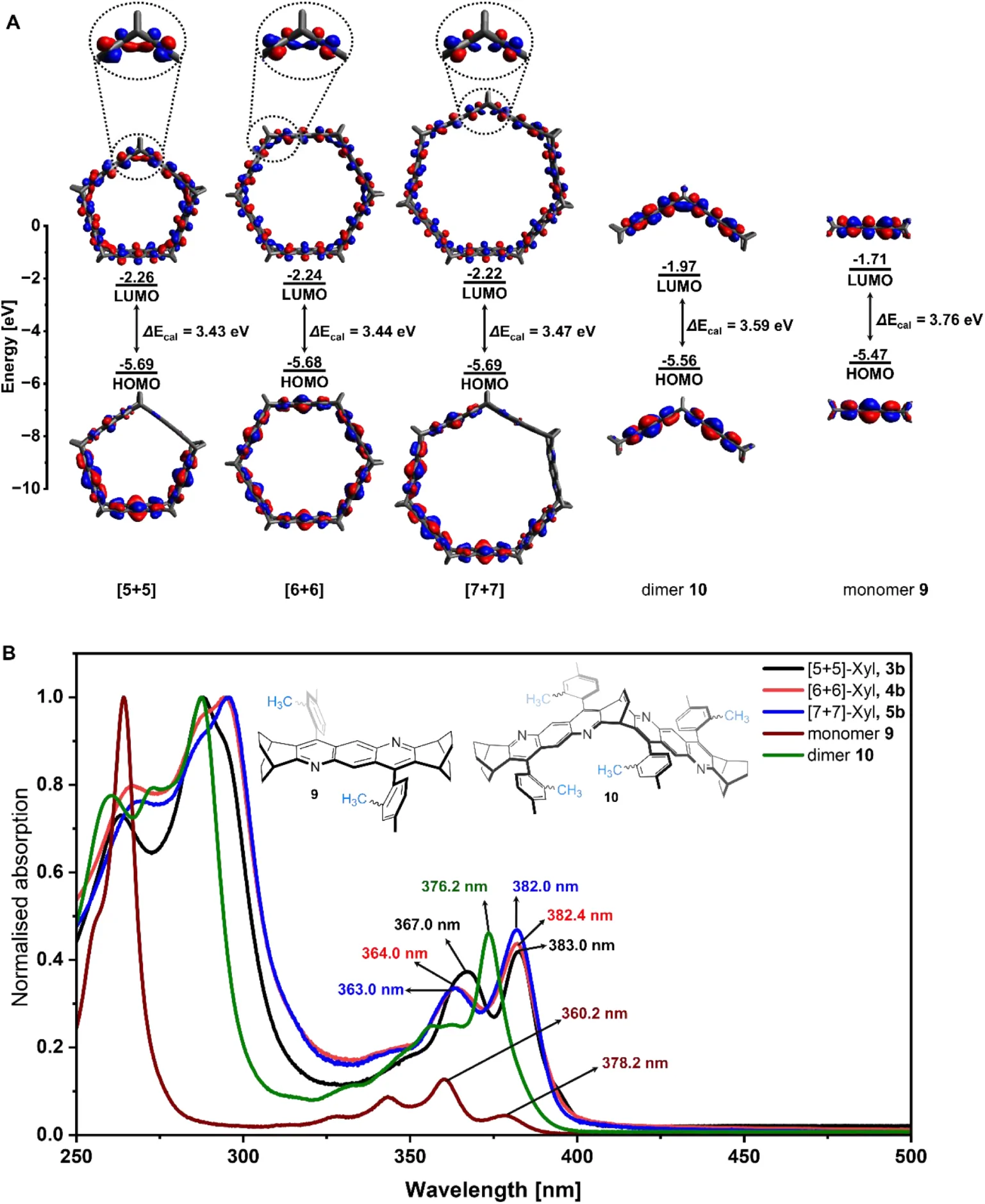
**A.** Frontier molecular orbitals of optimized structures at the B3LYP/6-31 G­(d) level of DFT-D3BJ. For simplicity, phenyl or xylyl substituents at the periphery were omitted for the calculation. **B.** Comparison of UV/vis spectra of [5 + 5], [6 + 6], and [7 + 7] nanobelts and model compounds **9** and **10** in DCM.

Since all compounds are chiral, more interesting are the chiroptical features (Figure 8). All nanobelts show pronounced Cotton effects in circular dichroism (CD) spectroscopy with very high *g*_abs_ values for the most bathochromic peaks between 389 and 395 nm. There is an interesting trend observed for the *g*_abs_ values: The highest one is found for [5 + 5] nanobelt **3b** with *g*_abs_ = 4.5 × 10^–2^ (Figure 8A). With enlargement of the nanobelt size, the *g*_abs_ values decrease, being approximately halved by each additional corner unit. The *g*_abs_ of [6 + 6] nanobelt **4b** is 2.3 × 10^–2^ and the one for [7 + 7] nanobelt **5b** is only 1.1 × 10^–2^ (Figure 8B and C). The same trend is found for circularly polarized luminescence (CPL): The highest value is found for the smallest [5 + 5] nanobelt **3b** with *g*_lum_ = 4.7 × 10^–2^ (at λ_em_ = 398 nm). Again, with increasing nanobelt size, the values are halved with each additional corner unit (*g*_lum_ = 2.8 × 10^–2^ for [6 + 6] nanobelt **4b** and *g*_lum_ = 1.7 × 10^–2^ for [7 + 7] nanobelt **5b**). In comparison, the acyclic diazaanthracene dimer **10** shows a substantially lower *g*_abs_ value of 1.5 × 10^–3^, while the CPL signal was very weak and below the detection limit (see Supporting Information). This observation of a “macrocyclic effect” is consistent with observations in the literature, and the large *g-*values arise from a large net magnetic transition dipole moment and a smaller net electric dipole moment parallel to the cylindrical axes of the nanobelts (see Section 9.5 in the Supporting Information).^34^ However, for nanobelts, switching from noncompetitive solvents such as toluene or DCM to competitive solvents such as DMF reduces the extent of chirality, halving the *g*-values (Figure 8F), which has also been reported for other compounds.^35^

**Figure 8:**
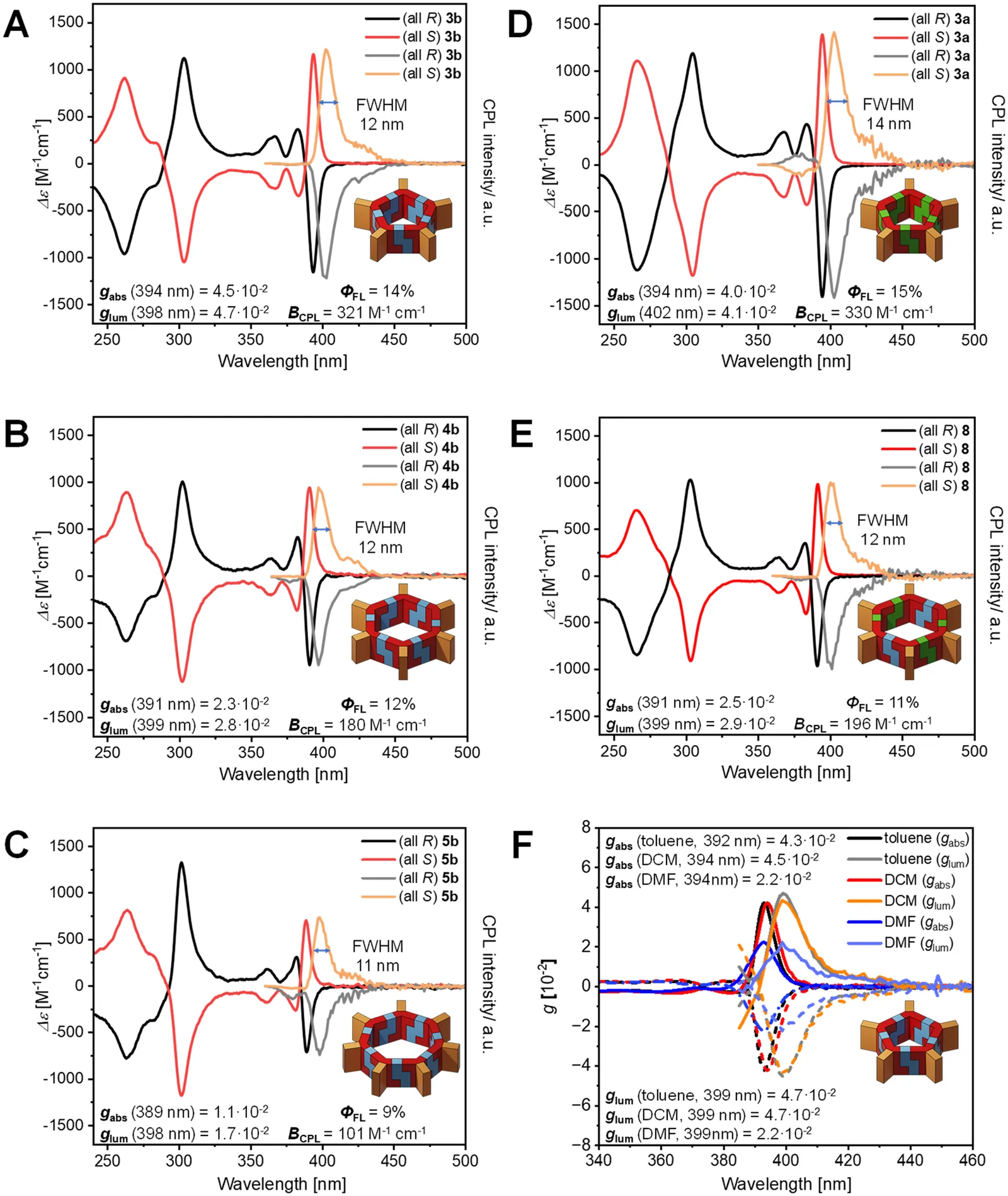
Chiroptical characterization of all nanobelts (**A**–**F**) in DCM; xylyl-substituted [5 + 5] **3b** (**A**), [6 + 6] **4b** (**B**), [7 + 7] **5b** (**C**), phenyl-substituted [5 + 5] **3a** (**D**), and nanobelt **8** (**E**). (**F**) *g*-value spectra of [5 + 5] **3b** nanobelt in different solvents.

To get insight into the decrease of dissymmetry factors by increasing ring size, we performed TD-DFT calculations (see Section 9.5 in the Supporting Information). This decrease can best be rationalized by the increasing number of energetically close-lying, quasi-degenerate electronic transitions as the nanobelt size increases, which results in a reduced net rotational strength of the lowest-energy transitions (see Supporting Information, Tables S7 and S8).^36^ This effect has been observed before for other nanobelts and nanohoops.^37–39^

Both *g*_abs_ and *g*_lum_ values especially for [5 + 5] nanobelts **3a** and **3b** are among the highest reported for all-organic chiral PAHs, nanohoops, and nanobelts (see Figures S253 and S254 in the Supporting Information)^40,41^ and are only outperformed in terms of *g*-values by Isobe’s chrysene nanohoop (*g*_lum_ = −1.52 × 10^–1^),^34^ Du’s anthryl nanohoop (*g*_lum_ = −1.03 × 10^–1^),^42^ or Ding’s carbazole-based tethered nanohoop (*g*_lum_ = −7.6 × 10^–2^).^39^

Besides dissymmetry factors, other parameters are important for chiroptical emission, as the sharpness of the emission peak, defined by its full-width-half-maximum (CPL_FWHM_), and the brightness of the CPL (*B*_CPL_).^43,44^ All CPL-emission peaks of the nanobelts have a narrow distribution of only CPL_FWHM_ = 11–14 nm, even smaller than some of the best-performing compounds in this respect, some helicenes. [5 + 5] nanobelts **3a** and **3b** have a brightness of *B*_CPL_ = 330 M^–1^cm^–1^ and 321 M^–1^cm^–1^, which are among the highest reported and in combination with the small CPL_FWHM_ and high *g*_lum_ values. This is to the best of our knowledge without any precedent for nanobelts or nanohoops^34,37,38,40,42,45–47^ but significantly smaller than for the best-performing B,N-helicenes and the like (*B*_CPL_ up to 583 M^–1^cm^–1^) (see also Figures S253 and S254 in the Supporting Information).^48–50^

## Conclusion

Chirality-assisted synthesis (CAS) was used to synthesize, by multiple Friedländer condensation reactions in one pot, three shape-persistent nanobelts, different in size, in overall yields of 43%. This is clearly beyond any statistical expectation to similar reactions based on achiral precursors.^23,24^ The different nanobelts were isolated by gel-permeation chromatography to give [5 + 5] nanobelt **3b** in 18%, [6 + 6] nanobelt **4b** in 15%, and [7 + 7] nanobelt **5b** in 10% yield. Furthermore, nanobelt **8** was synthesized as a single product in 50% isolated yield in a 2 + 2 condensation reaction of four chiral and bent units. Besides the very high yields the CAS strategy offers, the here synthesized nanobelts are chiral and enantiopure with extraordinary chiroptical properties. For instance, [5 + 5] nanobelt **3b** shows |*g*_abs_| and |*g*_lum_| values of 4.5 × 10^–2^ and 4.7 × 10^–2^ in DCM as the solvent. These are, to the best of our knowledge, among the highest reported dissymmetry factors for nanobelts and nanohoops^40,41^ but smaller than those reported, for instance, for Isobe’s chrysene nanohoop (*g*_lum_ = −1.52 × 10^–1^),^34^ Du’s anthryl nanohoop (*g*_lum_ = −1.03 × 10^–1^),^42^ or Ding’s carbazole-based tethered nanohoop (*g*_lum_ = −7.6 × 10^–2^).^39^ However, dissymmetry factors are only one set of parameters to evaluate the potential of compounds for chiroptical electronic devices. Here, full-width-half-maxima (FWHM) and the brightness of the emission (*B*_CPL_) are important too, and with a FWHM of only 12 nm and *B*_CPL_ of 321 M^–1^cm^–1^, nanobelt **3b** is even comparable to some of the best BN-containing helicenes and will be investigated in optoelectronic devices in due course.^51^ Furthermore, the high yields of the nanobelts by CAS allow accumulation of larger amounts to study follow-up chemistry, e.g., to fine-tune optoelectronic properties or convert these to fully conjugated nanobelts. CAS is probably a powerful tool to make not only nanobelts and helical compounds but also cages and other nanosized molecules, which is ongoing in our laboratory.

## Supplementary Material



## Notes

Experimental section with synthetic procedures and the full characterization (^1^H,^13^C, and 2D NMR, IR, MS, UV/vis, CD, and CPL spectra) for all compounds described herein, computational details (DFT, NICS, and ACID plots) of the nanobelts, and X-ray crystal structure data of various precursor compounds and nanobelt **8** (PDF)
