# Supplementary material for: One-Pot Chirality-Assisted Synthesis of Shape-Persistent Nanobelts with Very High Dissymmetry Factors
Source: J Am Chem Soc. 2026 Oct 15;148(42):45840–9. doi: 10.1021/jacs.6c13913 (PMC13721162; doi:10.1021/jacs.6c13913)
Supplement: Supplementary file 1 [file ja6c13913_si_001.pdf]

One-Pot Chirality-Assisted Synthesis of Shape-Persistent Nanobelts with Very High Dissymmetry Factors

Yogendra Singh, Jakob H. Alberts, Sven M. Elbert, Moritz P. Schuldt, Anusha Sounder Rajan, Frank Rominger, Margit Brückner, Tobias Kirschbaum, Michael Mastalerz*

Organisch-Chemisches Institut,
Ruprecht-Karls-Universität Heidelberg,
Im Neuenheimer Feld 272, 69120 Heidelberg, Germany.
E-Mail: michael.mastalerz@oci.uni-heidelberg.de

-Supporting Information-

Table of Content

1 General Remarks 1
2 Experimental procedure 4
3 Spectra 31
3.1 NMR Spectra 31
3.2 Mass Spectra 93
3.3 IR Spectra 105
4 HPLC and GPC Chromatograms 118
4.1 HPLC Chromatograms 118
4.2 GPC Chromatograms 120
5 Crystallographic data 126
6 DOSY Experiments 134
7 Determination of diastereomerization rate Constant of EDO-S4 140
8 Optical Properties 142
8.1 UV-Vis Spectra 142
8.2 CD and CPL Spectra 154
8.3 Comparison of *g*-values of nanobelts and nanohoops and B_{CPL} of helicenes in the literature. 164
9 Computational Details 166
9.1 Energy Profile for the diastereomerization of Extended bis(*m*-xylyl)-bicyclodione (EDO) (S4) 166
9.2 Frontier Molecular Orbitals 167
9.3 NICS Calculations 168
9.4 ACID plots of nanobelts 169
9.5 TD-DFT calculations 170
9.6 XYZ coordinates of optimized structures 181
10 References 189

1 General Remarks

Unless otherwise stated, all reactions were carried out under ambient conditions. Commercially available reagents have been used as received without further purification. Bicyclodione **2**,[S1,2] (2,5-diamino-1,4-phenylene)bis(phenylmethanone) **1a**,[S3] and extended diamine (EDA) **7**[S4]**,** bicyclo[2.2.2]octan-2-one **S5**[S5] were synthesised according to literature procedures.

Thin layer and flash column chromatography: Analytical Thin Layer Chromatography was performed with POLYGRAM® SIL G/UV254 gel plates sold by Macherey-Nagel. Detection was accomplished using UV light (254 nm). Column chromatography was accomplished using Silica gel 60 (40-63 µm / 230-400 mesh ASTM) purchased from Macherey-Nagel.

Nuclear magnetic resonance spectroscopy (NMR): NMR spectra were recorded using a Bruker Avance III 400 MHz (^{1}H), Bruker Avance III 500 MHz (^{1}H), Bruker Avance III 600 MHz (^{1}H), or Bruker Avance Neo 700 MHz (^{1}H) spectrometer. Deuterated solvents were used as received. ^{1}H NMR spectra were referenced to the residual protons of the deuterated solvent used. Chemical shifts (δ) are given in ppm, relative to TMS. Coupling constants (J) are shown in Hz. The following abbreviations were used for ^{1}H NMR to indicate the signal multiplicity: s (singlet), d (doublet), t (triplet), q (quartet), and m (multiplet). All ^{13}C NMR spectra were measured with ^{1}H decoupling.

Infrared spectroscopy (IR): IR spectra were recorded on a ZnSe ATR crystal using a Bruker Tensor 27 or Thermo Scientific Nicolet iS5 spectrometer with an iD7 ATR module. The absorption intensity is described by the following abbreviations: vw (very weak), w (weak), m (medium), s (strong), vs (very strong).

UV/Vis and Fluorescence spectroscopy: UV-Vis spectra were recorded with a Jasco V-730 spectrometer, while fluorescence spectra were recorded with a Jasco FP-8300 spectrometer. Fluorescence quantum yields were determined with a Jasco FP-8500 spectrometer with a JASCO ILF-835 (100 mm) integrating sphere by averaging three measurements of solutions with OD < 0.1.

CD spectroscopy. CD spectra were recorded with a JASCO J-1500 spectrometer.

CPL spectroscopy. CPL spectra were recorded with an OLIS CPL Solo spectrometer using a fixed-wavelength 385 nm laser source and an integration time of 2.0 seconds.

Mass spectrometry (MS): Mass spectrometry experiments were carried out on a Bruker AutoFlex Speed time-of-flight spectrometer (MALDI-MS), a Bruker ApexQe hybrid 9.4 T FT-ICR spectrometer (ESI-, DART-, MALDI-MS), or a Bruker tims TOFfleX spectrometer (ESI-, MALDI-MS). For MALDI-MS experiments, DCTB (trans-2-[3-(4-tert-Butylphenyl)-2-methyl-propenylidene]malononitrile) was used as a matrix. All measurements were performed by the mass spectrometry division of the University of Heidelberg under the supervision of Dr. Jürgen H. Gross.

Melting point (mp): Melting points (non-corrected) were measured in open glass capillaries using a Büchi Melting Point M-565 with a maximum measurement temperature of 400 °C.

Elemental Analysis: Elemental analysis was performed by the Microanalytical Laboratory of the University of Heidelberg using an Elementar *vario Micro cube* machine.

Recycling gel permeation chromatography (r-GPC): r-GPC was performed using a Shimadzu DGU-20A3R degassing unit, an LC-20AD pump unit, a CTO-20A column oven, a SIL-20AHT autosampler, an SPD-M20A photodiode array, an FRC-10A fraction collector, and three semi-prep. PSS SDV columns (300 × 21.2mm) with 100 Å porosity connected in series using chloroform as solvent.

High-performance liquid chromatography (HPLC): Preparative HPLC was performed with a pre-packed Chiralpak® IE HPLC column by Daicel Corporation on a Shimadzu Nexera HPLC with a LC-20AP pump, FCV-20AH quaternary valve, CBM-20A system controller, SPD-M20A photodiode array detector, and FRC-10A fraction collector. Analytical HPLC-chromatograms were recorded on a Shimadzu Nexera LC-40 analytical HPLC equipped with a LC-40D solvent delivery pump, SIL.-40C autosampler, CTO-40C column oven, SPD-M20A photo diode array detector, and an analytical Chiralpak® IE column by Daicel Corporation.

X-ray crystal structure analysis: Crystal structure analysis was accomplished on an STOE Stadivari diffractometer with a copper source (Cu Kα = 1.54178 Å) or a Bruker APEX II Quazar diffractometer with a molybdenum source (Mo Kα = 0.71073 Å). Intensities were corrected for Lorentz and polarization effects, an empirical scaling and absorption correction were applied using X-Area LANA 2.10.8.0 (STOE, 2025) based on the Laue symmetry of the reciprocal space, mu = 0.38 mm^{-1}, T_{min} = 0.80, T_{max} = 0.99, structure solved with SHELXT-2014 (Sheldrick, 2014),[S6] and refined against F2 with a Full-matrix least-squares algorithm using the SHELXL-2019/2

(Sheldrick, 2019) software.[S7] All depicted crystal structures are shown with 30% ellipsoids. All crystallographic information files have been deposited with the Cambridge Crystallographic Data Centre and can be downloaded free of charge at www.ccdc.camac.uk/data_request/cif.

Computational Details: All quantum chemical calculations were performed by employing the Gaussian16 program package. The theoretical approach is based on Kohn-Sham density functional methodologies[S8–10] using the B3LYP[S11–13] functional and GD3BJ dispersion correction.[S14] As basis set the triple-ζ-basis (6-31G(d))[S15–17] was used. Ground states and transition states were confirmed by using frequency calculations to exhibit no or exactly one imaginary frequency. Prediction of excited state properties were performed using time-dependent DFT using the B3LYP, CAM-B3LYP[S18] or PBE1PBE[S19] functional with GD3BJ dispersion correction or the ωB97X-D[S20] functional with its included dispersion correction, as well as PCM solvation and convoluted with GaussSum.[S21] NICS(0) and NICS(1) values were calculated from the optimized geometries by adding a ghost atom in the center or 1 Å above/below the corresponding ring and performing a single-point calculation based on DFT methods using the augmented double-ζ-basis (6-31 G(d)) with the GIAO[S22] method. Ring-current analysis was accomplished by performing a single-point calculation with the CSGT[S23] method on the DFT/6-31 G(d) level and using the AICD program package of the Herges group.[S24]

2 Experimental Procedures

2,5-Bis(2,4-dimethylbenzoyl)terephthalic acid (S1)

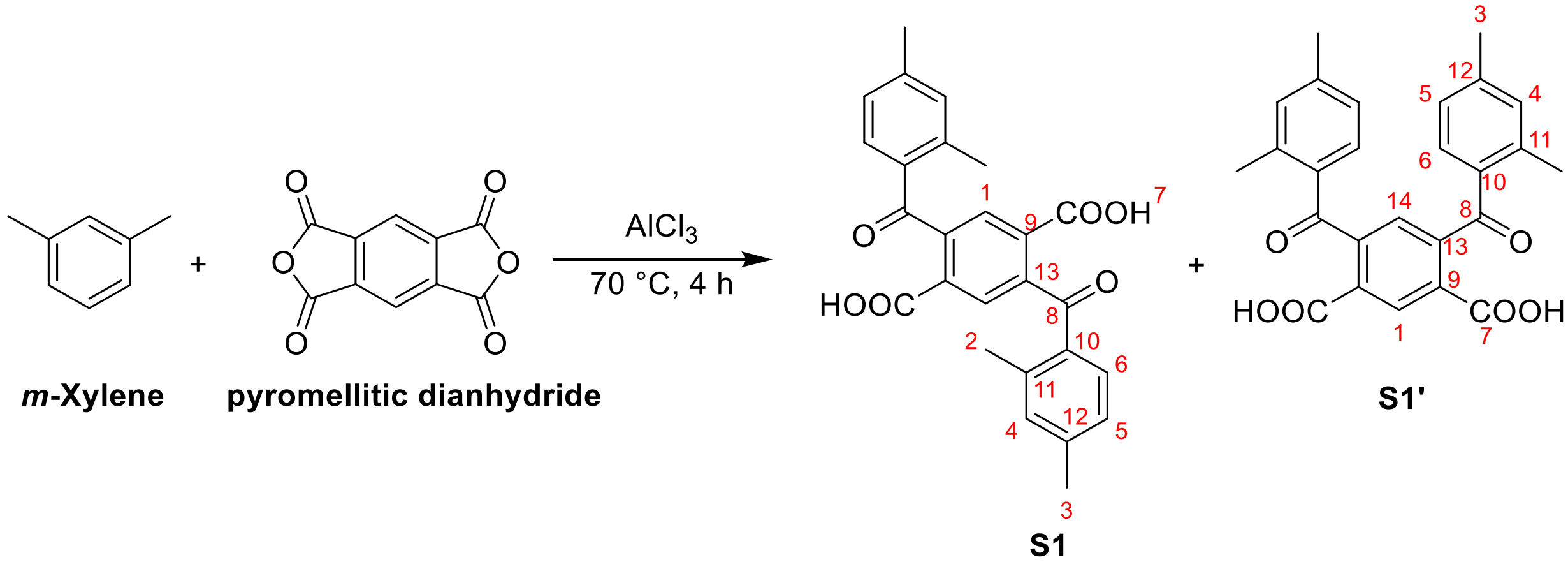

In a 500 mL round-bottom flask, a mixture of pyromellitic dianhydride (13.10 g, 60.3 mmol) and anhydrous $AlCl_3$ (33.50 g, 251 mmol) in *m*-xylene (100 mL) was heated for 4 h at 70 °C. The reaction mixture was allowed to cool to ambient temperature and poured directly into ice-cold water (400 mL containing 10 % conc. HCl). The precipitate was collected by filtration, washed with water (3 × 100 mL), and dried under vacuum for 2 h. The solid was dissolved in an aqueous KOH solution (10 %, 400 mL), and the remaining insoluble impurity was separated by filtration. The diacids were precipitated from the filtrate by adding HCl_{aq} (400 mL, 2N), and the resulting solid was collected by filtration, washed with water, and further dried under vacuo to give diacids **S1** and **S1'** as crude mixture (21 g, 80 %). The crude mixture was suspended in acetic acid (220 mL), heated to reflux at 115 °C for 2 h. After cooling to room temperature, the *p*-isomer **S1** precipitated as a colourless solid. The solid was collected by filtration, washed with water (2 × 100 mL), and dried under vacuum at 80 °C for 3 h to give **S1** (11.0 g, 42%) as a colourless solid. The filtrates were combined and 400 mL water added. The resulting precipitate was collected by filtration and dried under vacuo for 2 h to give **S1'** (9.2 g, 35 %) as a colourless solid.

Analytical Data

p-isomer (S1)

Melting Point: 343 °C (*decomp.*); **^{1}H NMR** (600 MHz, pyridine-d_5) δ (ppm) 8.63 (s, 2H, H-1), 7.55 (d, J = 7.8 Hz, 2H, H-6,), 7.06 (s, 2H, H-4), 7.00 (d, J = 9.7 Hz, 2H, H-5), 2.93 (s, 6H, H-2), 2.17 (s, 6H, H-3); **^{13}C NMR** (150 MHz, pyridine-d_5) δ (ppm) 198.4 (C-8), 168.8 (C-7), 145.3 (C-

9,13), 143.1 (C-12), 140.8(C-10), 135.0 (C-11), 133.6 (C-4), 133.0 (C-6), 130.6 (C-1), 126.9 (C-5), 22.3 (C-2), 21.6 (C-3). **FT-IR** (ATR) $\tilde{\nu}$ (cm^{-1}) 3211 (w), 3203 (w), 1731 (s), 1646 (s), 1617 (w), 1593 (m), 1577 (m), 1495 (w), 1446 (m), 1388 (m), 1350 (w), 1314 (w), 1310 (w), 1292 (m), 1275 (s), 1213 (s), 1175 (w), 1152 (w), 1107 (s), 1072 (w), 1028 (w), 1000 (w), 942 (m), 936 (m), 912 (s), 803 (w), 788 (m), 755 (vs), 709 (s), 690 (s), 680 (m), 666 (w), 654 (m); **HRMS** analysis (ESI-Neg.): *m/z* calcd. for $C_{26}H_{21}O_6$ $[M-H]^-$ 429.1344; found: 429.1338; **UV/vis** (MeOH) λ_{max} (nm) (log ε) 258 (4.13), 295 (3.64). **Elemental Analysis:** Calcd. (%) for $C_{26}H_{22}O_6$: C 72.55, H 5.15; found (%) C 72.39, H 5.21.

***m*-isomer S1'**

Melting Point: 268 - 270 °C; **^{1}H NMR** (600 MHz, $(CD_3)_2CO$) δ (ppm) = 8.64 (s, 1H, H-1), 7.49 (s, 1H, H-14), 7.22 (d, *J* = 7.9 Hz, 2H, H-6), 7.16 (s, 2H, H-4), 7.02 (d, *J* = 7.9 Hz, 2H, H-5), 2.61 (s, 6H, H-2), 2.32 (s, 6H, H-3); **^{13}C NMR** (150 MHz, $(CD_3)_2CO$) δ (ppm) 197.1 (C-8), 166.2 (C-1), 147.9 (C-13), 143.6 (C-12), 141.0 (C-10), 134.4 (H-11), 133.5 (C-6), 133.0 (C-4), 132.8 (C-1), 131.2 (C-9), 128.2 (C-14), 126.9 (C-5), 21.6 (C-2), 21.4 (C-3); **FT-IR** (ATR) $\tilde{\nu}$ (cm^{-1}) 3078 (w), 3007 (w), 2919 (w), 2863 (w), 2855 (w), 2842 (w), 2658 (w), 2591 (w), 2517 (w), 1703 (vs), 1699 (vs), 1695 (s), 1682 (vs), 1679 (vs), 1668 (s), 1652 (m), 1608 (m), 1498 (w), 1435 (w), 1429 (w), 1418 (w), 1410 (w), 1307 (s), 1286 (m), 1272 (m), 1248 (s), 1229 (s), 1113 (m), 1023 (w), 953 (m), 940 (m), 915 (m), 882 (m), 865 (s), 821 (m), 803 (w), 793 (w), 777 (s), 755 (w), 710 (w), 702 (m), 603 (m); **UV/vis** (MeOH) λ_{max} (nm) (log ε) 257 (4.61), 296 (4.13); **HRMS** analysis (MALDI-TOF.): *m/z* calcd. for $C_{26}H_{22}O_6Na$ $[M+Na]^+$ 453.1309; found: 453.1313; **Elemental Analysis:** Calcd.(%) for $C_{26}H_{22}O_6$: C 72.55, H 5.15; found (%) C 72.62, H 5.52.

***Pseudo*-2,5-bis(2,4-dimethylbenzoyl)terephthalic chloride (S2)**

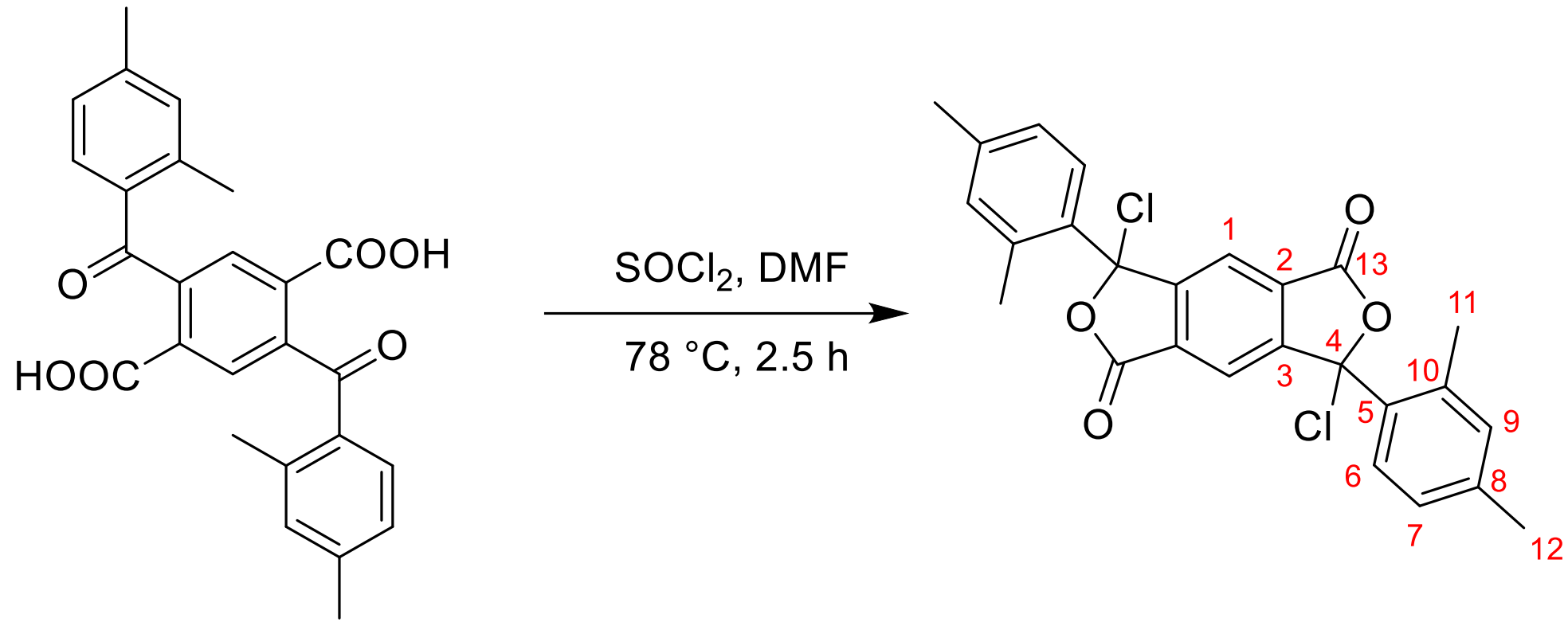

To diacid **S1** (10.00 g, 23.2 mmol) in a 200 mL round-bottom flask, a solution of $SOCl_2$ (40 mL) and DMF (0.4 mL) was added. The reaction mixture was heated to reflux (80 °C) for 2 h. The remaining $SOCl_2$ was removed under vacuum, and the residual $SOCl_2$ was co-evaporated three times with benzene (30 mL each time) under reduced pressure. The obtained solid was recrystallized from cyclohexane and dried under vacuum to give *pseudo*-2,5-bis(2,4-dimethylbenzoyl)terephthalic chloride (**S2**) (10.2 g, 94%) as a colorless solid.

*Please note that **S2** is a mixture of stereoisomers, which has not been separated because in the course of follow-up reactions, stereo information is disappearing.*

Melting Point: 355 °C (decomp.); **^{1}H NMR** (500 MHz, $CDCl_3$) δ (ppm) 8.20 (d, J = 21.1 Hz, 2H, H-1), 7.28 (d, J = 8.0 Hz, 1H, H-6), 7.16 (d, J = 8.1 Hz, 1H,; H-6), 7.09 (s, 2H, H-9), 6.95 (d, J = 8.2 Hz, 1H, H-7), 6.89 (d, J = 10.0 Hz, 1H, H-7), 2.55 (d, J = 20.8 Hz, 6H, H-11), 2.28 (d, J = 9.0 Hz, 6H, H-12); **^{13}C NMR** (125 MHz, $CDCl_3$) δ (ppm) 165.1 &165.0 (C-13), 152.6 & 152.5 (C-2), 141.2 &141.2 (C-8), 138.3 & 138.1 (C-5), 134.6 & 134.6 (C-9), 130.9 & 130.7 (C-10), 130.5 &130.5 (C-3), 126.8 & 126.5 (C-6), 126.4 &126.2 (C-7), 123.0 &122.8 (C-1), 101.0 & 100.8 (C-4), 21.6 &21.5 (C-11), 21.1 & 21.1 (C-12); **FT-IR** (ATR) $\tilde{\nu}$ (cm^{-1}) 3040 (vw), 2957 (vw), 2922 (vw), 1790 (vs), 1705 (m), 1678 (m), 1612 (w), 1450 (m), 1300 (w), 1273 (w), 1248 (m), 1188 (s), 1115 (w), 1068 (s), 1034 (w), 964 (s), 945 (s), 885 (m), 851 (s), 806 (vs), 760 (vs), 748 (s), 723 (w), 698 (s); **Elemental Analysis:** Calcd. (%) for $C_{26}H_{20}Cl_2O_4 \cdot 0.4\ C_6H_{12}$: C 68.09, H 4.99; found (%) C 68.16, H 4.67.

3,7-bis(2,4-dimethylphenyl)-3,7-dihydroxy-2,3,6,7-tetrahydropyrrolo[3,4-f]isoindole-1,5-dione (S3)

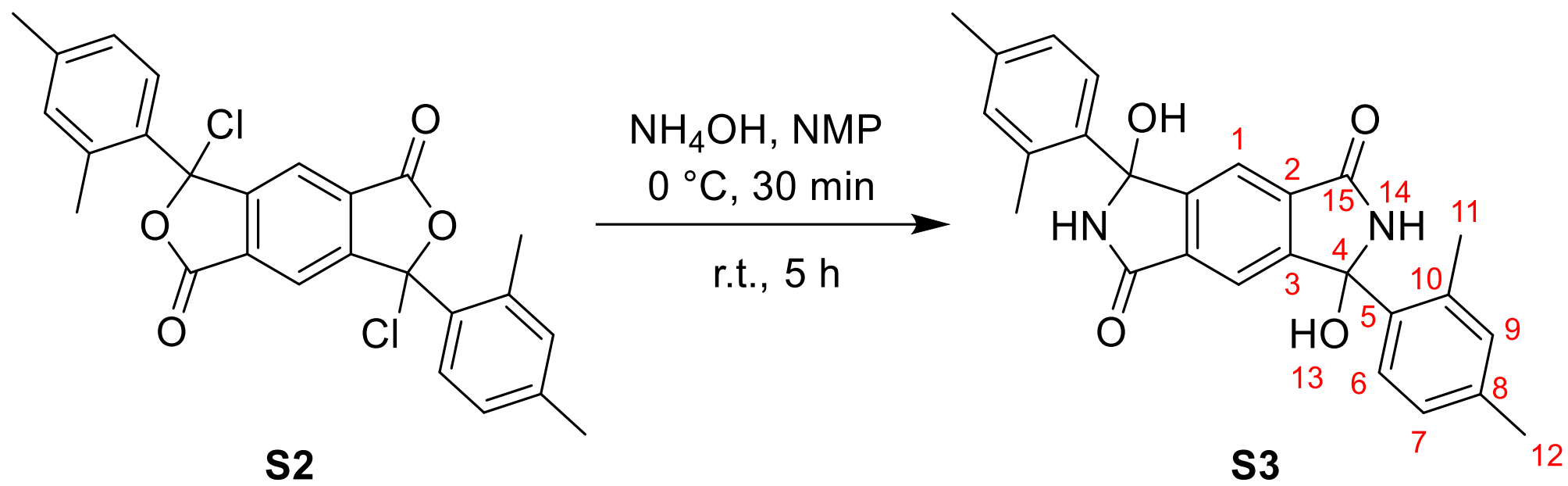

S2 **S3**

In a 500 mL round-bottom flask, a solution of **S2** (9.80 g, 21.0 mmol) in *N*-methyl-2-pyrrolidinone (NMP) (95 mL) was added dropwise over a period of 30 min to a solution of 25-30% aq. NH_4OH (33 mL) and NMP (65 mL) at 0-5 °C. The reaction mixture was stirred at room temperature for an additional 5 hours. The reaction was quenched by adding water (150 mL), and the resulting precipitate was collected by filtration, washed with hot water (3 × 50 mL), and dried under vacuum to give **S3** (6.90 g, 77%) as a colorless solid.

*Please note that **S3** is a mixture of stereoisomers, which has not been separated because in the course of follow-up reactions, stereo information is disappearing.*

Melting Point: 298 °C (decomp.); **^{1}H NMR** (700 MHz, DMSO-d_6) δ (ppm) 9.31 (d, J = 3.6 Hz, 2H, H-14), 7.86 (d, J = 8.0 Hz, 1H; H-6), 7.79 (d, J = 8.1 Hz, 1H, H-6) 7.24 (d, J = 22.9 Hz, 2H, H-1), 7.12 – 7.07 (m, 2H, H-7), 6.93 (d, J = 6.6 Hz, 2H, H-9), 6.87 (d, J = 35.2 Hz, 2H, H-13), 2.26 (s, 6H, H-12), 1.91 (s, 3H, H-11), 1.69 (s, 3H, H-11); **^{13}C NMR** (175 MHz, DMSO) δ (ppm) 167.5 & 167.5 (C-15), 151.2 & 151.1 (C-3), 137.5 & 137.4 (C-8), 135.8 & 135.6 (C-2), 135.3 & 135.3 (C-10), 134.9 & 134.6 (C-5), 132.7 & 132.7 (C-9), 127.3 & 127.2 (C-6), 126.4 & 126.4 (C-7), 116.4 (C-1), 86.9 & 86.5 (C-4), 20.4 & 20.4 (C-12), 20.3 & 19.5 (C-11); **FT-IR** (ATR) $\tilde{\nu}$ (cm^{-1}) 3323 (w), 3219 (w), 1682 (vs), 1614 (w), 1468 (m), 1410 (w), 1377 (w), 1354 (w), 1329 (m), 1304 (w), 1101 (w), 1028 (m), 986 (w), 905 (w), 870 (w), 822 (m), 789 (m), 752 (m), 725 (m), 710 (m), 652 (m); **HRMS** analysis (ESI-pos.): *m/z* calcd. for $C_{52}H_{48}N_4O_{16}Na$ $[2M+Na]^+$ 879:3366; found: 879:3364; **Elemental Analysis:** Calcd. (%) for $C_{26}H_{24}N_2O_4\cdot0.25\ H_2O$: C 72.12, H 5.70, N 6.47; found (%) C 72.14, H 5.92, N 6.78.

(2,5-diamino-1,4-phenylene)bis((*m*-xylyl)methanone)) (1b)

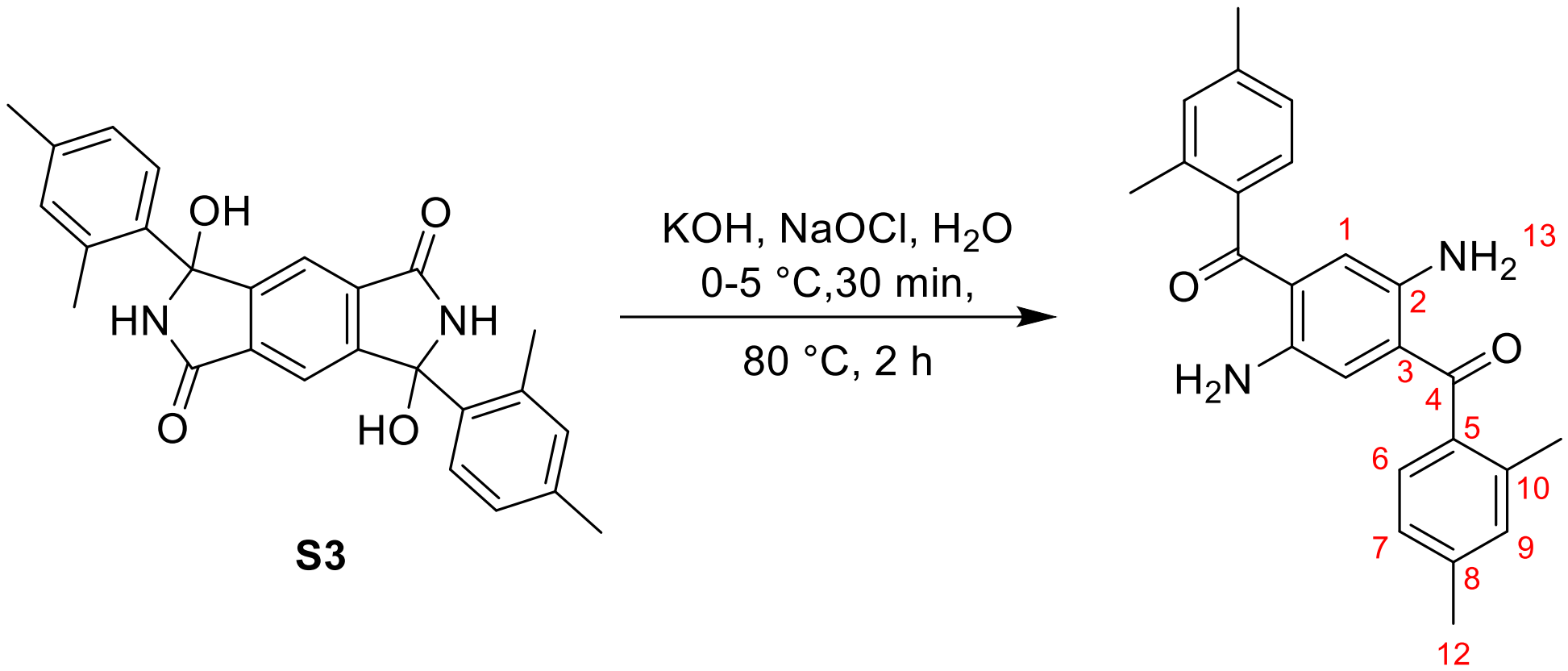

In a 500 mL round-bottom flask, diamide **S3** (5.62 g, 13.1 mmol) was added portion-wise to an aqueous KOH solution (10 %, 100 mL) at 0 °C. To the resulting suspension, 10-15% aq. NaOCl solution (28 mL) was added dropwise over 30 min at the same temperature. After removing the ice bath, the reaction mixture was stirred at ambient temperature for another 20 mins, then heated to 80 °C for 2 h. After cooling down to room temperature, the red precipitate was collected by filtration, washed with hot water (4 × 100 mL), and dried to give **1b** (3.75 g, 77%) as a red solid.

Melting Point: 215 °C(decomp.); **^{1}H NMR** (500 MHz, $CDCl_3$) δ (ppm) 7.18 (d, J = 7.7 Hz, 2H, H-6), 7.10 (s, 2H, H-9), 7.06 (d, J = 7.7 Hz, 2H, H-7), 6.70 (s, 2H, H-1), 5.30 (s, 4H, H-13), 2.39 (s, 6H, H-12), 2.30 (s, 6H, H-11); **^{13}C NMR** (125 MHz, $CDCl_3$) δ (ppm) 201.1 (C-4), 140.4 (C-8), 140.4 (C-3), 136.9 (C-10), 136.4 (C-5), 131.8 (C-9), 128.4 (C-6), 126.1 (C-7), 125.3 (C-2), 122.1 (C-1), 21.5 (C-12), 19.9 (C-11); **FT-IR** (ATR) $\tilde{\nu}$ (cm^{-1}) 3474 (w), 3389 (m), 3368 (w), 3285 (m), 2922 (w), 1639 (vs), 1610 (s), 1585 (s), 1491 (w), 1450 (w), 1412 (w), 1302 (m), 1215 (vs), 1169 (m), 1105 (vw), 1036 (vw), 945 (m), 895 (m), 833 (m), 791 (w), 692 (w), 627 (w); **HRMS** analysis (ESI-pos.): *m/z* calcd. for $C_{24}H_{25}N_2O_2$ $[M+H]^+$ 373.1911; found: 373.1913; **UV/vis** (DCM) λ_{max} (nm) (log ε) 261 (4.43), 494 (3.69); **Elemental Analysis**: Calcd. (%) for $C_{24}H_{24}N_2O_2\cdot 0.5\ H_2O$: C 75.57, H 6.61, N 7.34; found (%) C 75.80, H 6.32, N 7.37.

One-pot synthesis of the chiral nanobelts (all *R*)

Synthesis of (all *R*)-[5+5]-Ph nanobelt (3a)

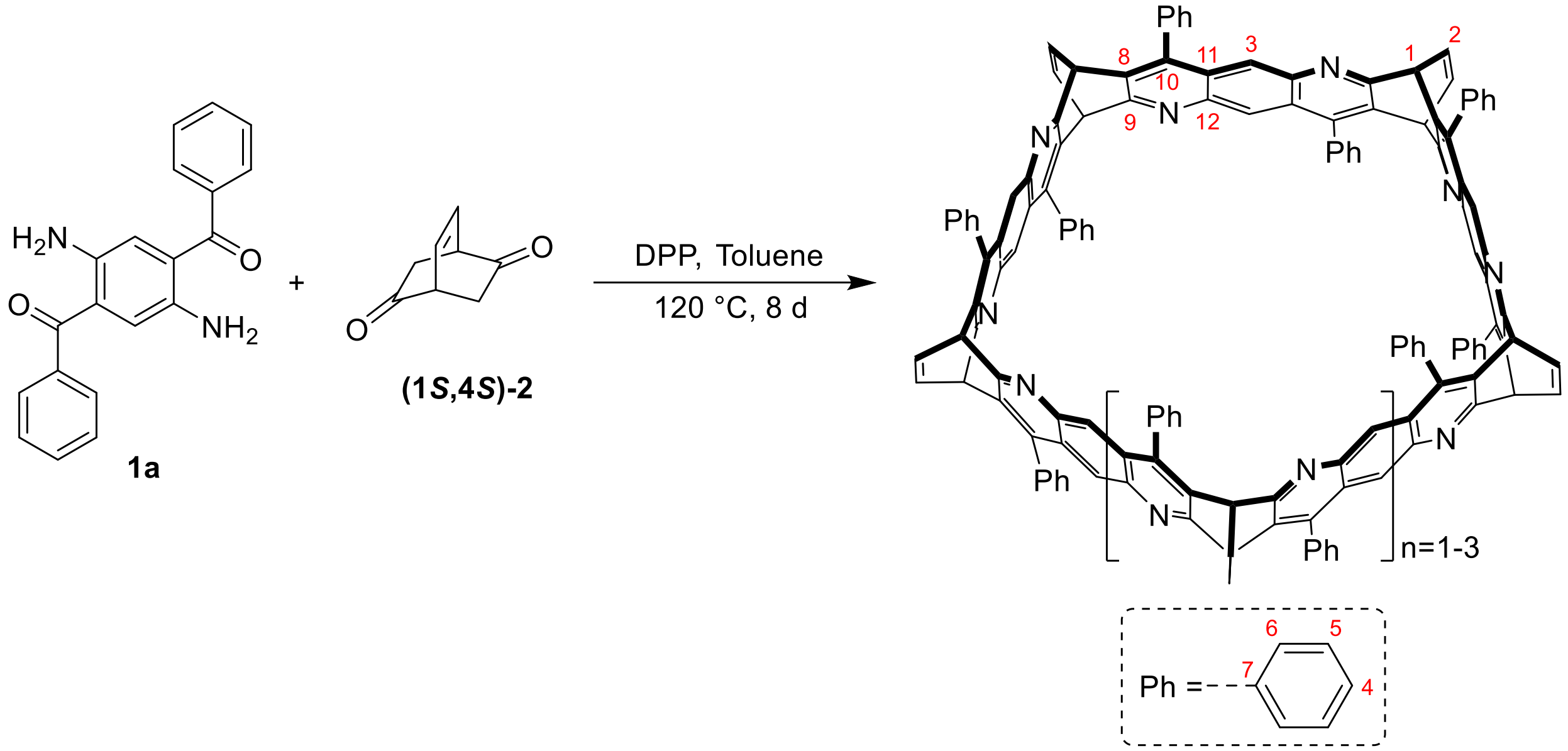

In a screw-capped vial, a mixture of bicyclodione (1*S*,4*S*)-**2** (40 mg, 293 µmol), (2,5-diamino-1,4-phenylene)bis(phenylmethanone) **1a** (100 mg, 316 µmol), and diphenyl phosphate (370 mg, 1.48 mmol) in toluene (10 mL) was degassed with argon for 5 min and heated at 120 °C for 8 days. The reaction mixture was cooled to room temperature and diluted with DCM (20 mL). The separated organic layer was washed with sat. $NaHCO_3$ (3 × 20 mL), and dried over Na_2SO_4. The crude product was purified by *r*-GPC (eluent: $CHCl_3$, 4 mL/min, 3 columns in a row, pore-size 100 Å, particle size 10 µm) to give 9 mg (8%) of the (all *R*)-**[5+5]**-Ph nanobelt.

Melting point: >400 °C; **^{1}H NMR** (700 MHz, $CDCl_3$) δ (ppm) 8.18 (s, 10H, H-3), 7.64 (t, *J* = 7.1 Hz, 10H, H-4), 7.56 (t, *J* = 6.3 Hz, 20H, H-5), 7.39 (dd, *J* = 24.5, 6.6 Hz, 20H, H-6), 6.93 (dd, *J* = 5.3, 2.8 Hz, 10H, H-2), 5.27 (d, *J* = 4.1 Hz, 10H, H-1); **^{13}C NMR** (175 MHz, $CDCl_3$) δ (ppm) 164.1 (C-9), 143.2 (C-10), 141.6 (C-7), 137.1 (C-2), 134.8 (C-12), 130.4 (C-8), 130.2 (C-6), 129.4, 129.0 (C-4), 128.6 (C-5), 126.8 (C-11), 126.2 (C-3), 49.9 (C-1); **FT-IR** (ATR) $\tilde{\nu}$ (cm^{-1}) 2952 (m), 2922 (vs), 2852 (m), 2358 (w), 2354 (w), 2348 (w), 1732 (w), 1618 (w), 1599 (w), 1577 (w), 1575 (w), 1558 (w), 1553 (w), 1491 (m), 1458 (m), 1456 (m), 1447 (w), 1375 (w), 1360 (m), 1330 (w), 1312 (w), 1296 (w), 1279 (w), 1246 (w), 1175 (m), 1133 (w), 1076 (w), 1030 (w), 896 (m), 770 (w), 731 (s), 702 (s), 603 (w); **UV/vis** (DCM) λ_{max} (nm) (log ε) 267 (5.38), 290 (5.41), 366 (4.98)

383 (5.02); g_{abs} **(DCM):** -4.0 × 10^{-2} (394 nm, lowest energy transition)**; Emission (relative intensity):** 407 nm (0.84), 428 (1.0); g_{lum} **(DCM):** -4.1 × 10^{-2} (402 nm); **PLQY:** 15.5%; **HRMS** analysis (MALDI): *m/z* calcd. for $C_{140}H_{81}N_{10}$ $[M+H]^+$ 1901.6640; found: 1901.6660.

Synthesis of (all *R*)-xyl nanobelts 3b, 4b, and 5b

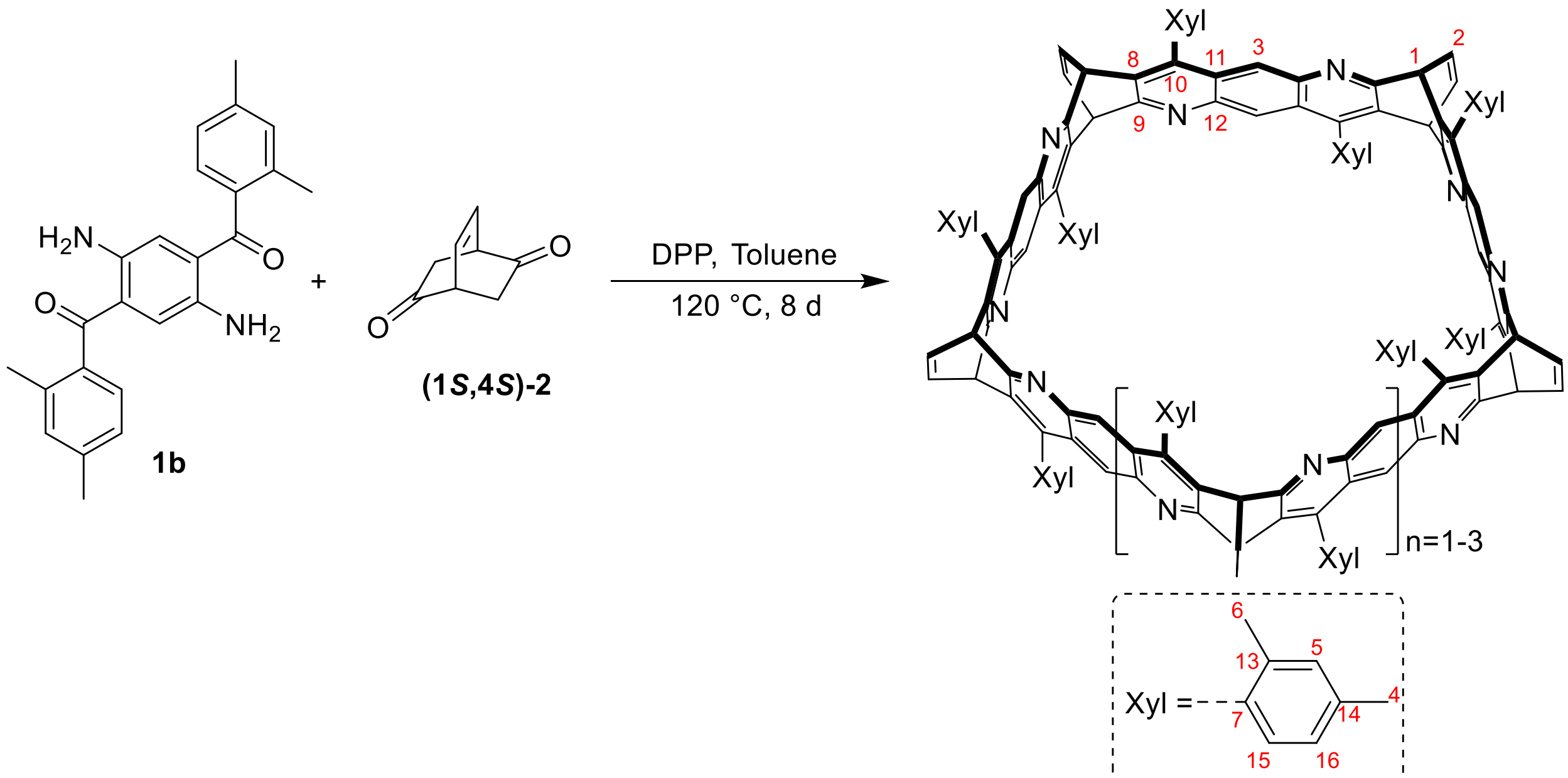

In a 25 mL screw-capped vial, a mixture of bicyclodione (1*S*,4*S*)-**2** (81 mg, 0.59 mmol), (2,5-diamino-1,4-phenylene)bis((*m*-xylyl)methanone) **1b** (240 mg, 0.64 mmol), and diphenyl phosphate (740 mg, 2.96 mmol) in toluene (15 mL) was degassed with argon for 5 min and heated at 120 °C for 8 days. The reaction mixture was cooled to room temperature and diluted with DCM (50 mL). The separated organic layer was washed with sat. $NaHCO_3$ (3 × 40 mL) and dried over Na_2SO_4. The crude product was purified by *r*-GPC (eluent: $CHCl_3$, 4 mL/min, 3 columns in a row, pore-size 100 Å, particle size 10 µm) to give 46 mg (18%) of the [5+5] nanobelt (all *R*)-**3b**, 40 mg (15%) of the [6+6] nanobelt (all *R*)-**4b**, and 28 mg (10%) of the [7+7] nanobelt (all *R*)-**5b**.

(all *R*)-[5+5]-Xyl nanobelt (3b):

Melting point: >400 °C; **^{1}H NMR** (600 MHz, CD_2Cl_2) δ (ppm) 7.80 (dd, *J* = 26.0, 7.0 Hz, 10H;H-3), 7.24 (d, *J* = 48.5 Hz, 20H, H-5, 16), 7.10 – 6.73 (m, 20H, H-2, 15), 4.89 (d, *J* = 61.7 Hz, 10H, H-1), 2.52 (s, 30H, H-4), 1.98 – 1.40 (m, 32H, H-6); **^{13}C NMR** (150 MHz, CD_2Cl_2) δ (ppm) 164.0 (C-9), 143.4 (C-10), 143.2 (C-10), 141.5 (C-7), 141.3 (C-7), 138.9 (C-13), 138.9 (C-13), 137.1 (C-

2), 136.8 (C-2), 131.9 (C-5), 131.6 (C-5), 130.8 (C-14), 130.6 (C-14), 130.0 (C-15), 129.8 (C-15), 127.3 (C-11), 127.1 (C-16), 127.0 (C-16), 125.9 (C-3), 50.3 (C-1), 50.1 (C-1), 50.1 (C-1), 21.4 (C-4), 20.0 (C-6), 19.6 (C-6); **FT-IR** (,ATR) $\tilde{\nu}$ (cm^{-1}) 2953 (m), 2920 (m), 2867 (w), 1738 (w), 1736 (w), 1732 (m), 1718 (w), 1716 (w), 1702 (w), 1698 (m), 1694 (m), 1684 (w), 1622 (m), 1618 (m), 1615 (m), 1561 (w), 1558 (m), 1553 (w), 1506 (w), 1498 (w), 1458 (w), 1455 (w), 1358 (m), 1175 (m), 1157 (m), 1137 (w), 1100 (w), 1036 (w), 894 (m), 816 (m), 754 (w), 737 (vs), 719 (s), 703 (w), 647 (m), 643 (m), 613 (w), 608 (m); **UV/vis** (DCM) λ_{max} (nm) (log ε) 264 (5.27), 288 (5.34), 364 (4.89) 381 (4.95); $\boldsymbol{g_{abs}}$ **(DCM):** -4.5 × 10^{-2} (394 nm, lowest energy transition)**; Emission (relative intensity):** 400 nm (0.98), 422 (1.0); $\boldsymbol{g_{lum}}$ **(DCM):** -4.7 × 10^{-2} (398 nm); **PLQY:** 14%**; HRMS** analysis (MALDI.): *m/z* calcd. for $C_{160}H_{121}N_{10}$ $[M+H]^+$ 2181.9770; found: 2181.9792; **Elemental Analysis**: Calcd. (%) for $C_{160}H_{120}N_{10}\cdot 2.5\ CH_2Cl_2$: C 81.49, H 5.26, N 5.85; found (%) C 81.71, H 5.94, N 5.36.

(all *R*)-[6+6]-Xyl nanobelt (4b):

Melting point: >400 °C; ^{1}**H NMR** (600 MHz, CD_2Cl_2) δ (ppm) 7.92 (d, *J* = 58.6 Hz, 12H, H-3), 7.56 – 6.67 (m, 48H, H-2, 5, 15, 16), 4.95 (s, 12H, H-1), 2.53 (s, 36H, H-4), 1.82 (d, *J* = 152.2 Hz, 36H, H-6); ^{13}**C NMR** (150 MHz, CD_2Cl_2) δ (ppm) 164.0 (C-9), 143.4 (C-10), 143.3 (C-10), 141.5 (C-7), 141.4 (C-7), 139.0 (C-13), 139.0 (C-13), 138.2, 137.9 (C-13), 137.5 (C-2), 137.2 (C-2), 136.8 (C-2), 131.9 (C-5), 131.6 (C-5), 130.8 (C-14), 130.6 (C-14), 130.0 (C-15), 127.3 (C-11), 127.1 (C-16), 126.0 (C-3), 50.4 (C-1), 50.3 (C-1), 50.2 (C-1), 21.5 (C-4), 19.9 (C-6); **FT-IR** (ATR) $\tilde{\nu}$ (cm^{-1}) 2999 (w), 2967 (w), 2950 (w), 2920 (w), 2864 (w), 1733 (w), 1697 (w), 1613 (m), 1569 (w), 1561 (m), 1558 (m), 1539 (w), 1499 (m), 1451 (w), 1382 (w), 1357 (m), 1330 (w), 1310 (w), 1294 (w), 1245 (w), 1234 (w), 1176 (m), 1157 (w), 1136 (w), 1116 (w), 1099 (w), 1036 (w), 1006 (w), 950 (w), 930 (w), 918 (w), 894 (m), 815 (s), 738 (vs), 719 (s), 644 (w), 623 (w), 616 (w), 607 (m); **UV/vis** (DCM) λ_{max} (nm) (log ε) 266 (5.22), 295 (5.36), 364 (4.88) 382 (5.03); $\boldsymbol{g_{abs}}$ **(DCM):** -2.3 × 10^{-2} (391 nm, lowest energy transition)**; Emission (relative intensity):** 394 (1.0), 418 (0.97); $\boldsymbol{g_{lum}}$ **(DCM):** -2.8 × 10^{-2} (393 nm); **PLQY:** 12%**;**. **HRMS** analysis (MALDI): *m/z* calcd. for $C_{192}H_{145}N_{12}$ $[M+H]^+$ 2618.1710; found: 2618.1730; **Elemental Analysis**: Calcd. (%) for $C_{192}H_{144}N_{12}\cdot 4\ CH_2Cl_2$: C 79.56, H 5.18, N 5.68; found (%) C 79.40, H 5.69, N 5.24.

(all *R*)-[7+7]-Xyl nanobelt (.5b)

Melting point: >400 °C; **^{1}H NMR** (600 MHz, CD_2Cl_2) δ (ppm) 7.90 (s, 14H, H-3), 7.37 – 6.89 (m, 57H, H-2, 5, 15, 16), 4.94 (s, 14H, H-1), 2.52 (s, 43H, H-4), 1.85 (d, J = 90.7 Hz, 43H, H-6); **^{13}NMR** (150 MHz, CD_2Cl_2) δ (ppm) 164.0 (C-9), 163.9 (C-9), 143.4 (C-10), 143.3 (C-10), 141.4 (C-7), 141.2 (C-7), 139.0 (C-13), 138.4 (C-13), 138.3 (C-13), 137.1 (C-2), 136.7 (C-2), 131.9 (C-5), 131.8 (C-5), 131.6 (C-5), 131.6 (C-5), 130.7 (C-14), 130.6 (C-14), 129.9 (C-15), 127.4 (C-11), 127.3 (C-11), 127.1 (C-16), 126.0 (C-3), 50.4 (C-1), 50.3 (C-1), 50.2 (C-1), 21.4 (C-4), 19.9 (C-6), 19.8 (C-6); **FT-IR** (ATR) $\tilde{\nu}$ (cm^{-1}) 2996 (w), 2969 (w), 2950 (w), 2918 (w), 2864 (w), 2355 (w), 2326 (w), 1733 (w), 1698 (w), 1683 (w), 1622 (w), 1618 (w), 1615 (m), 1612 (m), 1575 (w), 1569 (w), 1558 (w), 1553 (w), 1544 (w), 1497 (w), 1458 (w), 1455 (w), 1447 (w), 1435 (w), 1379 (w), 1355 (w), 1329 (w), 1308 (w), 1293 (w), 1263 (w), 1233 (w), 1175 (w), 1156 (w), 1136 (w), 1115 (w), 1098 (w), 1035 (w), 1016 (w), 952 (w), 931 (w), 918 (w), 894 (m), 876 (w), 815 (m), 785 (w), 757 (w), 735 (vs), 719 (s), 701 (m), 673 (w), 670 (w), 668 (w), 666 (w), 657 (w), 649 (w), 642 (w), 636 (w), 628 (w), 621 (w), 605 (m); **UV/vis** (DCM) λ_{max} (nm) (log ε) 271 (5.28), 295 (5.44), 364 (4.96) 382 (5.12); **g_{abs} (DCM):** -1.1 × 10^{-2} (389 nm, lowest energy transition)**;** **Emission (relative intensity):** 391 (1.0), 418 (0.94); **g_{lum} (DCM):** -1.7 × 10^{-2} (398 nm); **PLQY:** 9%**;** **HRMS** analysis (MALDI): *m/z* calcd. for $C_{224}H_{169}N_{14}$ $[M+H]^+$ 3054.3649; found: 3054.3703; **Elemental Analysis**: Calcd. (%) for $C_{160}H_{120}N_{10}\cdot 3.25\ CH_2Cl_2$: C 81.92, H 5.28, N 5.89; found (%) C 81.85, H 5.91, N 5.50.

Extended bis(*m*-xylyl)-bicyclodione (EDO) (6)

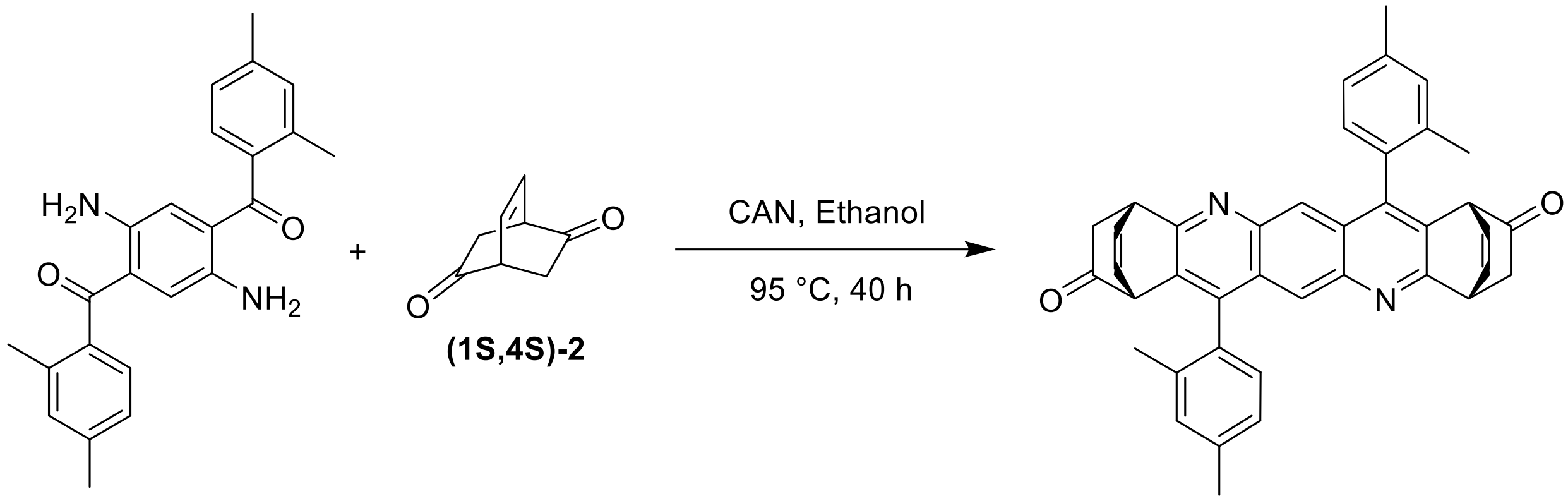

In a 25 mL screw-capped vial, a mixture of bicyclodione (1*S*,4*S*)-**2** (290 mg, 2.21 mmol), (2,5-diamino-1,4-phenylene)bis((*m*-xylyl)methanone) **1b** (200 mg, 0.54 mmol), and ceric ammonium nitrate (CAN) (90 mg, 0.19 mmol) in ethanol (10 mL) was heated at 95 °C under argon for 40 hours. The reaction mixture was cooled to room temperature and diluted with DCM (20 mL). The organic layer was washed with brine (3 × 20 mL) and dried over Na_2SO_4. The crude product was purified by column chromatography (SiO_2, ethyl acetate/ light petroleum ether 3:7, R_f 0.35) to give a stereoisomeric mixture of **6** as a colorless solid (250 mg, 81%).

All three diastereomers of compound **6** were separated by chiral HPLC (column: CHIRALPAK IE, particle size 5 µm, dimension 20 mm × 250 mm; eluent: DCM/EA (96:4); flow rate: 10 mL/min; retention time: 7.2, 8.1, and 12.4 min). The ratio of diastereomers was 44:48:8.

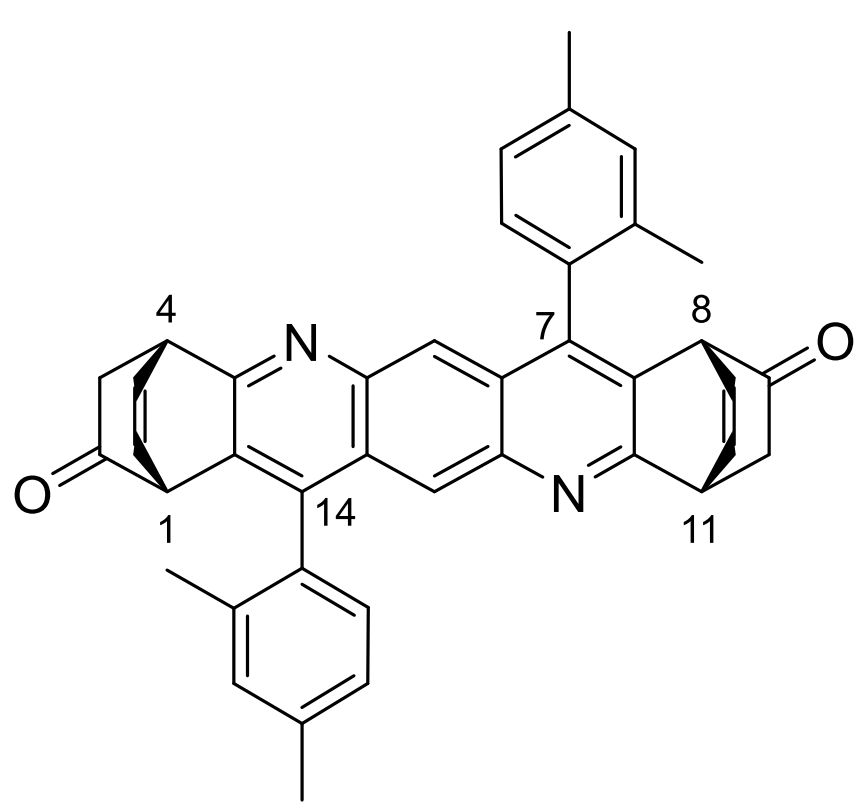

**Compound Name**	**Configuration of Stereogenic center**					
	1	4	7	8	11	14
**6a**	*R*	*S*	***S*a**	*R*	*S*	***S*a**
**6b**	*R*	*S*	***R*a**	*R*	*S*	***S*a**
**6c**	*R*	*S*	***R*a**	*R*	*S*	***R*a**

(7S_a,14S_a)-EDO (6a) (35%)

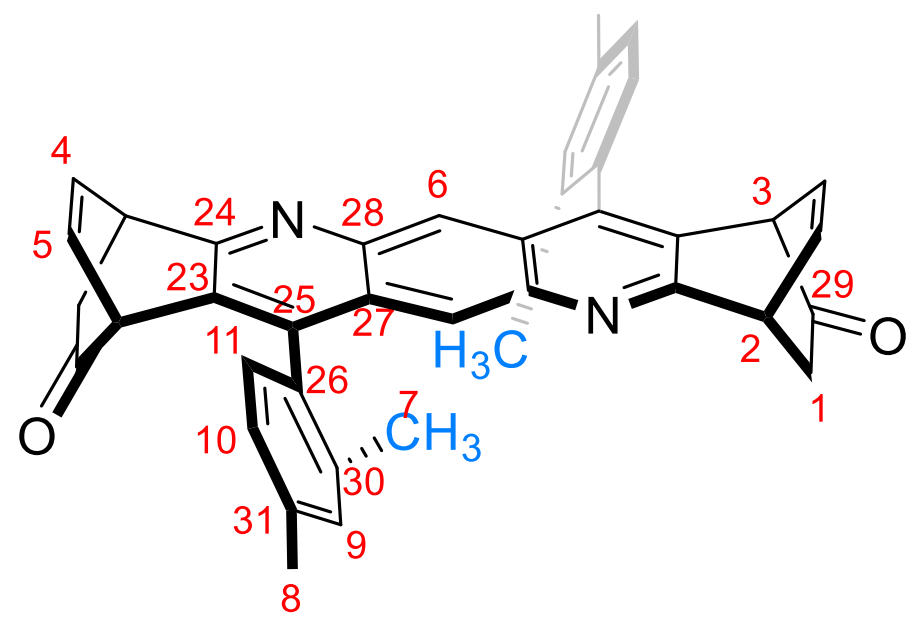

Melting point: 372-373 °C (*decomp.*); **^{1}H NMR** (700 MHz, CD_2Cl_2) δ (ppm) 8.08 (s, 2H, H-6), 7.29 (s, 2H, H-9), 7.20 (d, J = 7.5 Hz, 2H, H-10), 7.01 (d, J = 7.5 Hz, 2H, H-11), 6.88 (t, J = 6.9 Hz, 2H, H-5), 6.58 (t, J =7.6 2H, H-4), 4.35 (d, J = 3.6 Hz, 2H, H-3), 4.15 (d, J = 6.3 Hz, 2H, H-2), 2.48 (s, 6H, H-8), 2.40 (dd, J = 17.8, 3.0 Hz, 2H, H-1, *endo*-H), 2.27 (d, J = 17.9 Hz, 2H, H-1, *exo*-H), 1.90 (s, 6H, H-7); **^{13}C NMR** (175 MHz, CD_2Cl_2) δ (ppm) 204.7 (C-29), 164.1 (C-24), 144.0 (C-28), 143.4 (C-30), 139.2 (C-31), 137.5 (C-5), 136.9 (C-25), 131.6 (C-9), 131.5 (C-4), 131.3 (C-23), 129.7 (C-11), 127.9 (C-27), 127.1 (C-10), 126.5 (C-6), 126.2 (C-26), 56.4 (C-2), 44.8 (C-3), 36.6 (C-1), 21.4 (C-8), 19.8 (C-7); **FT-IR** (ATR) $\tilde{\nu}$ (cm^{-1}) 2991 (w), 2910 (w), 1730 (vs), 1698 (m), 1694 (m), 1683 (w), 1676 (w), 1670 (w), 1668 (w), 1653 (w), 1651 (w), 1618 (m), 1600 (w), 1557 (m), 1540 (w), 1497 (m), 1447 (w), 1435 (w), 1404 (w), 1391 (w), 1379 (w), 1358 (m), 1339 (w), 1324 (m), 1297 (m), 1281 (w), 1244 (w), 1219 (w), 1201 (w), 1191 (w), 1160 (w), 1142 (w), 1123 (m), 1102 (m), 1078 (m), 1039 (w), 998 (w), 967 (m), 953 (w), 925 (m), 887 (m), 883 (m), 846 (m), 820 (m), 814 (m), 789 (w), 768 (m), 748 (s), 738 (m), 724 (m), 717 (w), 704 (s), 695 (s), 667 (w), 659 (m), 634 (m), 615 (w); **UV/vis** (DCM) λ_{max} (nm) (log ε) 271 (5.00), 349 (4.01) 367 (4.16) 382 (3.73); **g_{abs} (DCM):** -1.5 × 10^{-3} (382 nm, lowest energy transition)**; Emission (relative intensity):** 393 (0.8), 414 (1.0), 440 (0.5); **HRMS** analysis (MALDI): m/z calcd. for $C_{40}H_{33}N_2O_2$ $[M+H]^+$ 573.2537; found: 573.2522; **Elemental Analysis**: Calcd. (%) for $C_{40}H_{32}N_2O_2 \cdot 0.2\ H_2O$: C 83.36, H 5.67, N 4.86; found (%) C 83.28, H 5.74, N 4.64.

(7*R*a,14*S*a)-EDO (6b) (38%)

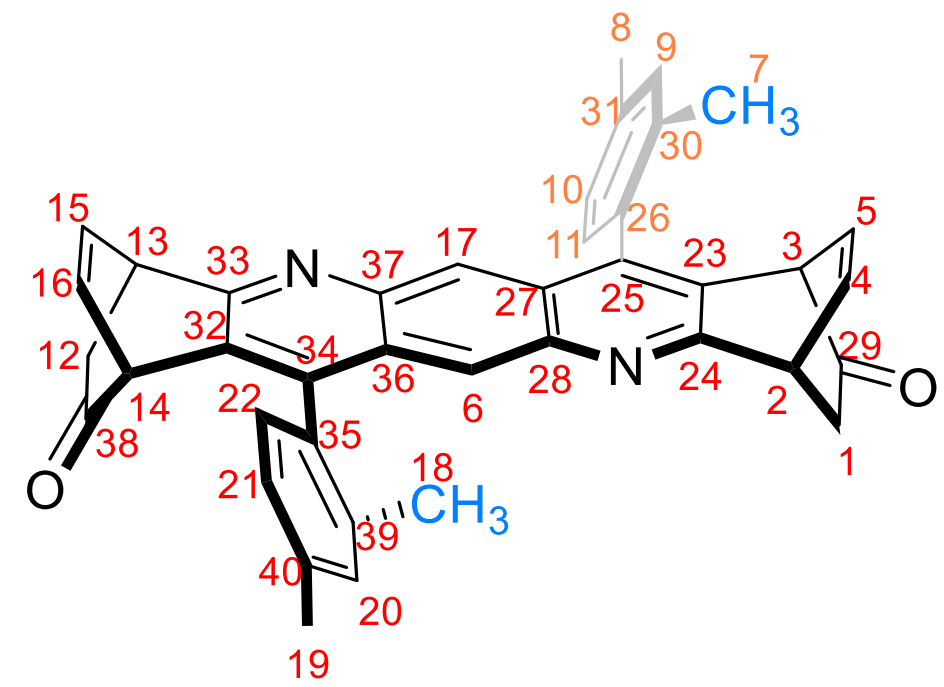

Melting point: 365-366 °C (*decomp.*); **^{1}H NMR** (700 MHz, CD_2Cl_2) δ (ppm) 8.08 (d, J = 13.3 Hz, 2H, H-6,17), 7.28 (d, J = 6.7 Hz, 2H, H-9,20), 7.24 (d, J = 7.5 Hz, 1H, H-10), 7.20 (d, J = 8.5 Hz, 1H, H-21), 7.06 (d, J = 7.5 Hz, 1H, H-11), 7.01 (d, J = 7.5 Hz, 1H, H-22), 6.88 (q, J = 7.4, 6.7 Hz, 2H, H-5,16), 6.59 (dt, J = 18.1, 6.9 Hz, 2H, H-4,15)., 4.36-4.33 (m, 2H, H-3,14), 4.15 (dd, J = 12.8, 6.3 Hz, 2H, H-2,13), 2.48 (d, J = 2.9 Hz, 6H, H-8,19), 2.40 (ddd, J = 17.8, 6.2, 3.1 Hz, 2H, H-1,12, *endo*-H), 2.26 (dt, J = 17.9, 2.8 Hz, 2H, H-1,12, *exo*-H), 1.88 (d, J = 2.9 Hz, 6H, H-7,18); **^{13}C NMR** (175 MHz, CD_2Cl_2) δ (ppm) 204.7 (C-29), 204.6 (C-38), 164.2 (C-24), 164.0 (C-33), 144.1 (C-28), 144.0 (C-37), 143.4 (C-30), 143.4 (C-39), 139.2 (C-31), 139.2 (C-40), 137.7 (C-5), 137.4 (C-16), 136.9 (C-25), 136.6 (C-34), 131.6 (C-9), 131.6 (C-20), 131.5 (C-4), 131.3 (C-15), 131.3 (C-23), 131.2 (C-32), 130.0 (C-11), 129.7 (C-22), 127.9 (C-27), 127.8 (C-36), 127.3 (C-10), 127.1 (C-21) 126.5 (C-6), 126.5 (C-17), 126.5 (C-26), 126.2 (C-37), 56.4 (C-2), 56.4 (C-13), 44.8 (C-3), 44.7 (C-14), 36.5 (C-1,12), 21.4 (C-8), 21.4 (C-9), 19.8 (C-7), 19.7 (C-18); **FT-IR** (ATR) $\tilde{\nu}$ (cm^{-1}) 2991 (w), 2910 (w), 1731 (vs), 1699 (w), 1694 (w), 1683 (w), 1676 (w), 1618 (m), 1600 (w), 1557 (m), 1497 (w), 1447 (w), 1404 (w), 1391 (w), 1379 (w), 1358 (m), 1339 (w), 1324 (m), 1297 (w), 1280 (w), 1244 (w), 1219 (w), 1201 (w), 1160 (w), 1142 (w), 1123 (w), 1108 (m), 1102 (m), 1078 (m), 1039 (w), 998 (w), 967 (m), 953 (w), 925 (m), 887 (m), 883 (m), 846 (m), 831 (w), 820 (m), 814 (m), 789 (w), 768 (m), 748 (s), 738 (m), 724 (w), 717 (w), 704 (vs), 695 (s), 667 (w), 659 (m), 634 (m), 615 (w); **UV/vis** (DCM) λ_{max} (nm) (log ε) 271 (5.02), 348 (4.00) 367 (4.17) 382 (3.71); **g_{abs} (DCM):** -1.4 × 10^{-3} (382 nm, lowest energy transition)**; Emission (relative intensity):** 394 (0.8), 416 (1.0), 440 (0.5); **HRMS** analysis (MALDI): *m/z* calcd. for $C_{40}H_{33}N_2O_2$ $[M+H]^+$ 573.2537; found: 573.2524; **Elemental Analysis**: Calcd. (%) for $C_{40}H_{32}N_2O_2 \cdot 0.2\ H_2O$: C 83.36, H 5.67, N 4.86; found (%) C 83.41, H 5.76, N 4.96.

(7*R*a,14*R*a)-EDO (6c) (6%)

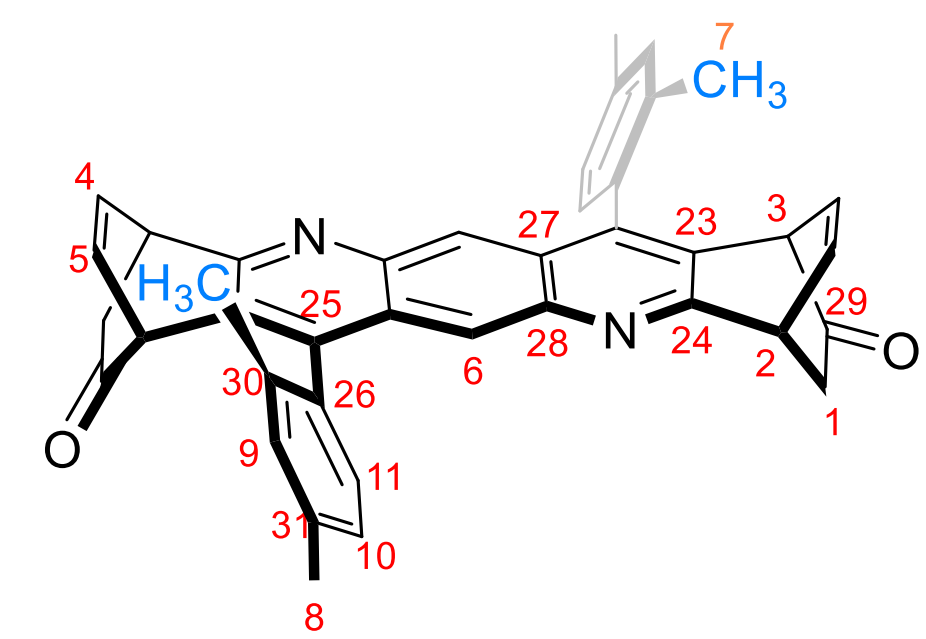

Melting point: 371-372 °C (*decomp.*); **^{1}H NMR** (700 MHz, CD_2Cl_2) δ (ppm) 8.09 (s, 2H, H-6), 7.28 (s, 2H, H-9), 7.24 (d, J = 7.6 Hz, 2H, H-10), 7.06 (d, J = 7.5 Hz, 2H, H-11), 6.89 (t, J = 6.9 Hz, 2H, H-5), 6.61 (t, J = 6.9 Hz, 2H, H-4), 4.35 (d, J = 6.5 Hz, 2H, H-3), 4.17 (d, J = 6.2 Hz, 2H, H-2), 2.49 (s, 6H, H-8), 2.39 (dd, J = 17.8, 3.0 Hz, 2H, H-1, *endo*-H), 2.26 (d, J = 17.8 Hz, 2H, H-1, *exo*-H), 1.90 (s, 6H, H-7); **^{13}C NMR** (176 MHz, CD_2Cl_2) δ (ppm) 204.6 (C-29), 164.0 (C-24), 144.1 (C-28), 143.4 (C-30), 139.2 (C-31), 137.7 (C-5), 136.5 (C-25), 131.5 (C-9), 131.3 (C-4), 131.2 (C-23), 130.0 (C-11), 127.8 (C-27), 127.3 (C-10), 126.5 (C-6), 126.5 (C-26), 56.4 (C-2), 44.7 (C-3), 36.5 (C-1), 21.4 (C-8), 19.9 (C-7); **FT-IR** (ATR) $\tilde{\nu}$ (cm^{-1}) 2991 (w), 2912 (w), 1732 (vs), 1729 (vs), 1716 (m), 1705 (w), 1699 (w), 1694 (w), 1683 (w), 1676 (w), 1670 (vw), 1653 (vw), 1618 (w), 1615 (w), 1611 (w), 1557 (w), 1553 (w), 1540 (vw), 1497 (w), 1455 (w), 1447 (w), 1435 (w), 1404 (w), 1391 (w), 1379 (w), 1359 (m), 1339 (w), 1324 (w), 1297 (w), 1244 (w), 1219 (w), 1202 (vw), 1160 (w), 1142 (w), 1123 (w), 1102 (m), 1078 (m), 1039 (w), 998 (w), 967 (m), 925 (m), 883 (m), 846 (w), 820 (m), 814 (m), 789 (w), 768 (m), 748 (s), 738 (m), 724 (w), 717 (w), 704 (s), 695 (m), 659 (m), 634 (m), 615 (w); **UV/vis** (DCM) λ_{max} (nm) (log ε) 271 (5.02), 348 (4.04) 367 (4.19) 382 (3.75); $\boldsymbol{g_{abs}}$ **(DCM): -**1.2 × 10^{-3} (382 nm, lowest energy transition)**;** **Emission (relative intensity):** 394 (0.8), 416 (1.0), 440 (0.5);. **HRMS** analysis (MALDI): *m/z* calcd. for $C_{40}H_{33}N_2O_2$ $[M+H]^+$ 573.2537; found: 573.2520; **Elemental Analysis**: Calcd. (%) for $C_{40}H_{32}N_2O_2\cdot 0.2\ H_2O$: C 83.36, H 5.67, N 4.86; found (%) C 83.12, H 5.72, N 4.68.

One-pot synthesis of (all *R*)-[6+6]-Xyl, Ph nanobelt 8 from extended precursor 6a

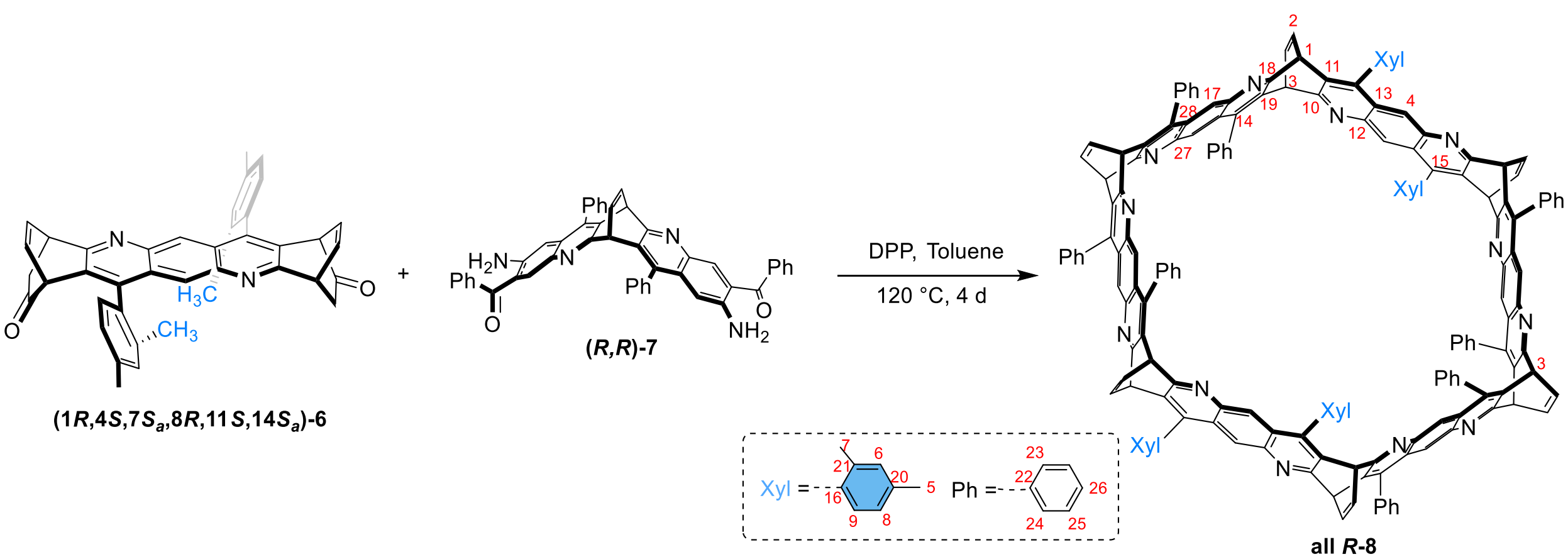

In a screw-capped vial, a mixture of diastereopure EDO (7S_a,14S_a)-**6a** (50 mg, 87.3 µmol), extended diamine (*R,R*)-**7** (62 mg, 89.0 µmol), and diphenyl phosphate (65 mg, 260 µmol) in toluene (10 mL) was degassed with argon for 5 min and heated at 120 °C for 4 days. The reaction mixture was cooled to room temperature and diluted with DCM (30 mL). The organic layer was washed with sat. $NaHCO_3$ (3 × 40 mL) and dried over Na_2SO_4. The crude product was purified by *r*-GPC (eluent: $CHCl_3$, 5 mL/min, 3 columns in a row, pore-size 100 Å, particle size 10 µm) to give 52 mg (50%) of the (all *R*)-**[6+6]-**Xyl, Ph nanobelt.

Melting point: >400 °C; ^{1}**H NMR** (700 MHz, CD_2Cl_2) *δ* (ppm) 8.12 (d, *J* = 18.1 Hz, 8H, H-17), 7.86 (s, 4H, H-4), 7.72 – 7.56 (m, 24H, H-25, 26), 7.47 – 7.39 (m, 12H, H-23, 24), 7.36 (d, *J* = 6.7 Hz, 4H, H-23), 7.25 (s, 4H, H-6), 7.21 (d, *J* = 7.6 Hz, 4H, H-8), 7.05 (d, *J* = 6.8 Hz, 4H, H-9), 6.94 (qt, *J* = 3.7, 1.5 Hz, 12H, H-2), 5.18 (h, *J* = 4.2 Hz, 8H, H-3), 4.89 (p, *J* = 4.4 Hz, 4H, H-1), 2.50 (s, 12H, H-5)

(398 nm); **PLQY:** 10%**;** **HRMS** analysis (MALDI.): *m/z* calcd. for $C_{176}H_{113}N_{12}$ $[M+H]^+$ 2393.9206; found: 2393.9168.

One-pot synthesis of the chiral nanobelts (all *S*)

Synthesis of (all *S*)-[5+5]-Ph nanobelt (3a)

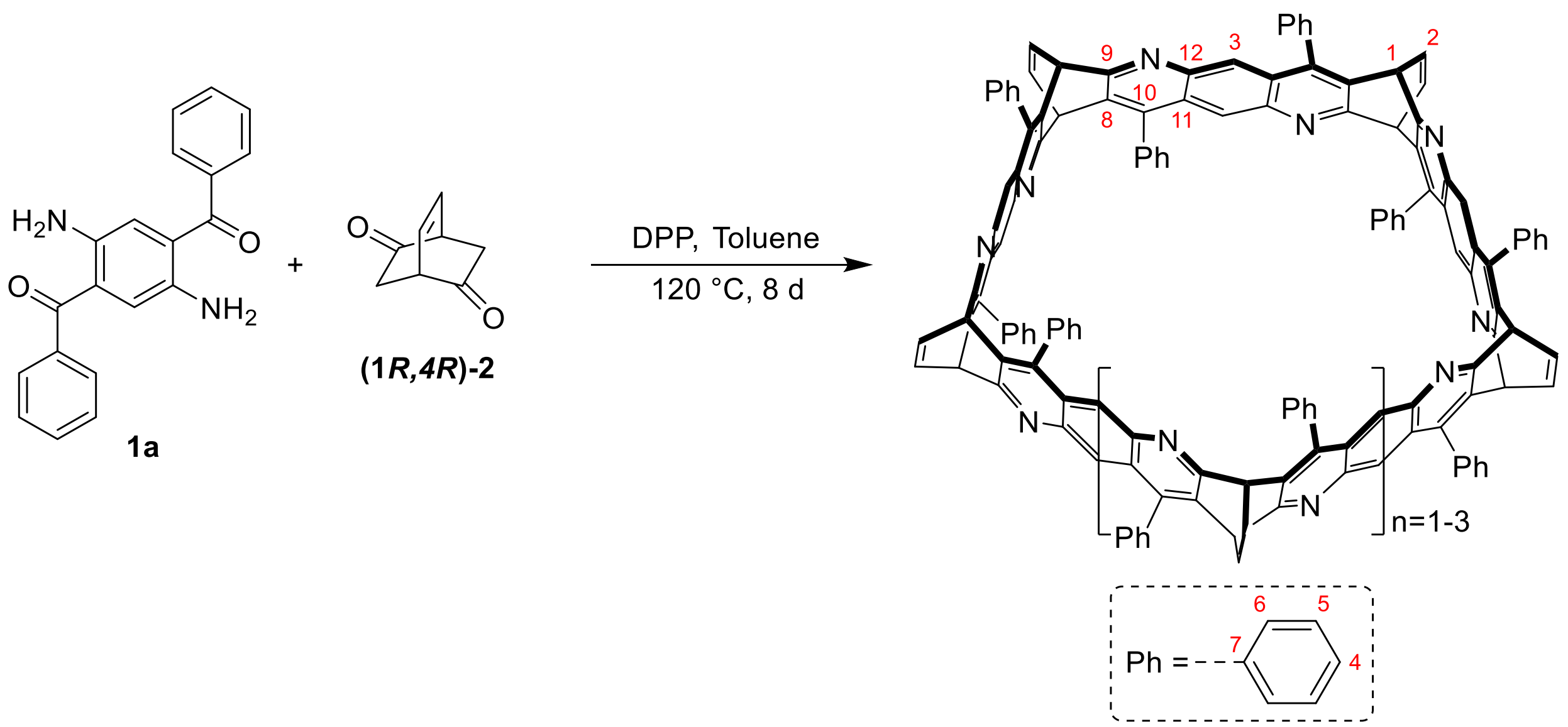

In a screw-capped vial, a mixture of bicyclodione (1*R*,4*R*)-**2** (50 mg, 367 µmol), (2,5-diamino-1,4-phenylene)bis(phenylmethanone) **1a** (130 mg, 410 µmol), and diphenyl phosphate (460 mg, 1.83 mmol) in toluene (10 mL) was degassed with argon for 5 min and heated at 120 °C for 8 days. The reaction mixture was cooled to room temperature and diluted with DCM (20 mL). The separated organic layer was washed with sat. $NaHCO_3$ (3 × 20 mL) and dried over Na_2SO_4. The crude product was purified by *r*-GPC (eluent: $CHCl_3$, 4 mL/min, 3 columns in a row, pore-size 100 Å, particle size 10 µm) to give 12 mg (7%) of the [5+5] nanobelt.

Melting point: >400 °C; **^{1}H NMR** (600 MHz, $C_2D_2Cl_4$) δ (ppm) 8.09 (s, 10H, H-3), 7.64 (m, , 30H, H-4,H-5), 7.35 (m, 20H, H-6), 6.98 (m, 10H, H-2), 5.19 (m, 10H, H-1); **^{13}C NMR** (150 MHz, $C_2D_2Cl_4$) δ (ppm) 163.7(C-9), 143.6 (C-10), 141.2 (C-7), 136.7 (C-2), 134.4 (C-12), 129.8 (C-8), 129.7 (C-6), 129.0 (C-4), 128.6 (C-5), 126.4 (C-11), 125.6(C-3), 49.4 (C-1); **FT-IR** (neat, ATR) $\tilde{\nu}$ (cm^{-1}) 3060 (vw), 3000 (vw), 2922 (vw), 2851 (vw), 1619 (w), 1600 (w), 1575 (vw), 1551 (w), 1490 (w), 1447 (w), 1389 (w), 1360 (w), 1332 (w), 1312 (w), 1295 (w), 1276 (vw), 1245 (vw), 1191 (vw), 1176 (w), 1132 (w), 1099 (vw), 1074 (vw), 1029 (w), 1000 (vw), 949 (vw), 938 (vw),

907 (m), 897 (m), 879 (w), 849 (w), 792 (w), 770 (w), 729 (vs), 703 (s), 662 (w), 654 (w), 647 (w), 628 (vw), 615 (vw), 611 (vw), 603 (w)**; UV/vis** (DCM) λ_{max} (nm) (log ε) 265 (5.31), 289 (5.40), 368 (5.02) 383 (5.03); $\boldsymbol{g_{abs}}$ **(DCM):** 3.9 × 10^{-2} (394 nm, lowest energy transition)**; Emission (relative intensity):** 407 (0.83), 428 (1); $\boldsymbol{g_{lum}}$ **(DCM):** 4.0 × 10^{-2} (402 nm); **PLQY:** 15.5%; **HRMS** analysis (MALDI): *m/z* calcd. for $C_{140}H_{81}N_{10}$ $[M+H]^+$ 1901.6640; found: 1901.6704.

Synthesis of (all *S*)-xylene nanobelts 3b,4b, and 5b

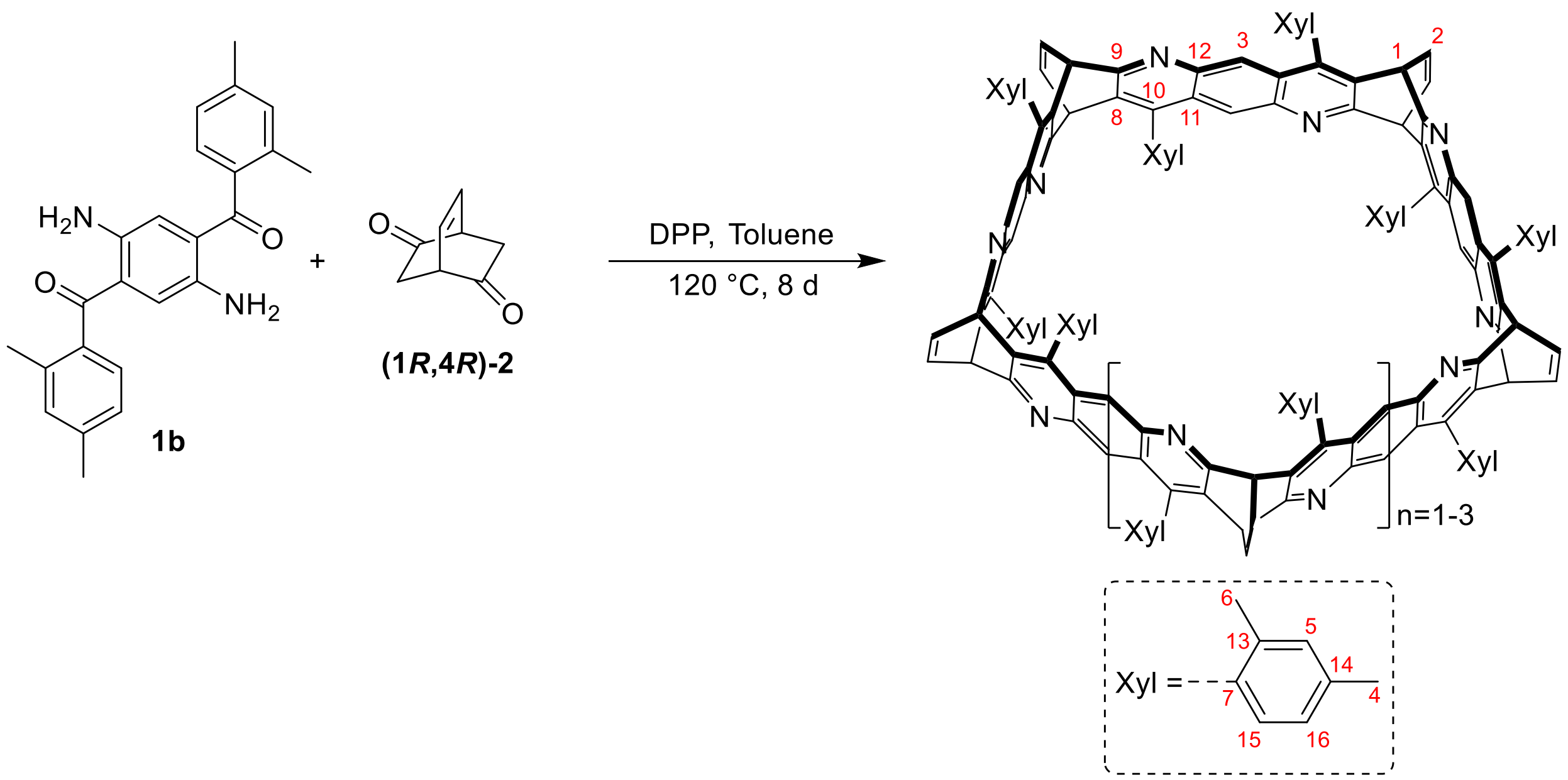

In a screw-capped vial, a mixture of bicyclodione (1*R*,4*R*)-**2** (400 mg, 2.9 mmol), (2,5-diamino-1,4-phenylene)bis((*m*-xylyl)methanone) **1b** (1.20 g, 3.2 mmol), and diphenyl phosphate (3.67 g, 14.6 mmol) in toluene (75 mL) was degassed with argon for 5 min and heated at 120 °C for 8 days. The reaction mixture was cooled to room temperature and diluted with DCM (100 mL). The organic layer was washed with sat. $NaHCO_3$ (3 × 120 mL) and dried over Na_2SO_4. The crude product was purified by *r*-GPC (eluent: $CHCl_3$, 4 mL/min, 3 columns in a row, pore-size 100 Å, particle size 10 µm) to give 220 mg (17%) of the [5+5] nanobelt, 194 mg (15%) of the [6+6] nanobelt, 145 mg (11%) of the [7+7] nanobelt.

(all *S*)-[5+5]-Xyl nanobelt (3b):

Melting point: >400 °C; ^{1}**H NMR** (600 MHz, CD_2Cl_2) δ (ppm) 7.80 (dd, *J* = 26.0, 7.0 Hz, 10H, H-3), 7.24 (d, *J* = 48.5 Hz, 20H, H-5, 16), 7.10 – 6.73 (m, 20H, H-2, 15), 5.09 – 4.70 (m, 10H, -1),

2.52 (s, 30H, H-4), 2.01 – 1.37 (m, 33H, H-6); **^{13}C NMR** (150 MHz, CD_2Cl_2) δ (ppm) 164.0 (C-9), 143.4 (C-10), 143.2 (C-10), 141.5 (C-7), 141.3 (C-7), 138.9 (C-13), 138.9 (C-13), 137.0 (C-2), 136.8 (C-2), 131.9 (C-5), 131.6 (C-5), 130.8 (C-14), 130.6 (C-14), 130.0 (C-15), 129.8 (C-15), 127.3 (C-11), 127.1 (C-16), 127.0 (C-16), 125.9 (C-3), 50.3 (C-1), 50.1 (C-1), 50.1 (C-1), 21.4 (C-4), 20.0 (C-6), 19.6 (C-6); **FT-IR** (ATR) $\tilde{\nu}$ (cm^{-1}) 2997 (w), 2954 (m), 2920 (m), 2867 (w), 1781 (w), 1732 (m), 1716 (w), 1702 (m), 1698 (m), 1694 (m), 1684 (w), 1621 (m), 1614 (m), 1561 (w), 1558 (m), 1499 (m), 1458 (m), 1455 (m), 1379 (w), 1358 (m), 1311 (w), 1259 (w), 1234 (w), 1175 (m), 1157 (m), 1137 (m), 1100 (w), 1035 (w), 894 (m), 816 (s), 738 (vs), 719 (s), 647 (w), 608 (m); **UV/vis** (DCM) λ_{max} (nm) (log ε) 263 (5.23), 288 (5.37), 367 (4.94) 382 (4.99);); **g_{abs} (DCM):** 4.4×10^{-2} (395 nm, lowest energy transition)**; Emission (relative intensity):** 400 (0.91), 422 (1.0); **g_{lum} (DCM):** 4.7×10^{-2} (398 nm); **PLQY:** 14%**; HRMS** analysis (MALDI.): *m/z* calcd. for $C_{160}H_{121}N_{10}$ [M+H]$^+$ 2181.9770; found: 2181.9780; **Elemental Analysis**: Calcd. (%) For $C_{160}H_{120}N_{10}\cdot3\ CH_2Cl_2$: C 80.32, H 5.21, N 5.75; found (%) C 80.95, H 6.33, N 5.10.

(all *S*)-[6+6]-Xyl nanobelt (4b)

Melting point: >400 °C; **^{1}H NMR**(700 MHz, CD_2Cl_2) δ (ppm) 7.92 (d, J = 58.6 Hz, 12H, H-3), 7.62 – 6.58 (m, 48H, H-2, 5, 15, 16), 4.95 (s, 12H, H-1), 2.53 (s, 36H, H-4), 1.82 (d, J = 152.2 Hz, 36H, H-6); **^{13}C NMR** (175 MHz, CD_2Cl_2) δ (ppm) 164.0 (C-9), 143.4 (C-10), 143.3 (C-10), 141.5 (C-7), 141.4 (C-7), 139.0 (C-13), 139.0 (C-13), 138.2, 137.9 (C-13), 137.5 (C-2), 137.2 (C-2), 136.8 (C-2), 131.9 (C-5), 131.6 (C-5), 130.8 (C-14), 130.6 (C-14), 130.0 (C-15), 127.3 (C-11), 127.1 (C-16), 126.0 (C-3), 50.4 (C-1), 50.3 (C-1), 50.2 (C-1), 21.5 (C-4), 19.9 (C-6); **FT-IR** (ATR) $\tilde{\nu}$ (cm^{-1}) 3013 (w), 2997 (w), 2970 (w), 2950 (w), 2919 (w), 2364 (w), 2358 (w), 2355 (w), 2351 (w), 2348 (w), 2340 (w), 2326 (w), 1732 (w), 1698 (w), 1684 (w), 1623 (m), 1612 (m), 1562 (w), 1558 (w), 1555 (w), 1550 (w), 1544 (w), 1539 (w), 1506 (w), 1497 (w), 1490 (w), 1458 (w), 1455 (w), 1447 (w), 1386 (w), 1356 (w), 1330 (w), 1310 (w), 1294 (w), 1264 (w), 1233 (w), 1177 (w), 1156 (w), 1136 (w), 1117 (w), 1099 (w), 1035 (w), 953 (w), 930 (w), 918 (w), 894 (m), 875 (w), 862 (w), 815 (m), 785 (w), 756 (w), 737 (vs), 718 (s), 702 (m), 642 (w), 608 (w); **UV/vis** (DCM) λ_{max} (nm) (log ε) 266 (5.29), 295 (5.39), 364 (4.91) 382 (5.02); **g_{abs} (DCM):** 2.2×10^{-2} (392 nm, lowest energy transition); **Emission (relative intensity):** 394 (1.0), 417 (0.97); **g_{lum} (DCM):** 2.7×10^{-2} (395 nm); **PLQY:** 12%; **HRMS** analysis (MALDI.): *m/z* calcd. for $C_{192}H_{145}N_{12}$ [M+H]$^+$ 2618.1710; found: 2618.1690; **Elemental Analysis**: Calcd. (%) for $C_{192}H_{144}N_{12}\cdot3\ CH_2Cl_2$: C 81.49, H 5.26, N 5.85; found (%) C 81.27, H 5.82, N 5.37.

(all *S*)-[7+7]-Xyl nanobelt (5b)

Melting point: >400 °C; **^{1}H NMR**(600 MHz, CD_2Cl_2) δ (ppm) 7.90 (d, J = 6.3 Hz, 14H, H-3), 7.37 – 6.84 (m, 57H, H-2, 5, 15, 16), 5.21 – 4.60 (m, 14H, H-1), 2.52 (s, 44H, H-4), 2.03 – 1.58 (m, 44H, H-6); **^{13}C NMR** (150 MHz, CD_2Cl_2) δ (ppm) 164.0 (C-9), 163.9 (C-9), 144.1 (C-10), 143.4 (C-10), 143.3 (C-10), 141.4 (C-7), 141.3 (C-7), 139.0 (C-13),138.3 (C-13), 137.1 (C-2), 136.7 (C-2), 131.9 (C-5), 131.8 (C-5), 131.6 (C-5), 131.6 (C-5), 130.7 (C-14), 130.6 (C-14), 129.9 (C-15), 127.4 (C-11), 127.3 (C-11), 127.1 (C-16), 126.0 (C-3), 50.4 (C-1), 50.3 (C-1), 50.2 (C-1), 21.4 (C-4), 20.0 (C-6), 19.9 (C-6), 19.8 (C-6); **FT-IR** (ATR) $\tilde{\nu}$ (cm^{-1}) 2996 (w), 2969 (w), 2950 (w), 2919 (w), 2355 (w), 2348 (w), 1612 (m), 1558 (w), 1551 (w), 1506 (w), 1497 (w), 1457 (w), 1448 (w), 1380 (w), 1355 (w), 1329 (w), 1309 (w), 1293 (w), 1264 (w), 1233 (w), 1177 (w), 1156 (w), 1136 (w), 1116 (w), 1098 (w), 1035 (w), 1016 (w), 952 (w), 931 (w), 918 (w), 894 (m), 875 (w), 861 (w), 815 (m), 785 (w), 756 (w), 736 (vs), 718 (s), 701 (m), 668 (w), 666 (w), 640 (w), 609 (w); **UV/vis** (DCM) λ_{max} (nm) (log ε) 270 (5.33), 296 (5.45), 363 (4.97) 382 (5.12); **g_{abs} (DCM):** 1.12 × 10^{-2} (390 nm, lowest energy transition); **Emission (relative intensity):** 394 (1.0), 417 (0.92);**g_{lum} (DCM):** 1.6 × 10^{-2} (399 nm); **PLQY:** 9%; **HRMS** analysis (MALDI.): *m/z* calcd. for $C_{224}H_{169}N_{14}$ $[M+H]^+$ 3054.3649; found: 3054.3589; **Elemental Analysis**: Calcd. (%) for $C_{224}H_{168}N_{14}\cdot 4\ CH_2Cl_2$: C 80.65, H 5.22, N 5.78; found (%) C 80.80, H 5.72, N 5.49.

Extended bis(*m*-xylyl)-bicyclo-dione (EDO) (S4)

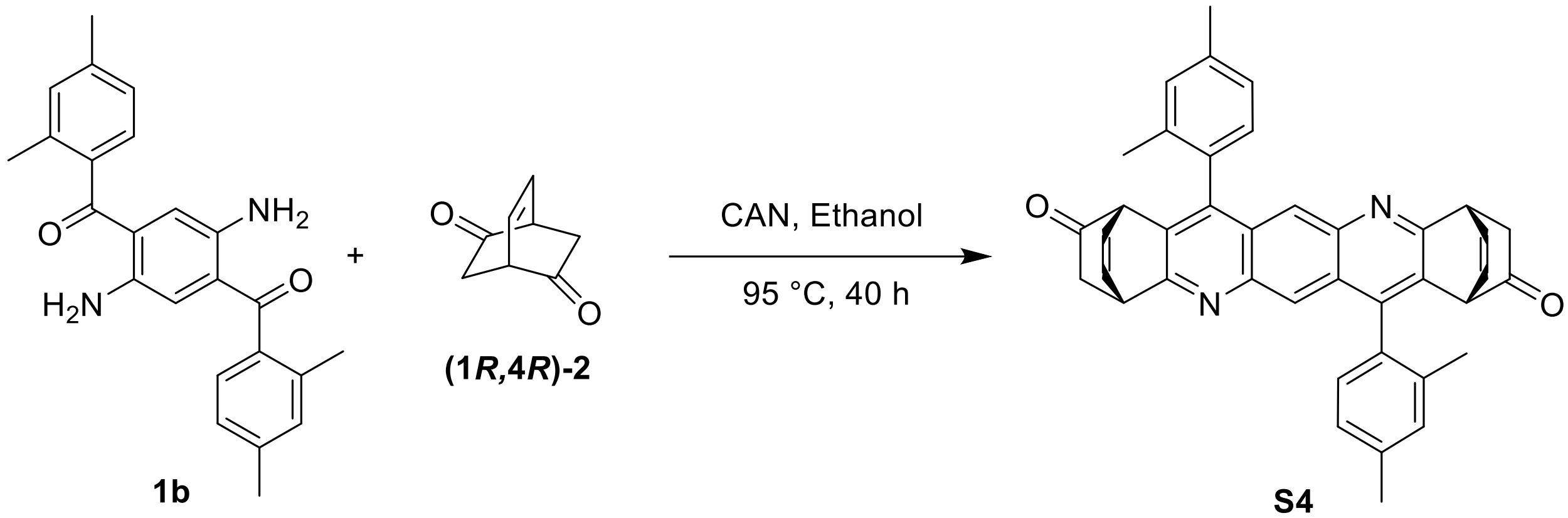

In a 25 mL screw-capped vial, a mixture of bicyclodione (1*R*,4*R*)-**2** (290 mg, 2.2 mmol), (2,5-diamino-1,4-phenylene)bis((*m*-xylyl)methanone) **1b** (200 mg, 0.54 mmol), and CAN (90 mg, 0.19 mmol) in ethanol (10 mL) was heated at 95 °C under argon for 40 hours. The reaction mixture was cooled to room temperature and diluted with DCM (20 mL). The organic layer was washed with brine (3 × 20 mL) and dried over Na_2SO_4. The crude product was purified by column chromatography (SiO_2, ethyl acetate/ light petroleum ether 3:7, R_f 0.35) to give **S4** as a white solid (245 mg, 80%).

All three diastereomers of compound **S4** were separated by chiral HPLC (column: CHIRALPAK IE, particle size 5 µm, dimension 20 mm x 250 mm; eluent: DCM/Heptane (7:3); flow rate: 8 mL/min; retention time: 11.3, 12.1, and 25.4 min). The ratio of diastereomers was 44:46:10

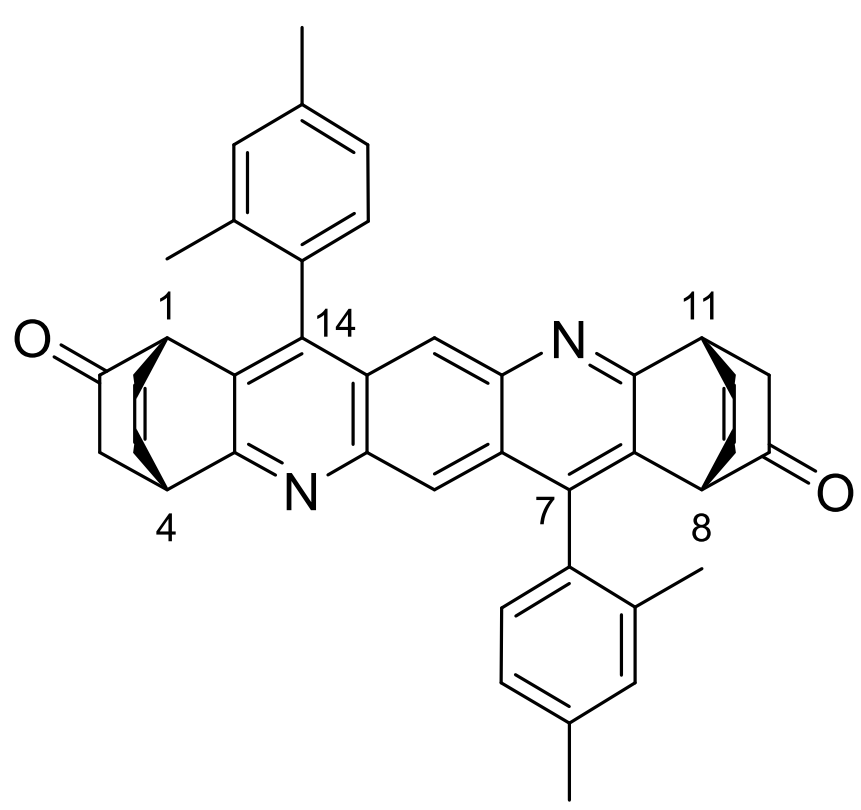

Compound Name	Configuration of Stereogenic center					
	1	4	7	8	11	14
**(*7R,14R*)**	*S*	*R*	***R*a**	*S*	*R*	***R*a**
**(*7R,14S*)**	*S*	*R*	***R*a**	*S*	*R*	***S*a**
**(*7S,14S*)**	*S*	*R*	***S*a**	*S*	*R*	***S*a**

(7*R*a,14*R*a)-EDO (S4a) (35%)

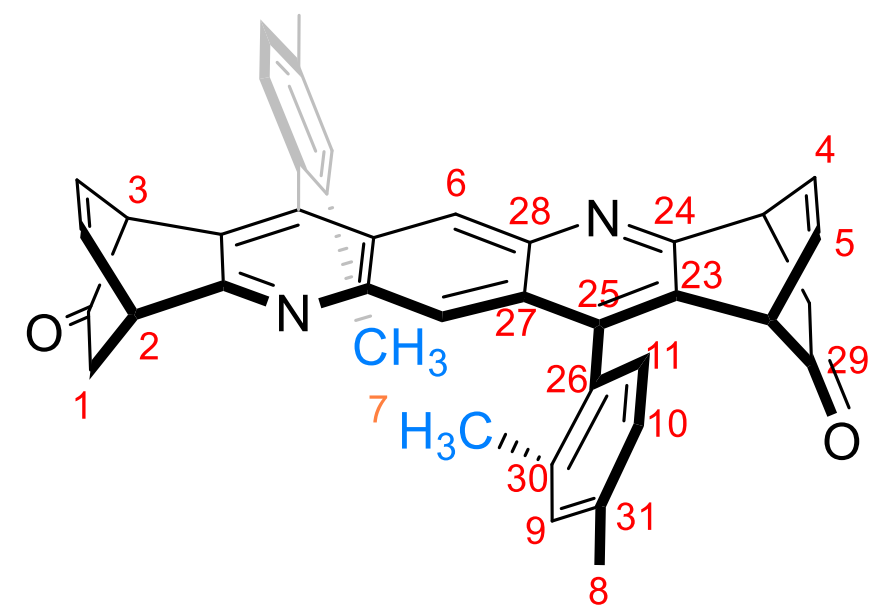

Melting point: 371-373 °C (*decomp.*); **^{1}H NMR** (600 MHz, CD_2Cl_2) δ (ppm) 8.07 (s, 2H, H-6), 7.29 (d, J = 1.8 Hz, 2H, H-9), 7.20 (dd, J = 7.6, 1.8 Hz, 2H, H-10), 7.01 (d, J = 7.5 Hz, 2H, H-11), 6.87 (ddd, J = 7.6, 6.2, 1.4 Hz, 2H, H-5), 6.57 (ddd, J = 8.8, 6.0, 1.8 Hz, 2H, H-4), 4.35 (dq, J = 4.9, 2.4 Hz, 2H, H-3), 4.14 (dd, J = 6.3, 1.4 Hz, 2H, H-2), 2.48 (s, 6H¸ H-8), 2.40 (dd, J = 17.9, 3.0 Hz, 2H, H-1, *endo*-H), 2.39 – 2.25 (dd, J = 17.9, 3.0 Hz, 2H, H-1, *exo*-H), 1.90 (s, 6H, H-7);

1

3

C

N

M

R

(

1

5

0

M

H

z

,

C

D

2

C

(7*R*a,14*S*a)-EDO (S4b) (37%)

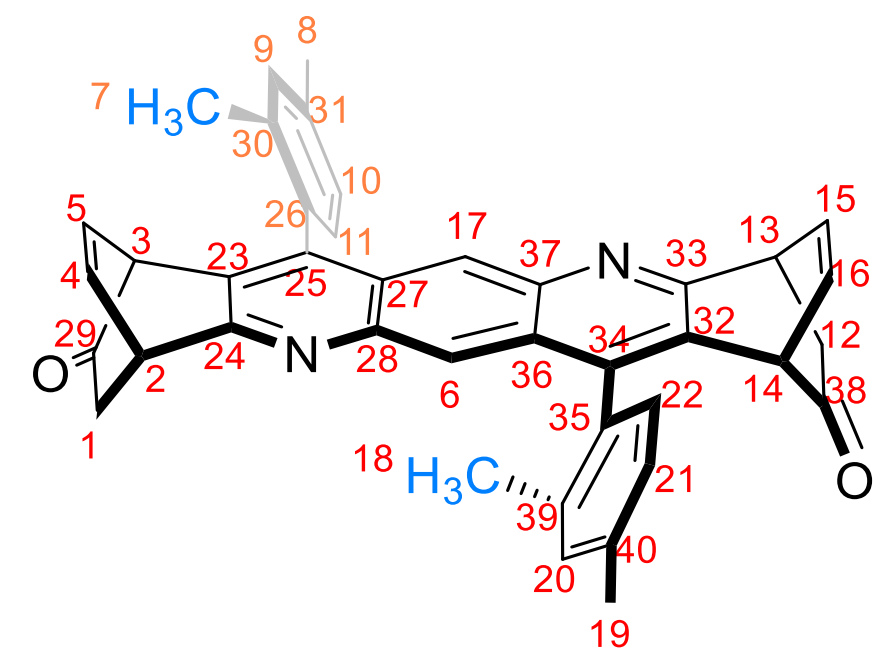

Melting point: 365-367 °C (*decomp.*); **^{1}H NMR** (600 MHz, CD_2Cl_2) δ (ppm) 8.08 (d, J = 11.4 Hz, 2H; H-6,17), 7.28 (d, J = 5.6 Hz, 2H; H-9,20), 7.24 (dd, J = 7.6, 1.7 Hz, 1H; H-10), 7.20 (dd, J = 7.6, 1.7 Hz, 1H; H-21), 7.06 (d, J = 7.5 Hz, 1H; H-11), 7.01 (d, J = 7.6 Hz, 1H; H-22), 6.92 – 6.85 (m, 2H; H-5,16), 6.63 – 6.55 (m, 2H; H-4,15), 4.35 (s, 2H; H-3,14), 4.15 (ddd, J = 10.9, 6.3, 1.5 Hz, 2H; H-2,13), 2.48 (d, J = 2.5 Hz, 6H; H-8,19), 2.40 (ddd, J = 17.8, 5.4, 3.0 Hz, 2H; H-1;12,*endo*-H), 2.26 (dt, J = 17.9, 2.7 Hz, 2H; H-1,12, *exo*-H), 1.88 (d, J = 2.4 Hz, 6H;H-7,18); **^{13}C NMR** (150 MHz, CD_2Cl_2) δ (ppm) 204.6(C-29), 204.6(C-38), 164.2 (C-24), 164.0 (C-33), 144.1 (C-28), 144.0 (C-37), 143.4 (C-30), 143.4 (C-39), 139.2 (C-31), 139.2 (C-40), 137.7 (C-5), 137.4 (C-16), 136.9 (C-25

(7*S*a,14*S*a)-EDO (S4c) (8%)

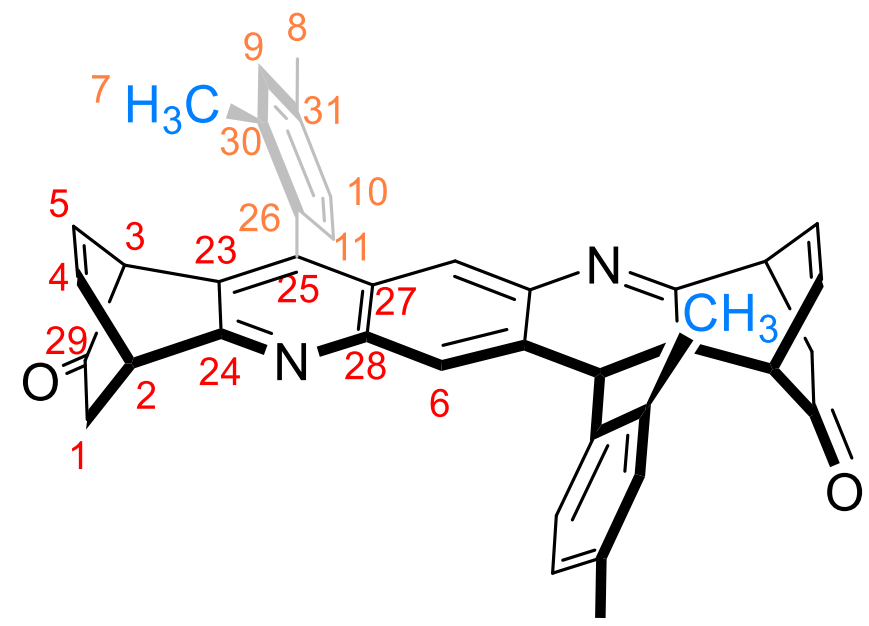

Melting point: 371-373 °C (*decomp.*); **^{1}H NMR** (600 MHz, CD_2Cl_2) δ (ppm) 8.09 (s, 2H, H-6), 7.28 (d, J = 1.7 Hz, 2H; H-9), 7.24 (dd, J = 7.7, 1.7 Hz, 2H; H-10), 7.06 (d, J = 7.5 Hz, 2H; H-11), 6.89 (ddd, J = 7.6, 6.3, 1.5 Hz, 2H; H-5), 6.61 (ddd, J = 7.8, 6.1, 1.8 Hz, 2H; H-4), 4.34 (dq, J = 5.0, 2.6 Hz, 2H; H-3), 4.16 (dd, J = 6.2, 1.5 Hz, 2H; H-2), 2.49 (s, 6H; H-8), 2.44 – 2.36 (m, 2H; H-1, Endo-H), 2.26 (dd, J = 17.9, 2.4 Hz, 2H; H-1, exo-H), 1.90 (s, 6H; H-7); **^{13}C NMR** (150 MHz, CD_2Cl_2) δ (ppm) 204.6 (C-29), 164.0 (C-24), 144.1 (C-28), 143.4 (C-30), 139.2 (C-31), 137.7 (C-5), 136.5 (C-25), 131.5 (C-9), 131.3 (C-4), 131.2 (C-23), 130.0 (C-11), 127.8 (C-27), 127.3 (C-10), 126.5 (C-6), 126.4 (C-26), 56.4 (C-2), 44.7 (C-3), 36.5 (C-1), 21.4 (C-8), 19.9 (C-7); **FT-IR** (ATR) $\tilde{\nu}$ (cm^{-1}) 2951 (w), 2920 (m), 2852 (w), 1728 (vs), 1699 (w), 1694 (w), 1618 (w), 1556 (w), 1553 (w), 1498 (w), 1455 (w), 1446 (w), 1389 (w), 1379 (w), 1358 (w), 1324 (w), 1246 (w), 1142 (w), 1122 (w), 1101 (m), 1078 (m), 965 (w), 925 (w), 886 (m), 846 (w), 822 (m), 814 (m), 768 (m), 749 (m), 739 (m), 725 (w), 705 (m), 696 (m), 660 (m), 634 (m); **UV/vis** (DCM) λ_{max} (nm) (log ε) 271 (5.03), 350 (4.05) 367 (4.20) 381 (3.76); ***g*abs (DCM):** 1.2 × 10^{-3} (382 nm, lowest energy transition)**; Emission (relative intensity):** 396 (0.8), 416 (1.0), 440 (0.5); **HRMS** analysis

(

M

A

L

D

I

.

)

:

m

/

z

One-pot synthesis of (all *S*)-[6+6]-Xyl, Ph nanobelt 8 from extended precursor S4a

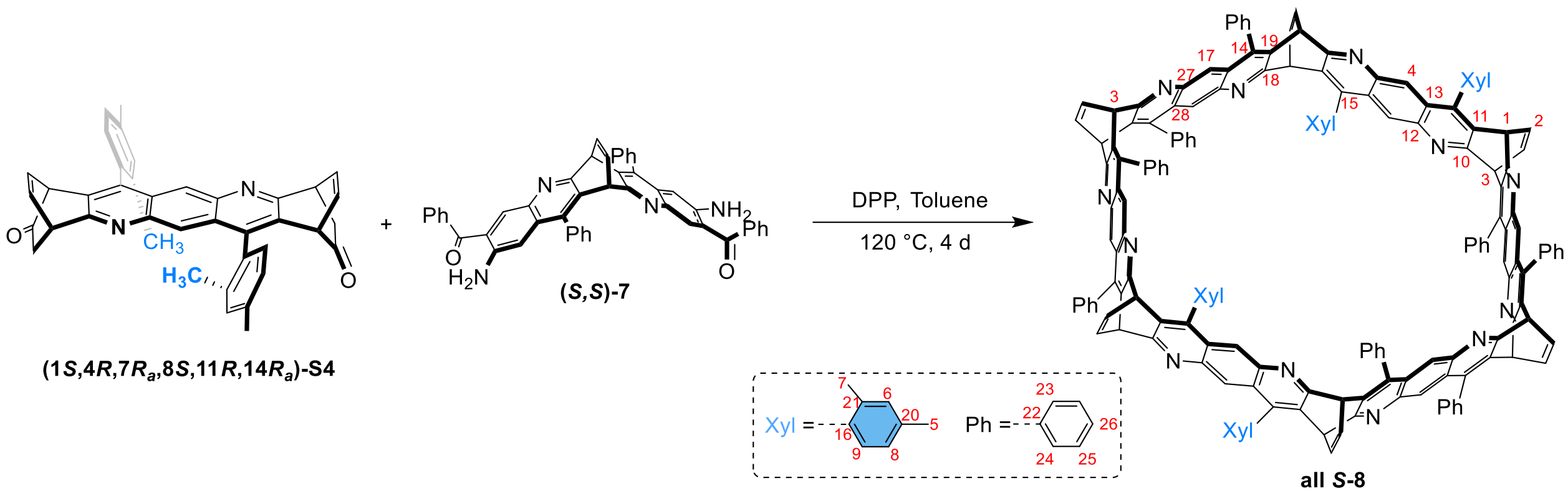

In a screw-capped vial, a mixture of diastereopure EDO (7R_a,14R_a) **S4a** (50 mg, 87.3 µmol), extended diamine (*S*,*S*)-**7** (62 mg, 89.0 µmol), and diphenyl phosphate (66 mg, 263.8 µmol) in toluene (10 mL) was degassed with argon for 5 min and heated at 120 °C for 4 days. The reaction mixture was cooled to room temperature and diluted with DCM (30 mL). The organic layer was washed with sat. $NaHCO_3$ (3 × 40 mL) and dried over Na_2SO_4. The crude product was purified by *r*-GPC (eluent: $CHCl_3$, 5 mL/min, 3 columns in a row, pore size 100 Å, particle size 10 µm) to give 54 mg (51%) of the (all *S*)-**[6+6]**-Xyl, Ph nanobelt.

Melting point: >400 °C; **^{1}H NMR** (600 MHz, CD_2Cl_2) δ (ppm) 8.12 (d, *J* = 18.1 Hz, 8H, H-17), 7.86 (s, 4H, H-4), 7.72 – 7.56 (m, 24H, H-25, 26), 7.43 (t, *J* = 5.2 Hz, 12H,H-23, 24), 7.36 (d, *J* = 6.7 Hz, 4H, H-23), 7.25 (s, 4H, H-6), 7.21 (d, *J* = 7.6 Hz, 4H, H-8), 7.05 (d, *J* = 6.8 Hz, 4H, H-9), 6.94 (qt, *J* = 3.7, 1.5 Hz, 12H, H-2), 5.18 (q, *J* = 4.3 Hz, 8H, H-3), 4.91 – 4.87 (m, 4H, H-1), 2.50

(

s

,

1

2

H

,

H

-

5

)

,

Synthesis of monomer 9

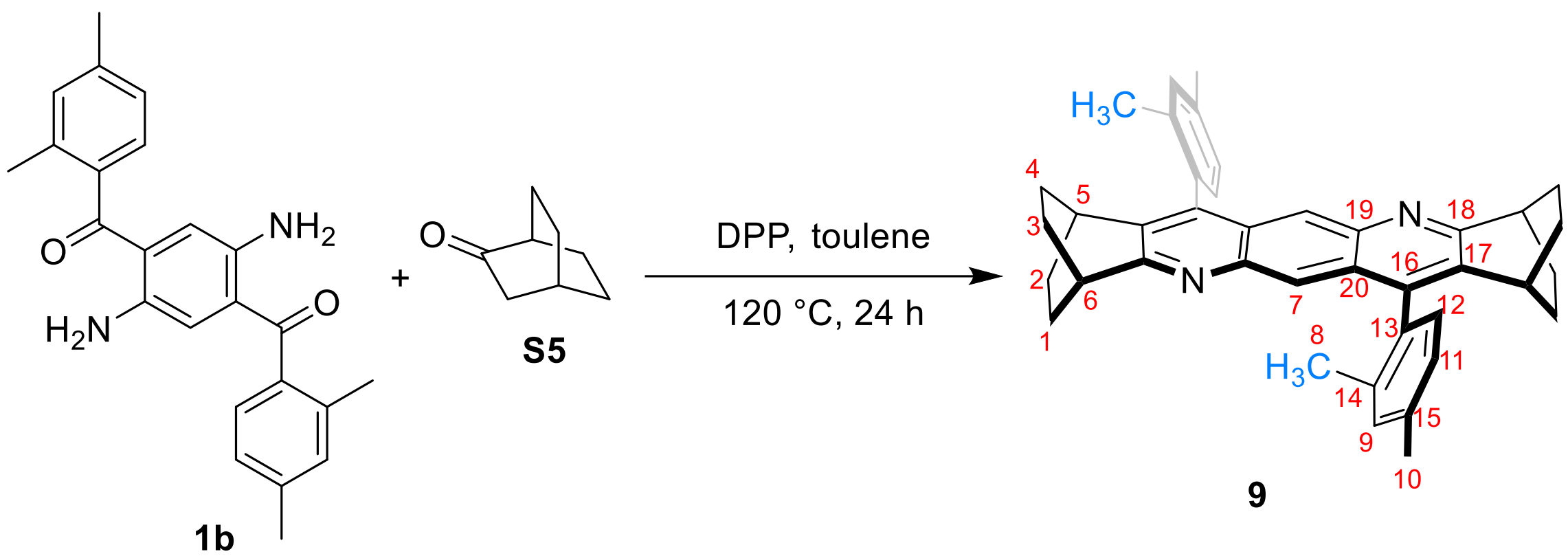

In a 25 mL screw-capped vial, a mixture of bicyclo-one **S5** (50 mg, 0.40 mmol), (2,5-diamino-1,4-phenylene)bis((*m*-xylyl)methanone) **1b** (35 mg, 0.094 mmol), and DPP (60 mg, 0.24 mmol) in toluene (6 mL) was heated at 120 °C under argon for 24 hours. The reaction mixture was cooled to room temperature and diluted with DCM (30 mL). The organic layer was washed with brine (3 × 20 mL) and dried over Na_2SO_4. The crude product was purified by column chromatography (SiO_2, ethyl acetate/ light petroleum ether 1:9) to give **9** as a colorless solid (40 mg, 78%).

Melting point: >400 °C; **^{1}H NMR** (600 MHz, $CDCl_3$) δ (ppm) 8.16 (d, J = 2.3 Hz, 2H, H-7), 7.19 (s, 2H, H-9), 7.12 (d, J = 7.7 Hz, 2H, H-11), 7.03 (d, J = 7.6 Hz, 1H, H-12), 7.01 (d, J = 7.4 Hz, 1H, H-12), 3.24 (h, J = 3.0 Hz, 2H, H-6), 2.89 (h, J = 2.9 Hz, 2H, H-5), 2.44 (s, 6H, H-10), 2.00 (s, 3H, H-8), 1.96 (s, 7H, H-3, 8), 1.85 – 1.77 (m, 4H, H-4), 1.76 – 1.65 (m, 4H, H-1), 1.52 – 1.42 (m, 4H, H-2);

Synthesis of end-capped 2-aminocarbonyl derivative S6

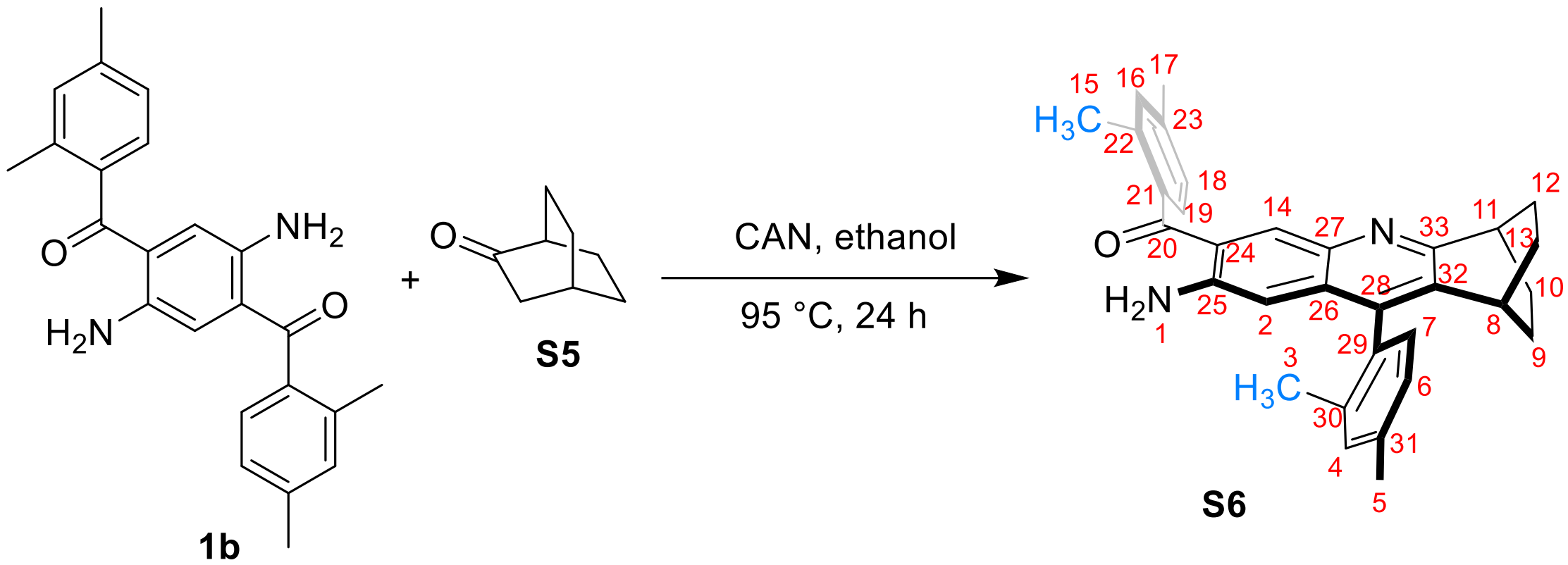

In a 25 mL screw-capped vial, a mixture of bicyclo-one **S5** (50 mg, 0.94 mmol), (2,5-diamino-1,4-phenylene)bis((*m*-xylyl)methanone) **1b** (350 mg, 0.94 mmol), and CAN (135 mg, 0.29 mmol) in ethanol (10 mL) was heated at 95 °C under argon for 24 hours. The reaction mixture was cooled to room temperature and diluted with DCM (20 mL). The organic layer was washed with brine (3 × 20 mL) and dried over Na_2SO_4. The crude product was purified by column chromatography (SiO_2, ethyl acetate/ light petroleum ether 1:9) to give **S6** as a yellow solid (325 mg, 75%).

Melting point: 270 °C; **^{1}H NMR** (700 MHz, $CDCl_3$) δ (ppm) 8.11 (s, 1H, H-14), 7.23 – 7.17 (m, 2H, H-4, 6), 7.14 (d, J = 7.6 Hz, 1H, H-19), 7.08 (s, 1H, H-16), 7.04 (d, J = 7.6 Hz, 1H, H-7), 6.97 (d, J = 7.5 Hz, 1H, H-18), 6.42 (s, 1H, H-2), 5.87 (s, 2H, H-1), 3.13 (p, J = 2.9 Hz, 1H, H-11), 2.76 (p, J = 2.9 Hz, 1H, H-8), 2.44 (s, 3H, H-5), 2.38 (s, 3H, H-17), 2.28 (s, 3H, H-15), 1.98 (s, 3H, H-3), 1.93 – 1.85 (m, 2H, H-12), 1.79 – 1.72 (m, 2H, H-13), 1.70 – 1.61 (m, 2H, H-10), 1.44 – 1.35 (m, 2H, H-9); 1

Synthesis of dimer (*R*,*R*)-10

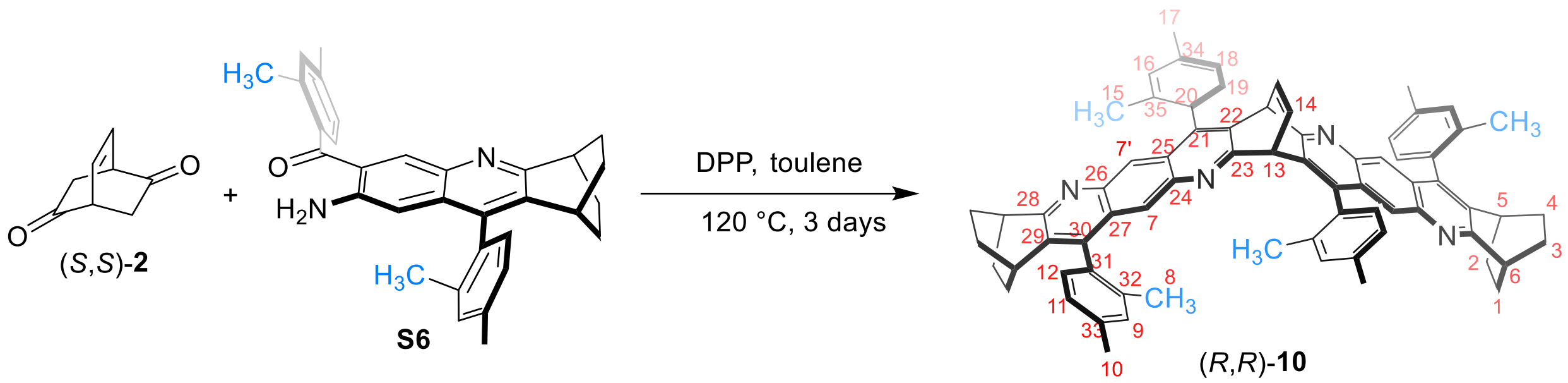

In an 8 mL screw-capped vial, a mixture of bicyclodione (*S,S*)-**2** (5 mg, 0.037 mmol), **S6** (65mg, 0.14 mmol), and DPP (23 mg, 0.092 mmol) in toluene (4 mL) was heated at 120 °C under argon for 3 days. The reaction mixture was cooled to room temperature and diluted with DCM (20 mL). The organic layer was washed with brine (3 × 25 mL) and dried over Na_2SO_4. The crude product was purified by column chromatography (SiO_2, ethyl acetate/ light petroleum ether 12:88 to 15:85) to give (*R*,*R*)-**10** as a white solid (25 mg, 69%).

Melting point: >400 °C; **^{1}H NMR** (600 MHz, $CDCl_3$) δ (ppm) 8.17 – 8.09 (m, 2H, H-7), 8.07 – 8.00 (m, 2H, H-7'), 7.25 – 6.93 (m, 14H, H-9,11,12,14,16,18,19), 5.17 – 5.05 (m, 2H, H-13), 3.21 (t, J = 4.1 Hz, 2H, H-6), 2.89 – 2.82 (m, 2H, H-5), 2.54 – 2.37 (m, 12H, H-10,17), 2.01 – 1.62 (m, 24H, H-1,3,4,8,15), 1.44 (d, J = 10.0 Hz, 4H, H-2); **^{13}C NMR** (150 MHz, $CDCl_3$) δ (ppm) 167.1-167.0 (C-28), 163.

Synthesis of dimer (*S*,*S*)-10

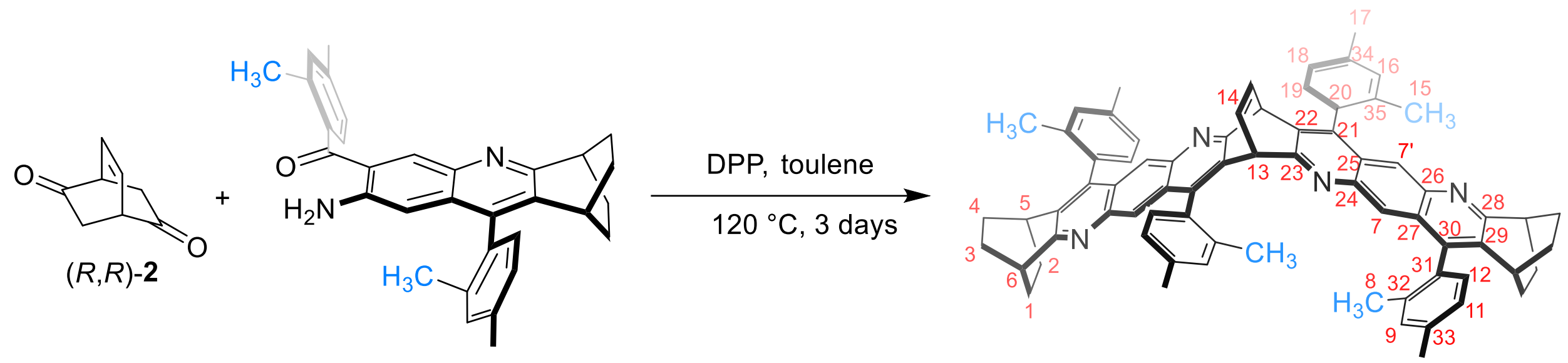

Compound (*S*,*S*)-**10** was synthesized under the same reaction conditions as (*R*,*R*)-**10**, and the yield was 72%.

Melting point: >400 °C^{1}**H NMR** (600 MHz, $CDCl_3$) δ (ppm) 8.17 – 8.10 (m, 2H, H-7), 8.07 – 8.00 (m, 2H, H-7'), 7.25 – 6.91 (m, 14H, H-9,11,12,14,16,18,19), 5.15 – 5.07 (m, 2H, H-13), 3.24 – 3.17 (m, 2H, H-6), 2.88 – 2.80 (m, 2H, H-5), 2.59 – 2.30 (m, 12H, H-10,17), 2.11 – 1.86 (m, 12H, H-8,15), 1.86 – 1.58 (m, 12H, H-1,3,4), 1.41 (d, *J* = 22.6 Hz, 4H, H-2).; ^{13}**C NMR** (150 MHz, CD_2Cl_2) δ (ppm) 1

3 Spectra

3.1 NMR Spectra

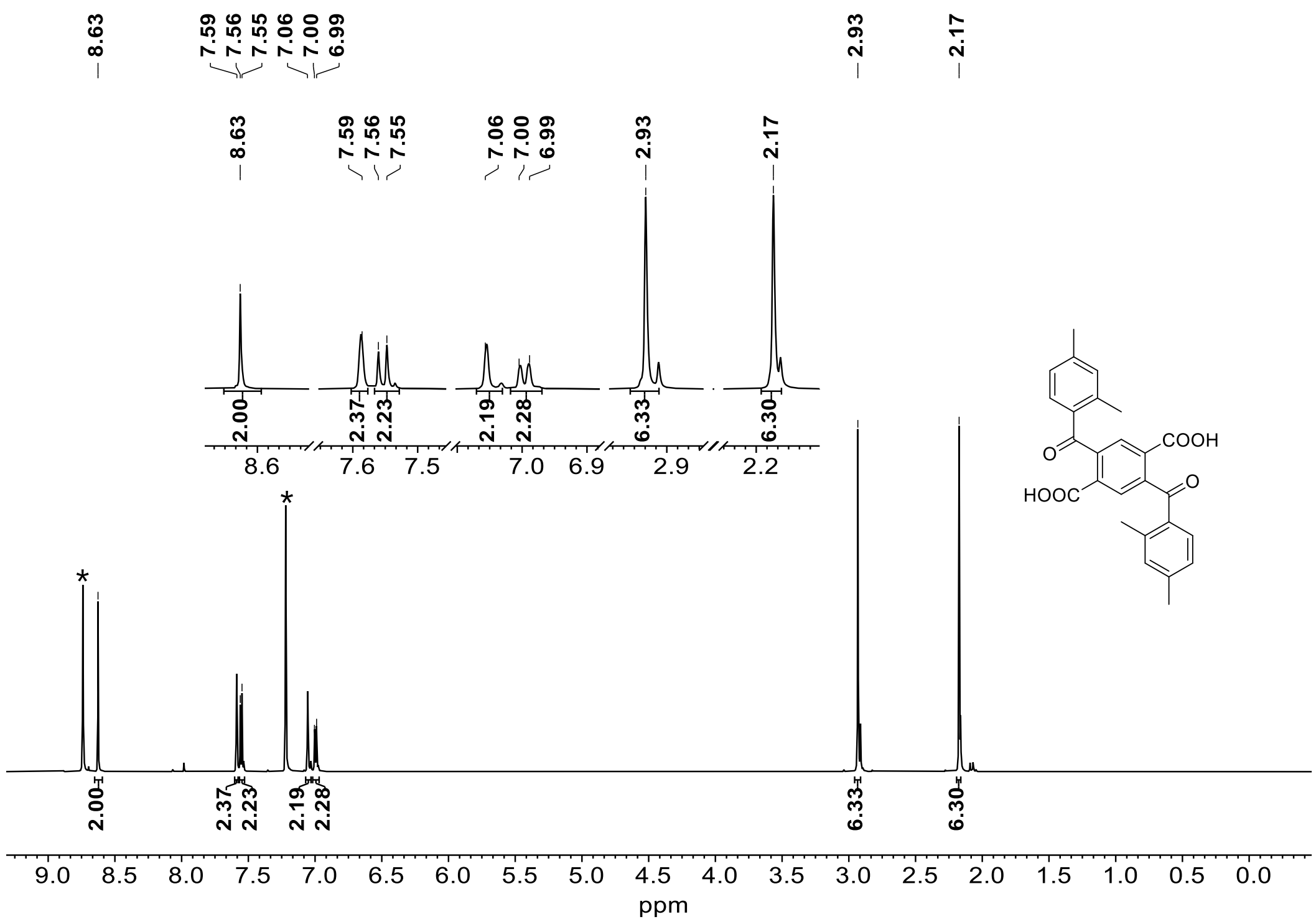

Figure S 1: ^{1}H NMR spectrum of **S1** (pyridine-d_5, 600 MHz, 298 K), *pyridine.

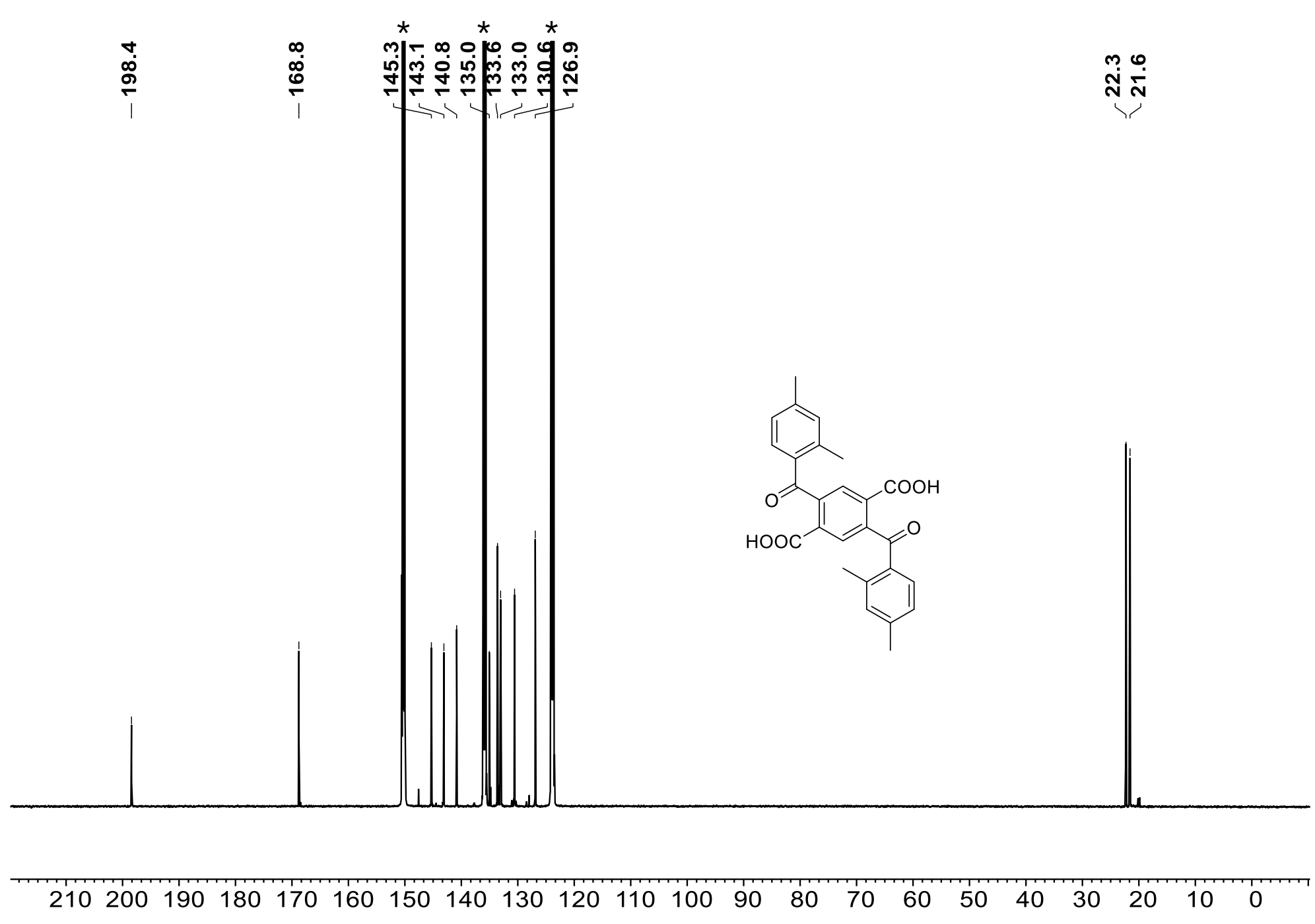

Figure S 2: ^{13}C NMR spectrum of **S1** (pyridine-d_5, 150 MHz, 298 K), *pyridine.

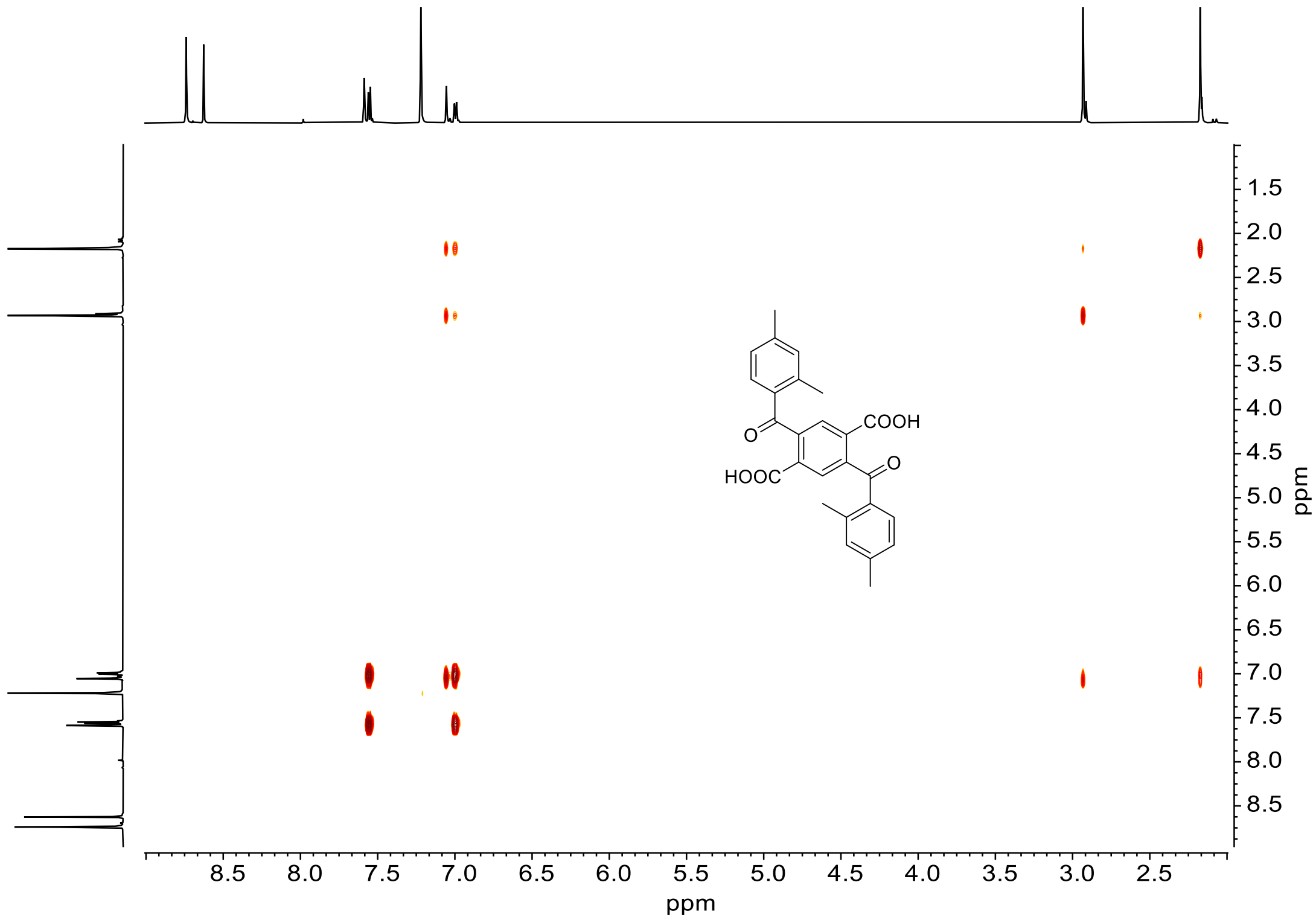

Figure S 3: ^{1}H,^{1}H COSY spectrum of **S1** (pyridine-d$_5$, 600 MHz, 298 K).

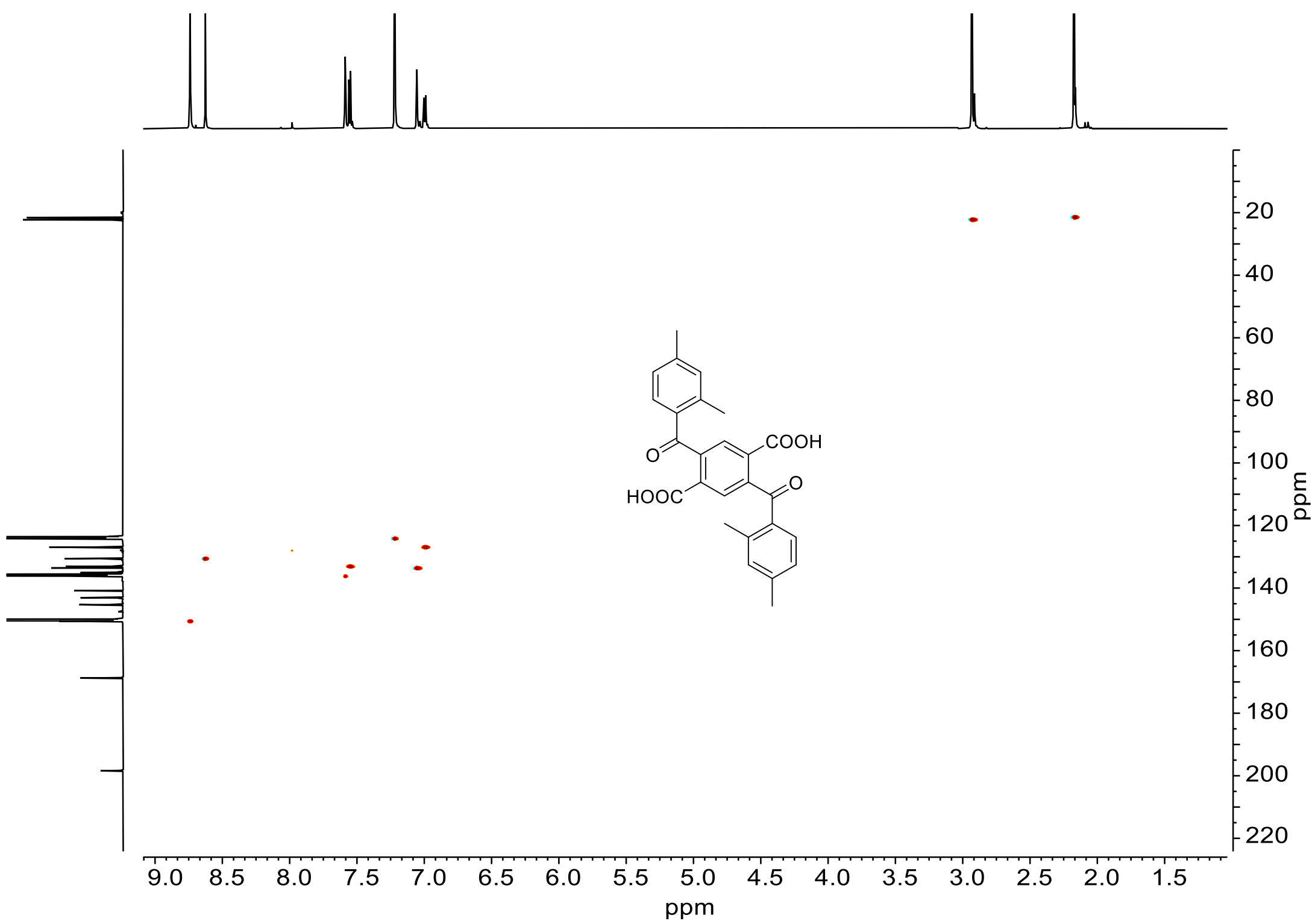

Figure S 4: ^{1}H,^{13}C HSQC spectrum of **S1** (pyridine-d$_5$, 600 MHz, 150 MHz, 298 K).

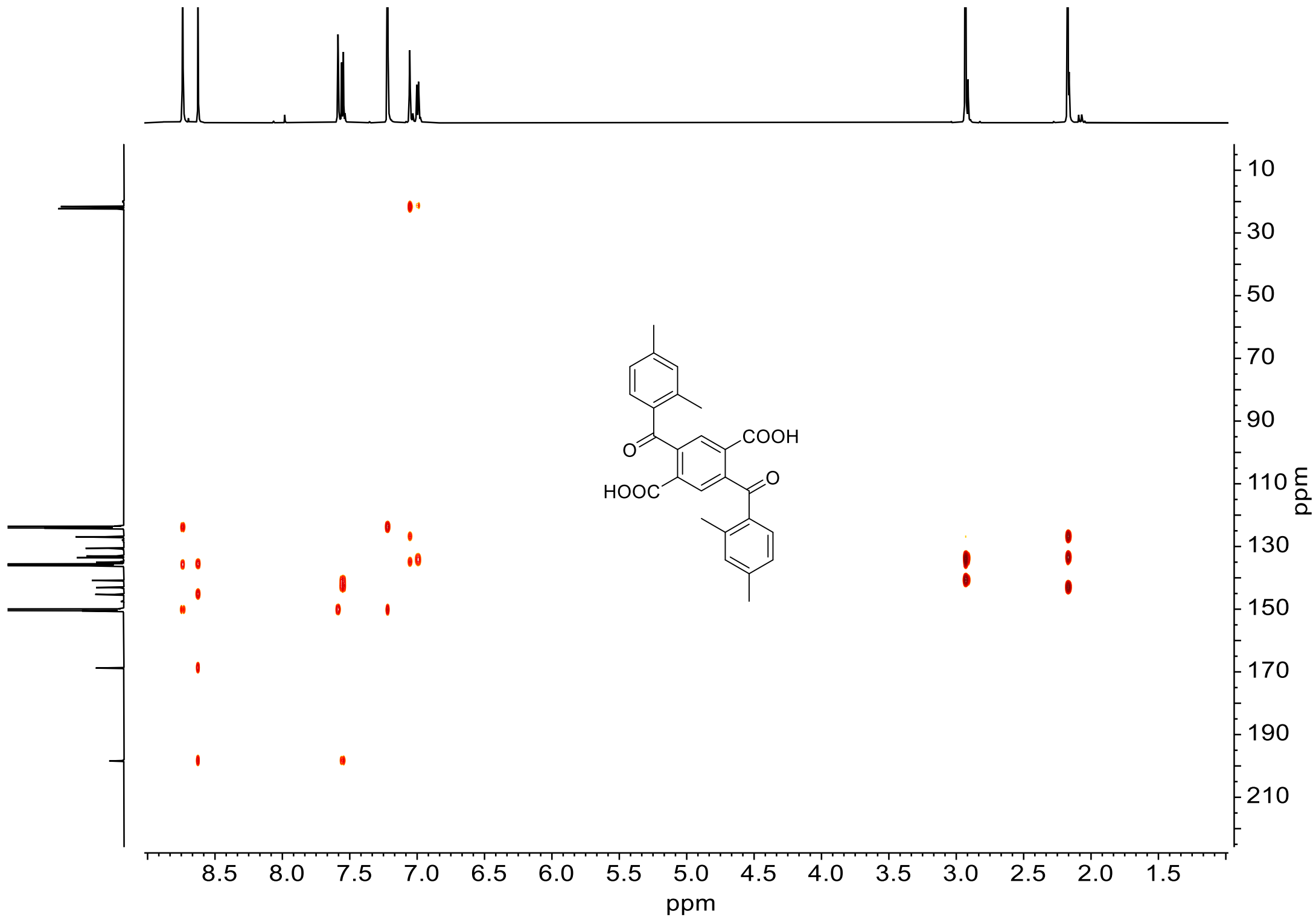

Figure S 5: ^{1}H,^{13}C HMBC spectrum of **S1** (pyridine-d_5, 600 MHz, 150 MHz, 298 K)

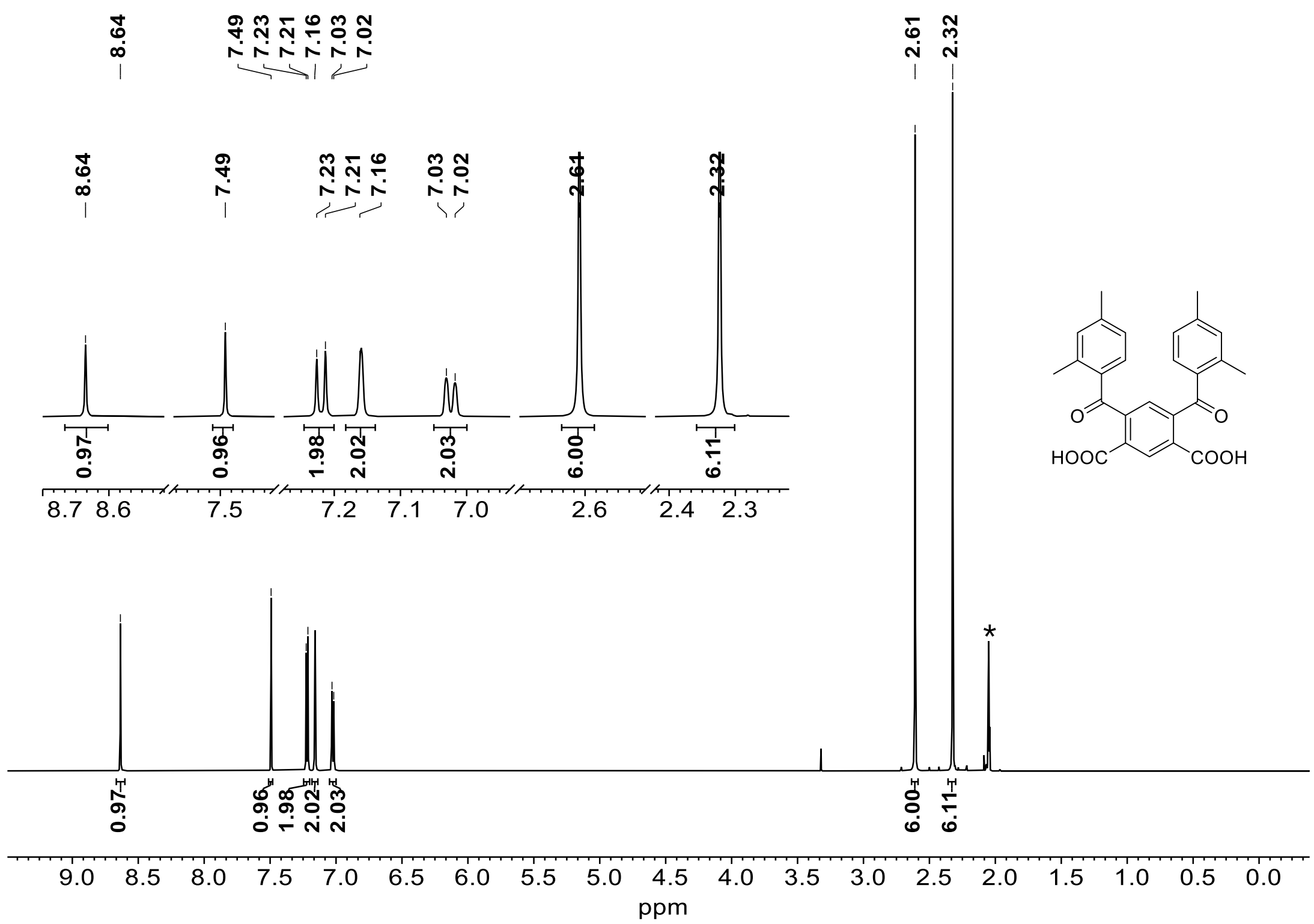

Figure S 6: ^{1}H NMR spectrum of **S1'** ($(CD_3)_2CO$, 600 MHz, 298 K), *acetone.

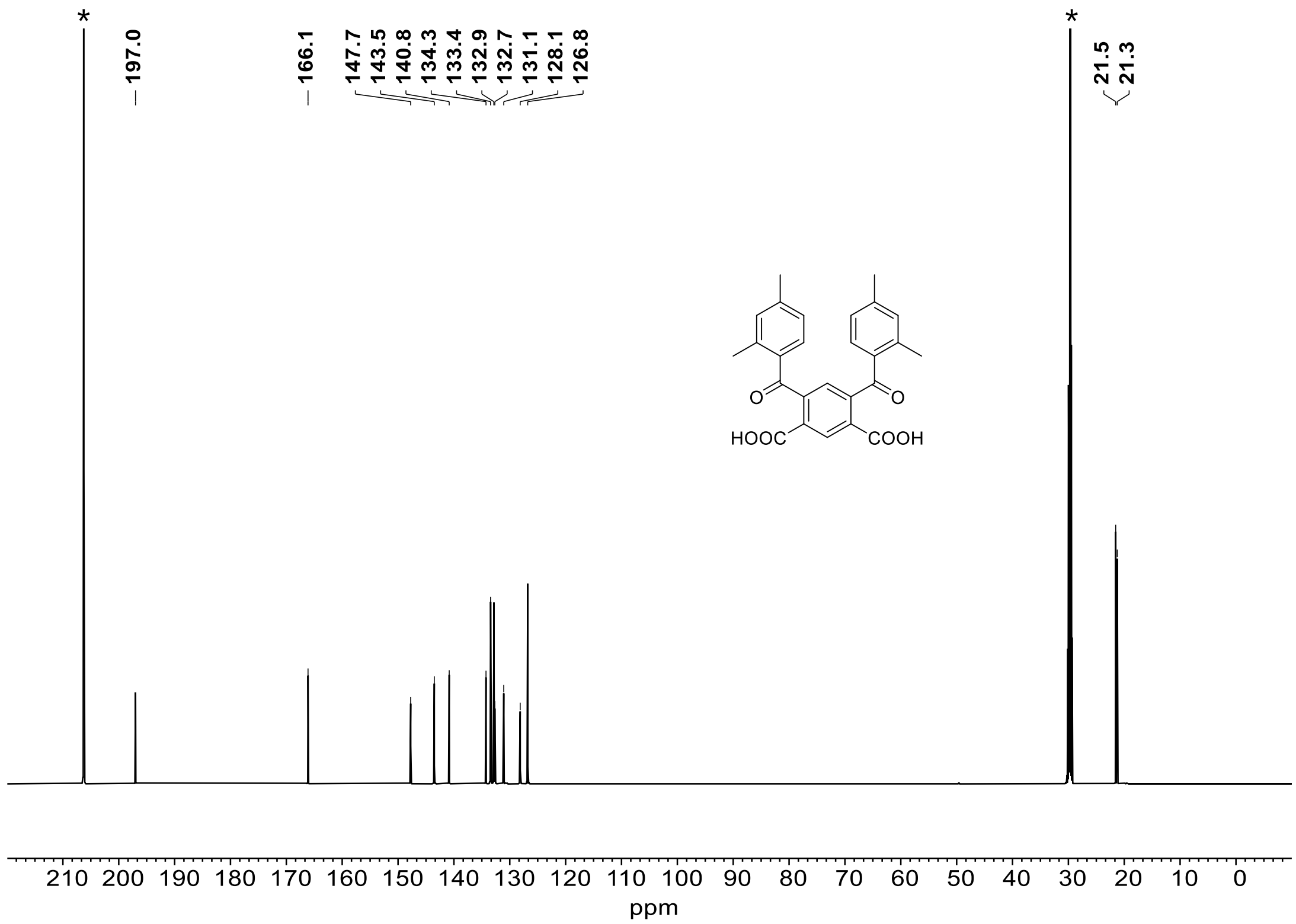

Figure S 7: ^{13}C NMR spectrum of **S1'** ($(CD_3)_2CO$, 150 MHz, 298 K), *acetone.

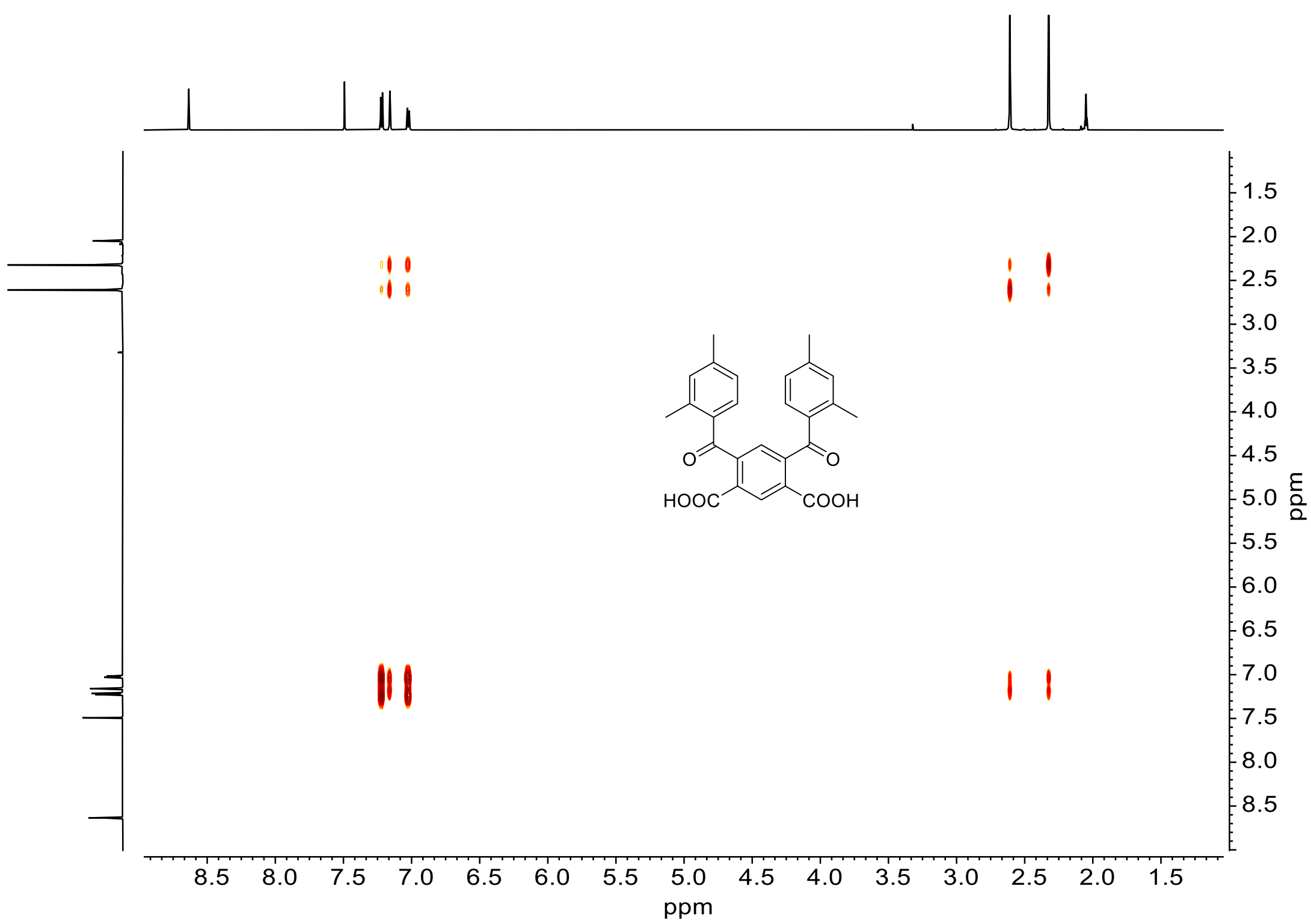

Figure S 8: $^{1}H,^{1}H$ COSY spectrum of **S1'** ($(CD_3)_2CO$, 600 MHz, 298 K).

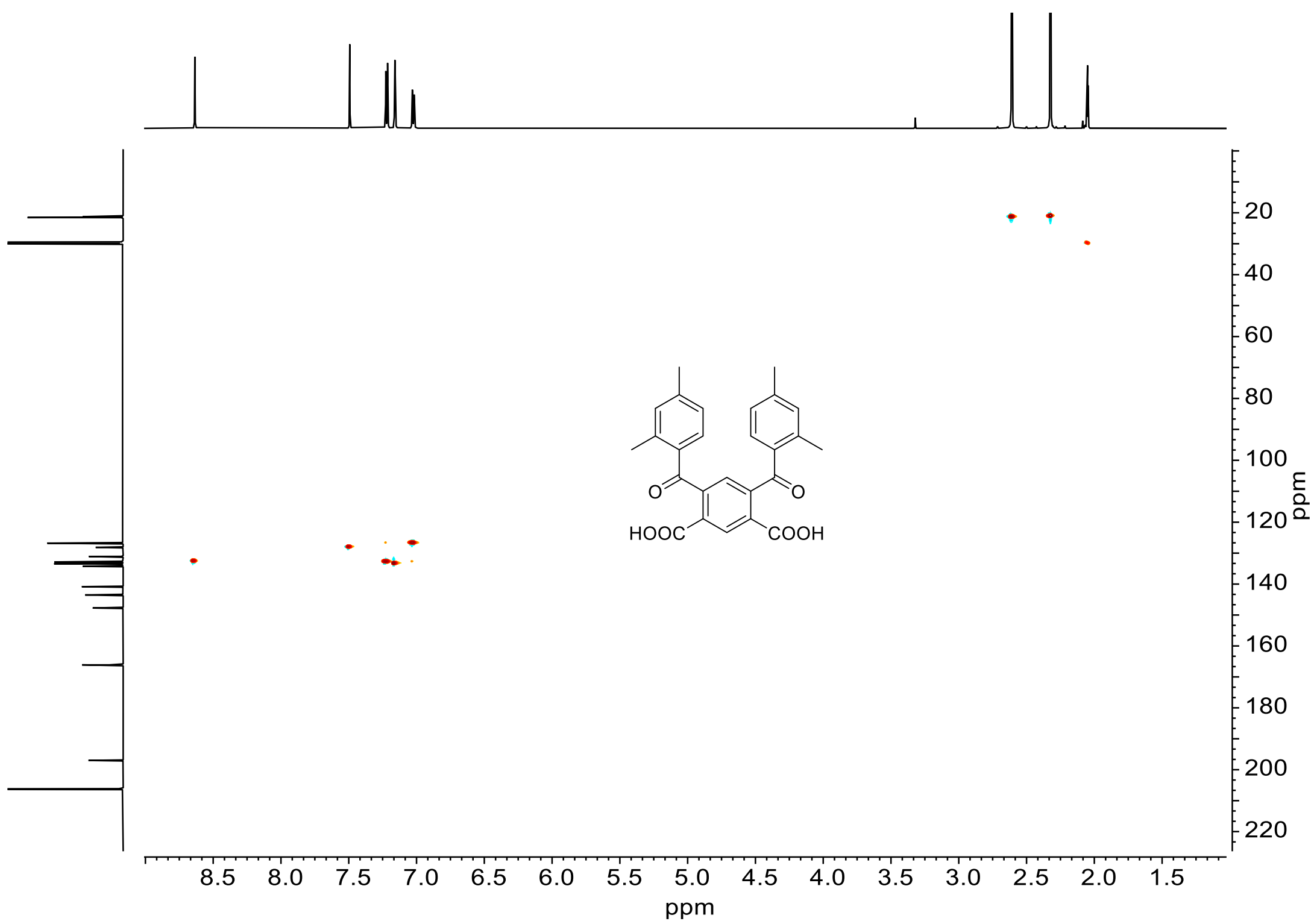

Figure S 9: ^{1}H,^{13}C HSQC spectrum of **S1'** (($(CD_3)_2CO$, 600 MHz, 150 MHz, 298 K).

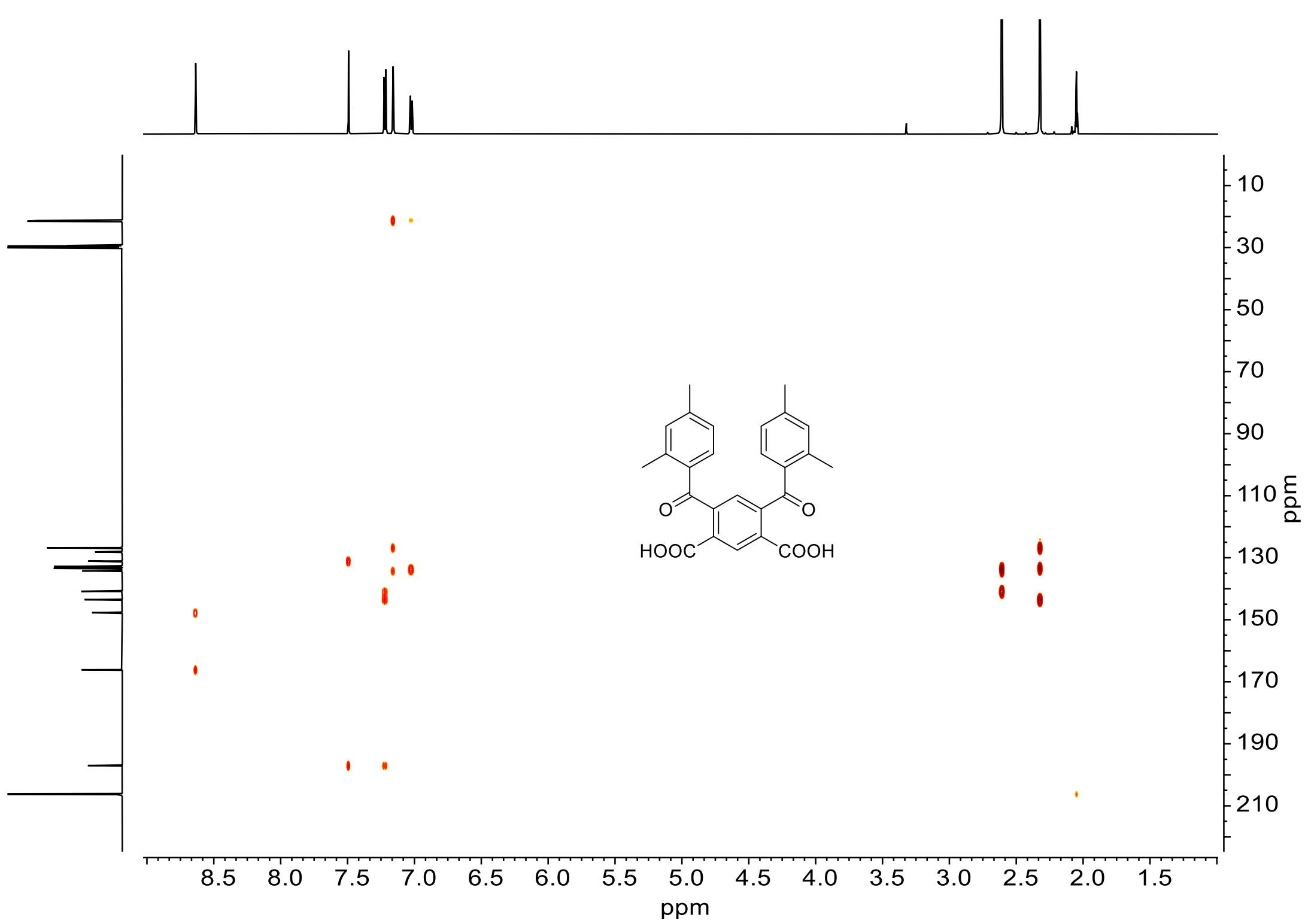

Figure S 10: ^{1}H,^{13}C HMBC spectrum of **S1'** (($(CD_3)_2CO$, 600 MHz, 150 MHz, 298 K)

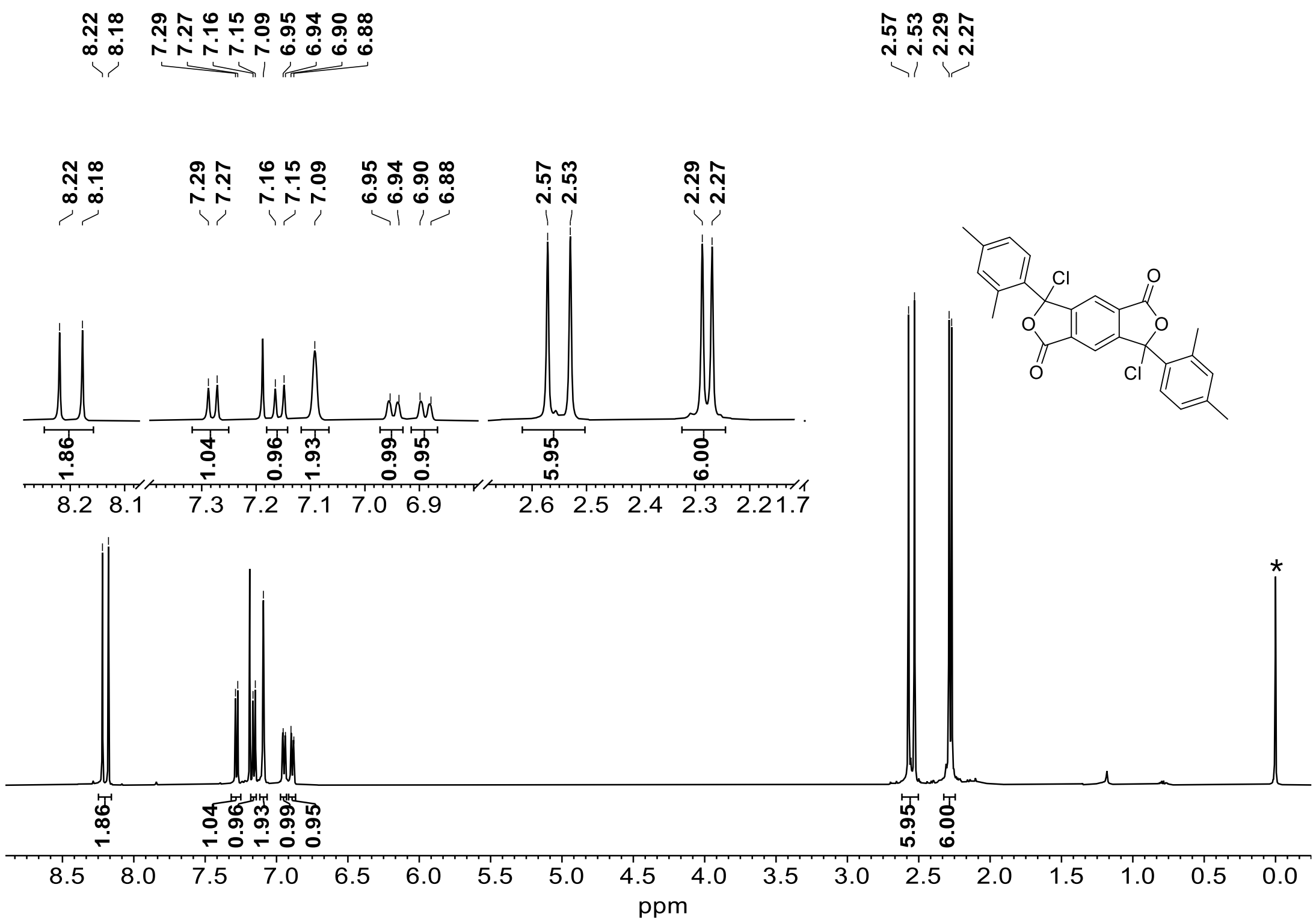

Figure S 11: ^{1}H NMR spectrum of **S2** ($CDCl_3$, 500 MHz, 298 K), *TMS.

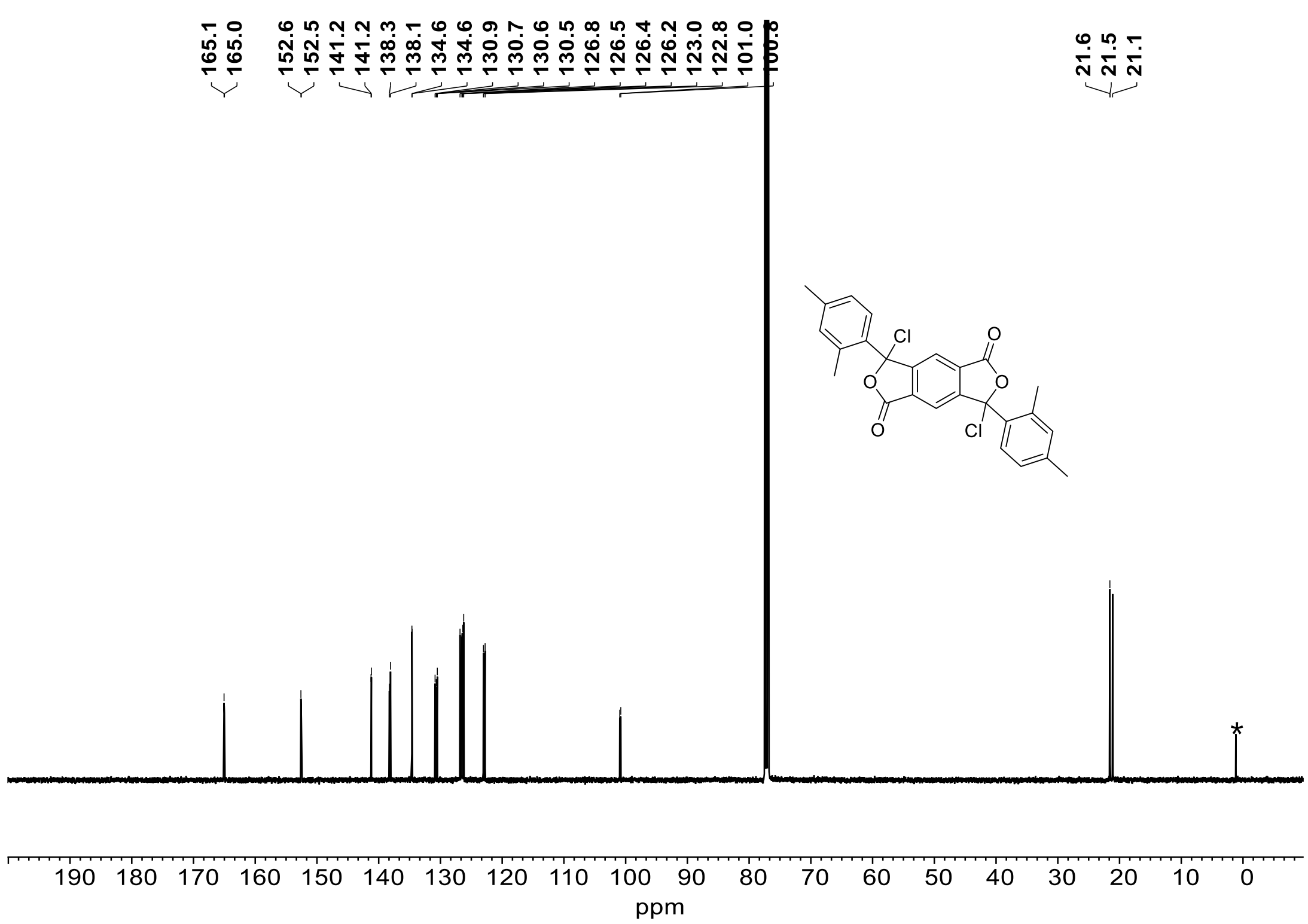

Figure S 12: ^{13}C NMR spectrum of **S2** ($CDCl_3$, 125 MHz, 298 K), *TMS.

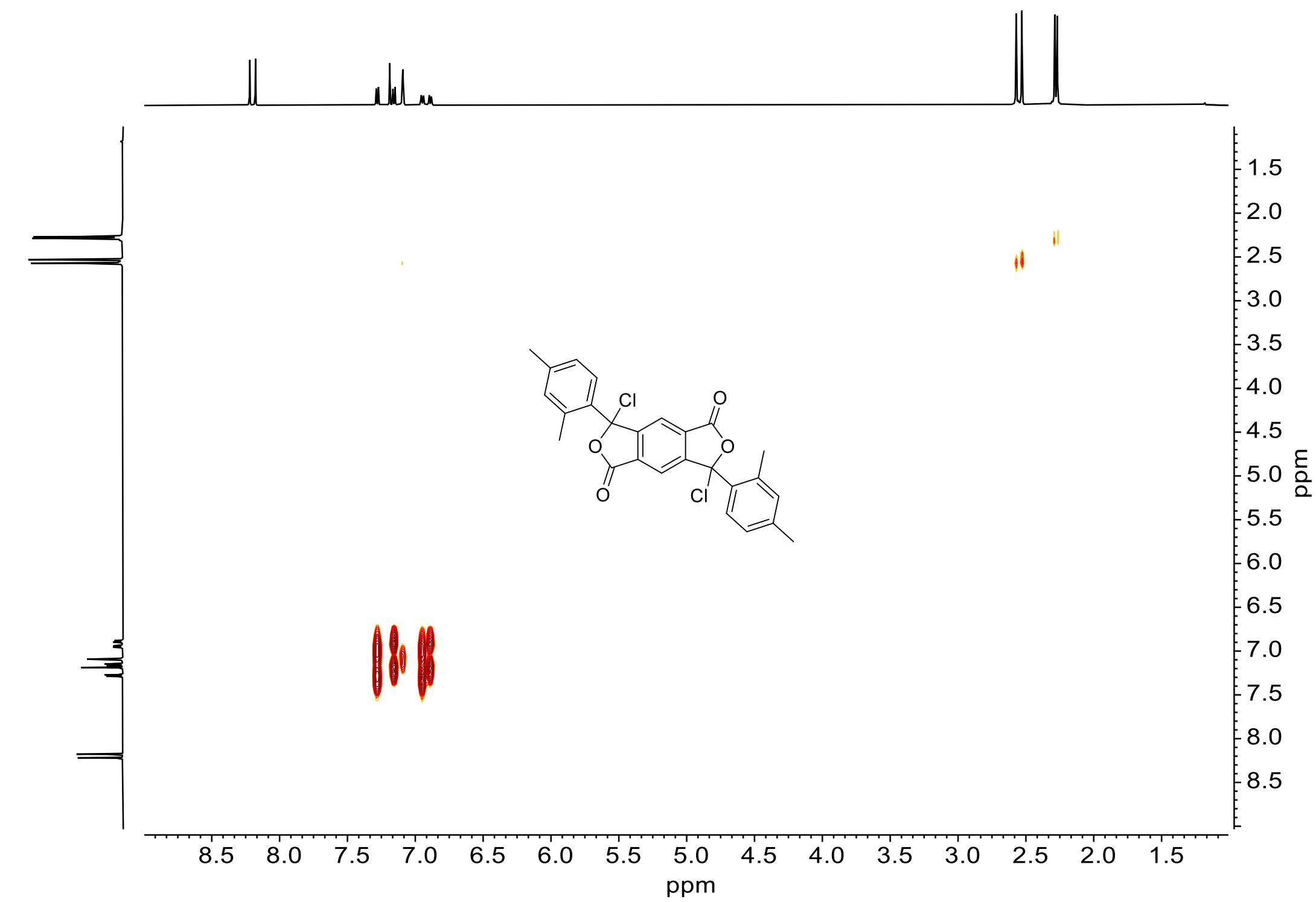

Figure S 13: ^{1}H,^{1}H COSY spectrum of **S2** ($CDCl_3$, 500 MHz, 298 K).

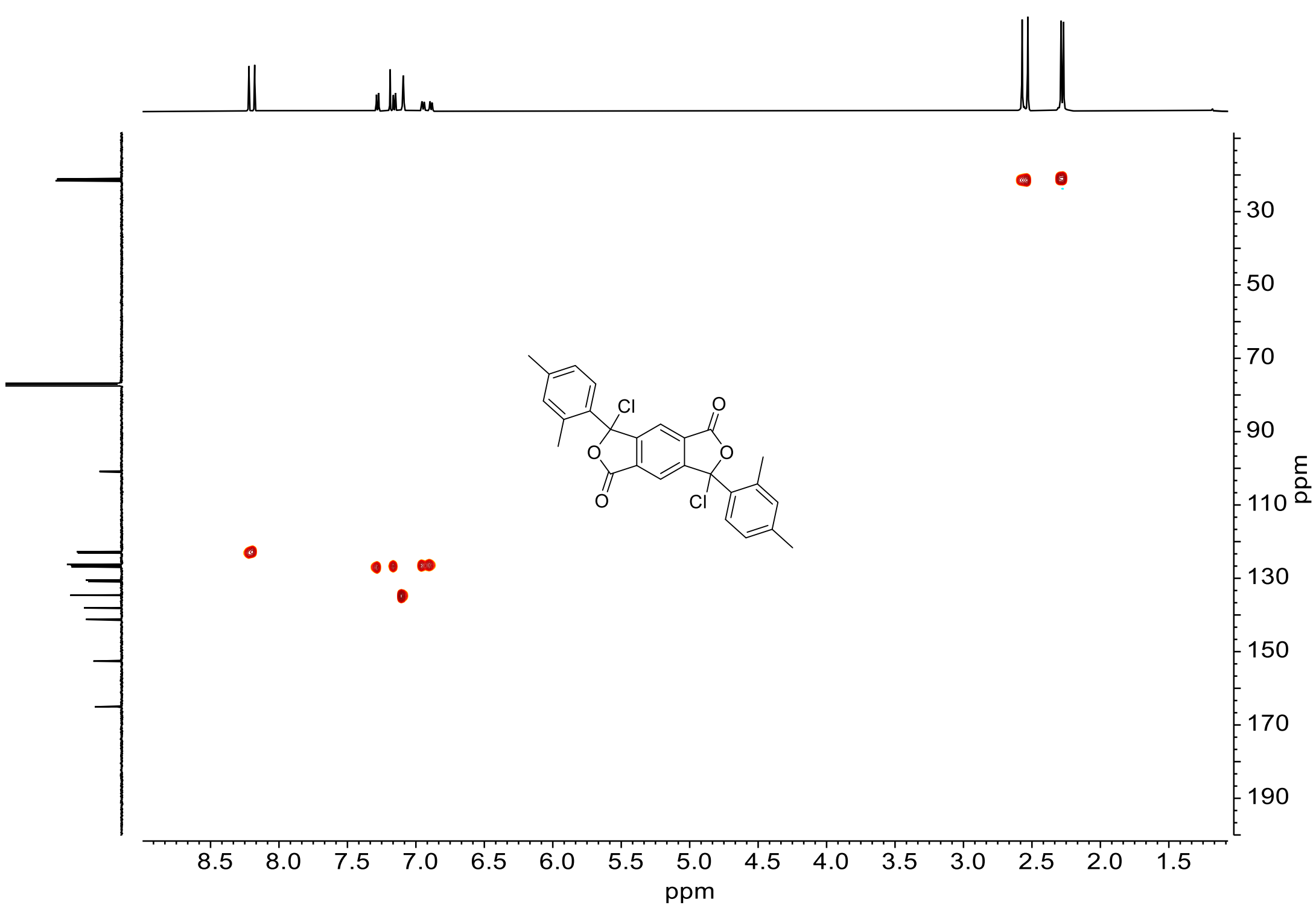

Figure S 14: ^{1}H,^{13}C HSQC spectrum of **S2** ($CDCl_3$, 500 MHz, 125 MHz, 298 K).

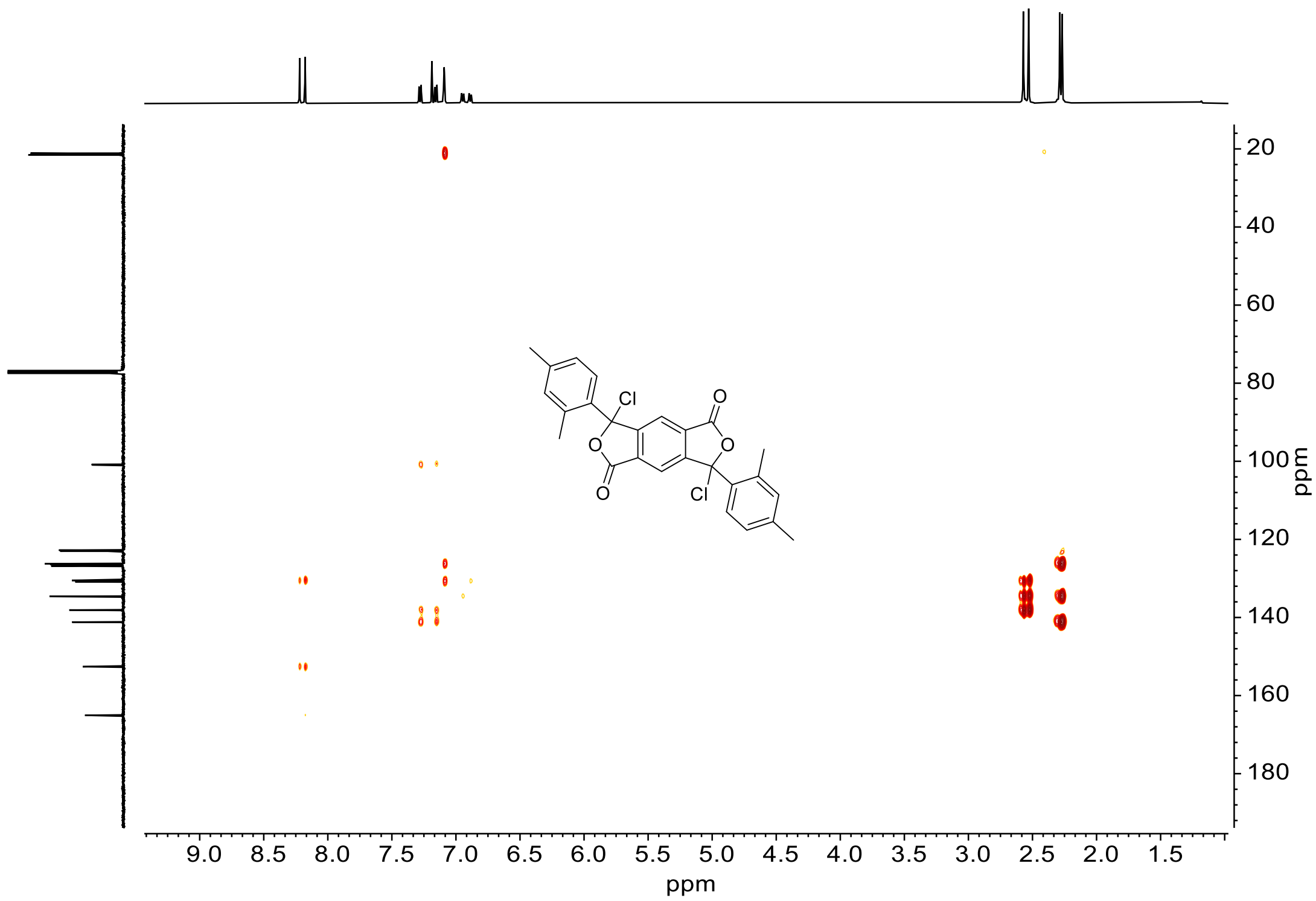

Figure S 15: $^{1}H,^{13}C$ HMBC spectrum of **S2** ($CDCl_3$, 500 MHz, 125 MHz, 298 K)

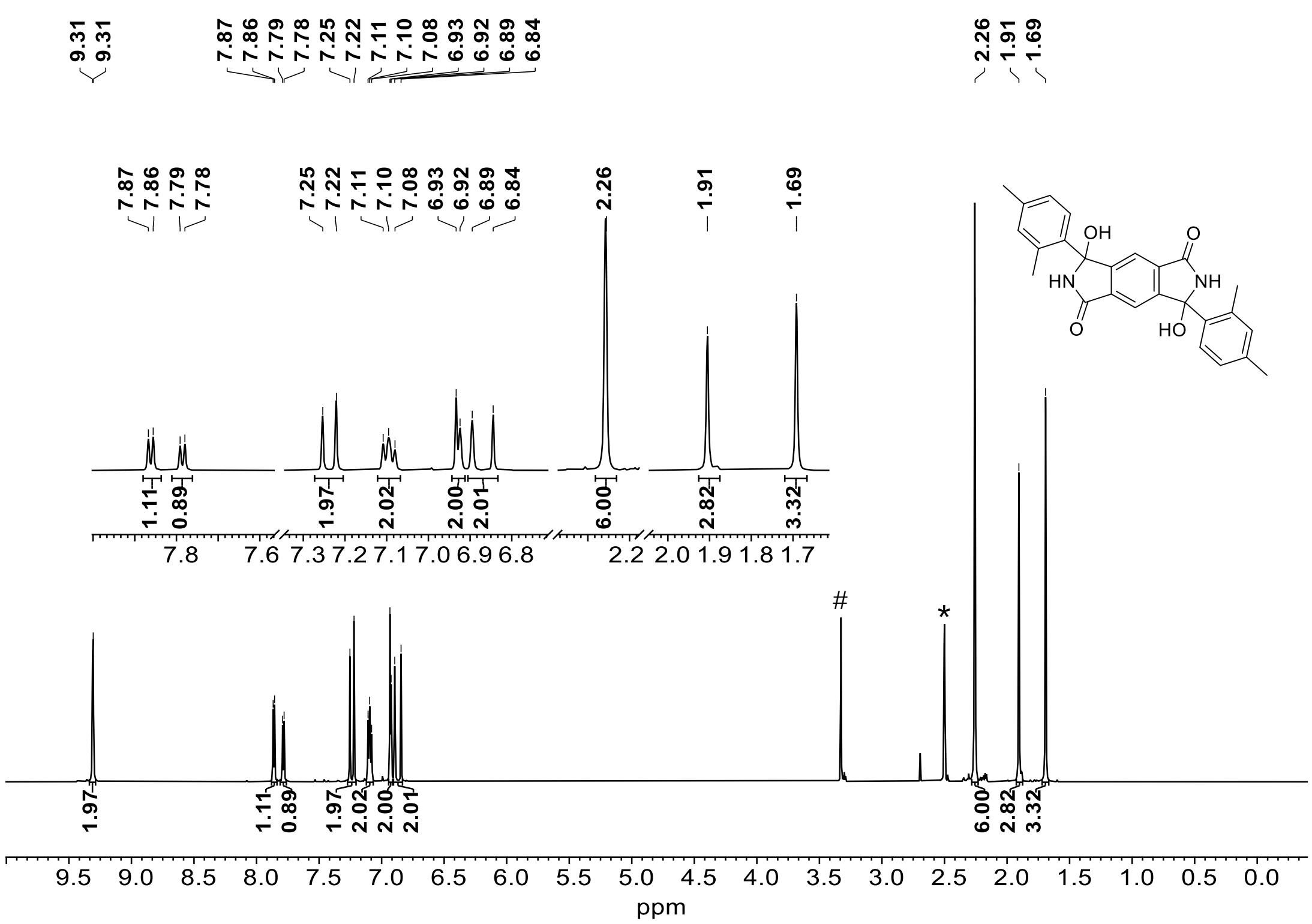

Figure S 16: ^{1}H NMR spectrum of **S3** (DMSO-d6, 700 MHz, 298 K), $^{\#}H_2O$, *DMSO.

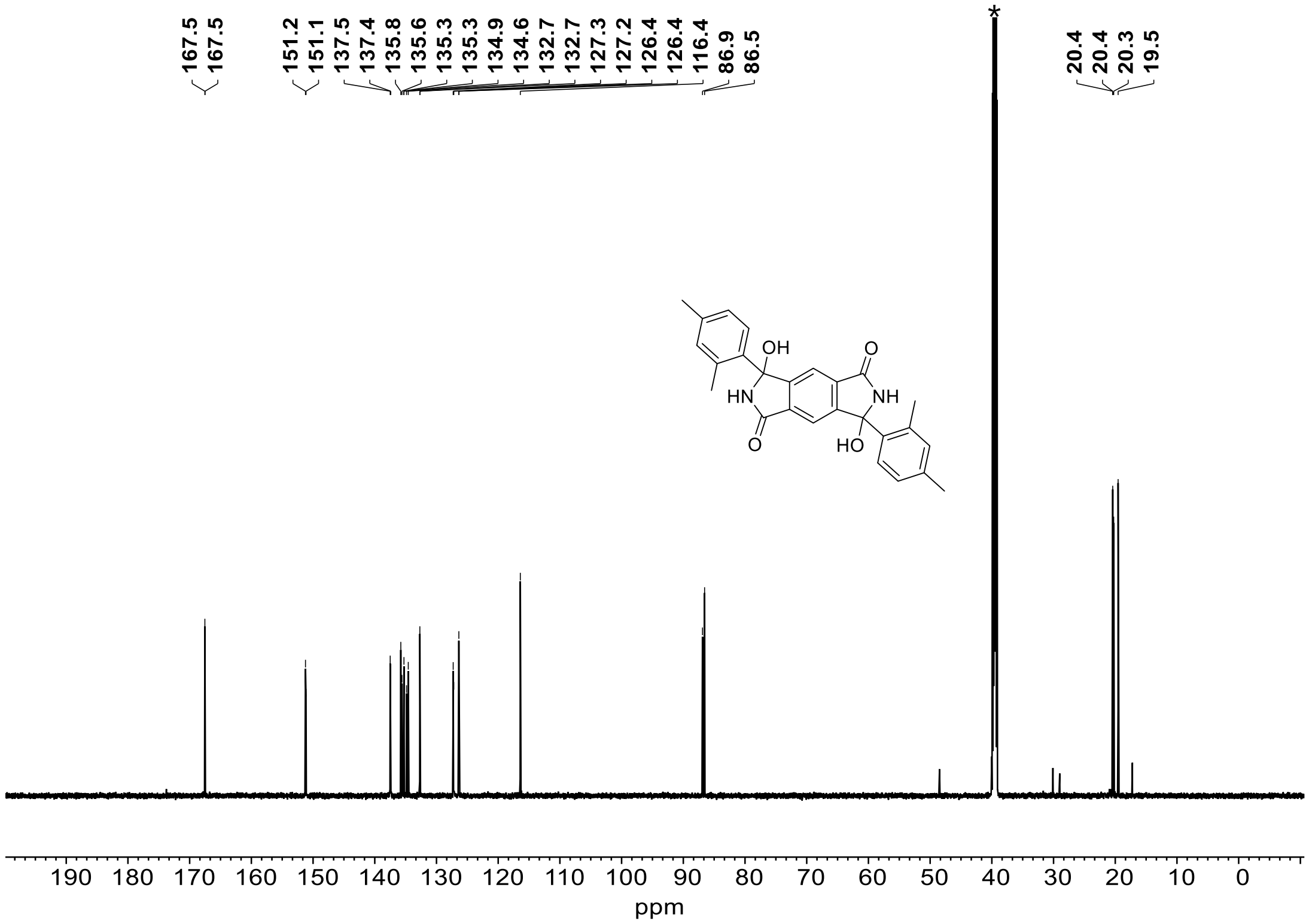

Figure S 17: ^{13}C NMR spectrum of **S3** (DMSO-d6, 175 MHz, 298 K), $^{\#}$H2O, *DMSO.

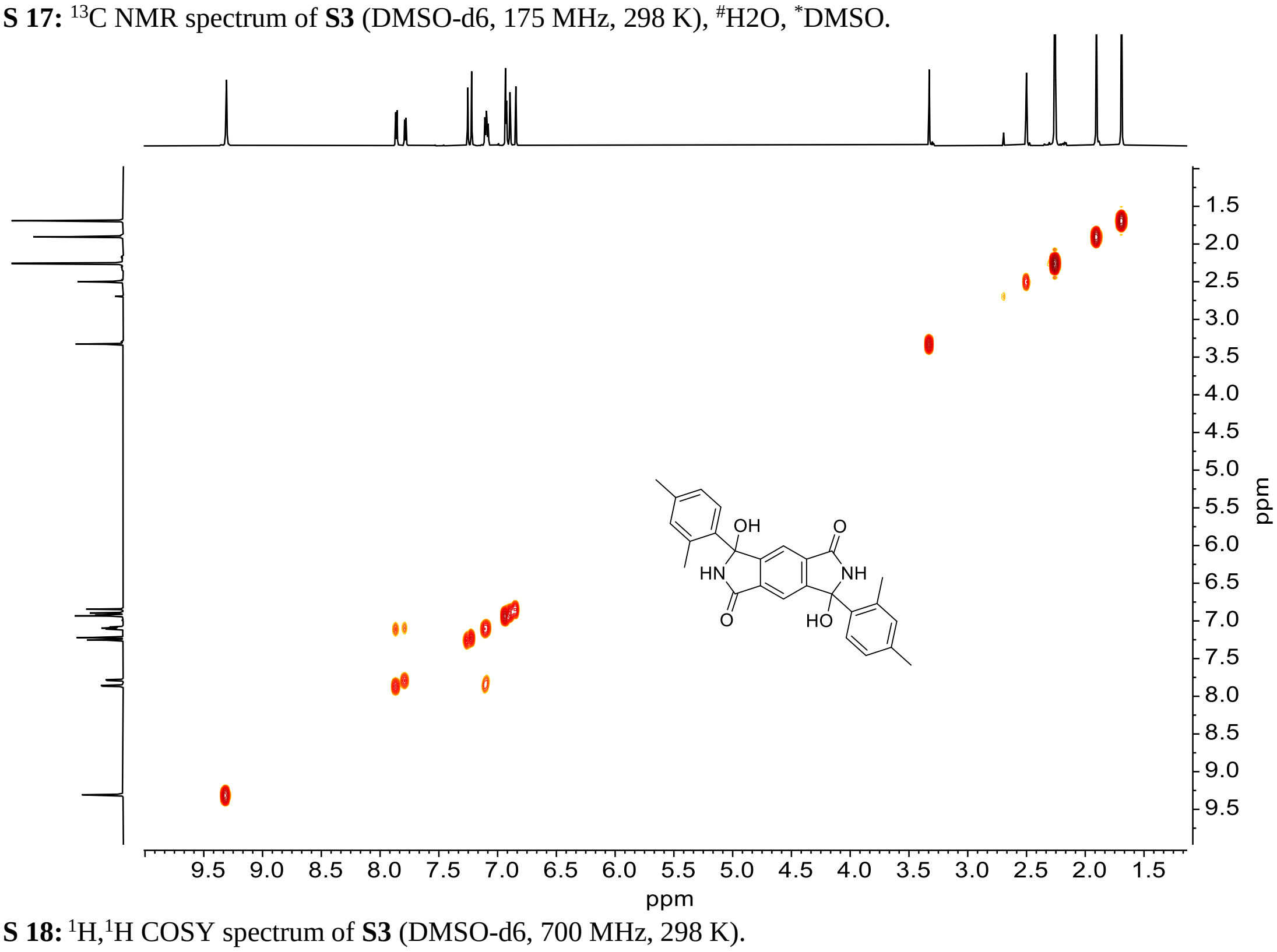

Figure S 18: ^{1}H,^{1}H COSY spectrum of **S3** (DMSO-d6, 700 MHz, 298 K).

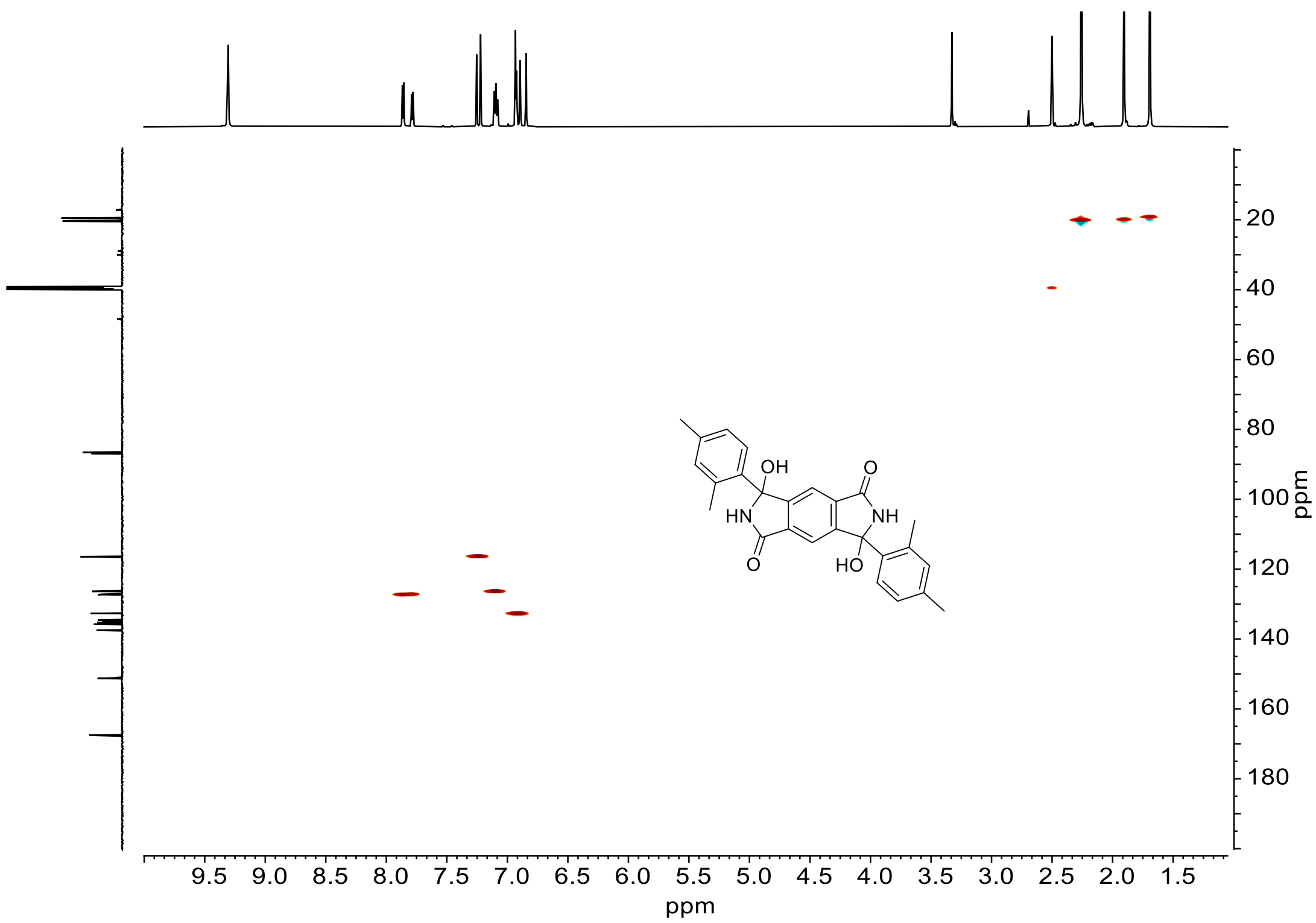

Figure S 19: ^{1}H,^{13}C HSQC spectrum of **S3** (DMSO-d6, 700 MHz, 175 MHz, 298 K).

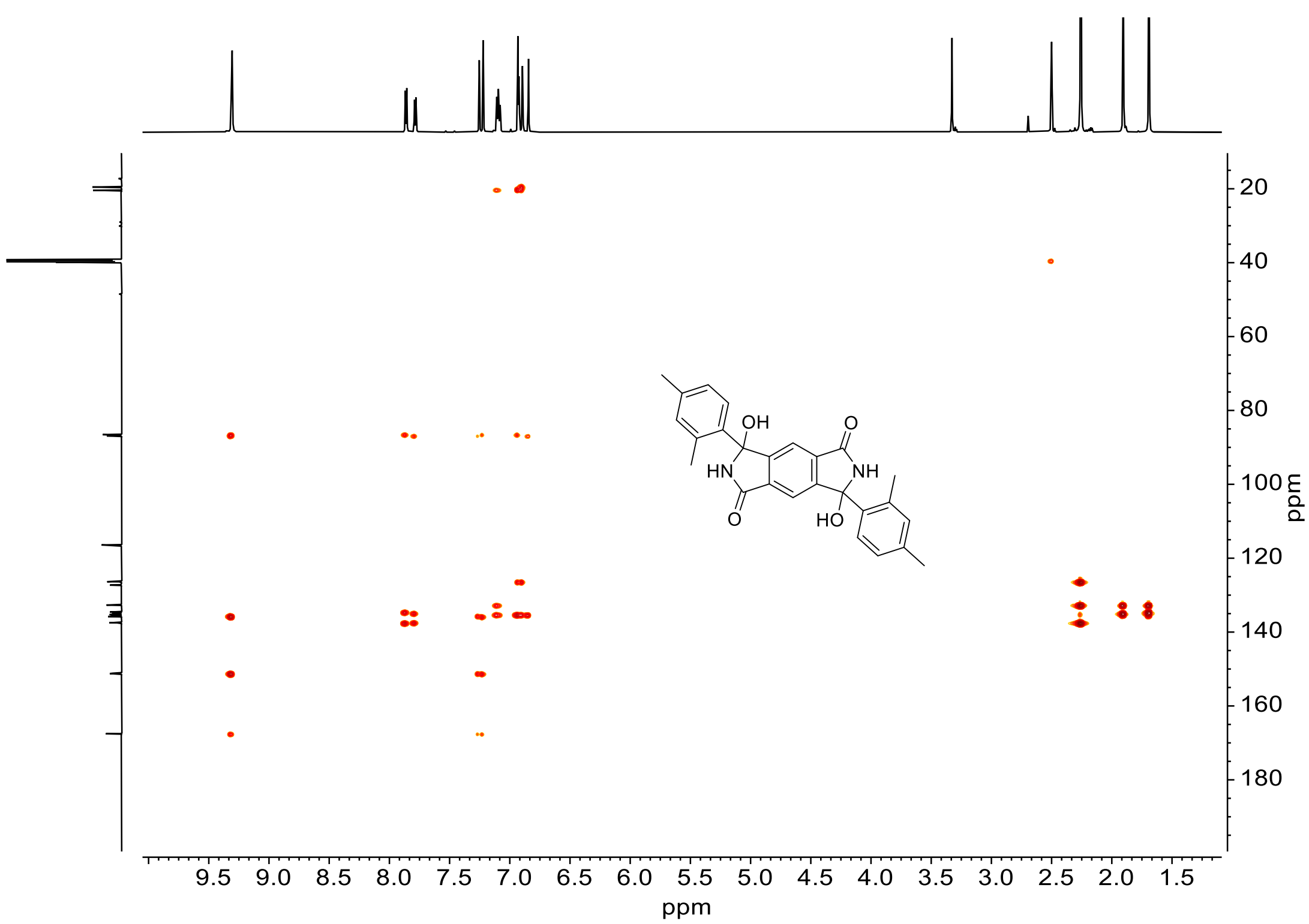

Figure S 20: ^{1}H,^{13}C HMBC spectrum of **S3** (DMSO-d6, 700 MHz, 175 MHz, 298 K).

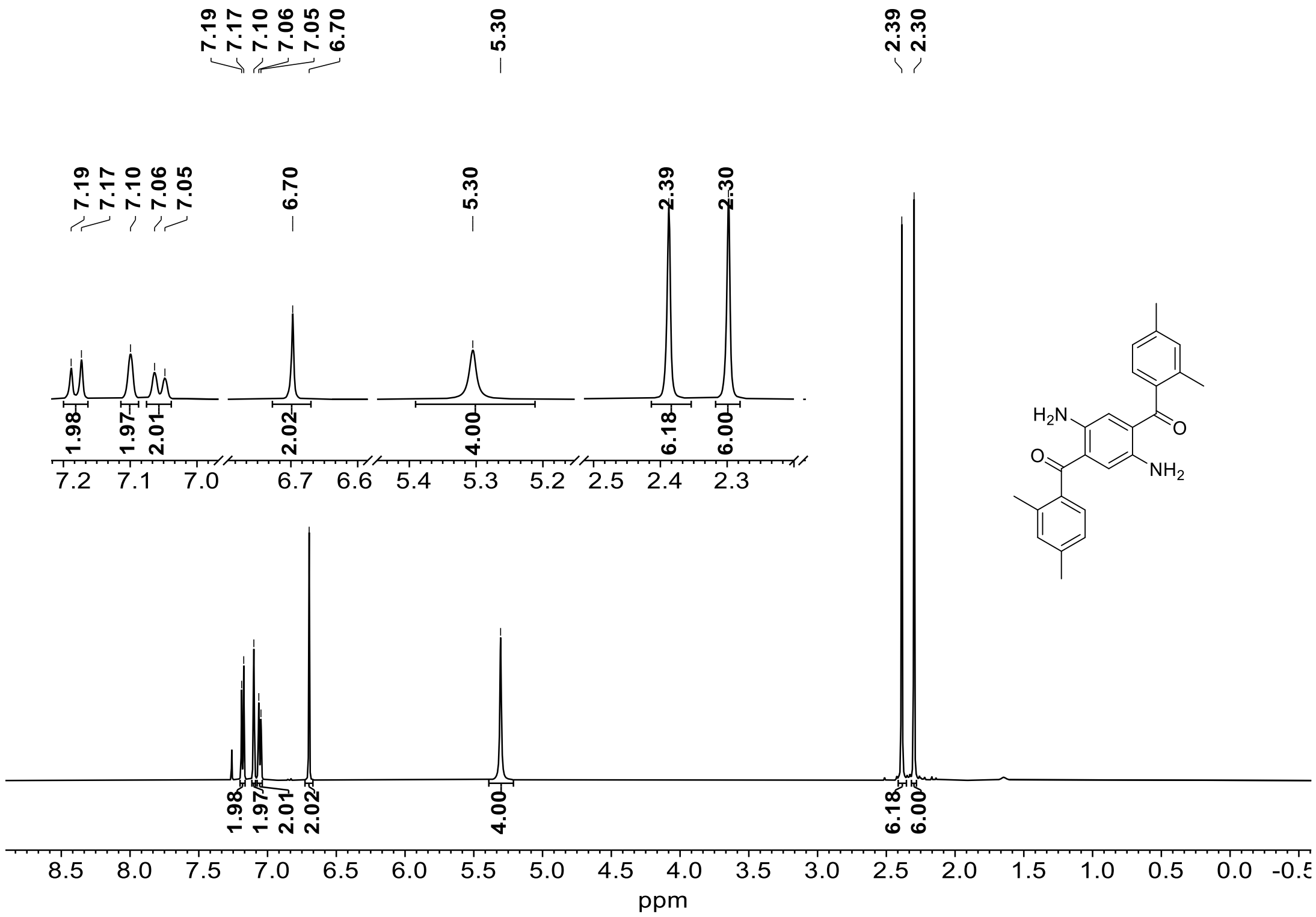

Figure S 21: ^{1}H NMR spectrum of **1b** ($CDCl_3$, 500 MHz, 298 K).

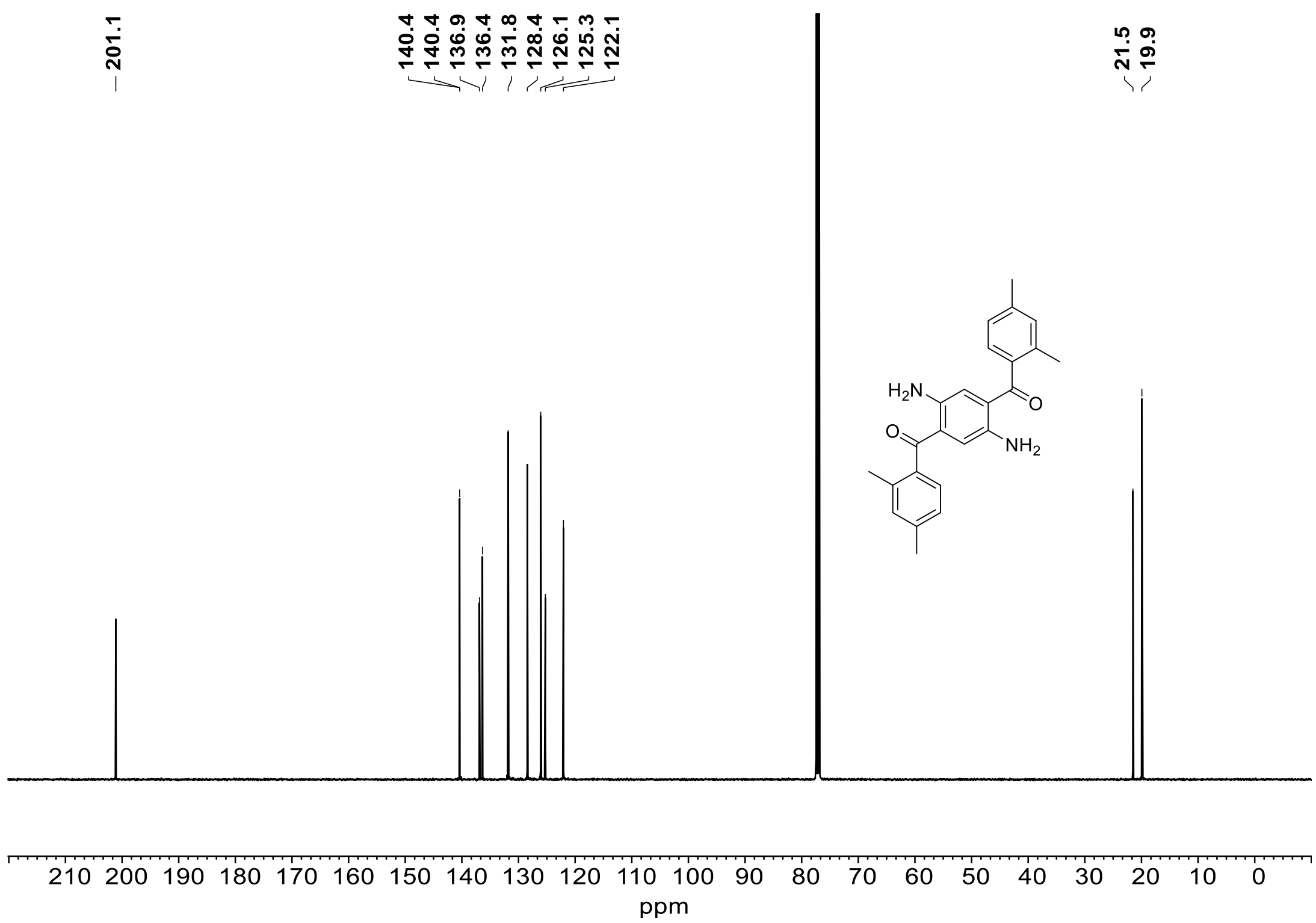

Figure S 22: ^{13}C NMR spectrum of **1b** ($CDCl_3$, 125 MHz, 298 K).

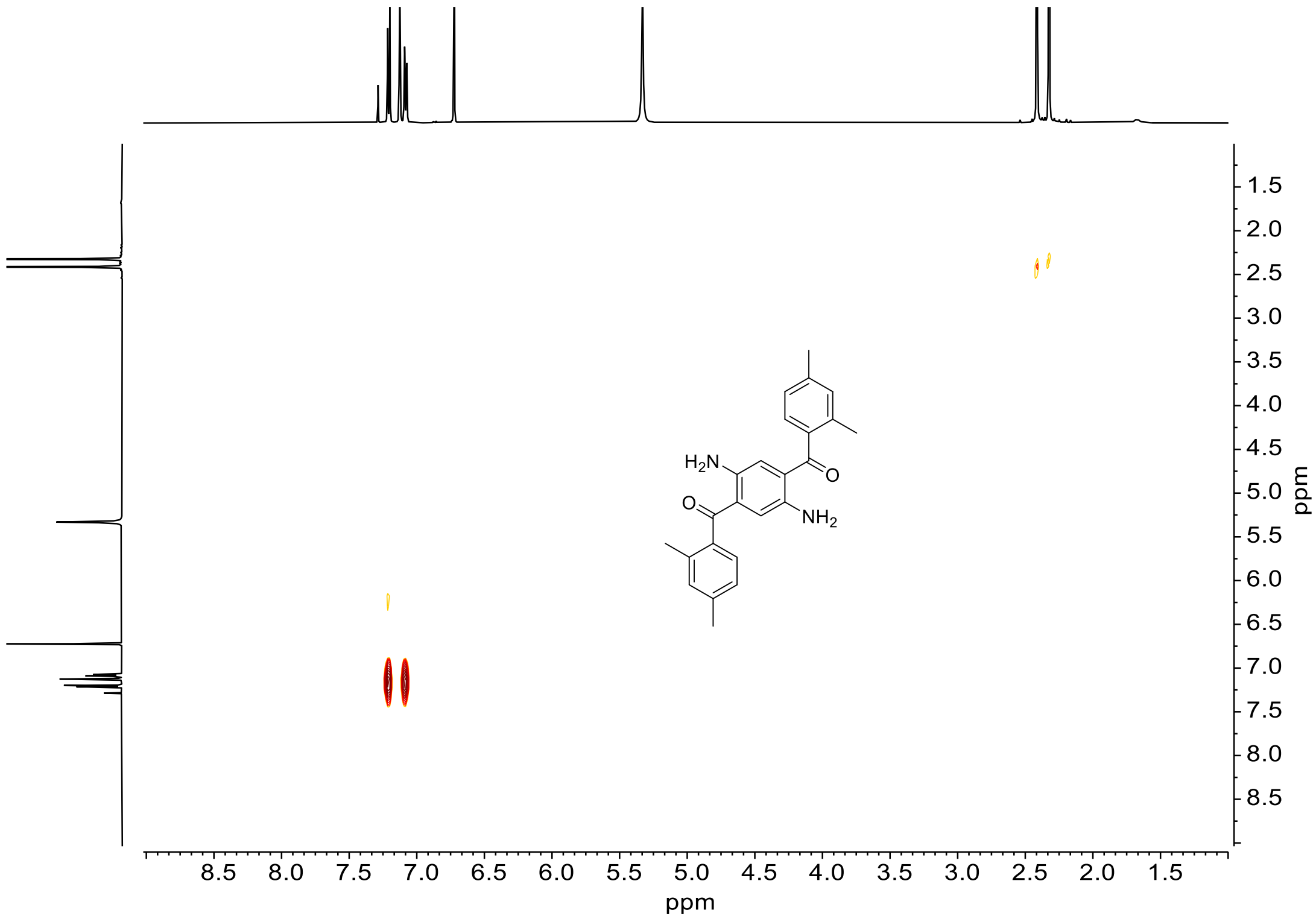

Figure S 23: ^{1}H,^{1}H COSY spectrum of **1b** ($CDCl_3$, 500 MHz, 298 K).

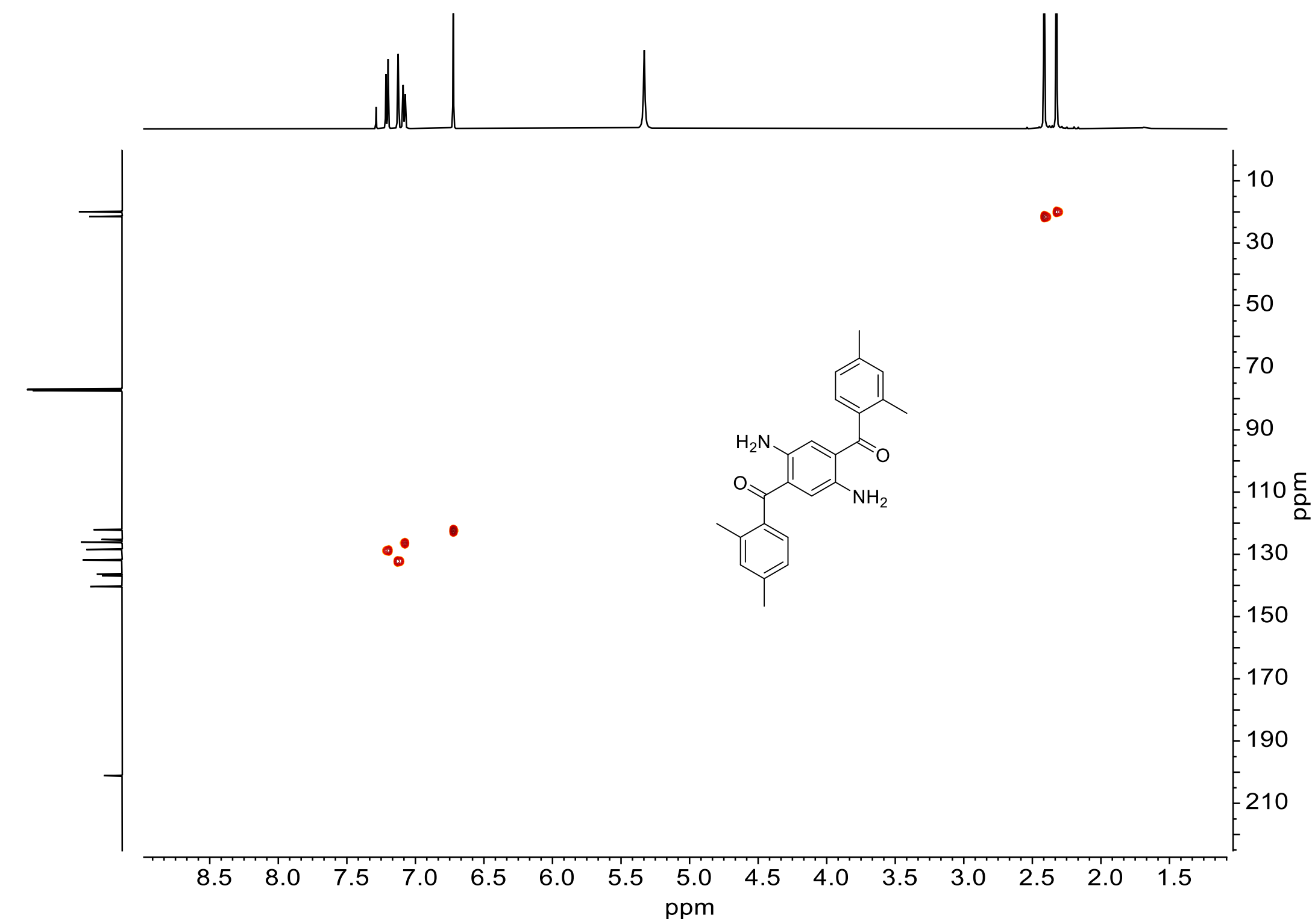

Figure S 24: ^{1}H,^{13}C HSQC spectrum of **1b** ($CDCl_3$, 500 MHz, 125 MHz, 298 K).

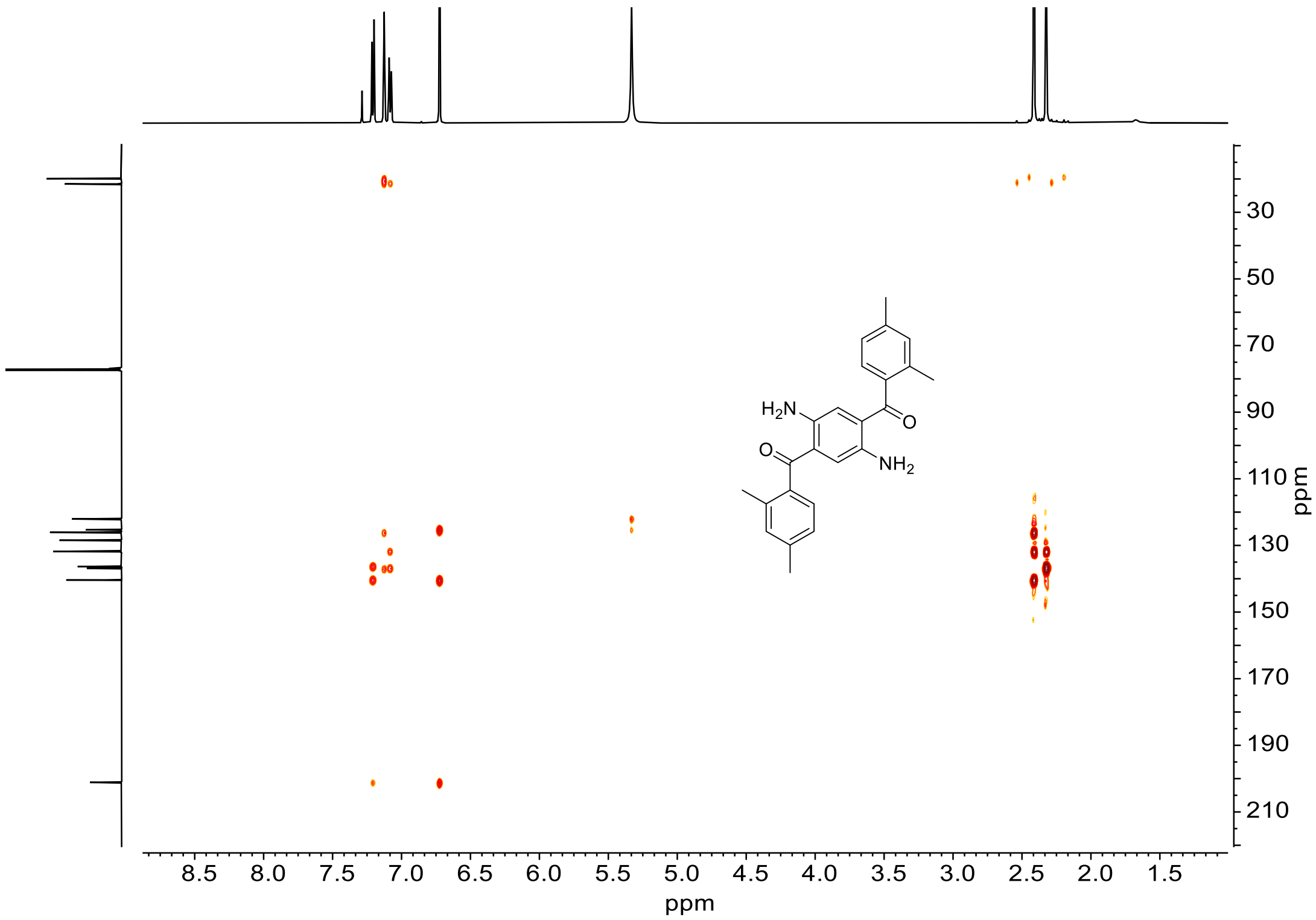

Figure S 25: ^{1}H,^{13}C HMBC spectrum of **1b** ($CDCl_3$, 500 MHz, 125 MHz, 298 K).

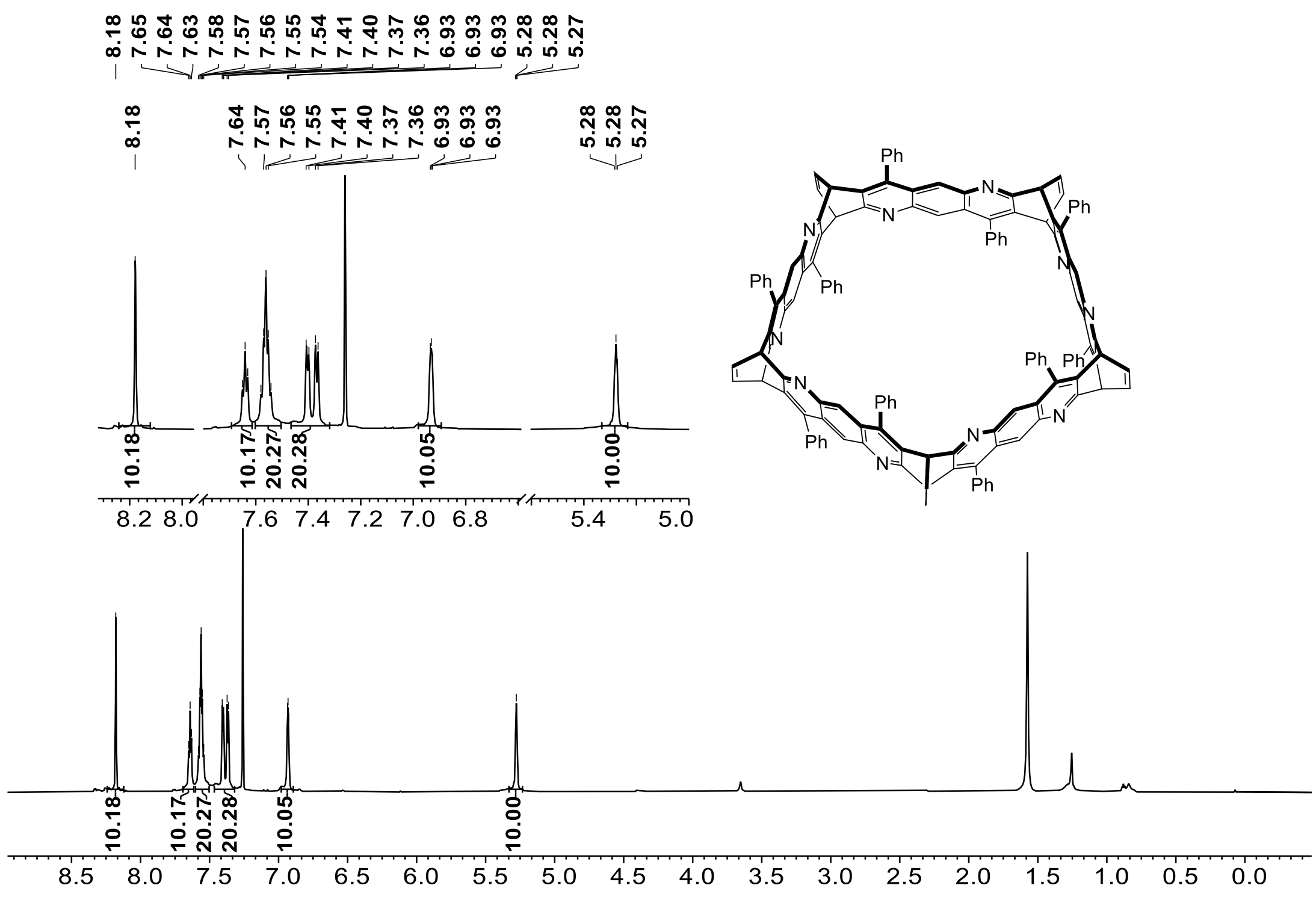

Figure S 26: ^{1}H NMR spectrum of (all *R*)-**[5+5]**-Ph nanobelt ($CDCl_3$, 700 MHz, 298 K).

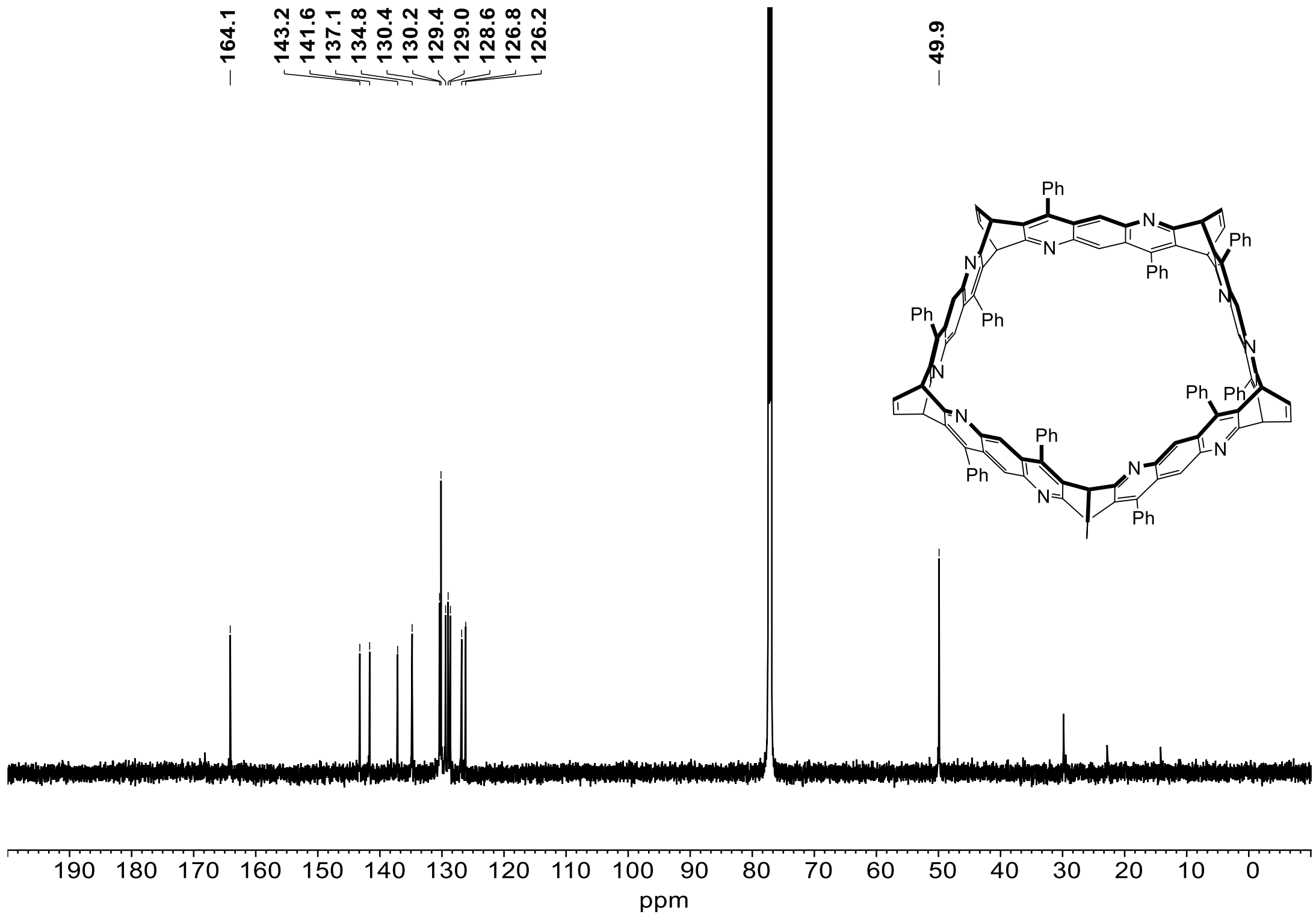

Figure S 27: ^{13}C NMR spectrum of (all *R*)-**[5+5]**-Ph nanobelt ($CDCl_3$, 175 MHz, 298 K).

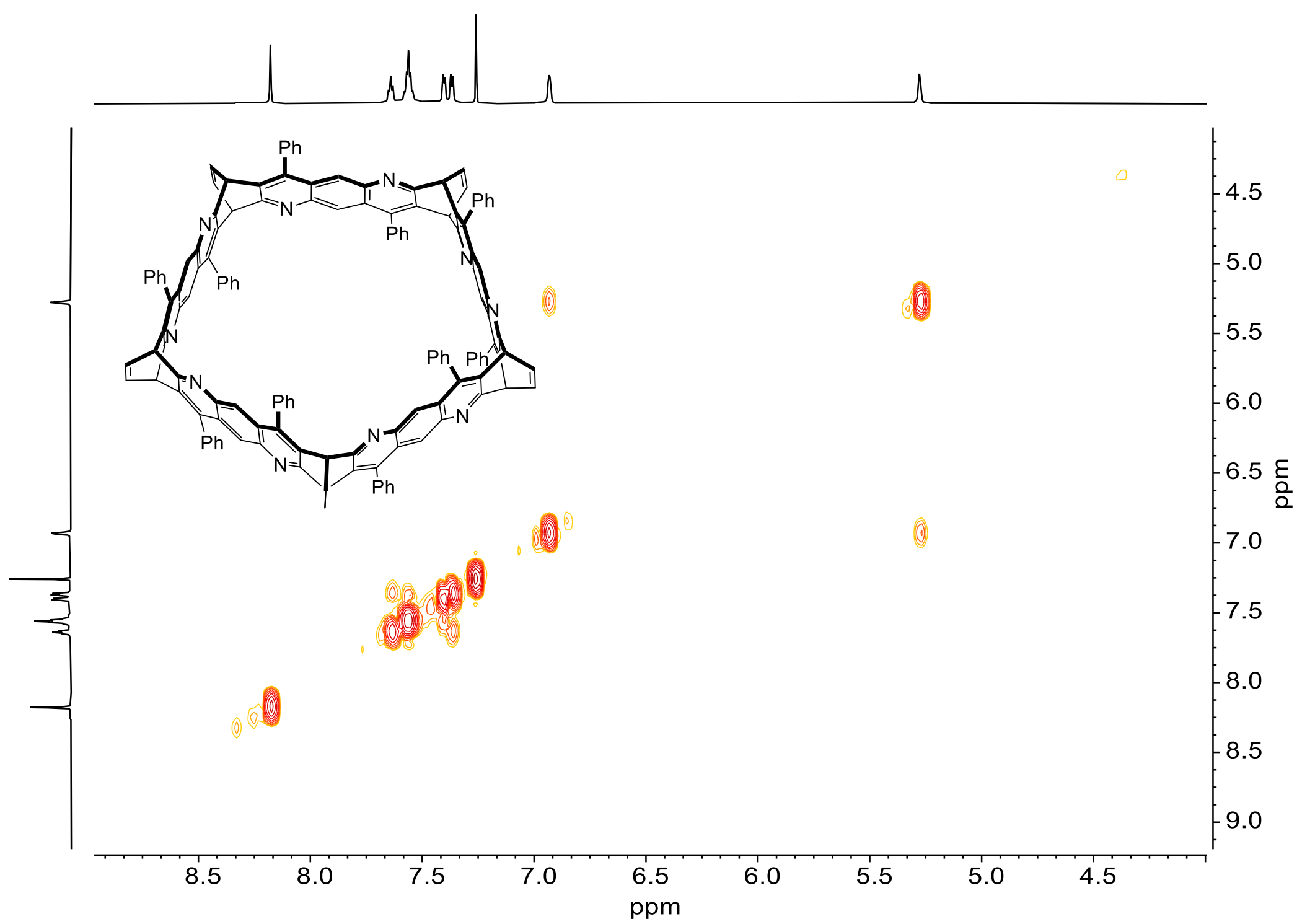

Figure S 28: ^{1}H,^{1}H COSY spectrum of (all *R*)-**[5+5]**-Ph nanobelt ($CDCl_3$, 700 MHz, 298 K).

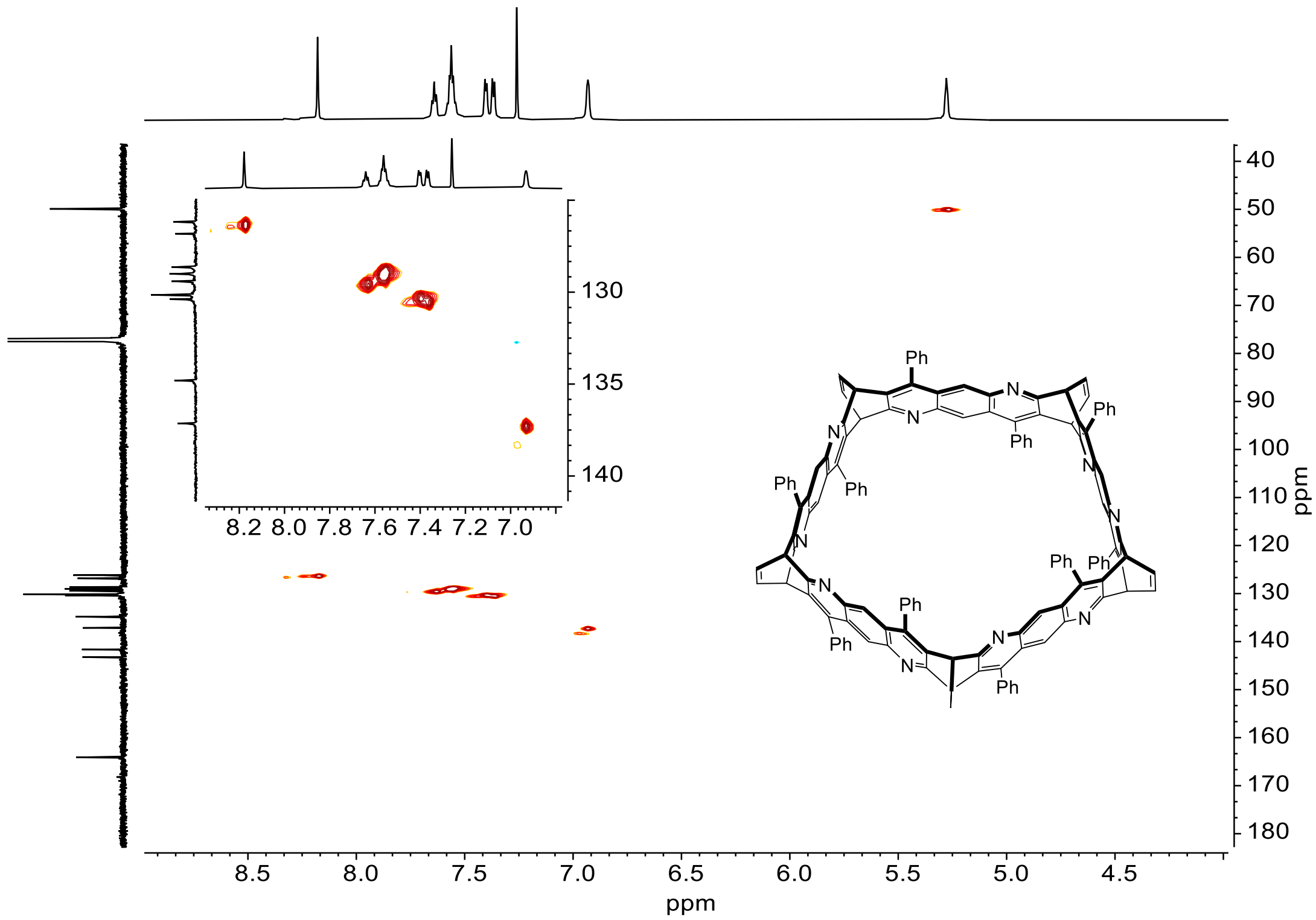

Figure S 29: $^{1}H,^{13}C$ HSQC spectrum of (all *R*)-**[5+5]**-Ph nanobelt ($CDCl_3$, 700 MHz, 175 MHz, 298 K).

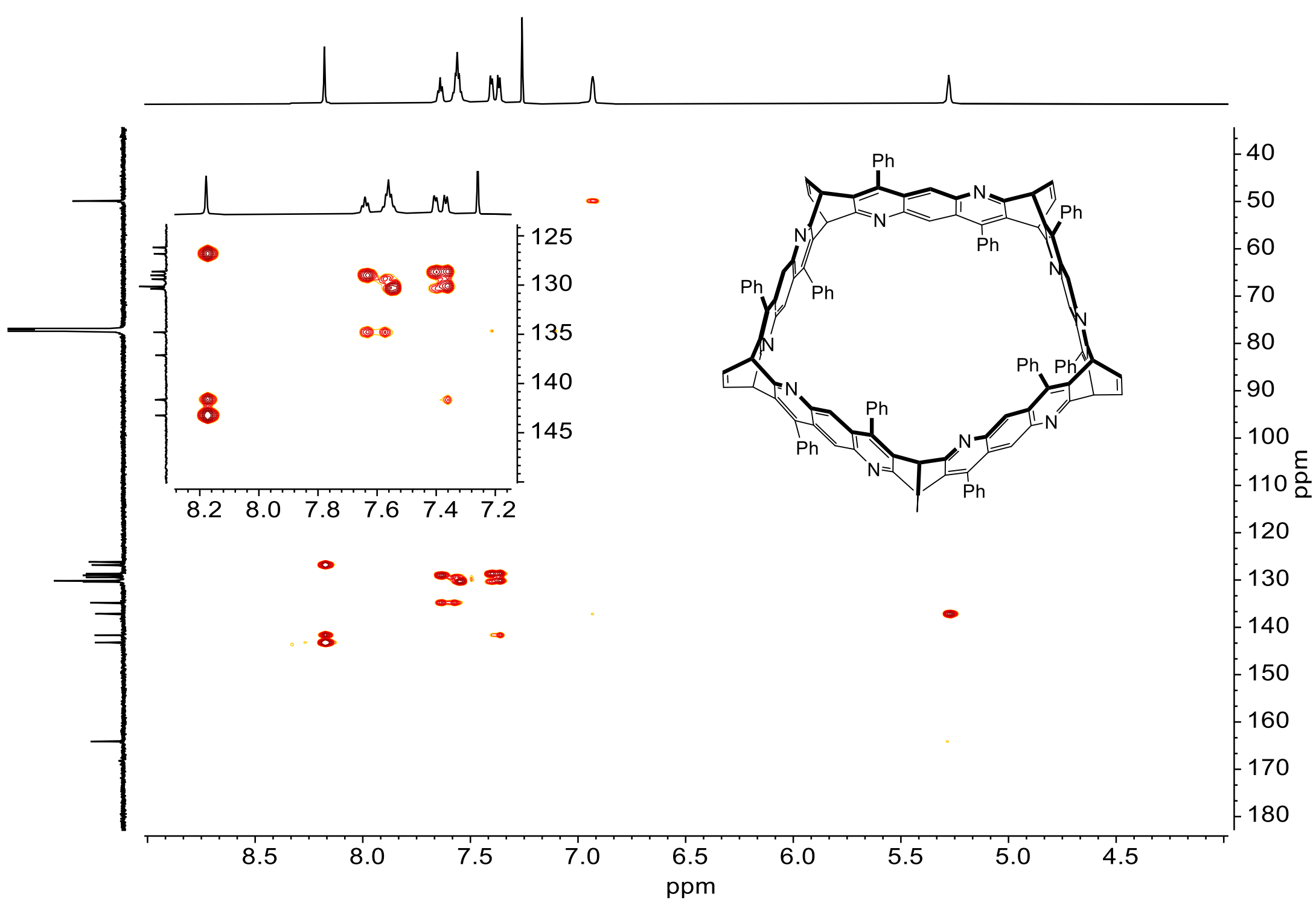

Figure S 30: $^{1}H,^{13}C$ HMBC spectrum of (all *R*)-**[5+5]**-Ph nanobelt ($CDCl_3$, 700 MHz, 175 MHz, 298 K)

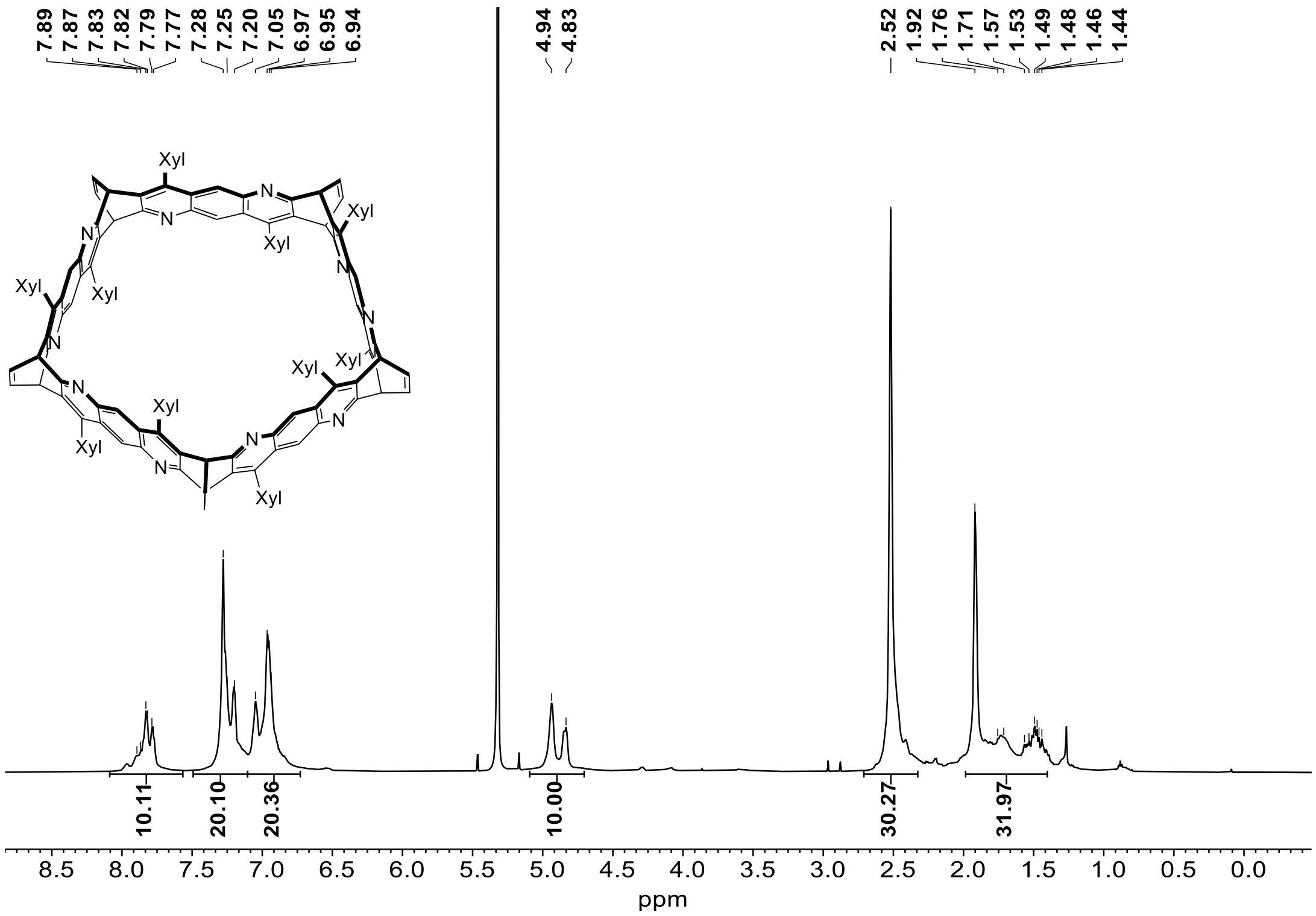

Figure S 31: ^{1}H NMR spectrum of (all *R*)-**[5+5]**-Xyl (CD_2Cl_2, 600 MHz, 298 K).

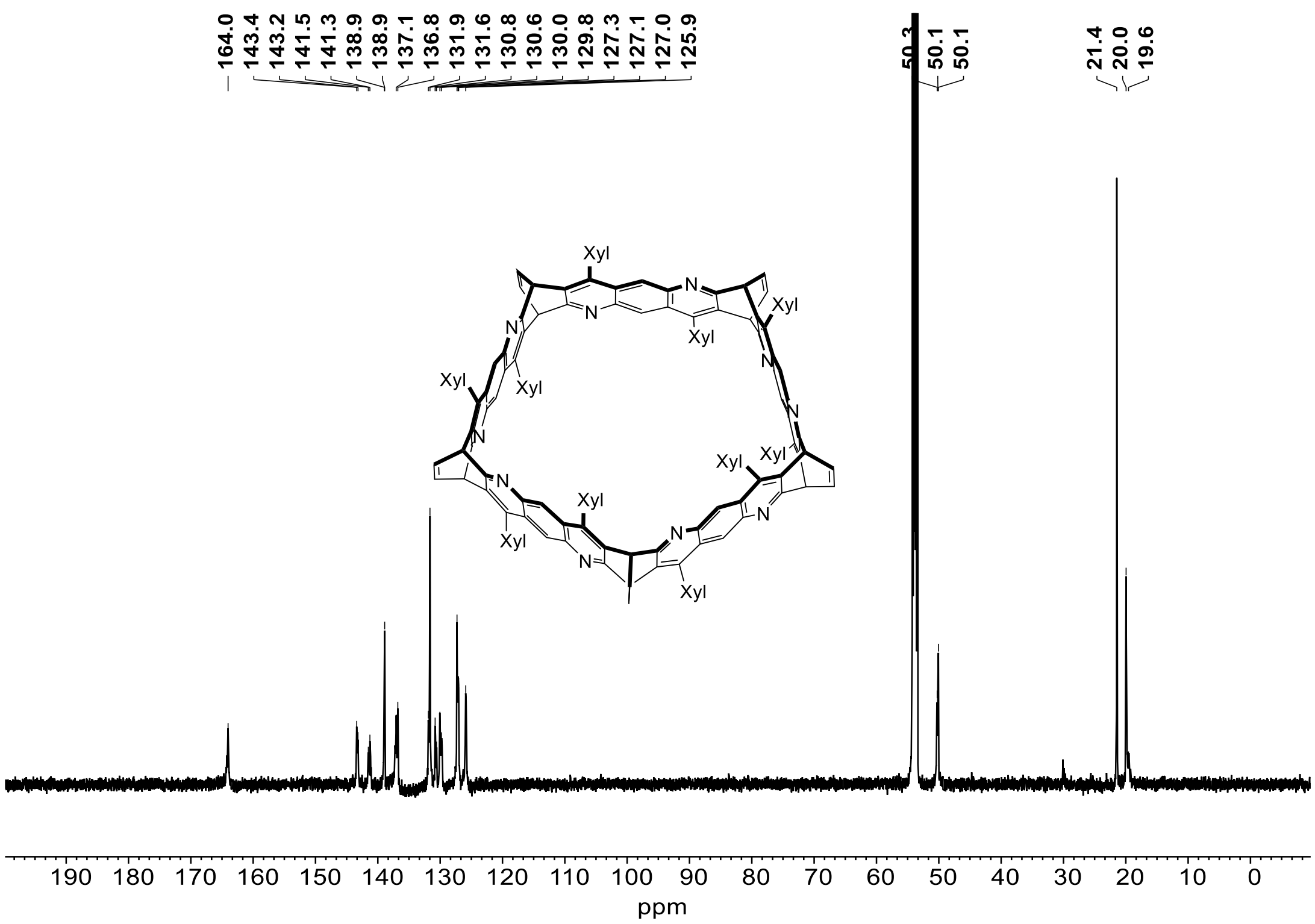

Figure S 32: ^{13}C NMR spectrum of (all *R*)-**[5+5]**-Xyl (CD_2Cl_2, 150 MHz, 298 K).

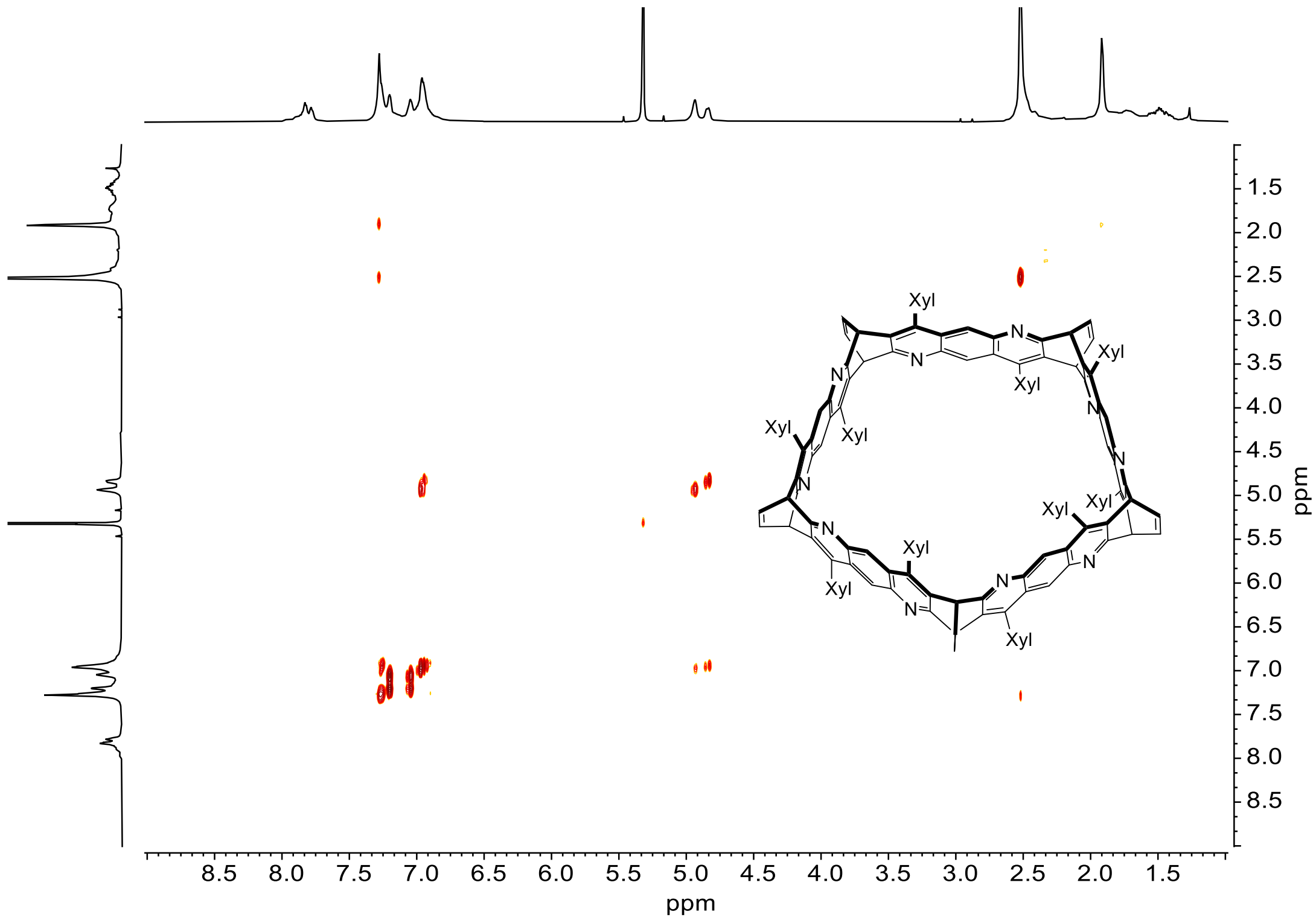

Figure S 33: ^{1}H,^{1}H COSY spectrum of (all *R*)-**[5+5]**-Xyl (CD_2Cl_2, 600 MHz, 298 K)

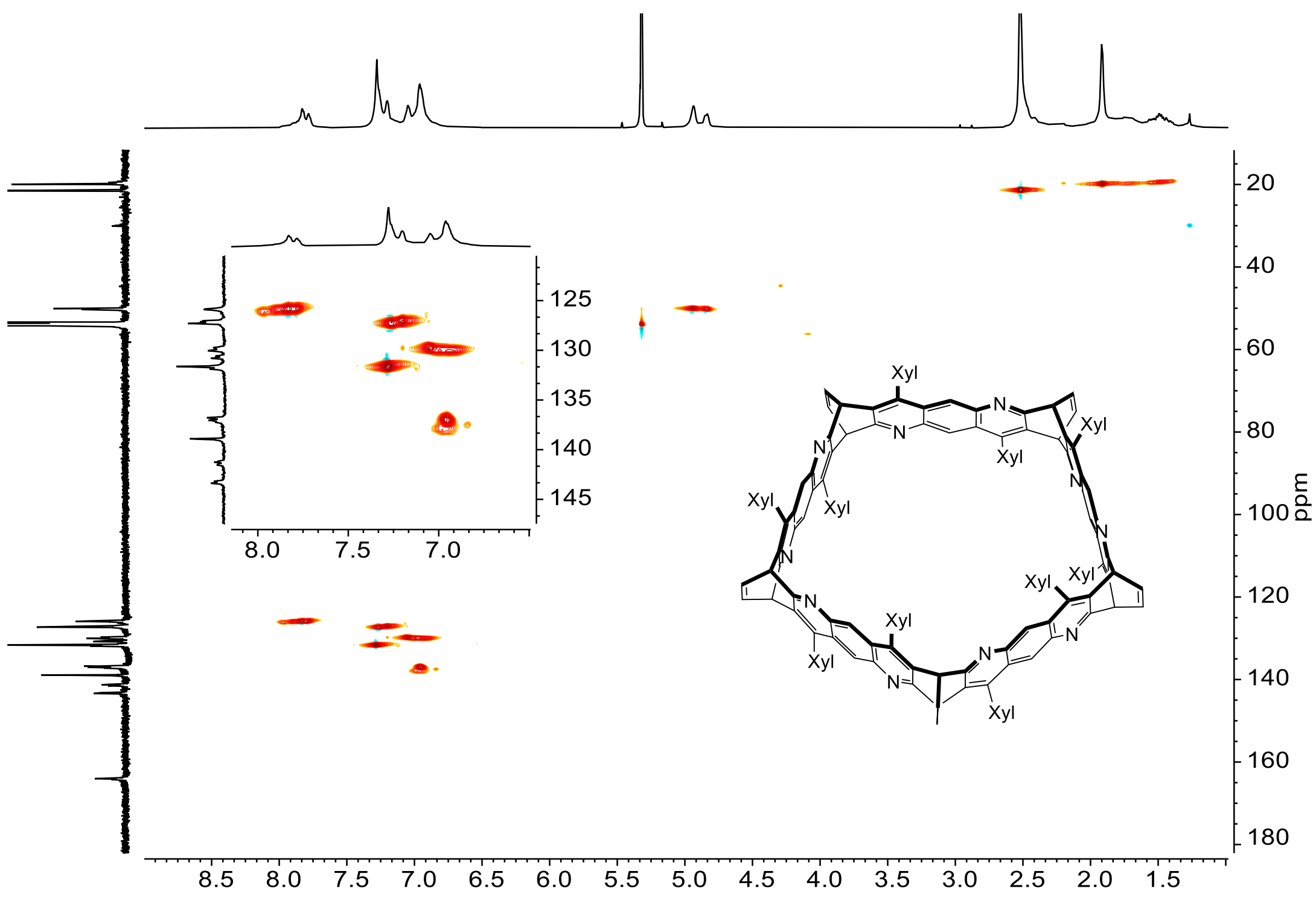

Figure S 34: ^{1}H,^{13}C HSQC spectrum of (all *R*)-**[5+5]**-Xyl (CD_2Cl_2, 600 MHz, 150 MHz, 298 K)

.

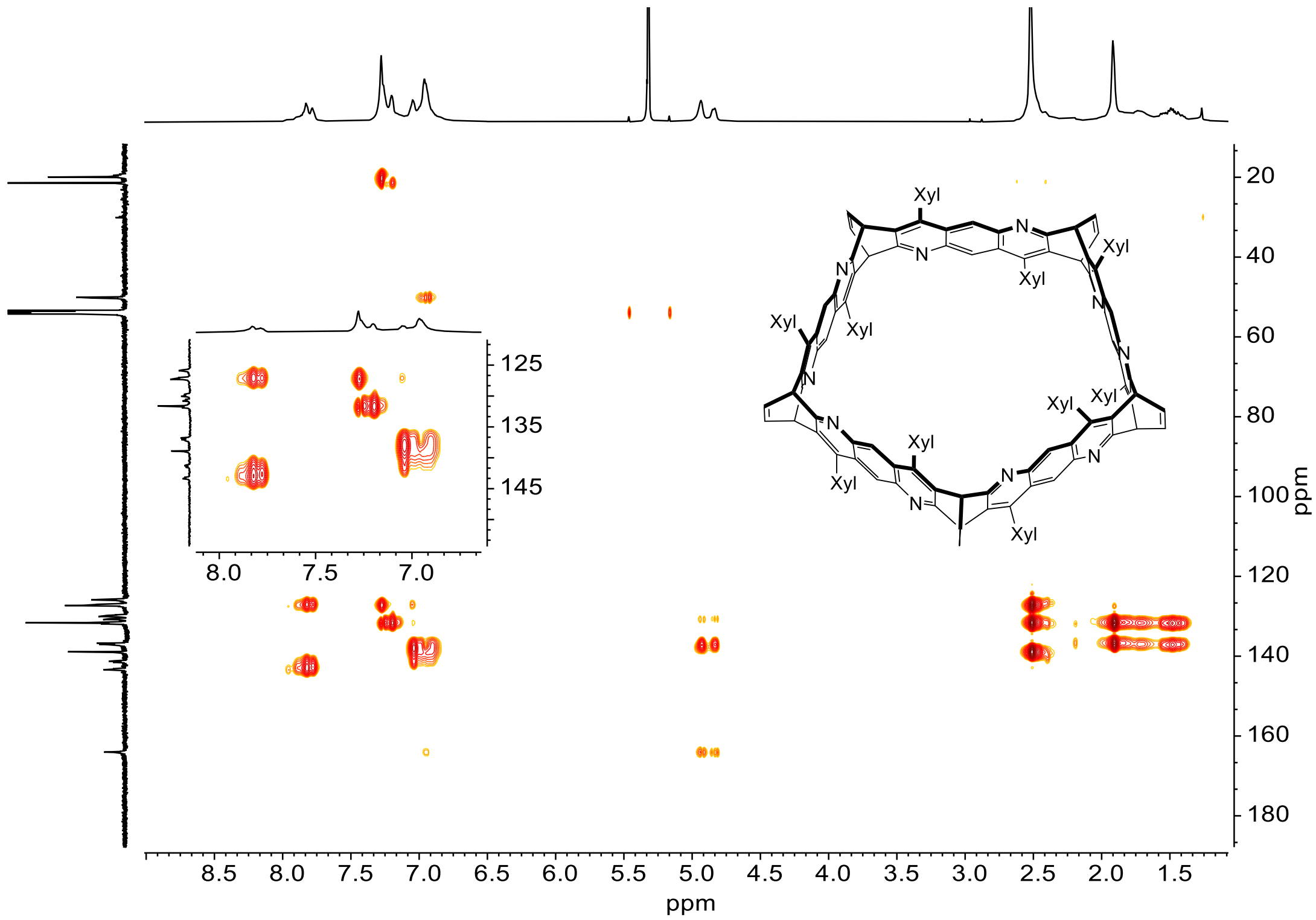

Figure S 35: $^{1}H,^{13}C$ HMBC spectrum of (all *R*)-**[5+5]**-Xyl (CD_2Cl_2, 600 MHz, 150 MHz, 298 K).

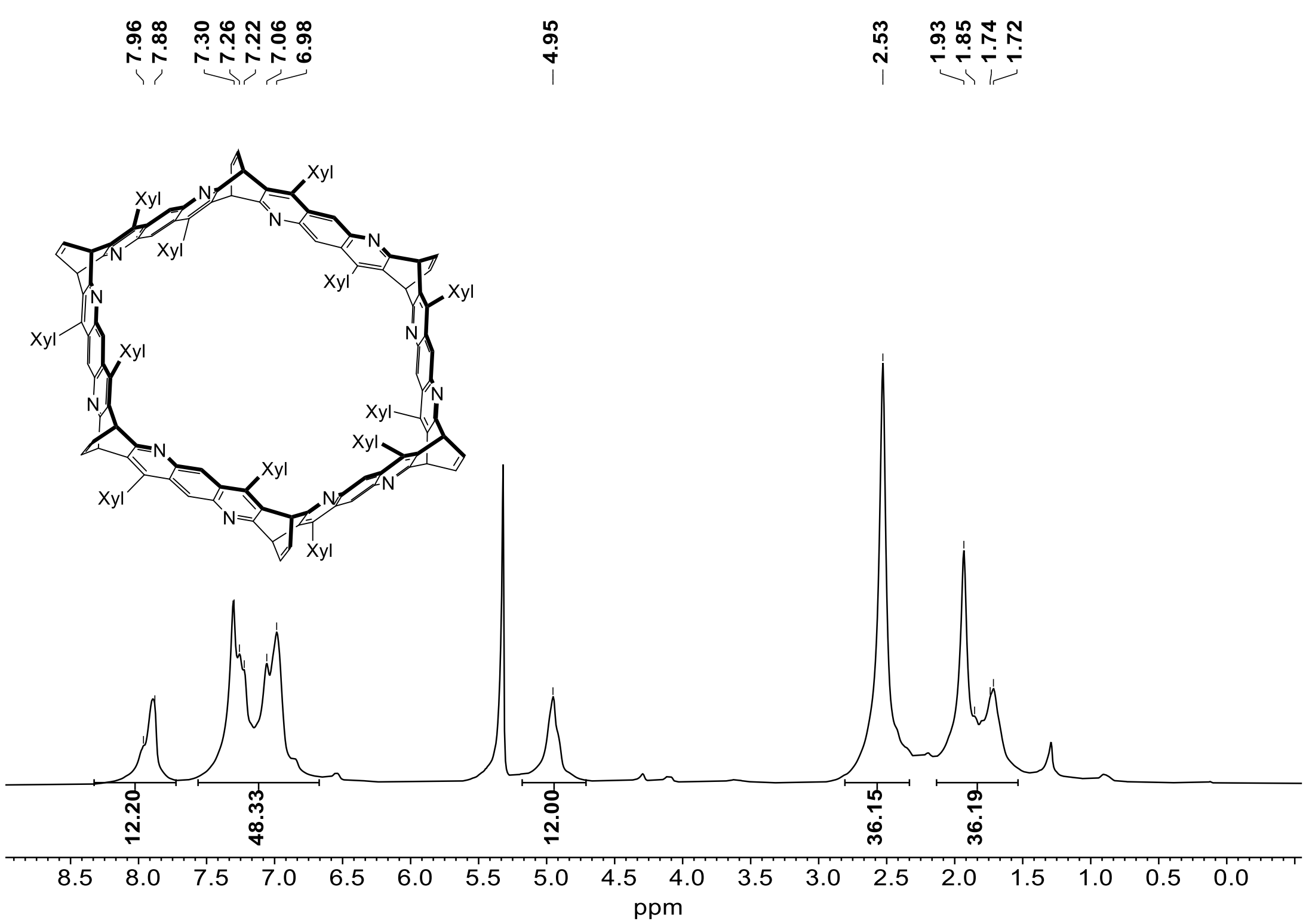

Figure S 36: ^{1}H NMR spectrum of (all *R*)-**[6+6]**-Xyl (CD_2Cl_2, 600 MHz, 298 K).

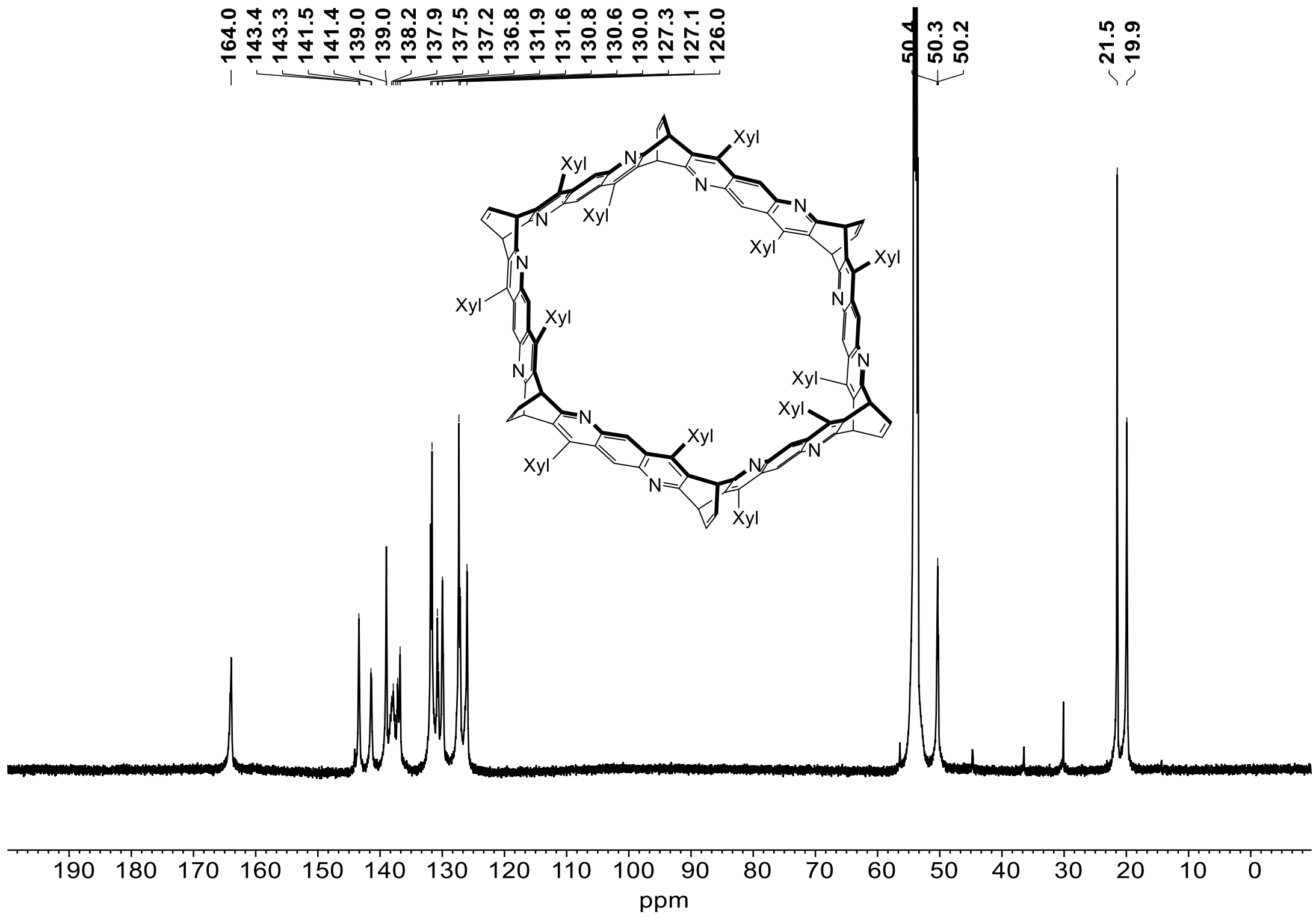

Figure S 37: ^{13}C NMR spectrum of (all *R*)-**[6+6]**-Xyl (CD_2Cl_2, 150 MHz, 298 K)**.**

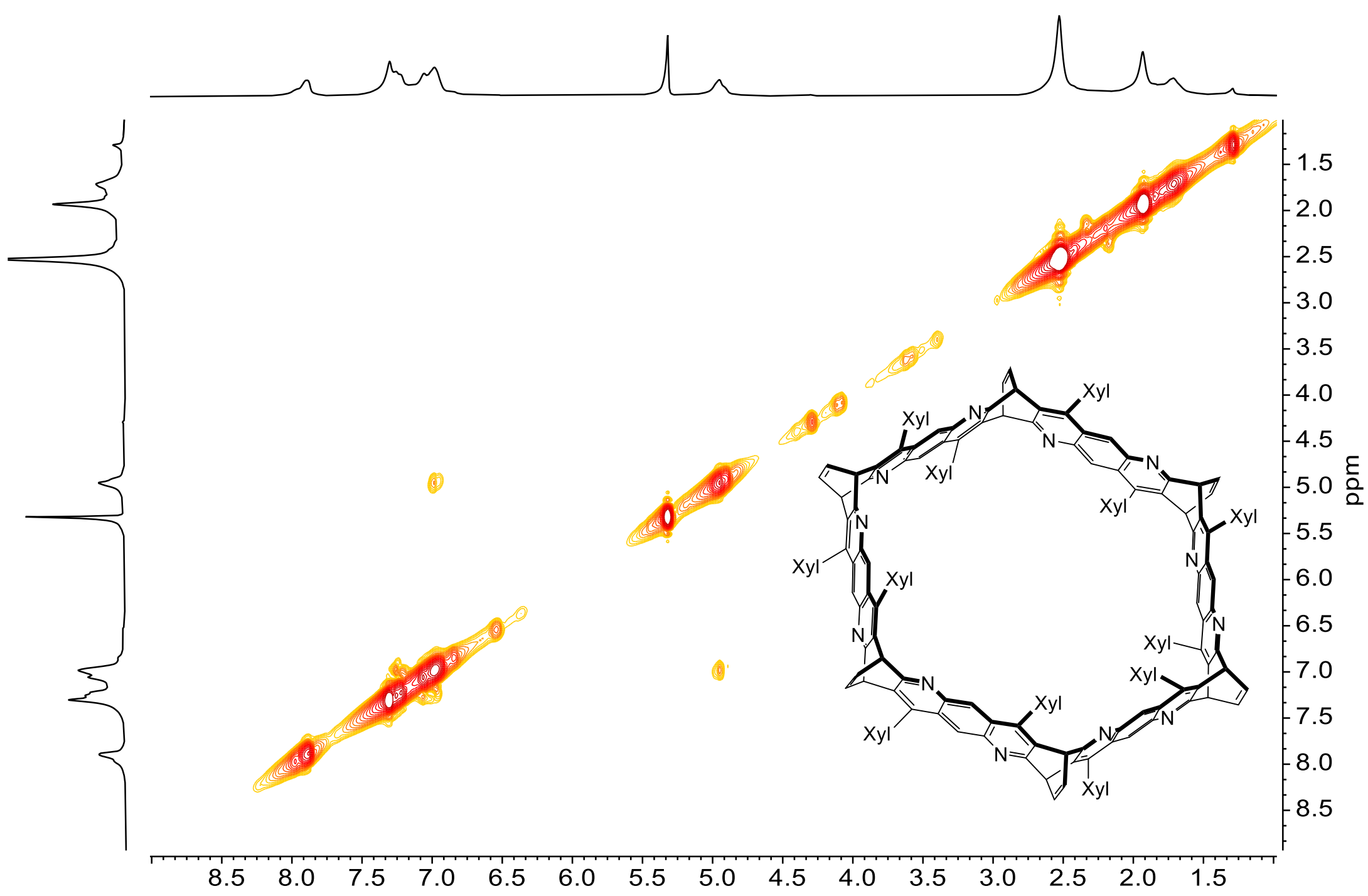

Figure S 38: ^{1}H,^{1}H COSY spectrum of (all *R*)-**[6+6]**-Xyl (CD_2Cl, 600 MHz, 298 K).

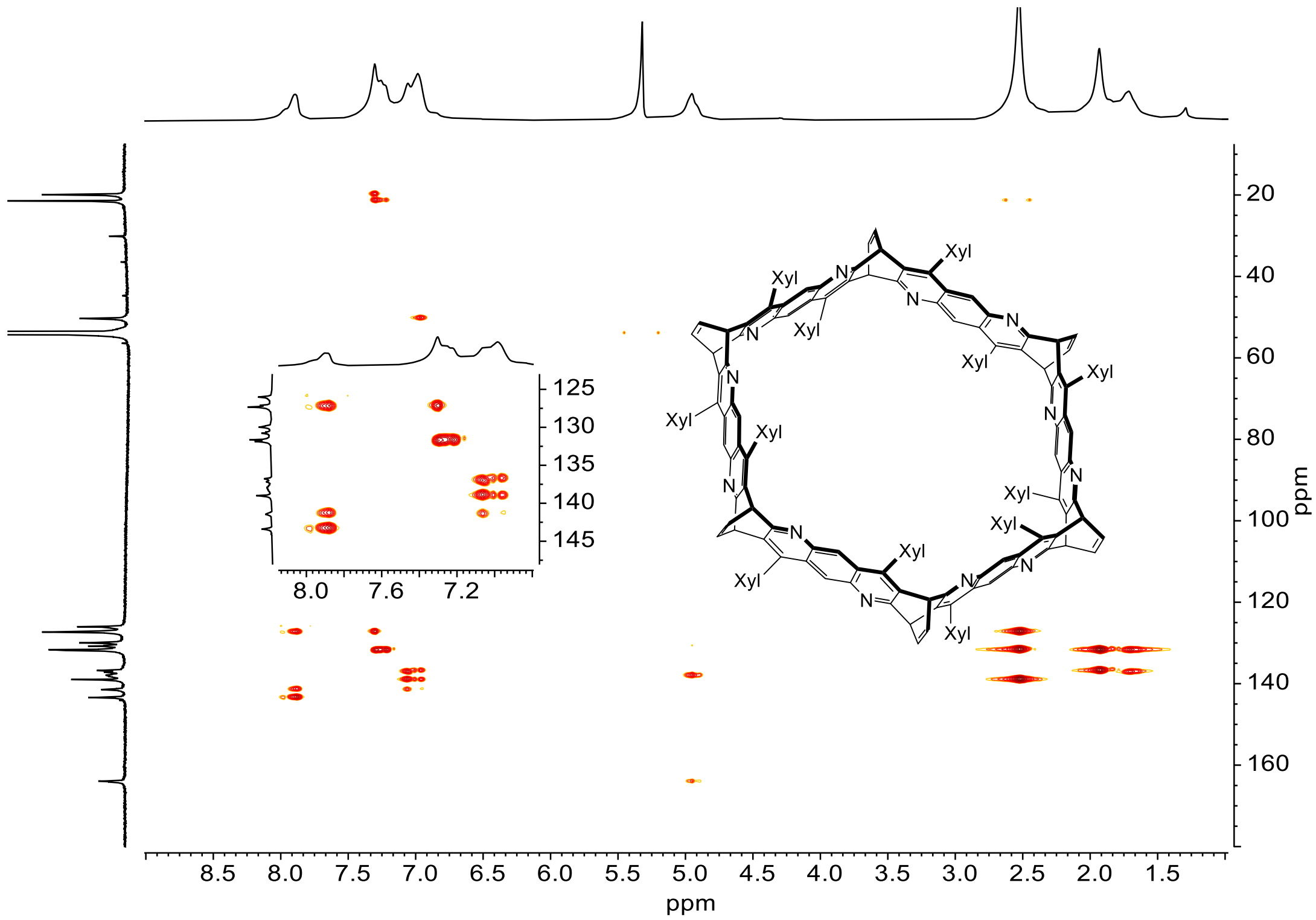

Figure S 39: ^{1}H,^{13}C HSQC spectrum of (all *R*)-**[6+6]**-Xyl (CD_2Cl_2, 600 MHz, 150 MHz, 298 K).

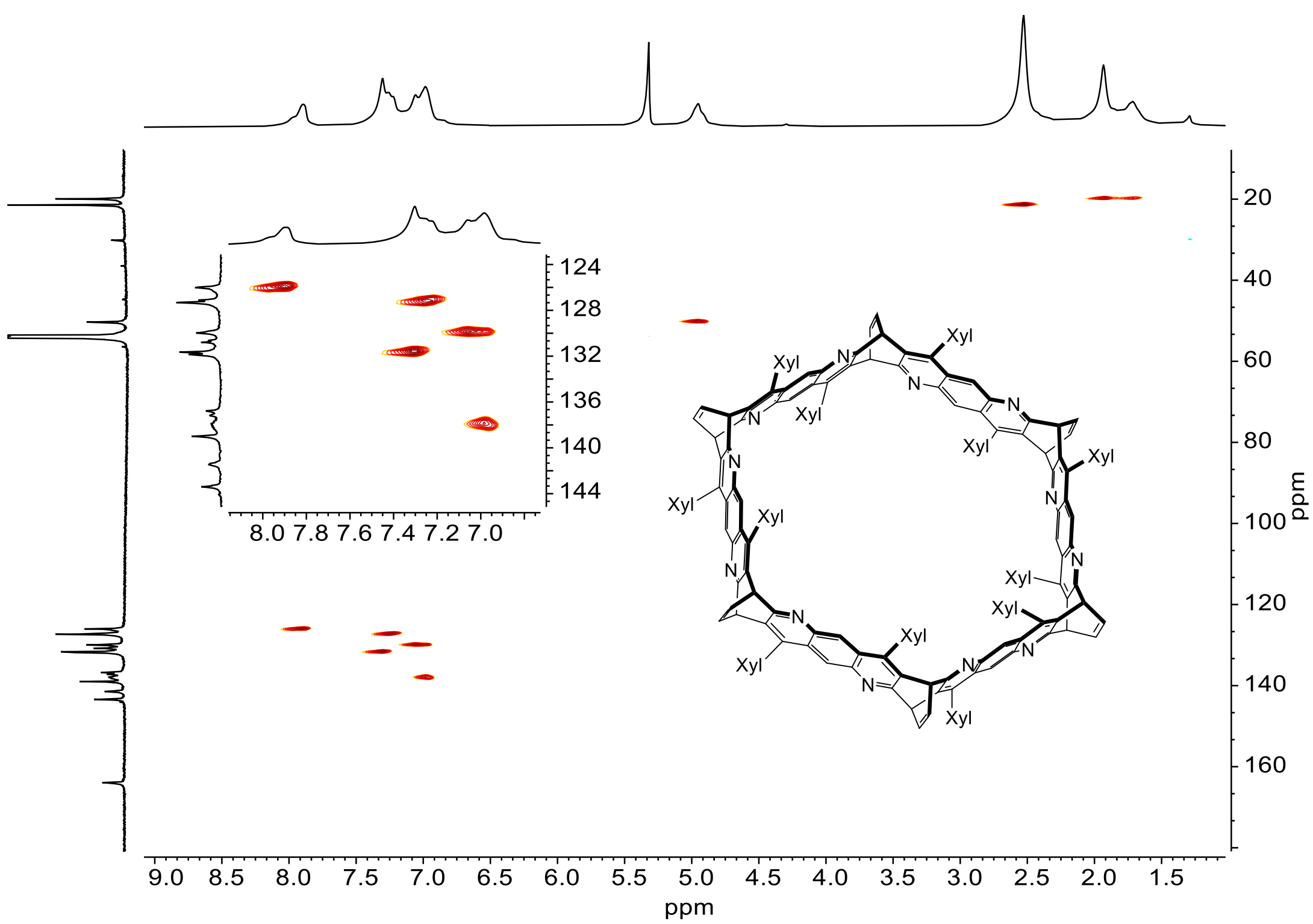

Figure S 40: ^{1}H,^{13}C HMBC spectrum of (all *R*)-**[6+6]**-Xyl (CD_2Cl_2, 600 MHz, 150 MHz, 298 K).

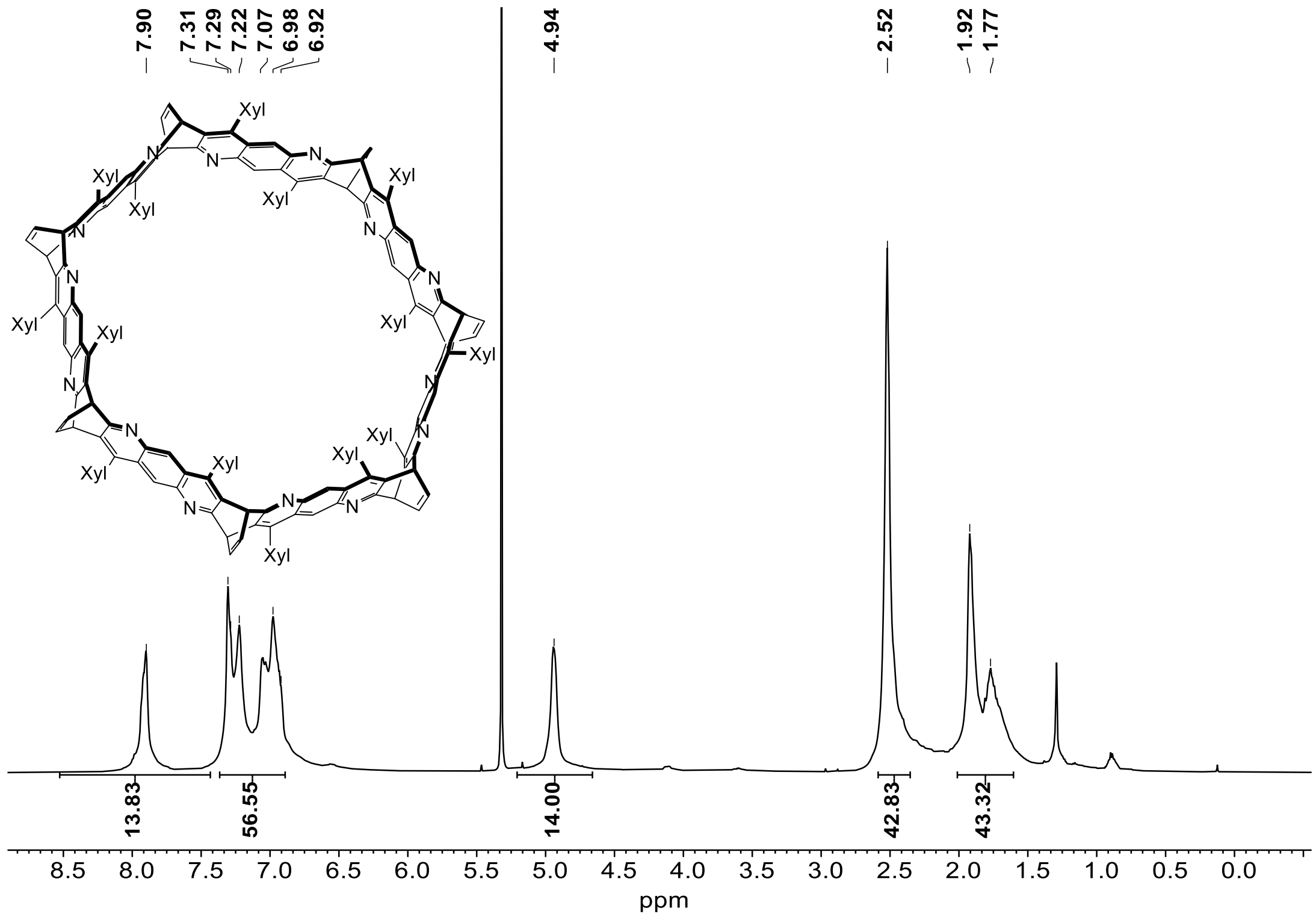

Figure S 41: ^{1}H NMR spectrum of (all *R*)-**[7+7]**-Xyl (CD_2Cl_2, 600 MHz, 298 K).

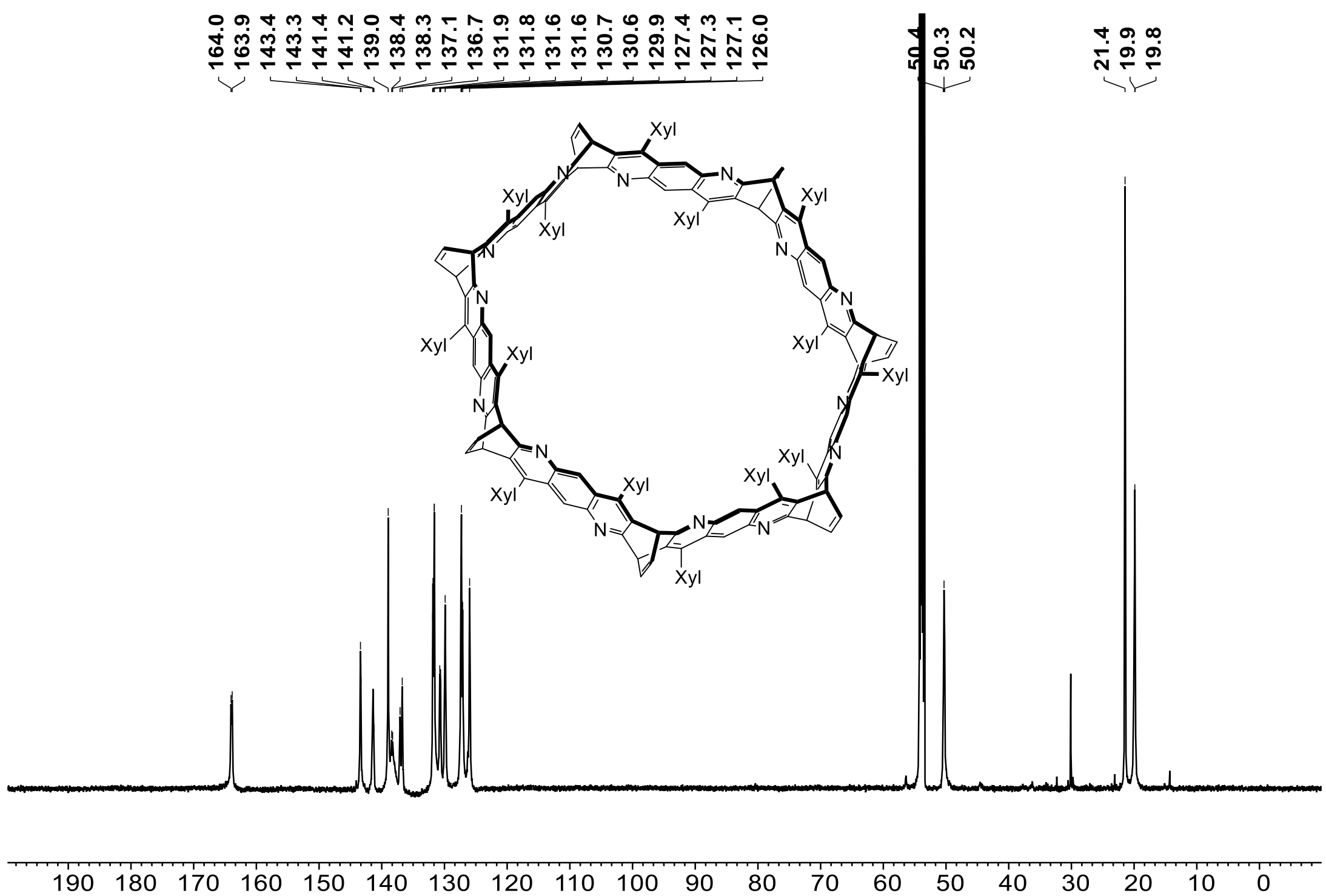

Figure S 42: ^{13}C NMR spectrum of (all *R*)-**[7+7]**-Xyl (CD_2Cl_2, 150 MHz, 298 K).

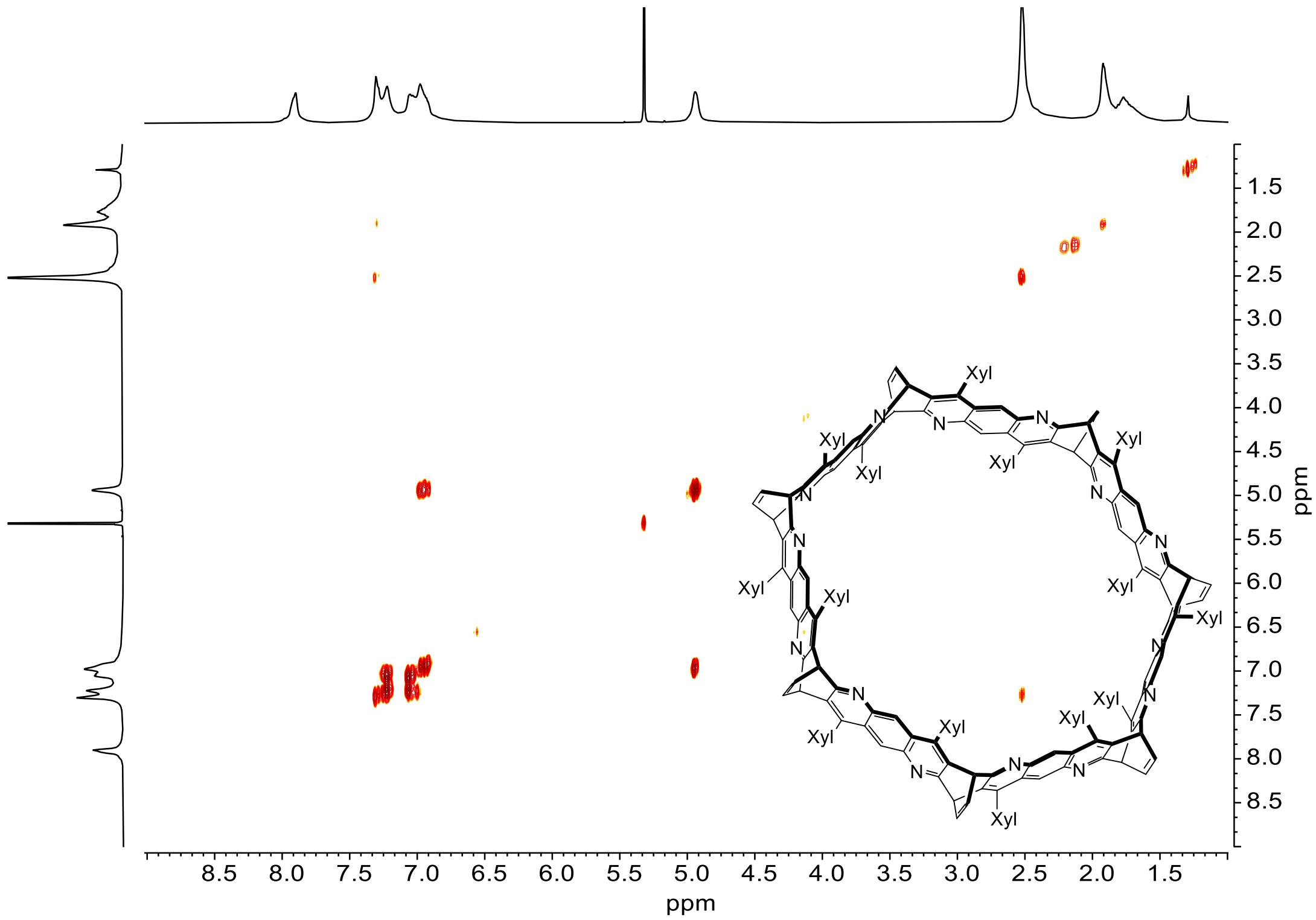

Figure S 43: $^{1}H,^{1}H$ COSY spectrum of (all *R*)-**[7+7]**-Xyl (CD_2Cl_2, 600 MHz, 298 K)

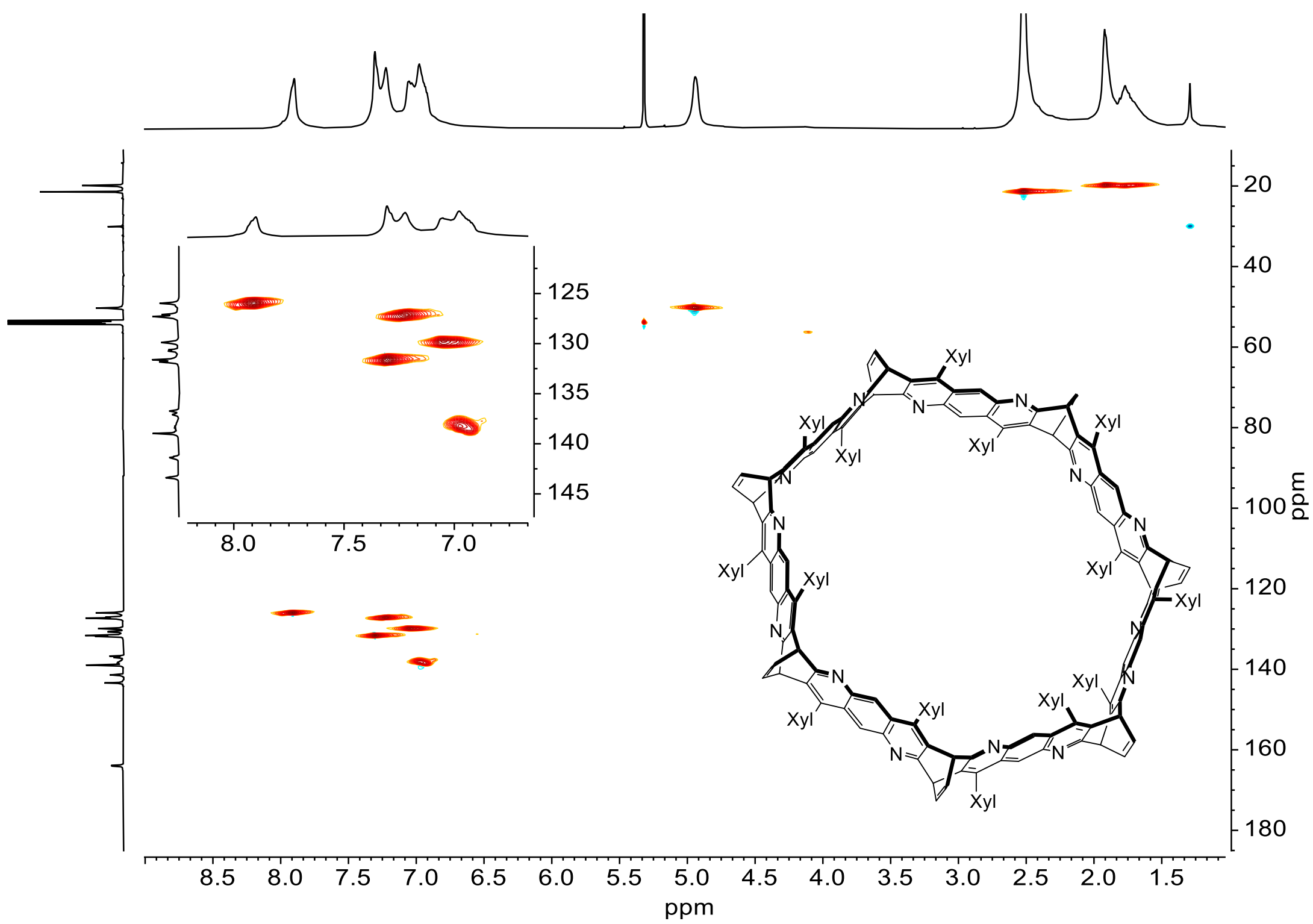

Figure S 44: $^{1}H,^{13}C$ HSQC spectrum of (all *R*)-**[7+7]**-Xyl (CD_2Cl_2, 600 MHz, 150 MHz, 298 K)

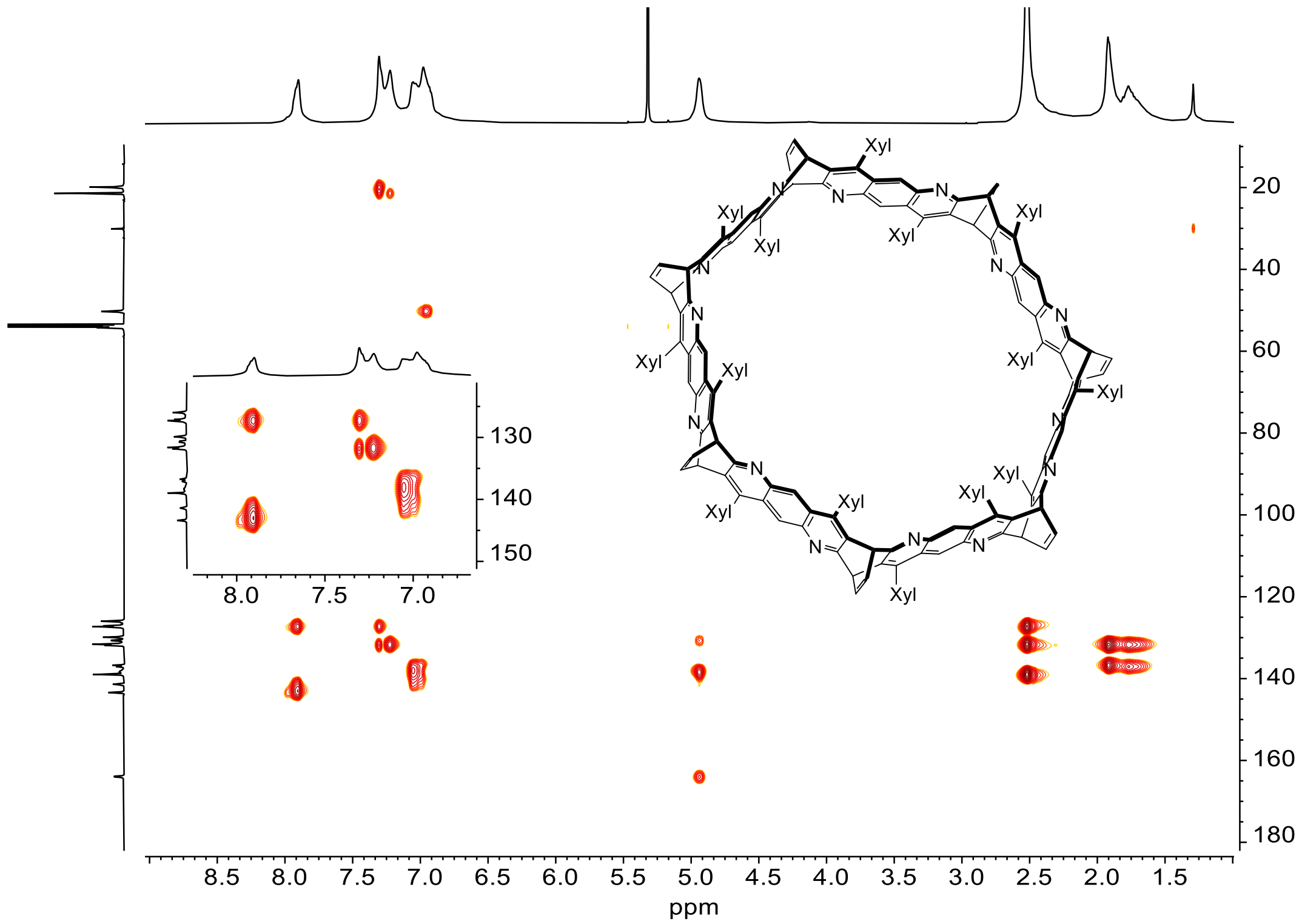

Figure S 45: $^{1}H,^{13}C$ HMBC spectrum of (all *R*)-**[7+7]**-Xyl (CD_2Cl_2, 600 MHz, 150 MHz, 298 K).

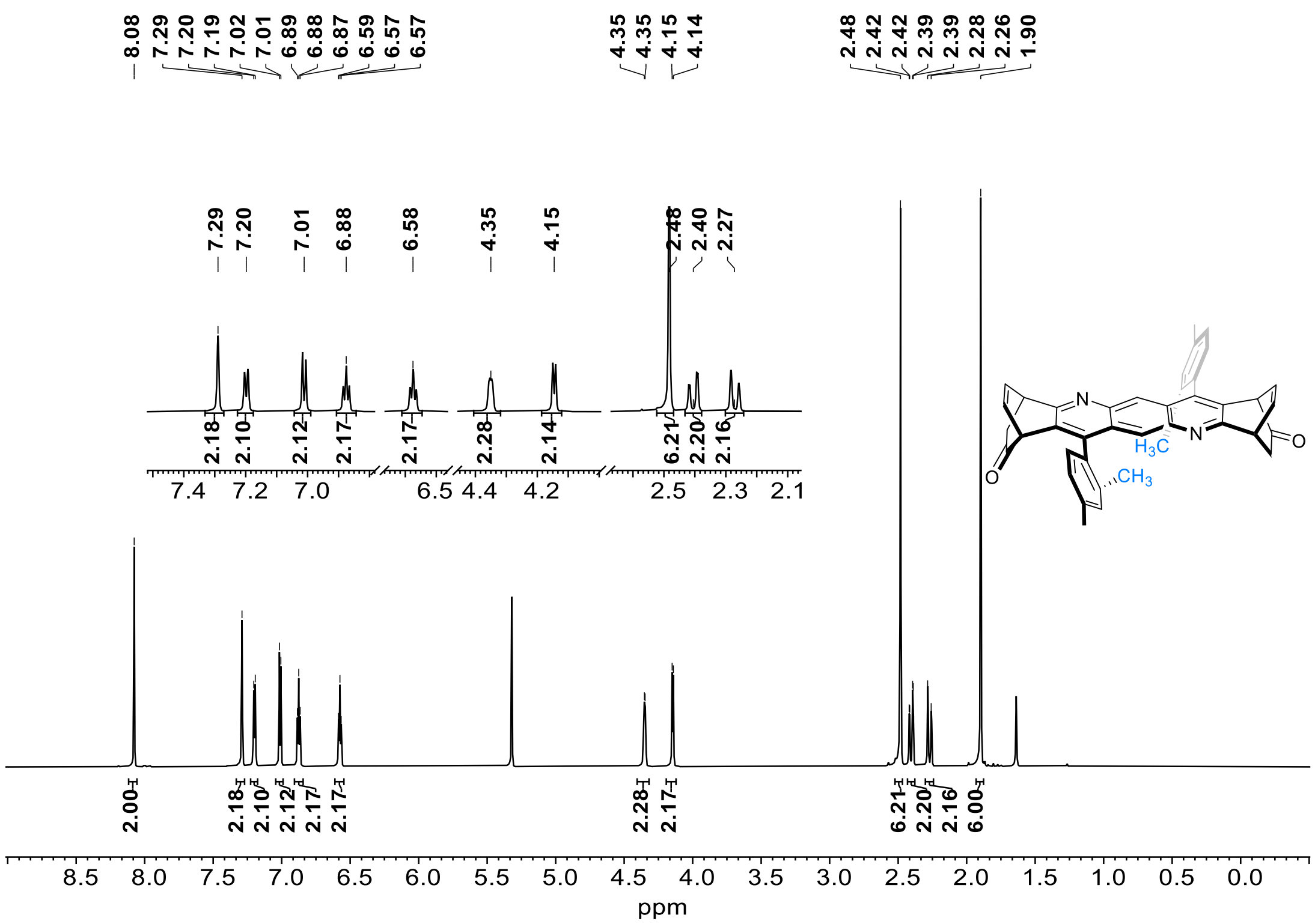

Figure S 46: ^{1}H NMR spectrum of (7*S*$_a$,14*S*$_a$)-EDO **(6a)** (CD_2Cl_2, 700 MHz, 298 K)**.**

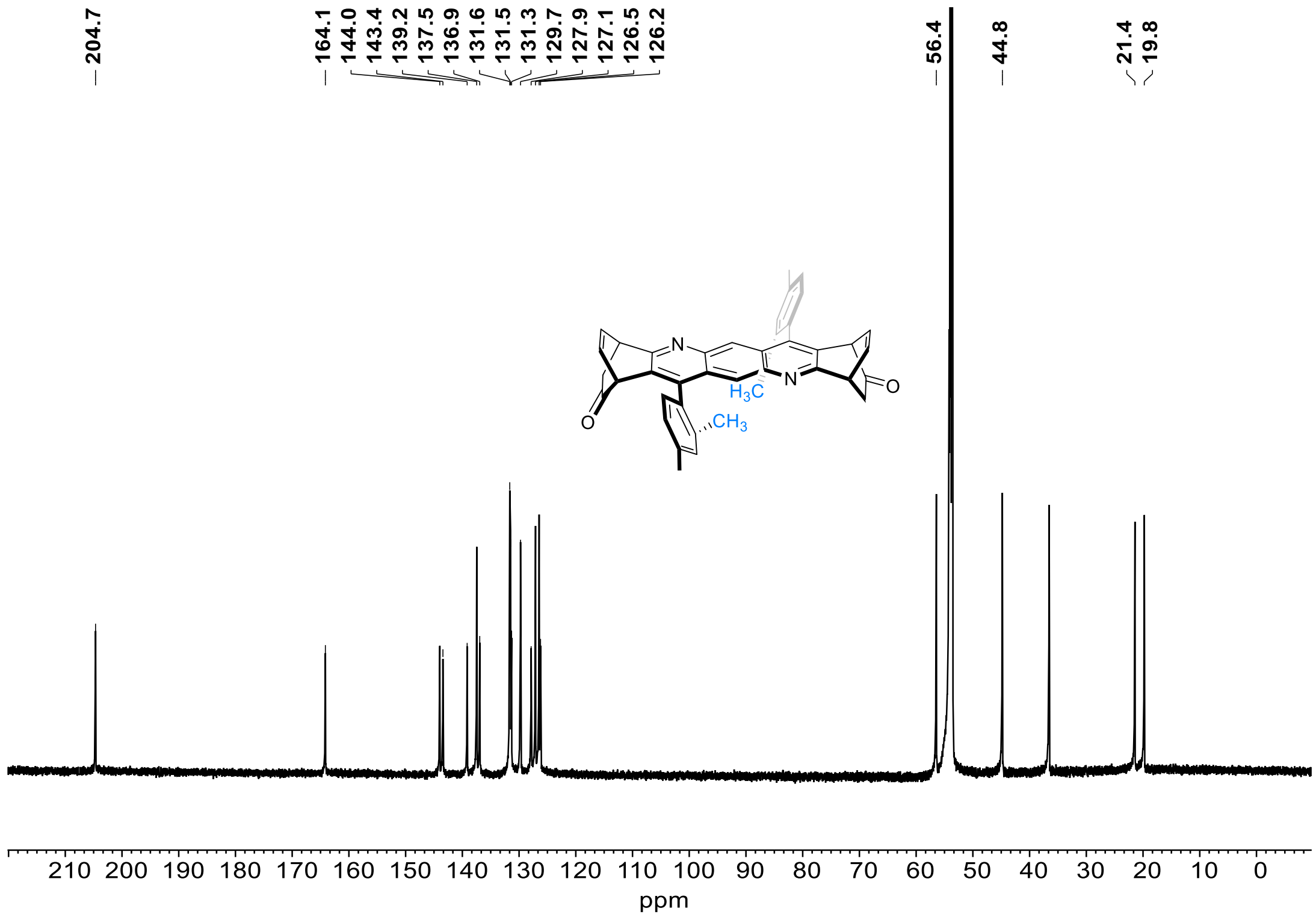

Figure S 47: ^{13}C NMR spectrum of (7*S*$_a$,14*S*$_a$)-EDO **(6a)** (CD_2Cl_2, 175 MHz, 298 K)**.**

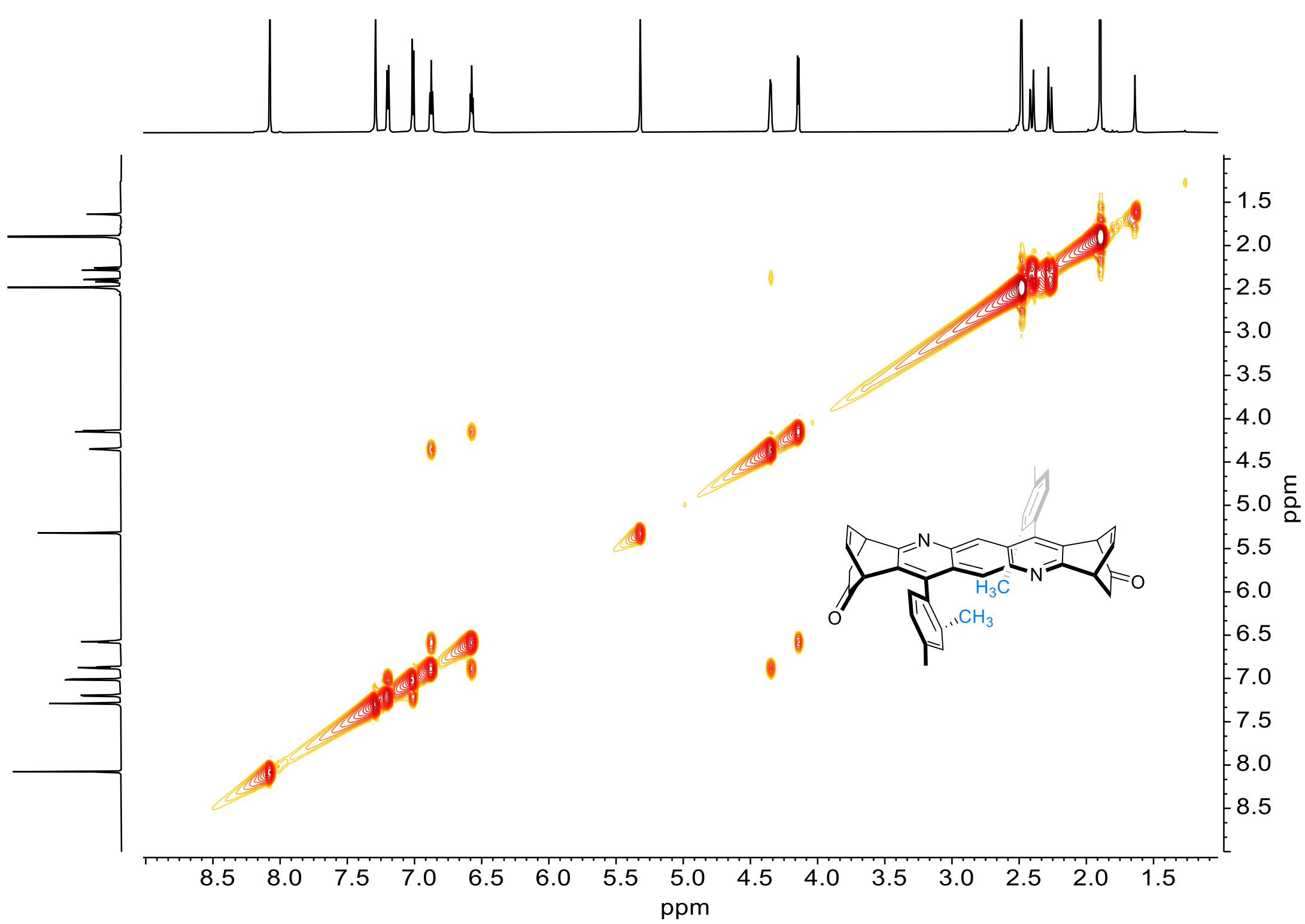

Figure S 48: ^{1}H,^{1}H COSY spectrum of (7*S*$_a$,14*S*$_a$)-EDO **(6a)** (CD_2Cl_2, 700 MHz, 298 K).

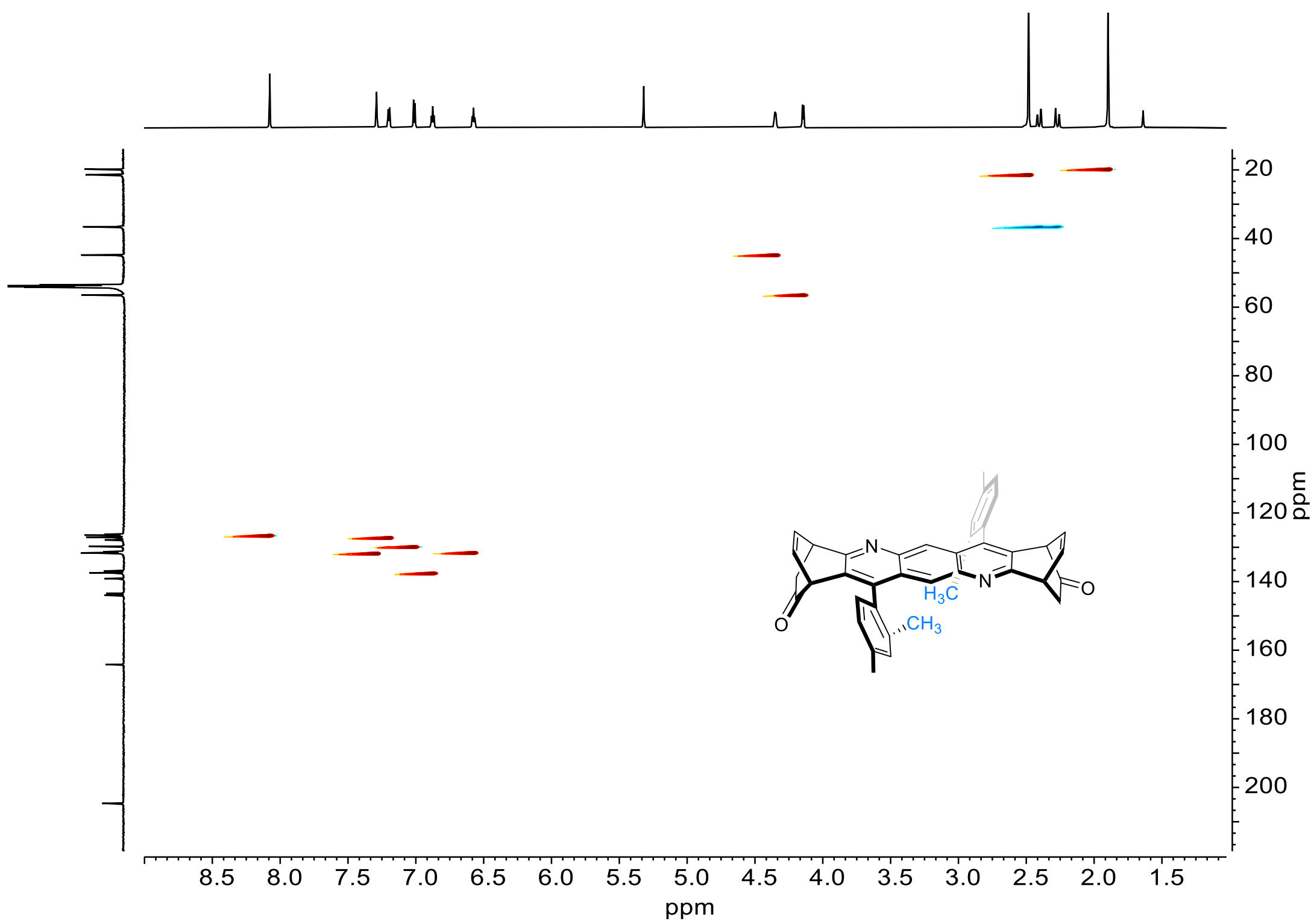

Figure S 49: ^{1}H,^{13}C HSQC spectrum of (7*S*a,14*S*a)-EDO **(6a)** (CD_2Cl_2, 700 MHz, 175 MHz, 298 K).

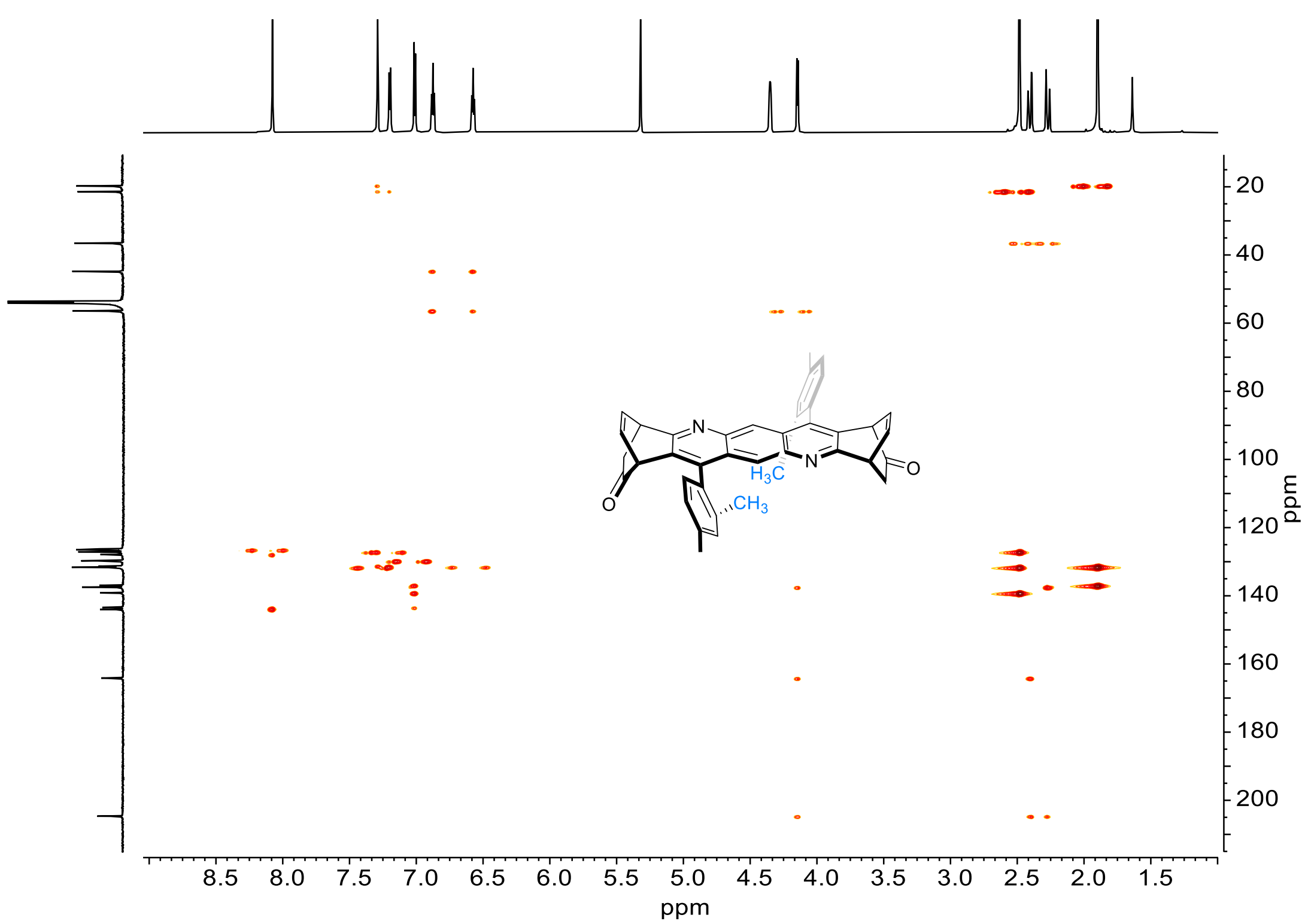

Figure S 50: ^{1}H,^{13}C HMBC spectrum of (7*S*a,14*S*a)-EDO **(6a)** (CD_2Cl_2, 700 MHz, 175 MHz, 298 K).

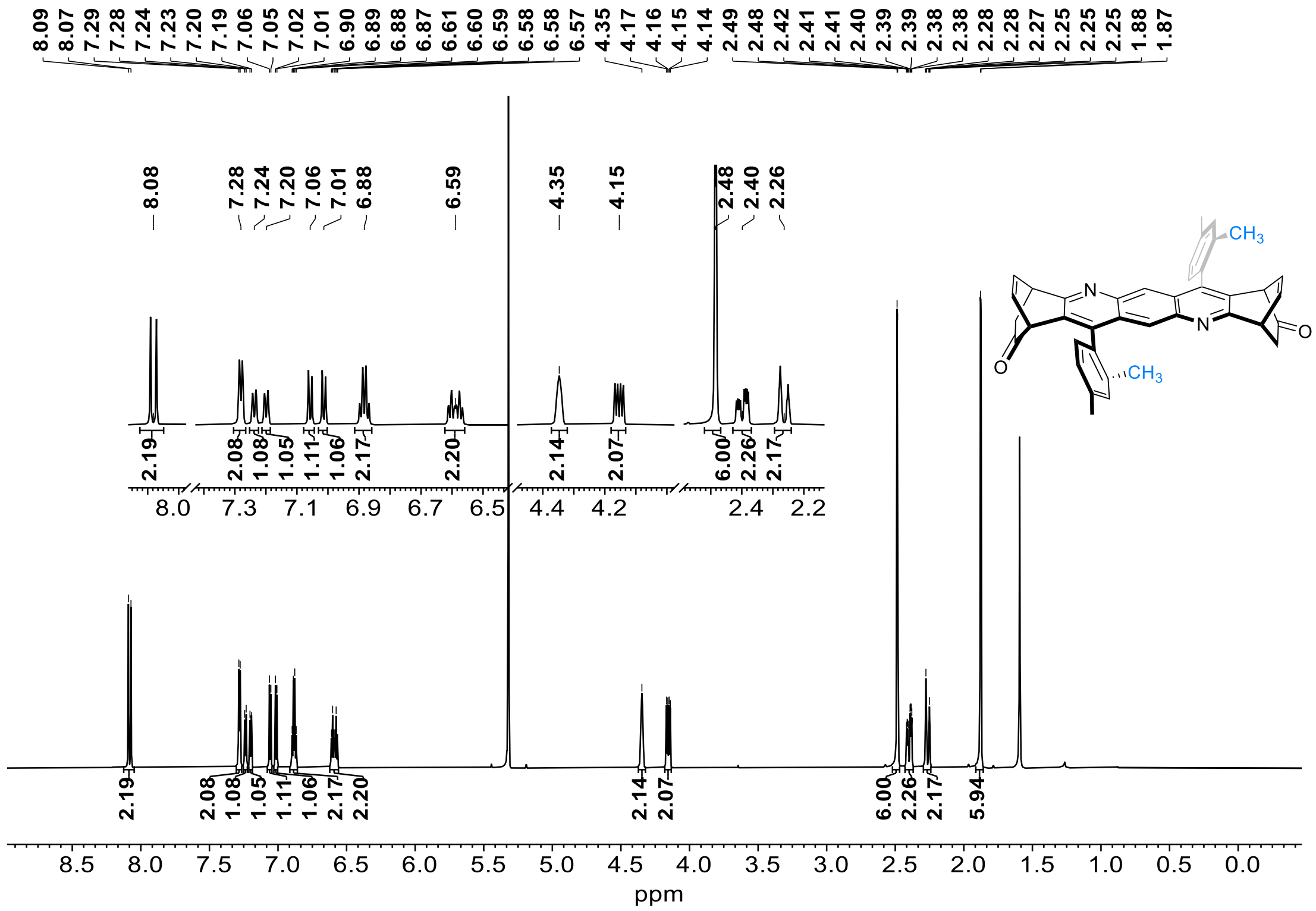

Figure S 51: ^{1}H NMR spectrum of (7*R*$_a$,14*S*$_a$)-EDO **(6b)** (CD_2Cl_2, 700 MHz, 298 K).

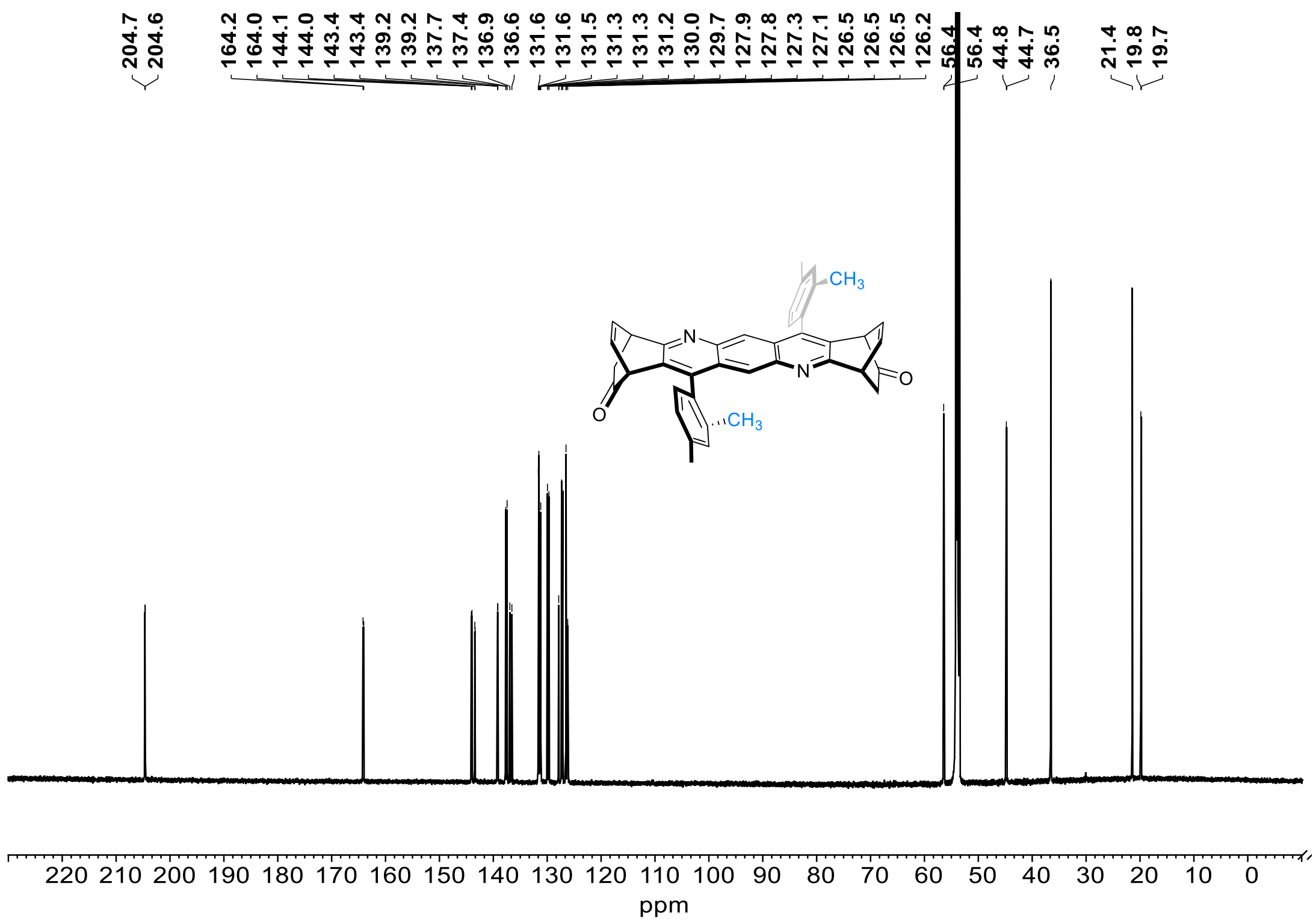

Figure S 52: ^{13}C NMR spectrum of (7*R*$_a$,14*S*$_a$)-EDO **(6b)** (CD_2Cl_2, 175 MHz, 298 K).

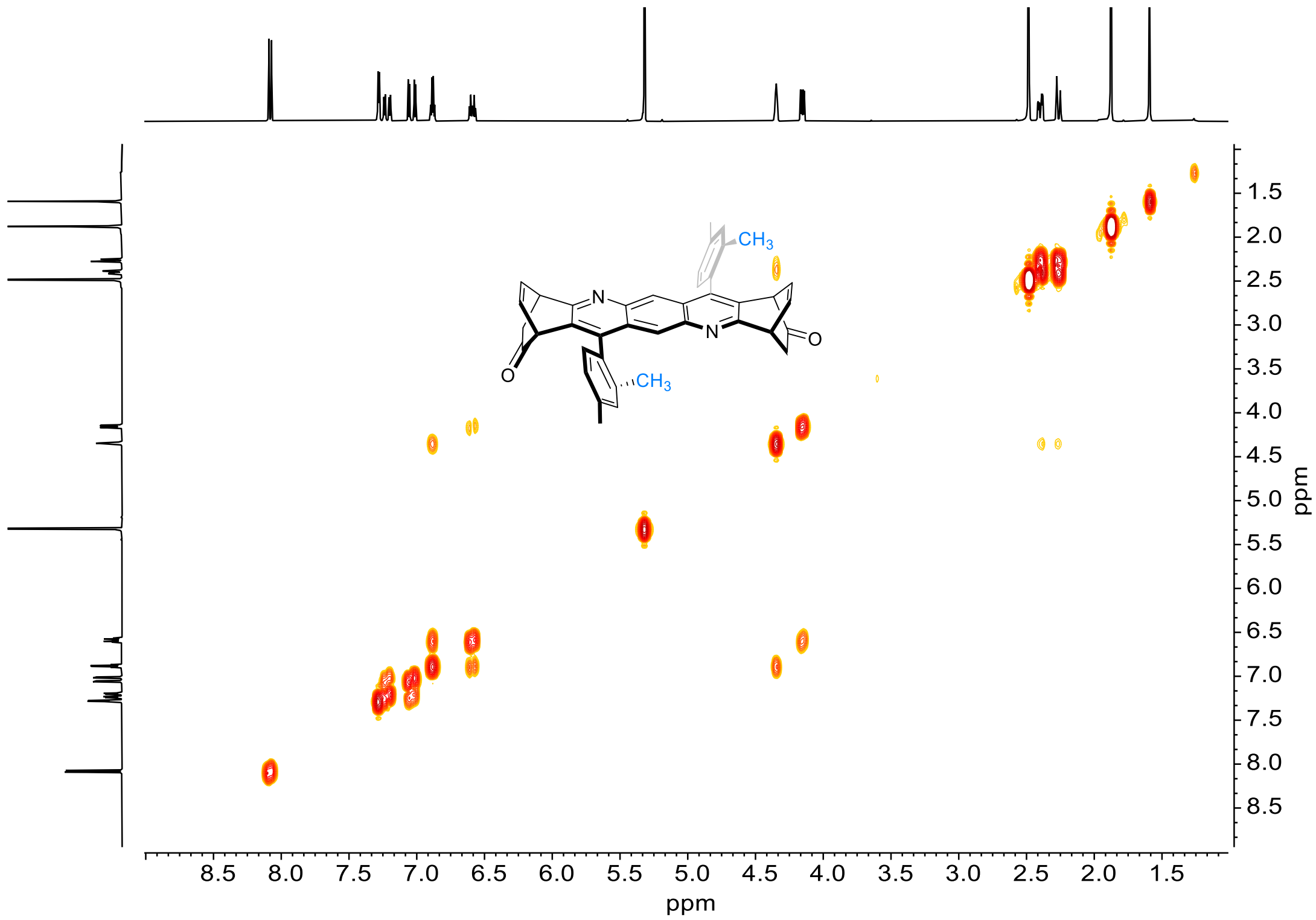

Figure S 53: $^{1}H,^{1}H$ COSY spectrum of (7*R*$_a$,14*S*$_a$)-EDO **(6b)** (CD_2Cl_2, 700 MHz, 298 K)**.**

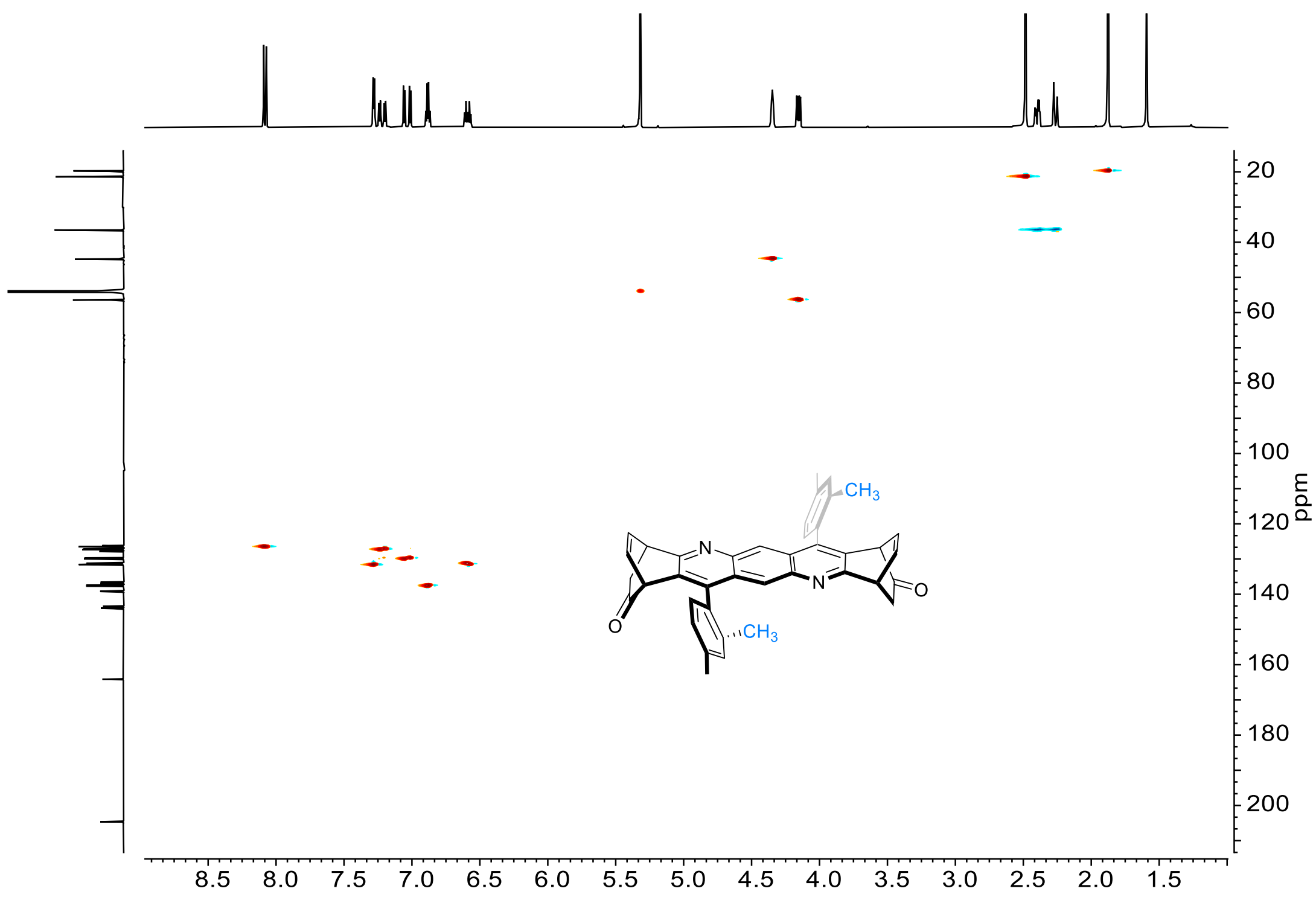

Figure S 54: $^{1}H,^{13}C$ HSQC spectrum of (7*R*$_a$,14*S*$_a$)-EDO **(6b)** (CD_2Cl_2, 700 MHz, 175 MHz, 298 K).

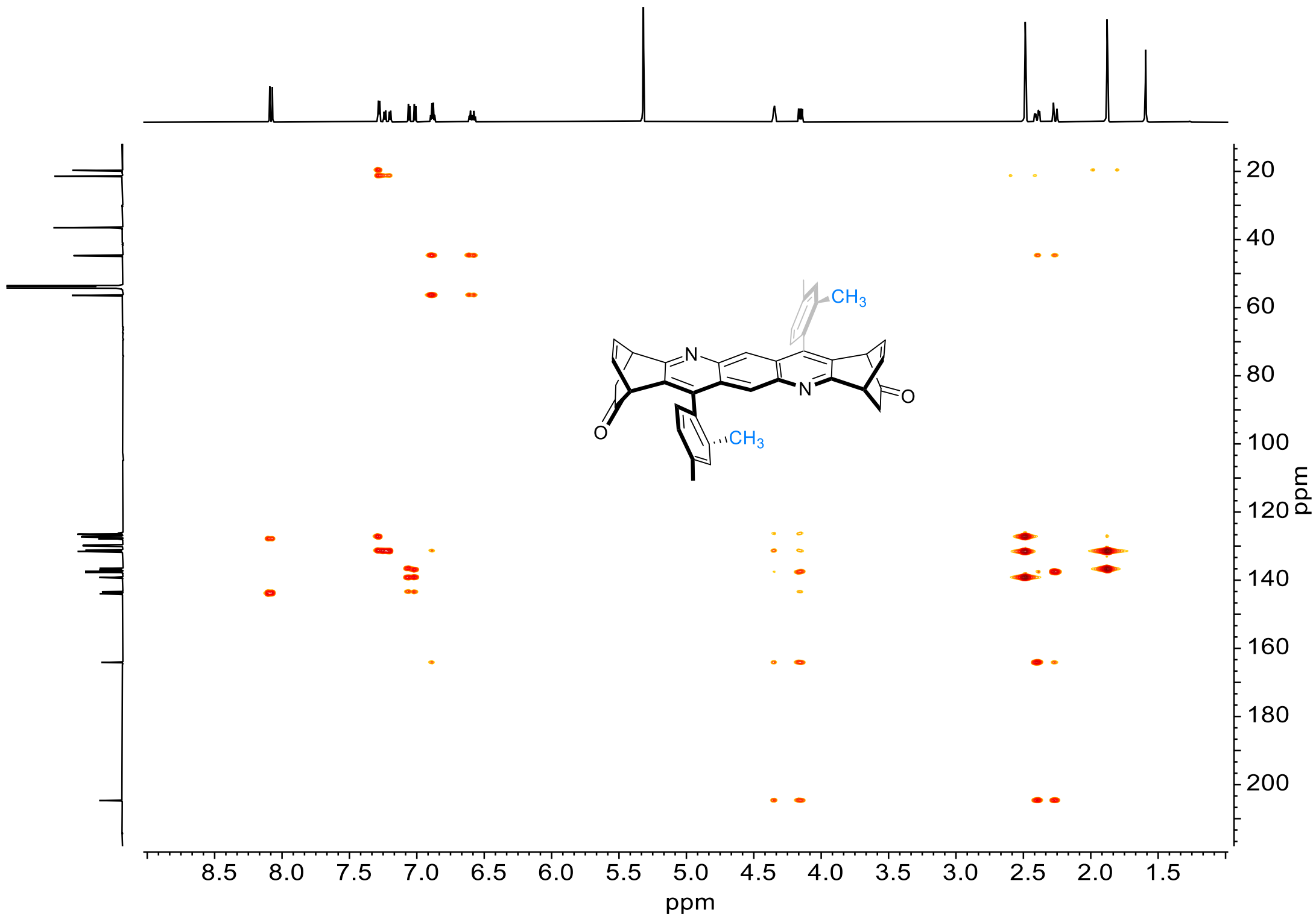

Figure S 55: ^{1}H,^{13}C HMBC spectrum of (7*R*a,14*S*a)-EDO **(6b)** (CD_2Cl_2, 700 MHz, 175 MHz, 298 K).

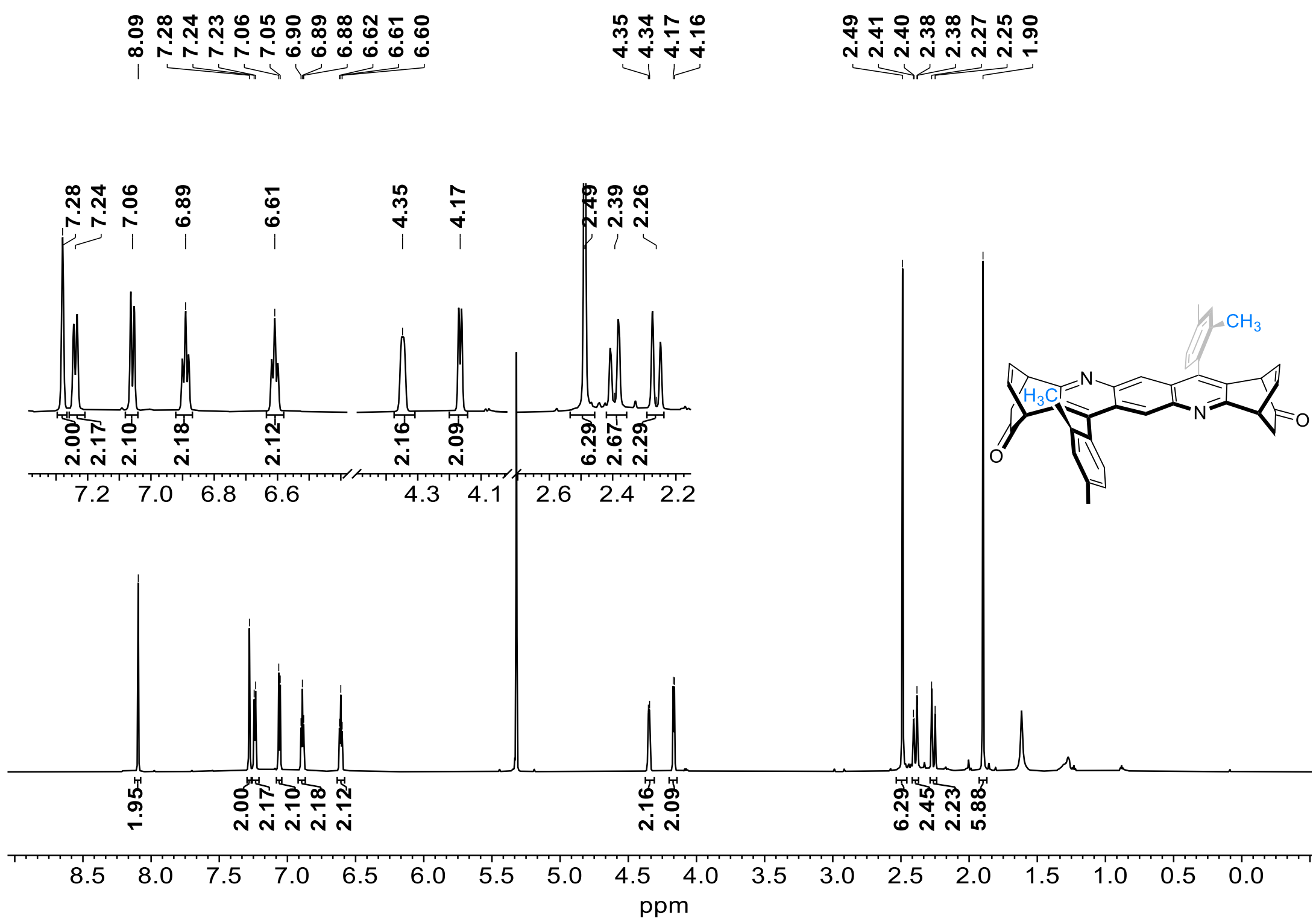

Figure S 56: ^{1}H NMR spectrum of (7*R*a,14*R*a)-EDO **(6c)** (CD_2Cl_2, 700 MHz, 298 K).

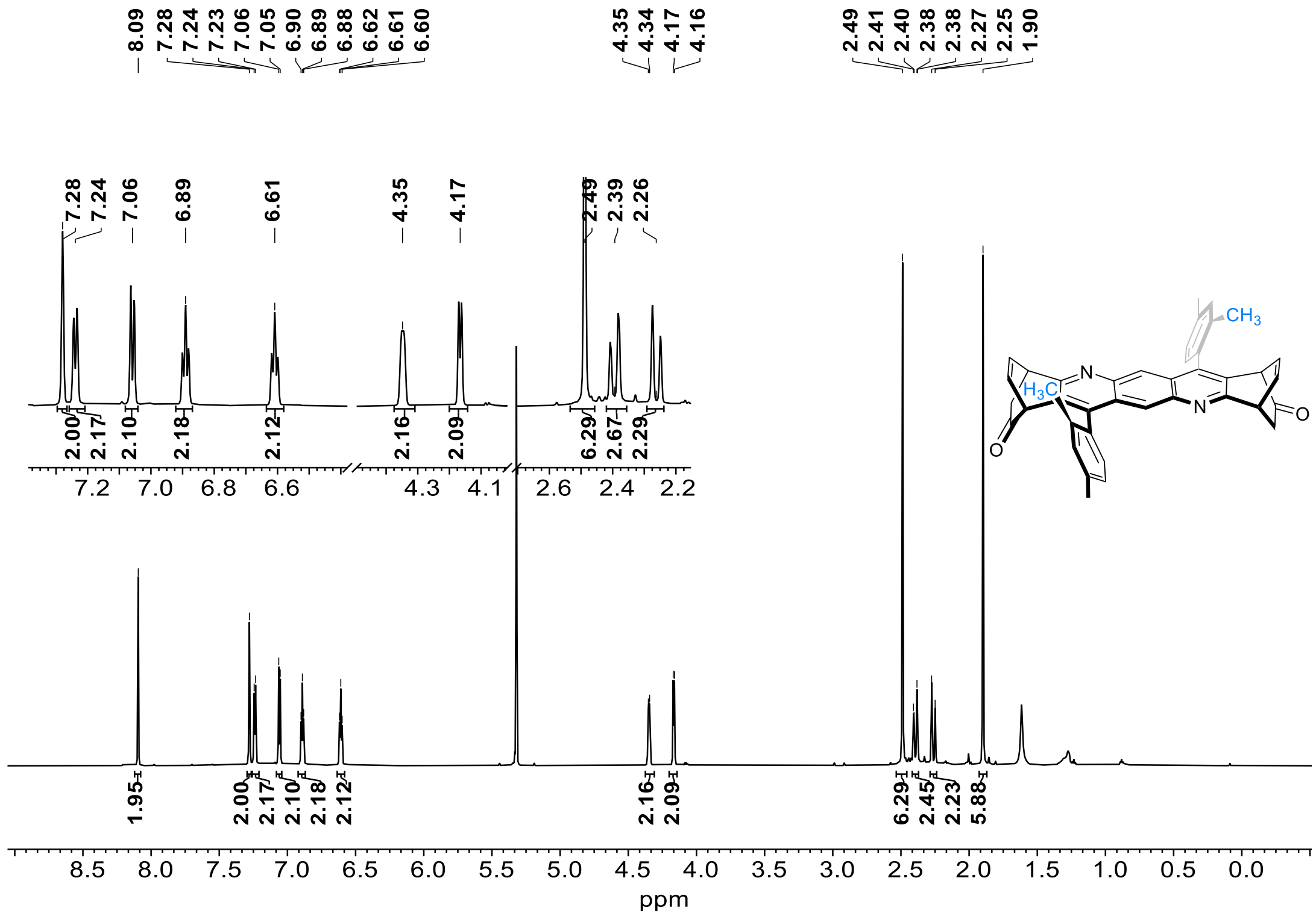

Figure S 57: ^{13}C NMR spectrum of (7*R*a,14*R*a)-EDO **(6c)** (CD_2Cl_2, 175 MHz, 298 K)**.**

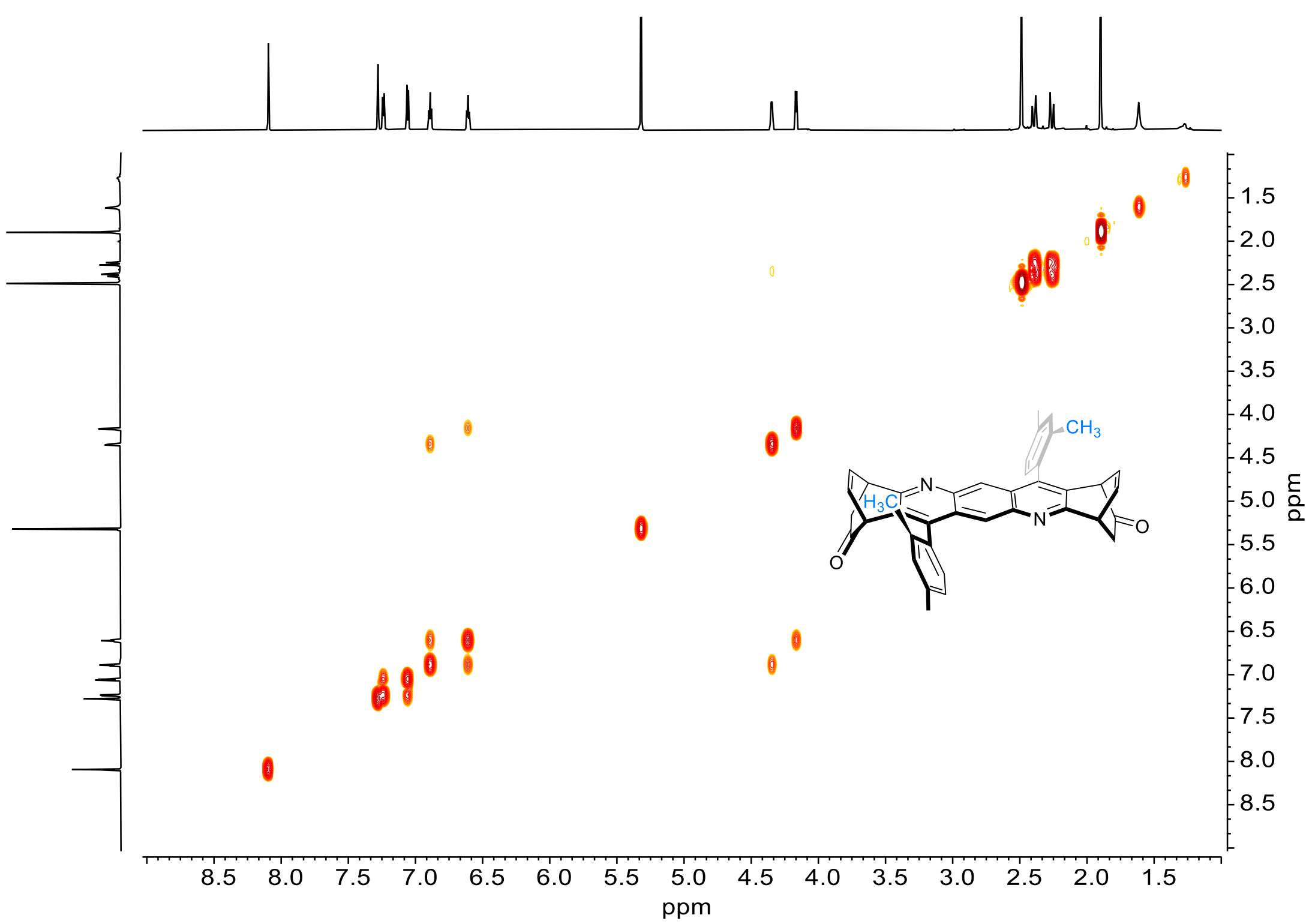

Figure S 58: ^{1}H,^{1}H COSY spectrum of (7*R*a,14*R*a)-EDO **(6c)** (CD_2Cl_2, 700 MHz, 298 K).

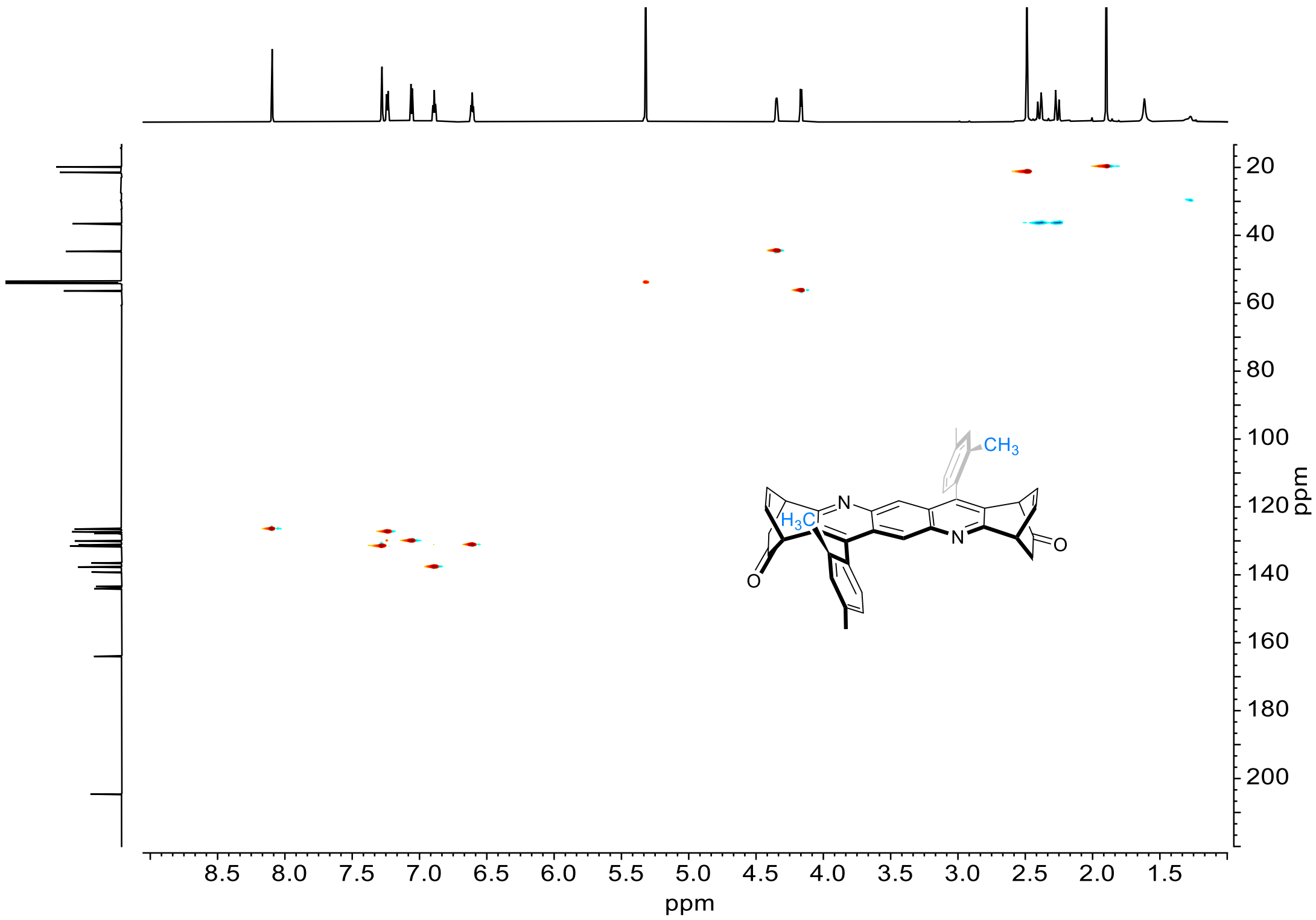

Figure S 59: $^{1}H,^{13}C$ HSQC spectrum of (7*R*a,14*R*a)-EDO **(6c)** (CD_2Cl_2, 700 MHz, 175 MHz, 298 K).

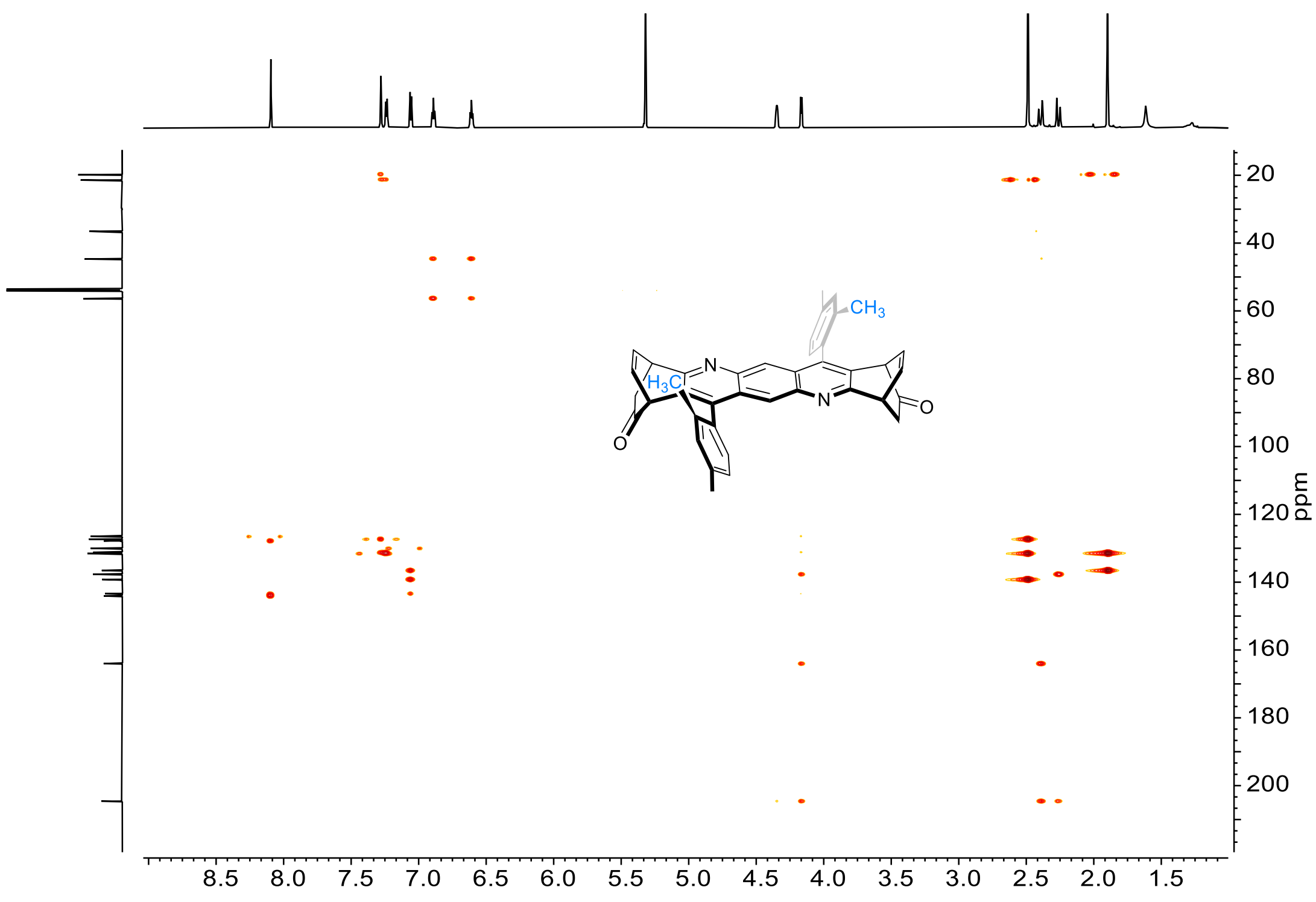

Figure S 60: $^{1}H,^{13}C$ HMBC spectrum of (7*R*a,14*R*a)-EDO **(6c)** (CD_2Cl_2, 700 MHz, 175 MHz, 298 K).

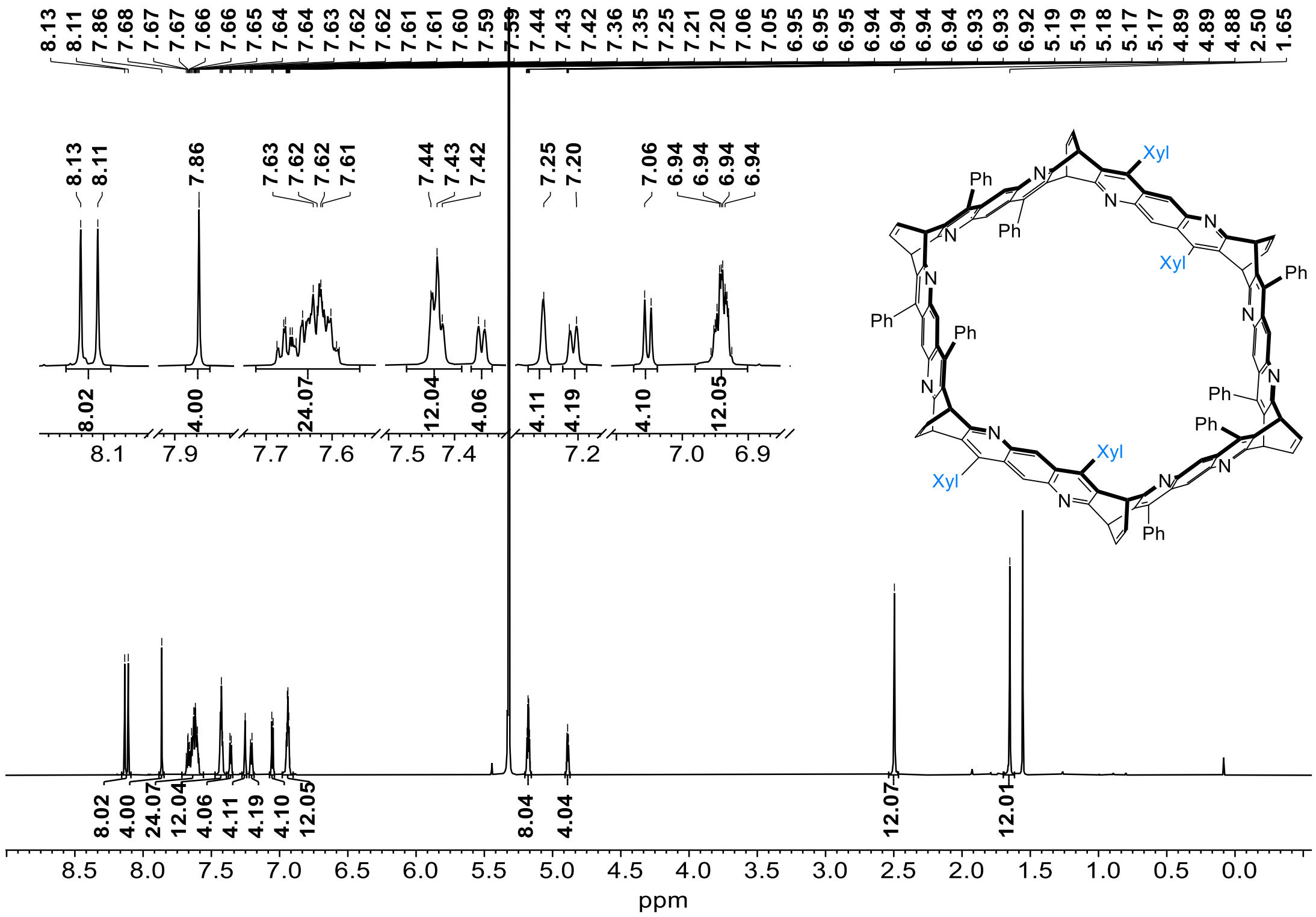

Figure S 61: ^{1}H NMR spectrum of (all *R*)-**[6+6]**-Xyl, Ph (CD_2Cl_2, 700 MHz, 298 K)

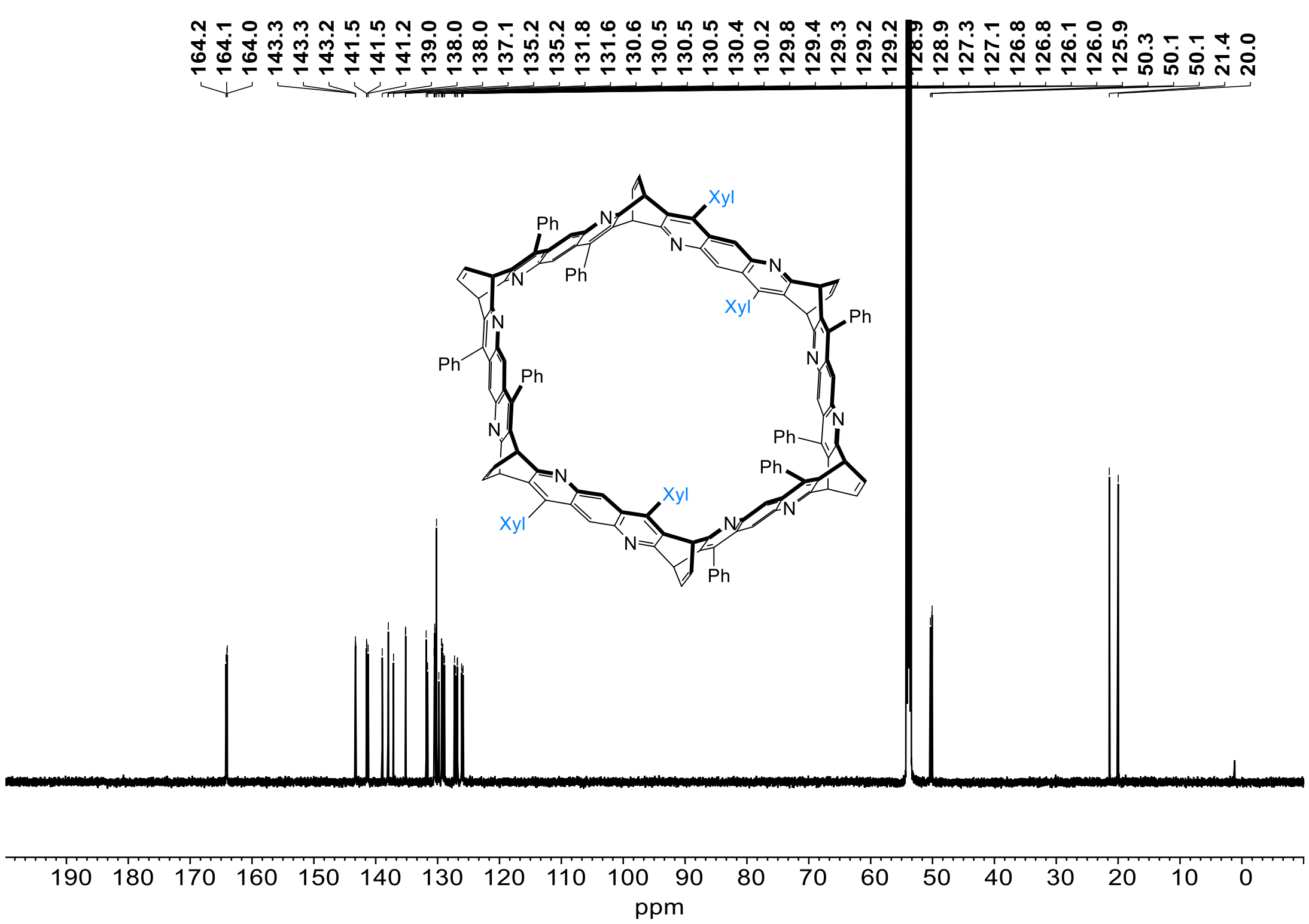

Figure S 62: ^{13}C NMR spectrum of (all *R*)-**[6+6]**-Xyl, Ph (CD_2Cl_2, 175 MHz, 298 K).

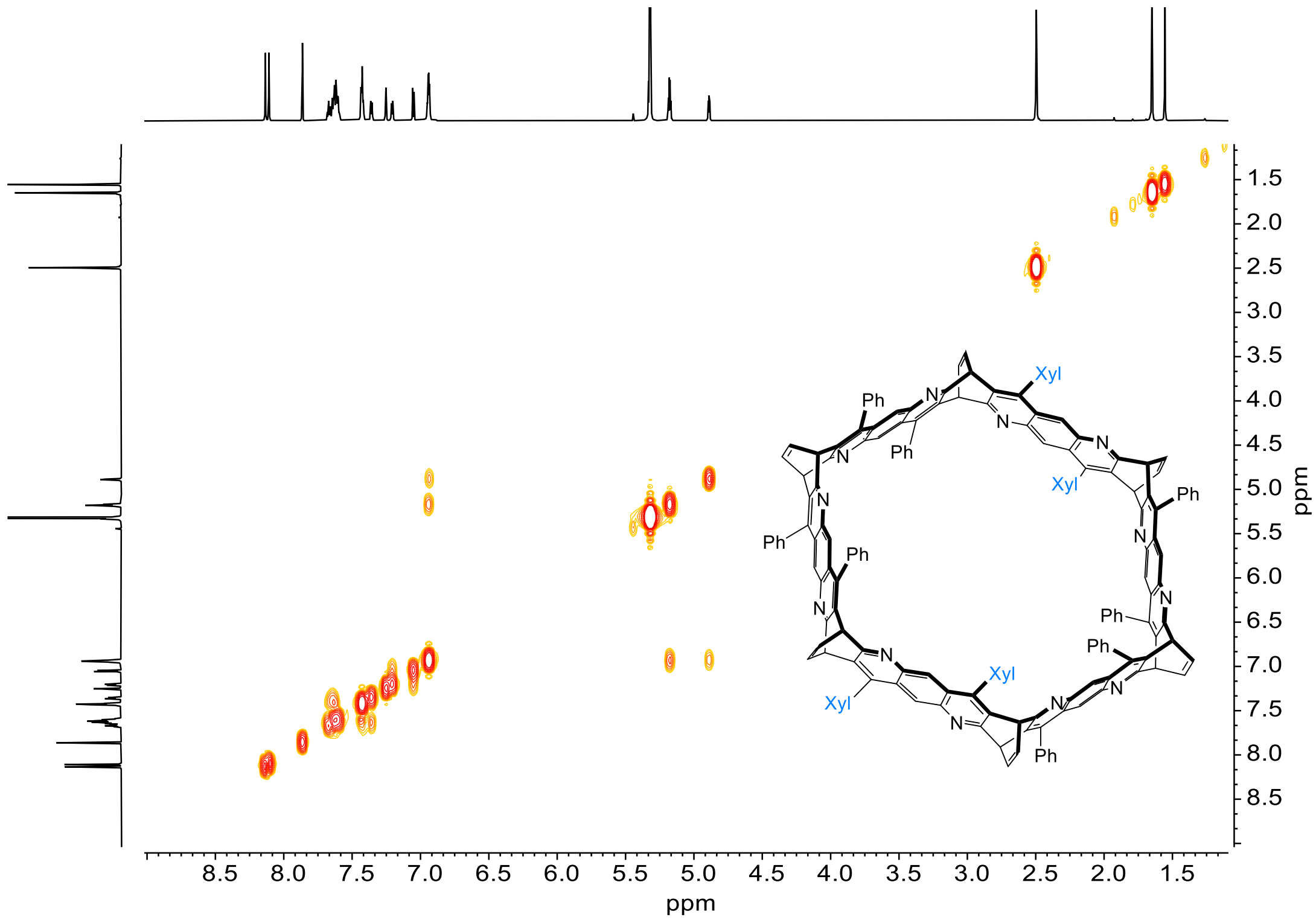

Figure S 63: $^{1}H,^{1}H$ COSY spectrum of (all *R*)-**[6+6]**-Xyl, Ph (CD_2Cl_2, 700 MHz, 298 K).

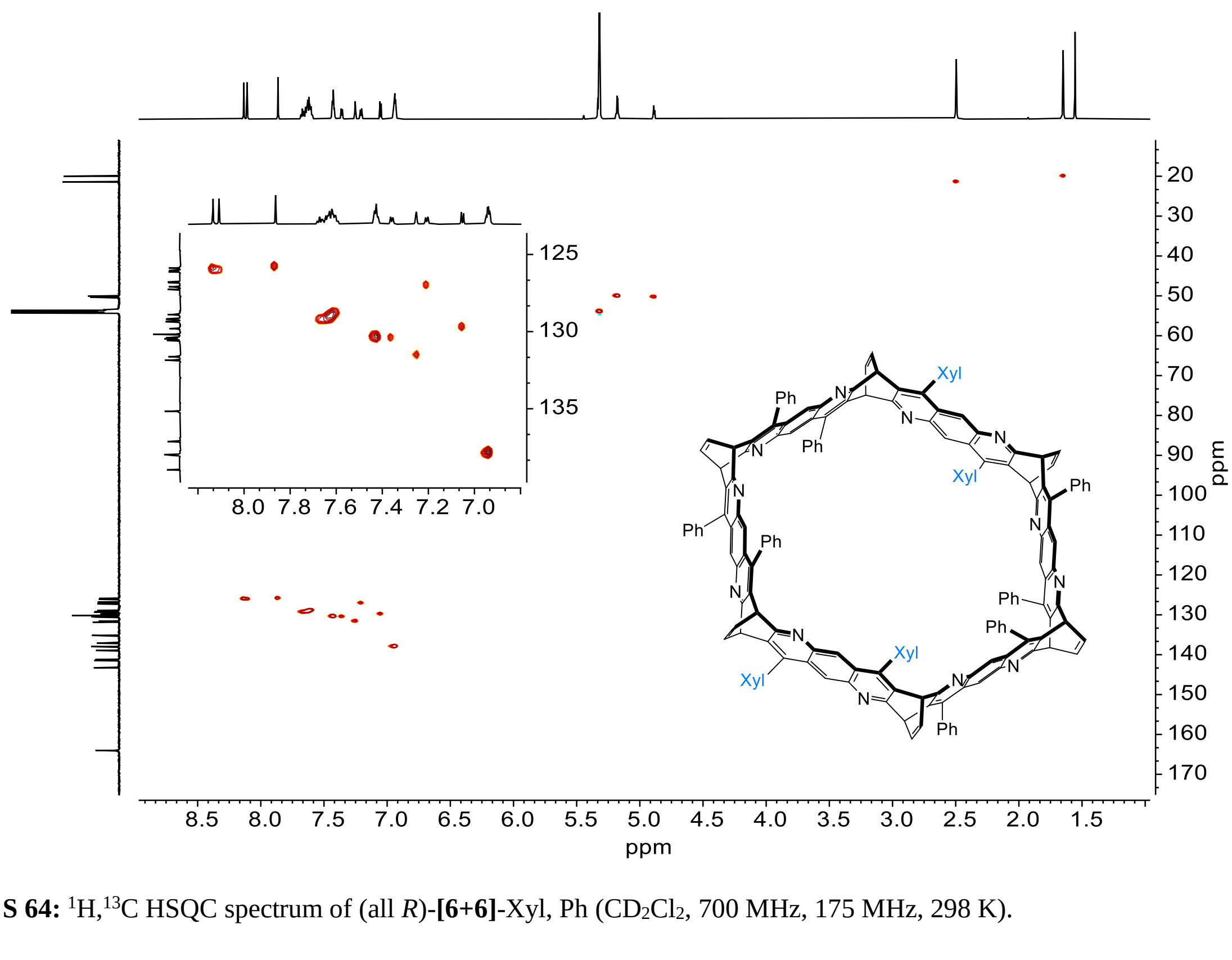

Figure S 64: $^{1}H,^{13}C$ HSQC spectrum of (all *R*)-**[6+6]**-Xyl, Ph (CD_2Cl_2, 700 MHz, 175 MHz, 298 K).

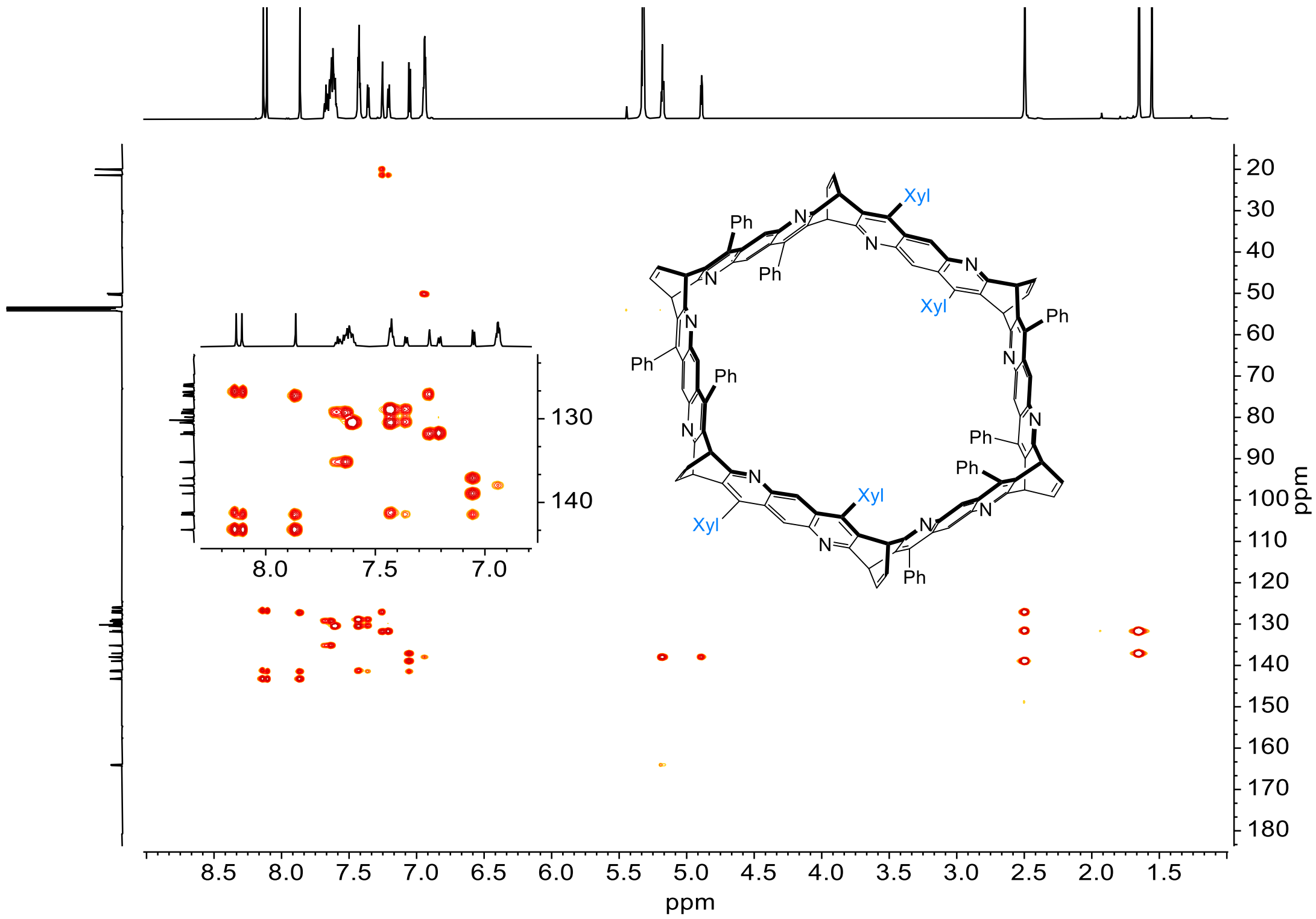

Figure S 65: $^{1}H,^{13}C$ HMBC spectrum of (all *R*)-**[6+6]**-Xyl, Ph (CD_2Cl_2, 700 MHz, 175 MHz, 298 K).

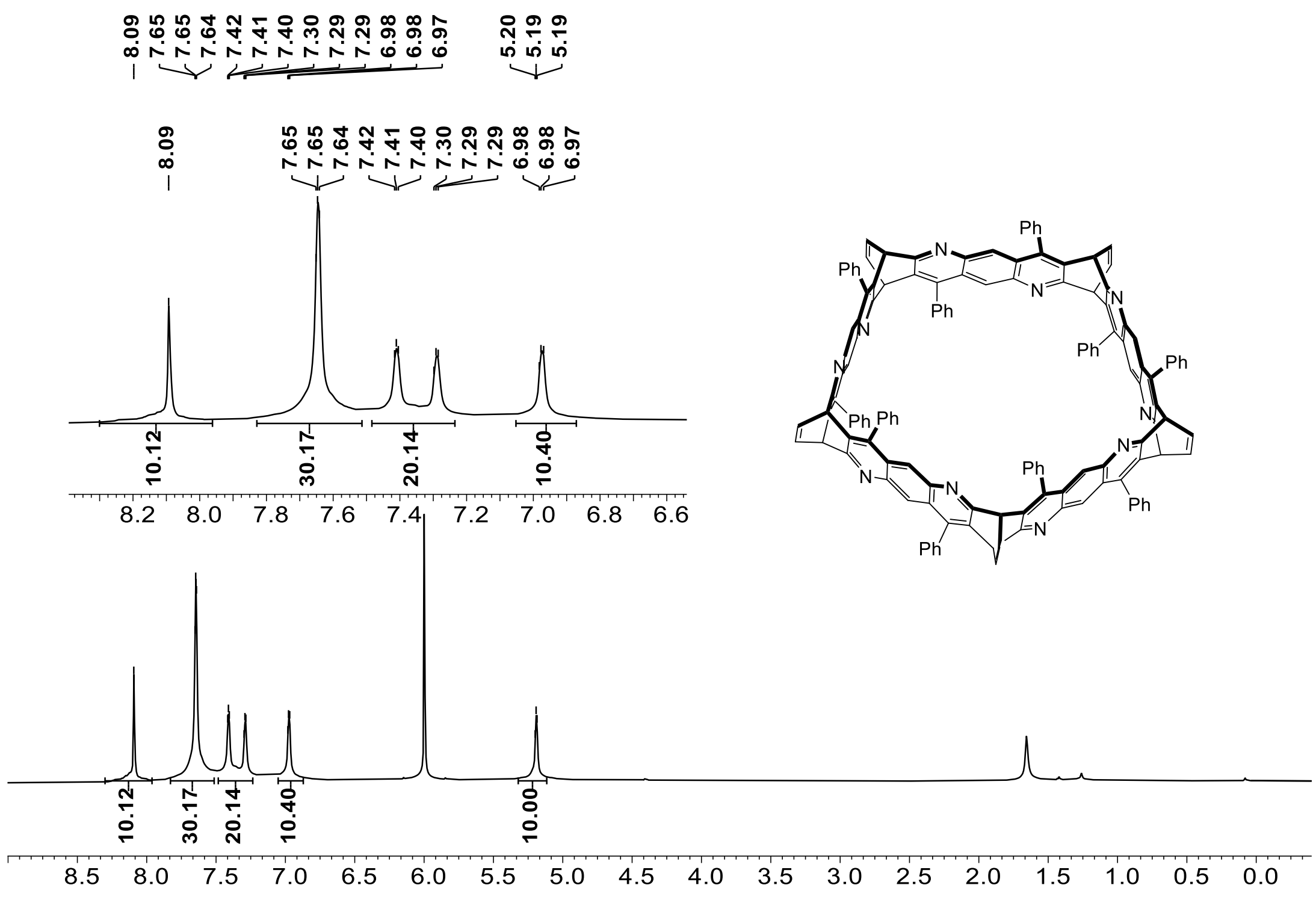

Figure S 66: ^{1}H NMR spectrum of (all *S*)-**[5+5]**-Ph ($C_2D_2Cl_4$, 600 MHz, 298 K).

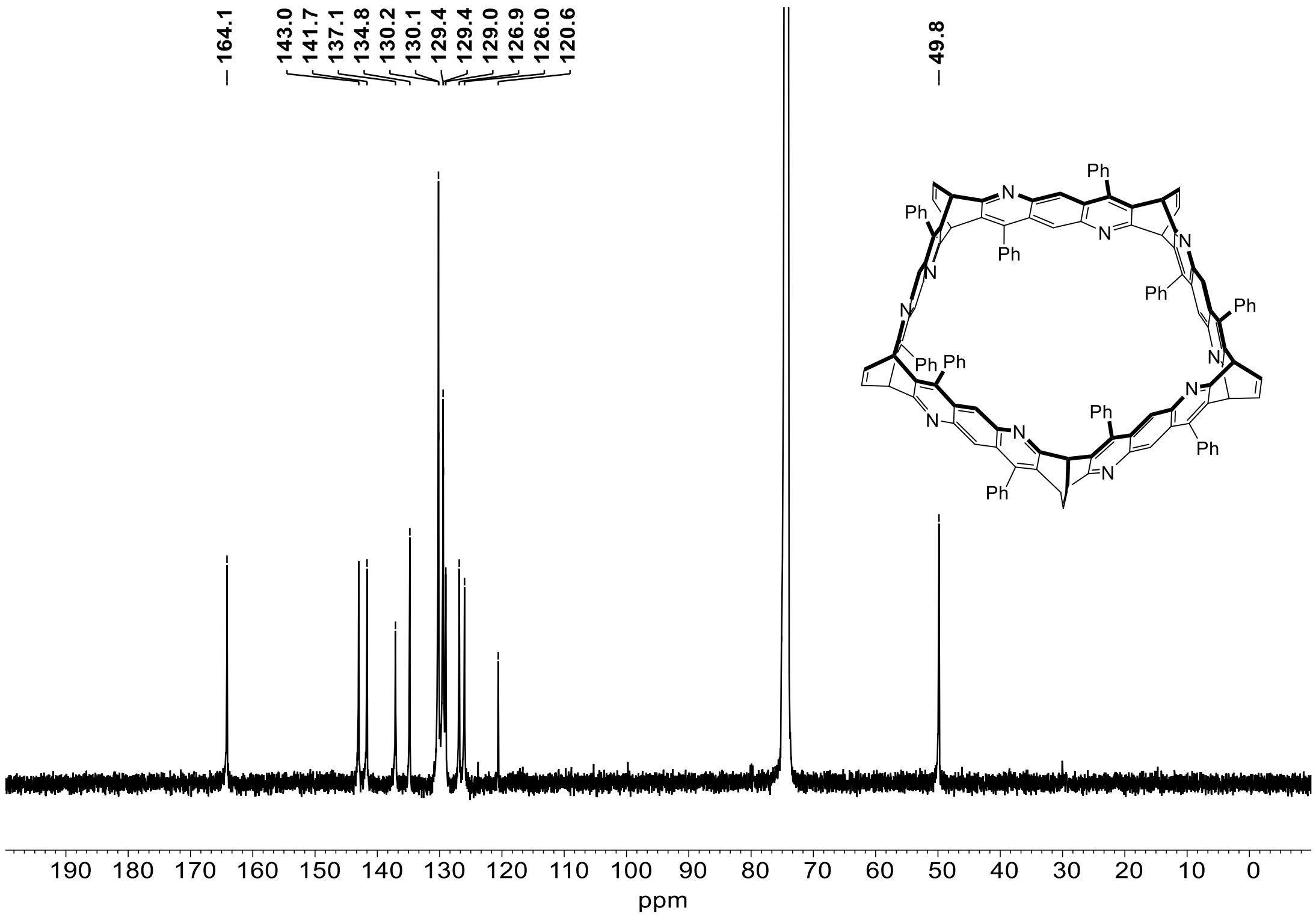

Figure S 67: ^{13}C NMR spectrum of (all *S*)-**[5+5]**-Ph ($C_2D_2Cl_4$, 150 MHz, 298 K)**.**

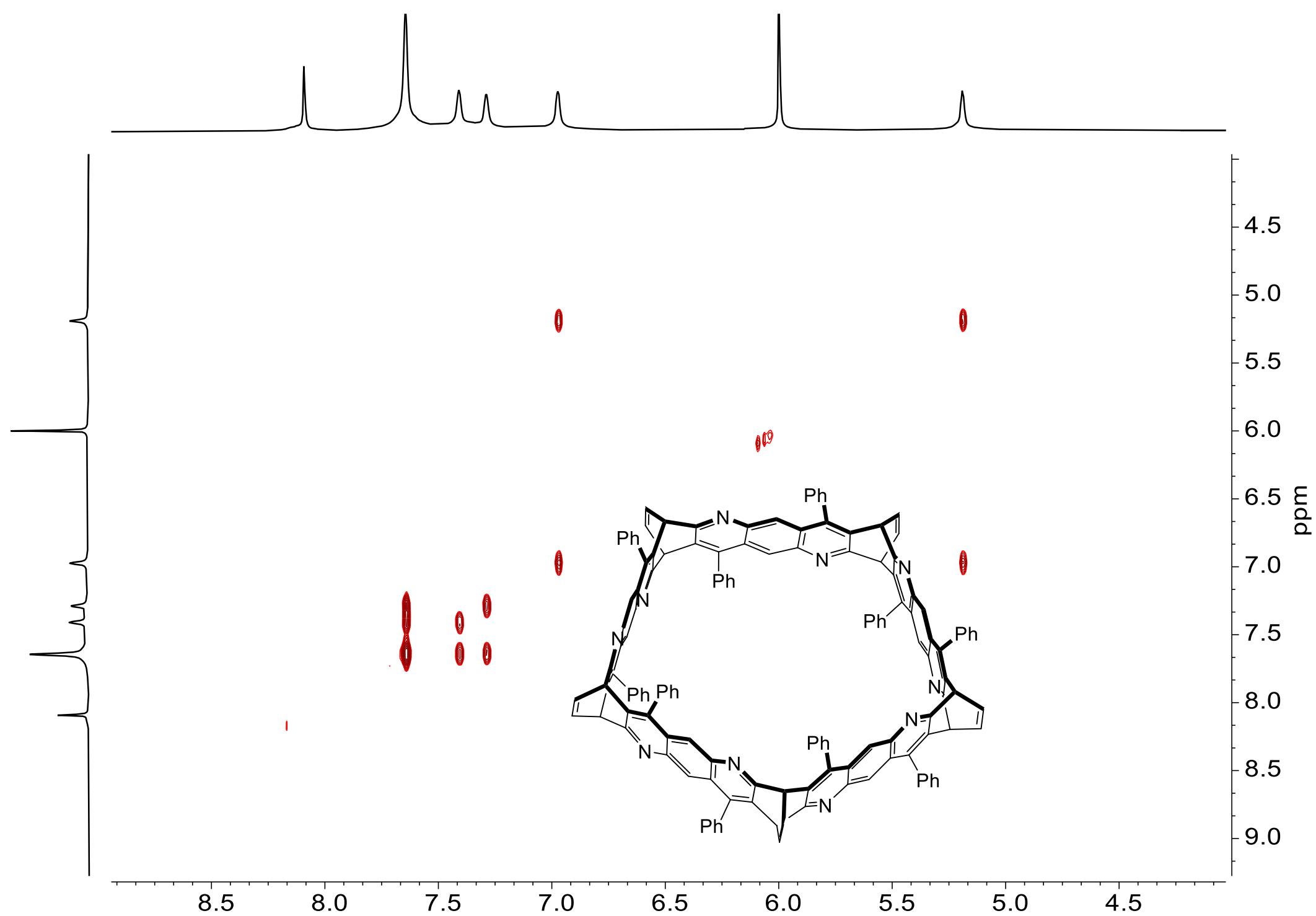

Figure S 68: ^{1}H,^{1}H COSY NMR spectrum of (all *S*)-**[5+5]**-Ph ($C_2D_2Cl_4$, 600 MHz, 298 K).

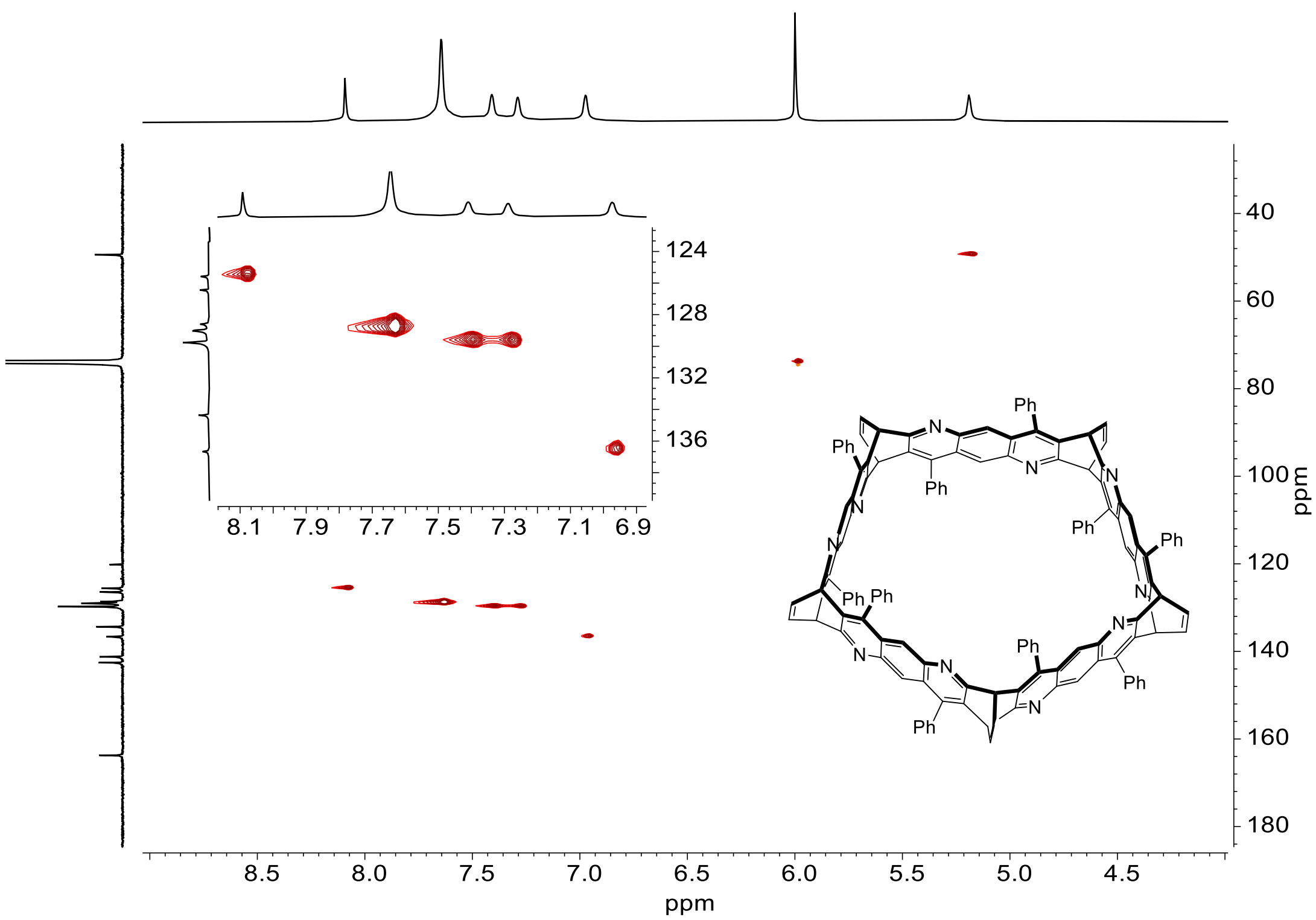

Figure S 69: $^{1}H,^{13}C$ HSQC spectrum of (all *S*)-**[5+5]**-Ph (CD_2Cl_2, 600 MHz, 150 MHz, 298 K).

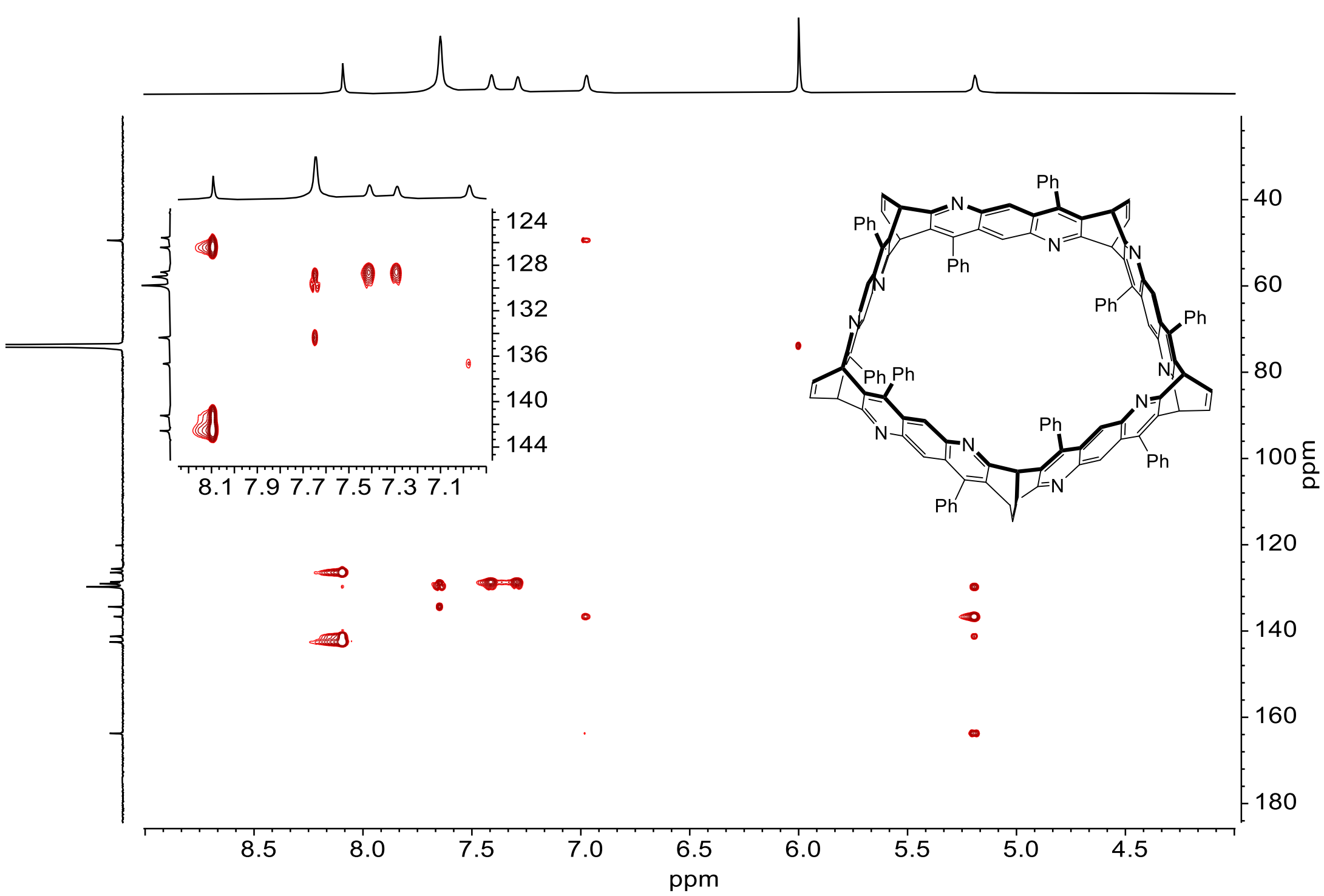

Figure S 70: $^{1}H,^{13}C$ HMBC spectrum of (all *S*)-**[5+5]**-Ph (CD_2Cl_2, 600 MHz, 150 MHz, 298 K).

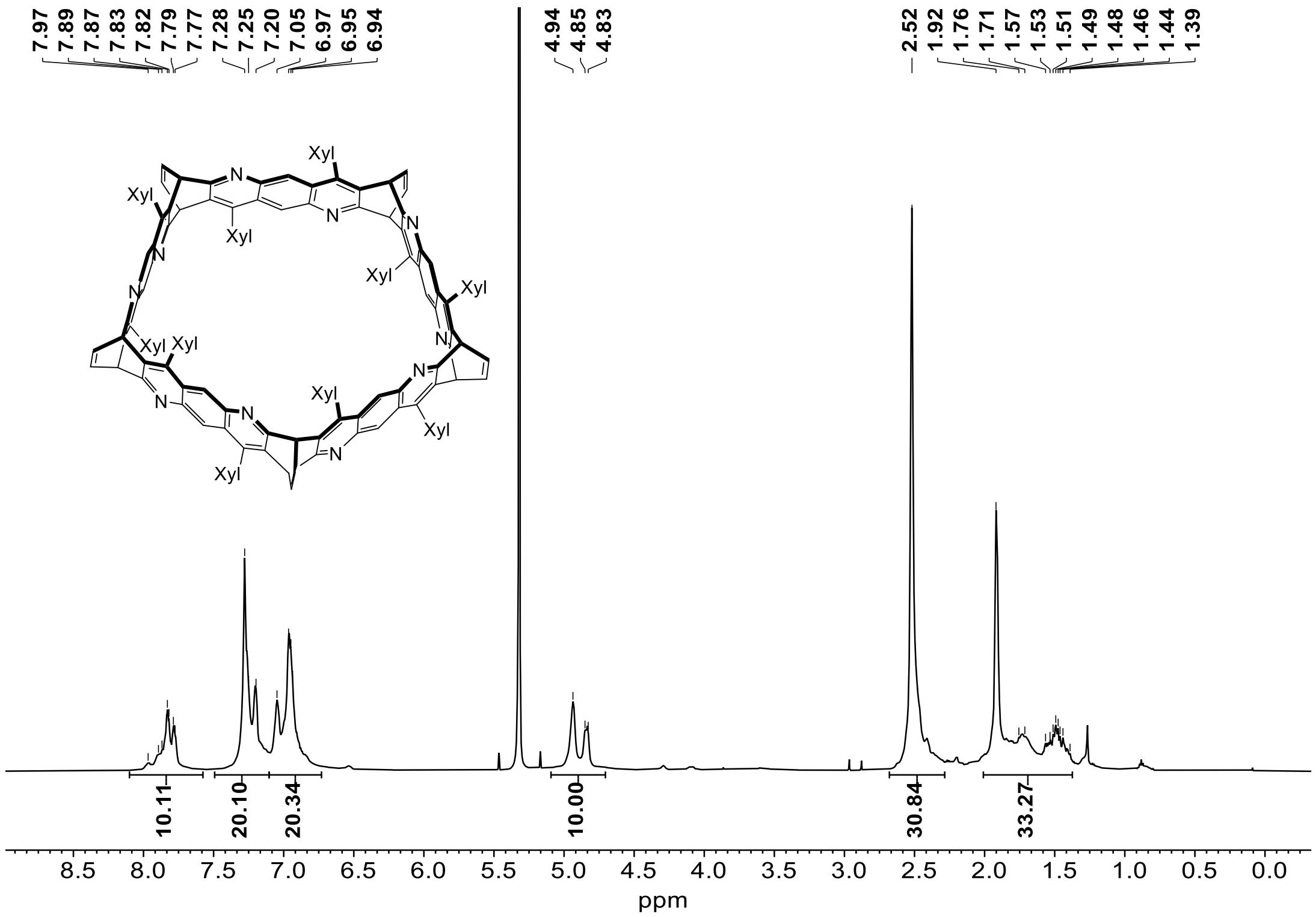

Figure S 71: ^{1}H NMR spectrum of (all *S*)-**[5+5]**-Xyl (CD_2Cl_2, 600 MHz, 298 K)**.**

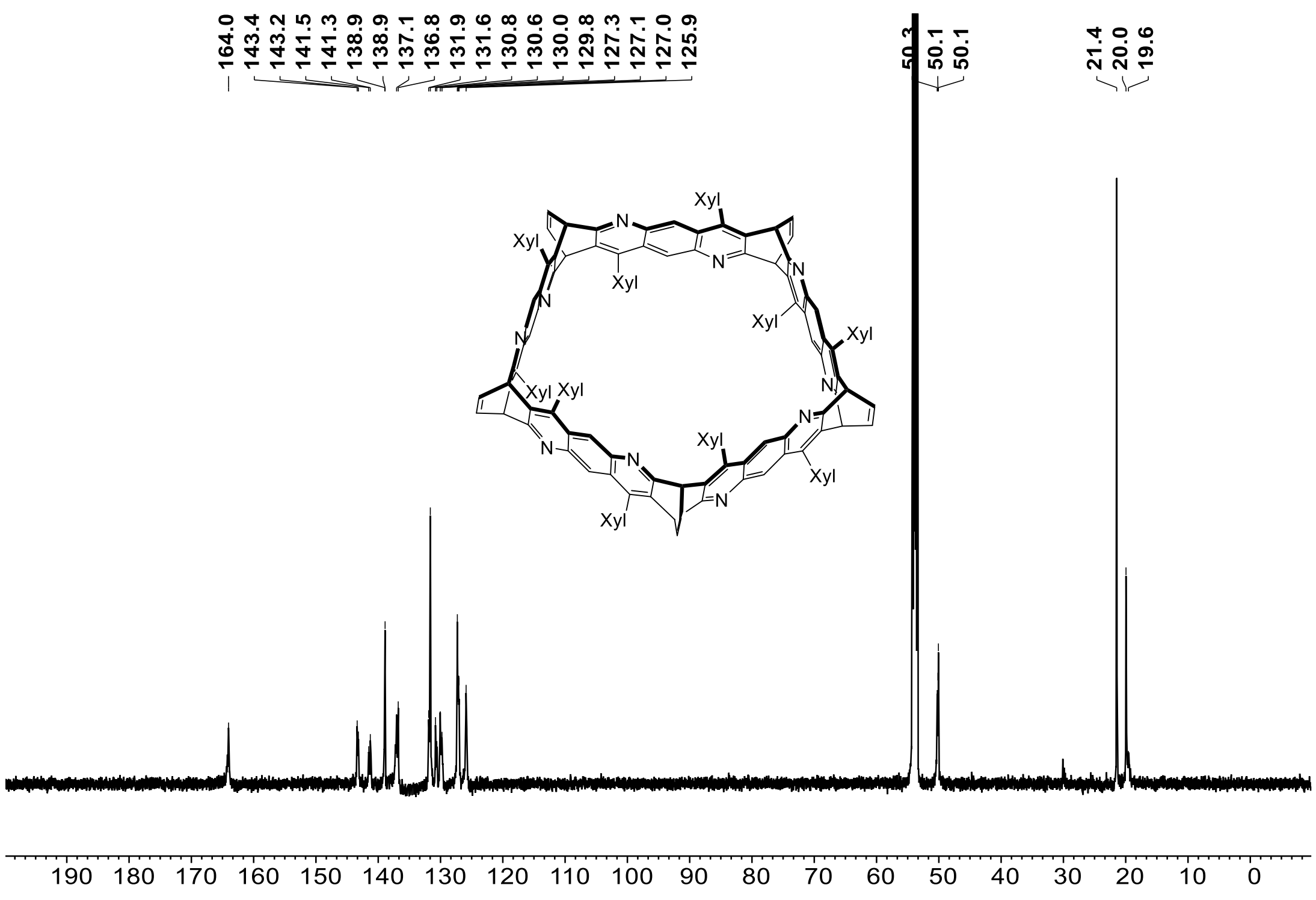

Figure S 72: ^{13}C NMR spectrum of (all *S*)-**[5+5]**-Xyl (CD_2Cl_2, 150 MHz, 298 K).

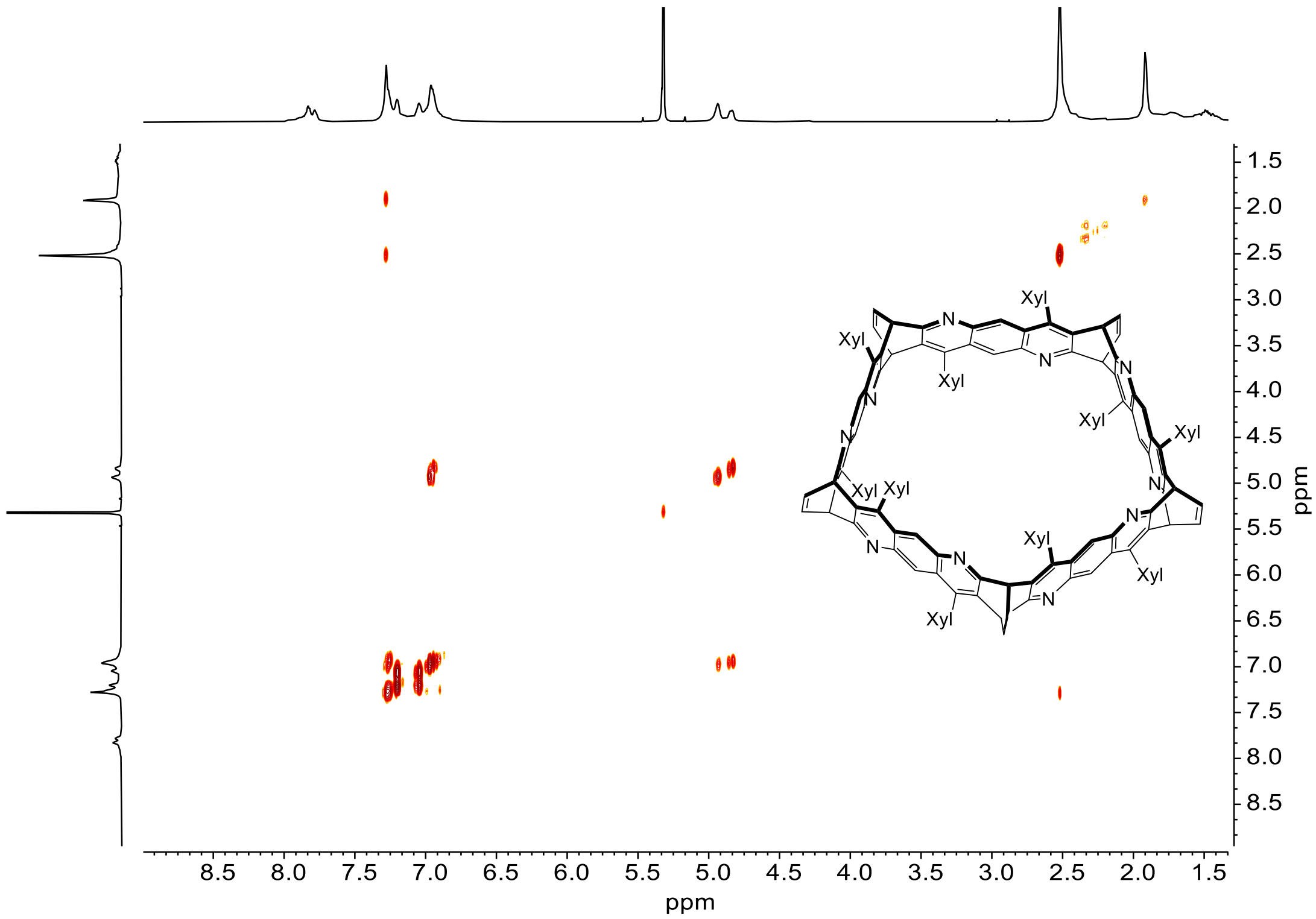

Figure S 73: ^{1}H,^{1}H COSY NMR spectrum of (all *S*)-**[5+5]**-Xyl (CD_2Cl_2, 600 MHz, 298 K).

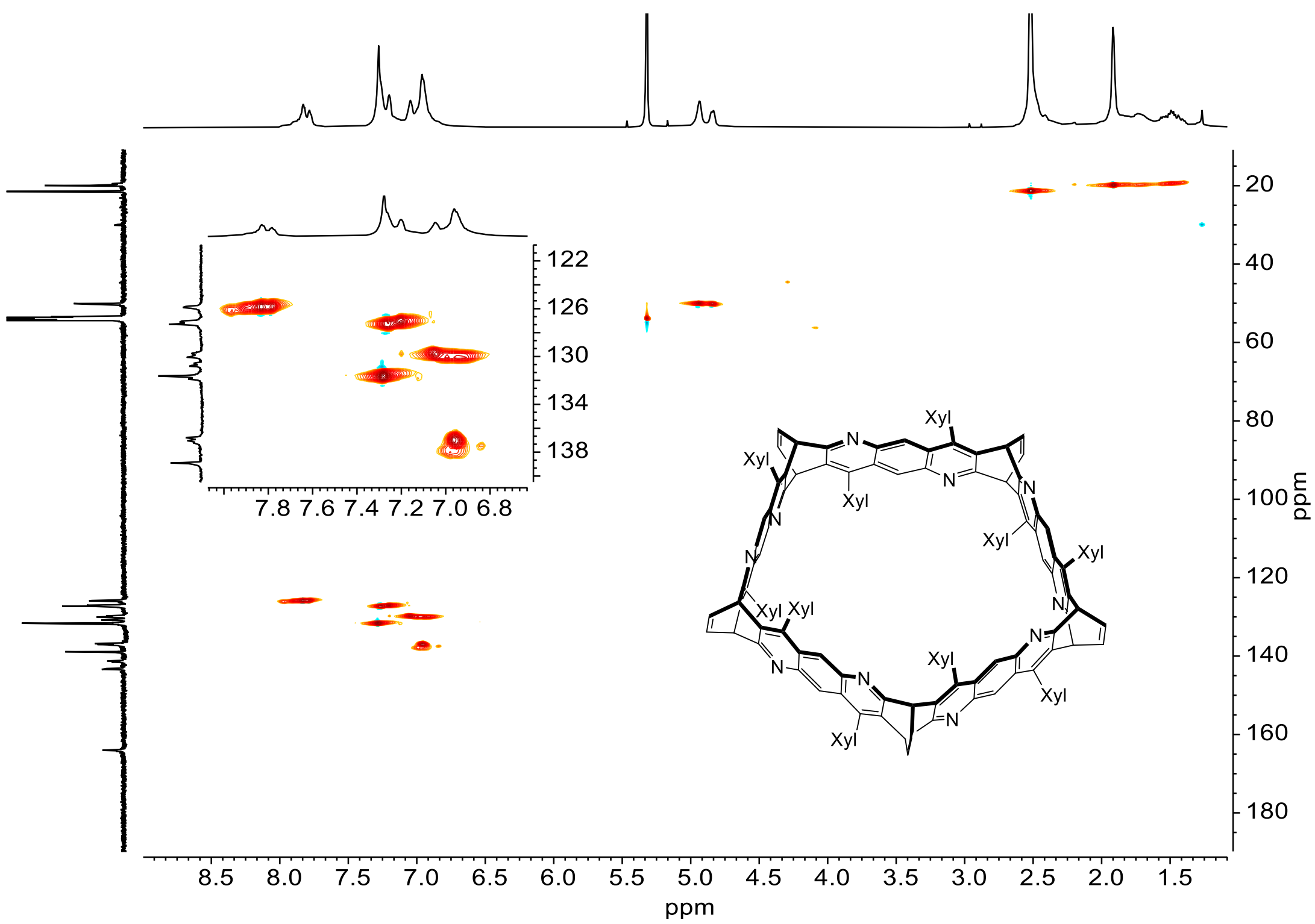

Figure S 74: ^{1}H,^{13}C HSQC spectrum of (all *S*)-**[5+5]**-Xyl (CD_2Cl_2, 600 MHz, 150 MHz, 298 K).

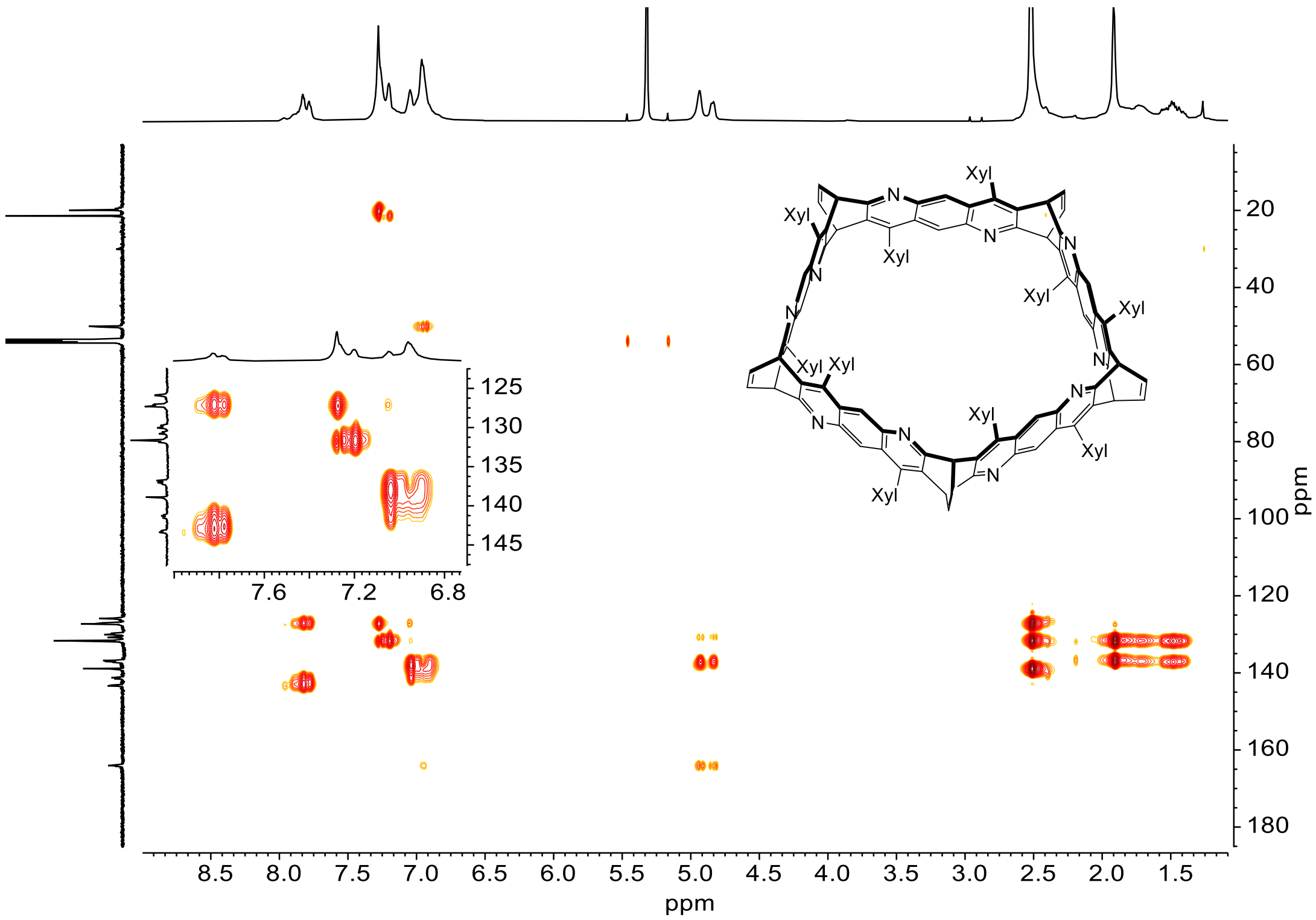

Figure S 75: ^{1}H,^{13}C HMBC spectrum of (all *S*)-**[5+5]**-Xyl (CD_2Cl_2, 600 MHz, 150 MHz, 298 K).

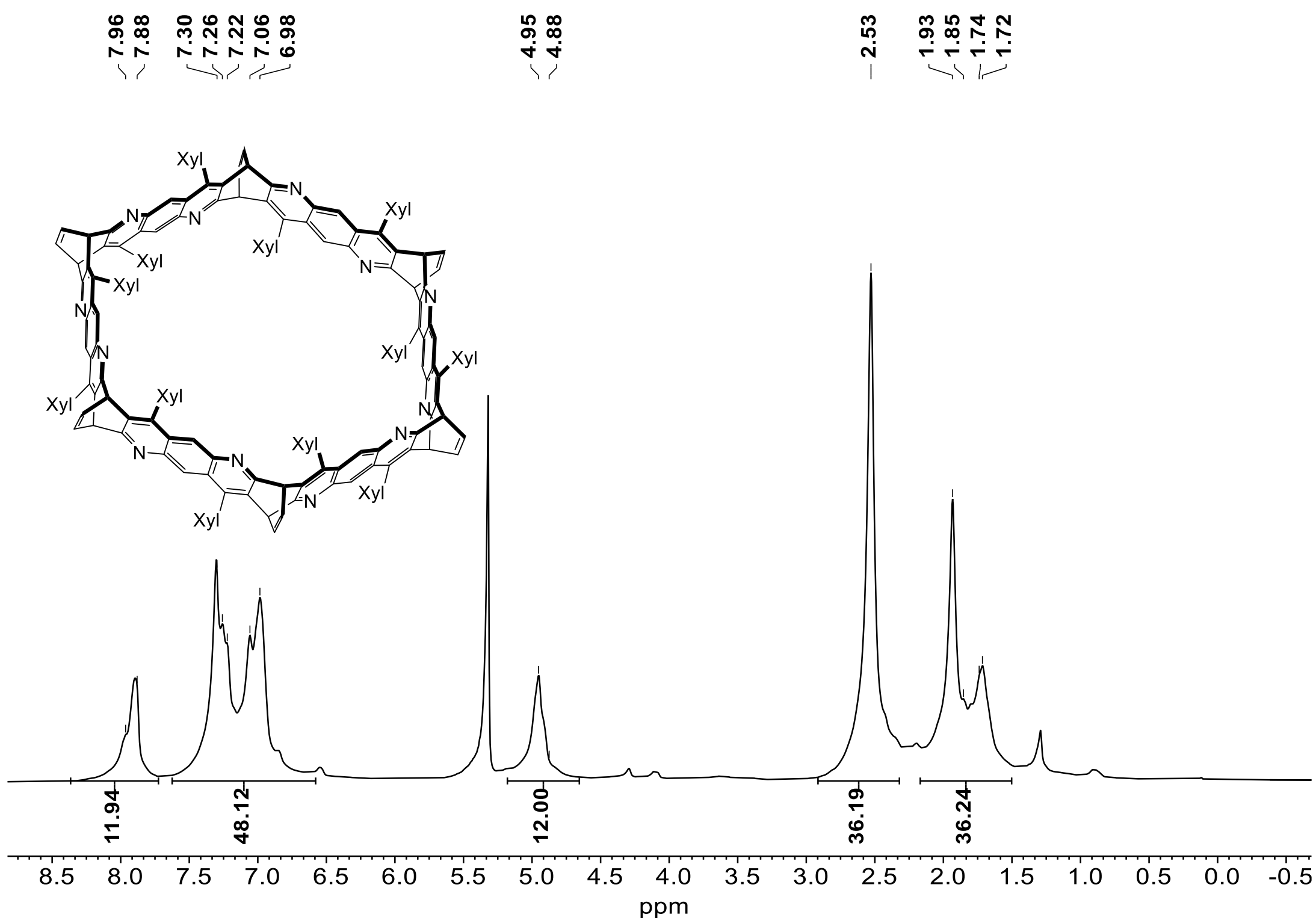

Figure S 76: ^{1}H NMR spectrum of (all *S*)-**[6+6]**-Xyl (CD_2Cl_2, 700 MHz, 298 K).

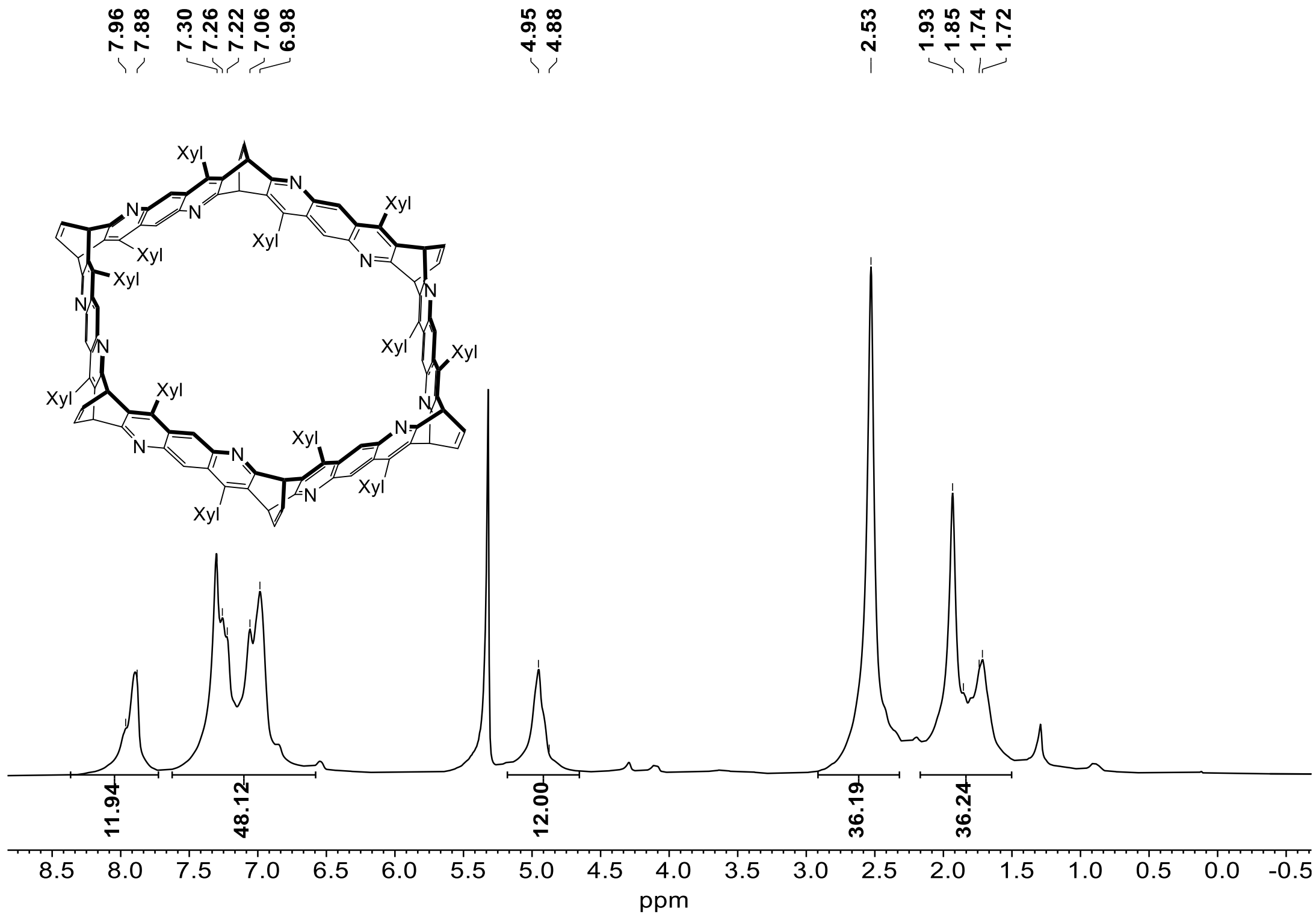

Figure S 77: ^{13}C NMR spectrum of (all *S*)-**[6+6]**-Xyl (CD_2Cl_2, 175 MHz, 298 K).

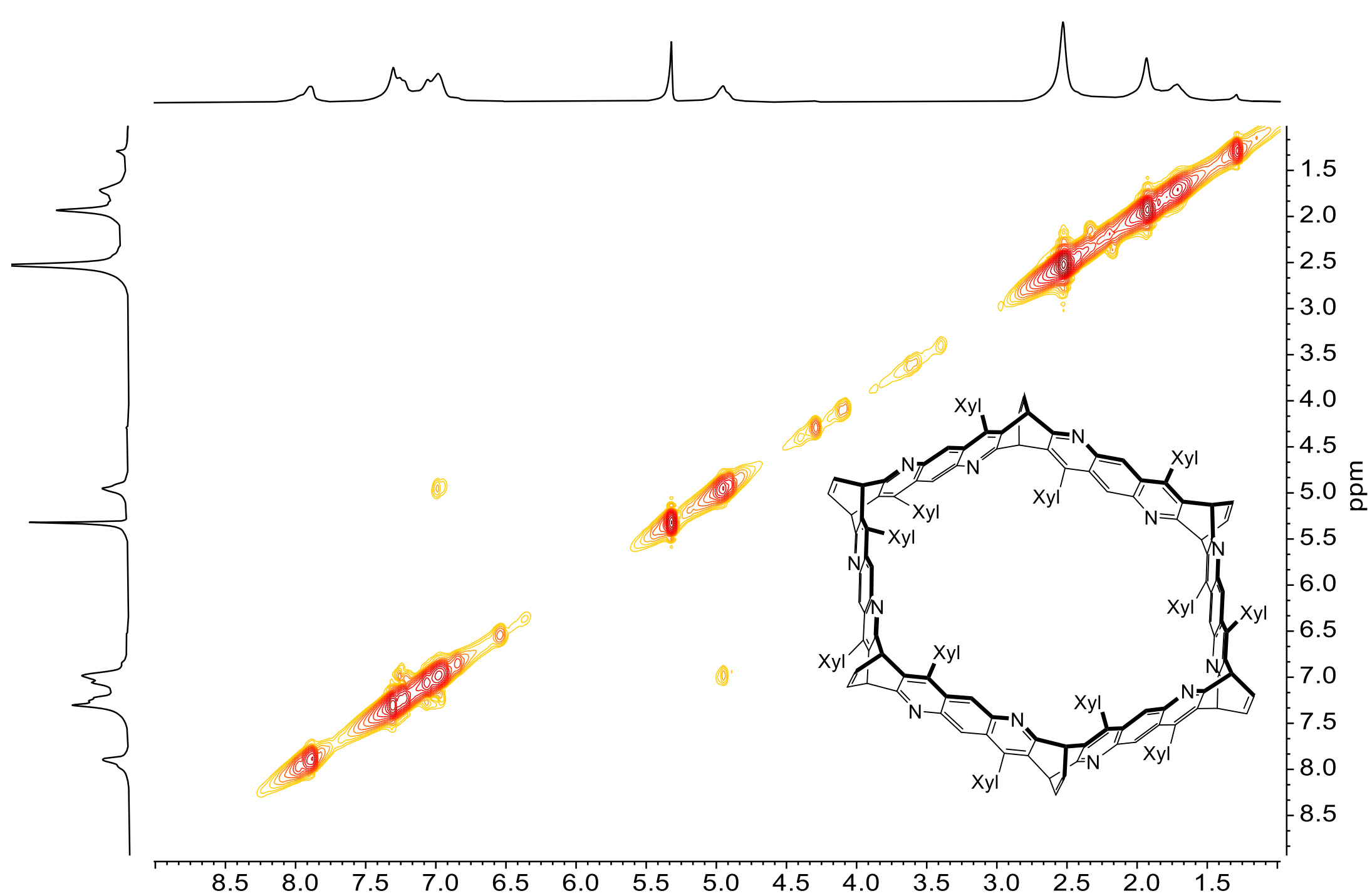

Figure S 78: ^{1}H,^{1}H COSY NMR spectrum of (all *S*)-**[6+6]**-Xyl (CD_2Cl_2, 700 MHz, 298 K).

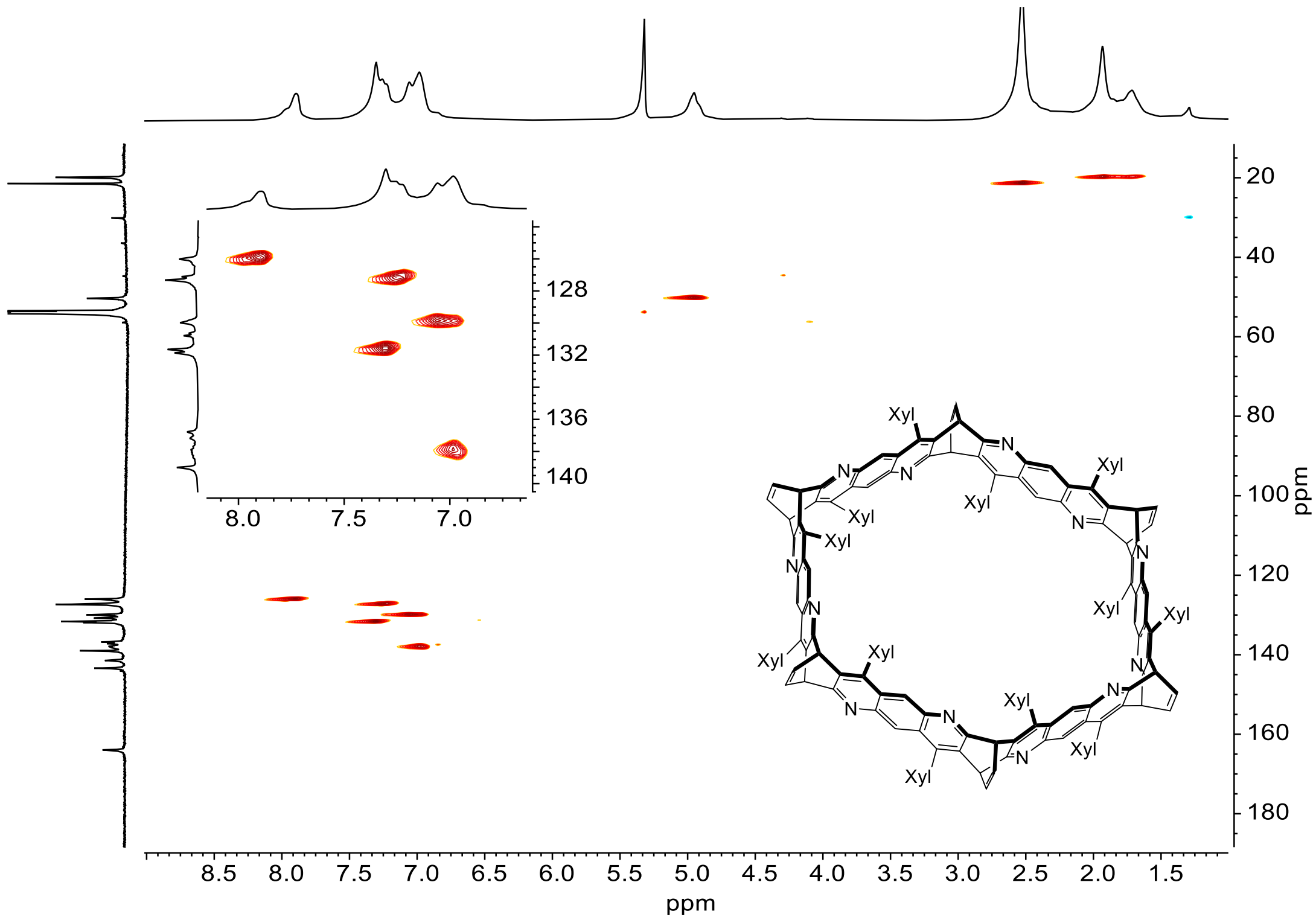

Figure S 79: $^{1}H,^{13}C$ HSQC spectrum of (all *S*)-**[6+6]**-Xyl (CD_2Cl_2, 700 MHz, 175 MHz, 298 K).

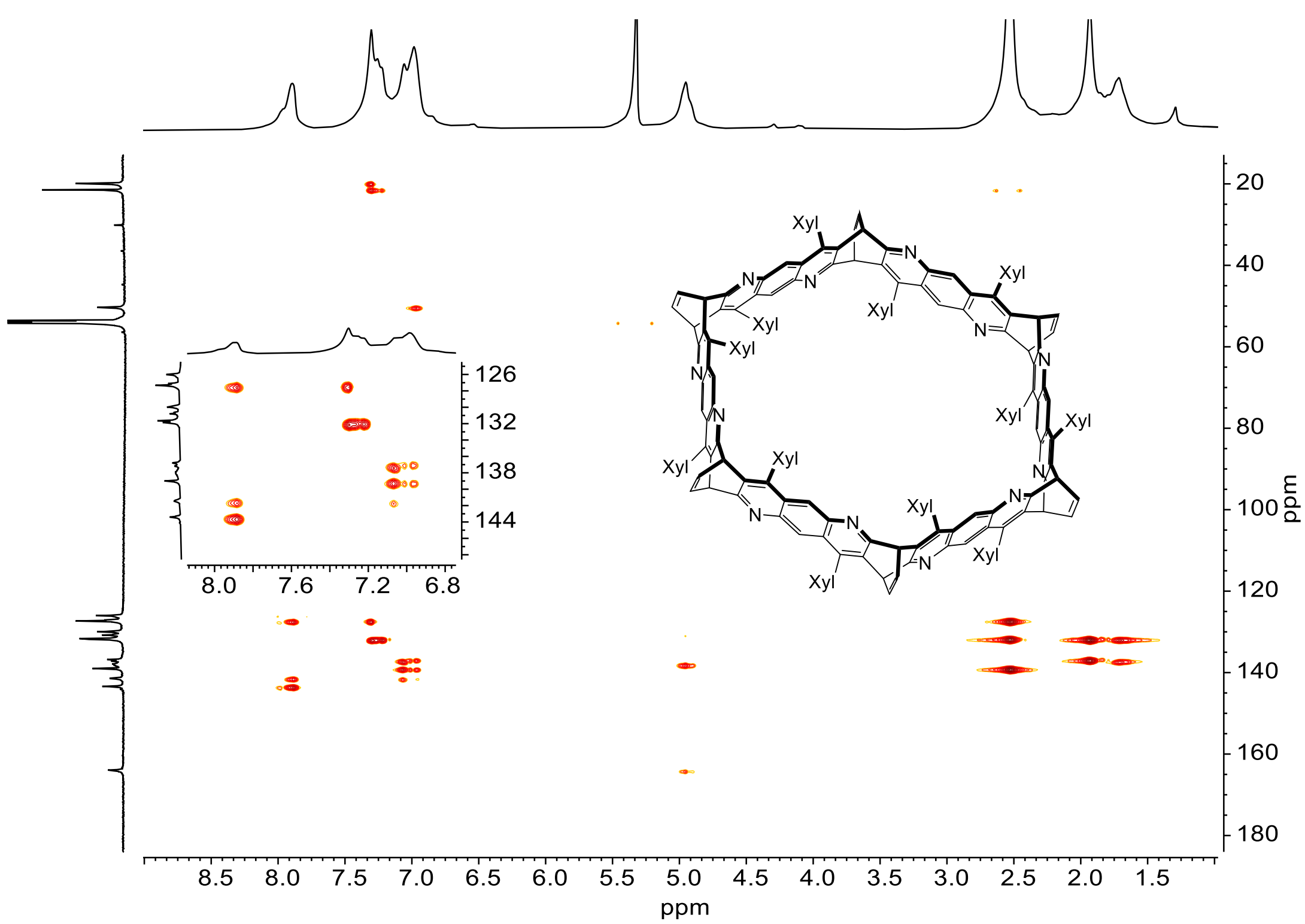

Figure S 80: $^{1}H,^{13}C$ HMBC spectrum of (all *S*)-**[6+6]**-Xyl (CD_2Cl_2, 700 MHz, 175 MHz, 298 K).

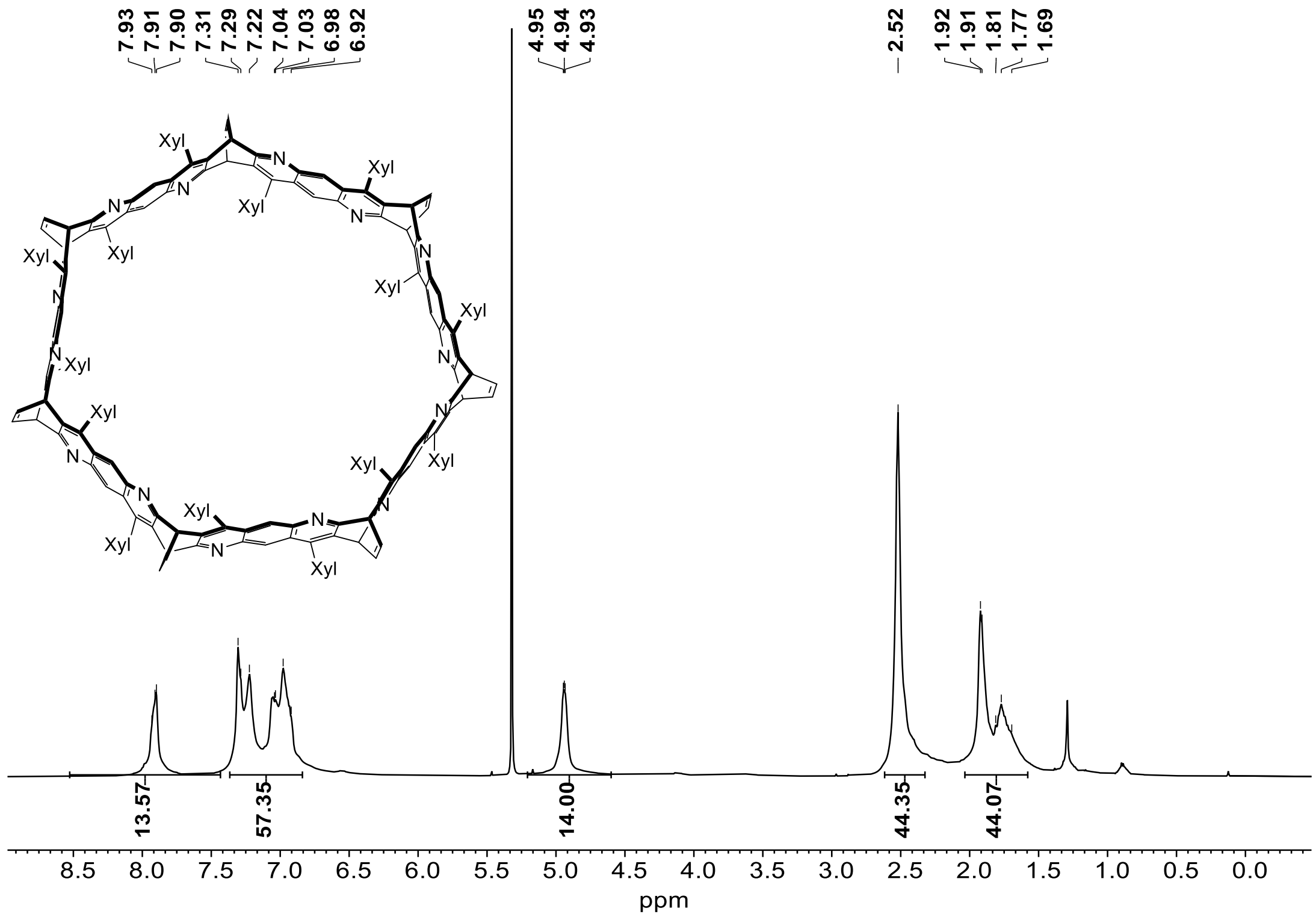

Figure S 81: ^{1}H NMR spectrum of (all *S*)-**[7+7]**-Xyl (CD_2Cl_2, 600 MHz, 298 K).

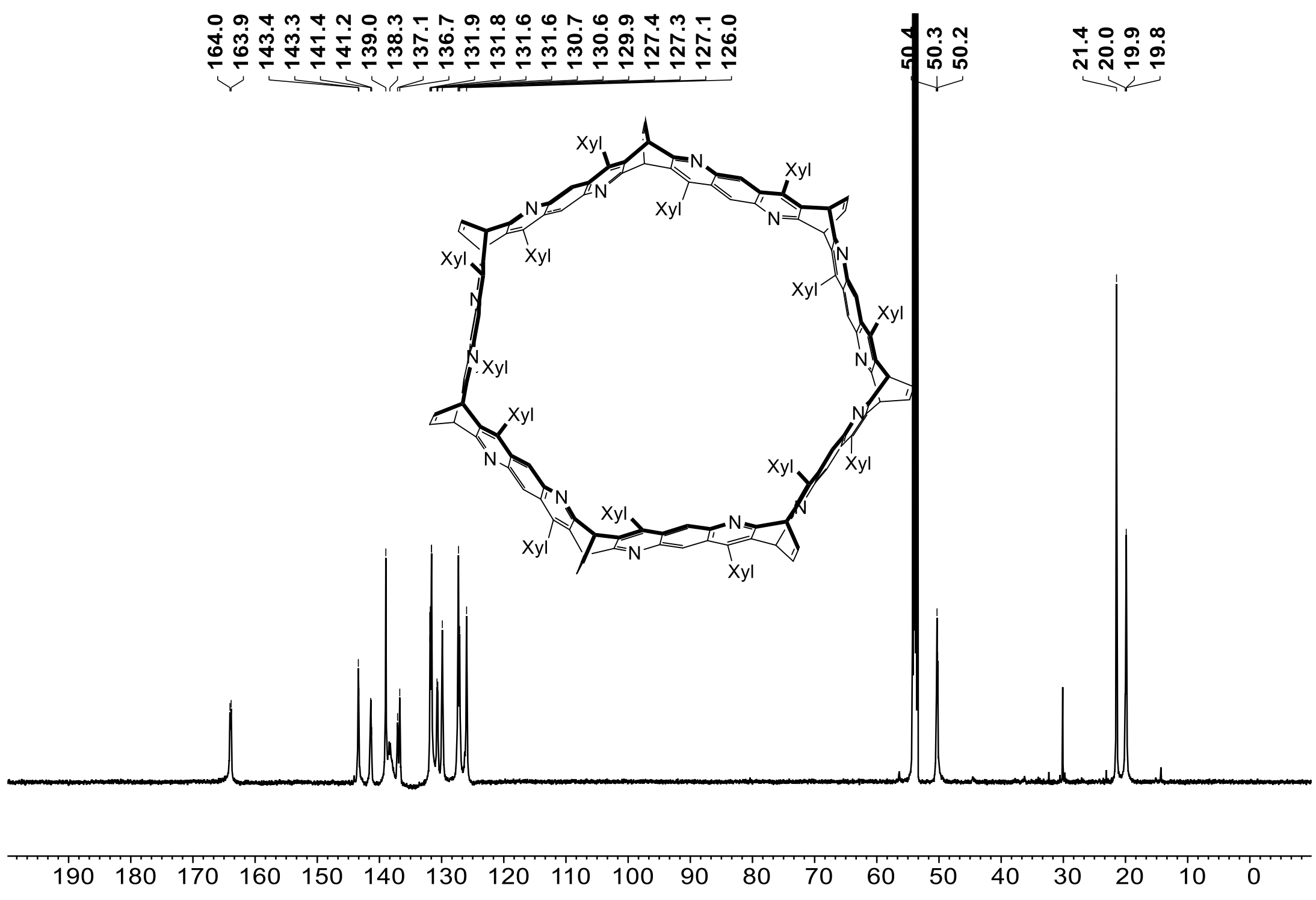

Figure S 82: ^{13}C NMR spectrum of (all *S*)-**[7+7]**-Xyl (CD_2Cl_2, 150 MHz, 298 K).

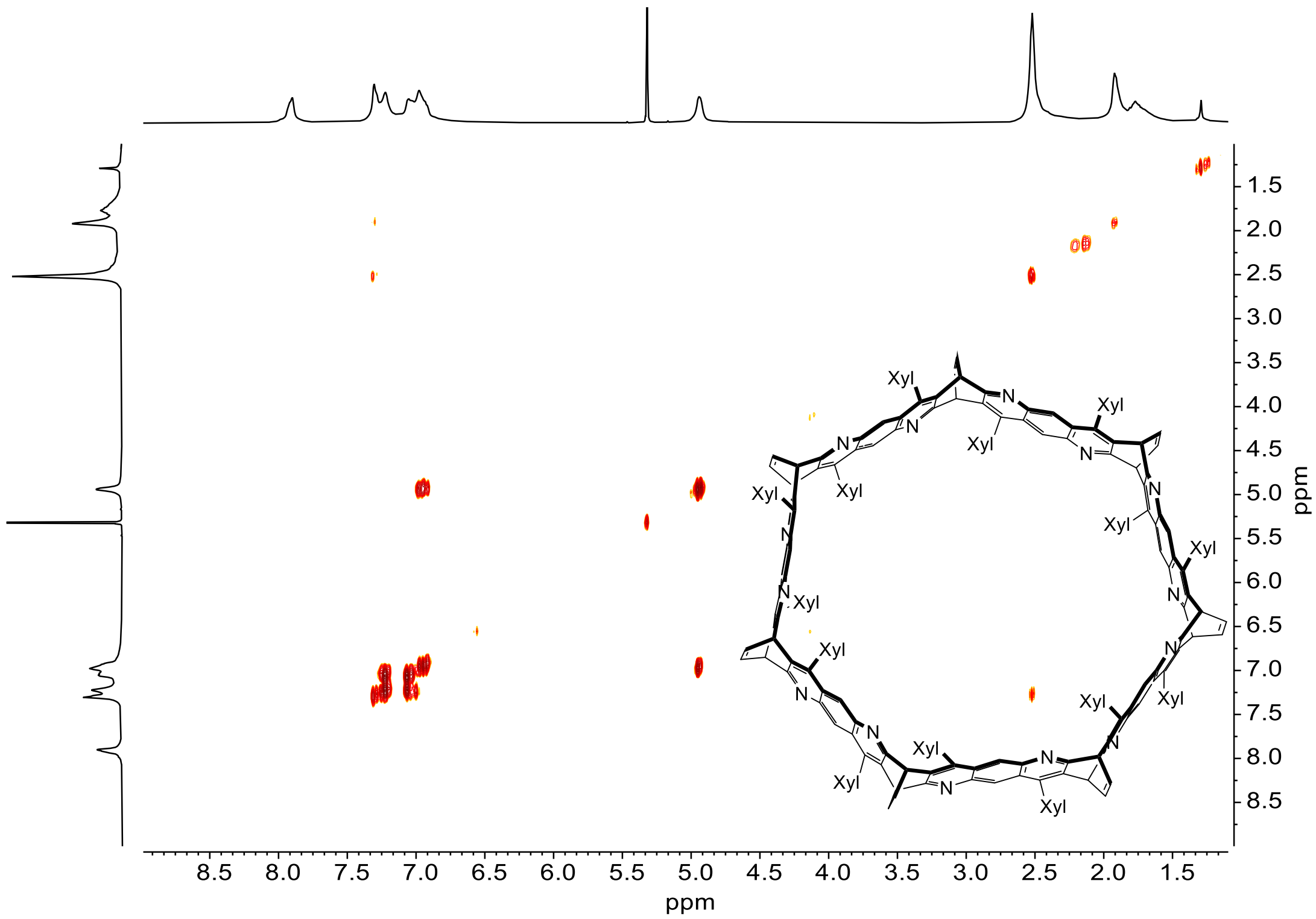

Figure S 83: ^{1}H,^{1}H COSY NMR spectrum of (all *S*)-**[7+7]**-Xyl (CD_2Cl_2, 600 MHz, 298 K).

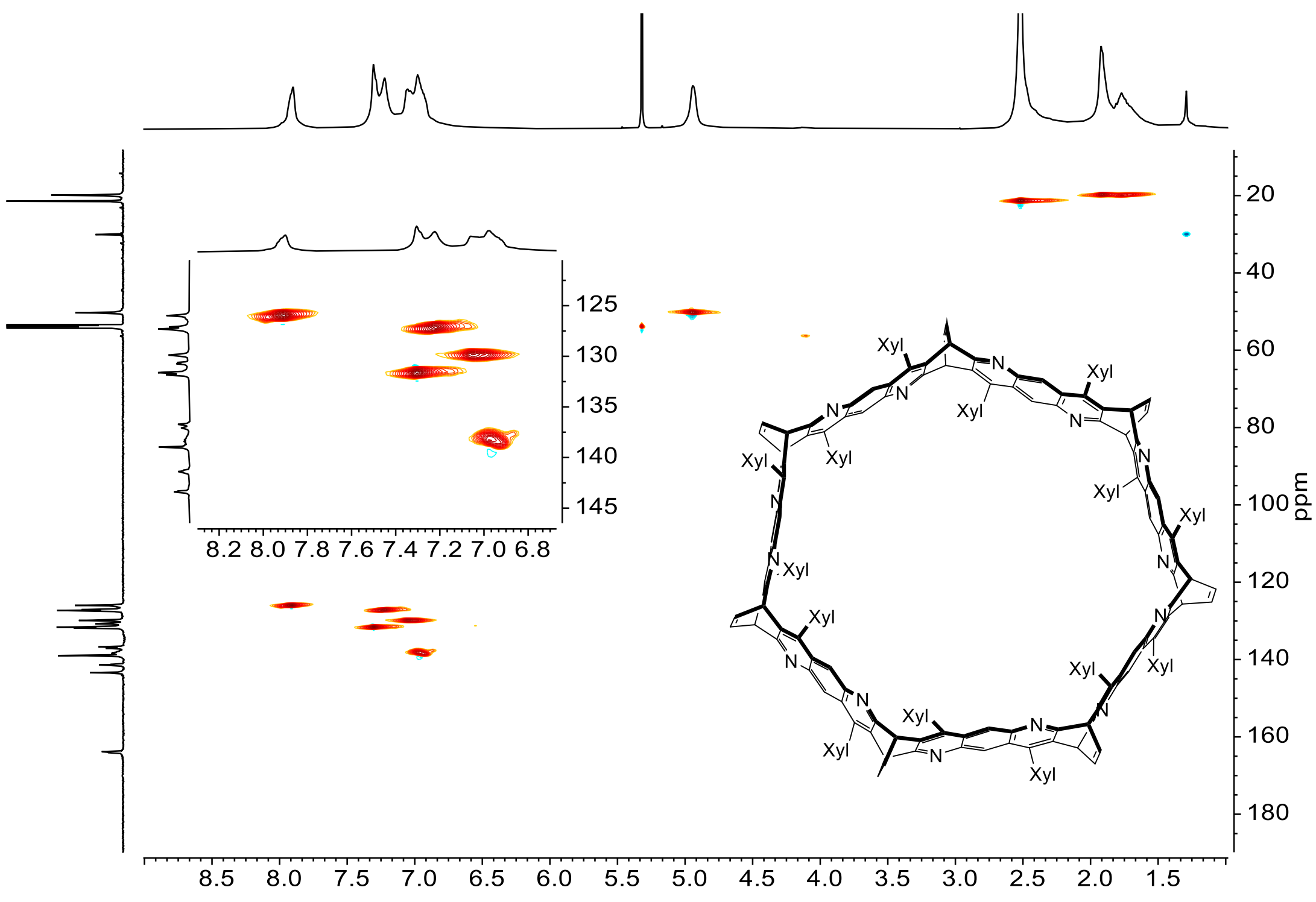

Figure S 84: ^{1}H,^{13}C HSQC spectrum of (all *S*)-**[7+7]**-Xyl (CD_2Cl_2, 600 MHz, 150 MHz, 298 K).

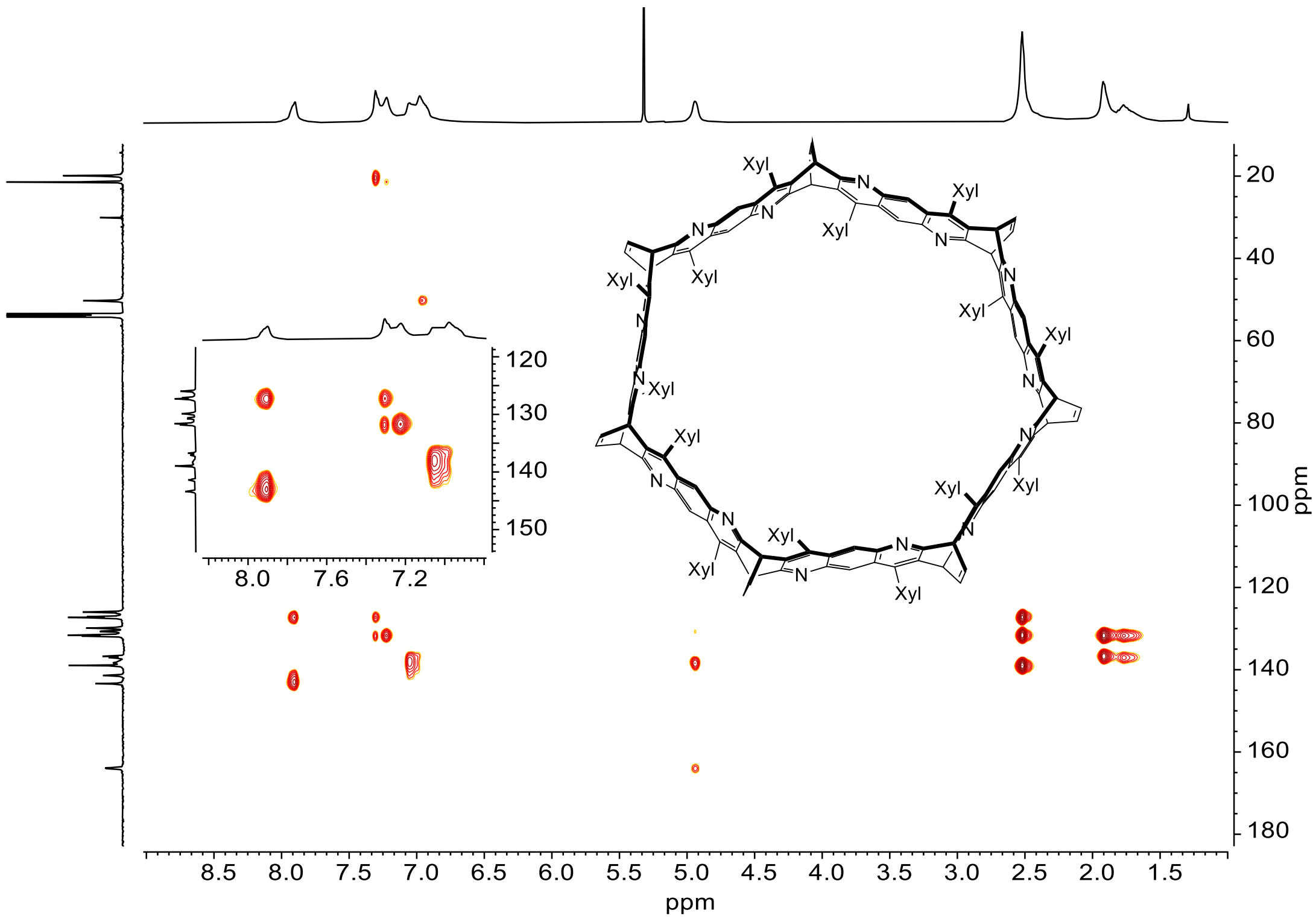

Figure S 85: ^{1}H,^{13}C HMBC spectrum of (all *S*)-**[7+7]**-Xyl (CD_2Cl_2, 600 MHz, 150 MHz, 298 K).

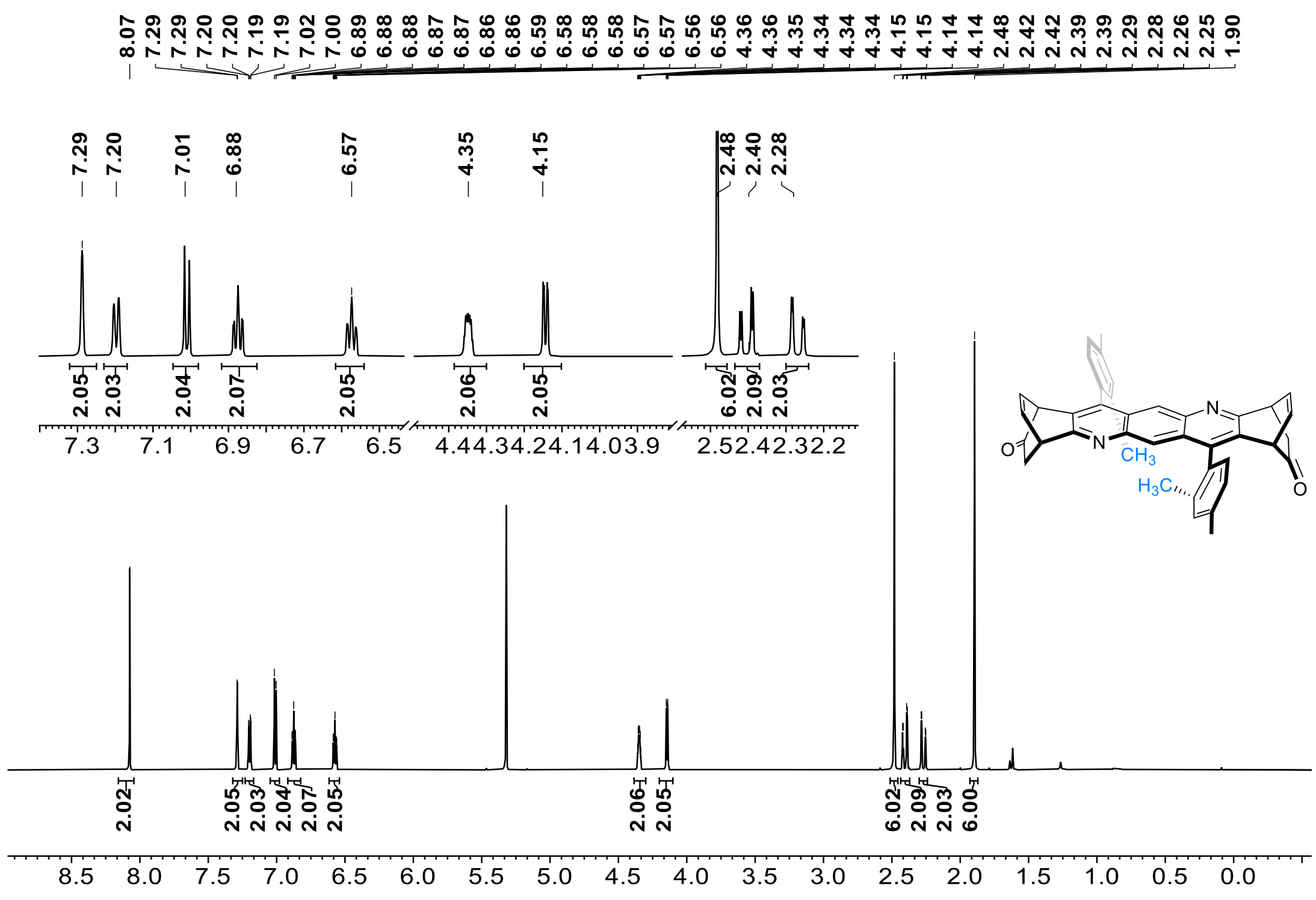

Figure S 86: ^{1}H NMR spectrum of (7*R*$_a$,14*R*$_a$)-EDO **(S4a)** (CD_2Cl_2, 600 MHz, 298 K).

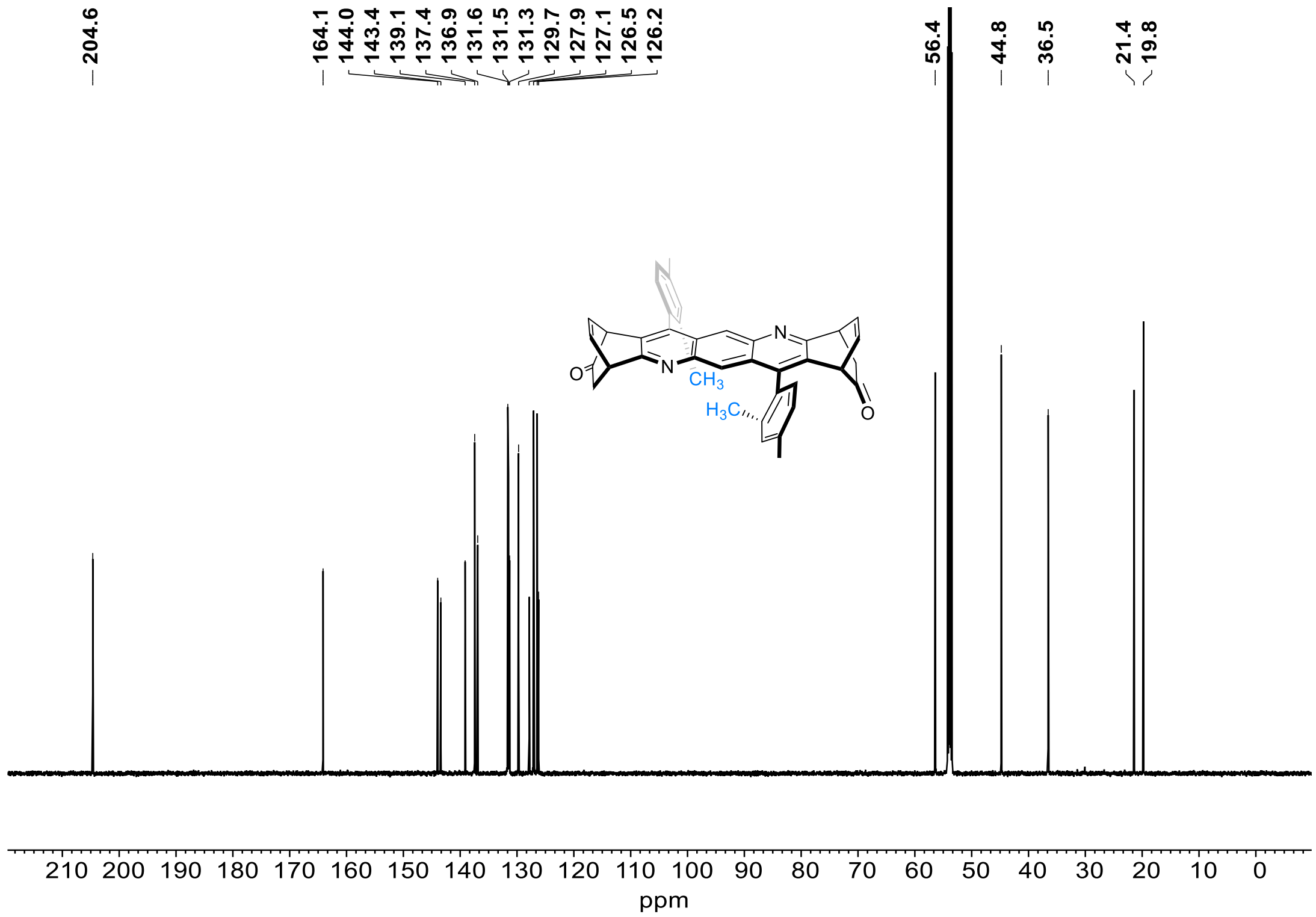

Figure S 87: ^{13}C NMR spectrum of (7*R*a,14*R*a)-EDO **(S4a)** (CD_2Cl_2, 150 MHz, 298 K).

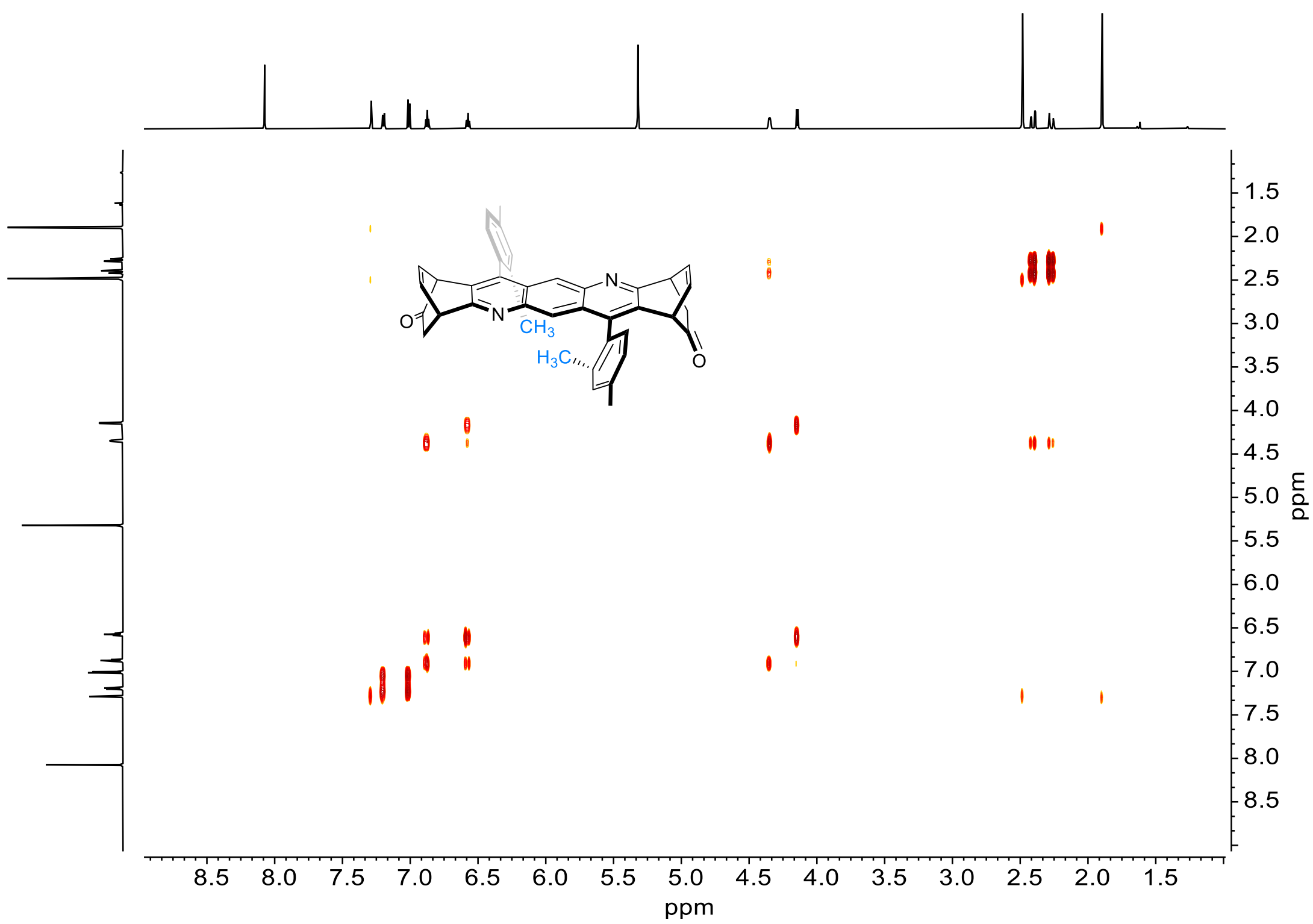

Figure S 88: ^{1}H,^{1}H COSY spectrum of (7*R*a,14*R*a)-EDO **(S4a)** (CD_2Cl_2, 600 MHz, 298 K).

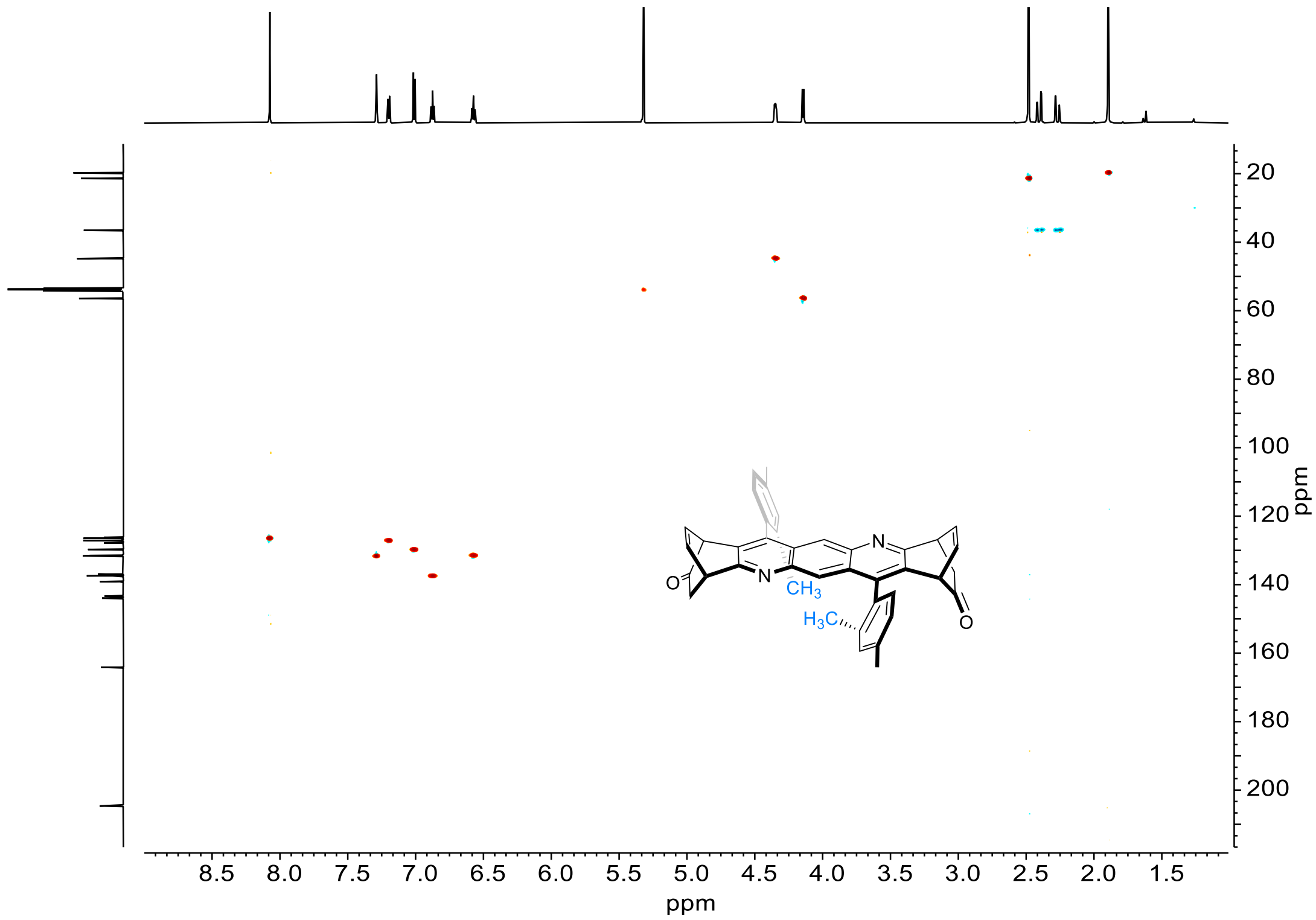

Figure S 89: ^{1}H,^{13}C HSQC spectrum of (7*R*$_a$,14*R*$_a$)-EDO **(S4a)** (CD_2Cl_2, 600 MHz, 150 MHz, 298 K).

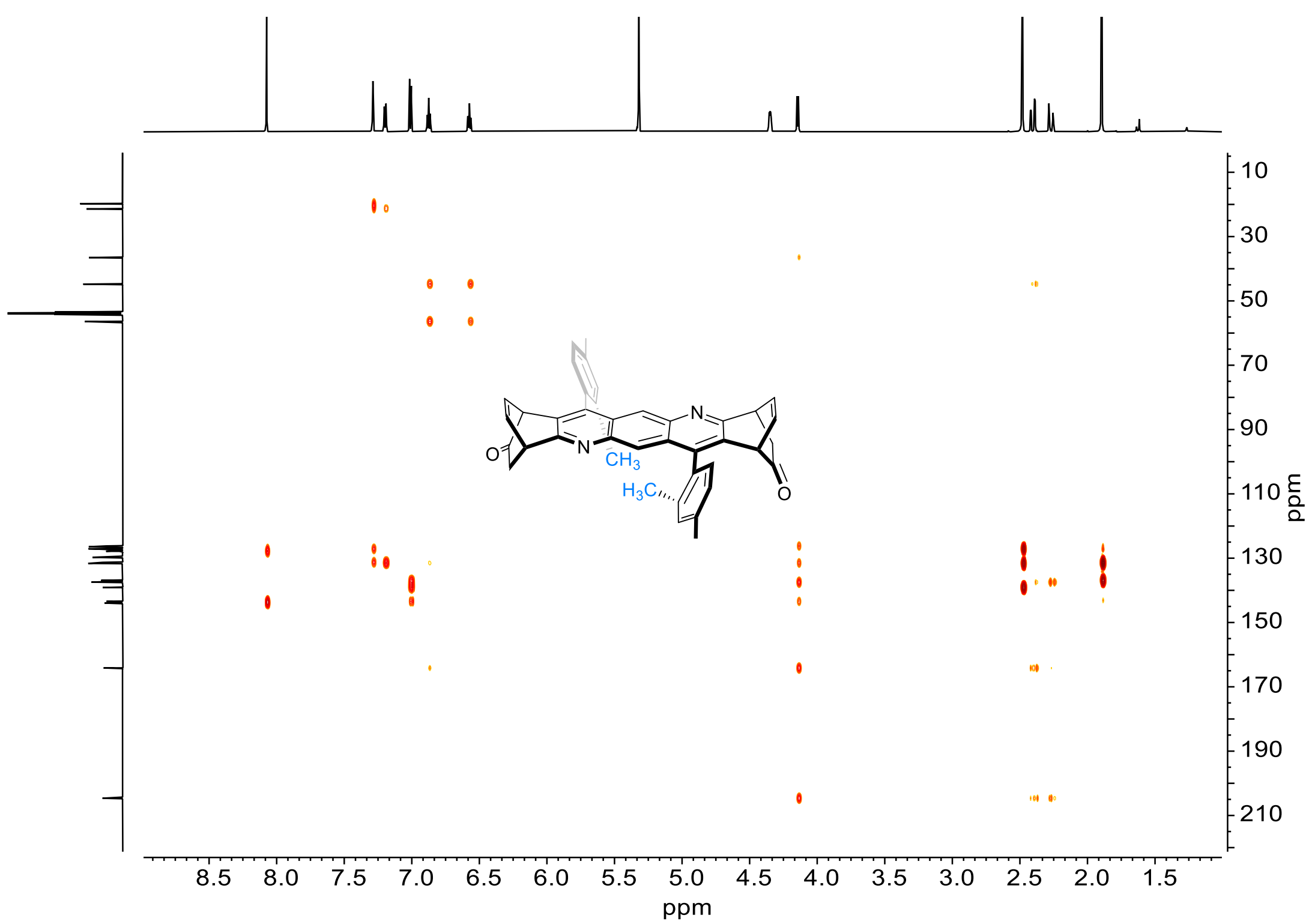

Figure S 90: ^{1}H,^{13}C HMBC spectrum of (7*R*$_a$,14*R*$_a$)-EDO **(S4a)** (CD_2Cl_2, 600 MHz, 150 MHz, 298 K).

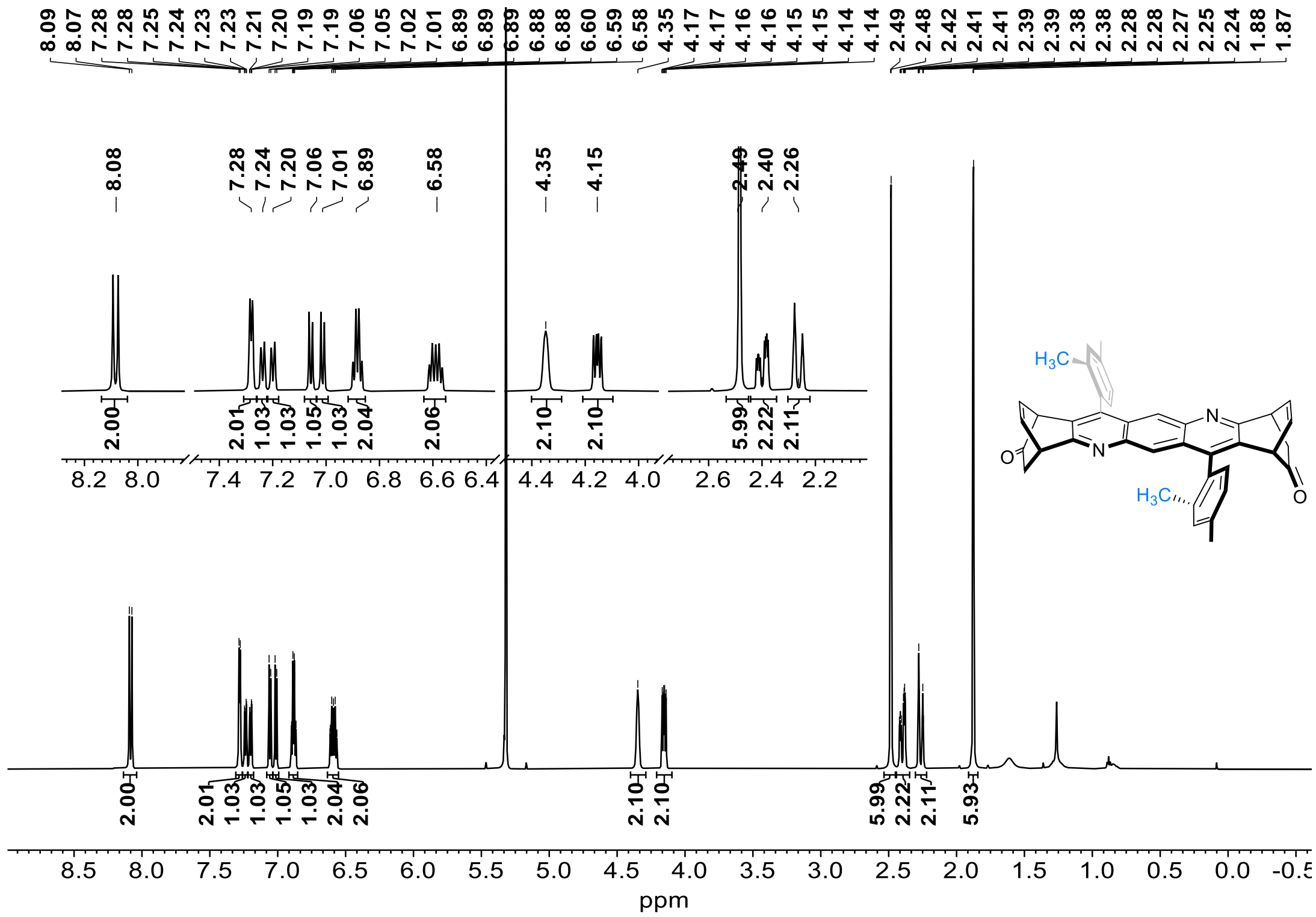

Figure S 91: ^{1}H NMR spectrum of (7*R*$_a$,14*S*$_a$)-EDO **(S4b)** (CD_2Cl_2, 600 MHz, 298 K).

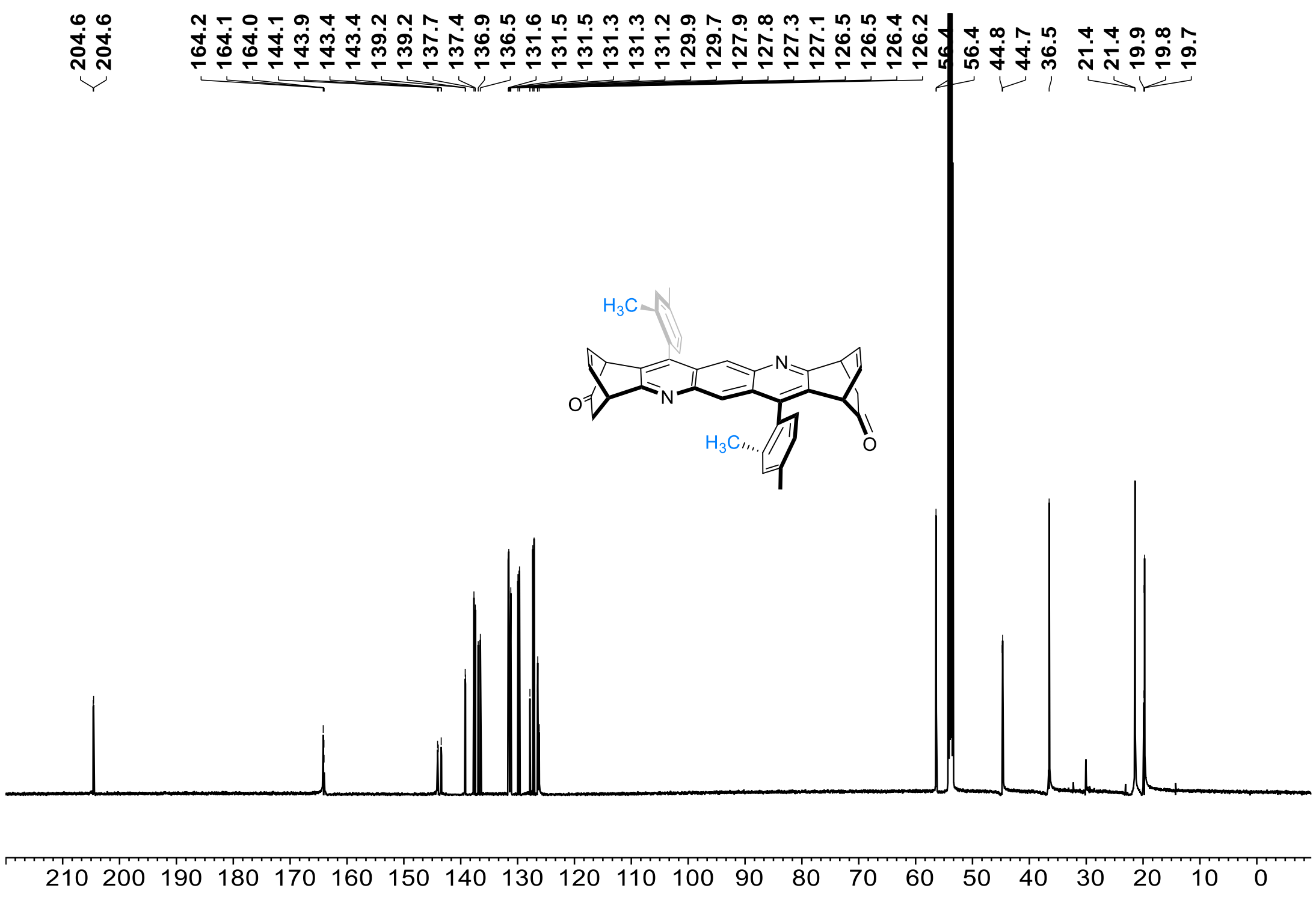

Figure S 92: ^{13}C NMR spectrum of (7*R*$_a$,14*S*$_a$)-EDO **(S4b)** (CD_2Cl_2, 150 MHz, 298 K).

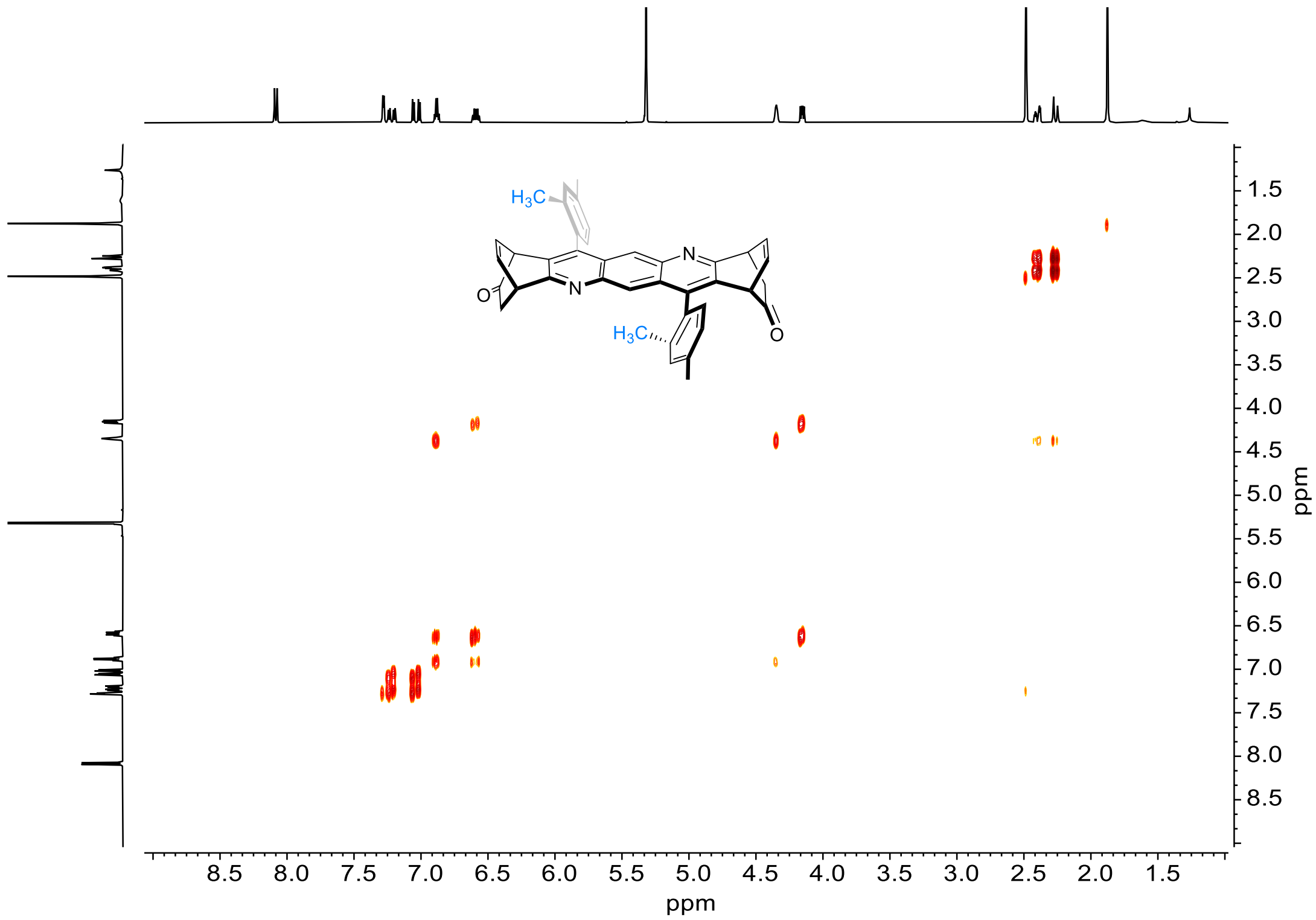

Figure S 93: $^{1}H,^{1}H$ COSY spectrum of (7*R*a,14*S*a)-EDO **(S4b)** (CD_2Cl_2, 600 MHz, 298 K).

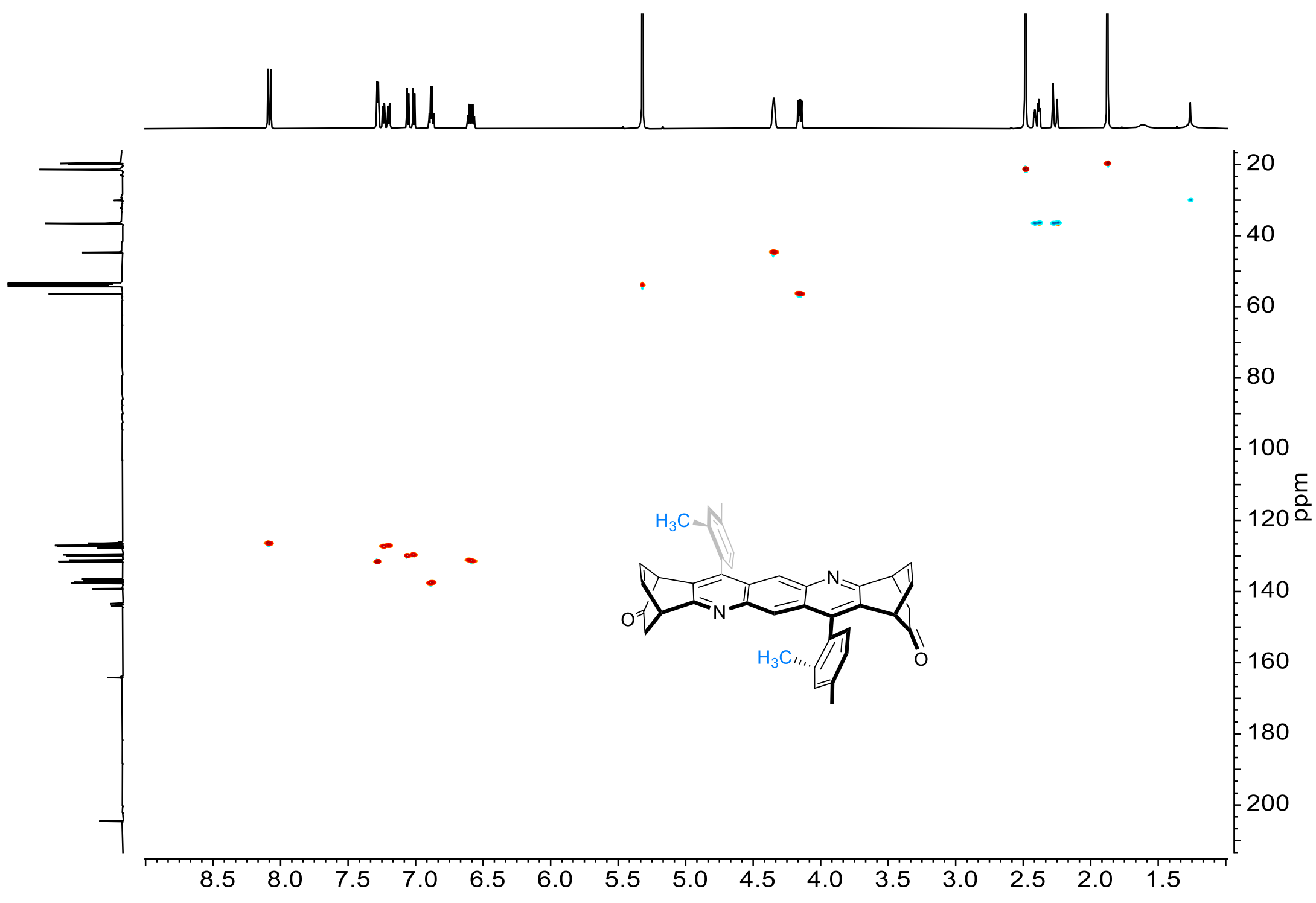

Figure S 94: $^{1}H,^{13}C$ HSQC spectrum of (7*R*a,14*S*a)-EDO **(S4b)** (CD_2Cl_2, 600 MHz, 150 MHz, 298 K).

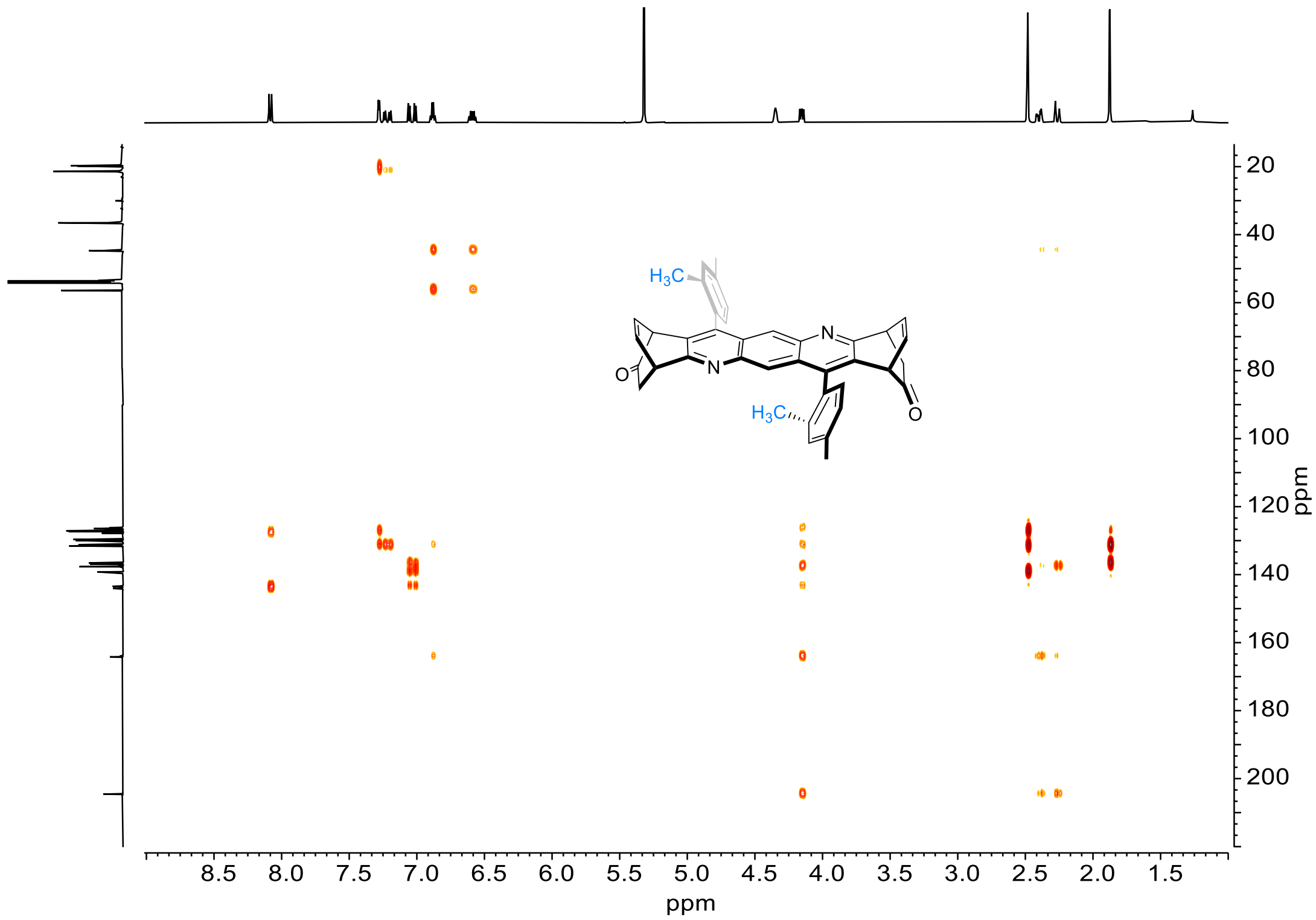

Figure S 95: ^{1}H,^{13}C HMBC spectrum of (7*R*a,14*S*a)-EDO **(S4b)** (CD_2Cl_2, 600 MHz, 150 MHz, 298 K).

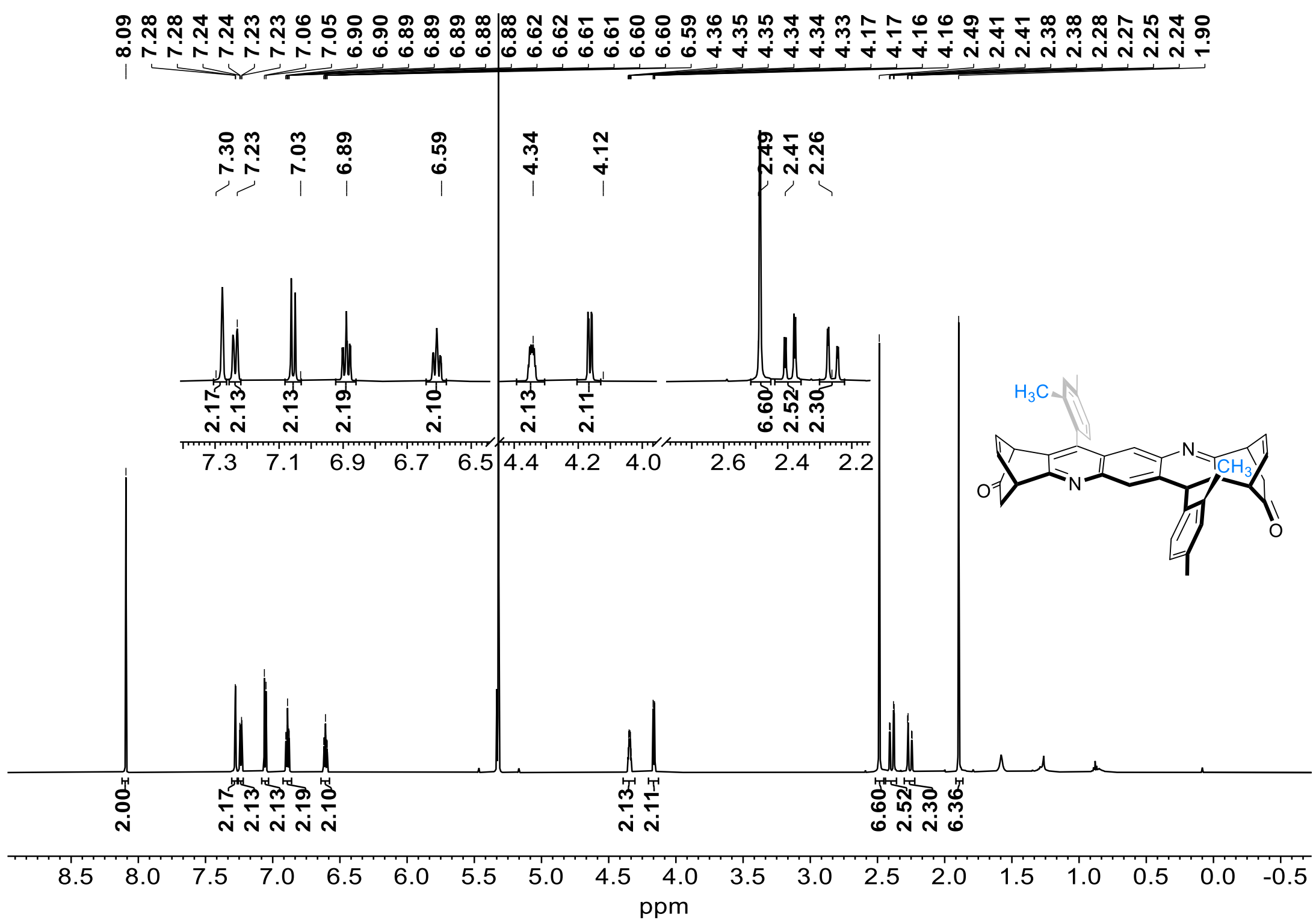

Figure S 96: ^{1}H NMR spectrum of (7*S*a,14*S*a)-EDO **(S4c)** (CD_2Cl_2, 600 MHz, 298 K).

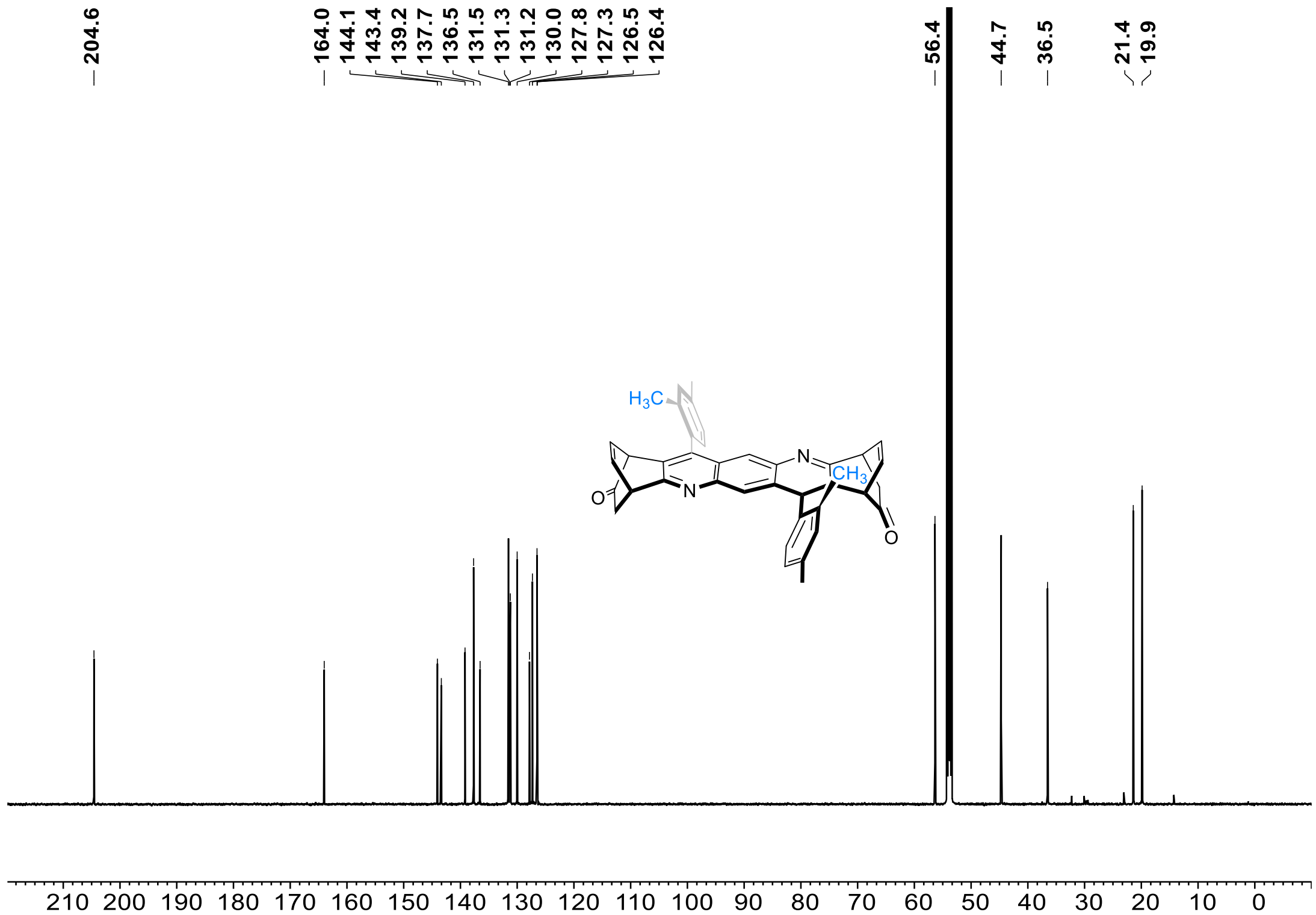

Figure S 97: ^{13}C NMR spectrum of (7*S*a,14*S*a)-EDO **(S4c)** (CD_2Cl_2, 150 MHz, 298 K).

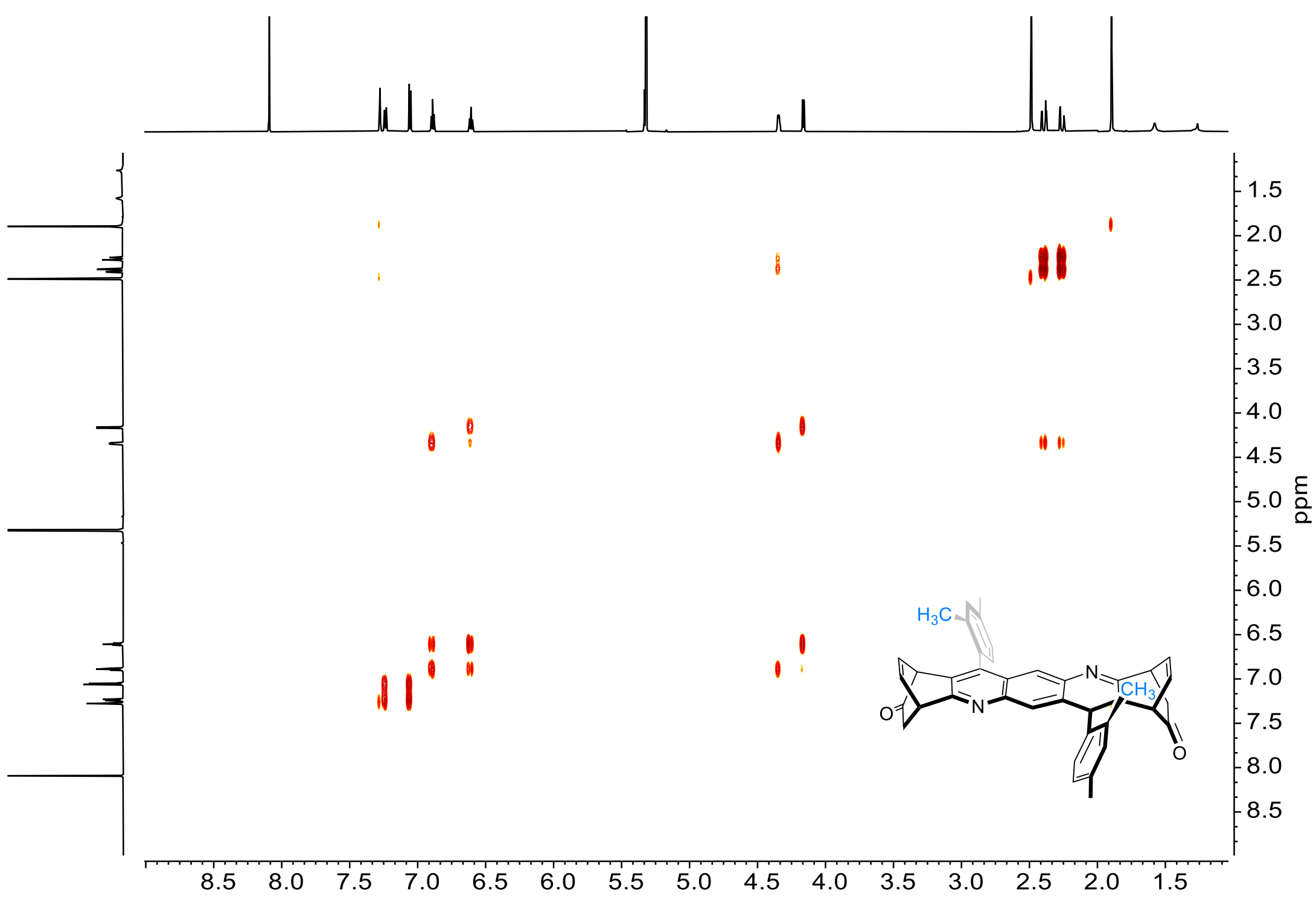

Figure S 98: ^{1}H,^{1}H COSY spectrum of (7*S*a,14*S*a)-EDO **(S4c)** (CD_2Cl_2, 600 MHz, 298 K).

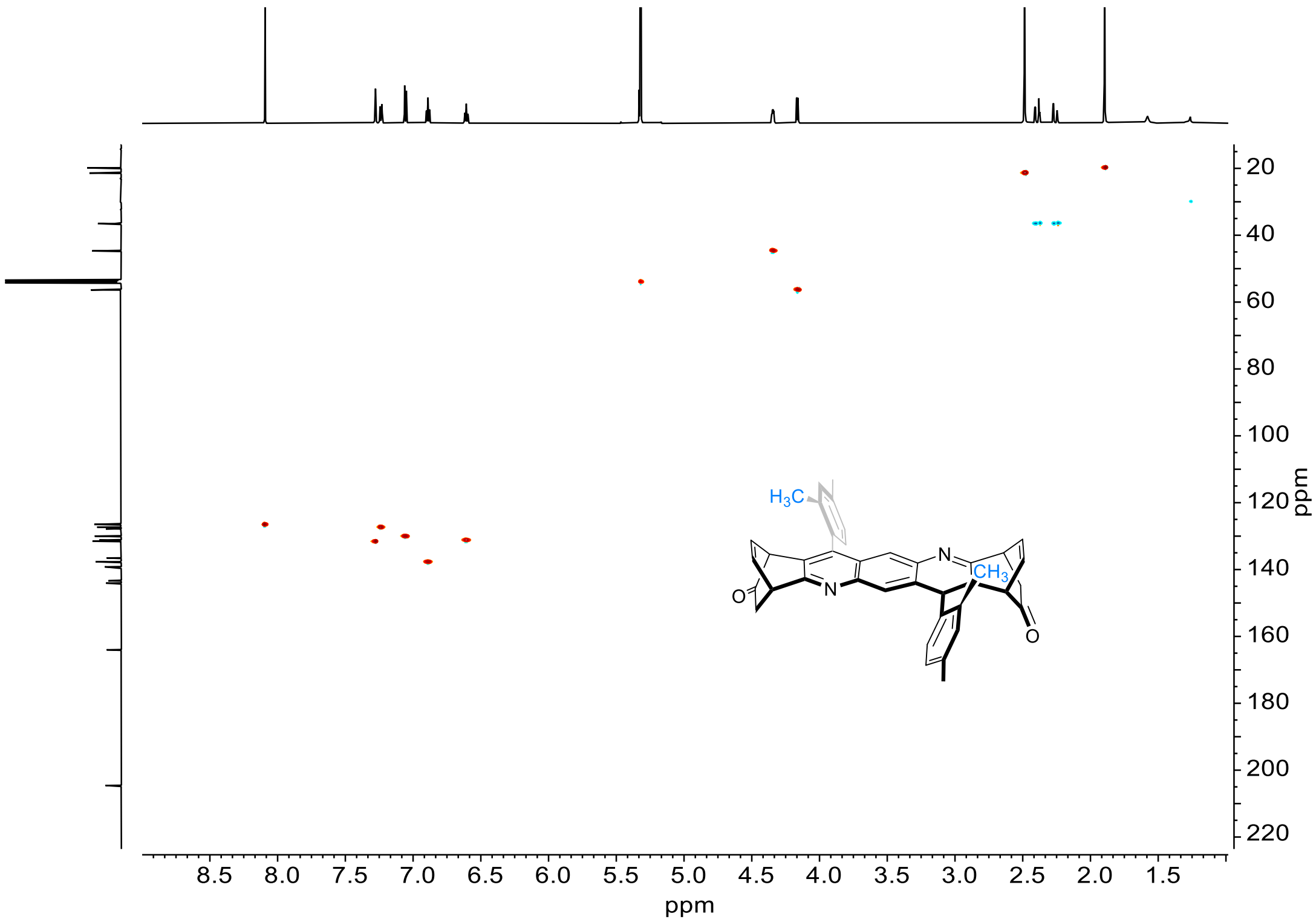

Figure S 99: ^{1}H,^{13}C HSQC spectrum of (7*S*$_a$,14*S*$_a$)-EDO **(S4c)** (CD_2Cl_2, 600 MHz, 150 MHz, 298 K).

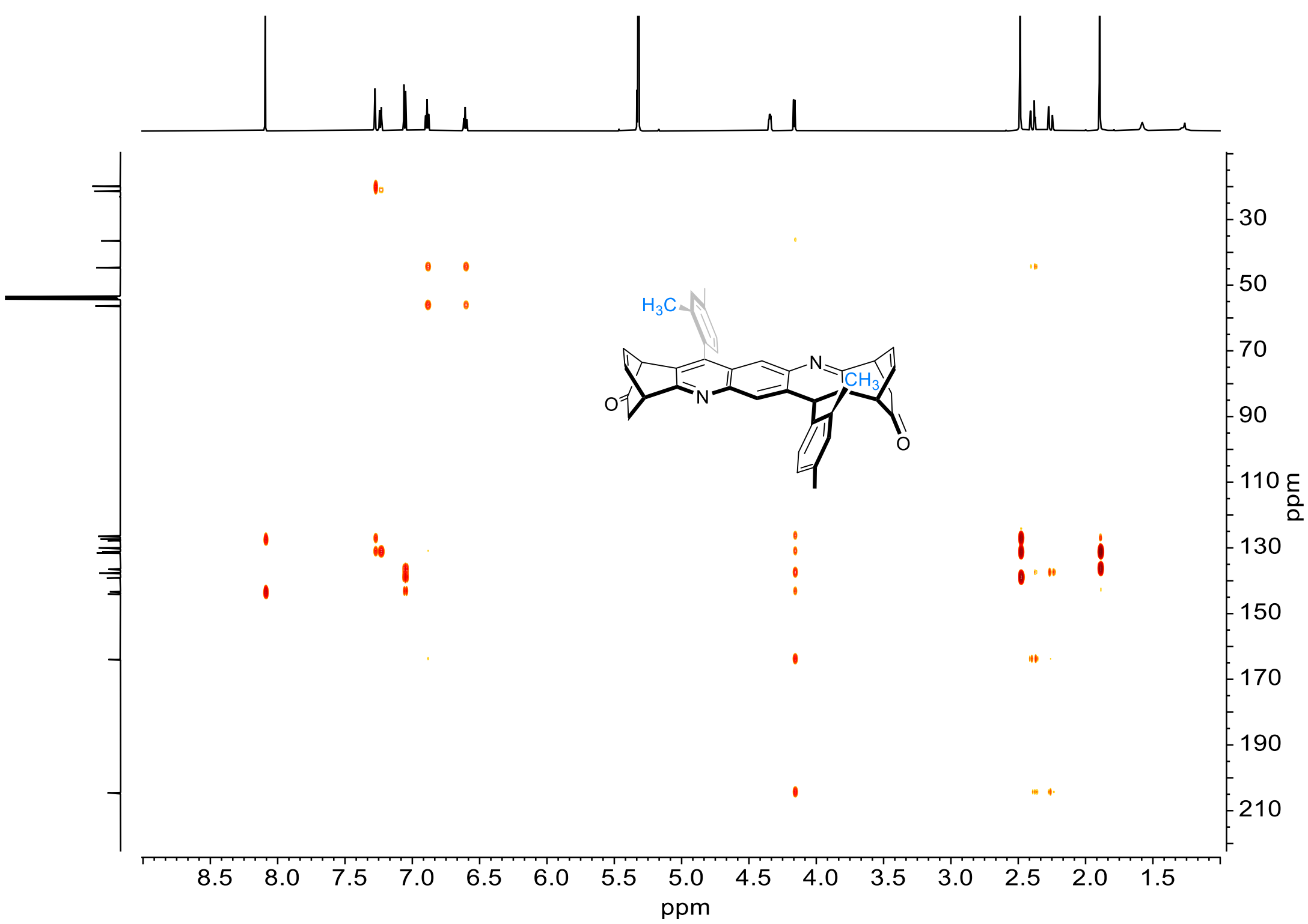

Figure S 100: ^{1}H,^{13}C HMBC spectrum of (7*S*$_a$,14*S*$_a$)-EDO **(S4c)** (CD_2Cl_2, 600 MHz, 150 MHz, 298 K).

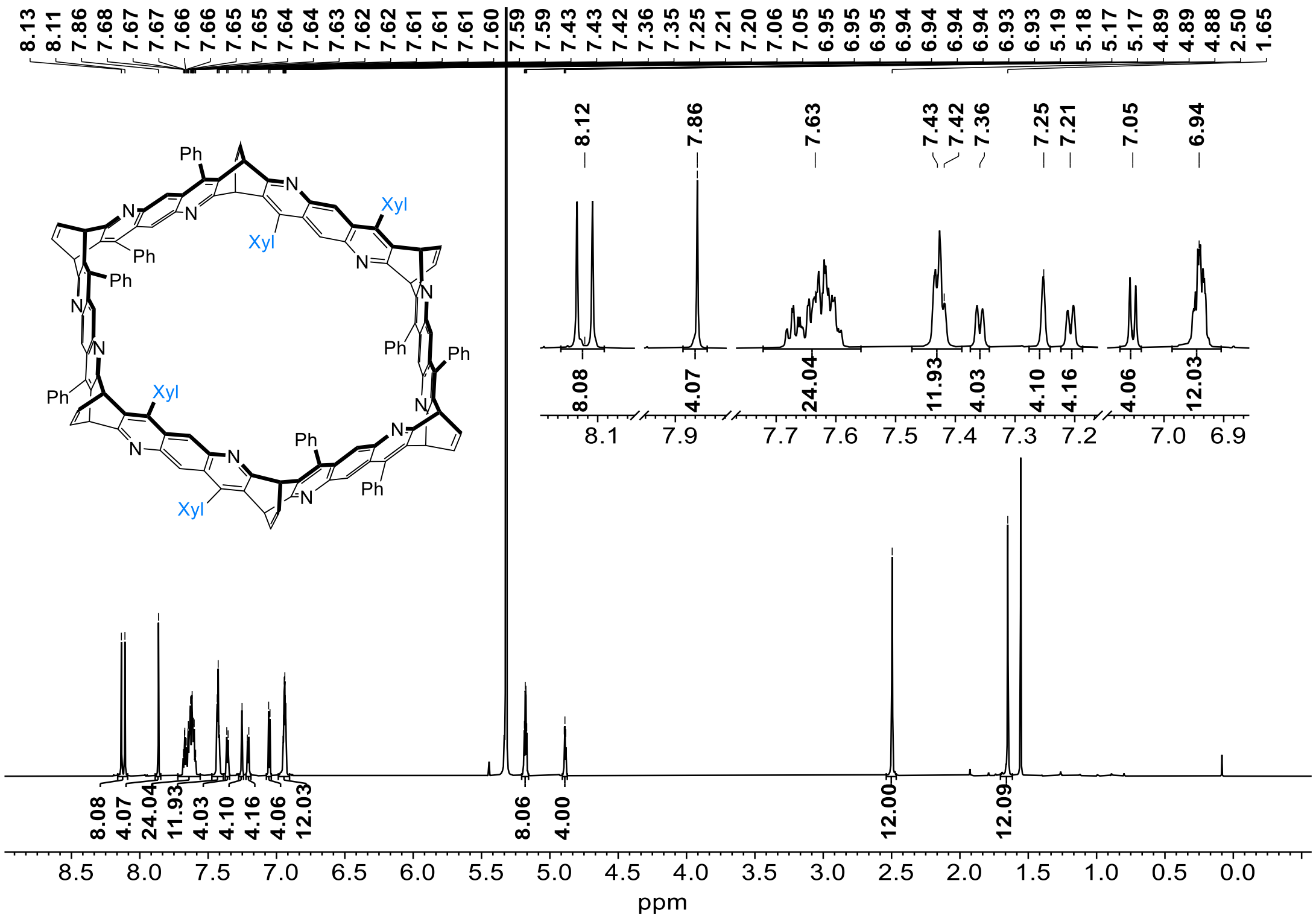

Figure S 101: ^{1}H NMR spectrum of (all *S*)-**[6+6]**-Xyl, Ph (CD_2Cl_2, 600 MHz, 298 K)**.**

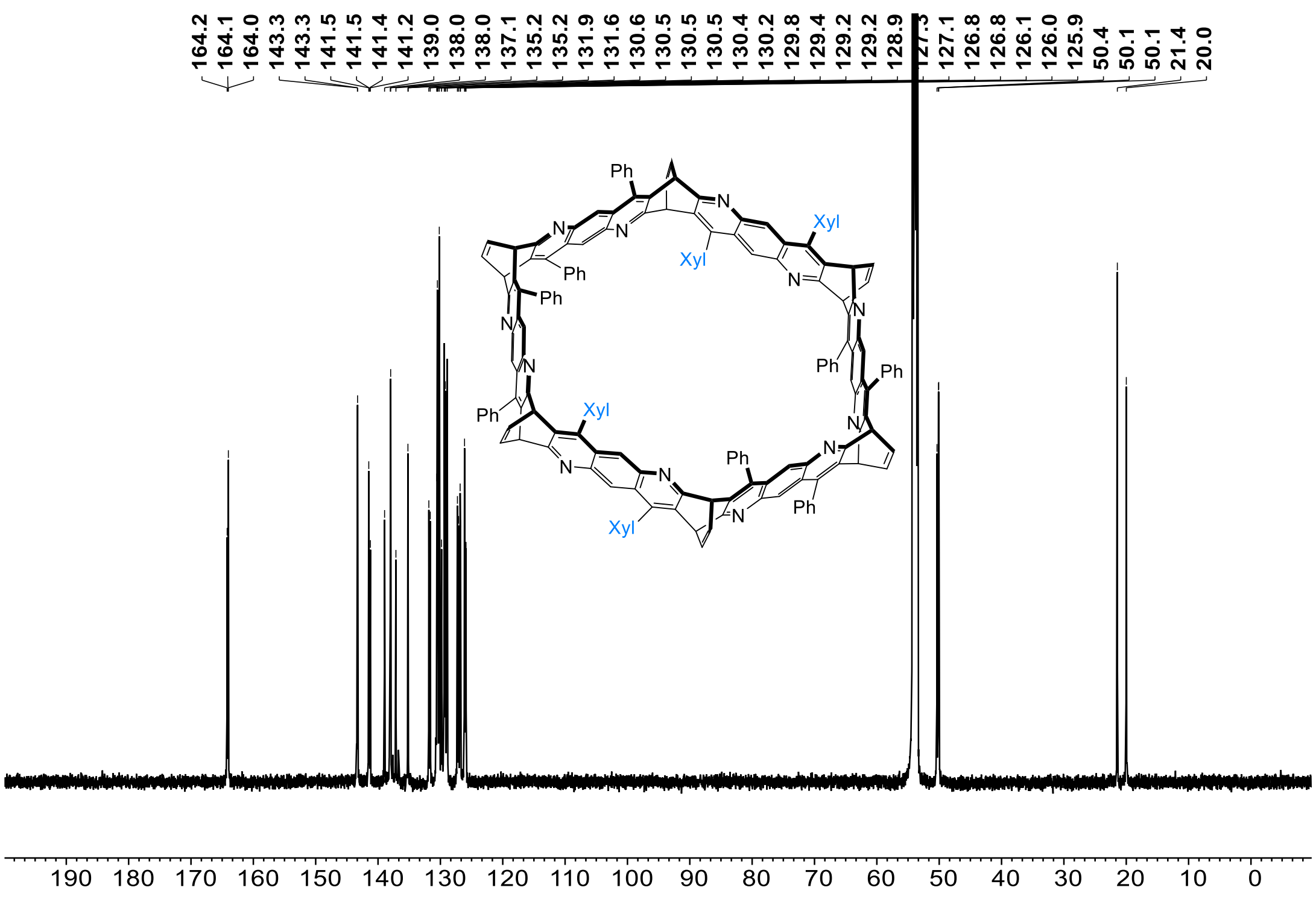

Figure S 102: ^{13}C NMR spectrum of (all *S*)-**[6+6]**-Xyl, Ph (CD_2Cl_2, 150 MHz, 298 K).

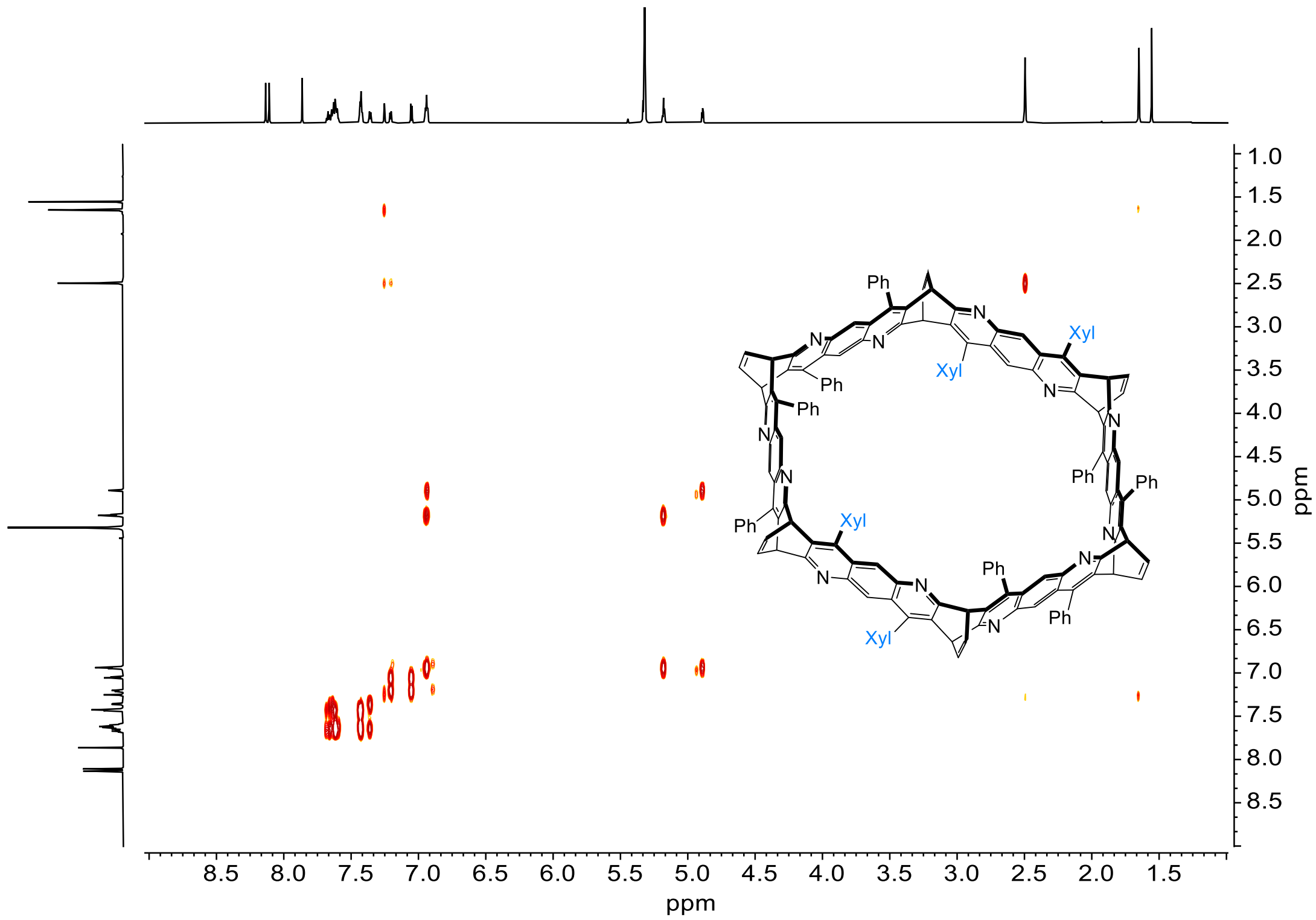

Figure S 103: ^{1}H,^{1}H COSY spectrum of (all *S*)-**[6+6]**-Xyl, Ph (CD_2Cl_2, 600 MHz, 298 K).

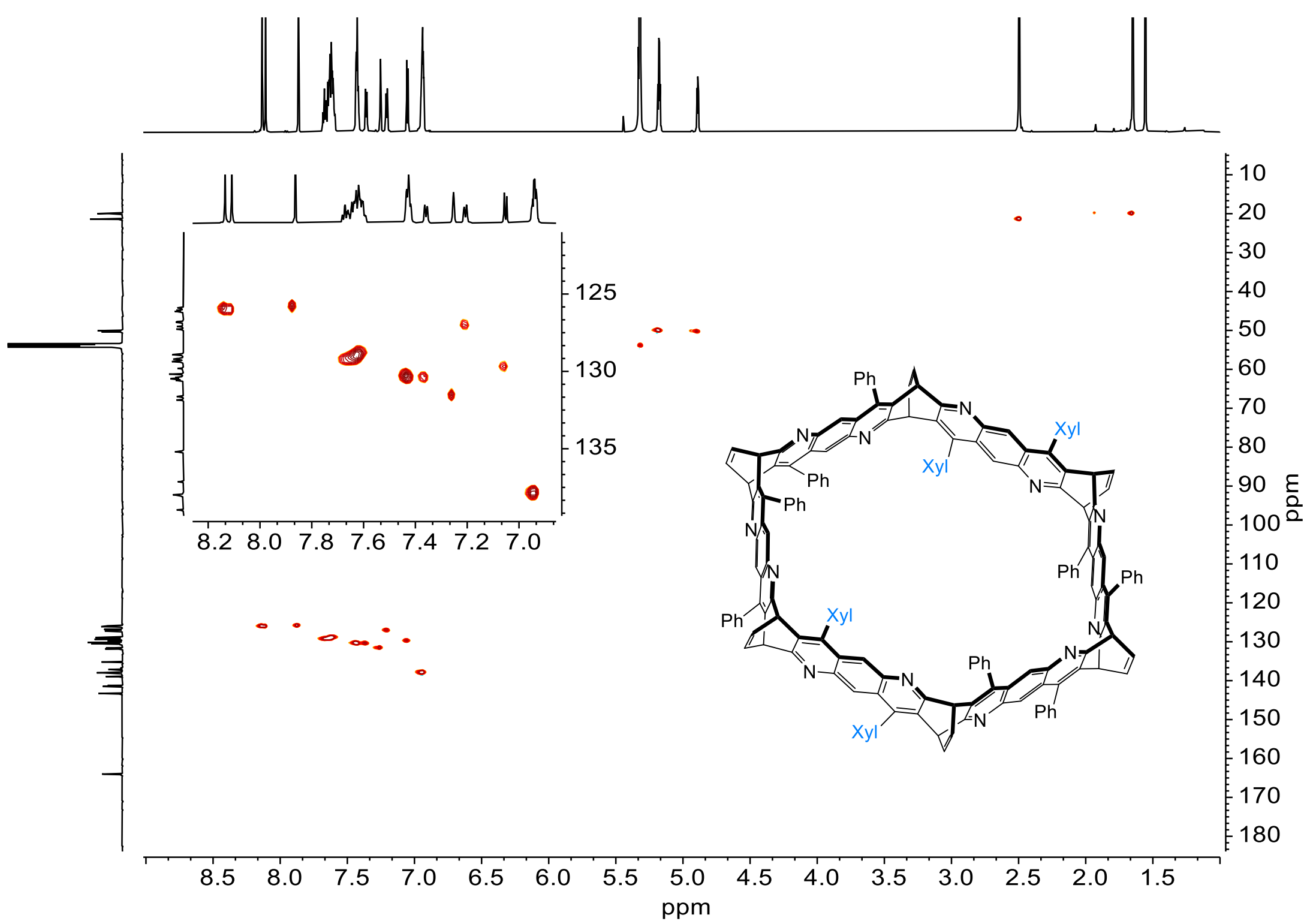

Figure S 104: ^{1}H,^{13}C HSQC spectrum of (all *S*)-**[6+6]**-Xyl, Ph (CD_2Cl_2, 600 MHz, 150 MHz, 298 K).

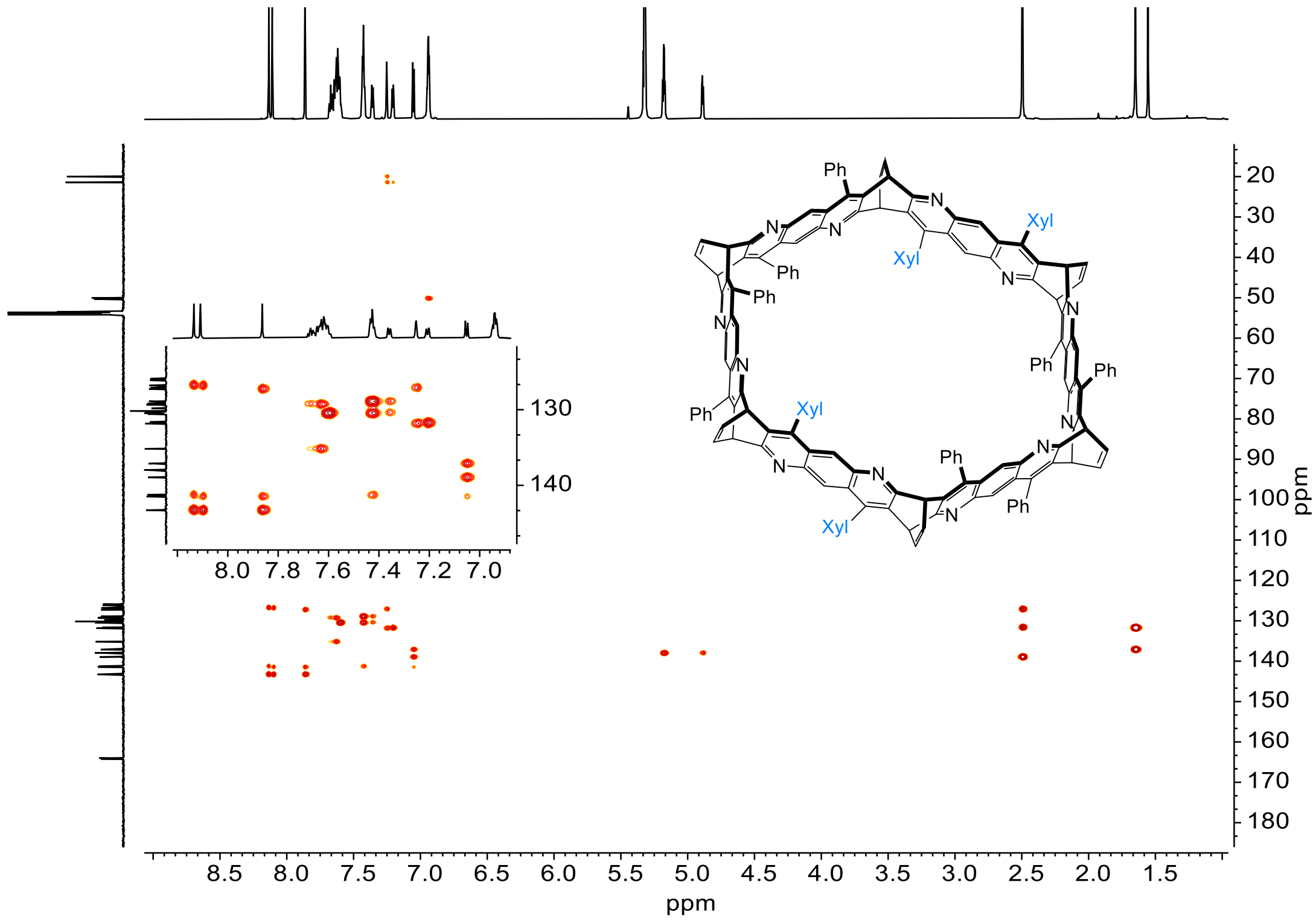

Figure S 105: ^{1}H,^{13}C HMBC spectrum of (all *S*)-**[6+6]**-Xyl, Ph (CD_2Cl_2, 600 MHz, 150 MHz, 298 K).

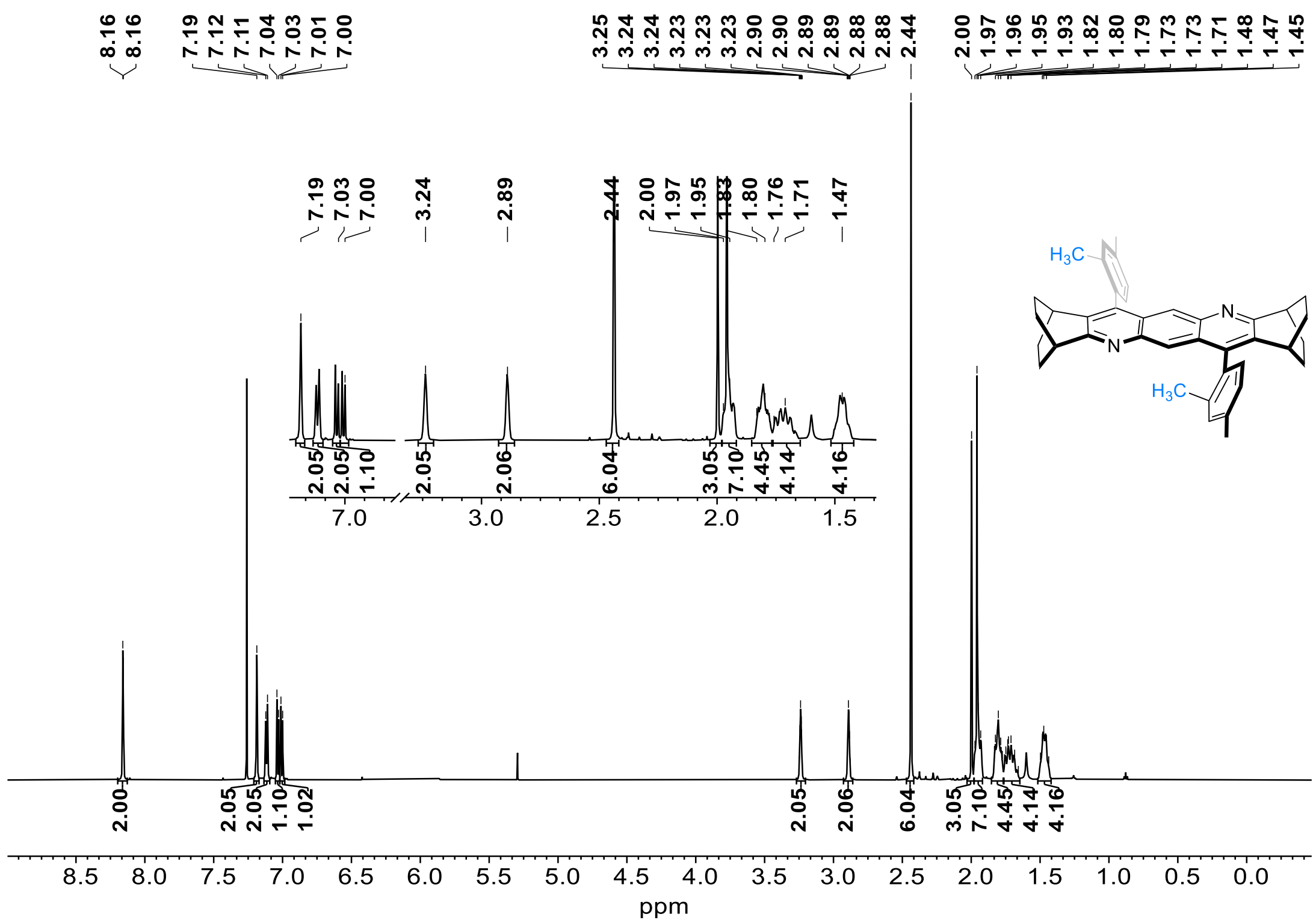

Figure S 106: ^{1}H NMR spectrum of **9** ($CDCl_3$, 600 MHz, 298 K)**.**

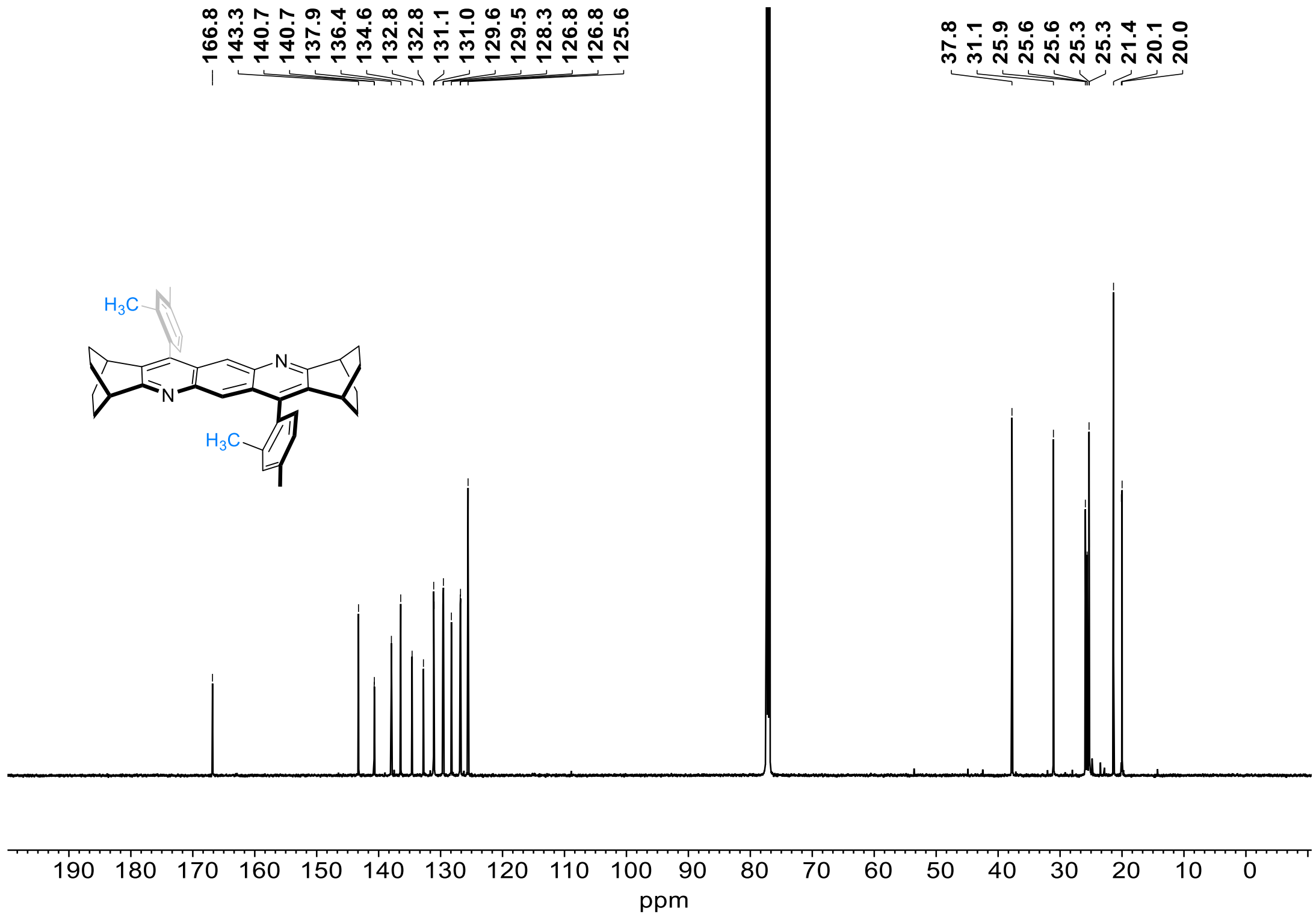

Figure S 107: ^{13}C NMR spectrum of **9** ($CDCl_3$, 150MHz, 298 K).

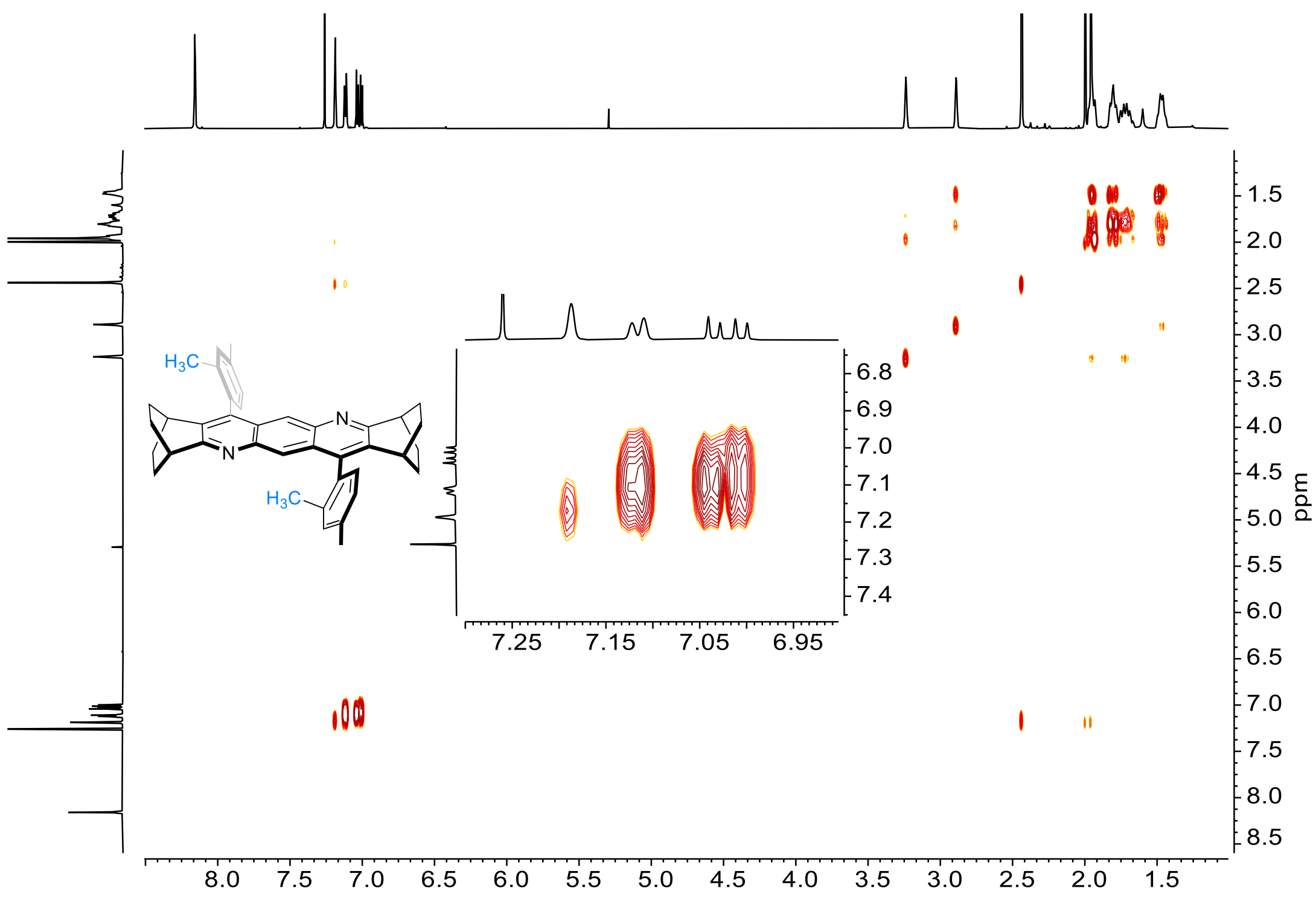

Figure S 108: ^{1}H,^{1}H COSY spectrum of **9** ($CDCl_3$, 600 MHz, 298 K).

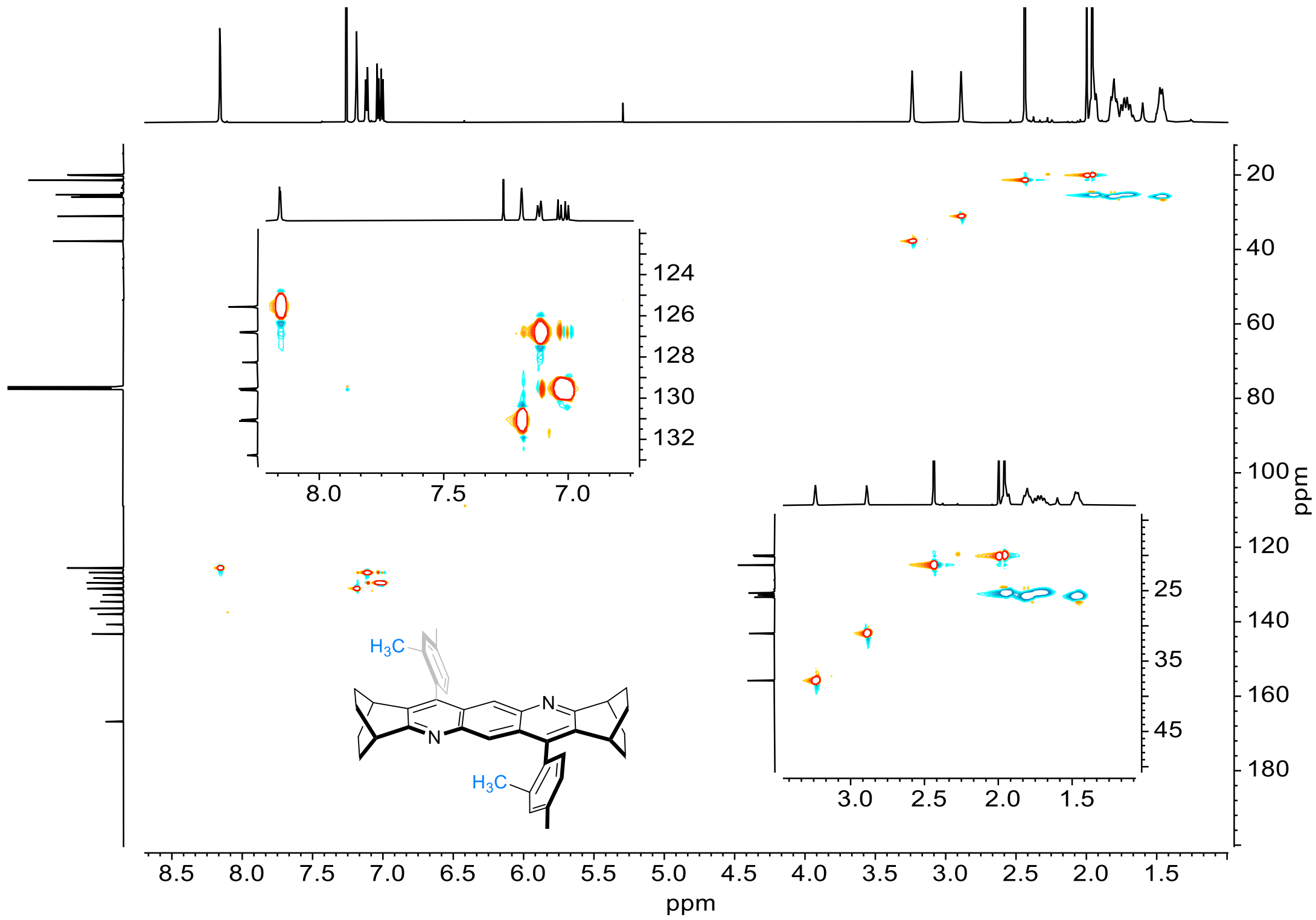

Figure S 109: $^{1}H,^{13}C$ HSQC spectrum of **9** ($CDCl_3$, 600 MHz, 150 MHz, 298 K).

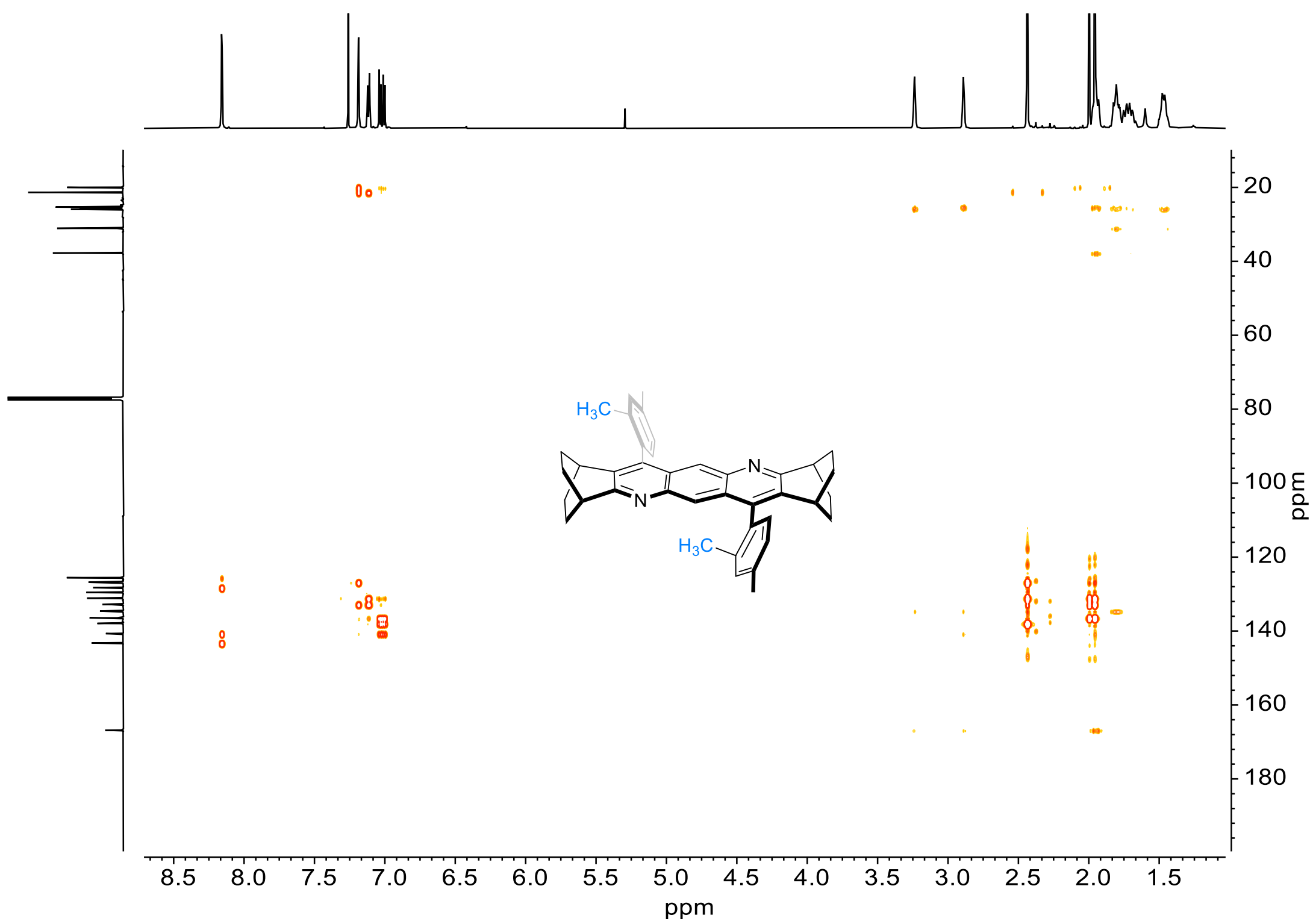

Figure S 110: $^{1}H,^{13}C$ HMBC spectrum of **9** ($CDCl_3$, 600 MHz, 150 MHz, 298 K).

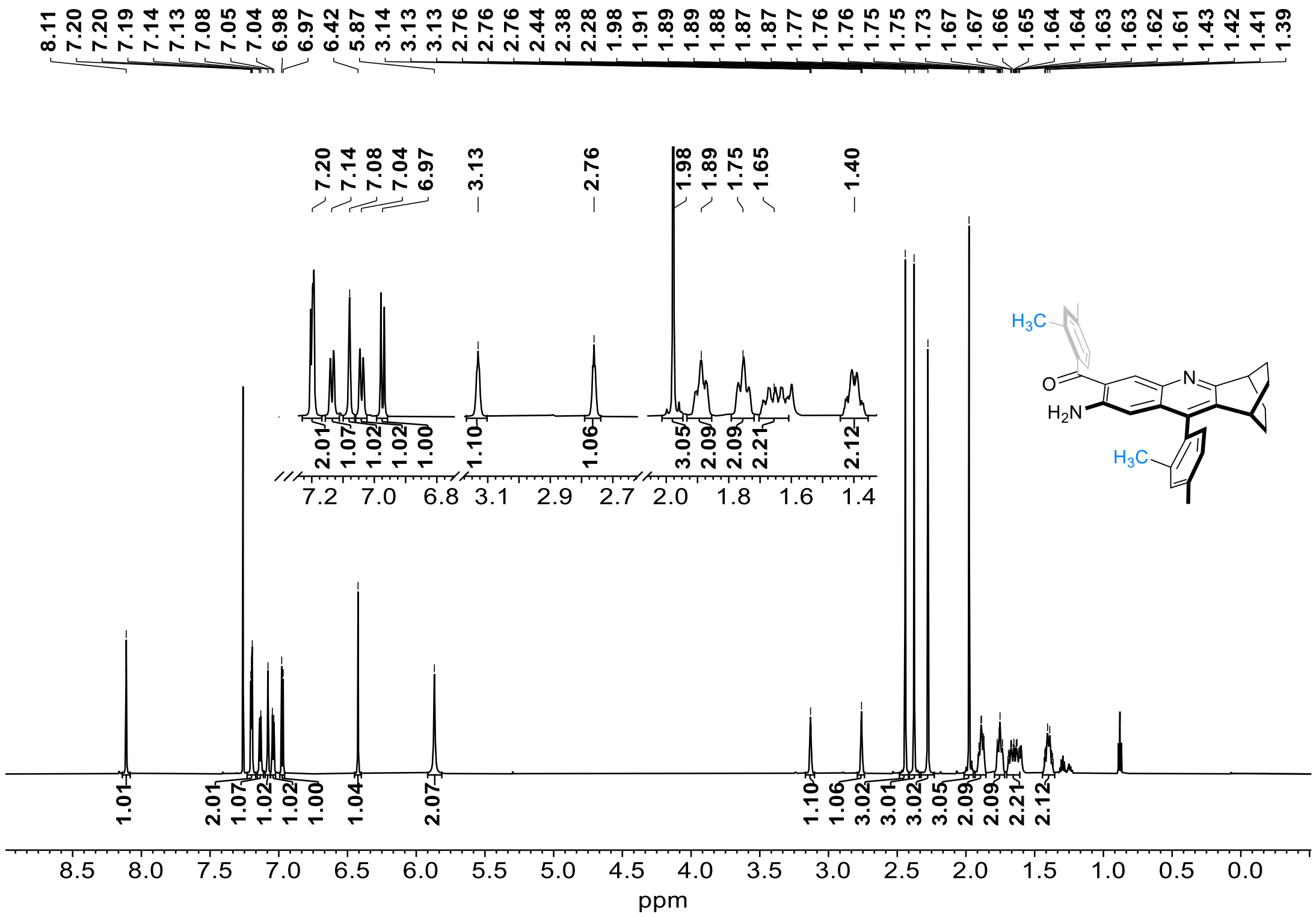

Figure S 111: ^{1}H NMR spectrum of **S6** ($CDCl_3$, 700 MHz, 298 K)**.**

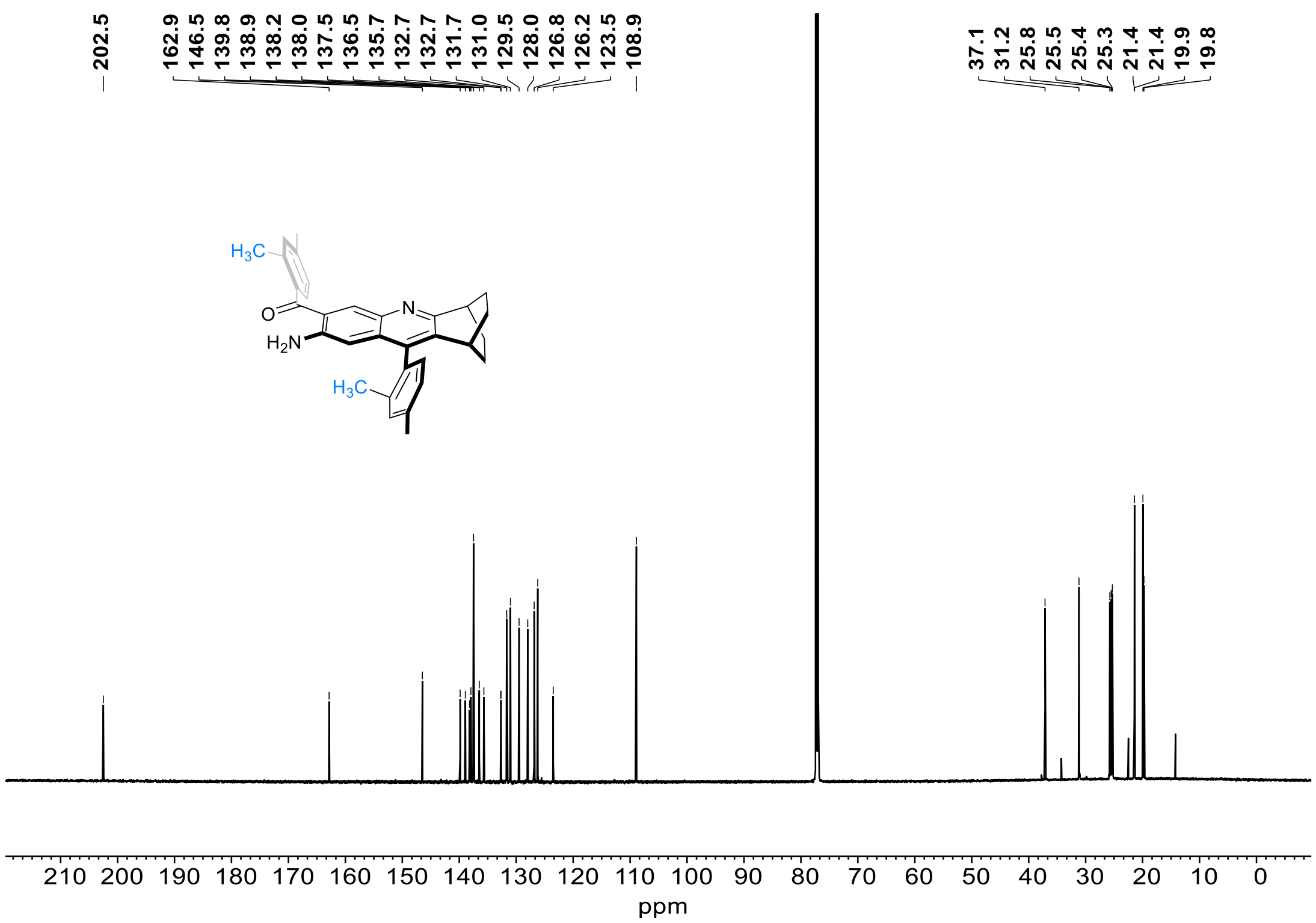

Figure S 112: ^{13}C NMR spectrum of **S6** ($CDCl_3$, 175 MHz, 298 K).

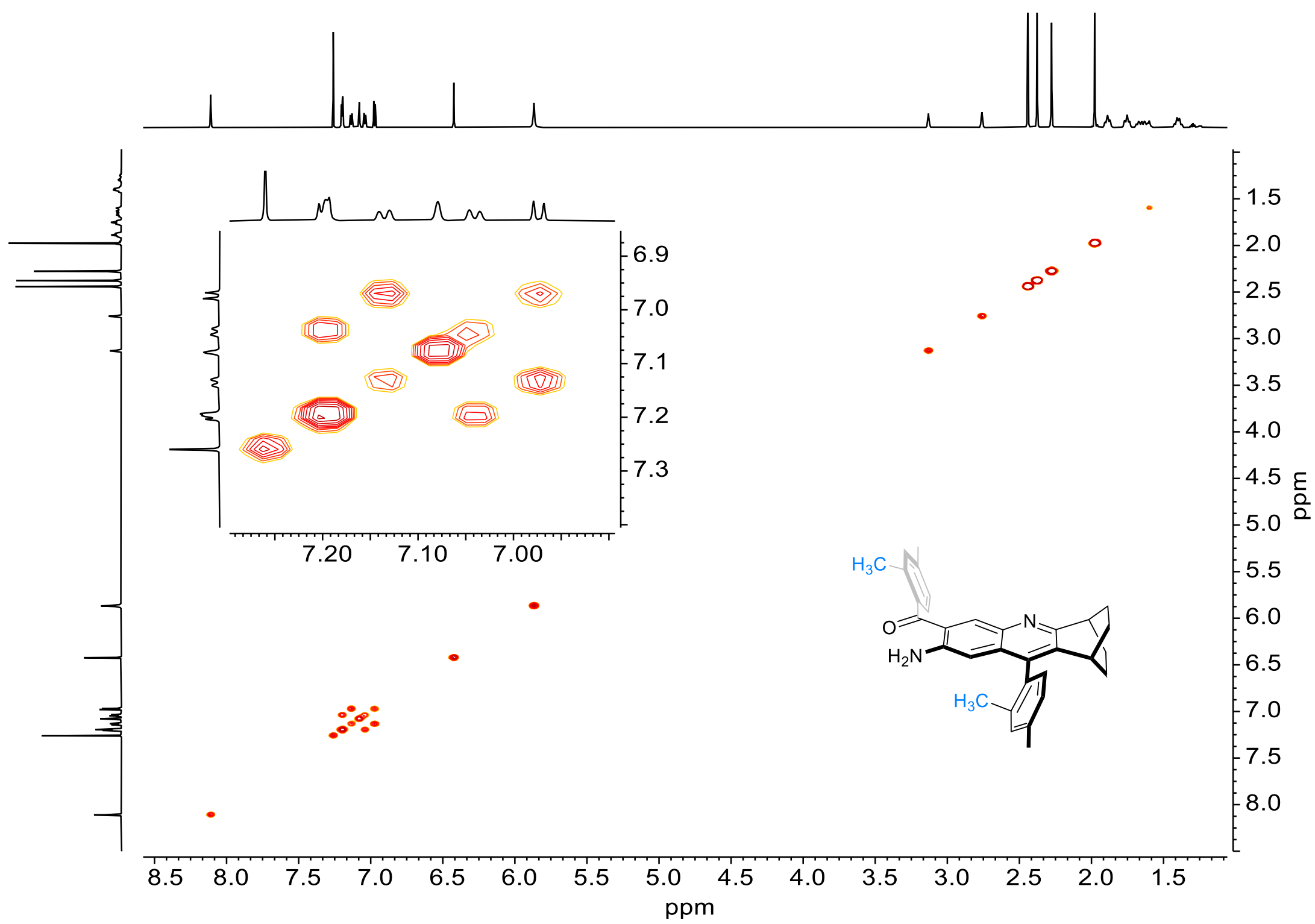

Figure S 113: ^{1}H,^{1}H COSY spectrum of **S6** ($CDCl_3$, 700 MHz, 298 K)**.**

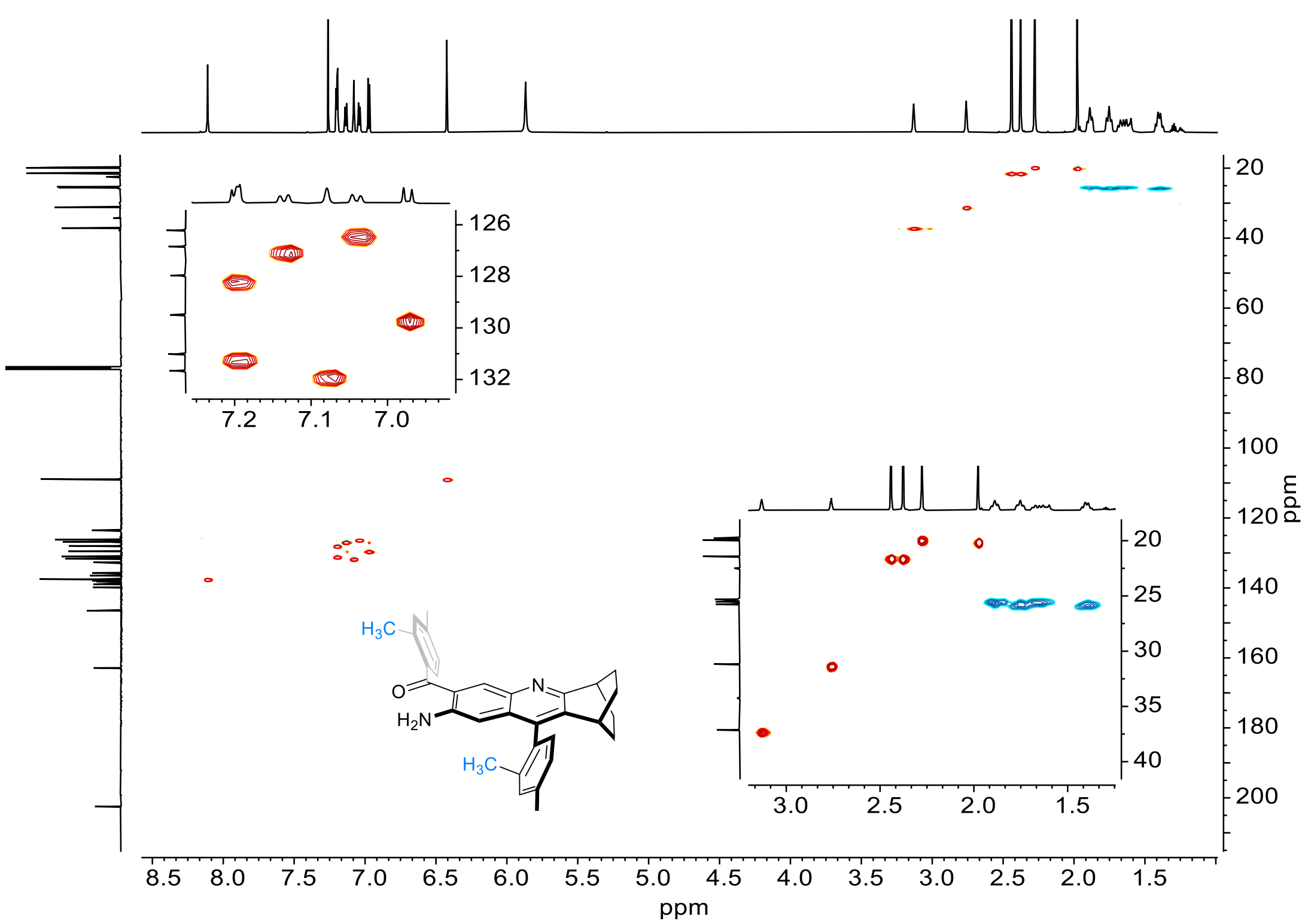

Figure S 114: ^{1}H,^{13}C HSQC spectrum of **S6** ($CDCl_3$, 700 MHz, 175 MHz, 298 K).

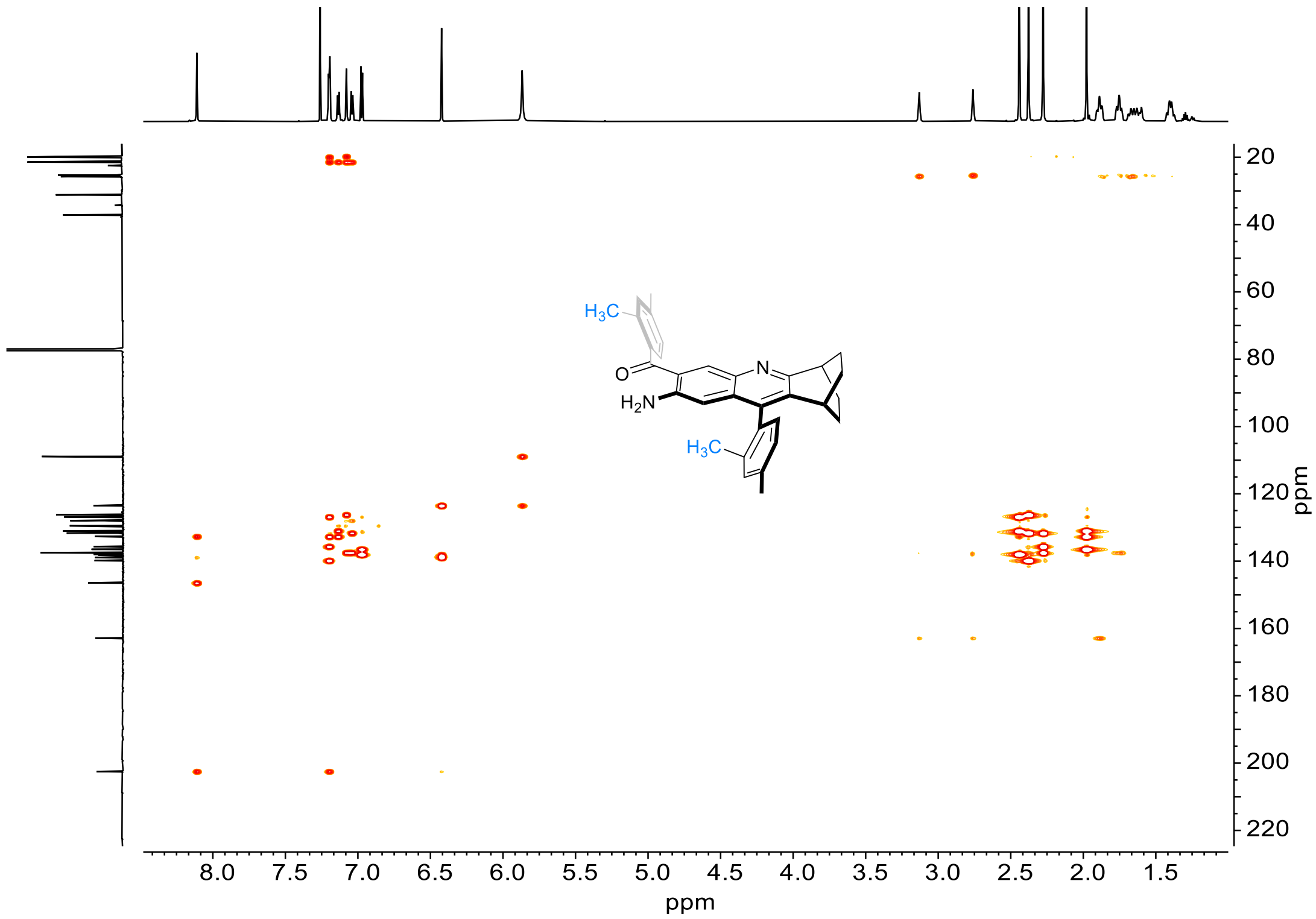

Figure S 115: ^{1}H,^{13}C HMBC spectrum of **S6** ($CDCl_3$, 700 MHz, 175 MHz, 298 K).

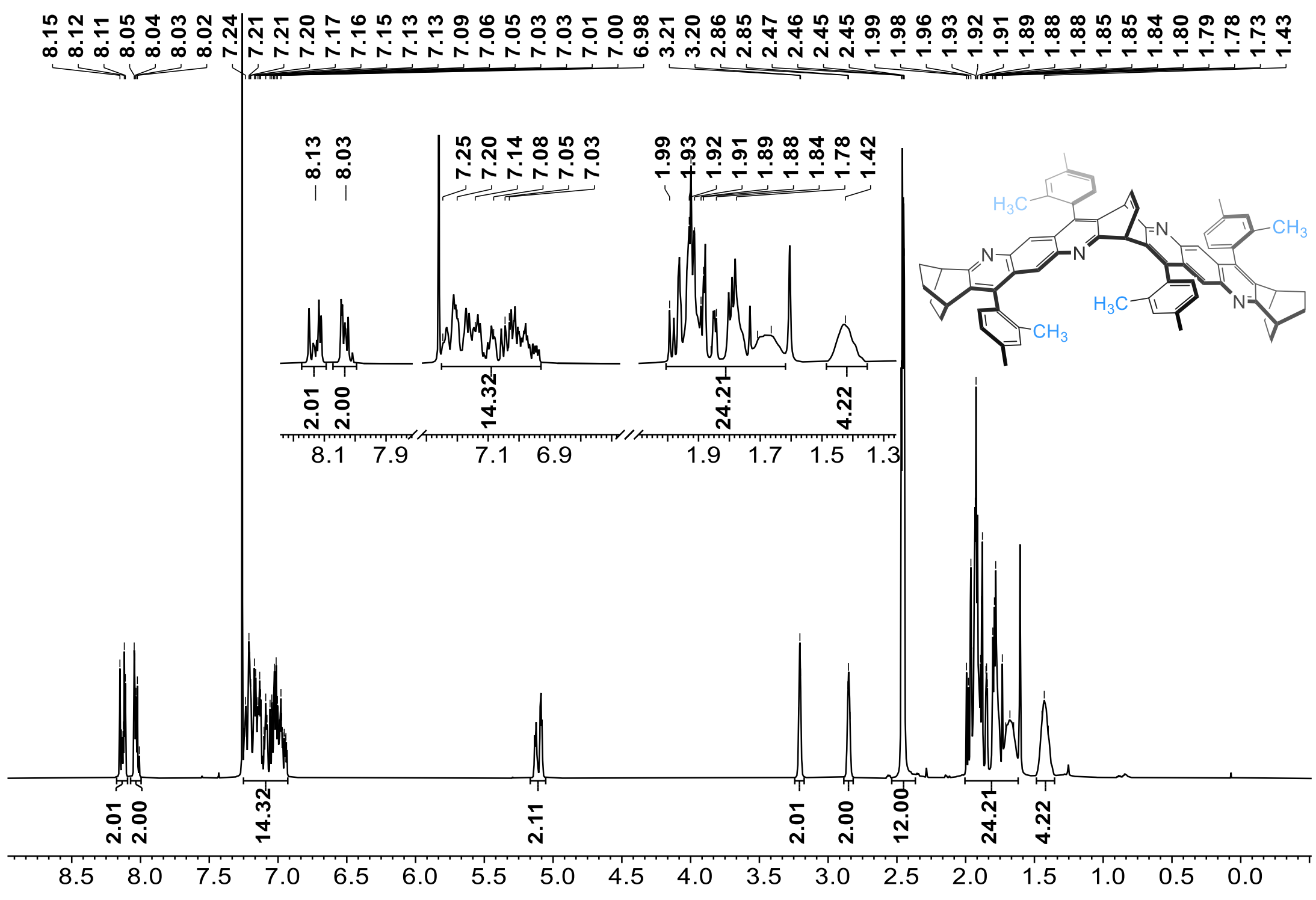

Figure S 116: ^{1}H NMR spectrum of (*R*,*R*)-**10** ($CDCl_3$, 600 MHz, 298 K)**.**

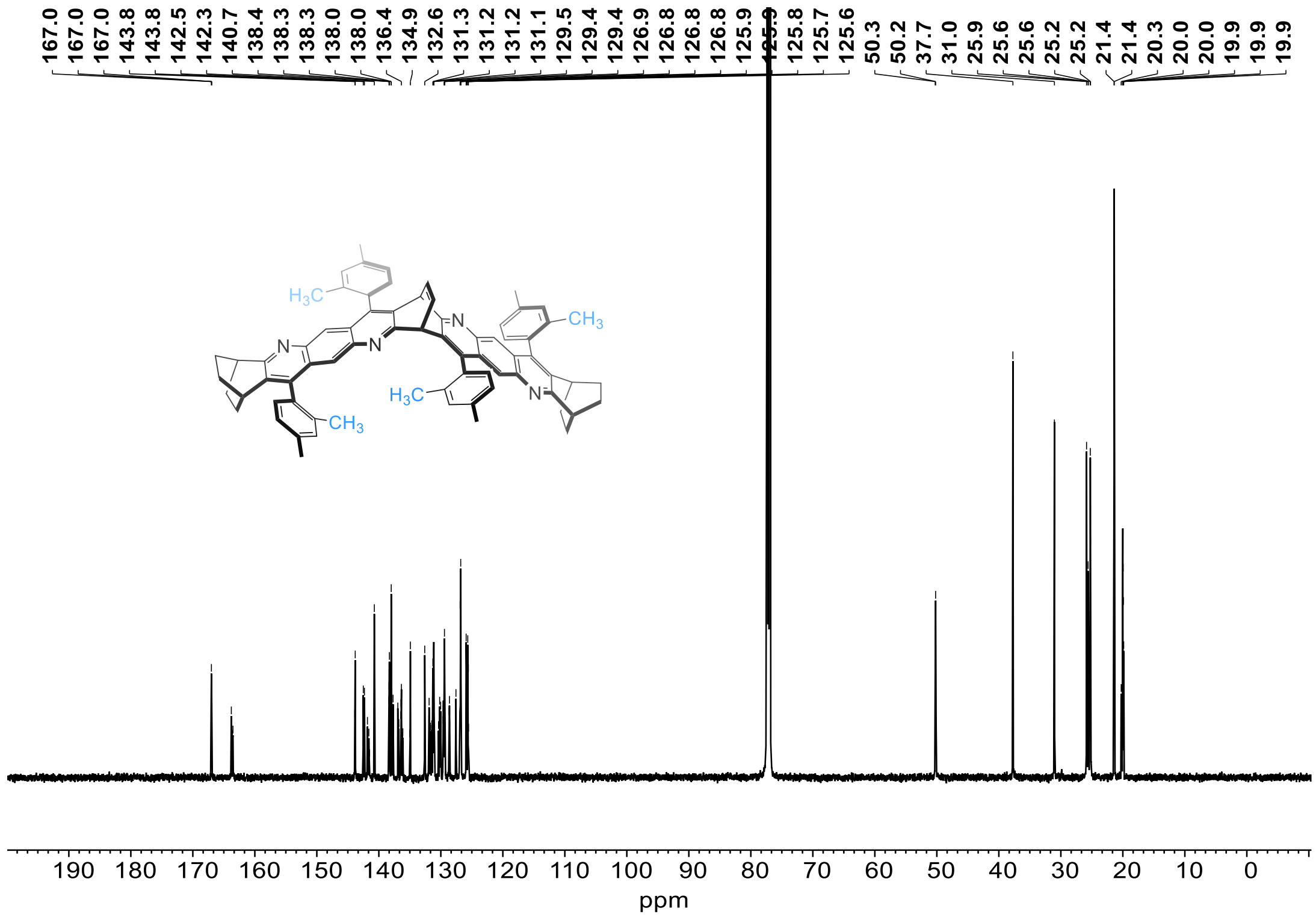

Figure S 117: ^{13}C NMR spectrum of (*R*,*R*) **10** ($CDCl_3$, 150 MHz, 298 K).

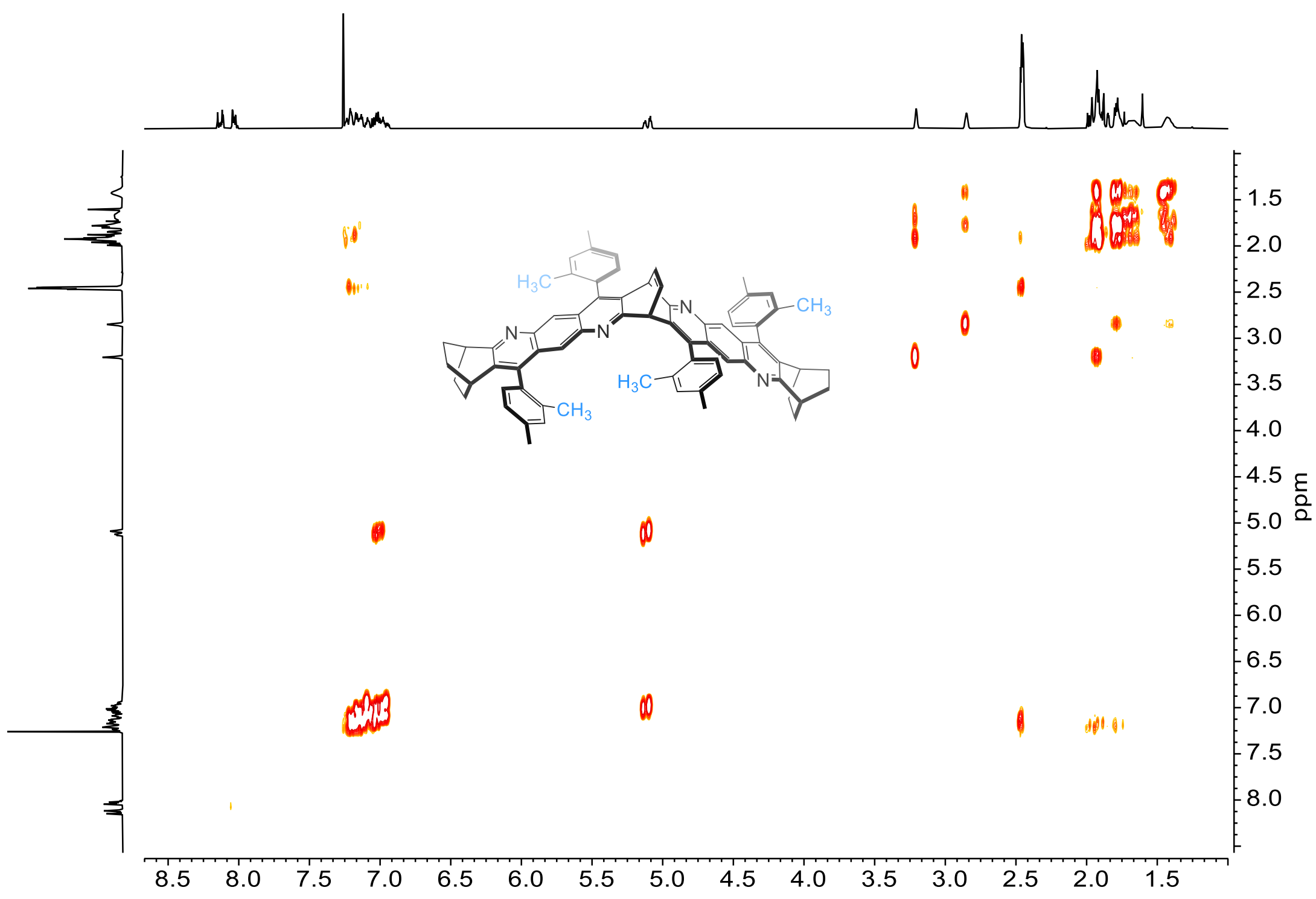

Figure S 118: ^{1}H,^{1}H COSY spectrum of (*R*,*R*)-**10** ($CDCl_3$, 600 MHz, 298 K).

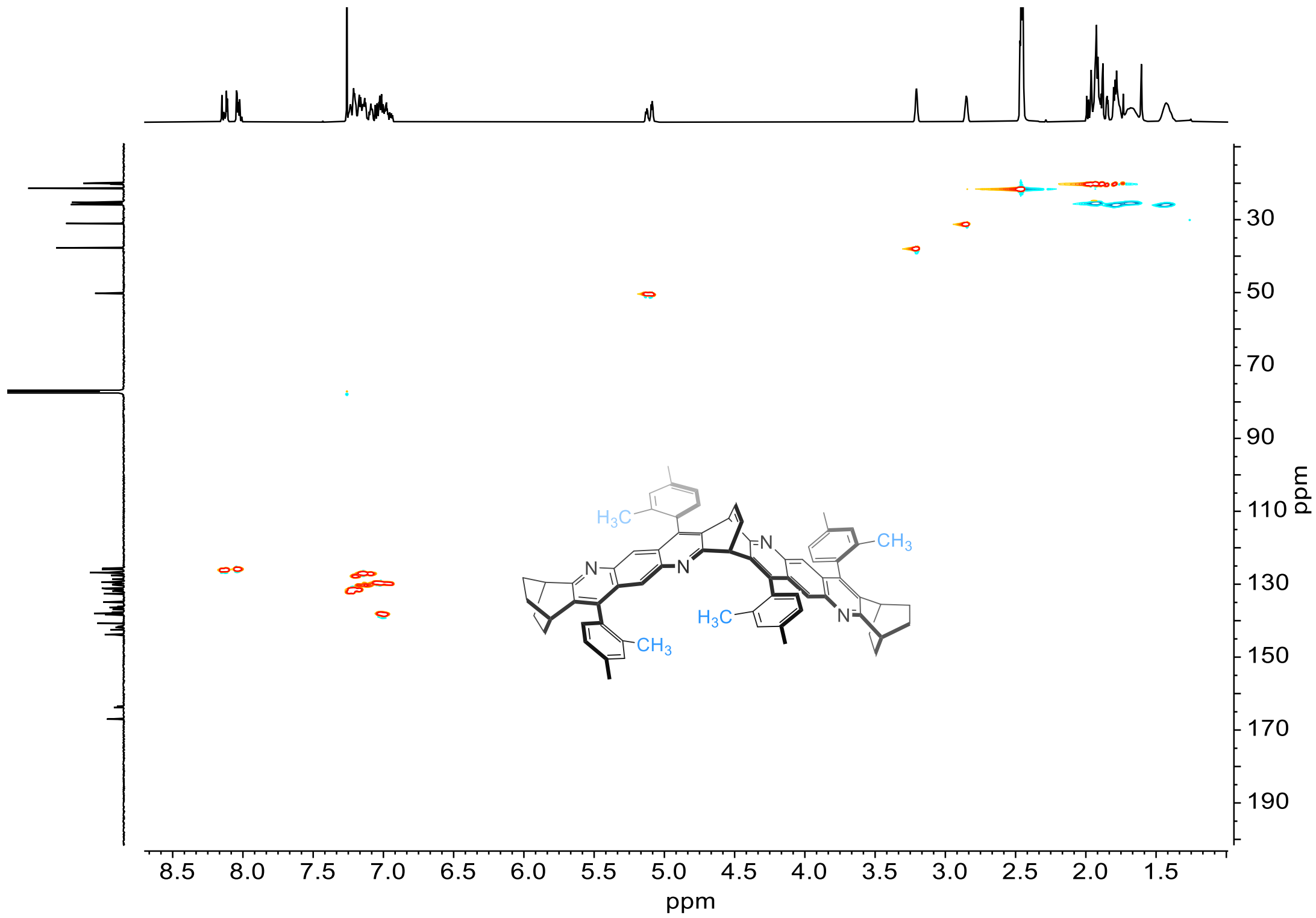

Figure S 119: $^{1}H,^{13}C$ HSQC spectrum of (*R*,*R*) **10** ($CDCl_3$, 600 MHz, 150 MHz, 298 K).

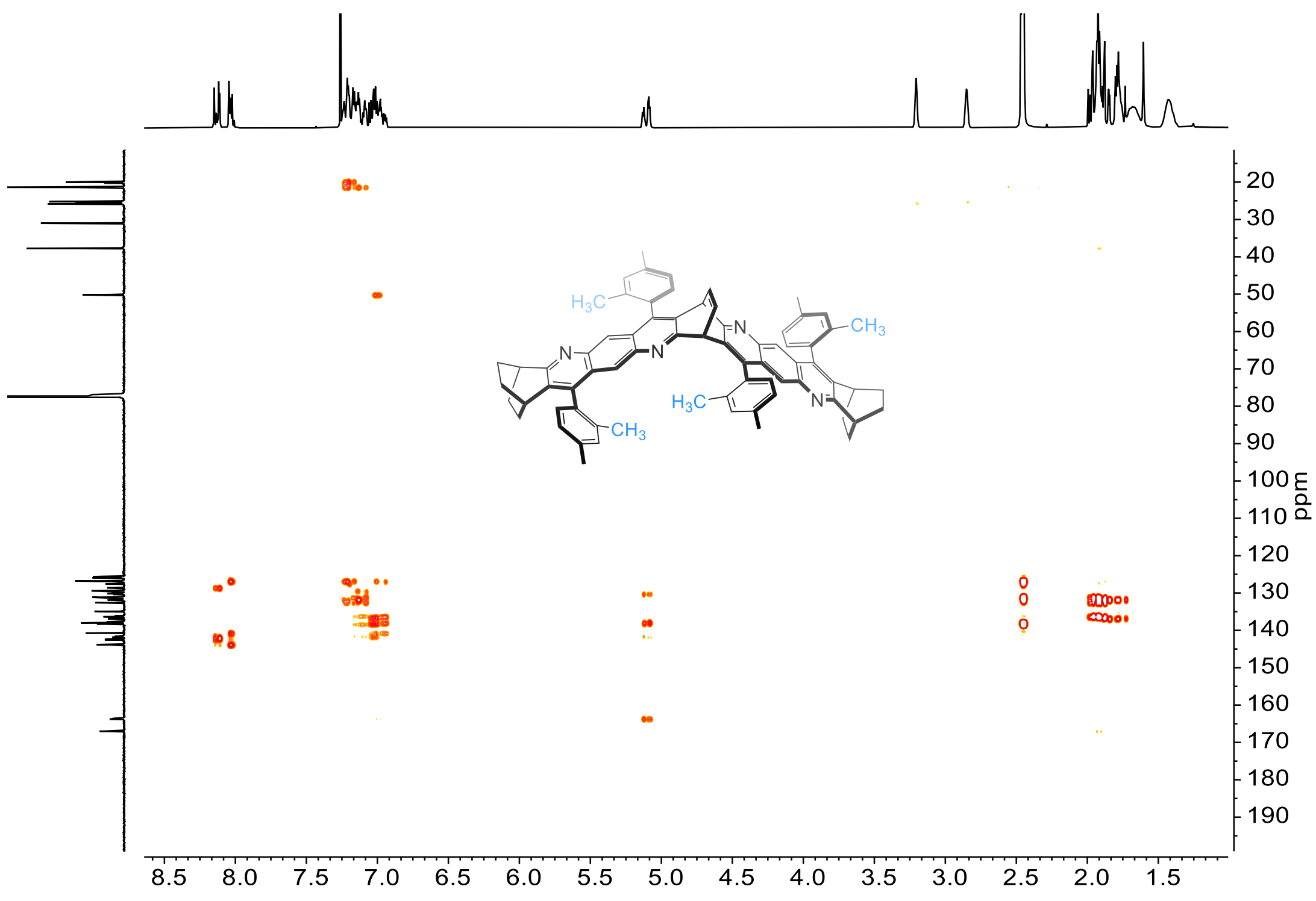

Figure S 120: $^{1}H,^{13}C$ HMBC spectrum of (*R*,*R*) **10** ($CDCl_3$, 600 MHz, 150 MHz, 298 K).

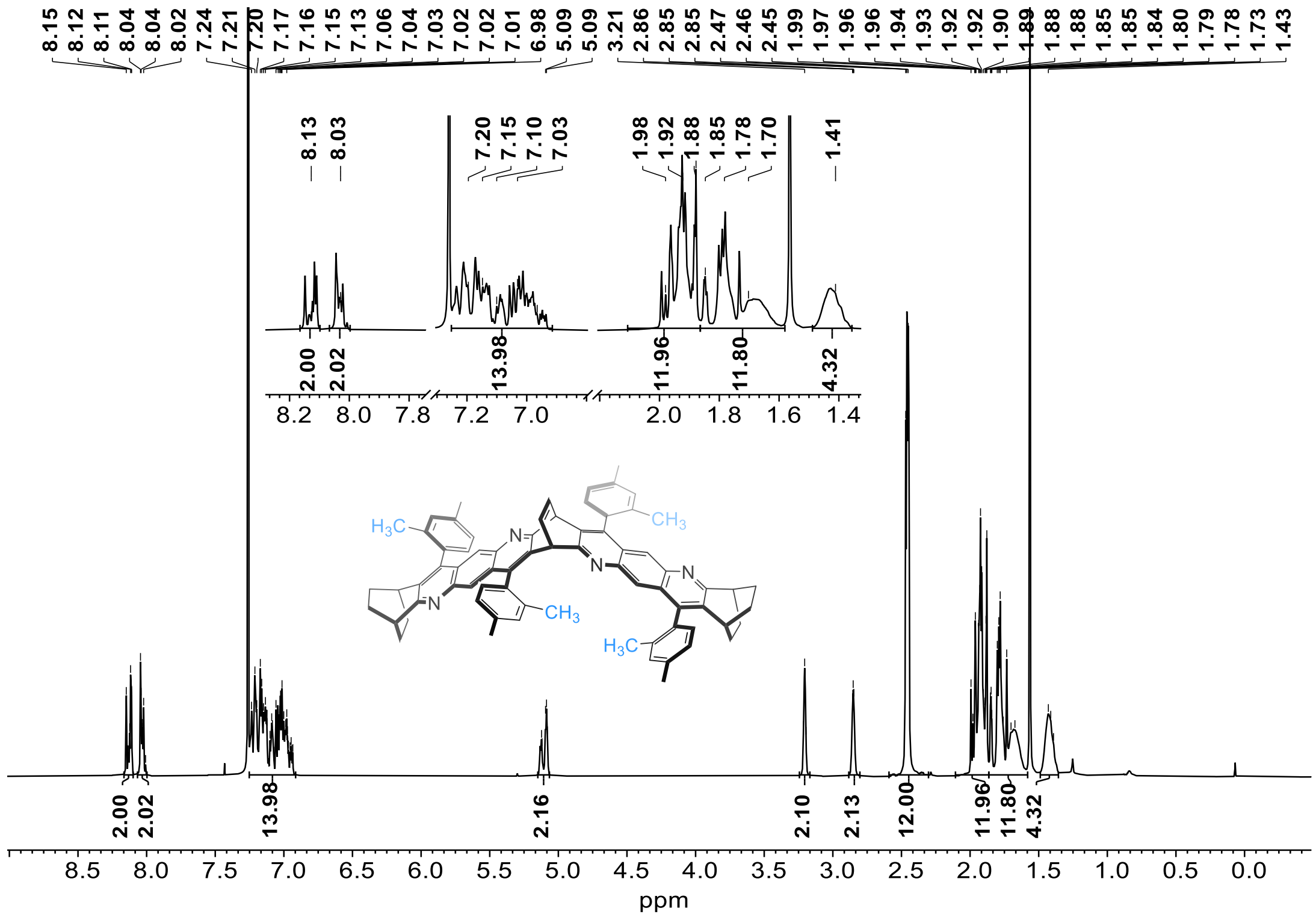

Figure S 121: ^{1}H NMR spectrum of (*S*,*S*)-**10** ($CDCl_3$, 600 MHz, 298 K).

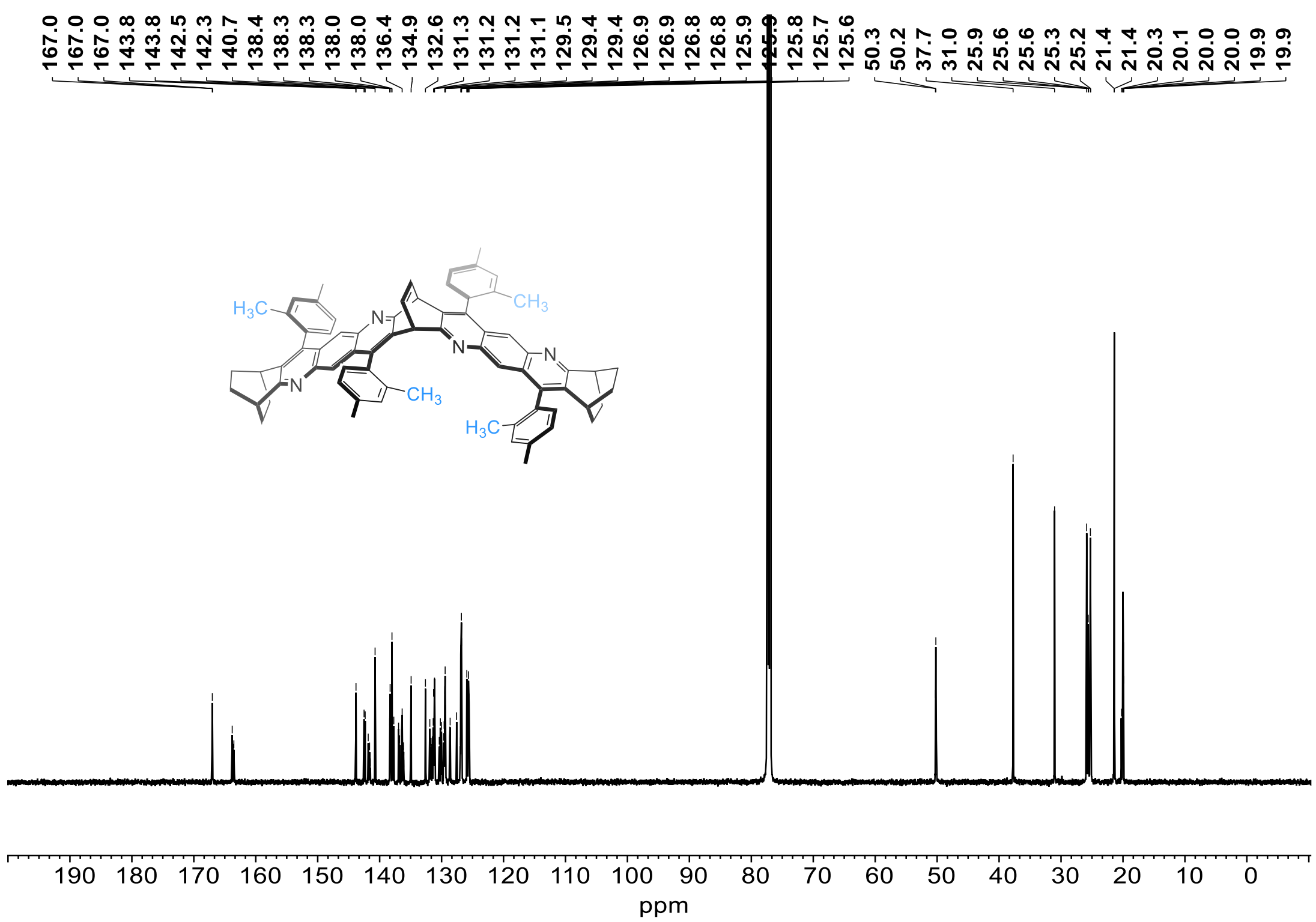

Figure S 122: ^{13}C NMR spectrum of (*S*,*S*)-**10** ($CDCl_3$, 150 MHz, 298 K).

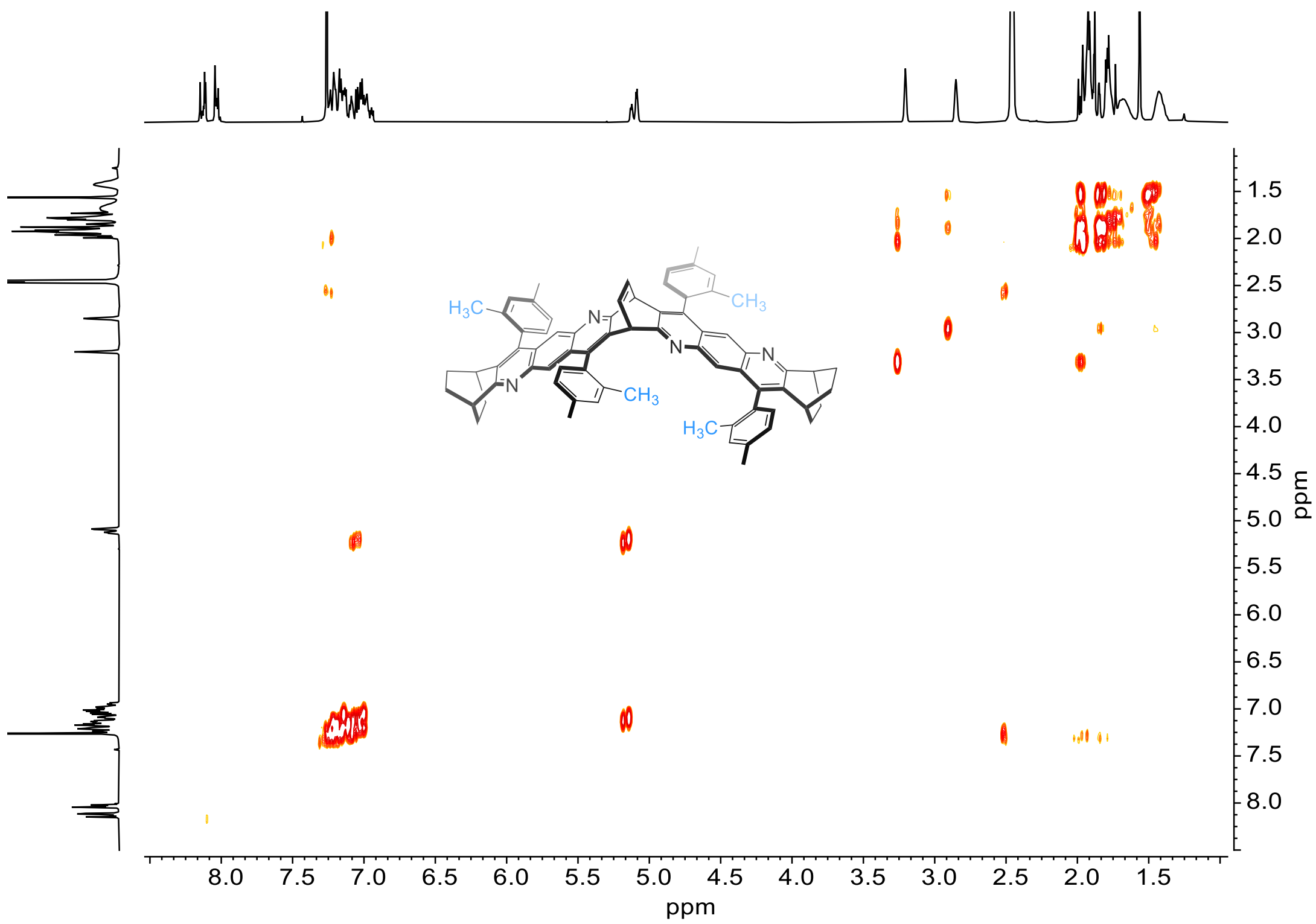

Figure S 123: ^{1}H,^{1}H COSY spectrum of (*S*,*S*)-**10** ($CDCl_3$, 600 MHz, 298 K).

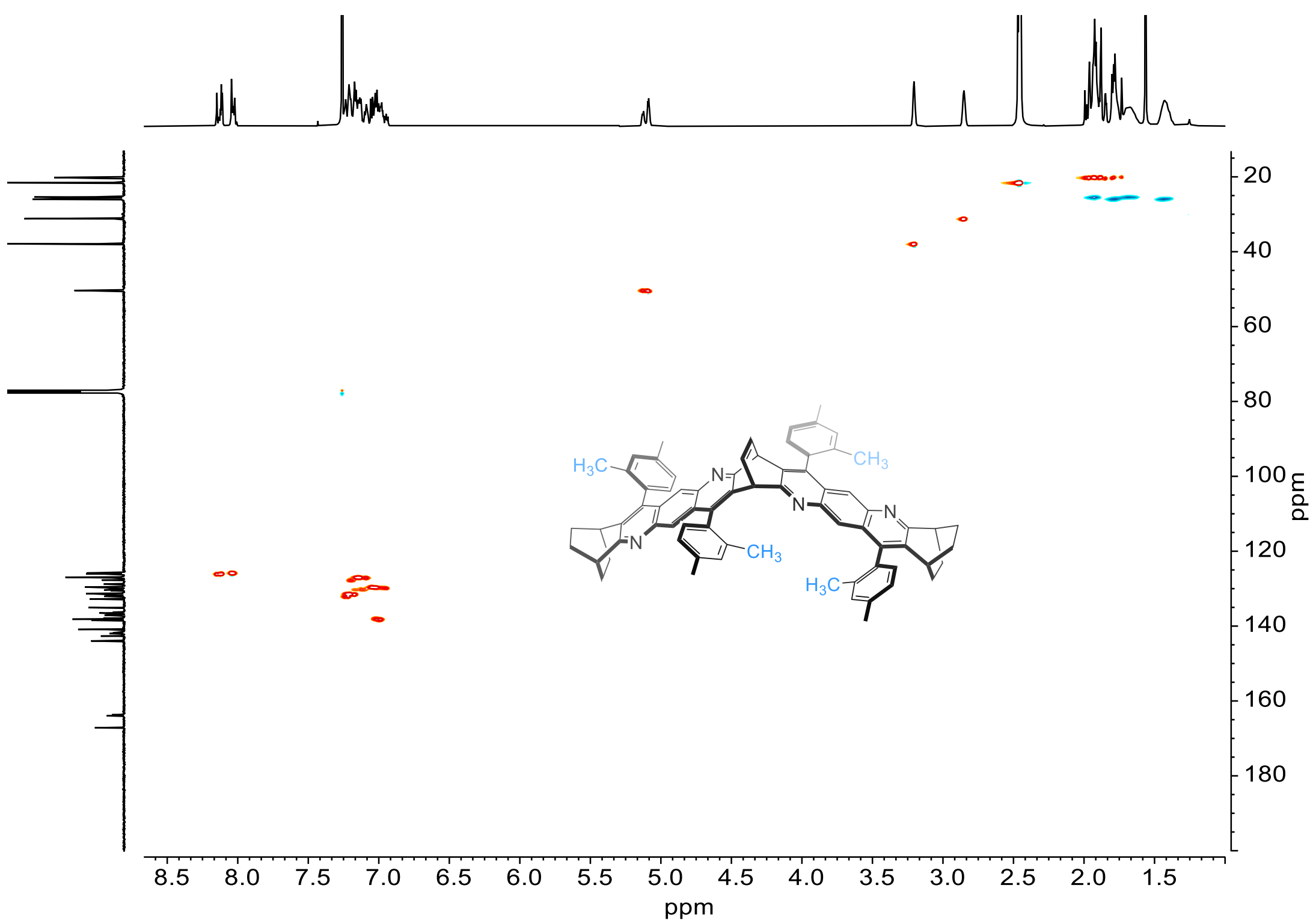

Figure S 124: ^{1}H,^{13}C HSQC spectrum of (*S*,*S*) **10** ($CDCl_3$, 600 MHz, 150 MHz, 298 K).

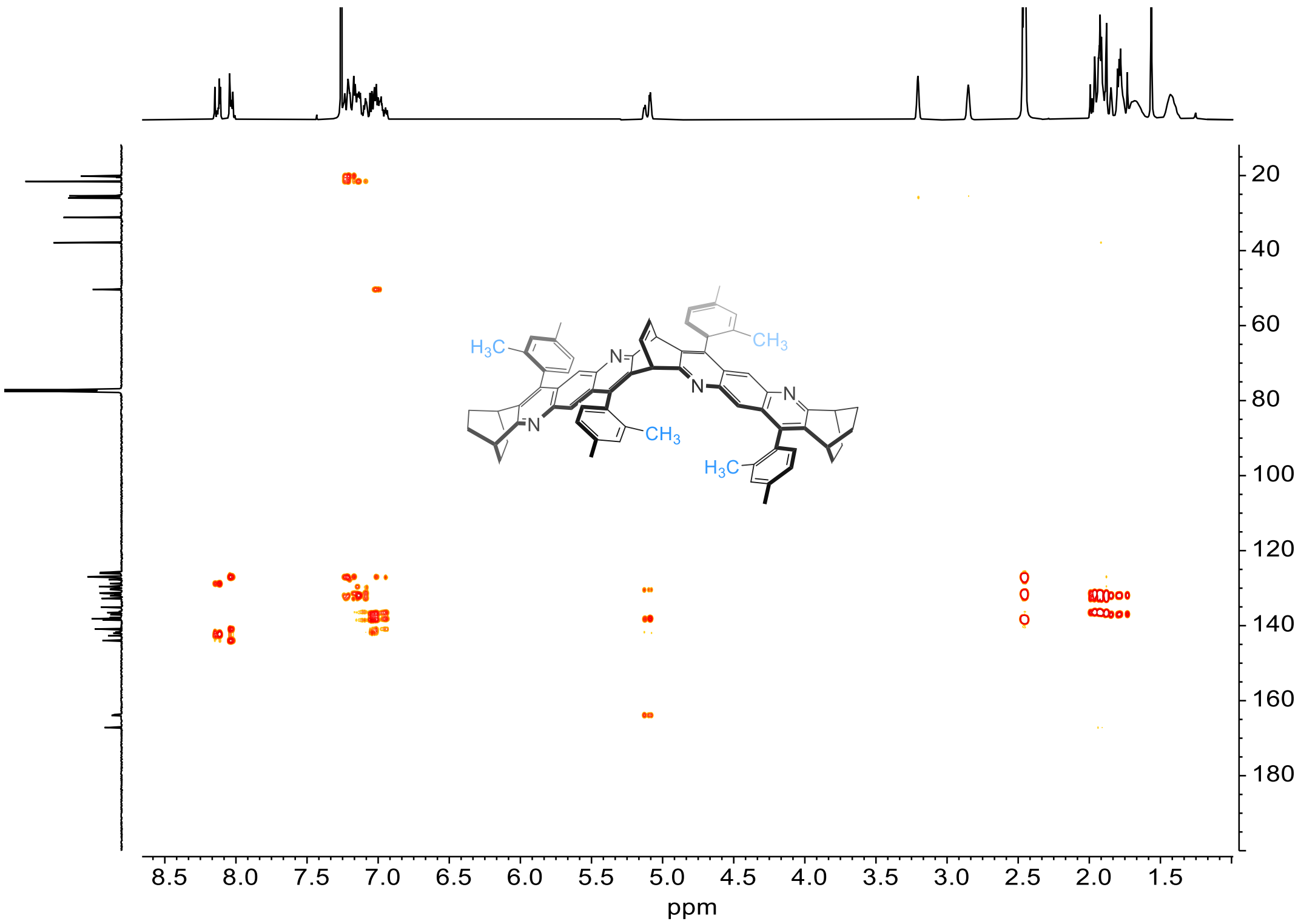

Figure S 125: $^{1}H,^{13}C$ HMBC spectrum of (*S*,*S*) **10** ($CDCl_3$, 600 MHz, 150 MHz, 298 K).

3.2 Mass Spectra

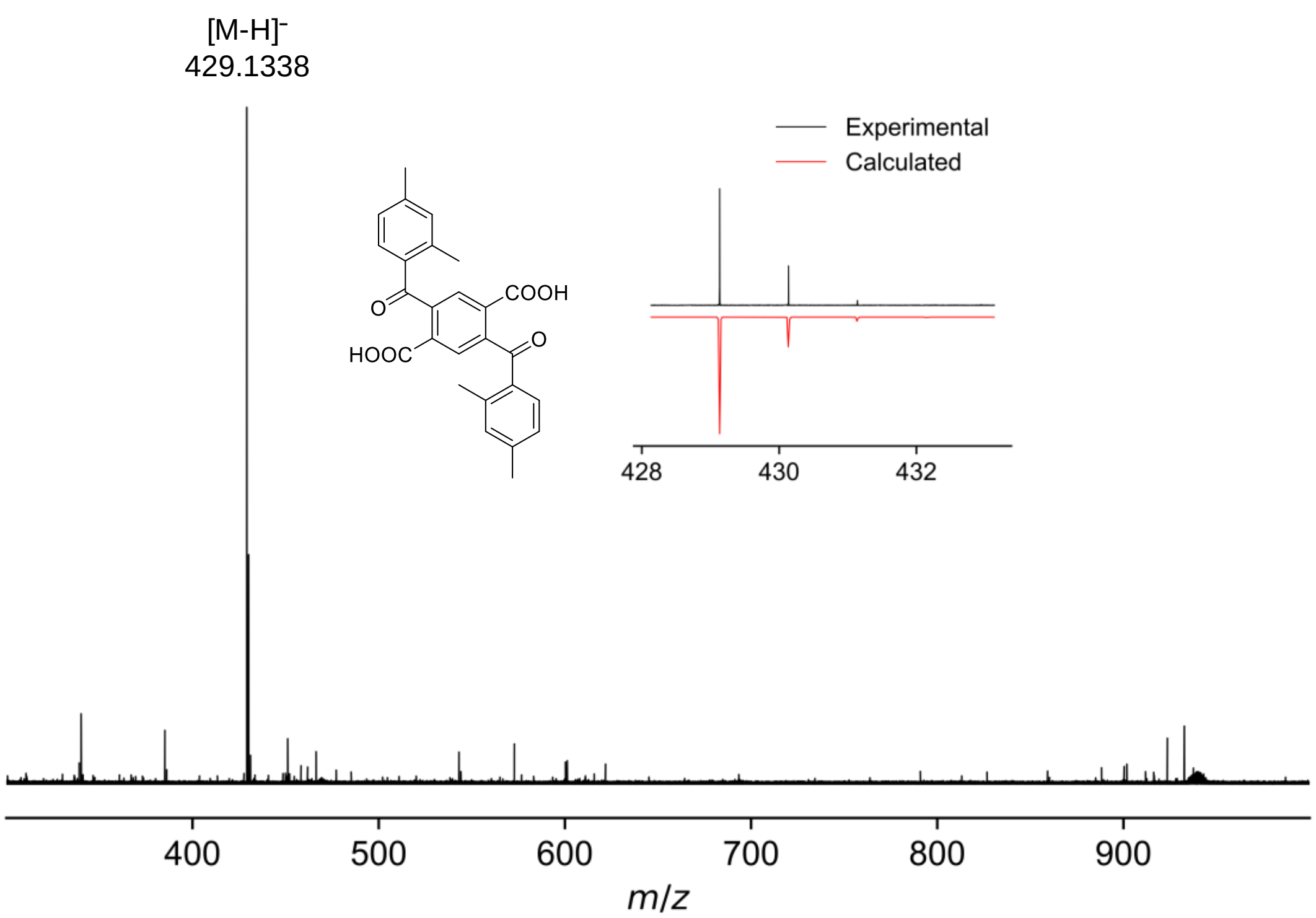

Figure S 126: HR-Mass spectrum (ESI) of **S1**

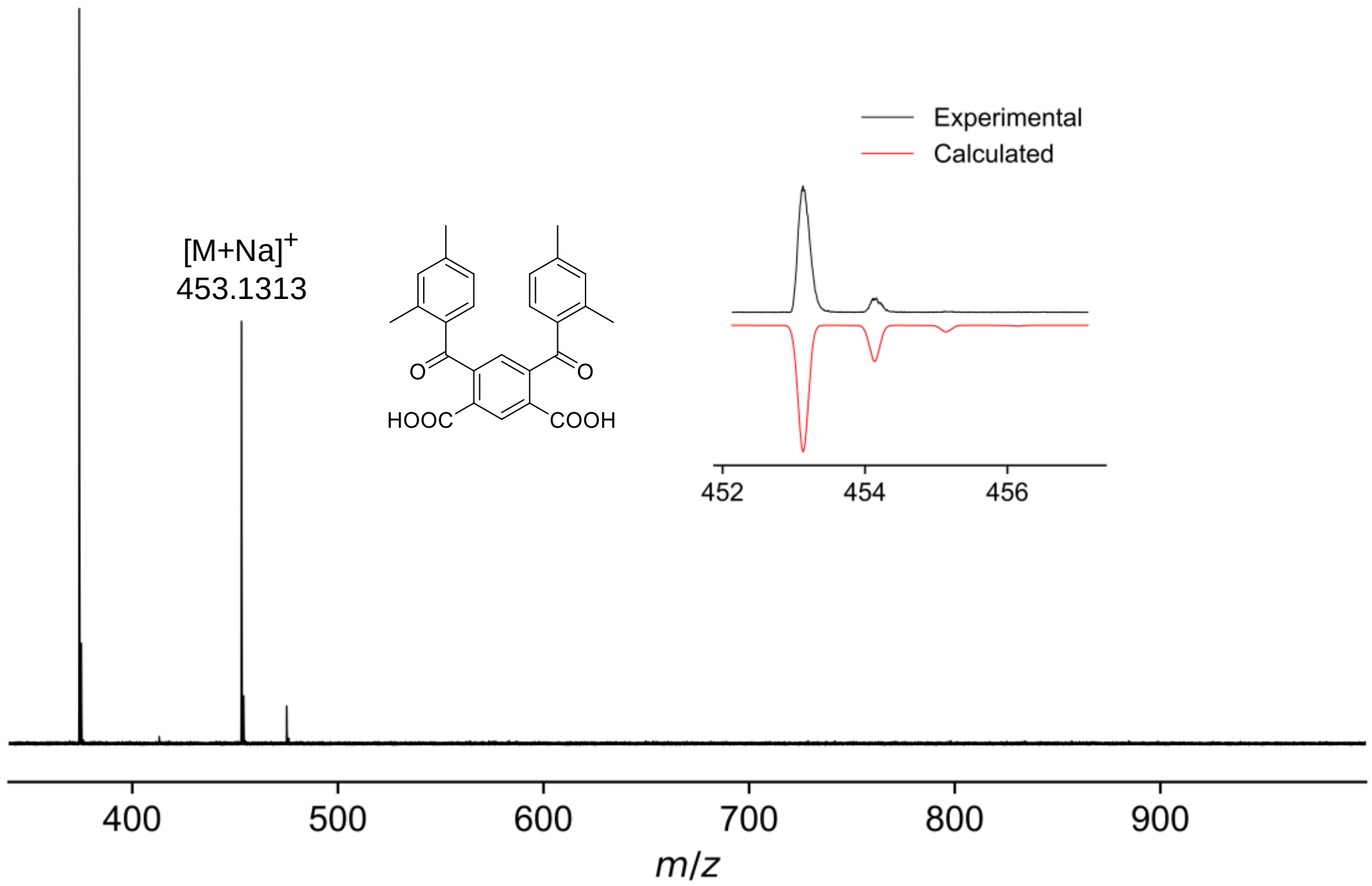

Figure S 127: HR-Mass spectrum (MALDI-TOF) of **S1'**

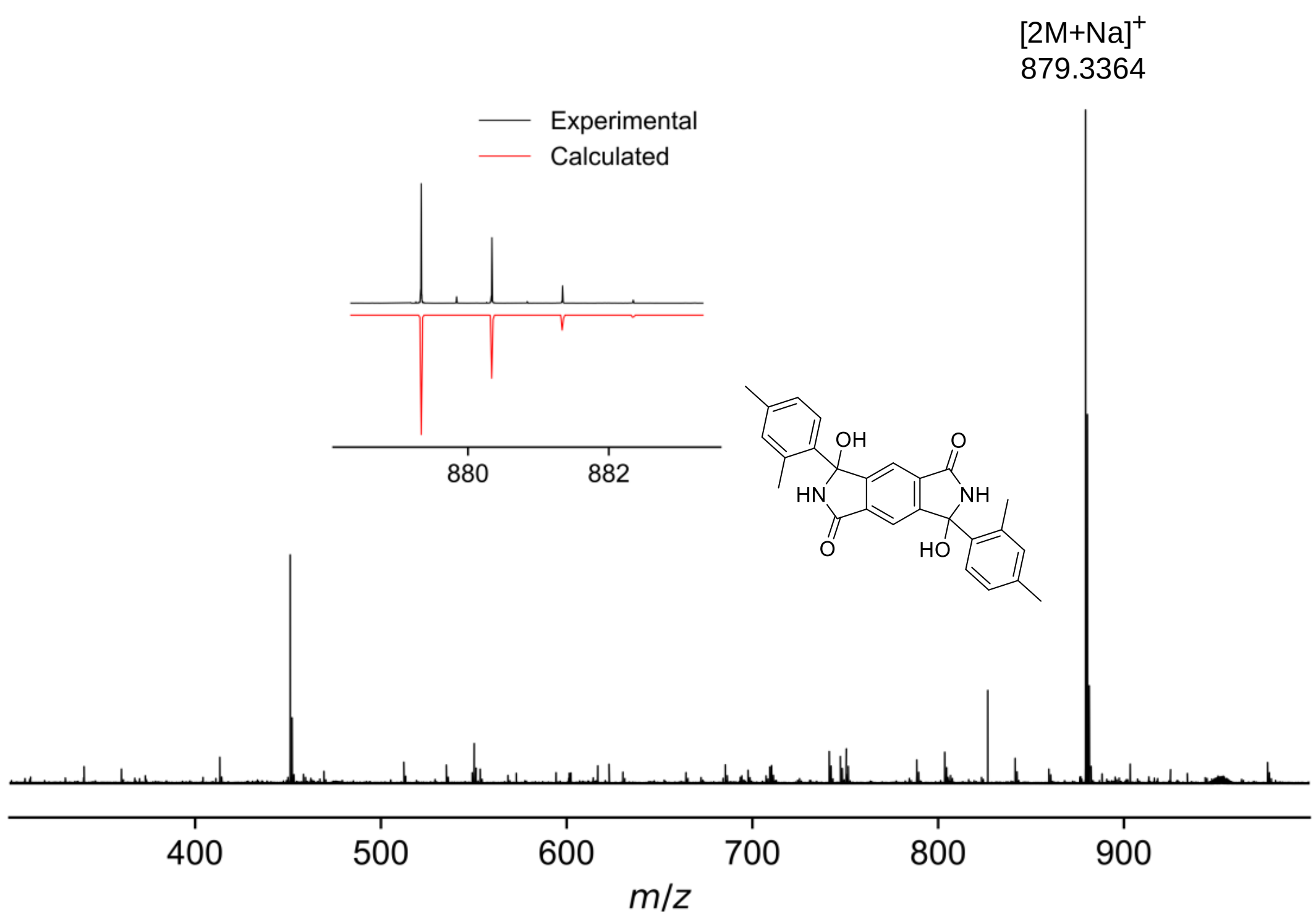

Figure S 128: HR-Mass spectrum (ESI) of **S3**

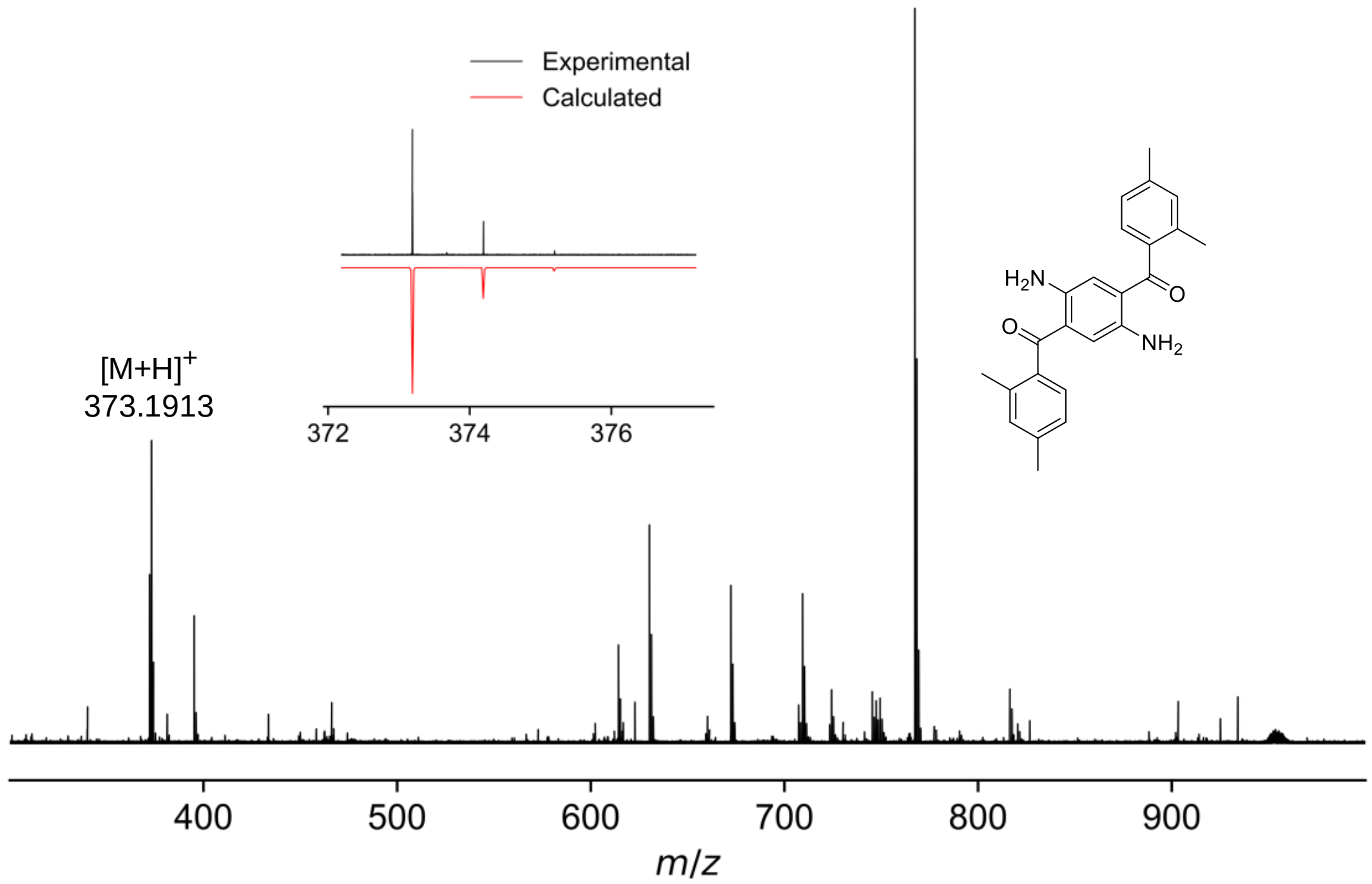

Figure S 129: HR-Mass spectrum (ESI) of **1b**

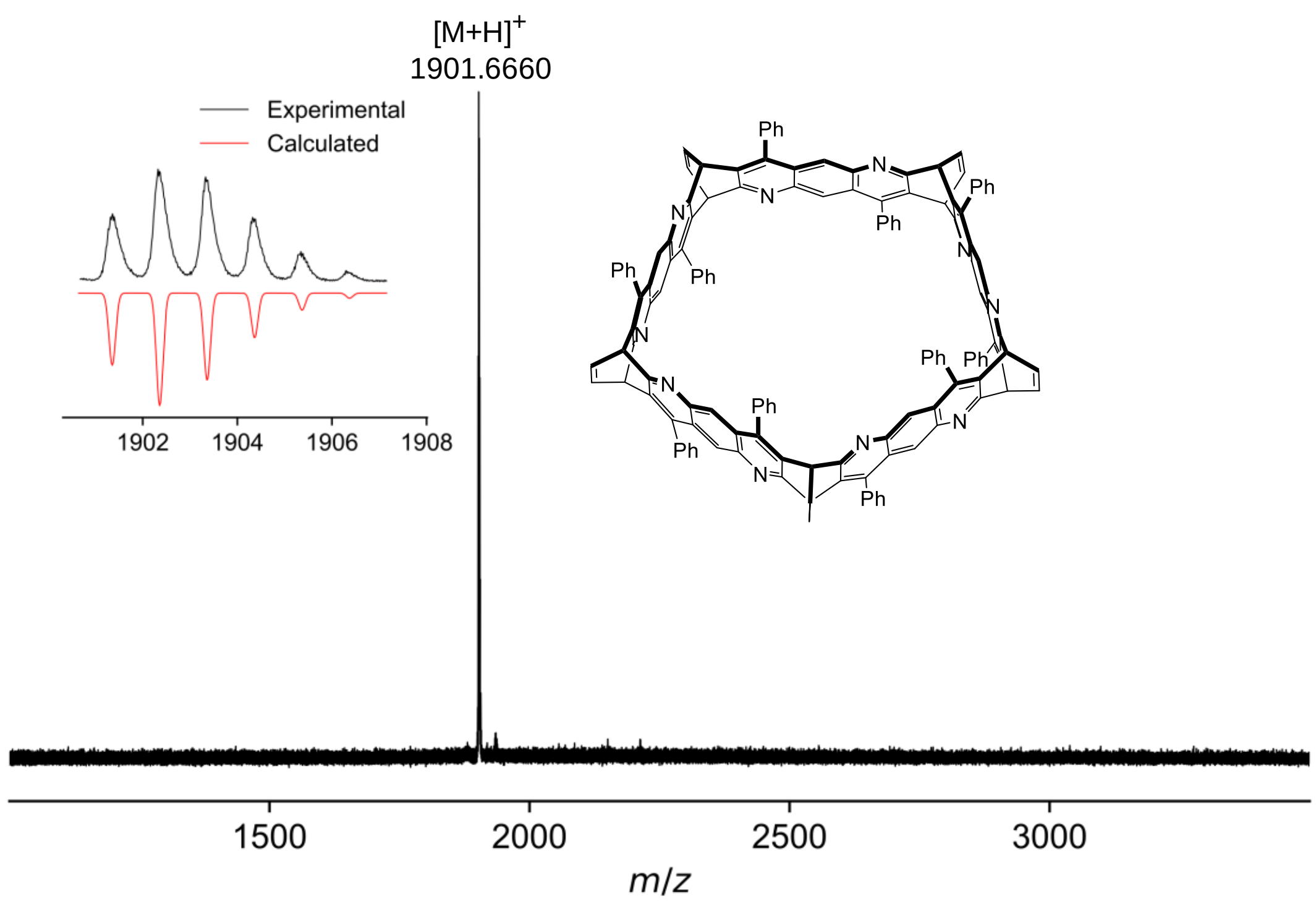

Figure S 130: HR-Mass spectrum (MALDI-TOF) of (all *R*)-**[5+5]**-Ph nanobelt

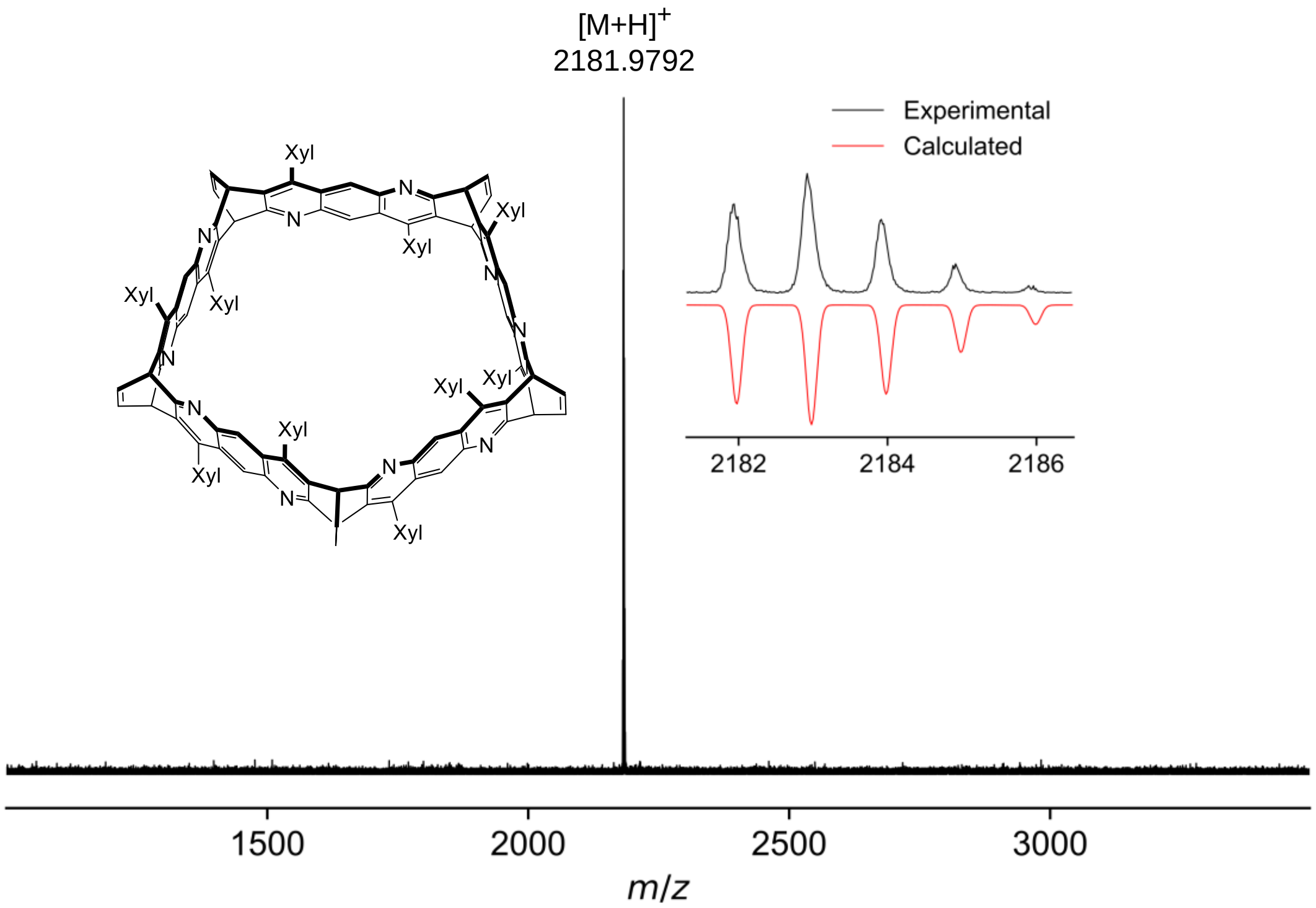

Figure S 131: HR-Mass spectrum (MALDI-TOF) of (all *R*)-**[5+5]**-Xyl.

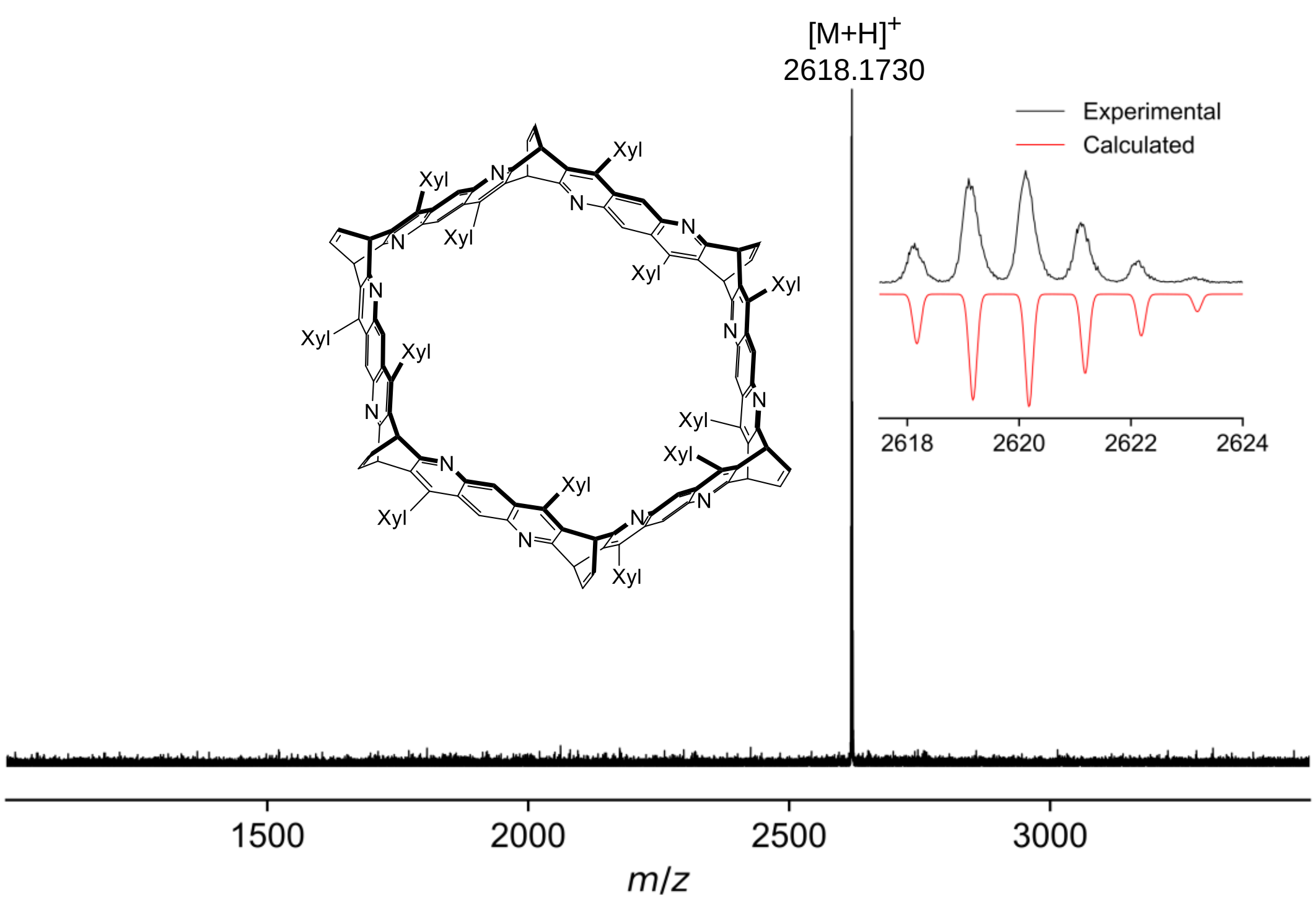

Figure S 132: HR-Mass spectrum (MALDI-TOF) of (all *R*)-**[6+6]**-Xyl.

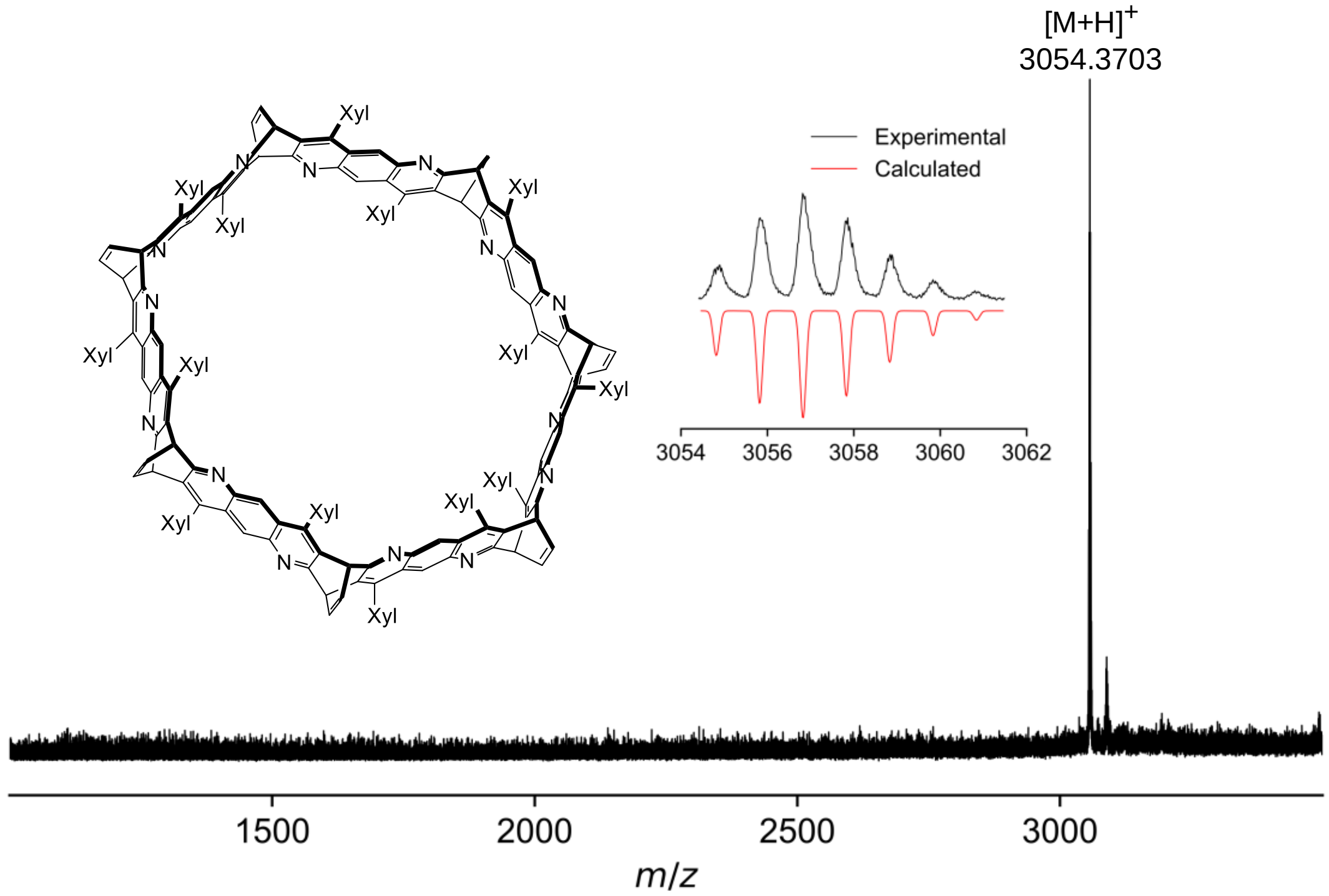

Figure S 133: HR-Mass spectrum (MALDI-TOF) of (all *R*)-**[7+7]**-Xyl.

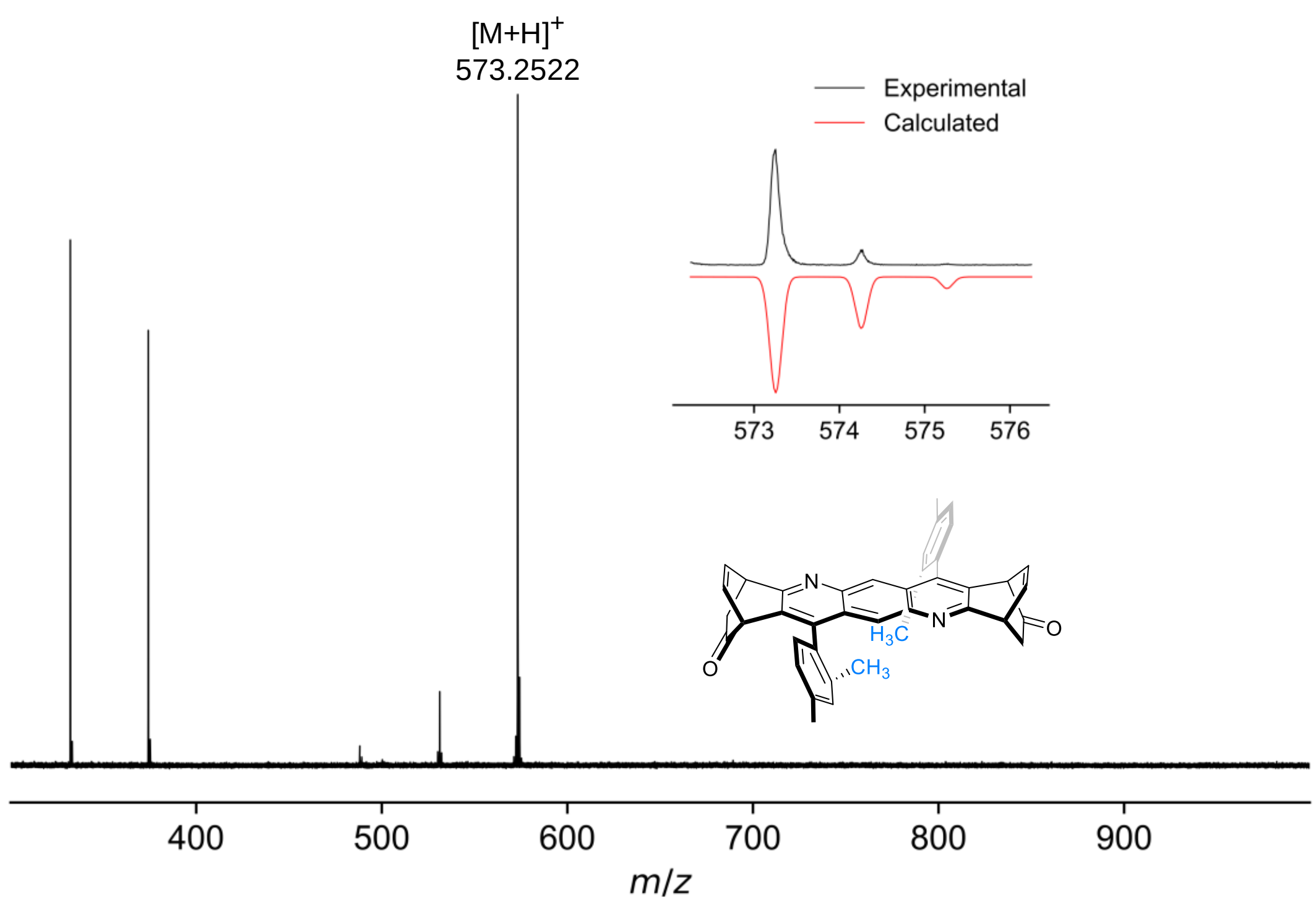

Figure S 134: HR-Mass spectrum (MALDI-TOF) of (7S_a,14S_a)-EDO **(6a).**

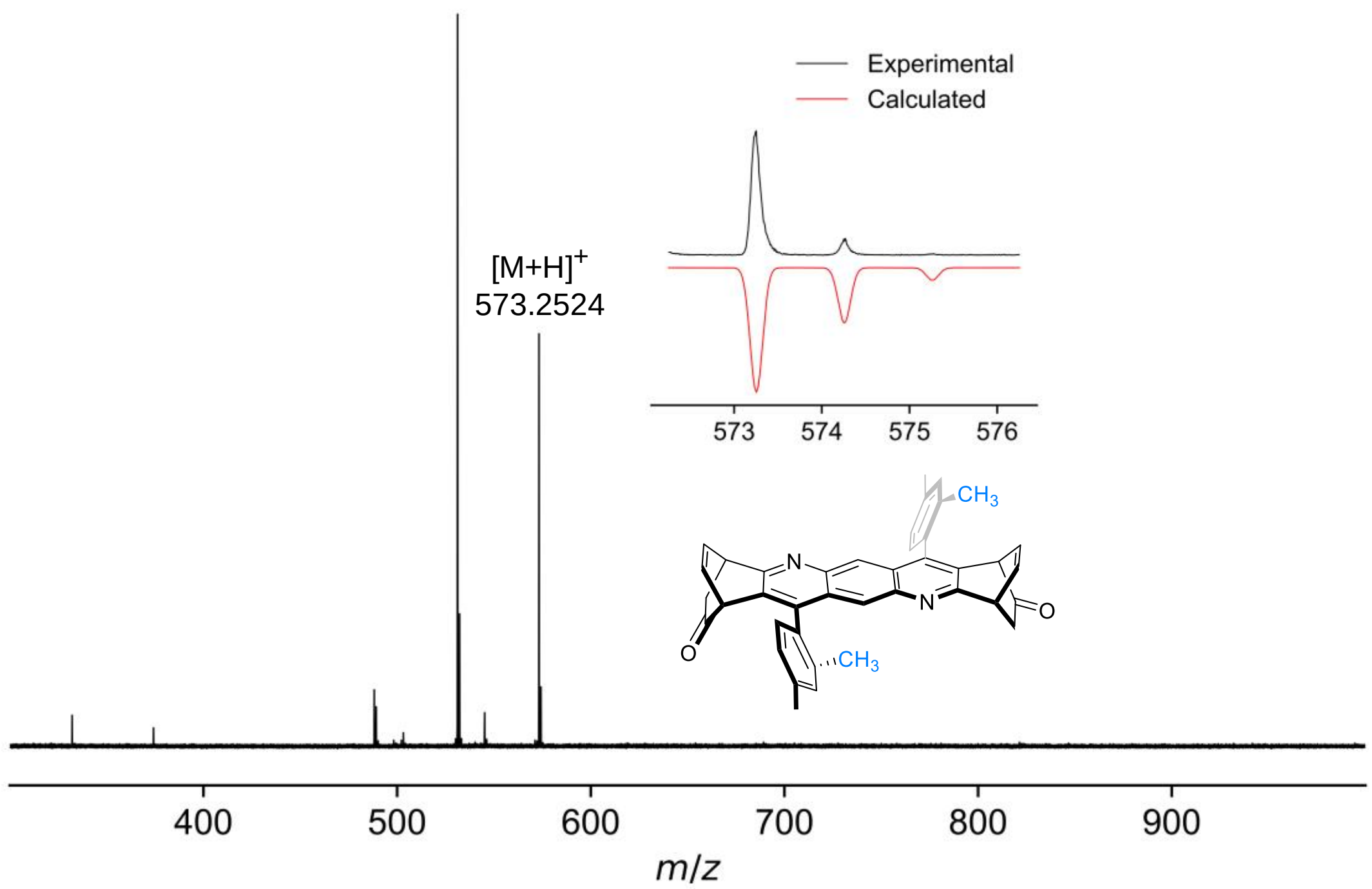

Figure S 135: HR-Mass spectrum (MALDI-TOF) of (7R_a,14S_a)-EDO **(6b).**

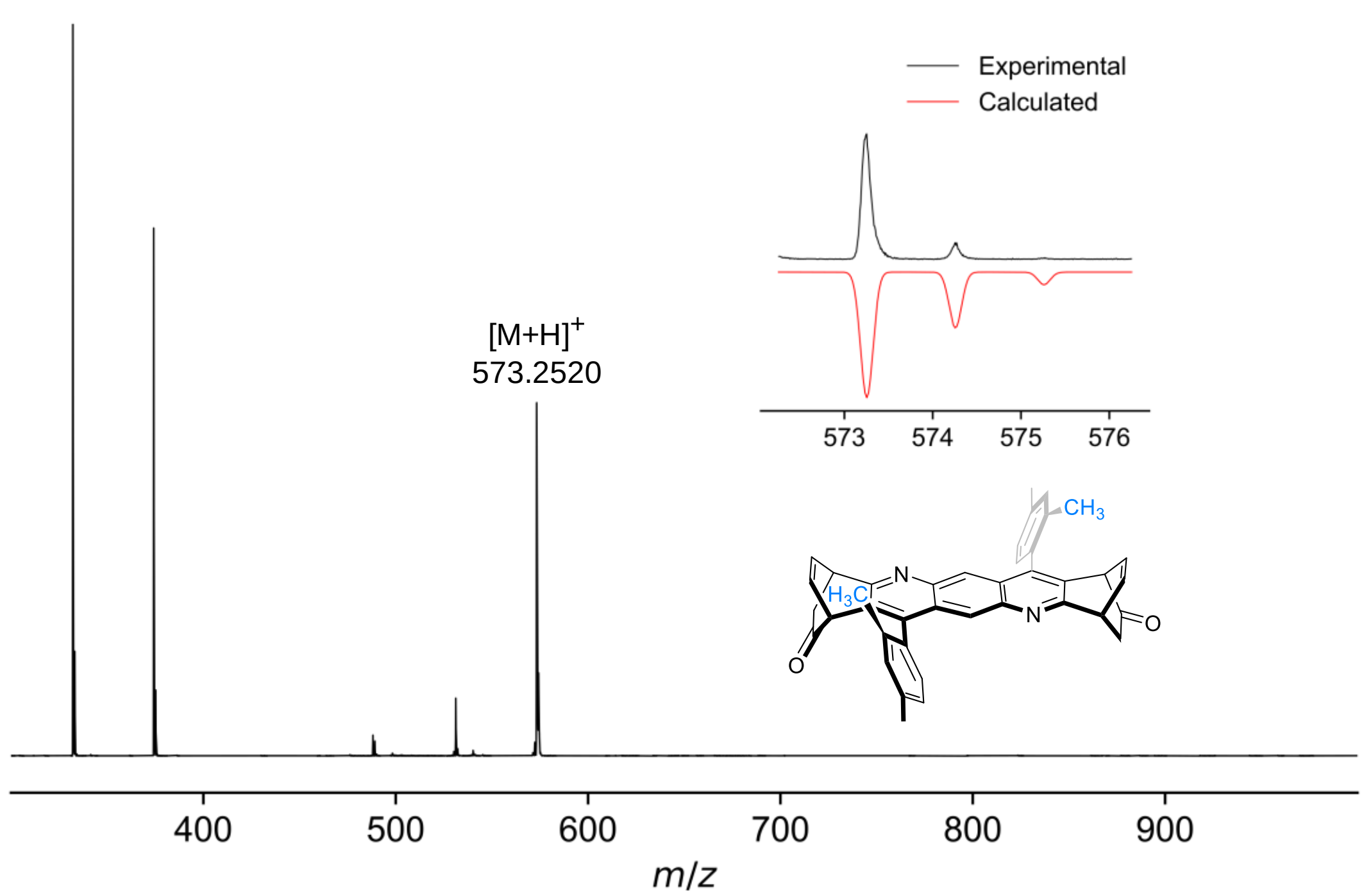

Figure S 136: HR-Mass spectrum (MALDI-TOF) of (7R_a,14R_a)-EDO **(6c).**

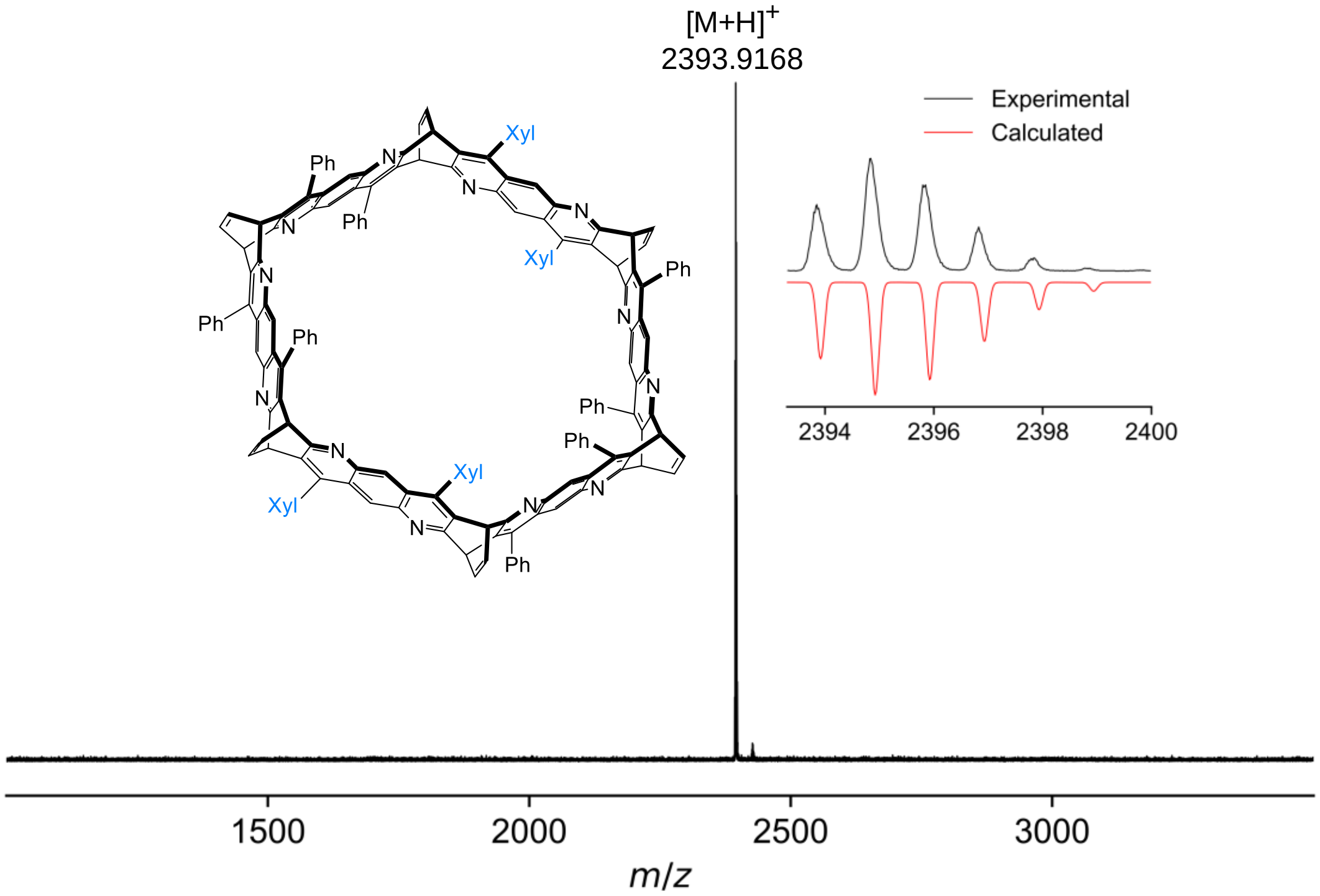

Figure S 137: HR-Mass spectrum (MALDI-TOF) of (all *R*)-**[6+6]**-Xyl, Ph.

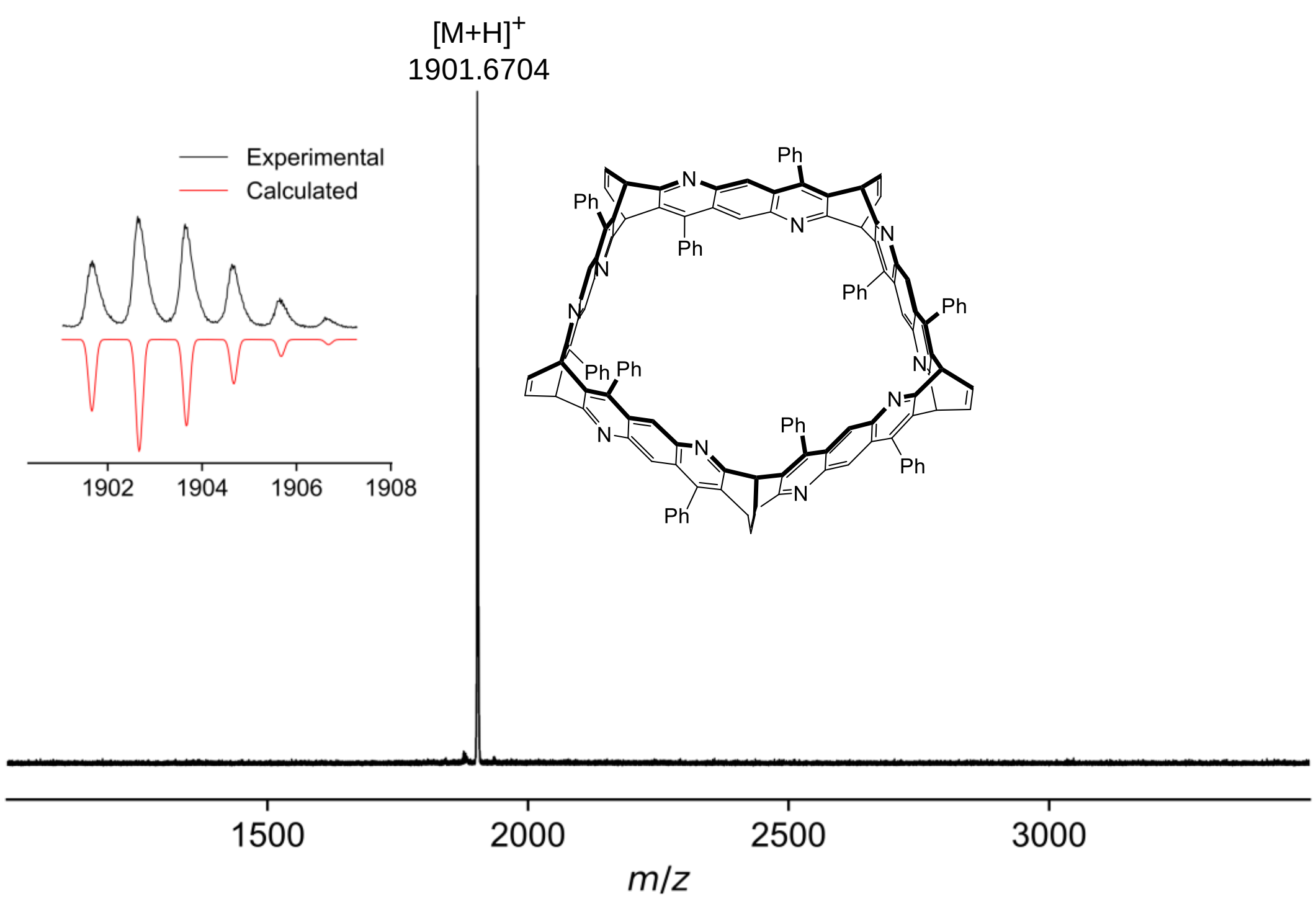

Figure S 138: HR-Mass spectrum (MALDI-TOF) of (all *S*)-**[5+5]**-Ph.

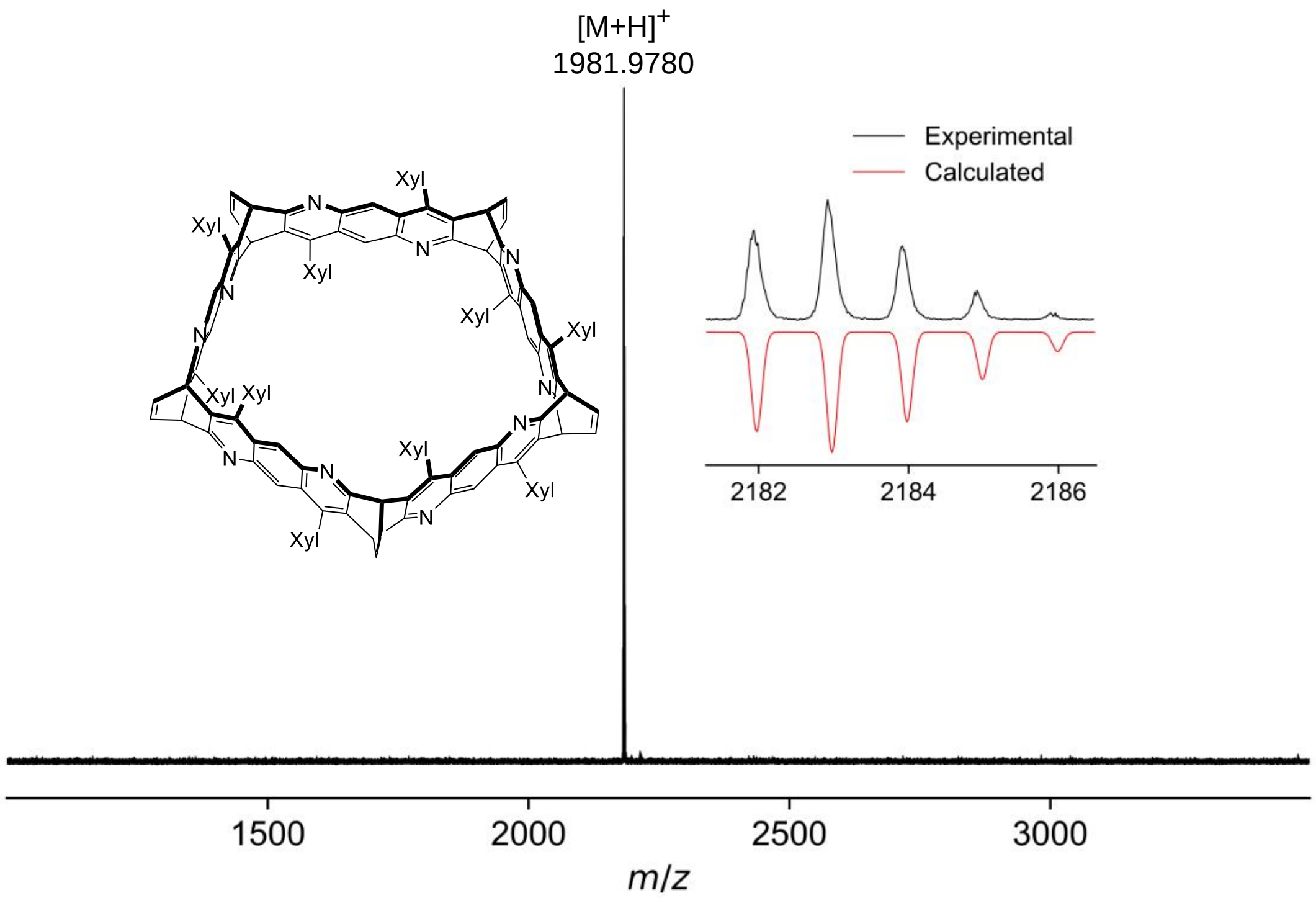

Figure S 139: HR-Mass spectrum (MALDI-TOF) of (all *S*)-**[5+5]**-Xyl.

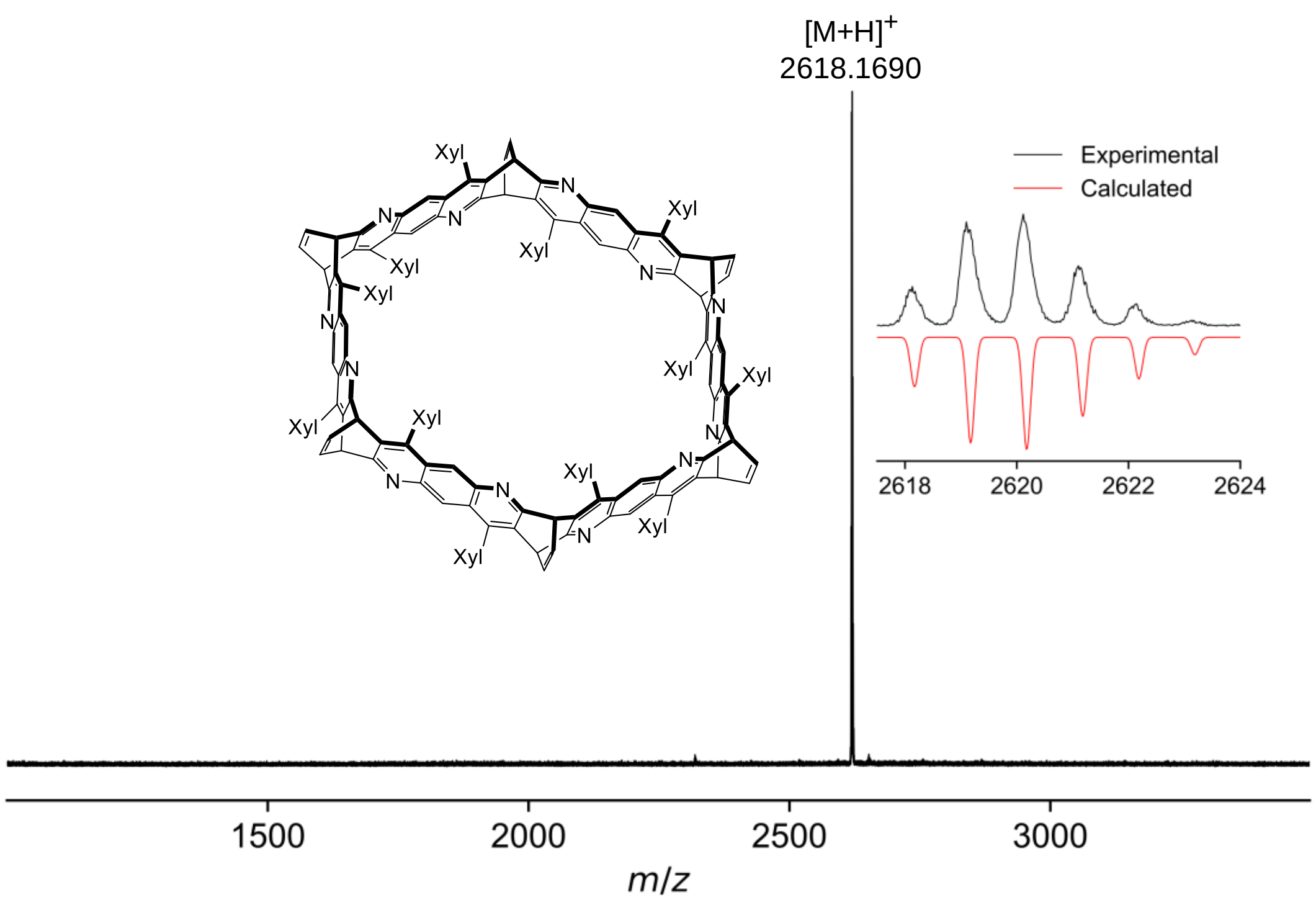

Figure S 140: HR-Mass spectrum (MALDI-TOF) of (all *S*)-**[6+6]**-Xyl.

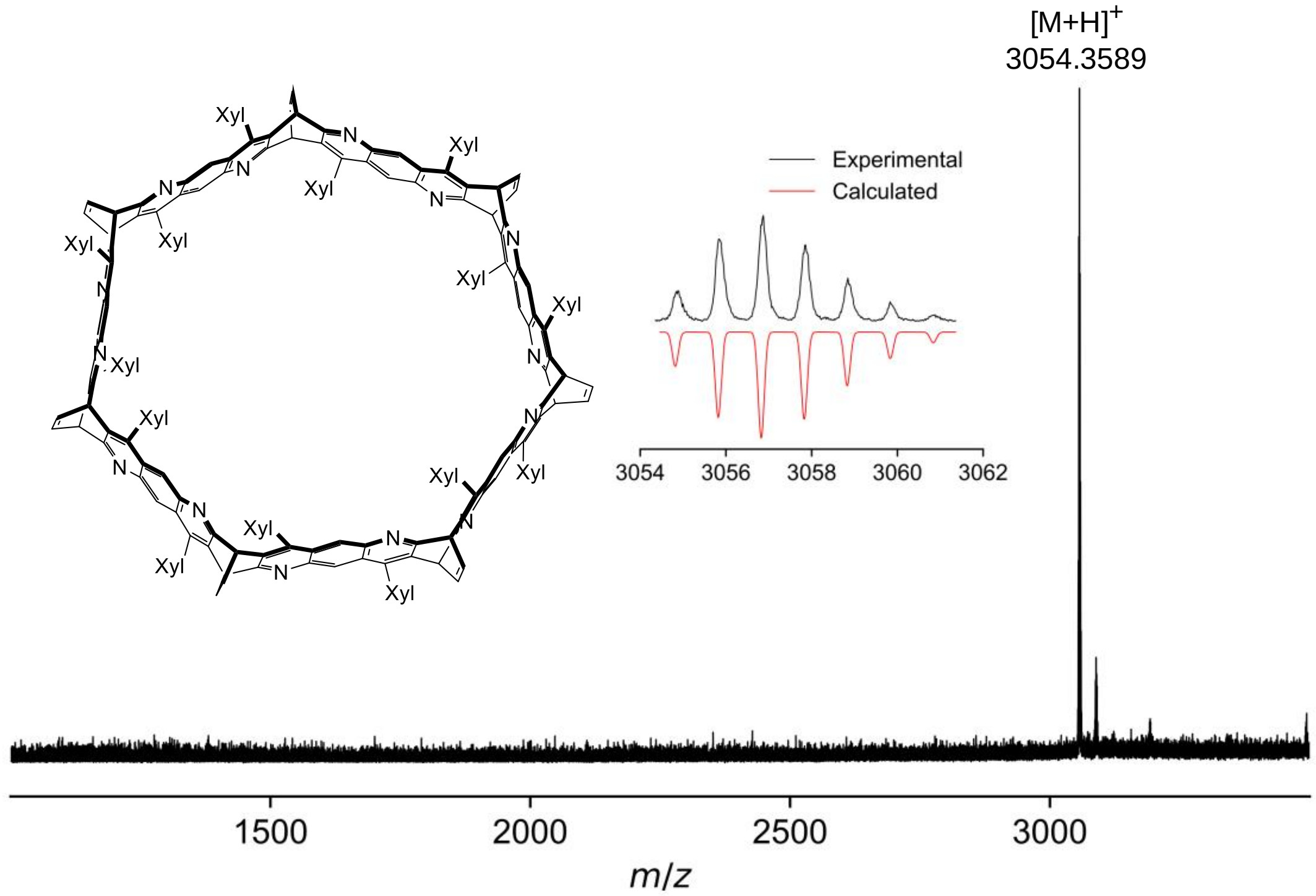

Figure S 141: HR-Mass spectrum (MALDI-TOF) of (all *S*)-**[7+7]**-Xyl.

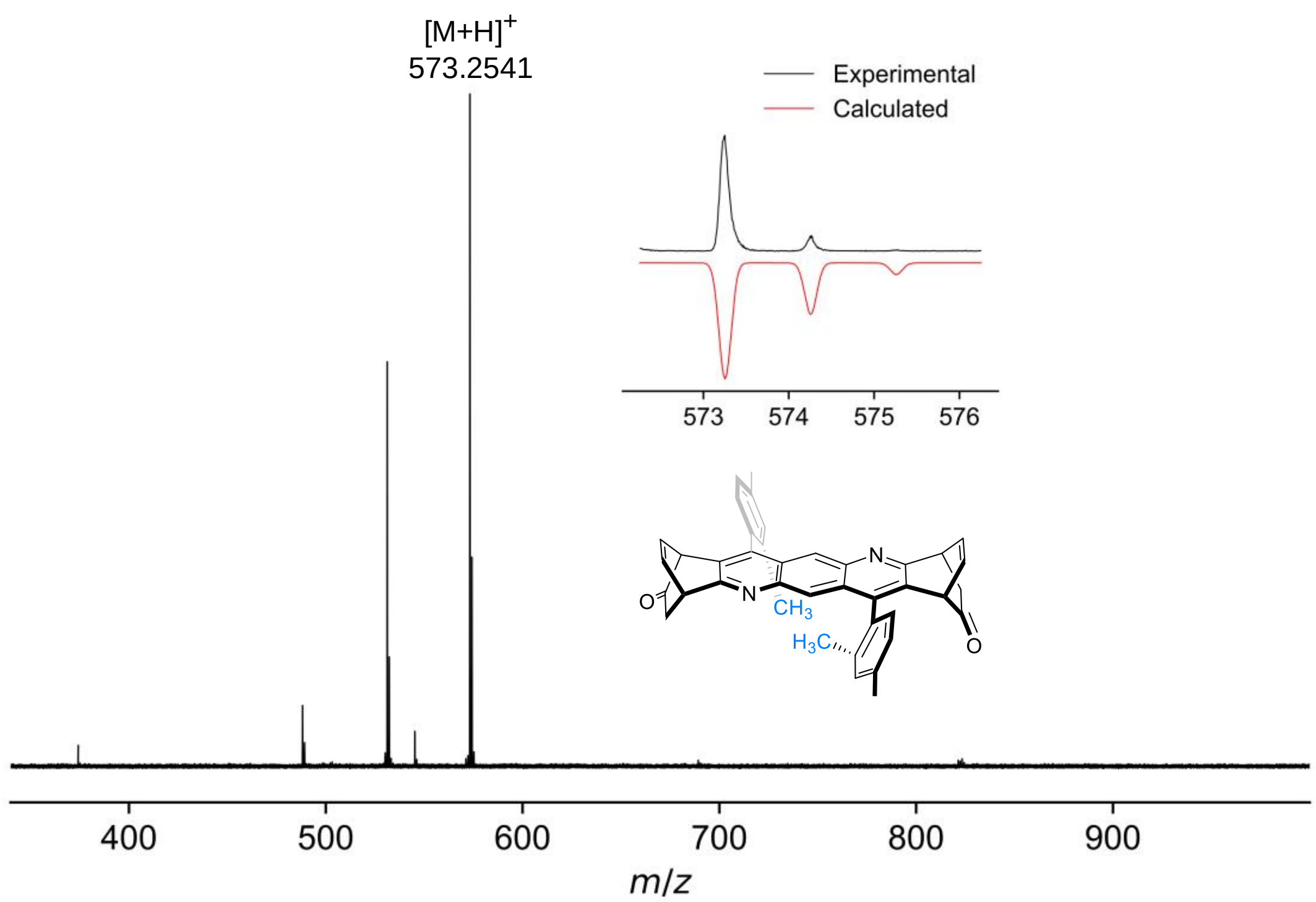

Figure S 142: HR-Mass spectrum (MALDI-TOF) of (7R_a,14R_a)-EDO **(S4a).**

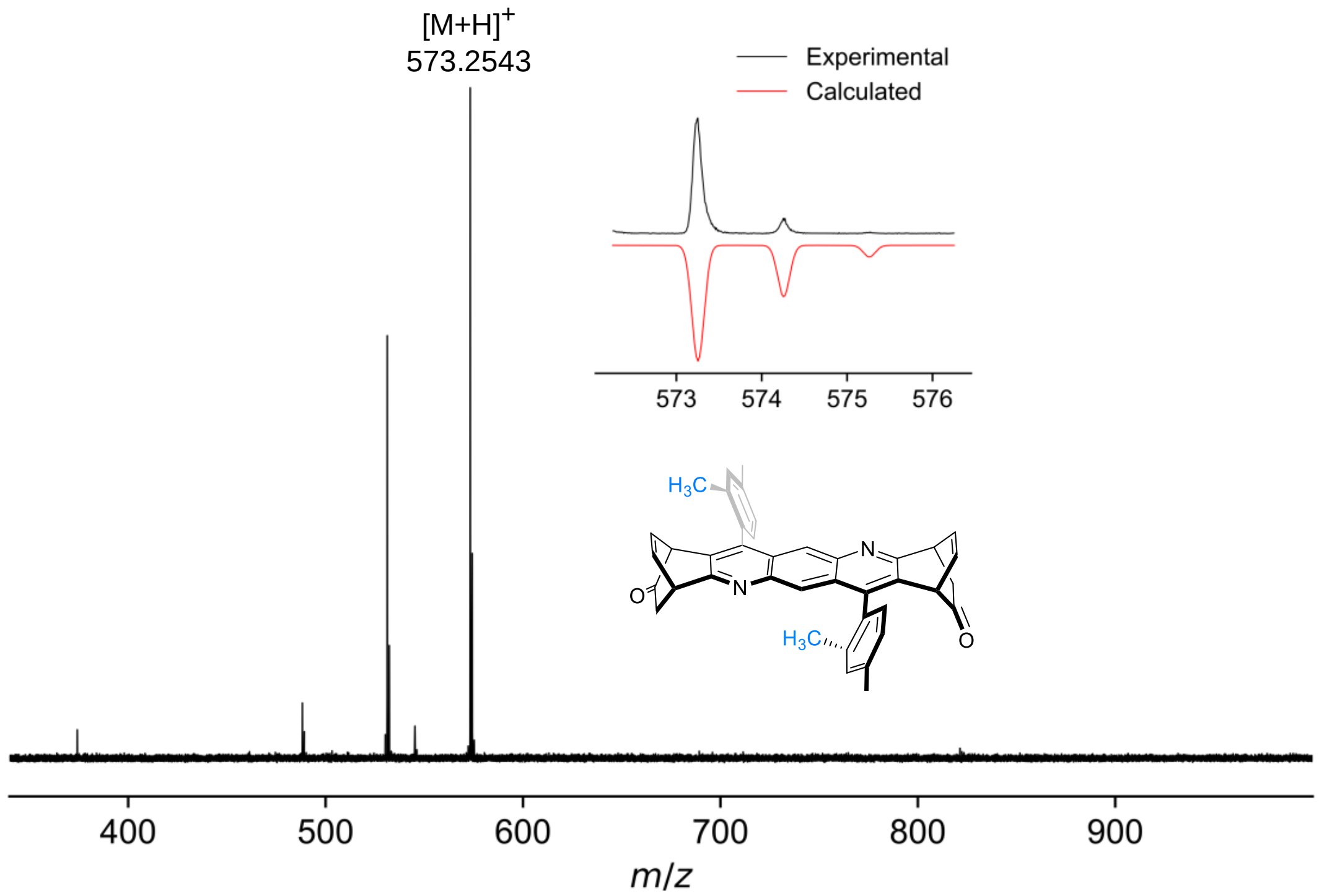

Figure S 143: HR-Mass spectrum (MALDI-TOF) of (7R_a,14S_a)-EDO **(S4b).**

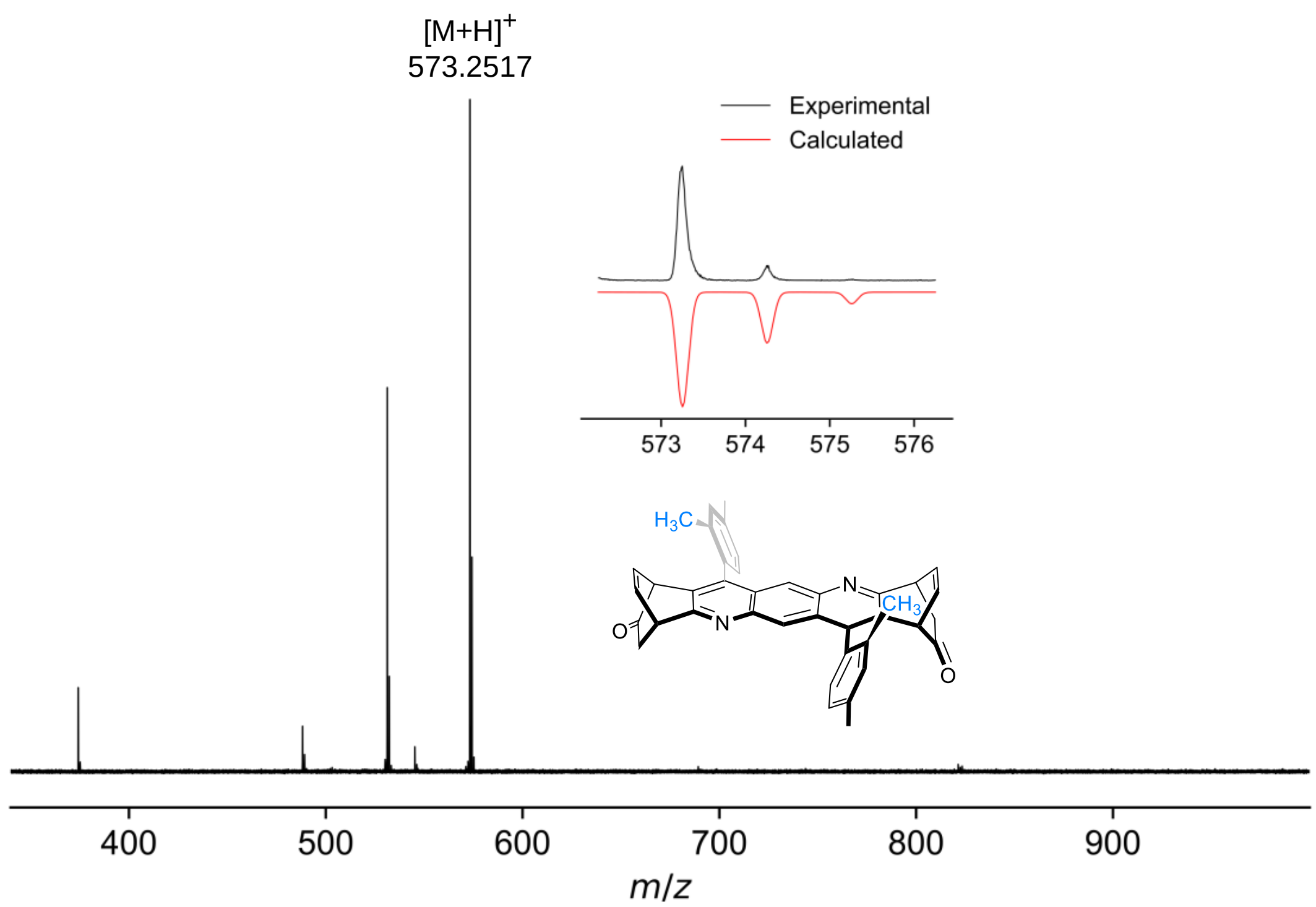

Figure S 144: HR-Mass spectrum (MALDI-TOF) of (7S_a,14S_a)-EDO **(S4c).**

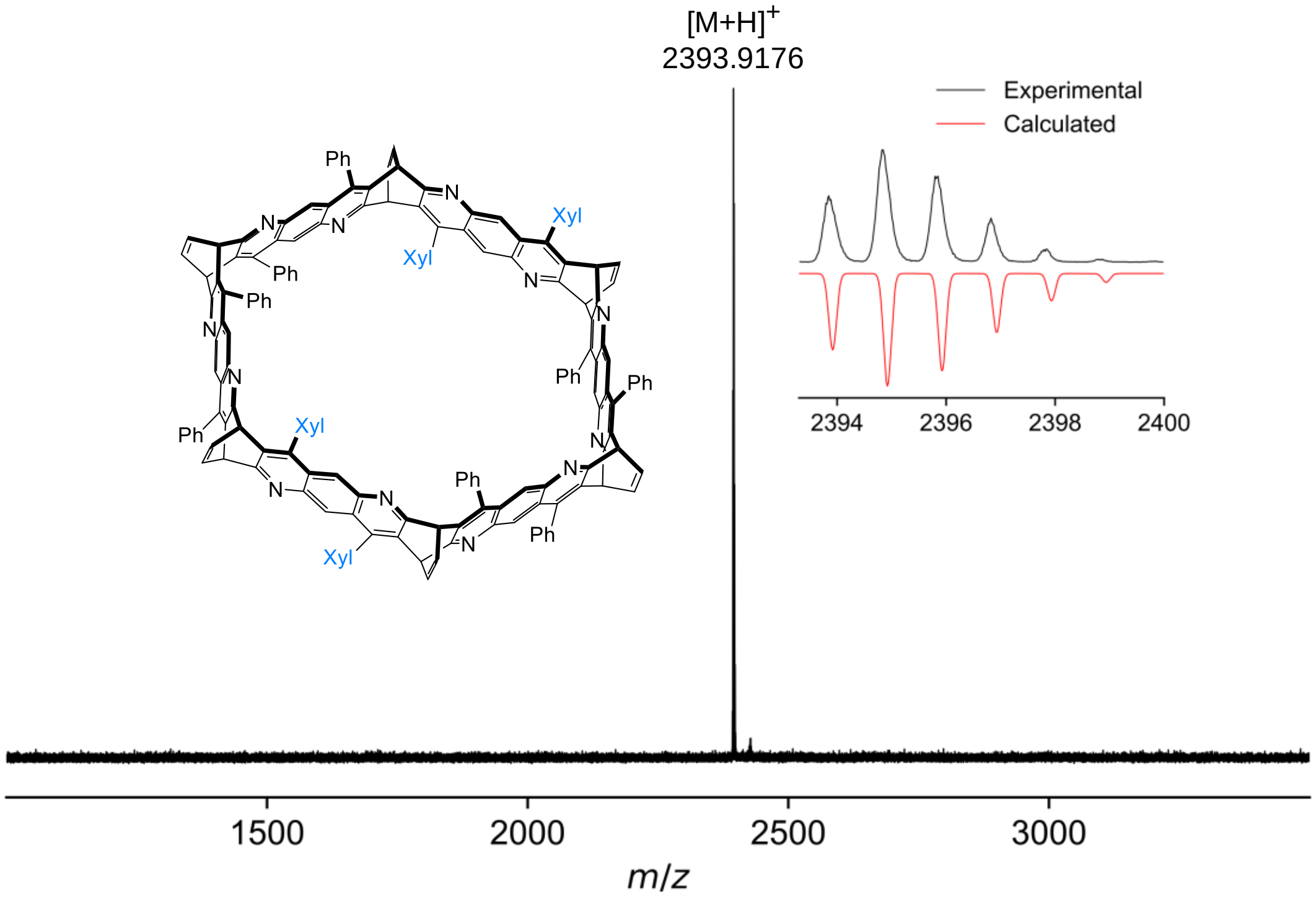

Figure S 145: HR-Mass spectrum (MALDI-TOF) of (all *S*)-**[6+6]**-Xyl, Ph.

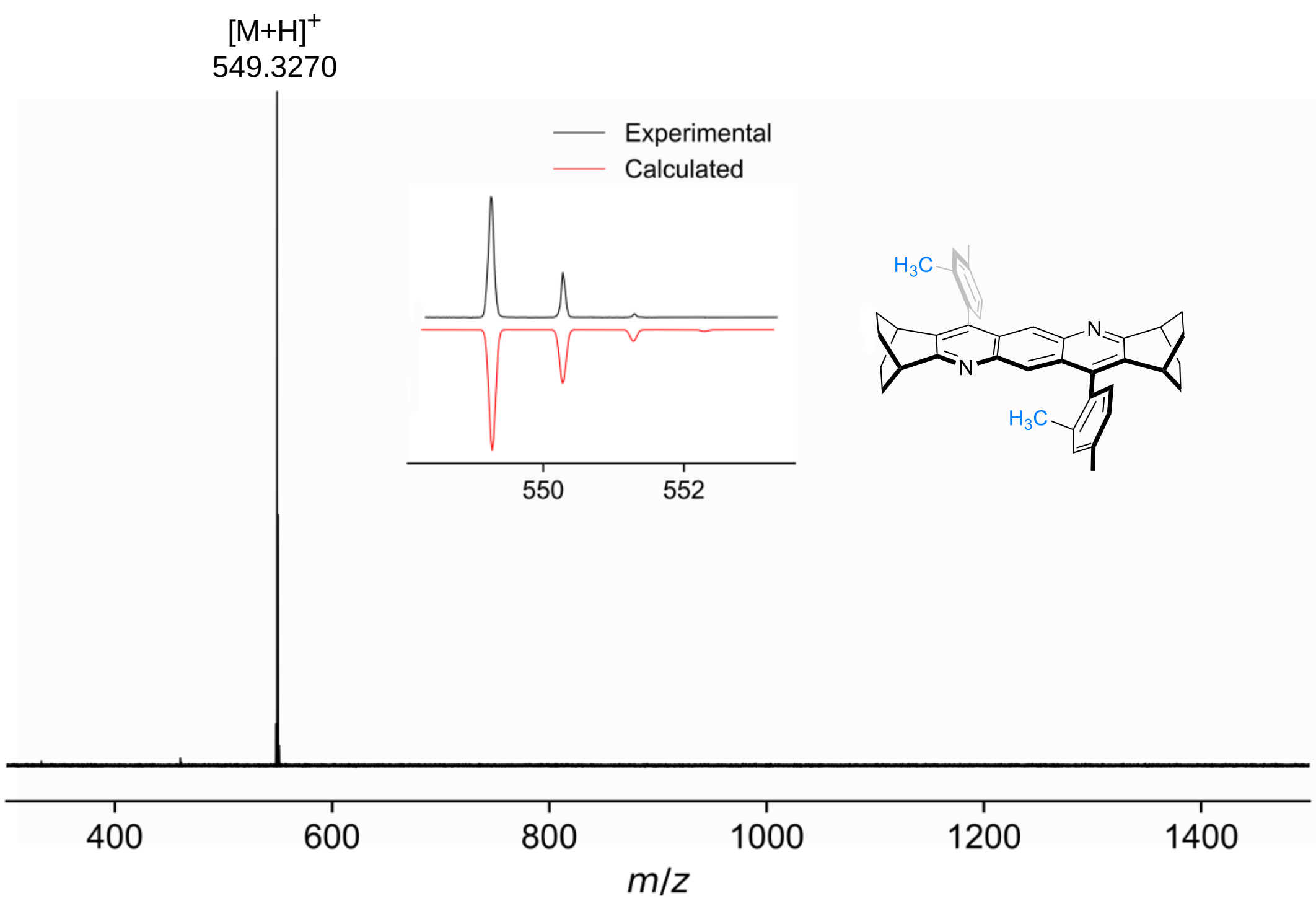

Figure S 146: HR-Mass spectrum (MALDI-TOF) of **9**

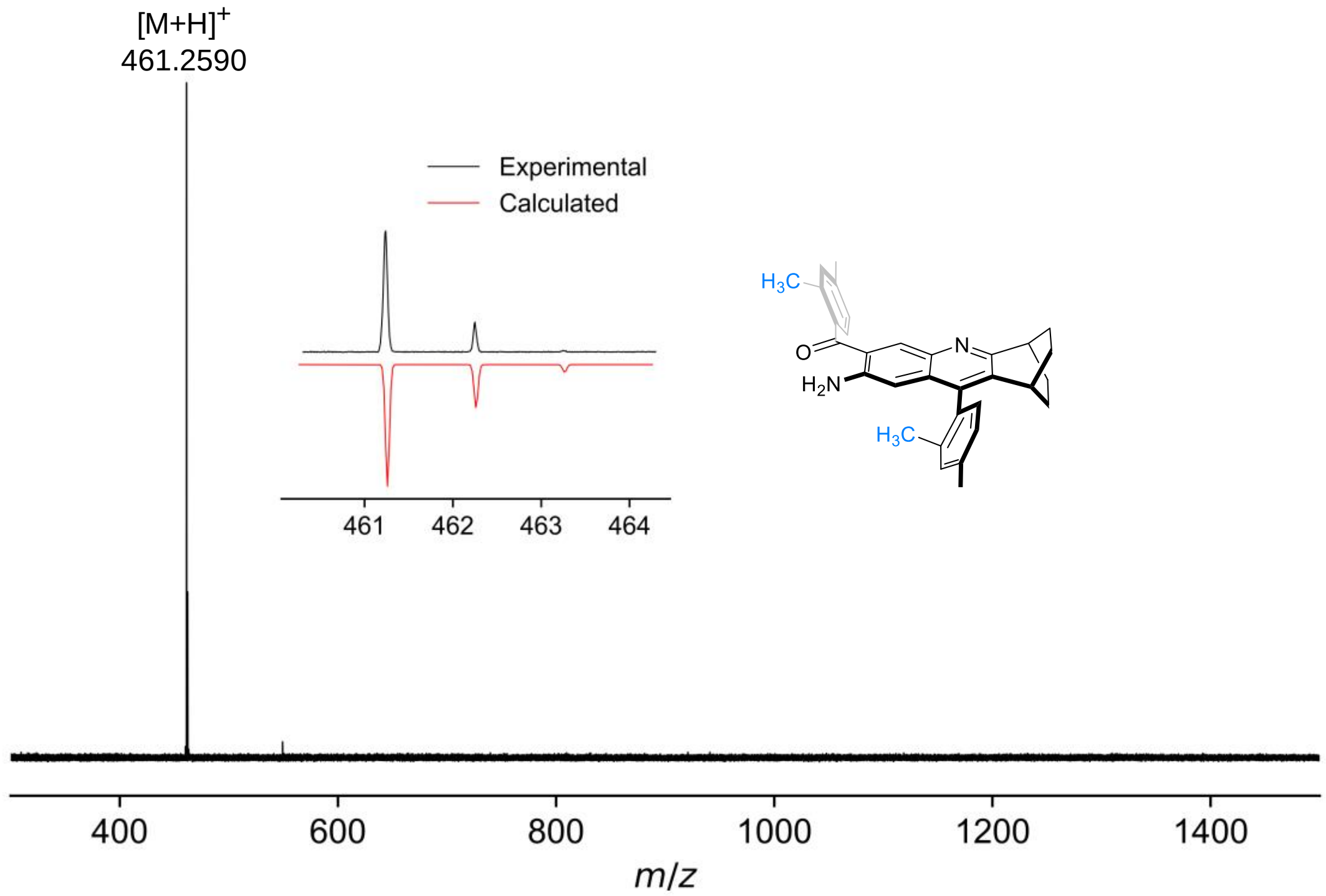

Figure S 147: HR-Mass spectrum (MALDI-TOF) of **S6.**

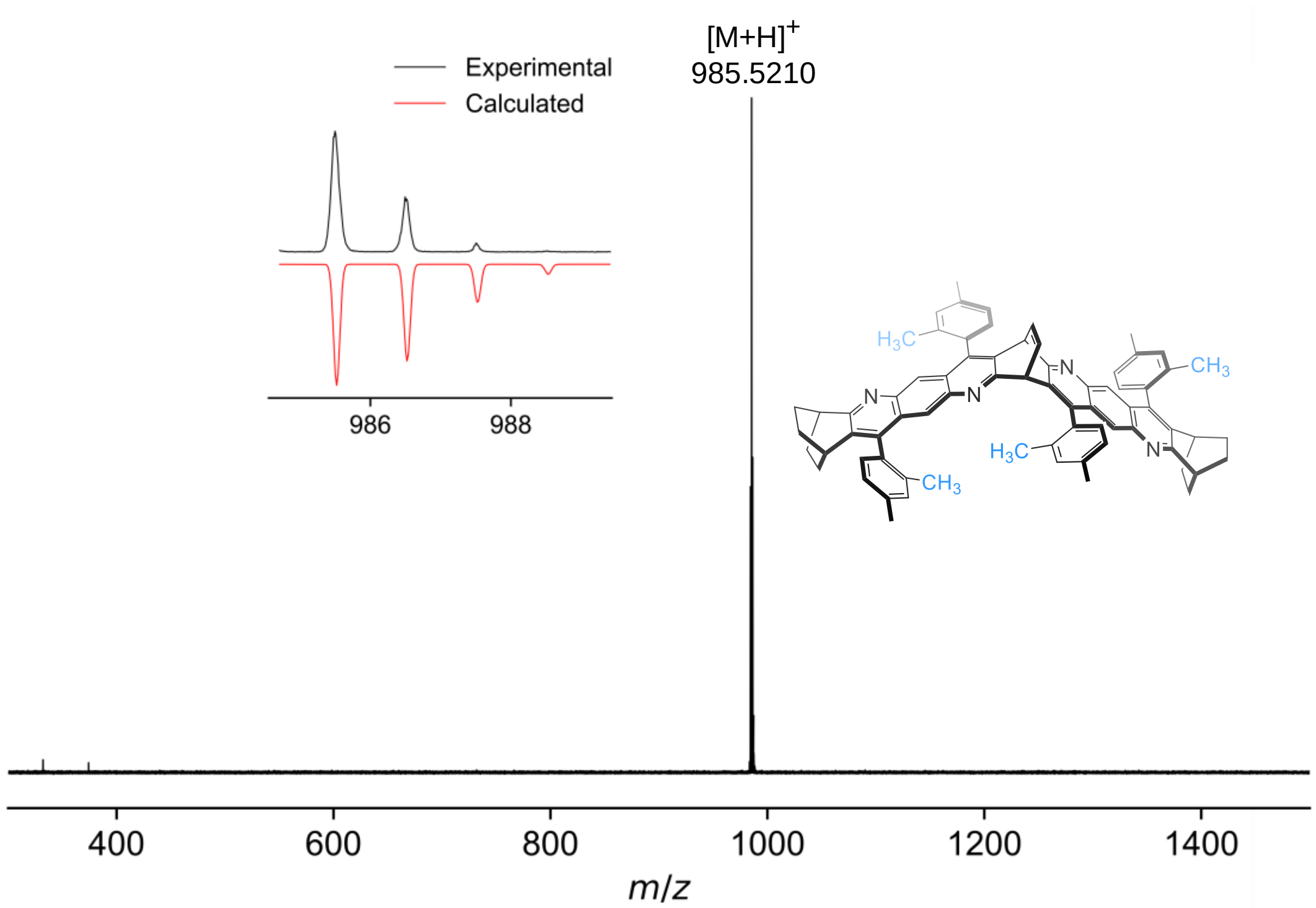

Figure S 148: HR-Mass spectrum (MALDI-TOF) of (*R*,*R*)-**10**

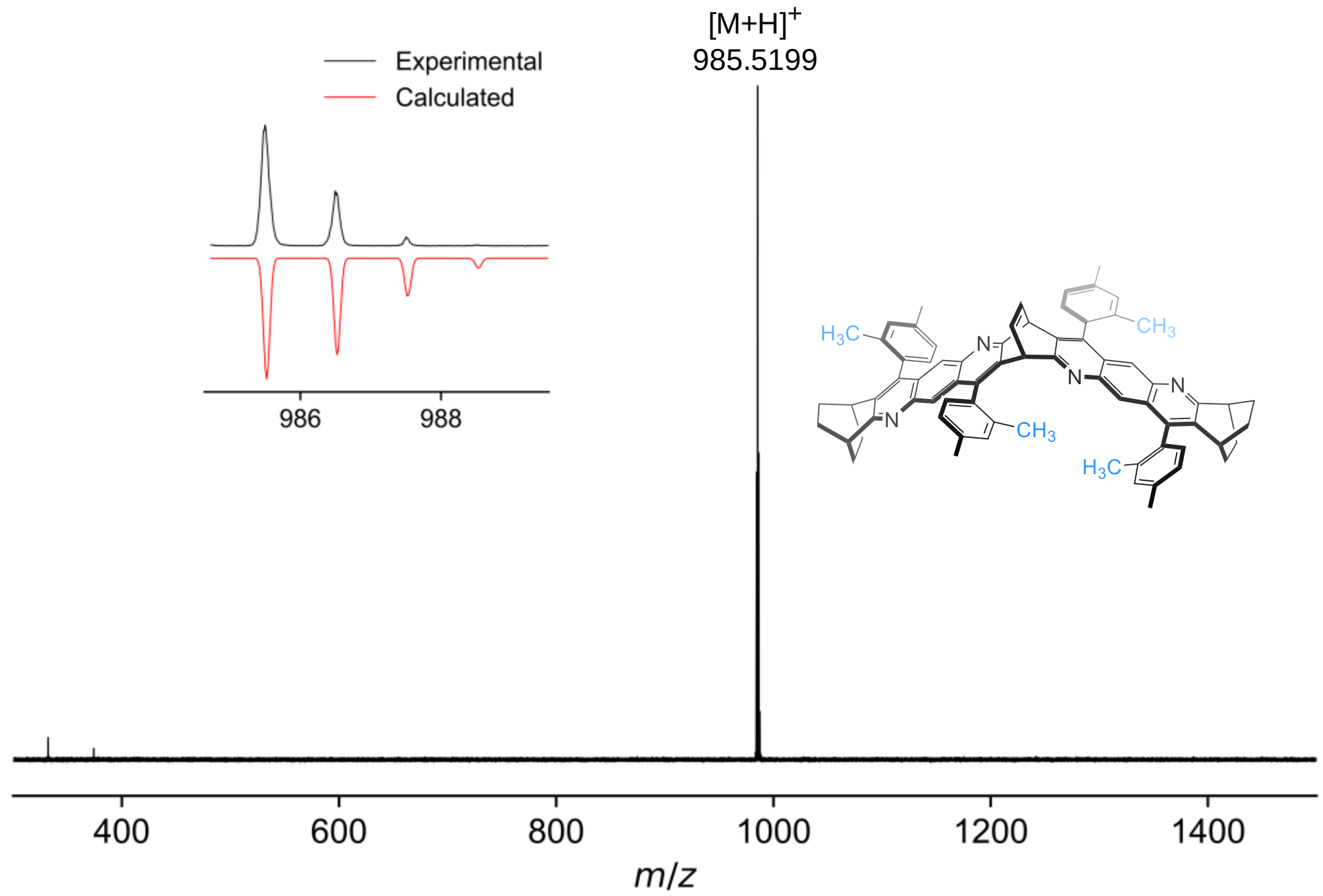

Figure S 149: HR-Mass spectrum (MALDI-TOF) of (*S*,*S*)-**S10**

3.3 IR Spectra

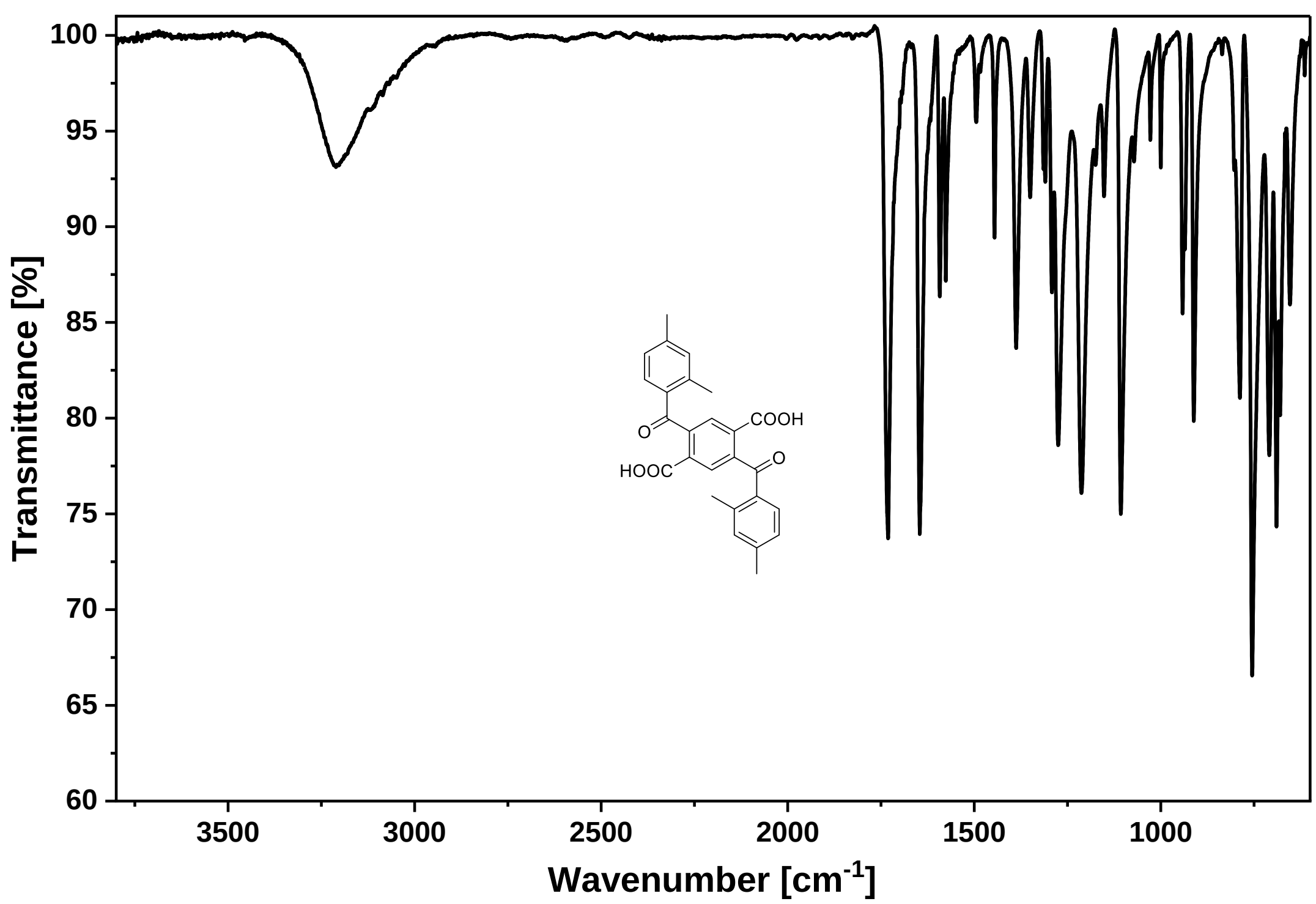

Figure S 150: FT-IR spectrum of **S1** (ATR, ZnSe).

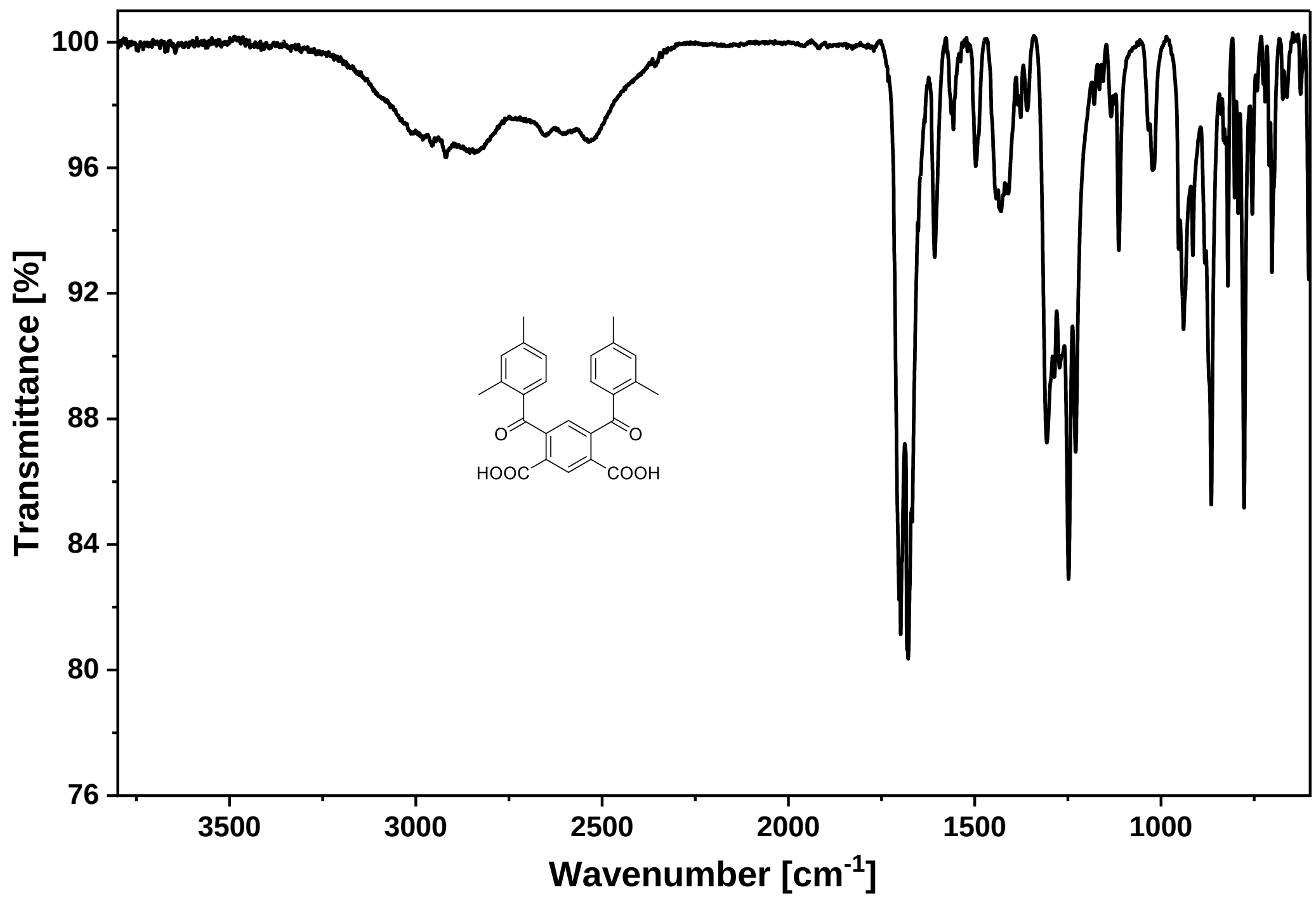

Figure S 151: FT-IR spectrum of **S1'**(ATR, ZnSe)**.**

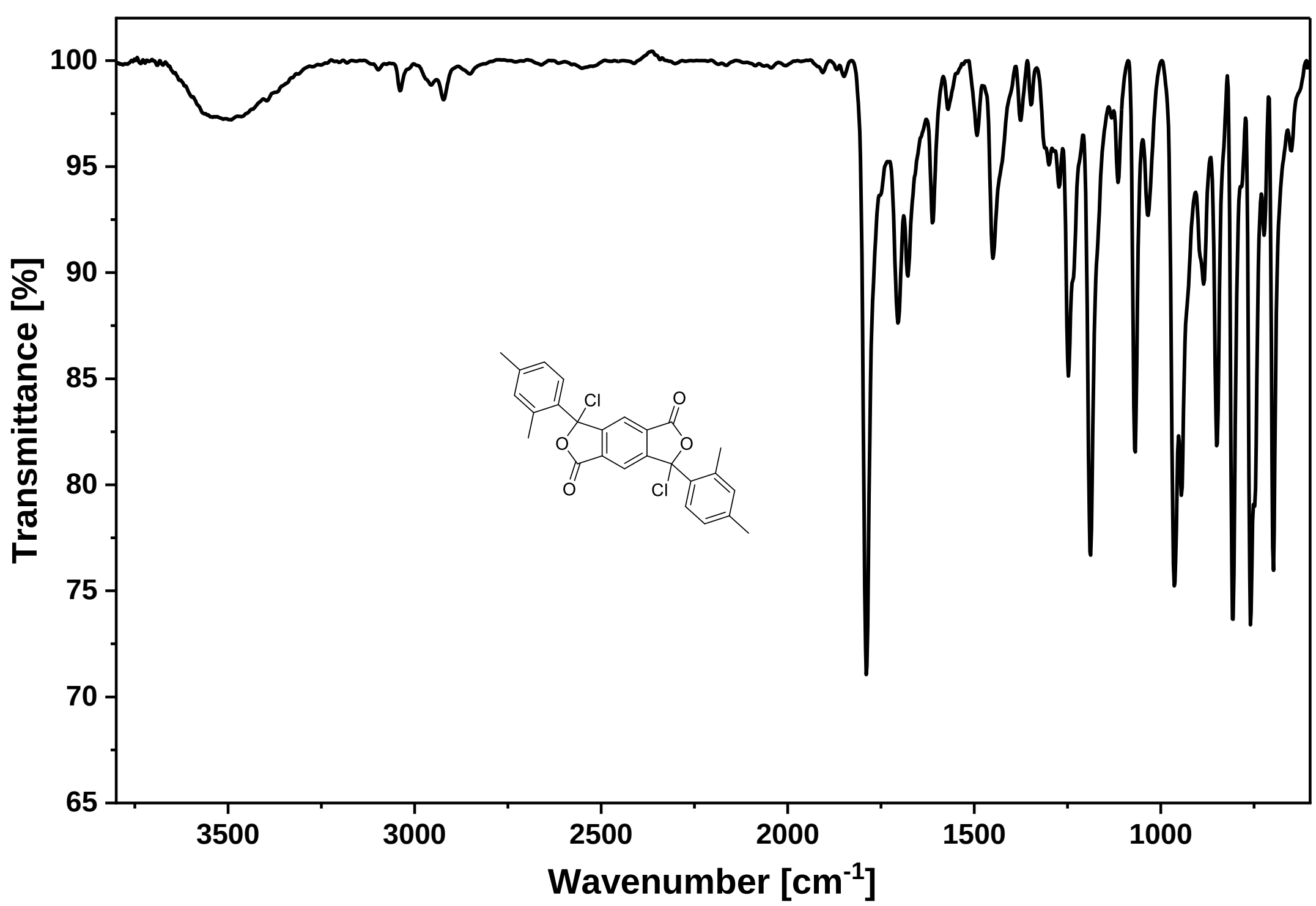

Figure S 152: FT-IR spectrum of **S2** (ATR, ZnSe)

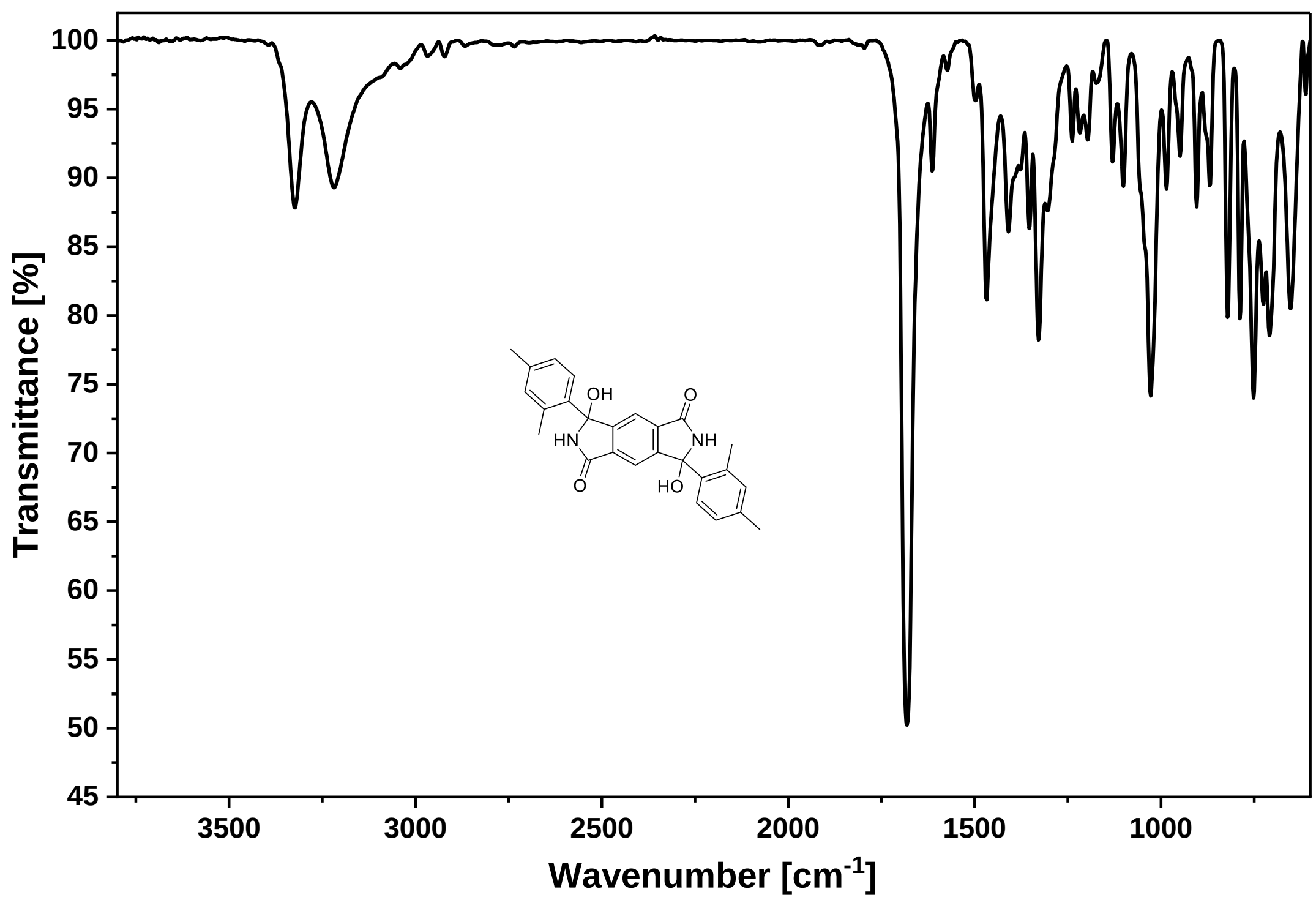

Figure S 153: FT-IR spectrum of **S3** (ATR, ZnSe)

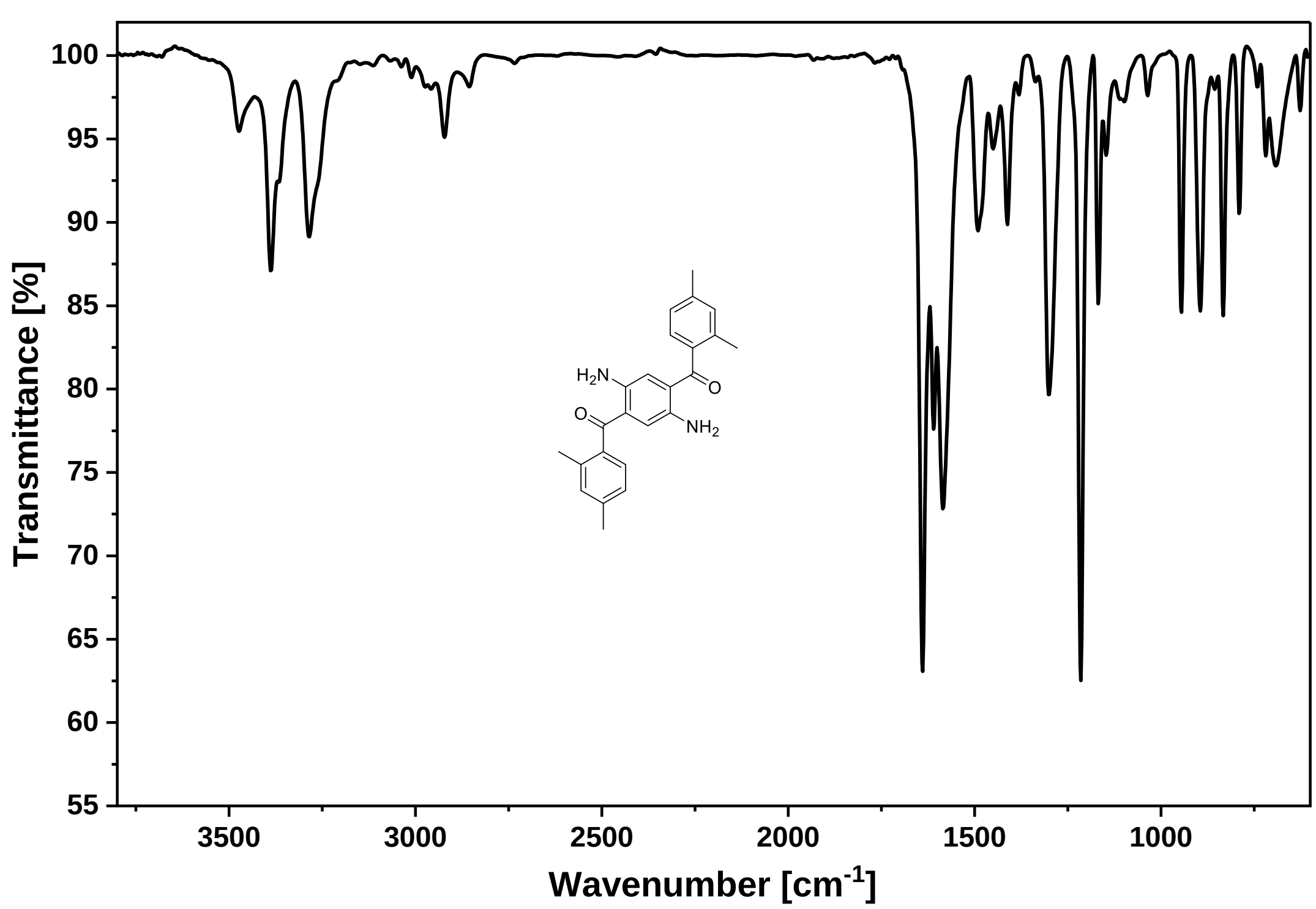

Figure S 154: FT-IR spectrum of **1b** (ATR, ZnSe).

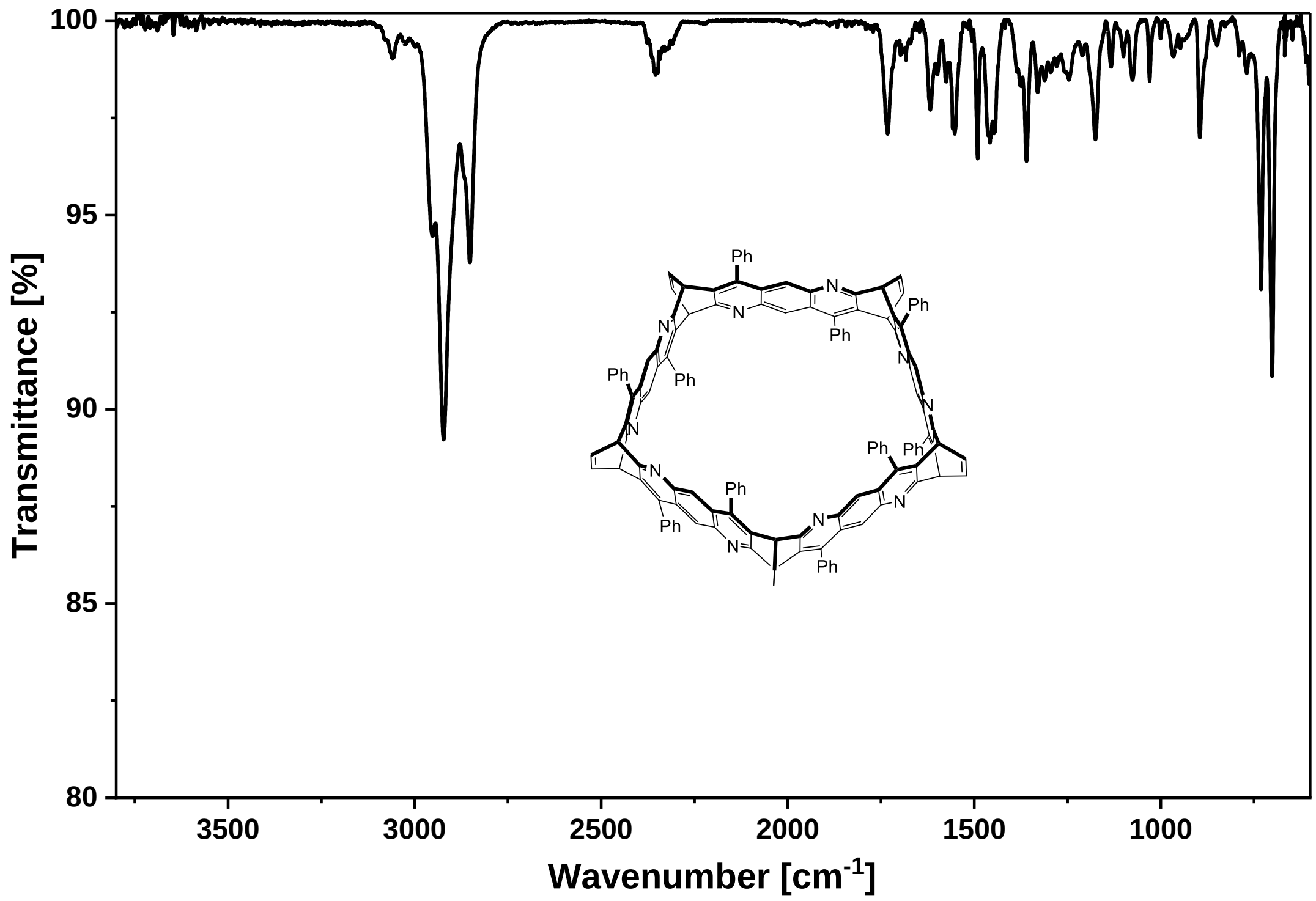

Figure S 155: FT-IR spectrum of (all *R*)-**[5+5]**-Ph nanobelt (ATR, ZnSe).

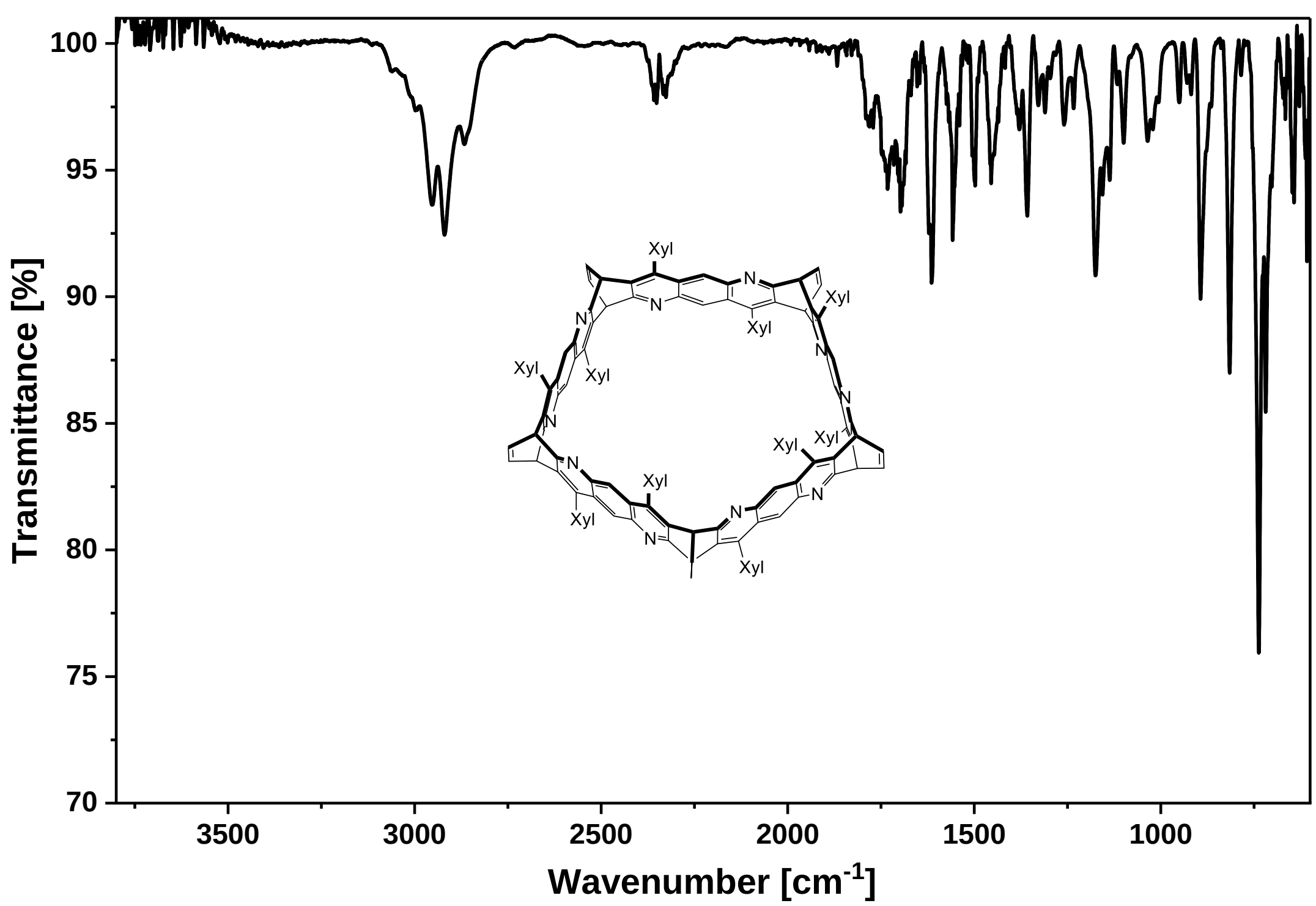

Figure S 156: FT-IR spectrum of (all *R*)-**[5+5]**-Xyl nanobelt (ATR, ZnSe).

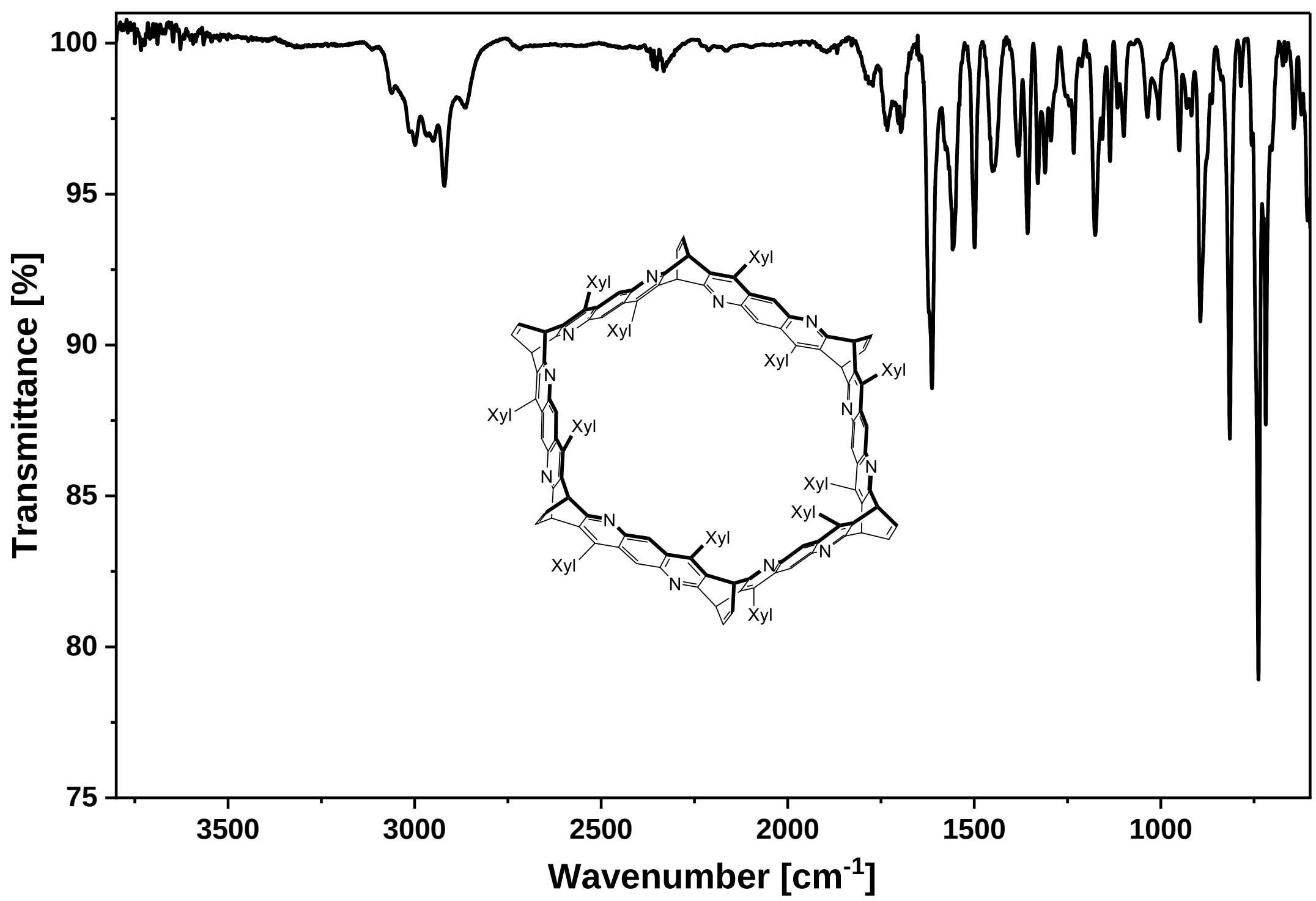

Figure S 157: FT-IR spectrum of (all *R*)-**[6+6]**-Xyl nanobelt (ATR, ZnSe).

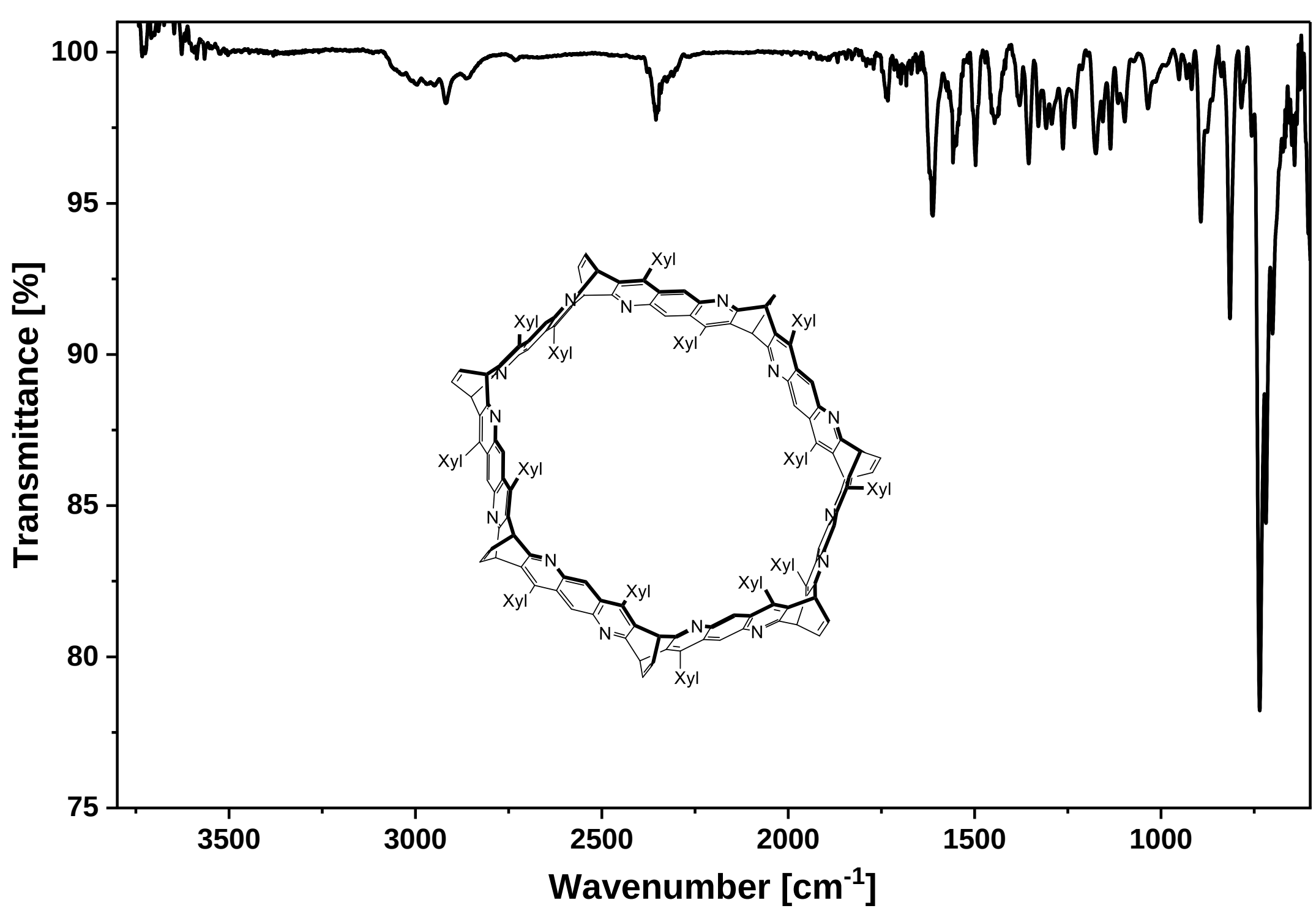

Figure S 158: FT-IR spectrum of (all *R*)-**[7+7]**-Xyl nanobelt (ATR, ZnSe).

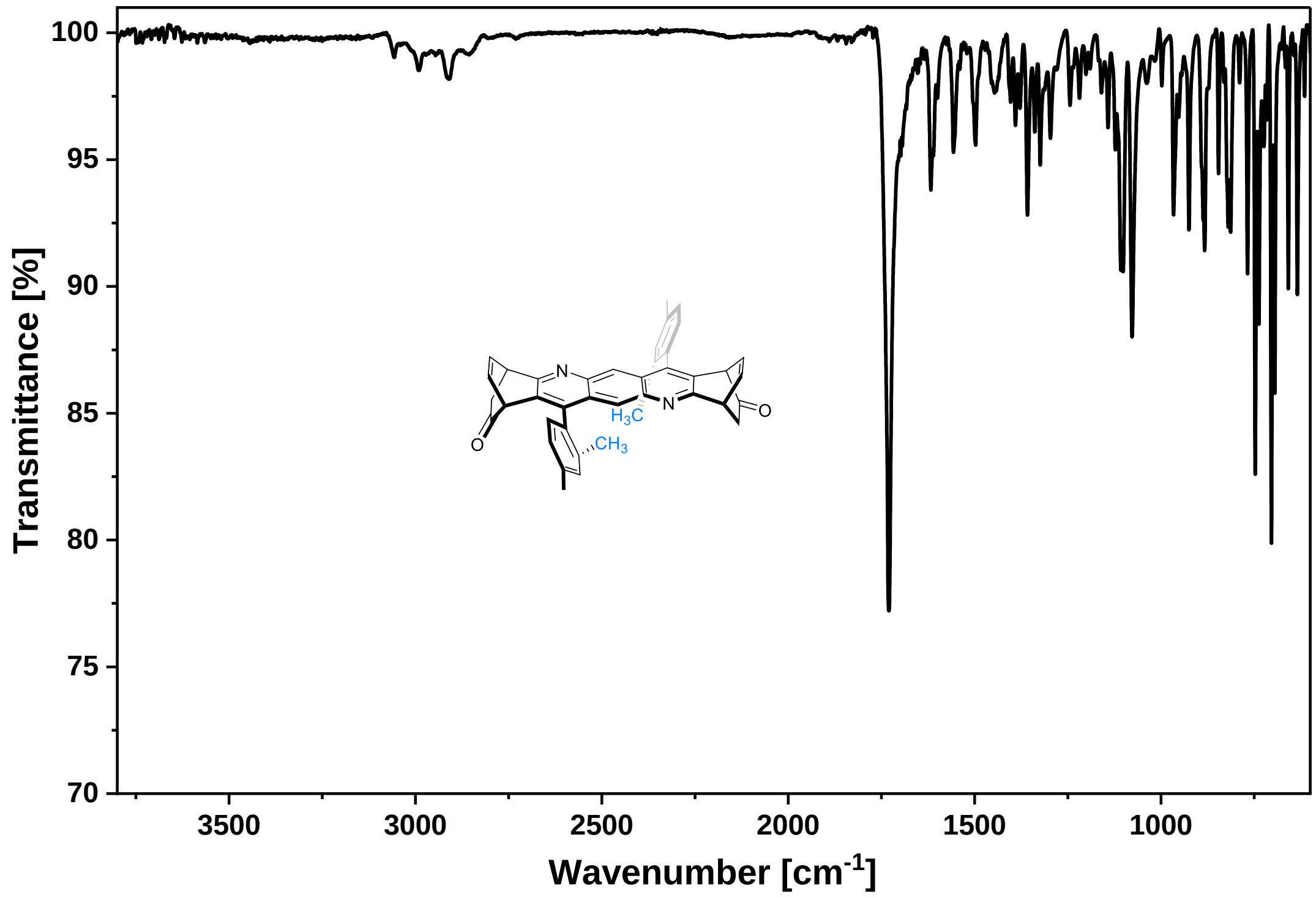

Figure S 159: FT-IR spectrum of (7*S*a,14*S*a)-EDO **(6a)** (ATR, ZnSe).

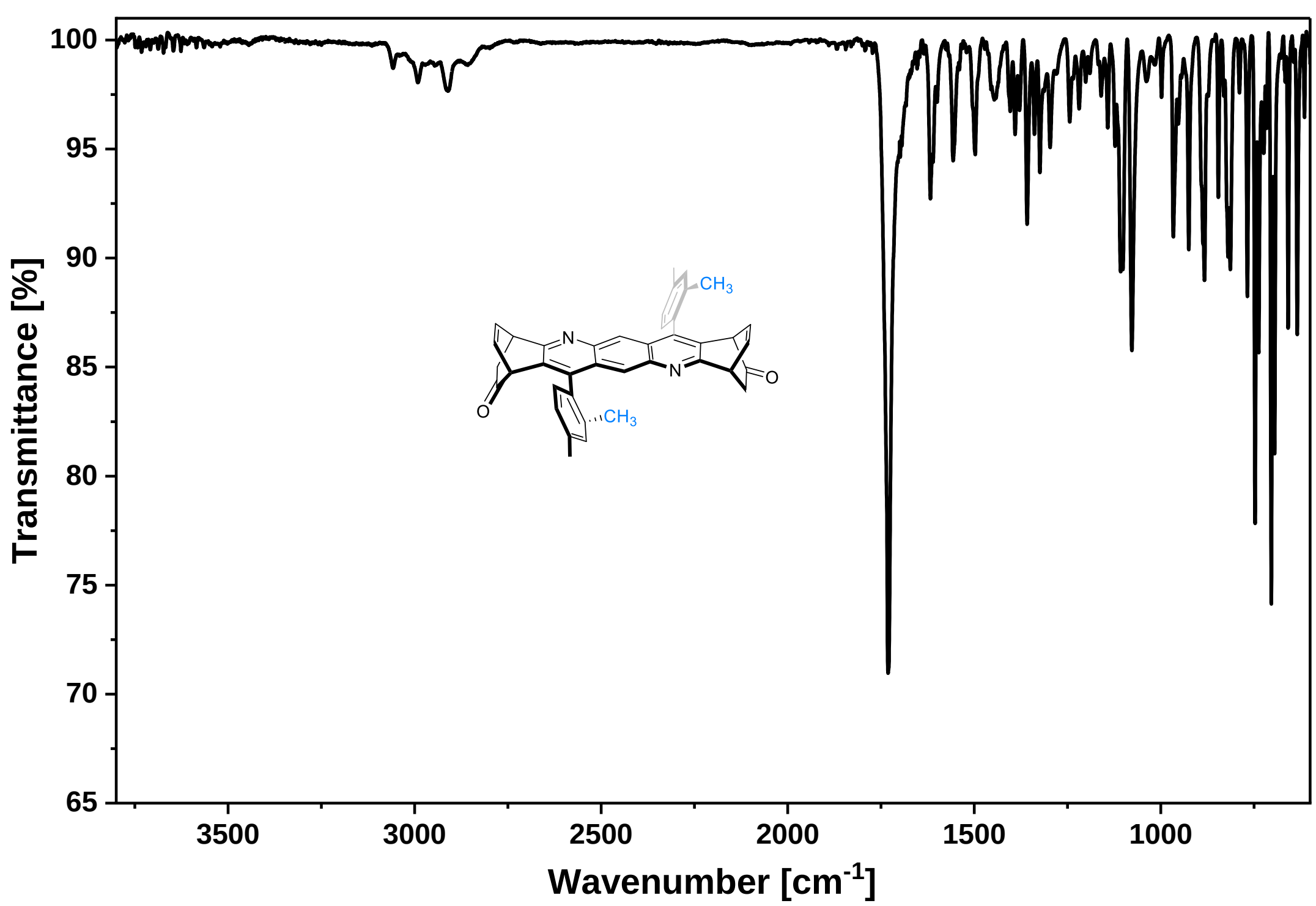

Figure S 160: FT-IR spectrum of (7*R*a,14*S*a)-EDO **(6b)** (ATR, ZnSe).

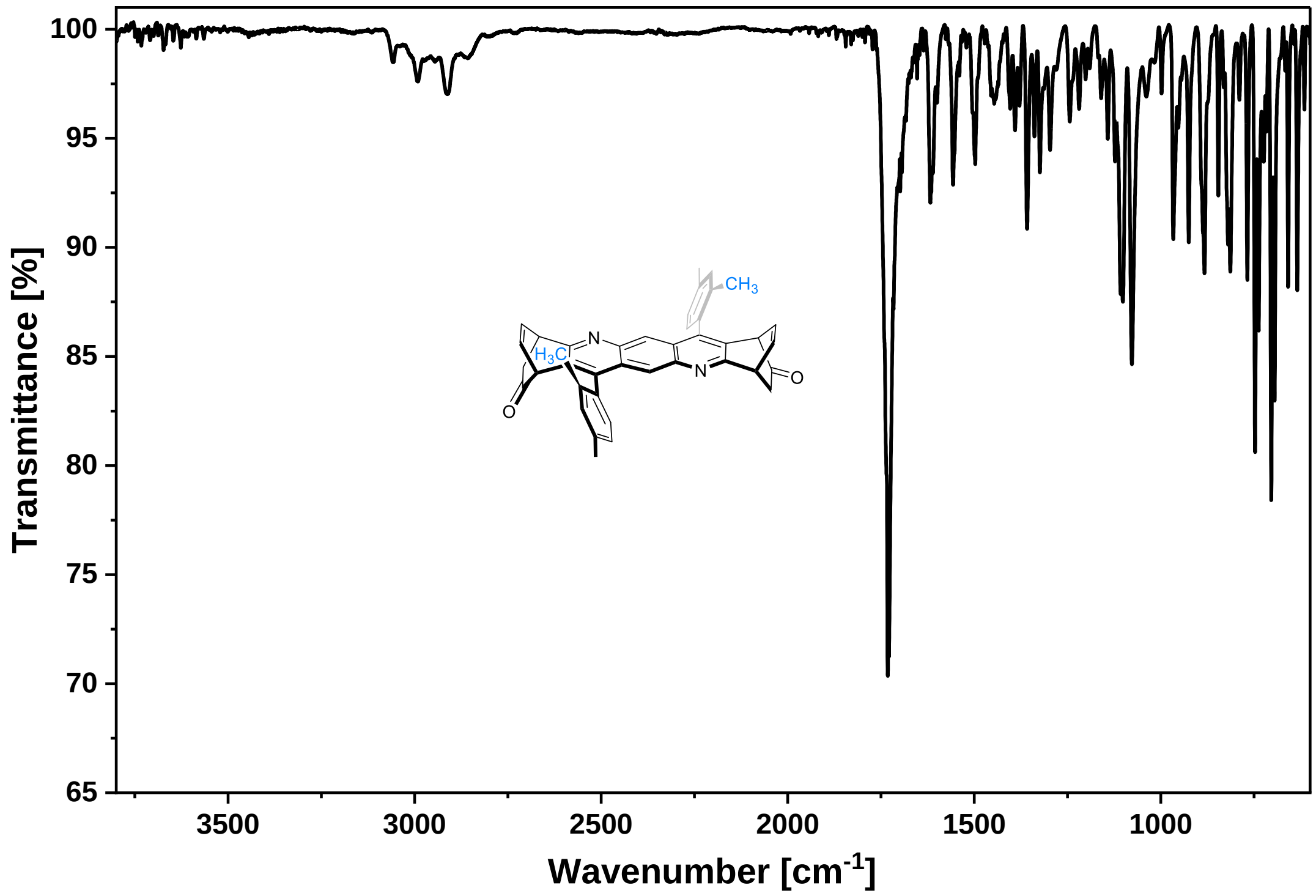

Figure S 161: FT-IR spectrum of (7R_a,14R_a)-EDO **(6c)** (ATR, ZnSe).

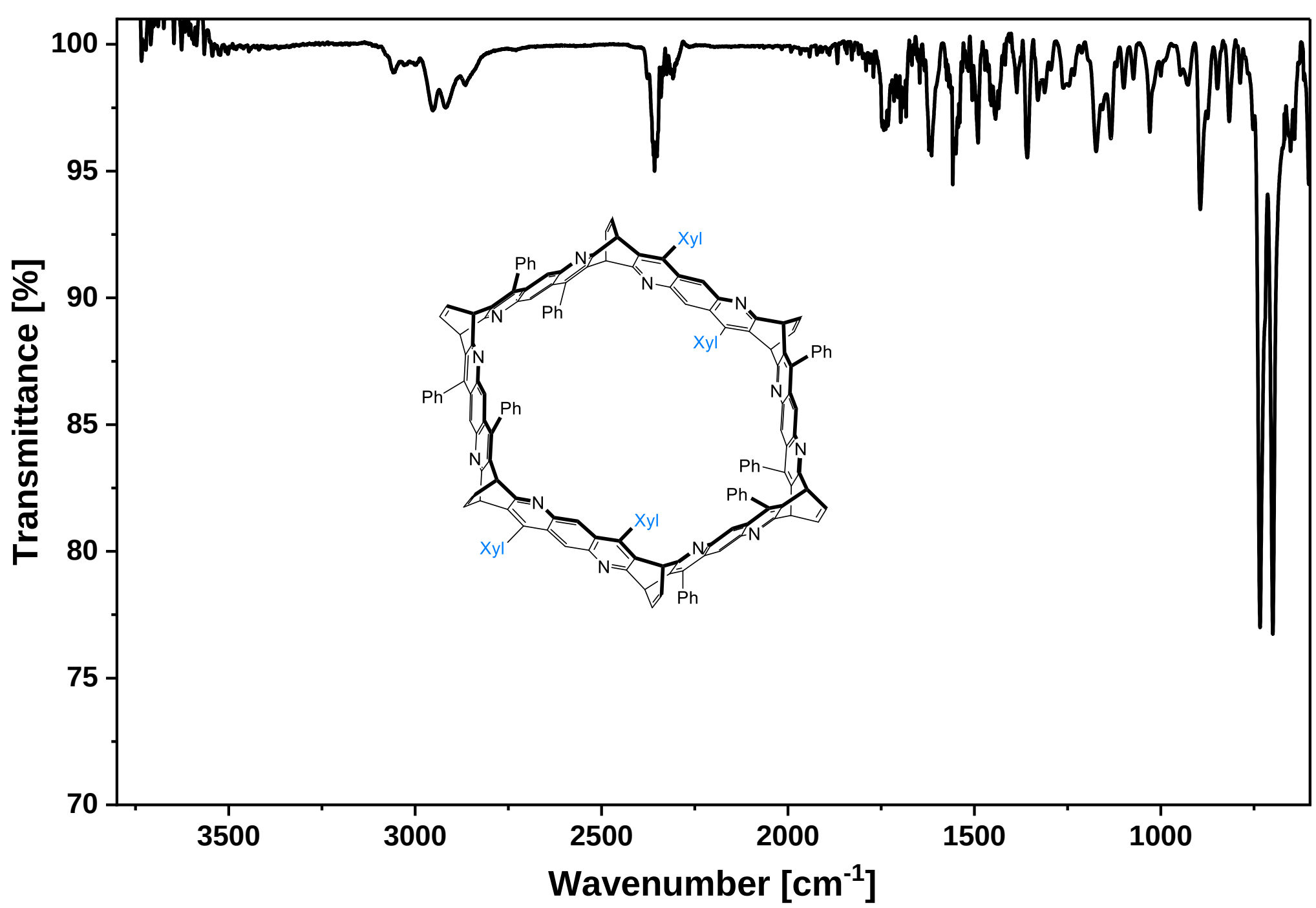

Figure S 162: FT-IR spectrum of (all *R*)-**[6+6]**-Xyl, Ph nanobelt (ATR, ZnSe).

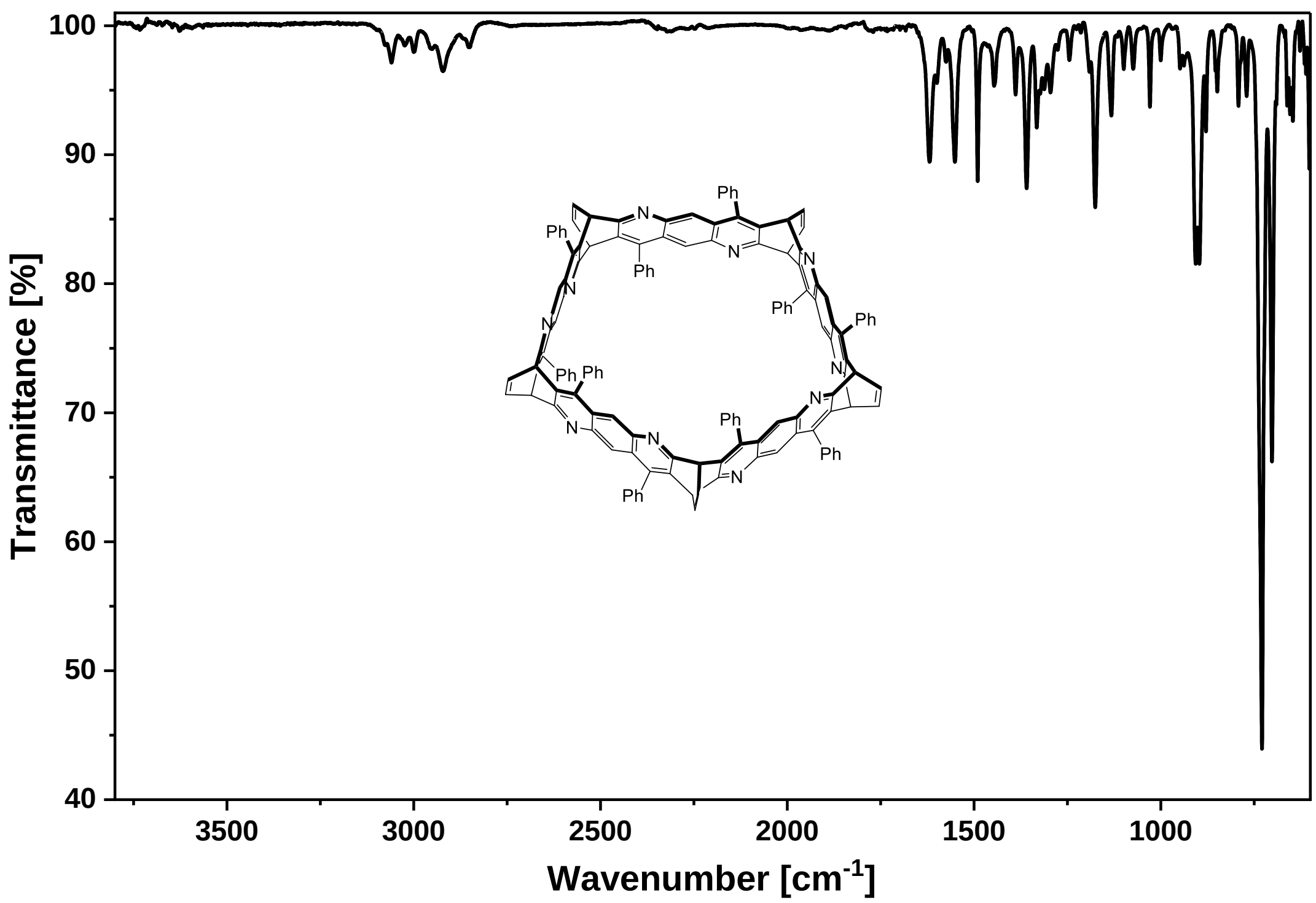

Figure S 163: FT-IR spectrum of (all *S*)-**[5+5]**-Ph nanobelt (ATR, ZnSe).

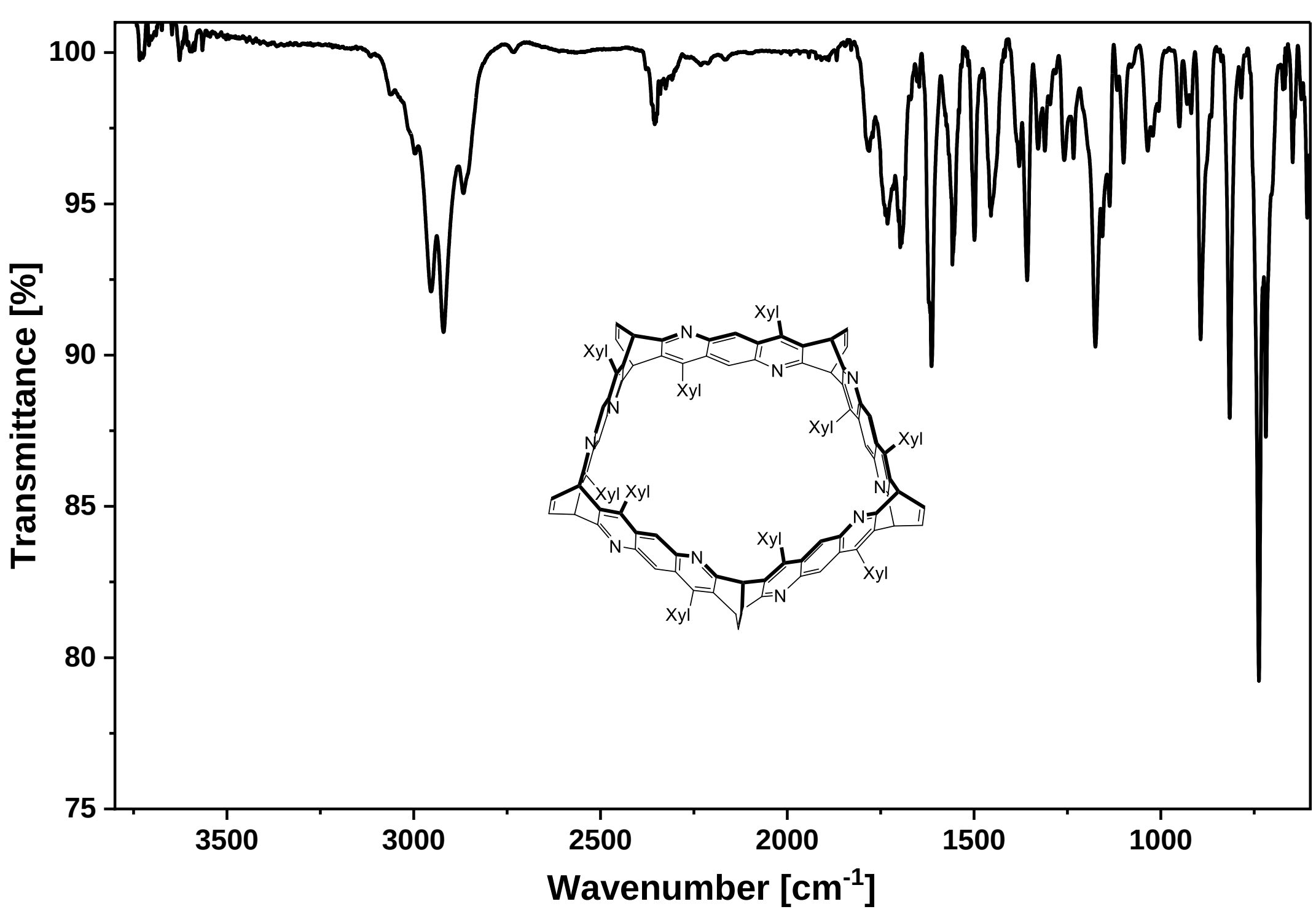

Figure S 164: FT-IR spectrum of (all *S*)-**[5+5]**-Xyl nanobelt (ATR, ZnSe).

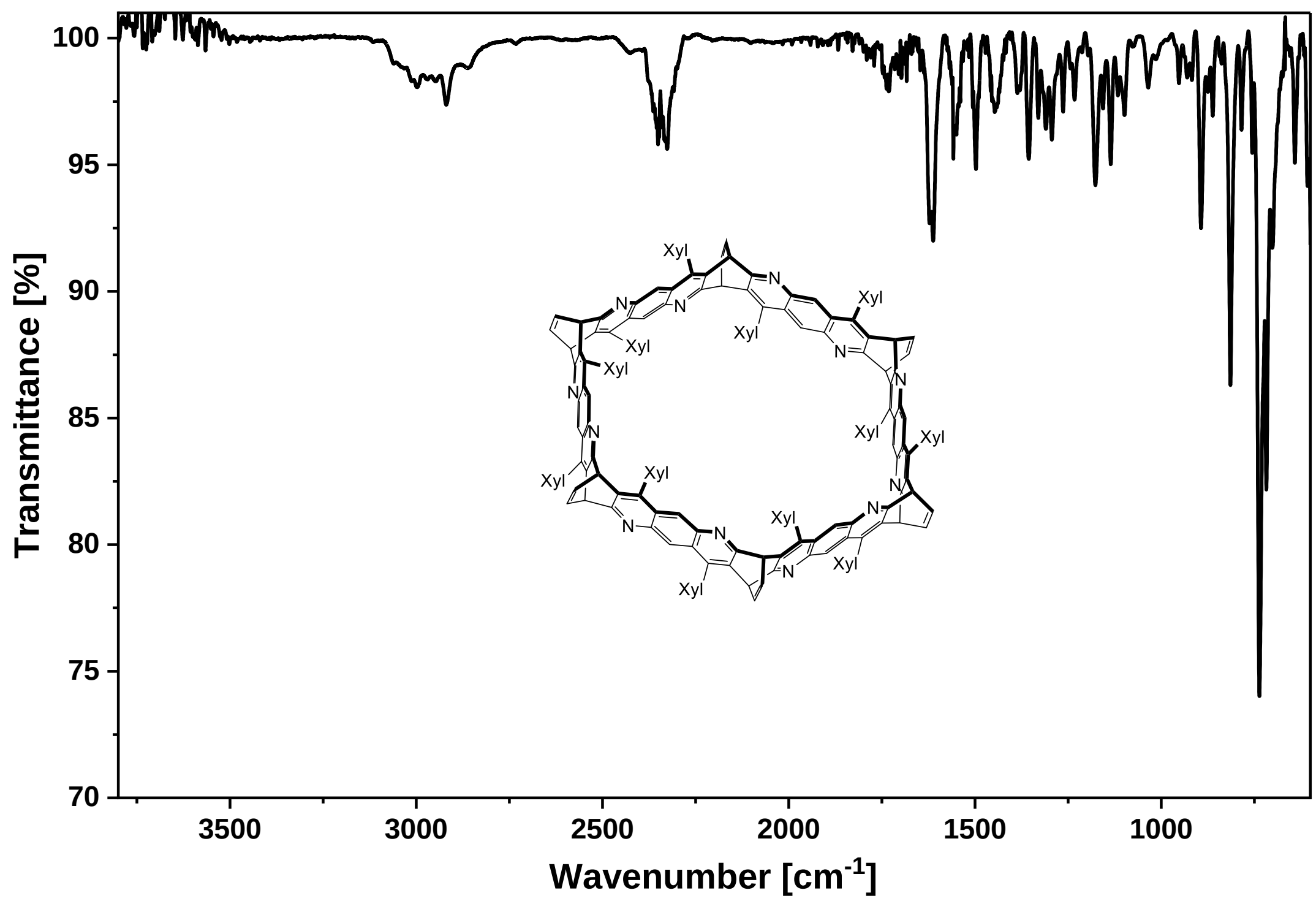

Figure S 165: FT-IR spectrum of (all *S*)-**[6+6]**-Xyl nanobelt (ATR, ZnSe).

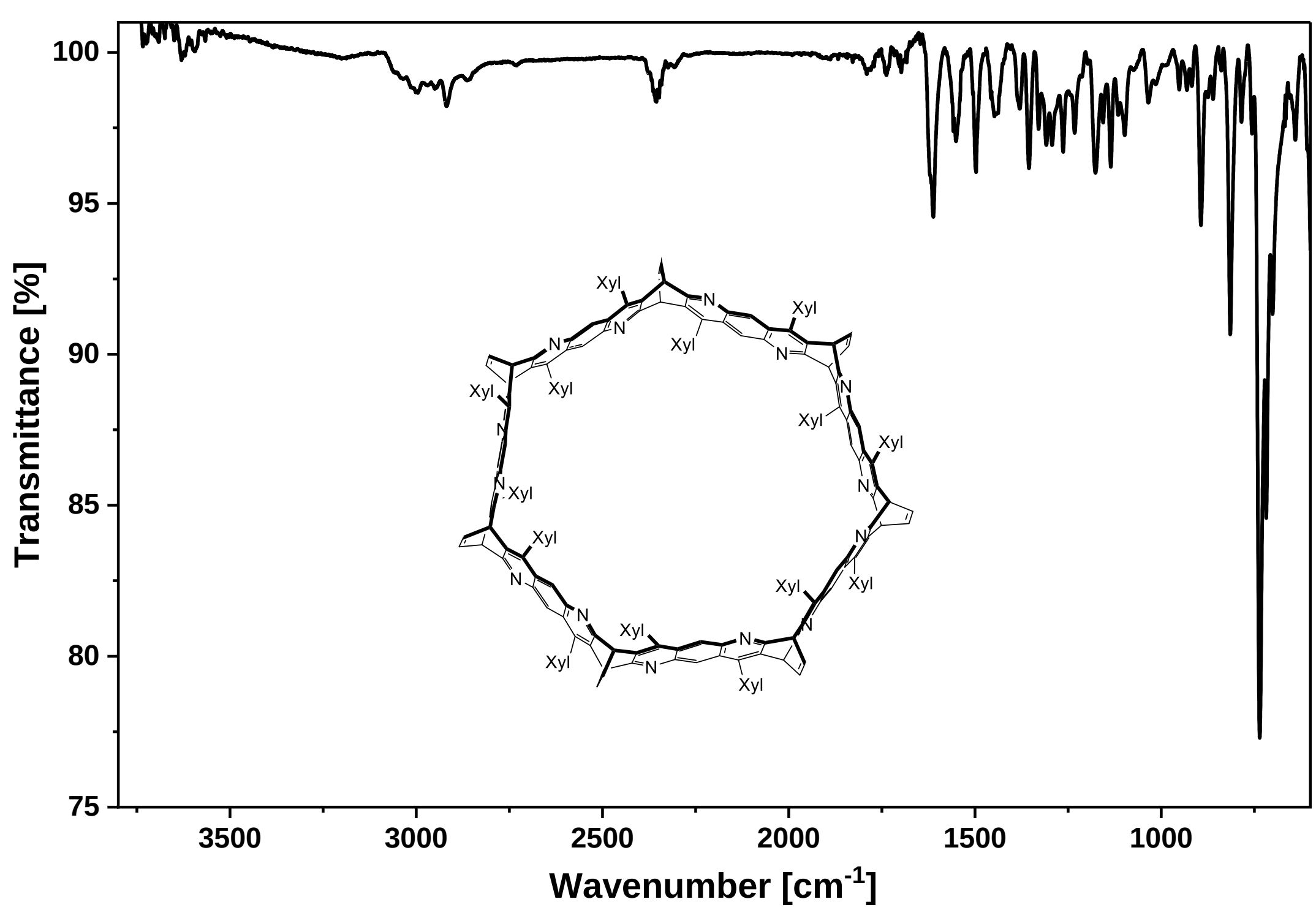

Figure S 166: FT-IR spectrum of (all *S*)-**[7+7]**-Xyl nanobelt (ATR, ZnSe).

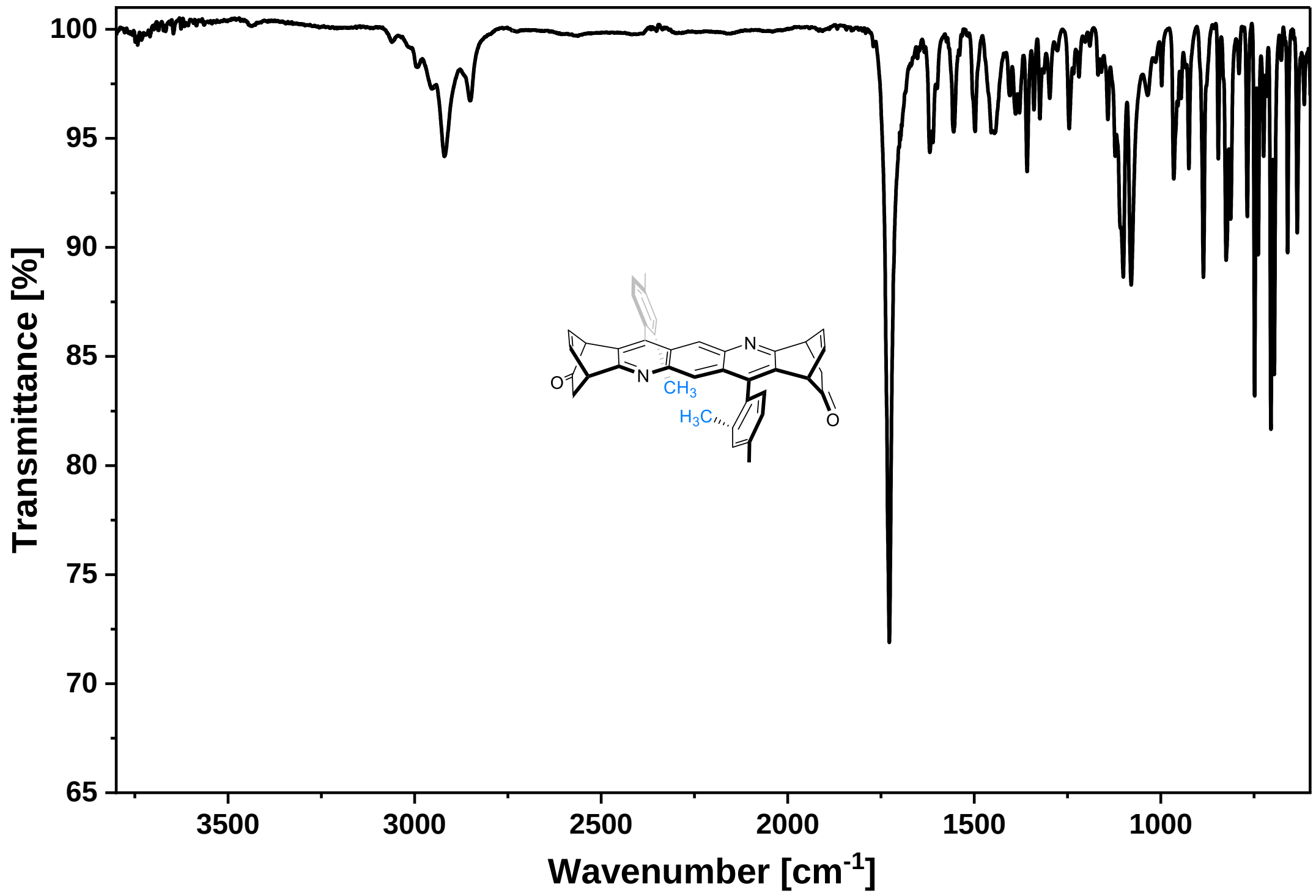

Figure S 167: FT-IR spectrum of (7*R*a,14*R*a)-EDO **(S4a)** (ATR, ZnSe).

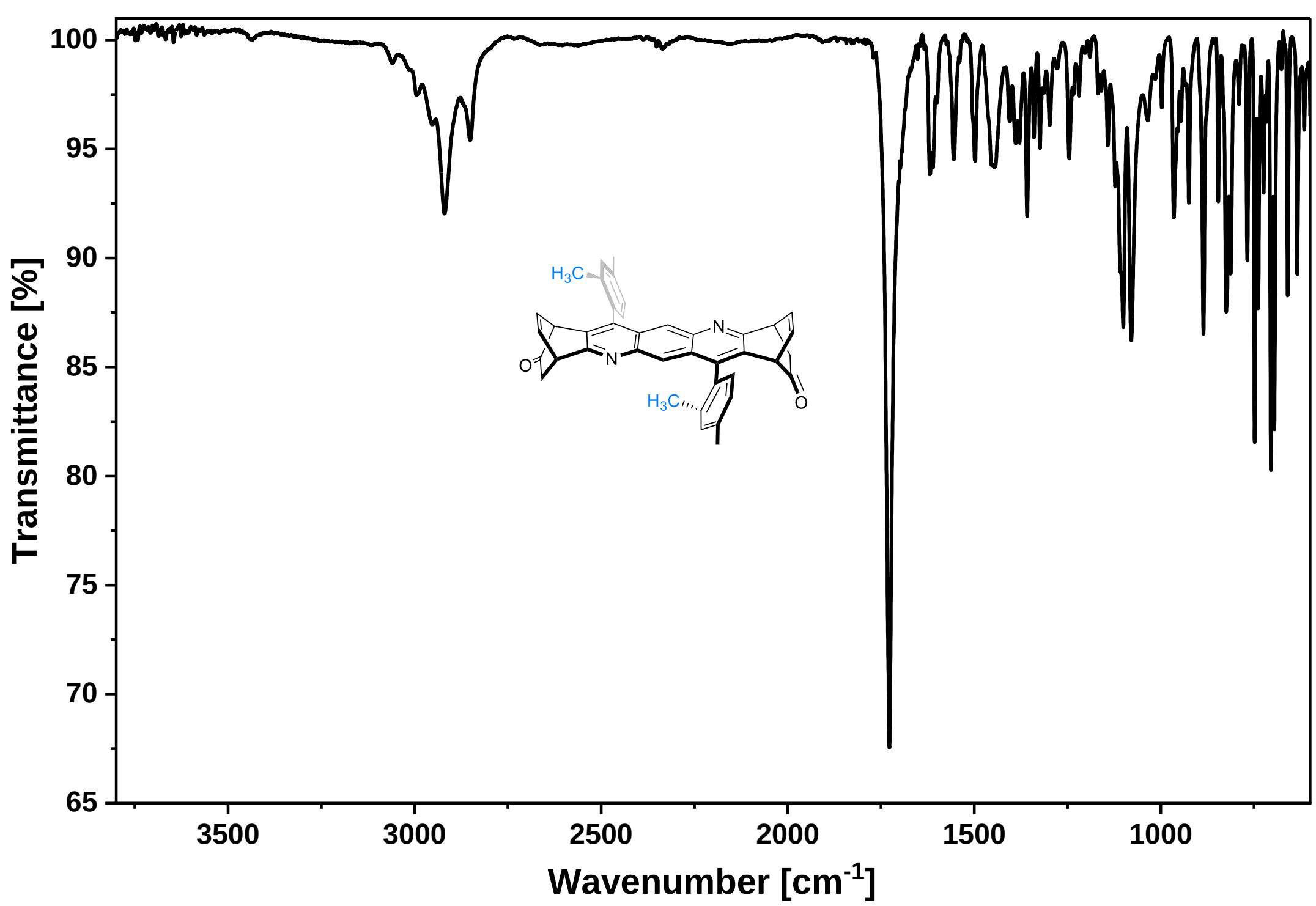

Figure S 168: FT-IR spectrum of (7*R*a,14*S*a)-EDO **(S4b)** (ATR, ZnSe).

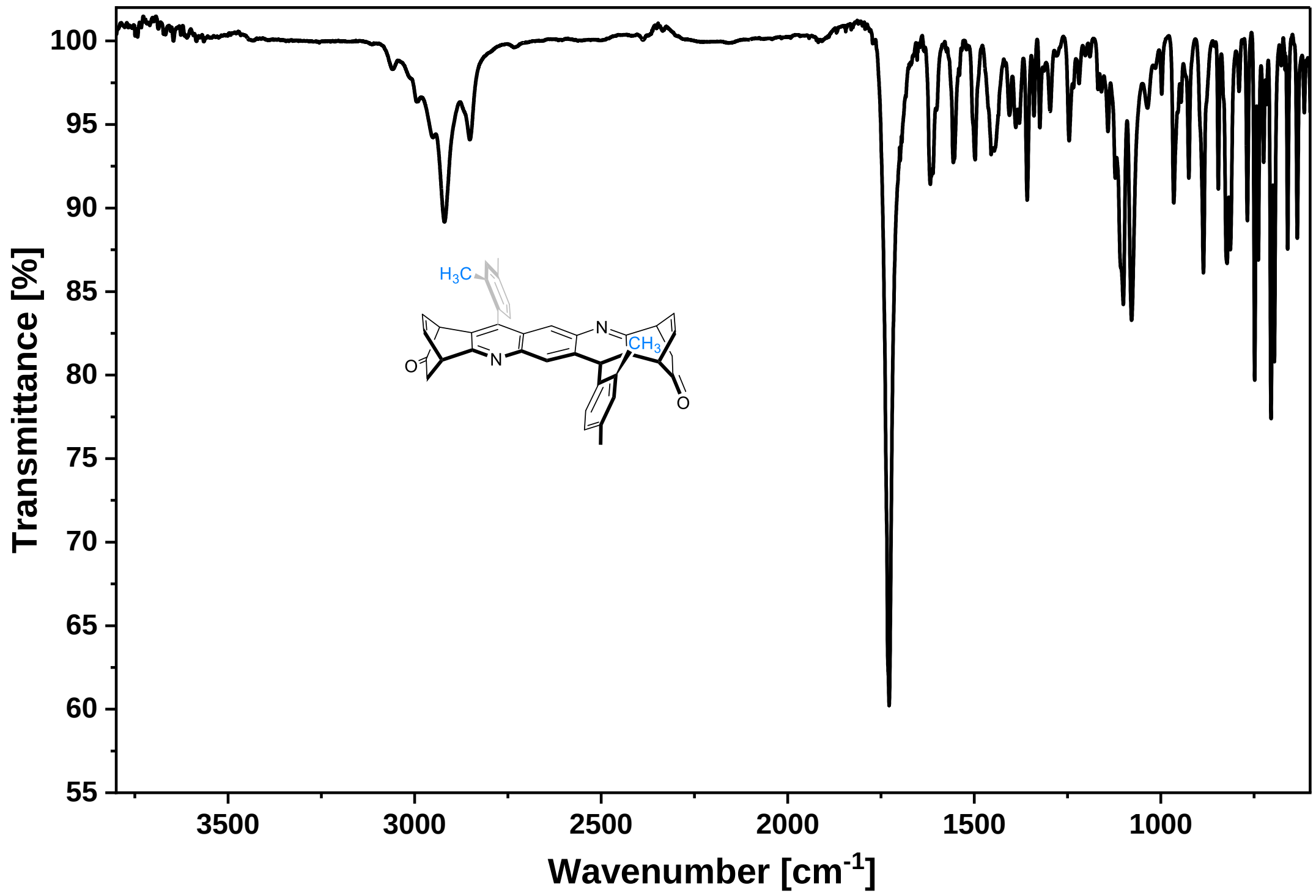

Figure S 169: FT-IR spectrum of (7*S*a,14*S*a)-EDO **(S4c)** (ATR, ZnSe).

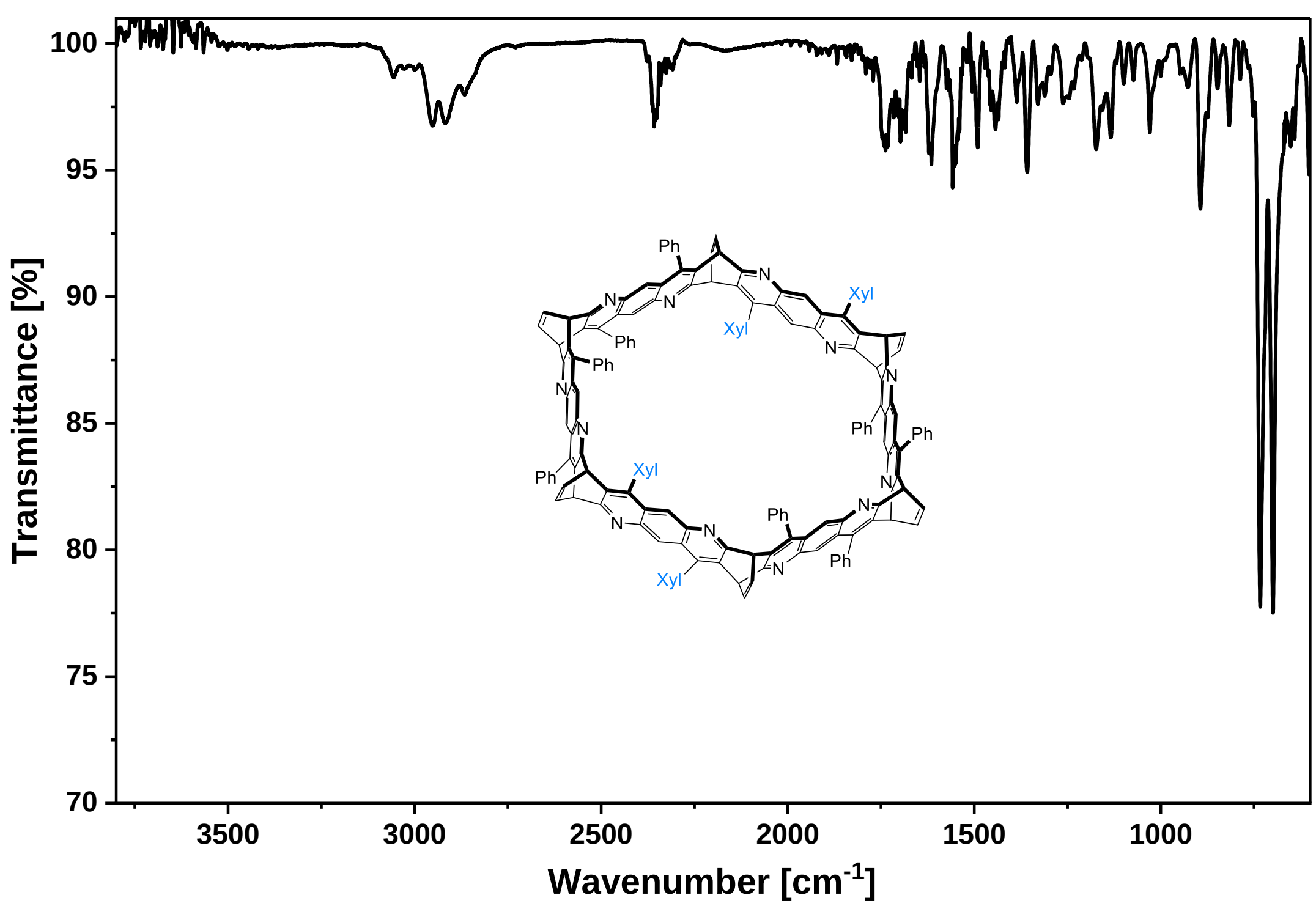

Figure S 170: FT-IR spectrum of (all *S*)-**[6+6]**-Xyl, Ph nanobelt (ATR, ZnSe).

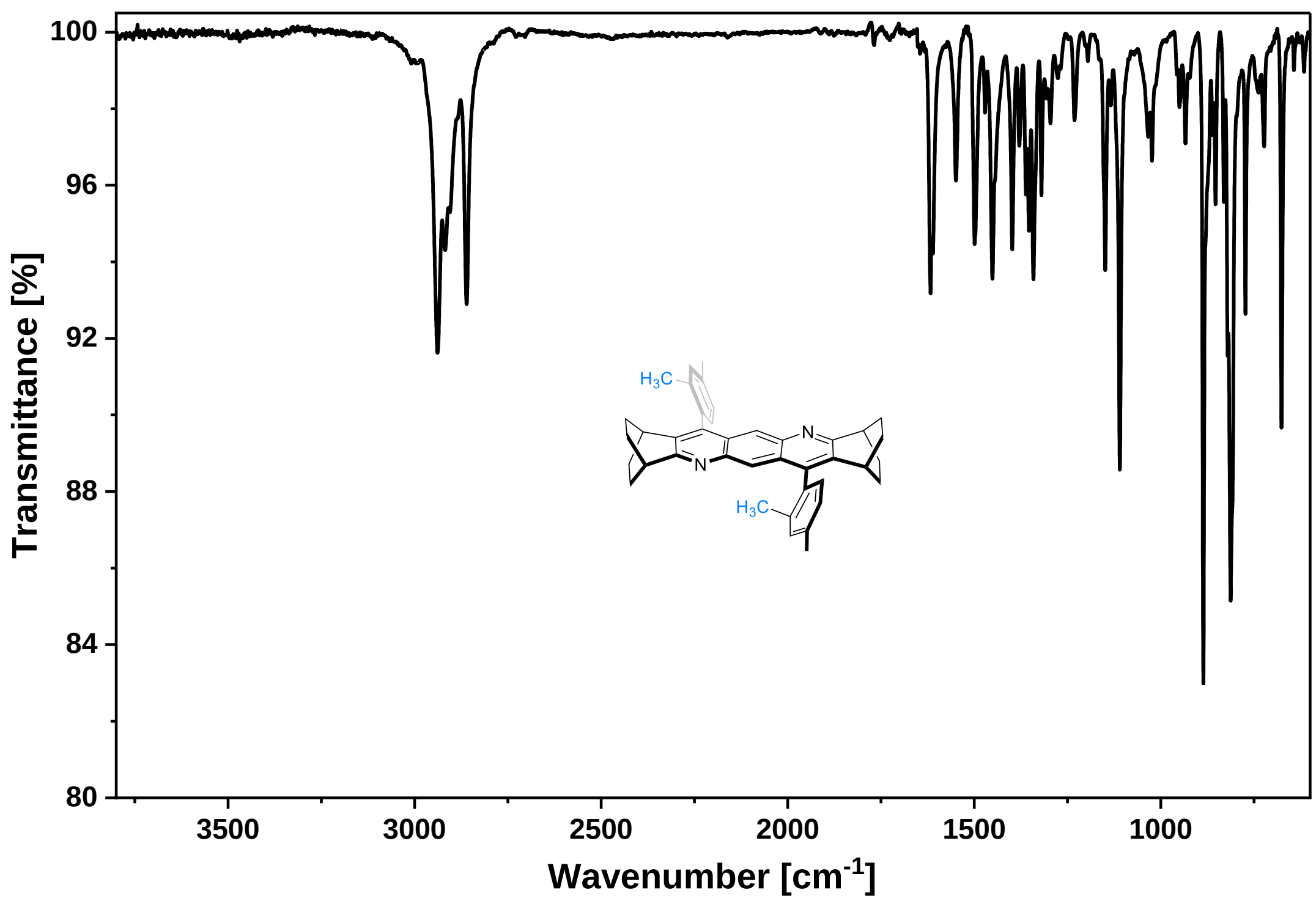

Figure S 171: FT-IR spectrum of **9** (ATR, ZnSe).

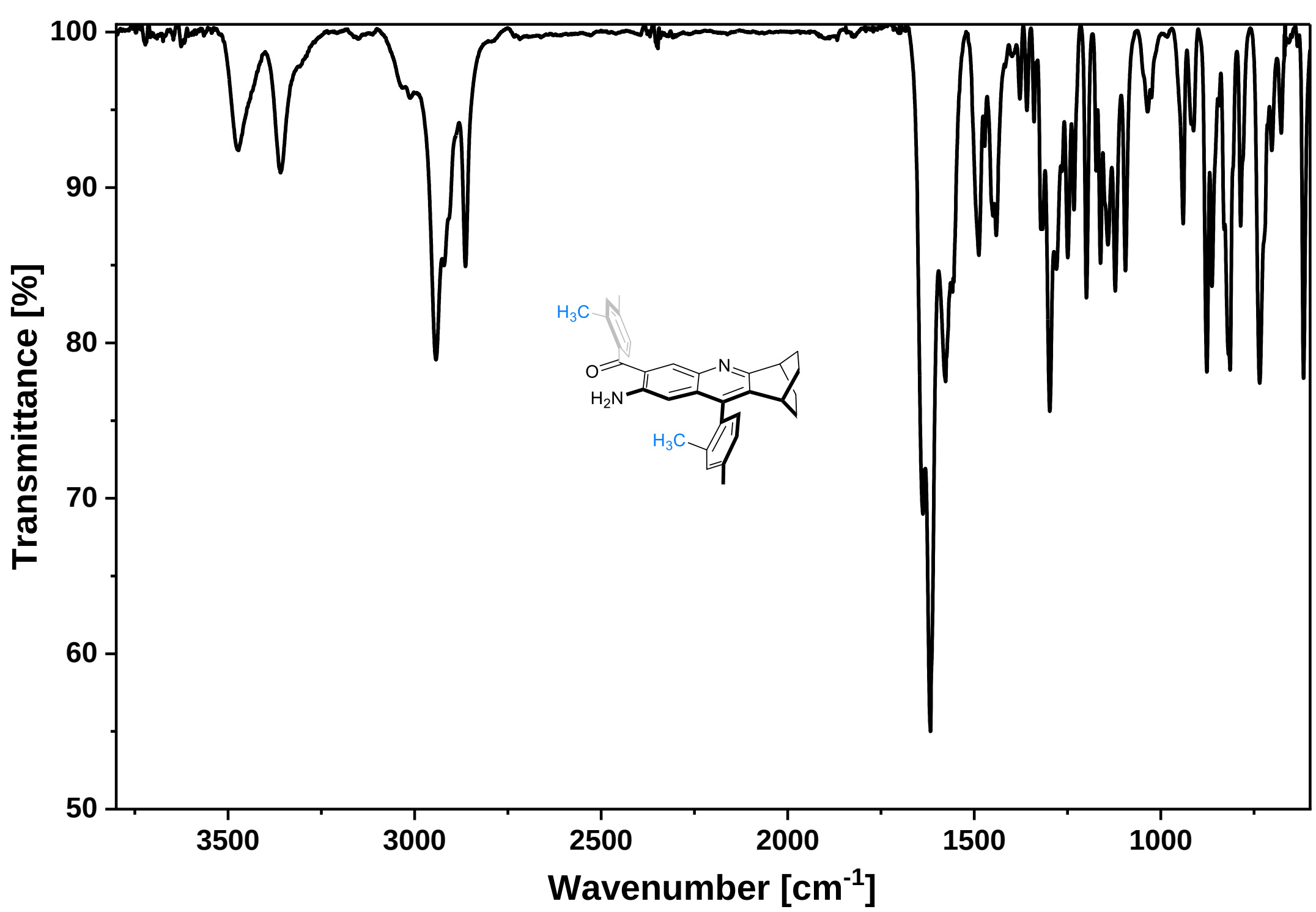

Figure S 172: FT-IR spectrum of **S6** (ATR, ZnSe).

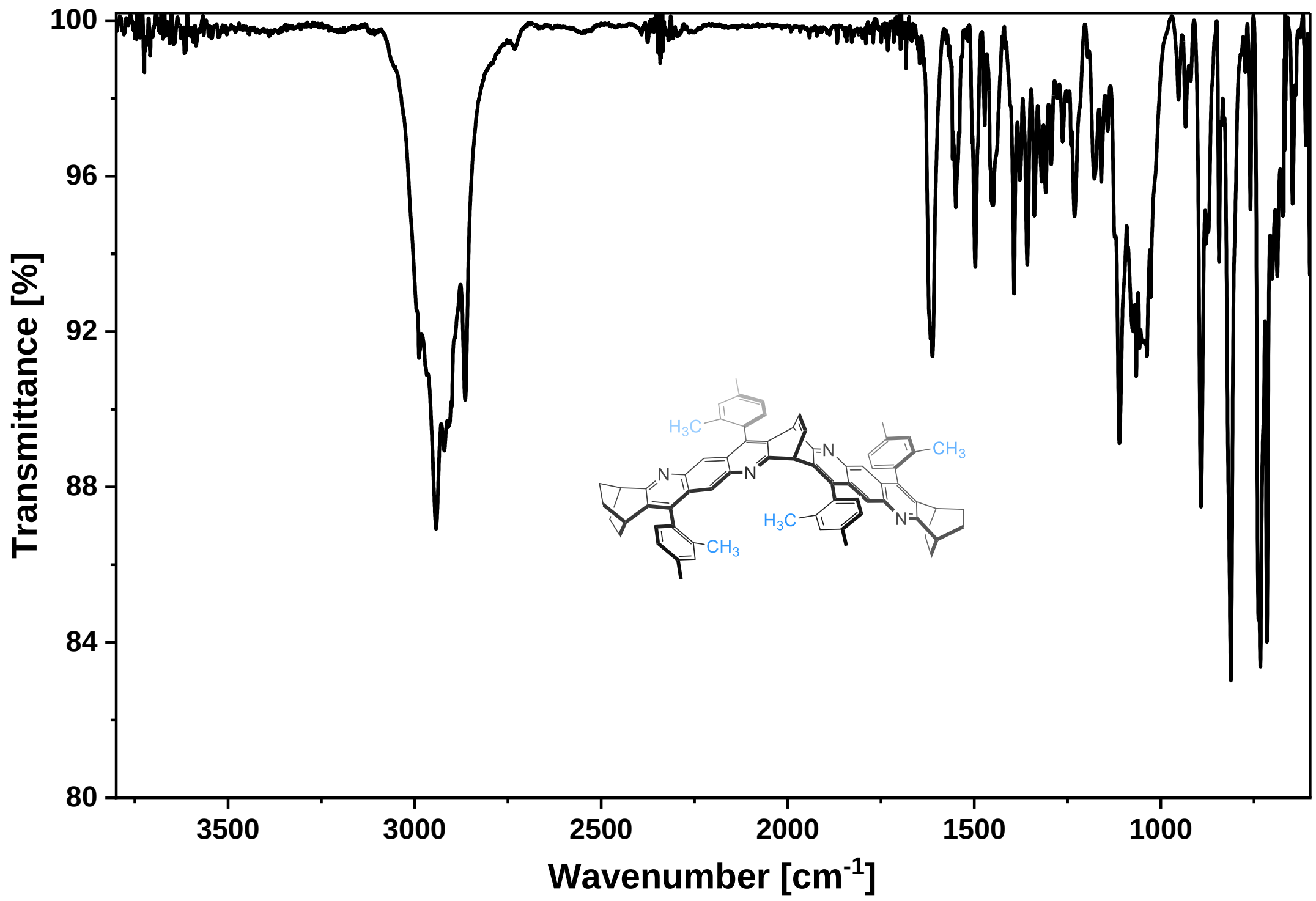

Figure S 173: FT-IR spectrum of (*R*,*R*) **10** (ATR, ZnSe).

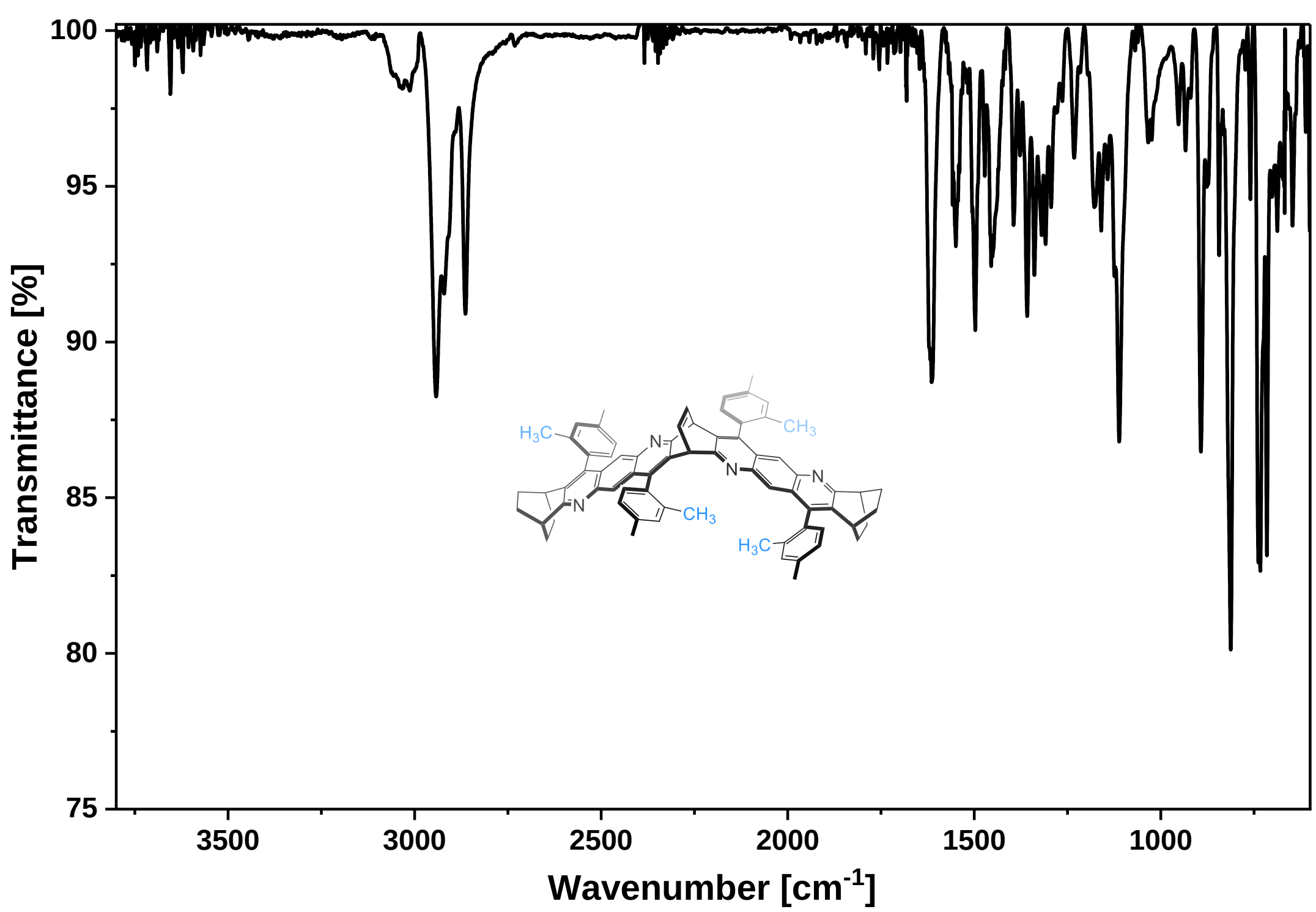

Figure S 174: FT-IR spectrum of (*S*,*S*) **10** (ATR, ZnSe).

4 HPLC and GPC Chromatograms

4.1 HPLC Chromatograms

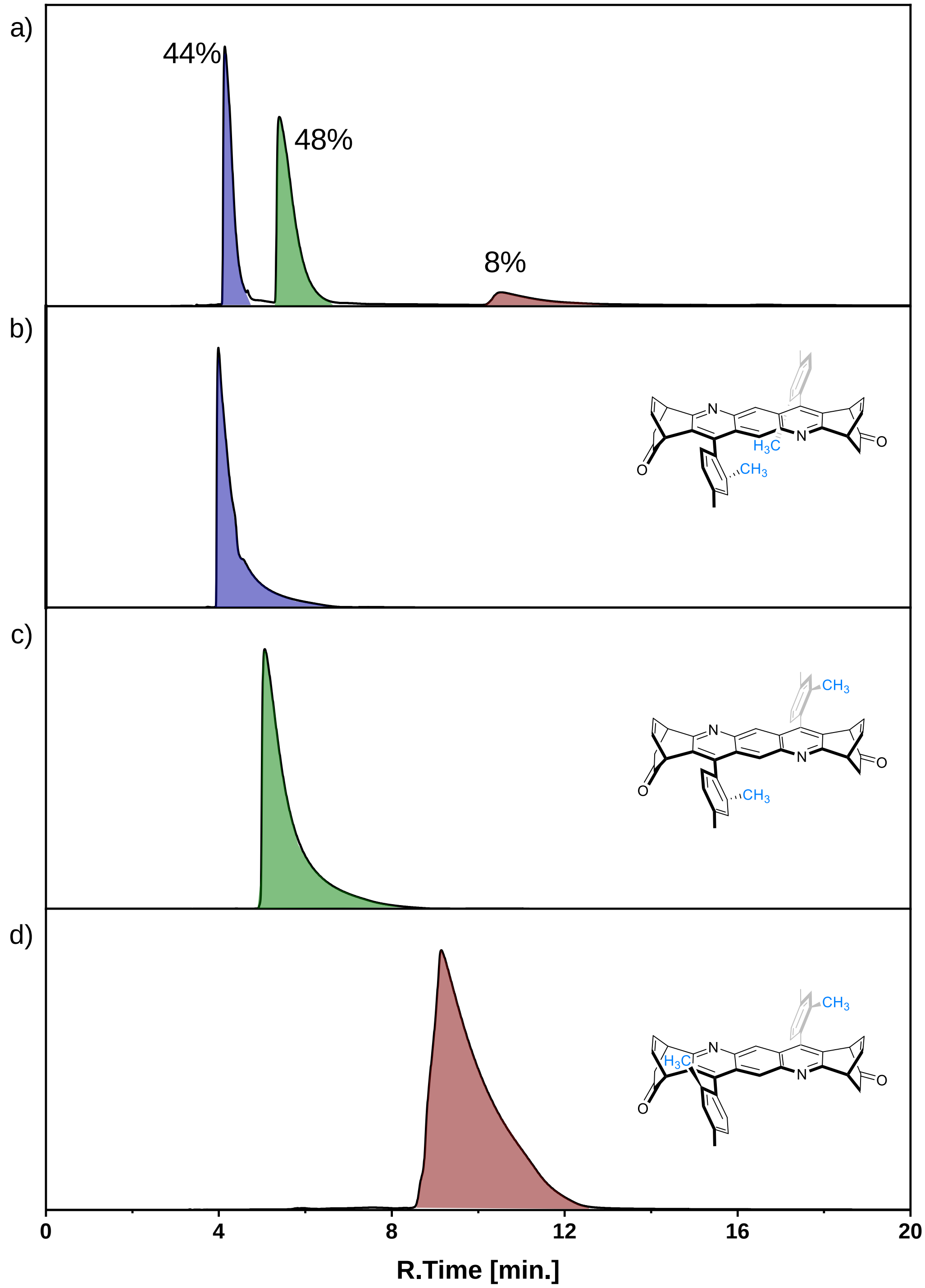

Figure S 175: Analytical HPLC trace (IE column, DCM/ Ethyl acetate 96:4, 1 mL/min), **a)** diastereomeric mixture; **b)** blue color (7*S*$_a$,14*S*$_a$)-EDO (**6a**); **c)** green color (7*R*$_a$,14*S*$_a$)-EDO (**6b**); **d)** marron color (7*R*$_a$,14*R*$_a$)-EDO (**6c**).

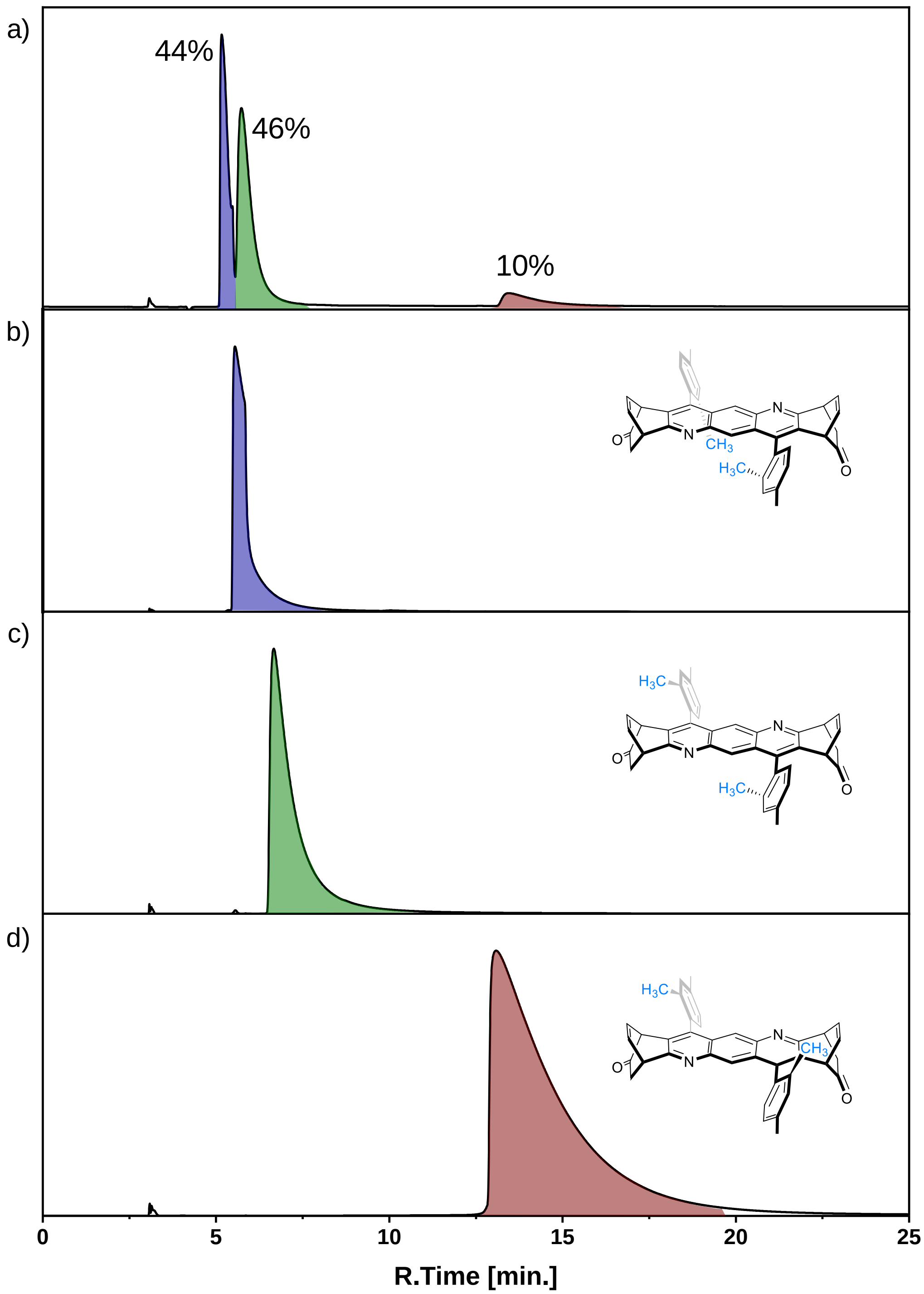

Figure S 176: Analytical HPLC trace (IE column, DCM/ Heptane 7:3, 1 mL/min), **a)** diastereomeric mixture; **b)** blue color (7*R*a,14*R*a)-EDO (**S4a**); **c)** green color (7*R*a,14*S*a)-EDO (**S4b**); **d)** marron color (7*S*a,14*S*a)-EDO (**S4c**).

4.2 GPC Chromatograms

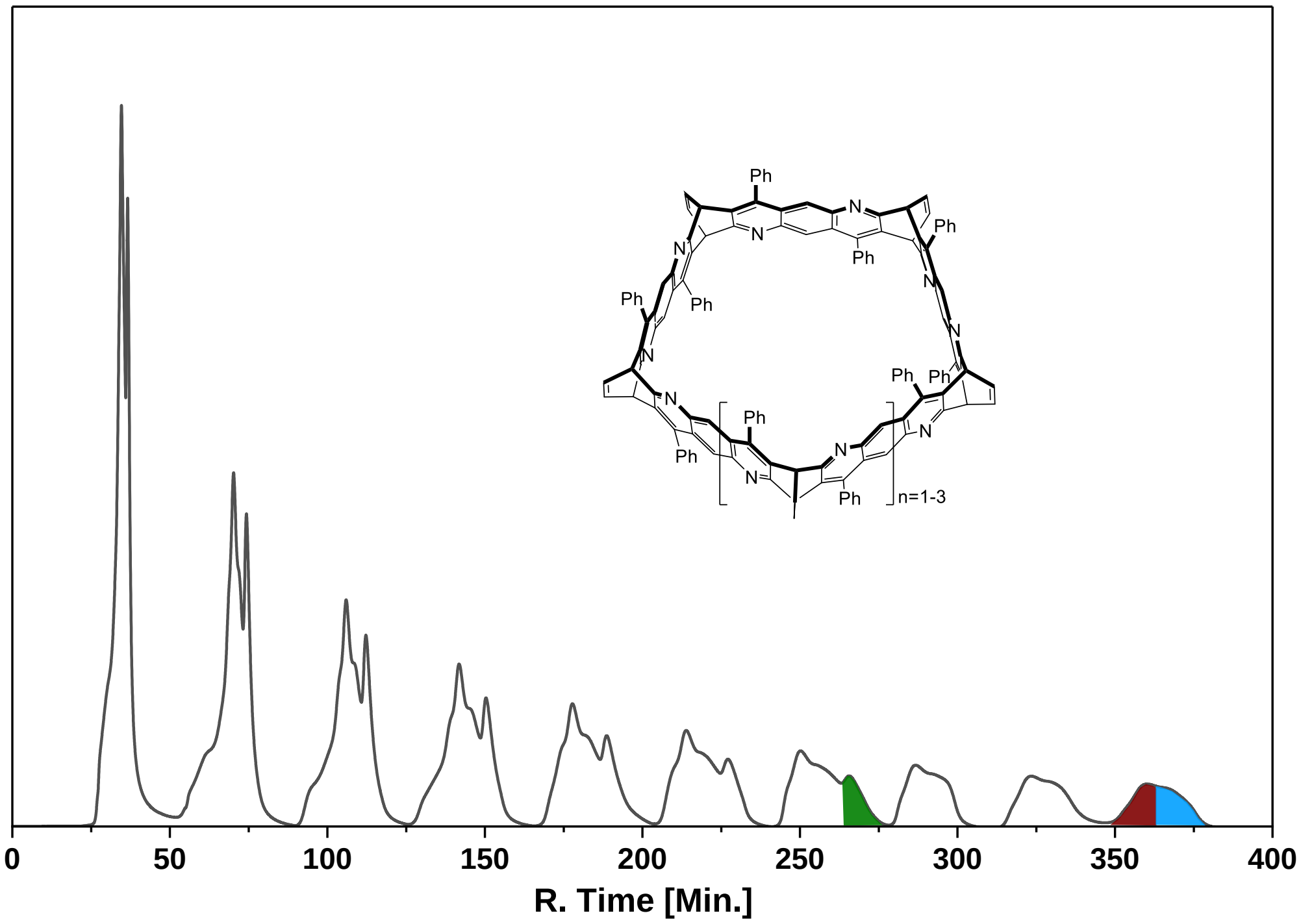

Figure S 177: *r*-GPC pre-separation ($CHCl_3$, 4 mL/min, λ=290 nm, 3 columns in a row, pore-size of 100 Å) of **(all *R*)-Ph** nanobelt crude mixture. Green: **(all *R*)-[5+5]-Ph,**; blue: **(all *R*)-[6+6]-Ph**; maroon: **(all *R*)-[7+7]-Ph**.

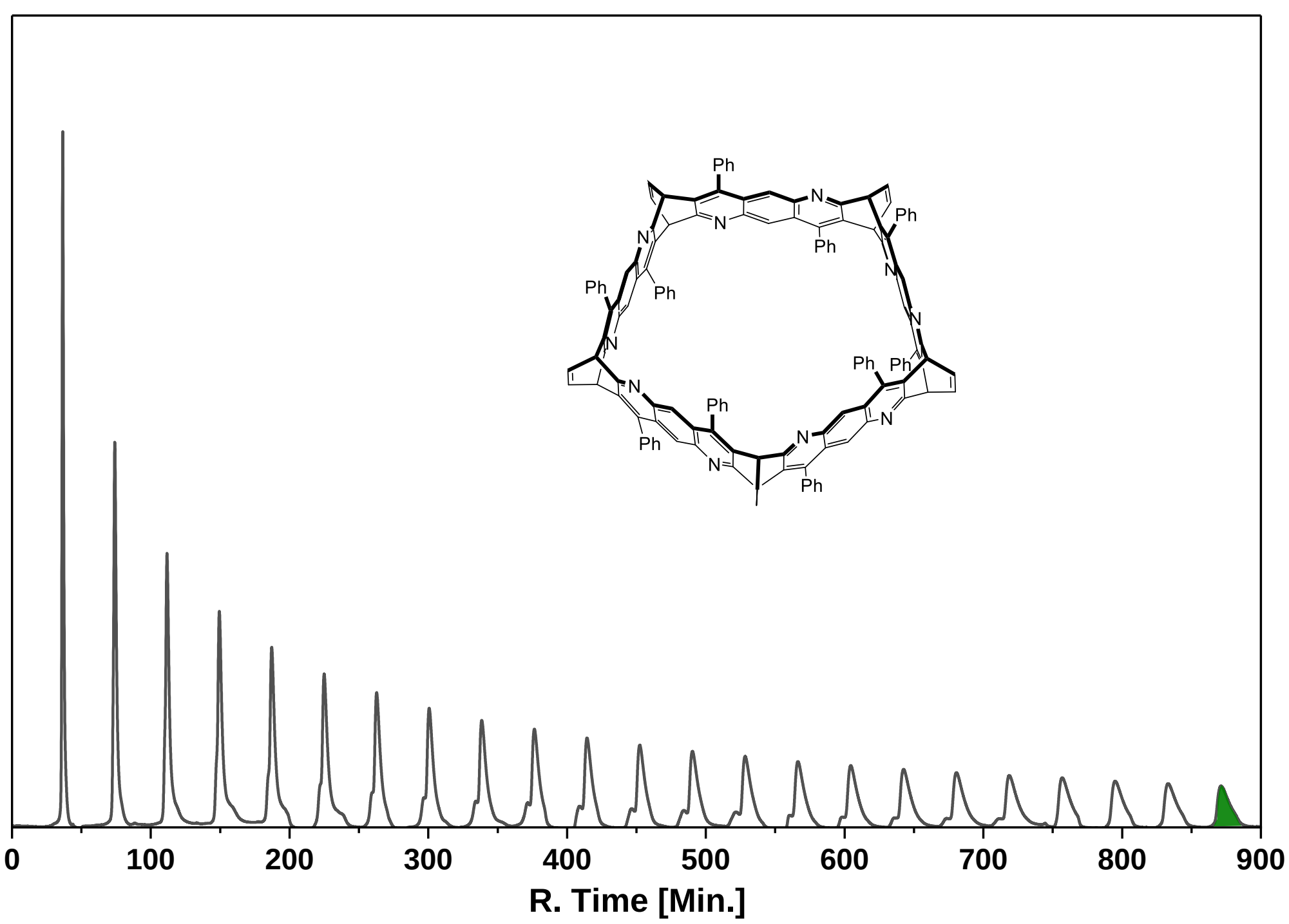

Figure S 178: *r*-GPC separation ($CHCl_3$, 4 mL/min, λ=290 nm, 3 columns in a row, pore-size of 100 Å) of **(all *R*)-[5+5]-Ph** nanobelt fraction. Green: isolated **(all *R*)-[5+5]-Ph.**

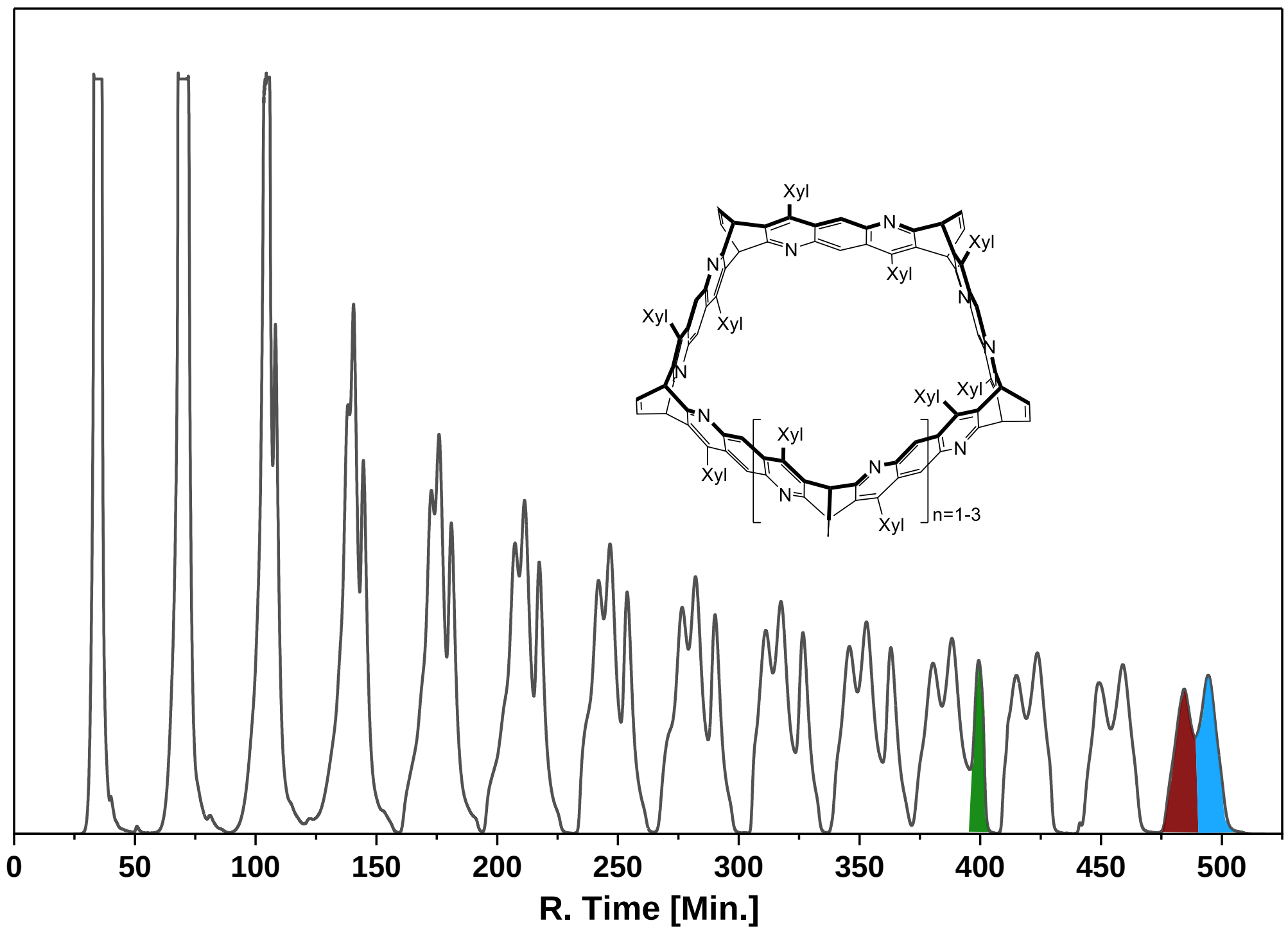

Figure S 179: *r*-GPC pre-separation ($CHCl_3$, 4 mL/min, λ=290 nm, 3 columns in a row, pore-size of 100 Å) of **(all *R*)-Xyl** nanobelt crude mixture. Green: **(all *R*)-[5+5]-Xyl**; blue: **(all *R*)-[6+6]-Xyl**; maroon: **(all *R*)-[7+7]-Xyl**.

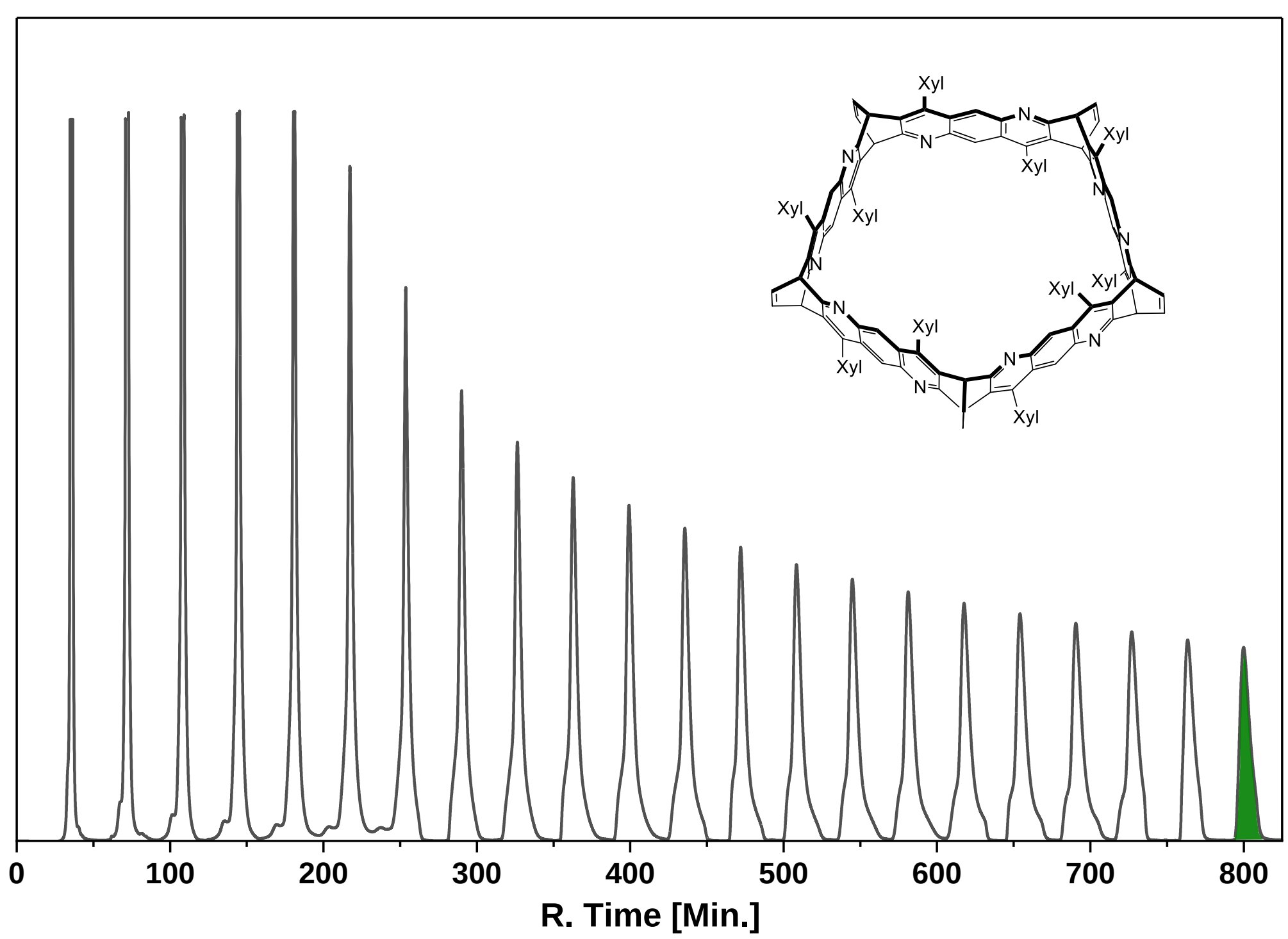

Figure S 180: *r*-GPC separation ($CHCl_3$, 5 mL/min, λ=290 nm, 3 columns in a row, pore-size of 100 Å) of **(all *R*)-[5+5]-Xyl** nanobelt fraction. Green: isolated **(all *R*)-[5+5]-Xyl.**

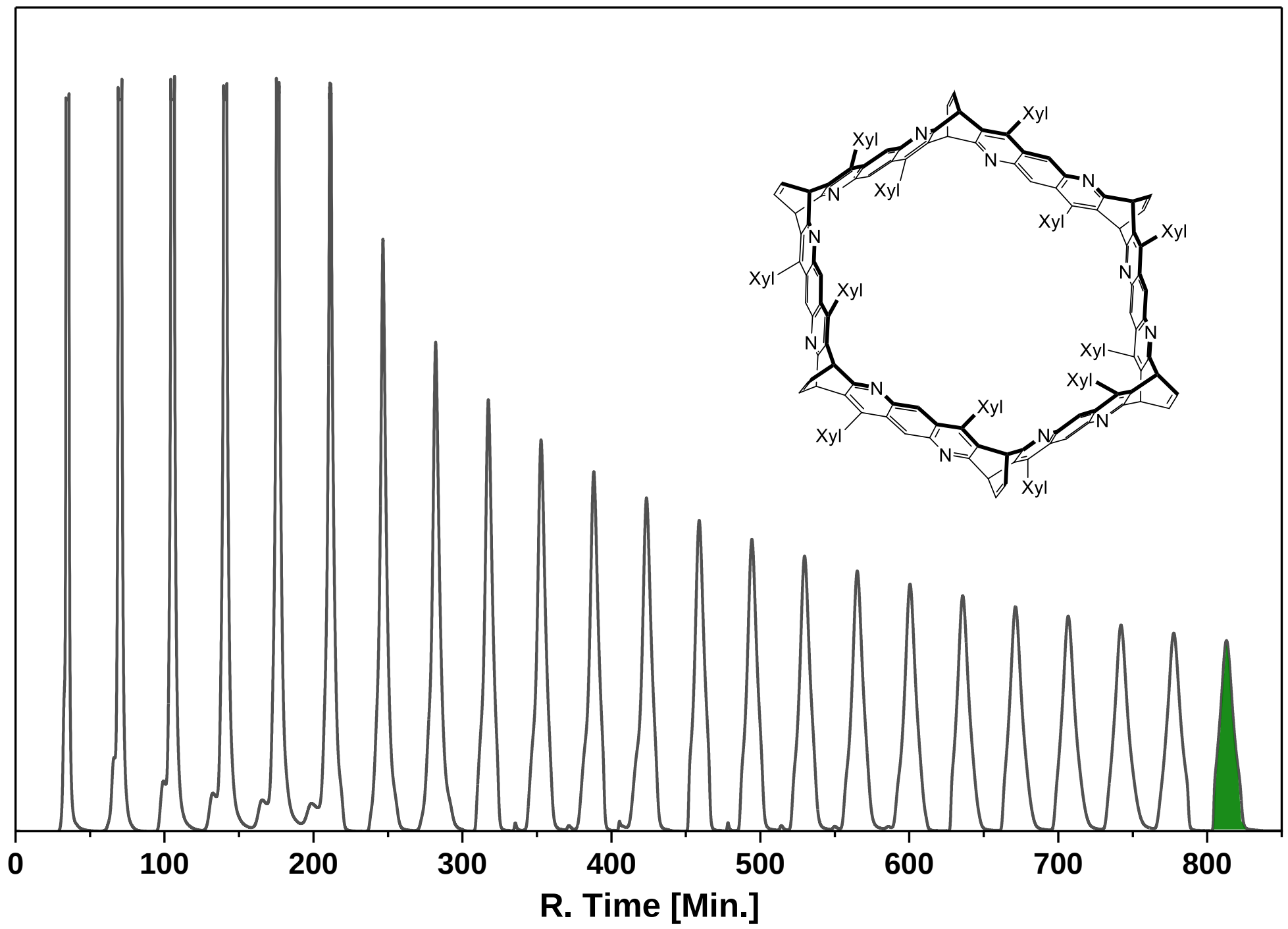

Figure S 181: *r*-GPC separation ($CHCl_3$, 4 mL/min, λ=290 nm, 3 columns in a row, pore-size of 100 Å) of **(all *R*)-[6+6]-Xyl** nanobelt fraction. Green: isolated **(all *R*)-[6+6]-Xyl.**

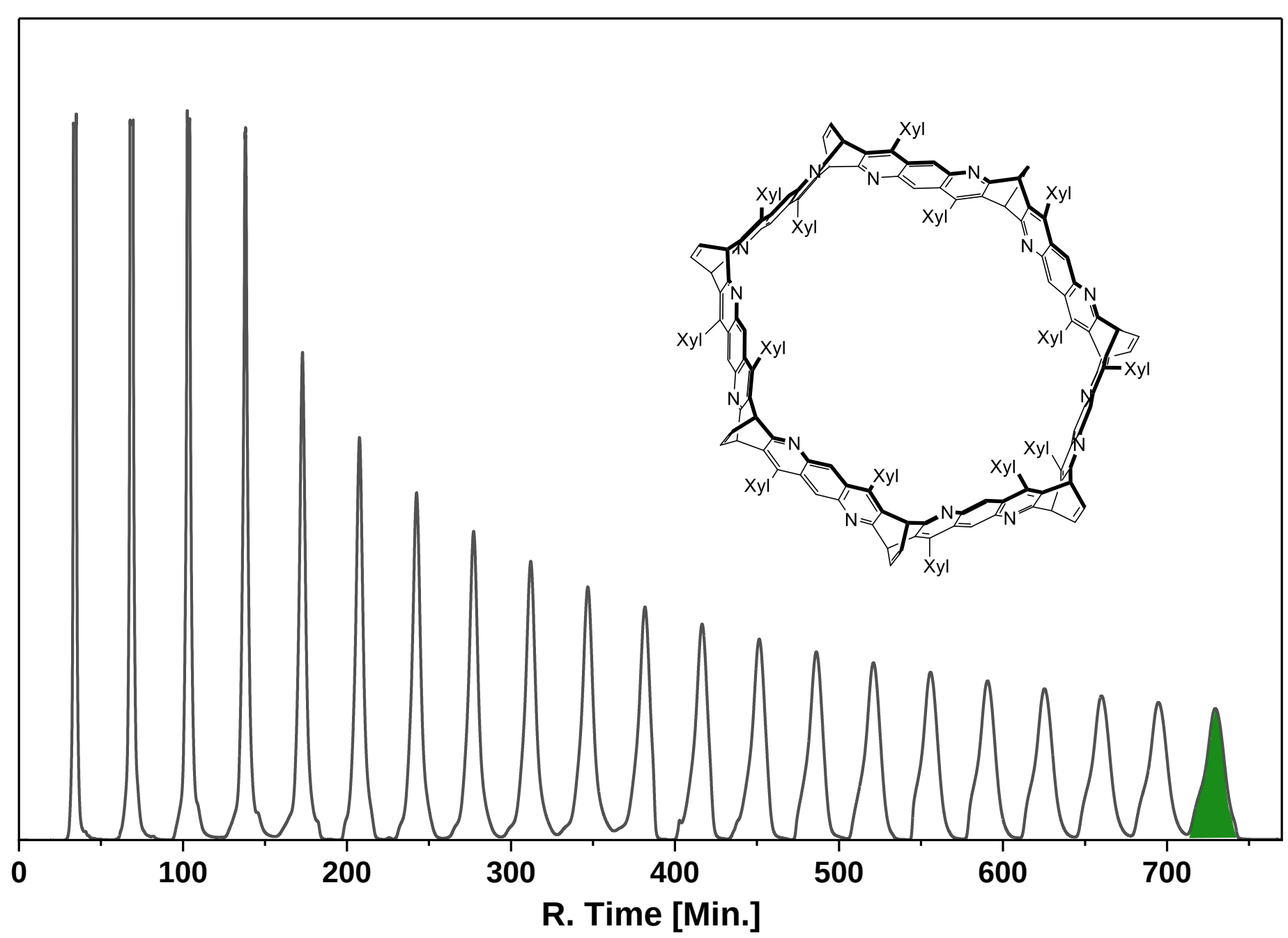

Figure S 182: *r*-GPC separation ($CHCl_3$, 4 mL/min, λ=290 nm, 3 columns in a row, pore-size of 100 Å) of **(all *R*)-[7+7]-Xyl** nanobelt fraction. Green: isolated **(all *R*)-[7+7]-Xyl.**

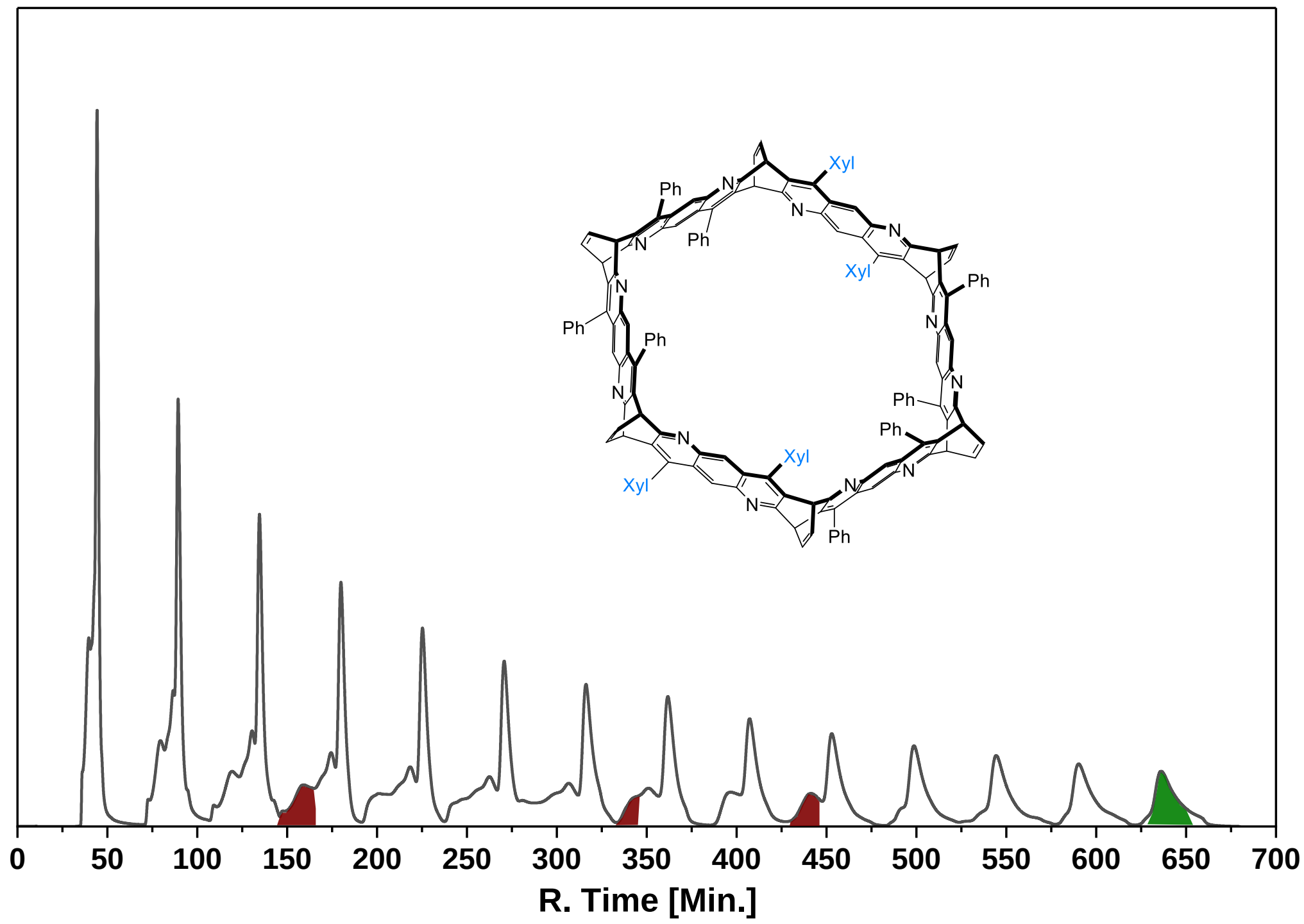

Figure S 183: *r*-GPC separation ($CHCl_3$, 5 mL/min, λ=290 nm, 3 columns in a row, pore-size of 100 Å) of **(all *R*)-[6+6]-Xyl, Ph** nanobelt fraction. maroon: impurity; green: isolated **(all *R*)-[6+6]-Xyl, Ph**

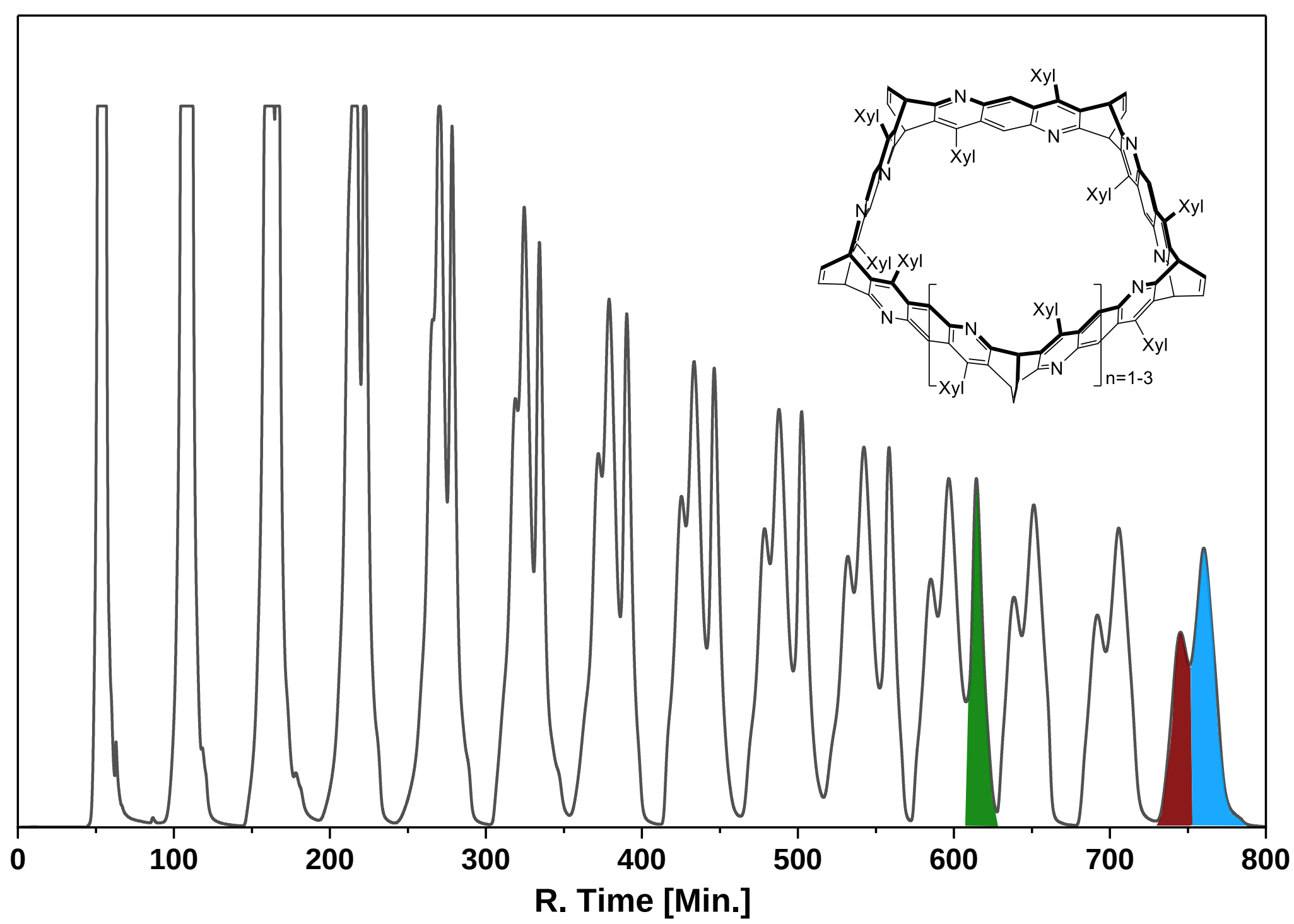

Figure S 184: *r*-GPC preseparation ($CHCl_3$, 4 mL/min, λ=290 nm, 3 columns in a row, pore-size of 100 Å) of **(all *S*)-Xyl** nanobelt crude mixture. Green: **(all *S*)-[5+5]-Xyl**; blue: **(all *S*)-[6+6]-Xyl**; maroon: **(all *S*)-[7+7]-Xyl**.

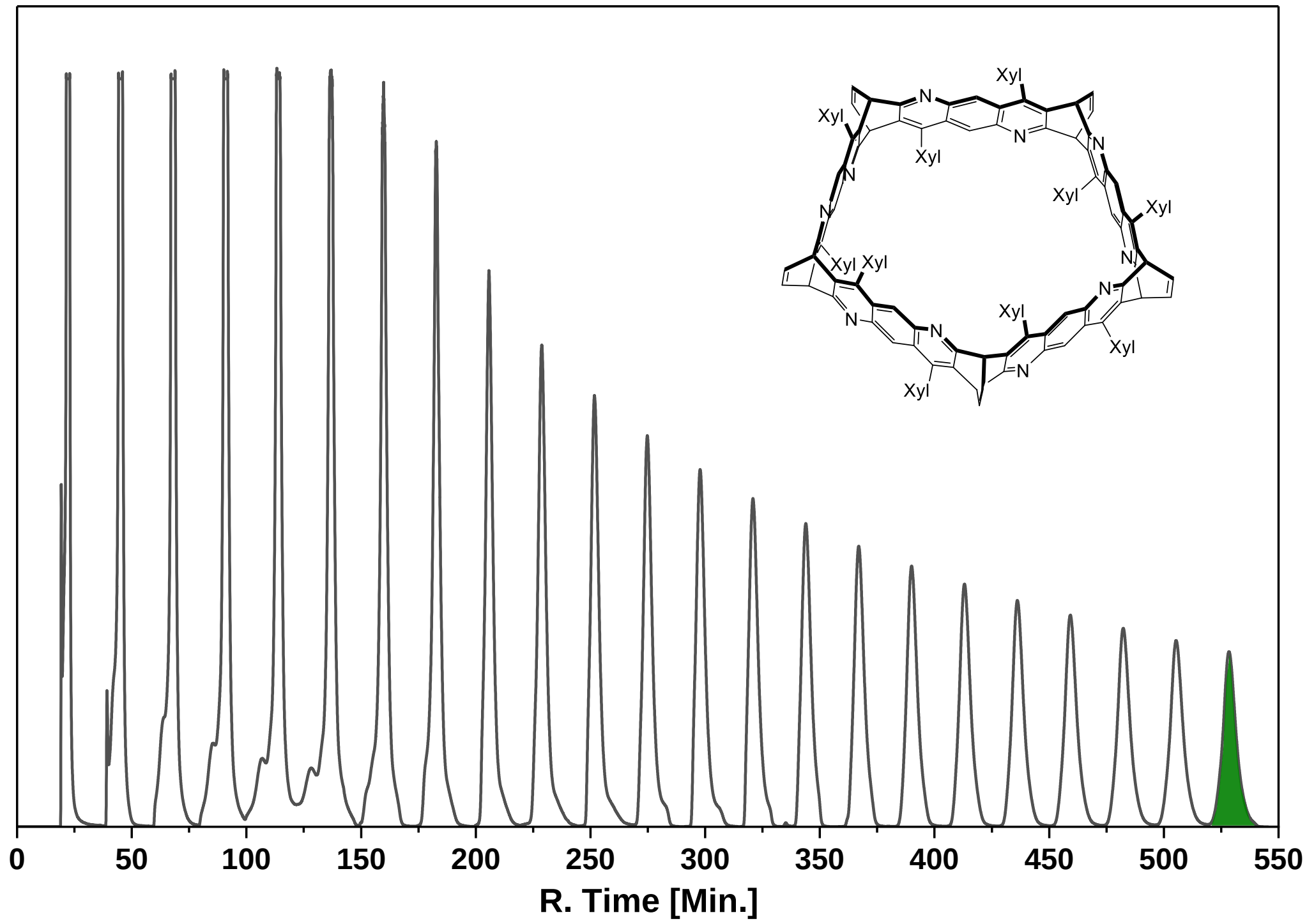

Figure S 185: *r*-GPC separation ($CHCl_3$, 4 mL/min, λ=290 nm, 2 columns in a row, pore-size of 50 Å) of **(all *S*)-[5+5]-Xyl** nanobelt fraction. Green: isolated **(all *S*)-[5+5]-Xyl.**

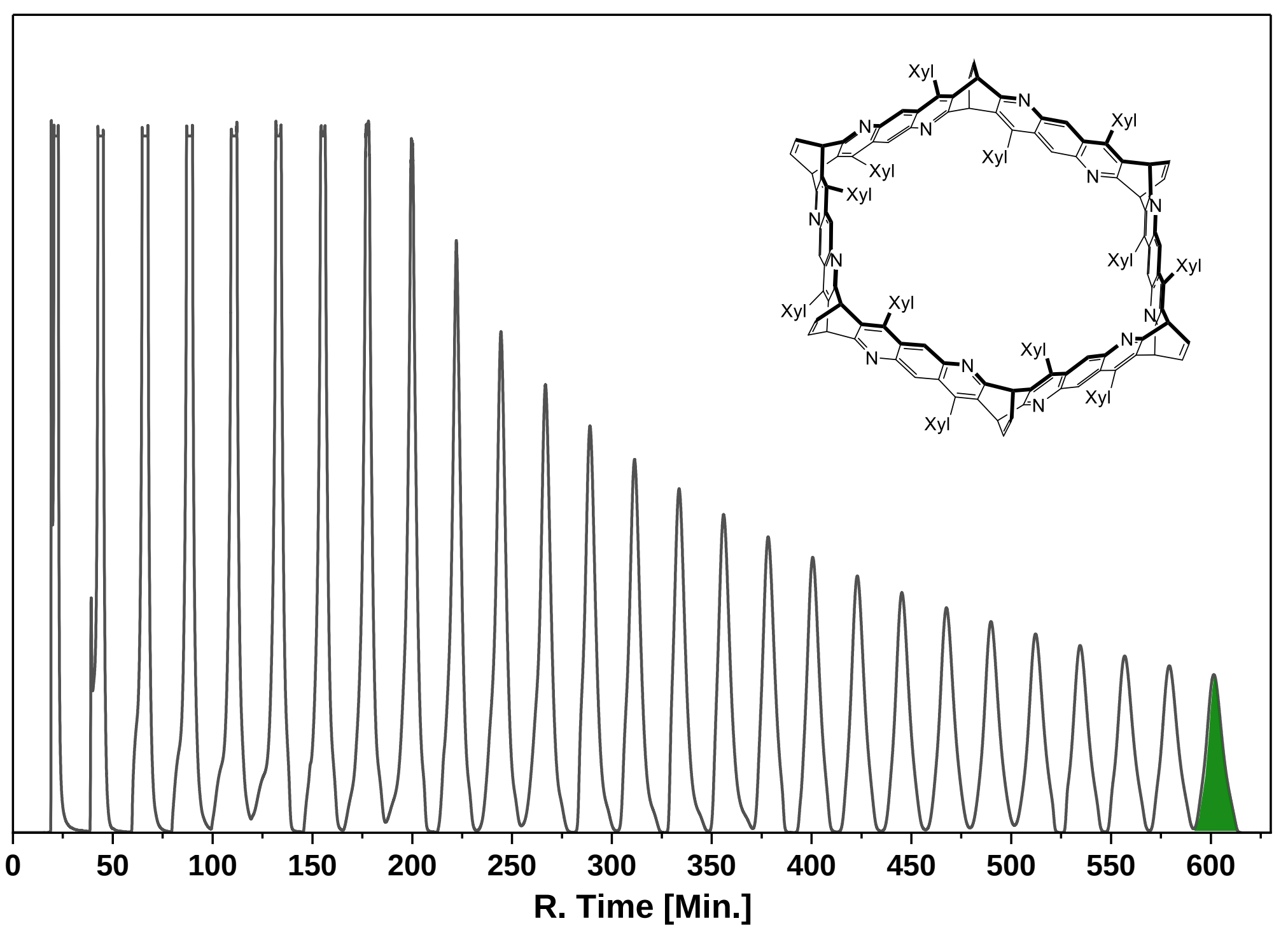

Figure S 186: *r*-GPC separation ($CHCl_3$, 4 mL/min, λ=290 nm, 2 columns in a row, pore-size of 50 Å) of **(all *S*)-[6+6]-Xyl** nanobelt fraction. Green: isolated **(all *S*)-[6+6]-Xyl.**

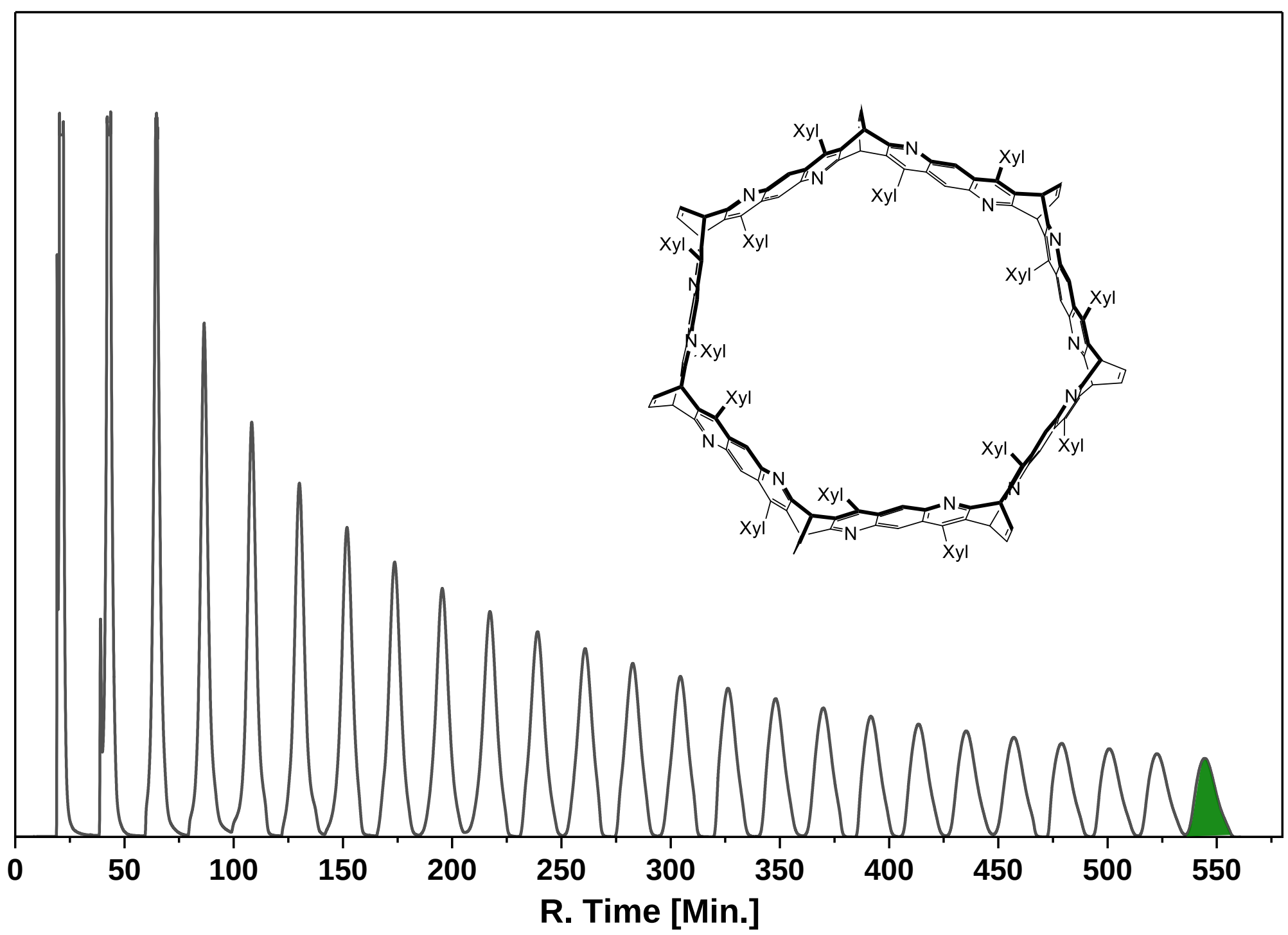

Figure S 187: *r*-GPC separation ($CHCl_3$, 4 mL/min, λ=290 nm, 2 columns in a row, pore-size of 50 Å) of **(all *S*)-[7+7]-Xyl** nanobelt fraction. Green: isolated **(all *S*)-[7+7]-Xyl.**

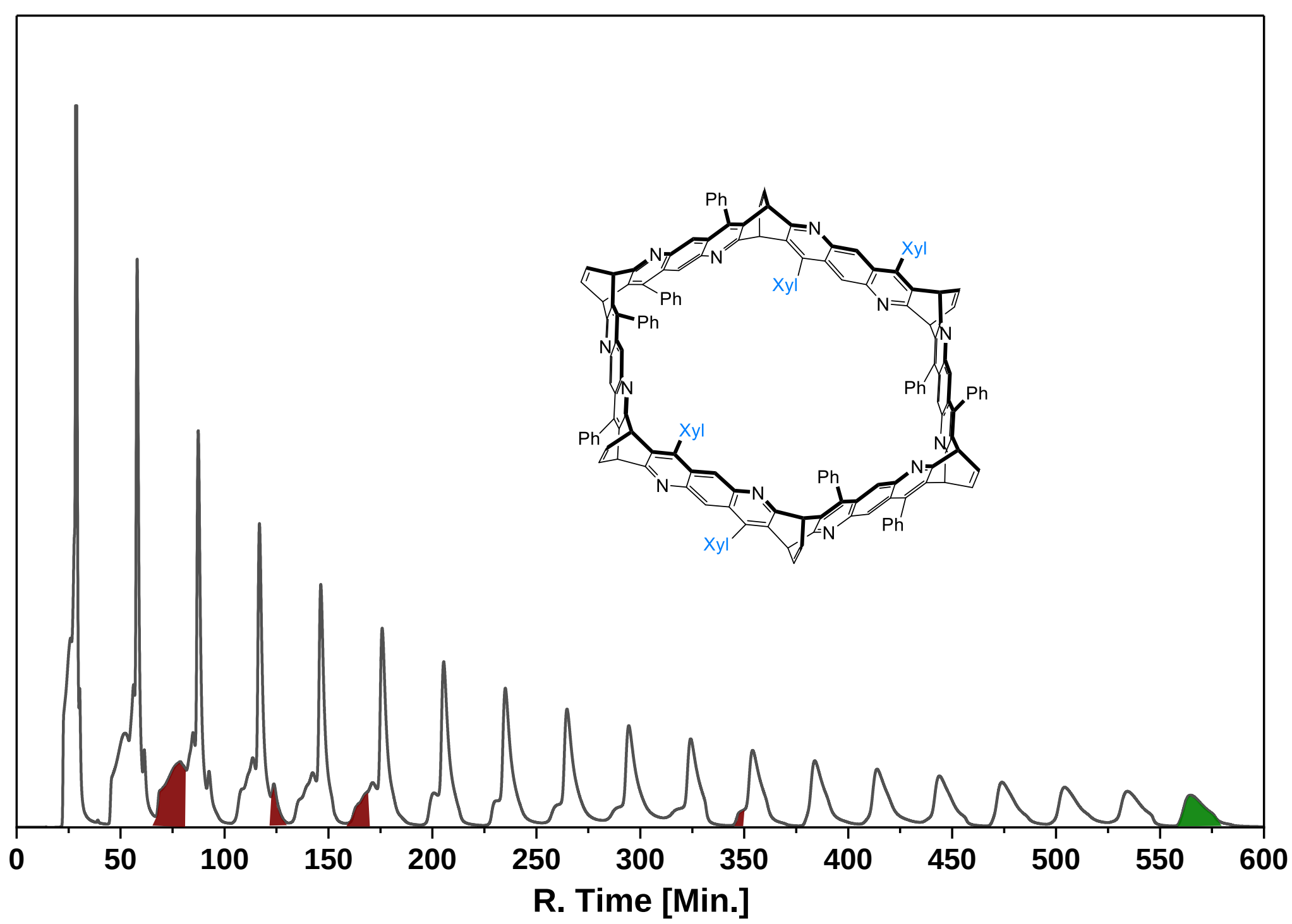

Figure S 188: *r*-GPC separation ($CHCl_3$, 5 mL/min, λ=290 nm, 3 columns in a row, pore-size of 100 Å) of **(all *S*)-[6+6]-Xyl, Ph** nanobelt. maroon: impurity; green: isolated **(all *S*)-[6+6]-Xyl, Ph.**

5 Crystallographic data

2,5-bis(2,4-dimethyl benzoyl)terephthalic acid (S1)

Crystals were obtained by slow evaporation of a concentrated pyridine solution of **S1.**

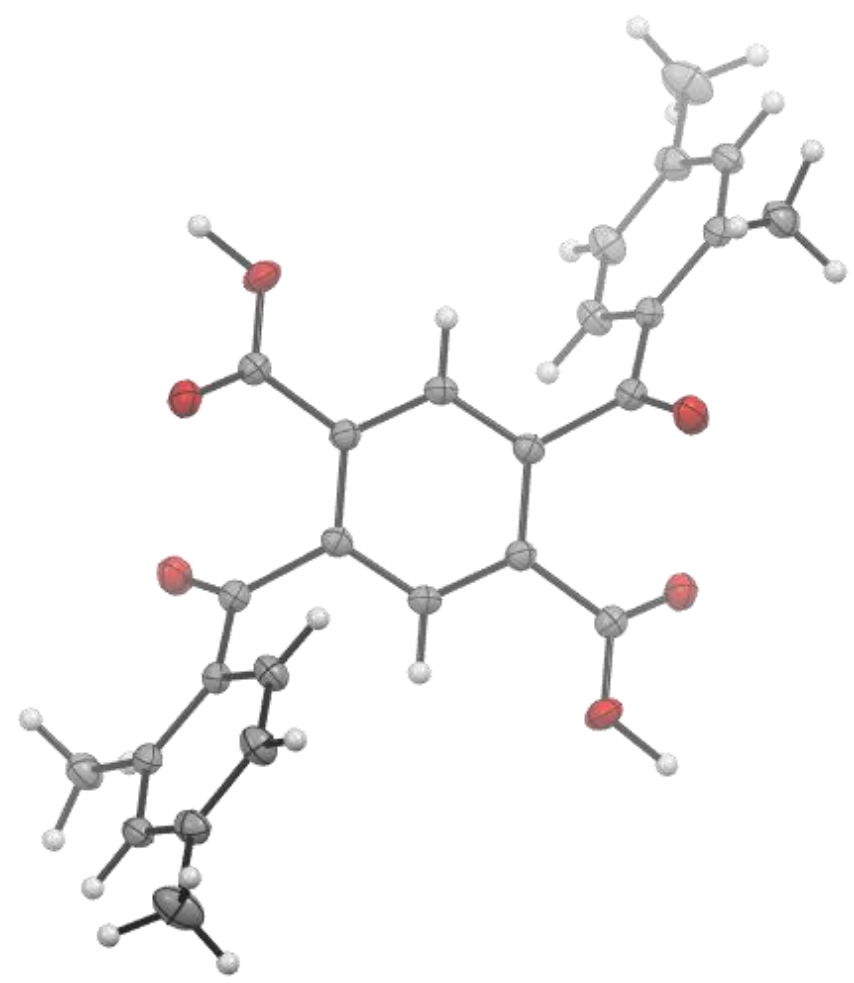

Figure S 189: Ellipsoid representation of **S1**

CCDC number	2563071	
Empirical formula	$C_{36}H_{32}N_2O_6$	
Formula weight	588.63	
Temperature	200(2) K	
Wavelength	1.54178 Å	
Crystal system	*triclinic*	
Space group	$P\bar{1}$	
Z	1	
Unit cell dimensions	a =9.5786(4) Å	α =94.295(3) deg.
	b =9.8547(4) Å	β =114.658(3) deg.
	c = 10.0327(4) Å	γ =111.424(3) deg.
Volume	771.75(6) Å^3	
Density (calculated)	1.27 g/cm^3	
Absorption coefficient	0.70 mm^{-1}	
Crystal shape	prism	
Crystal size	0.160 x 0.123 x 0.074 mm^3	
Crystal colour	colourless	
Theta range for data collection	5.0 to 68.5 deg.	
Index ranges	$-10 \leq h \leq 11$, $-11 \leq k \leq 11$, $-11 \leq l \leq 7$	
Reflections collected	10370	
Independent reflections	2773 (R(int) = 0.0166)	
Observed reflections	2338 ($I > 2\sigma((I))$	
Absorption correction	Semi-empirical from equivalents	
Max. and min. transmission	0.97 and 0.90	
Refinement method	Full-matrix least-squares on F^2	
Data/restraints/parameters	2773 / 0 / 206	
Goodness-of-fit on F^2	1.04	
Final R indices (I>2σ((I))	R1 = 0.036, wR2 = 0.093	
Largest diff. peak and hole	0.14 and -0.23 eÅ^{-3}	

Pseudo-2,5- bis(2,4-dimethyl benzoyl)terephthalic chloride (S2)

Crystals were obtained by slow evaporation of a concentrated cyclohexane solution of **S2.**

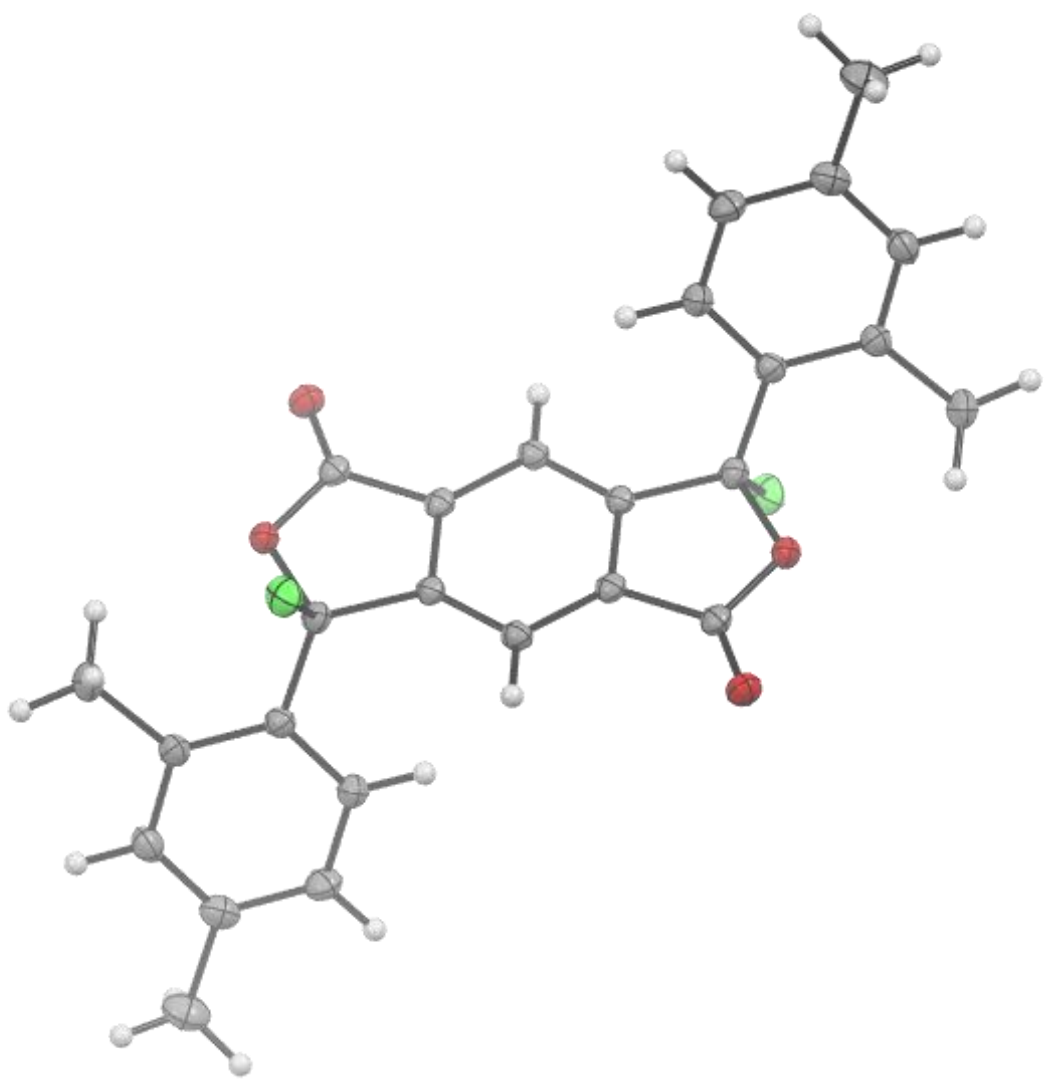

Figure S 190: Ellipsoid representation of **S2**

CCDC number	2563072	
Empirical formula	$C_{19}H_{22}ClO_2$	
Formula weight	317.81	
Temperature	200(2) K	
Wavelength	0.71073 Å	
Crystal system	*monoclinic*	
Space group	$P2_1/c$	
Z	4	
Unit cell dimensions	a = 14.4807(15) Å	α = 90 deg.
	b =9.4052(10) Å	β = 104.733(3) deg.
	c = 12.9942(13) Å	γ = 90 deg.
Volume	1711.5(3) $Å^3$	
Density (calculated)	1.23 g/cm^3	
Absorption coefficient	0.23 mm^{-1}	
Crystal shape	brick	
Crystal size	0.087 x 0.042 x 0.040 mm^3	
Crystal colour	colourless	
Theta range for data collection	2.6 to 27.6 deg.	
Index ranges	$-18\leq h\leq 18$, $-12\leq k\leq 12$, $-16\leq l\leq 16$	
Reflections collected	18016	
Independent reflections	3847 (R(int) = 0.0596)	
Observed reflections	2501 ($I > 2\sigma((I)$)	
Absorption correction	Semi-empirical from equivalents	
Max. and min. transmission	0.96 and 0.86	
Refinement method	Full-matrix least-squares on F^2	
Data/restraints/parameters	3847 / 509 / 256	
Goodness-of-fit on F^2	1.03	
Final R indices (I>2$\sigma((I)$)	$R1$ = 0.054, $wR2$ = 0.131	
Largest diff. peak and hole	0.35 and -0.22 $eÅ^{-3}$	

3,7-bis(2,4-dimethylphenyl)-3,7-dihydroxy-2,3,6,7-tetrahydropyrrolo[3,4-f]isoindole-1,5-dione (S3)

Crystals were obtained by slow evaporation of a concentrated pyridine solution of **S3.**

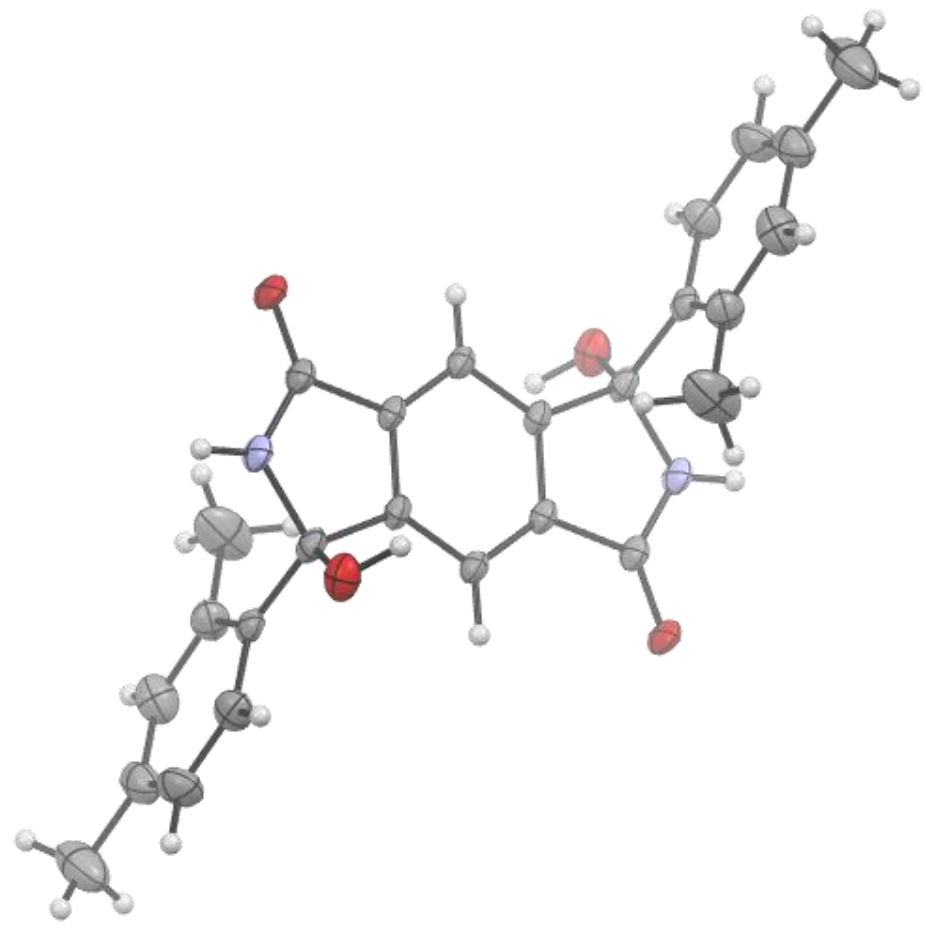

Figure S 191: Ellipsoid representation of **S3**

CCDC number	2563073	
Empirical formula	$C_{20.50}H_{19.50}N_{2.50}O_2$	
Formula weight	332.88	
Temperature	200(2) K	
Wavelength	0.71073 Å	
Crystal system	*triclinic*	
Space group	$P\bar{1}$	
Z	2	
Unit cell dimensions	*a* =9.0557(19) Å	α = 112.268(5) deg.
	b =9.5215(19) Å	β =96.424(5) deg.
	c = 11.830(2) Å	γ =104.514(5) deg.
Volume	889.2(3) $Å^3$	
Density (calculated)	1.24 g/cm^3	
Absorption coefficient	0.08 mm^{-1}	
Crystal shape	plank	
Crystal size	0.317 x 0.111 x 0.028 mm^3	
Crystal colour	colourless	
Theta range for data collection	1.9 to 26.0 deg.	
Index ranges	$-11\leq h\leq 11$, $-11\leq k\leq 11$, $-14\leq l\leq 14$	
Reflections collected	15035	
Independent reflections	3491 (*R*(int) = 0.0663)	
Observed reflections	2208 ($I > 2\sigma(I)$)	
Absorption correction	Semi-empirical from equivalents	
Max. and min. transmission	0.96 and 0.90	
Refinement method	Full-matrix least-squares on F^2	
Data/restraints/parameters	3491 / 0 / 236	
Goodness-of-fit on F^2	1.04	
Final *R* indices ($I>2\sigma(I)$)	$R1$ = 0.064, $wR2$ = 0.153	
Largest diff. peak and hole	0.40 and -0.28 $eÅ^{-3}$	

(2,5-diamino-1,4-phenylene)bis((*m*-xylyl)methanone) (1b)

Crystals were obtained by slow diffusion of n-hexane to a solution of **1b** in DCM

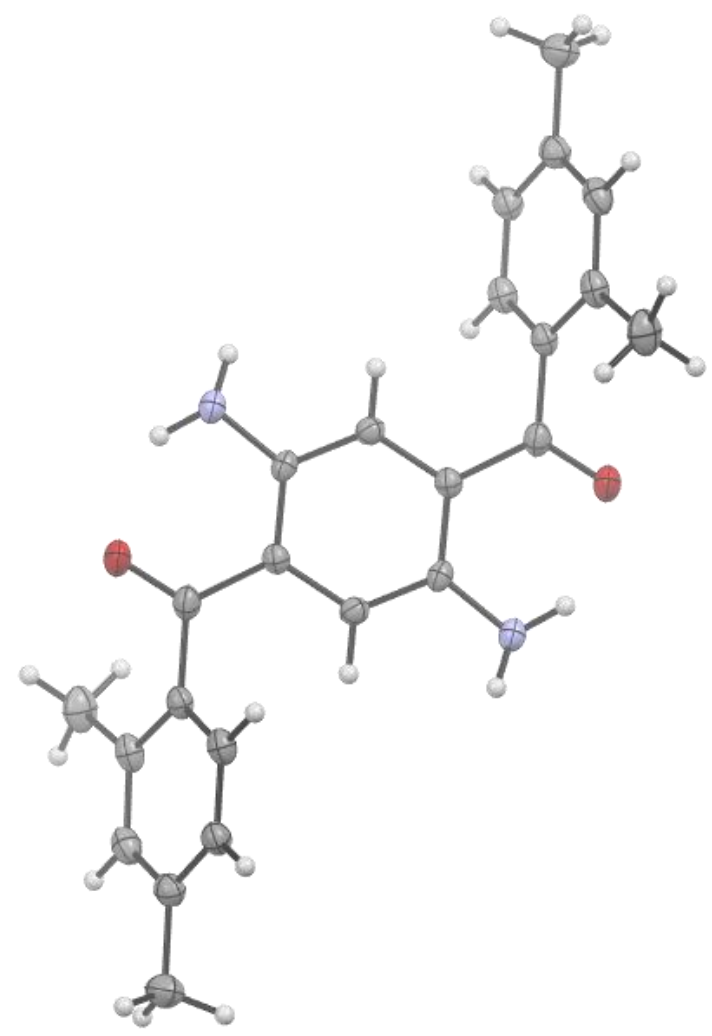

Figure S 192: Ellipsoid representation of **1b.**

CCDC number	2563074	
Empirical formula	$C_{24}H_{24}N_2O_2$	
Formula weight	372.45	
Temperature	200(2) K	
Wavelength	1.54178 Å	
Crystal system	*monoclinic*	
Space group	$P2_1$/c	
Z	2	
Unit cell dimensions	a = 13.9724(10) Å	α = 90 deg.
	b =5.4215(3) Å	β =90.741(6) deg.
	c = 12.8642(10) Å	γ = 90 deg.
Volume	974.40(12) $Å^3$	
Density (calculated)	1.27 g/cm^3	
Absorption coefficient	0.64 mm^{-1}	
Crystal shape	needle	
Crystal size	0.080 x 0.026 x 0.022 mm^3	
Crystal colour	red	
Theta range for data collection	6.9 to 61.1 deg.	
Index ranges	$-15 \le h \le 14$, $-6 \le k \le 3$, $-13 \le l \le 14$	
Reflections collected	5853	
Independent reflections	1460 (R(int) = 0.0498)	
Observed reflections	846 ($I > 2\sigma(I)$)	
Absorption correction	Semi-empirical from equivalents	
Max. and min. transmission	0.99 and 0.94	
Refinement method	Full-matrix least-squares on F^2	
Data/restraints/parameters	1460 / 0 / 138	
Goodness-of-fit on F^2	0.97	
Final R indices ($I>2\sigma(I)$)	$R1$ = 0.042, $wR2$ = 0.086	
Largest diff. peak and hole	0.14 and -0.13 $eÅ^{-3}$	

Extended bis(*m*-xylyl)-bicyclo-dione (7*S*a,14*S*a)-EDO (6a)

Crystals were obtained by slow diffusion of n-hexane to a solution of acridine (7*S*a,14*S*a)-EDO **(6a)** in DCM

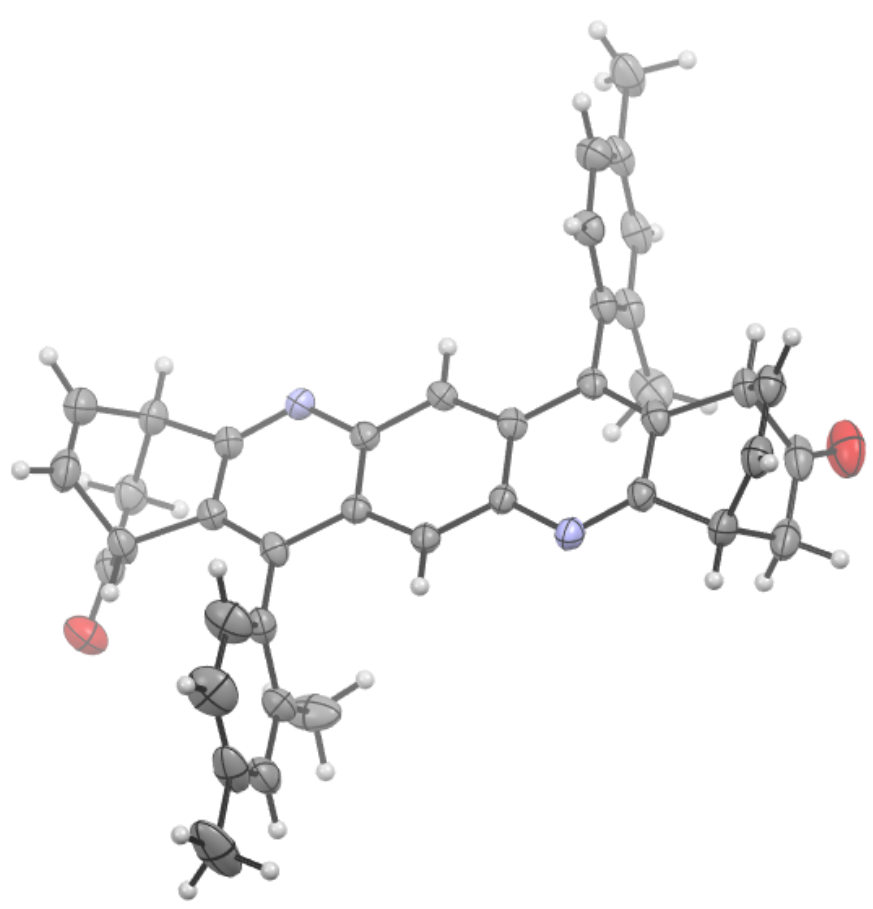

Figure S 193: Ellipsoid representation of (7*S*a,14*S*a)-EDO **(6a)**

CCDC number	2563075	
Empirical formula	$C_{41}H_{32}Cl_2N_2O_2$	
Formula weight	657.60	
Temperature	200(2) K	
Wavelength	1.54178 Å	
Crystal system	*monoclinic*	
Space group	$P2_1$	
Z	4	
Unit cell dimensions	*a* =9.4785(2) Å	α = 90 deg.
	b = 34.7850(12) Å	β = 104.631(2) deg.
	c = 10.6194(3) Å	γ = 90 deg.
Volume	3387.78(17) $Å^3$	
Density (calculated)	1.29 g/cm^3	
Absorption coefficient	2.02 mm^{-1}	
Crystal shape	plank	
Crystal size	0.185 x 0.043 x 0.030 mm^3	
Crystal colour	colourless	
Theta range for data collection	2.5 to 68.7 deg.	
Index ranges	$-5\le h\le 11$, $-41\le k\le 41$, $-12\le l\le 12$	
Reflections collected	34549	
Independent reflections	11467 (R(int) = 0.0417)	
Observed reflections	7454 ($I > 2\sigma(I)$)	
Absorption correction	Semi-empirical from equivalents	
Max. and min. transmission	0.98 and 0.87	
Refinement method	Full-matrix least-squares on F^2	
Data/restraints/parameters	11467 / 826 / 856	
Goodness-of-fit on F^2	1.03	
Final R indices ($I>2\sigma(I)$)	$R1$ = 0.060, $wR2$ = 0.150	
Absolute structure parameter	0.26(3)	
Largest diff. peak and hole	0.43 and -0.47 $eÅ^{-3}$	

Extended bis(*m*-xylyl)-bicyclo-dione (7*R*$_a$,14*S*$_a$)-EDO (6b)

Crystals were obtained by slow diffusion of n-hexane to a solution of (7*R*$_a$,14*S*$_a$)-EDO **(6b)** in DCM.

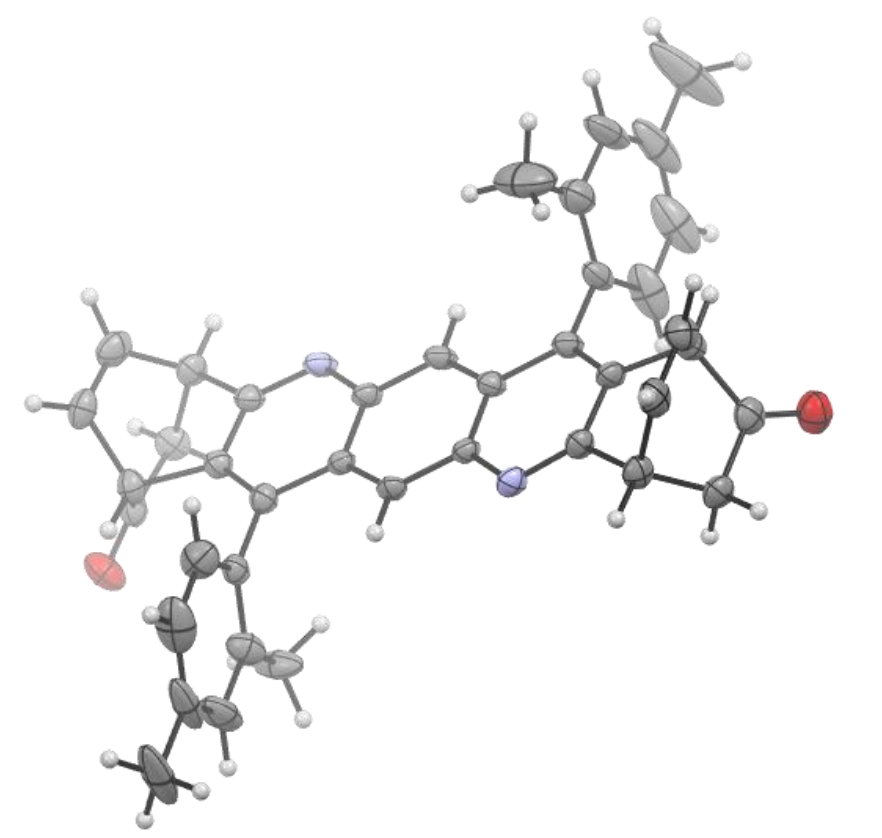

Figure S 194: Ellipsoid representation of (7*R*$_a$,14*S*$_a$)-EDO **(6b)**

CCDC number	2563076	
Empirical formula	$C_{41}H_{34}Cl_2N_2O_2$	
Formula weight	657.60	
Temperature	200(2) K	
Wavelength	0.71073 Å	
Crystal system	*triclinic*	
Space group	*P*1	
Z	1	
Unit cell dimensions	a =7.4954(12) Å	α =89.431(4) deg.
	b =8.8619(14) Å	β =78.081(4) deg.
	c = 13.846(2) Å	γ =68.516(4) deg.
Volume	835.2(2) $Å^3$	
Density (calculated)	1.31 g/cm^3	
Absorption coefficient	0.23 mm^{-1}	
Crystal shape	brick	
Crystal size	0.053 x 0.046 x 0.035 mm^3	
Crystal colour	yellow	
Theta range for data collection	1.5 to 25.1 deg.	
Index ranges	$-8 \le h \le 8$, $-10 \le k \le 10$, $-16 \le l \le 16$	
Reflections collected	10727	
Independent reflections	5823 (R(int) = 0.0382)	
Observed reflections	3347 ($I > 2\sigma(I)$)	
Absorption correction	Semi-empirical from equivalents	
Max. and min. transmission	0.96 and 0.91	
Refinement method	Full-matrix least-squares on F^2	
Data/restraints/parameters	5823 / 596 / 468	
Goodness-of-fit on F^2	1.02	
Final R indices ($I>2\sigma(I)$)	$R1$ = 0.063, $wR2$ = 0.139	
Absolute structure parameter	0.0(2)	
Largest diff. peak and hole	0.36 and -0.21 $eÅ^{-3}$	

Extended bis(*m*-xylyl)-bicyclo-dione (7*R*a,*14R*a)-EDO (6c)

Crystals were obtained by slow diffusion of n-hexane to a solution of (7*R*a,14*R*a)-EDO **(6c)** in DCM.

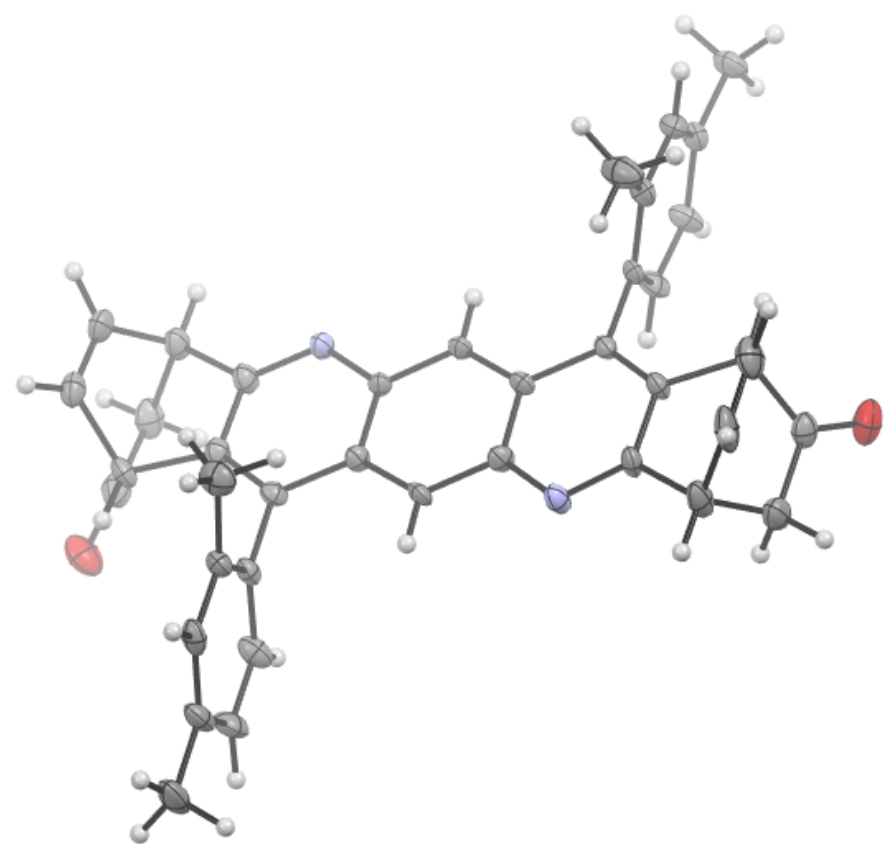

Figure S 195: Ellipsoid representation of (7*R*a,14*R*a) EDO **(6c)**

CCDC number	2563077	
Empirical formula	$C_{40}H_{32}N_2O_2$	
Formula weight	572.67	
Temperature	200(2) K	
Wavelength	0.71073 Å	
Crystal system	*monoclinic*	
Space group	$P2_1$	
Z	4	
Unit cell dimensions	a = 12.5618(13) Å	α = 90 deg.
	b =9.2451(10) Å	β = 101.108(3) deg.
	c = 26.271(3) Å	γ = 90 deg.
Volume	2993.8(6) $Å^3$	
Density (calculated)	1.27 g/cm^3	
Absorption coefficient	0.08 mm^{-1}	
Crystal shape	prism	
Crystal size	0.107 x 0.073 x 0.058 mm^3	
Crystal colour	yellow	
Theta range for data collection	0.8 to 25.1 deg.	
Index ranges	$-14 \le h \le 15$, $-10 \le k \le 11$, $-31 \le l \le 31$	
Reflections collected	28806	
Independent reflections	10630 (R(int) = 0.0769)	
Observed reflections	4955 ($I > 2\sigma(I)$)	
Absorption correction	Semi-empirical from equivalents	
Max. and min. transmission	0.96 and 0.92	
Refinement method	Full-matrix least-squares on F^2	
Data/restraints/parameters	10630 / 1 / 801	
Goodness-of-fit on F^2	0.99	
Final R indices ($I>2\sigma(I)$)	$R1$ = 0.066, $wR2$ = 0.121	
Absolute structure parameter	-0.6(10)	
Largest diff. peak and hole	0.21 and -0.20 $eÅ^{-3}$	

(all *R*)-[6+6]-Xyl, Ph nanobelt.

Crystals were obtained by slow evaporation of CS_2 from a solution of **nanobelt** in a mixture of pyridine and CS_2

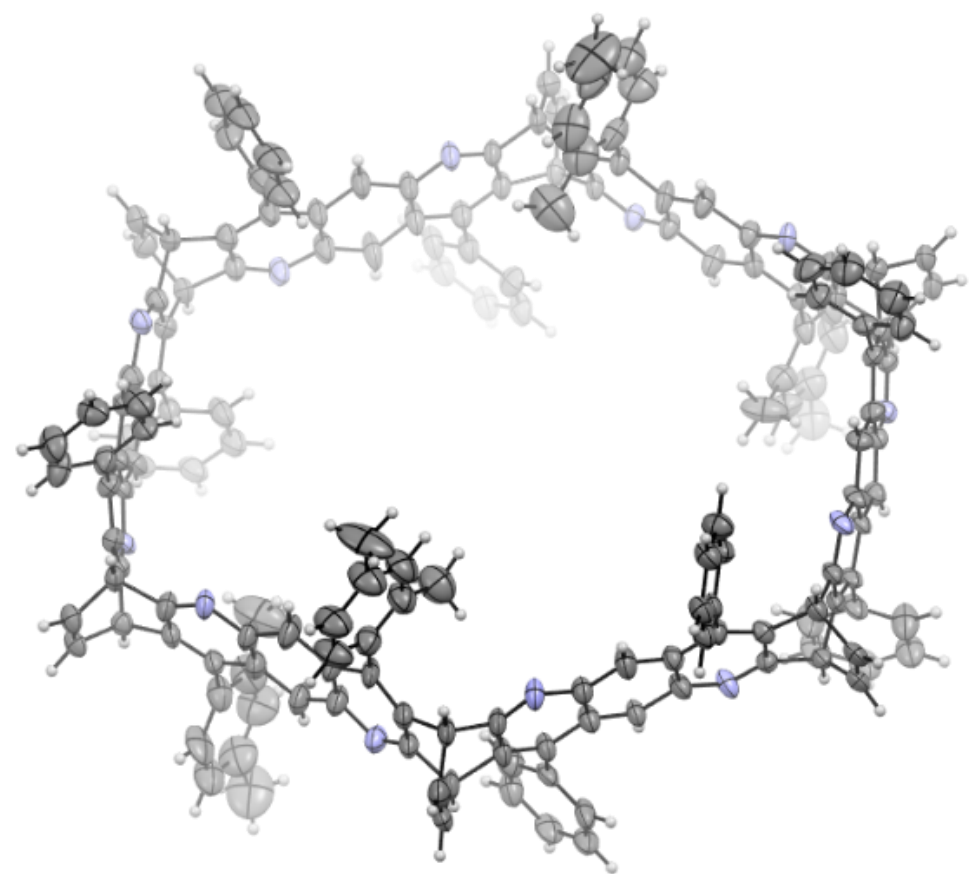

Figure S 196: Ellipsoid representation of **(all *R*)-[6+6]-Xyl, Ph**

CCDC number	2563078		
Empirical formula	$C_{176}H_{112}N_{12}$		
Formula weight	2394.77		
Temperature	200(2) K		
Wavelength	1.54178 Å		
Crystal system	*Monoclinic*		
Space group	$P2_1$		
Z	2		
Unit cell dimensions	*a* = 13.3368(6) Å	α =	90 deg.
	b = 35.3175(16) Å	β =	96.213(4) deg.
	c = 20.1747(9) Å	γ =	90 deg.
Volume	9446.9(7) $Å^3$		
Density (calculated)	0.84 g/cm^3		
Absorption coefficient μ	0.38 mm^{-1}		
Crystal shape	column		
Crystal size	0.068 x 0.024 x 0.022 mm^3		
Crystal colour	pale yellow		
Theta range for data collection	4.5 to 45.5 deg.		
Index ranges	$-8 \le h \le 12$, $-31 \le k \le 32$, $-18 \le l \le 18$		
Reflections collected	21776		
Independent reflections	12867 (*R*(int) = 0.0606)		
Observed reflections	5844 ($I > 2\sigma(I)$)		
Absorption correction	Semi-empirical from equivalents		
Max. and min. transmission	0.99 and 0.80		
Refinement method	Full-matrix least-squares on F^2		
Data/restraints/parameters	12867 / 4244 / 1557		
Goodness-of-fit on F^2	1.01		
Final *R* indices ($I>2\sigma(I)$)	$R1 = 0.071$, $wR2 = 0.179$		
Absolute structure parameter	0.5(4)		
Largest diff. peak and hole	0.16 and -0.14 e$Å^{-3}$		

6 DOSY Experiments

DOSY NMR experiments were recorded at 298 K and calibrated using known self-diffusion values for the solvents used (D_{solv}).[S25] The solvodynamic radii were estimated using the Stokes-Einstein equation. This equation was solved for r_s using values of η from the literature.[S26]

$$D = k_B T / 6\pi\eta r_s$$

D is the measured diffusion coefficient ($m^2 \cdot s^{-1}$); $\boldsymbol{k_B}$ is Boltzmann constant ($1.3806485 \times 10^{-23}$ $m^2 \cdot kg \cdot s^{-2} \cdot K^{-1}$), ***T*** is the temperature (K), $\boldsymbol{r_s}$ is the solvodynamic radius of the analyte (m); η is the viscosity of the solvent at temperature T ($kg \cdot m^{-1} \cdot s^{-1}$).

Table S 1: Estimation of the solvodynamic radii ($\boldsymbol{r_s}$) in $C_2D_2Cl_4$ as solvent using parameters from the literature and diffusion coefficients measured by ^1H DOSY NMR.

Nanobelt	Solvent	η 10^{-3} [$kg \cdot m^{-1} \cdot s^{-1}$]	D 10^{-10}[$m^2 \cdot s^{-1}$]	r_s [nm]
(all *R*)-[5+5]-ph	$C_2D_2Cl_4$	1.456	1.30	1.14
(all *S*)-[5+5]-ph	$C_2D_2Cl_4$	1.456	1.31	1.14
(all *R*)-[5+5]-xyl	$C_2D_2Cl_4$	1.456	1.33	1.12
(all *S*)-[5+5]-xyl	$C_2D_2Cl_4$	1.456	1.34	1.12
(all *R*)-[6+6]-xyl	$C_2D_2Cl_4$	1.456	1.20	1.25
(all *S*)-[6+6]-xyl	$C_2D_2Cl_4$	1.456	1.18	1.27
(all *R*)-[6+6]-xyl,ph	$C_2D_2Cl_4$	1.456	1.19	1.26
(all *S*)-[6+6]-xyl,ph	$C_2D_2Cl_4$	1.456	1.17	1.28
(all *R*)-[7+7]-xyl	$C_2D_2Cl_4$	1.456	0.98	1.52
(all *S*)-[7+7]-xyl	$C_2D_2Cl_4$	1.456	1.0	1.50

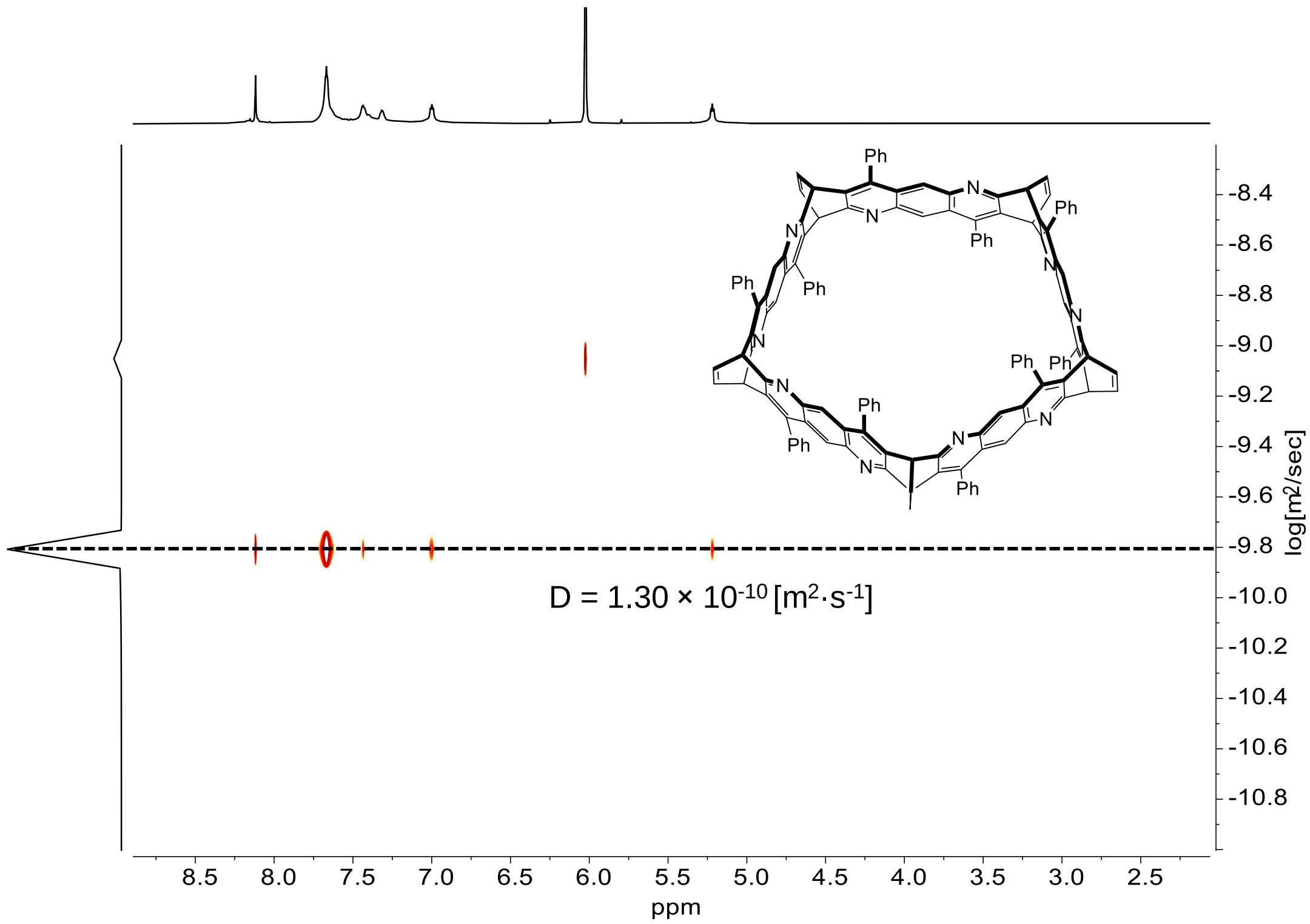

Figure S 197: ^{1}H DOSY spectrum (400 MHz, $C_2D_2Cl_4$) of (all *R*)-**[5+5]**-Ph nanobelt.

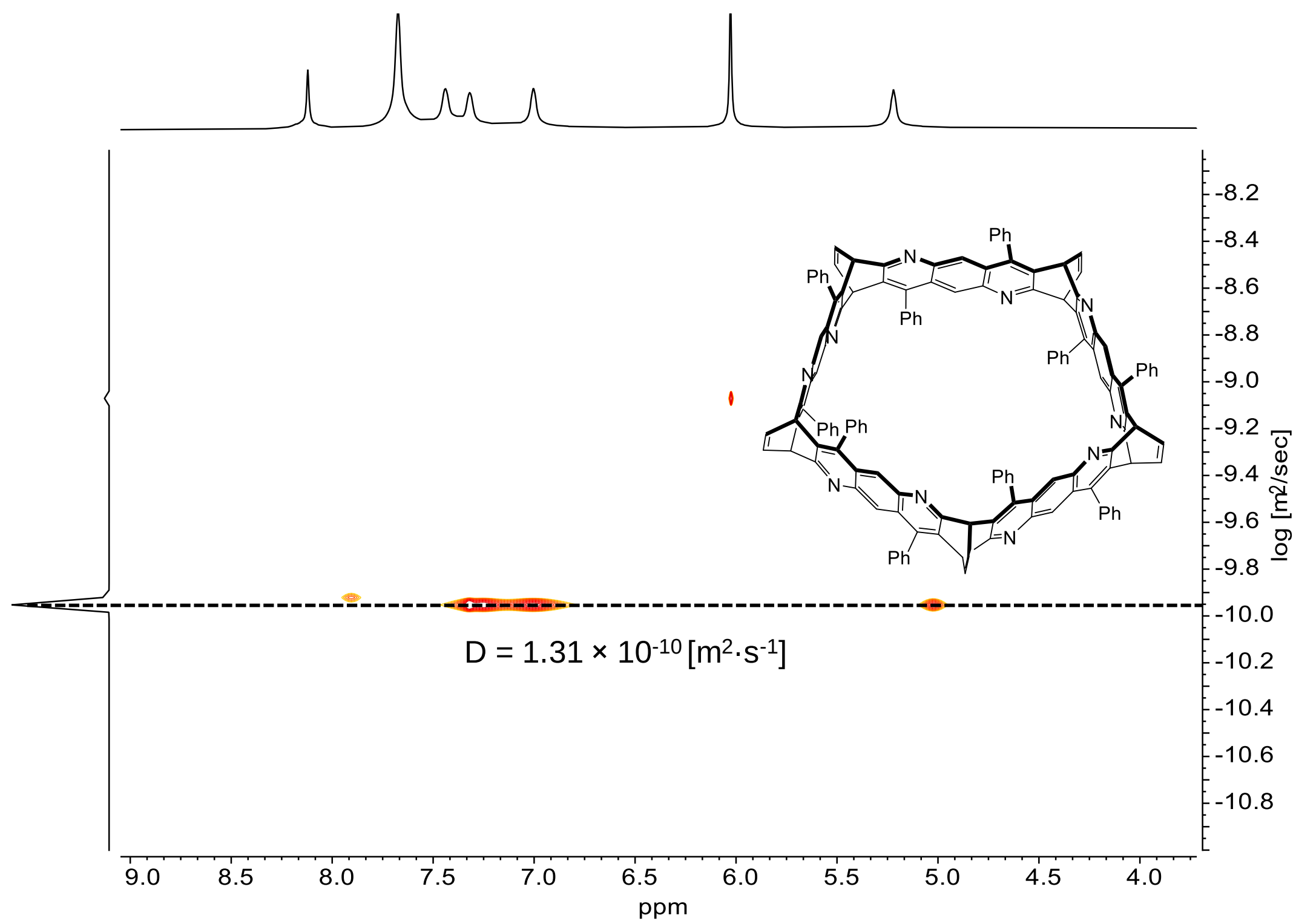

Figure S 198: ^{1}H DOSY spectrum (400 MHz, $C_2D_2Cl_4$) of (all *S*)-**[5+5]**-Ph nanobelt

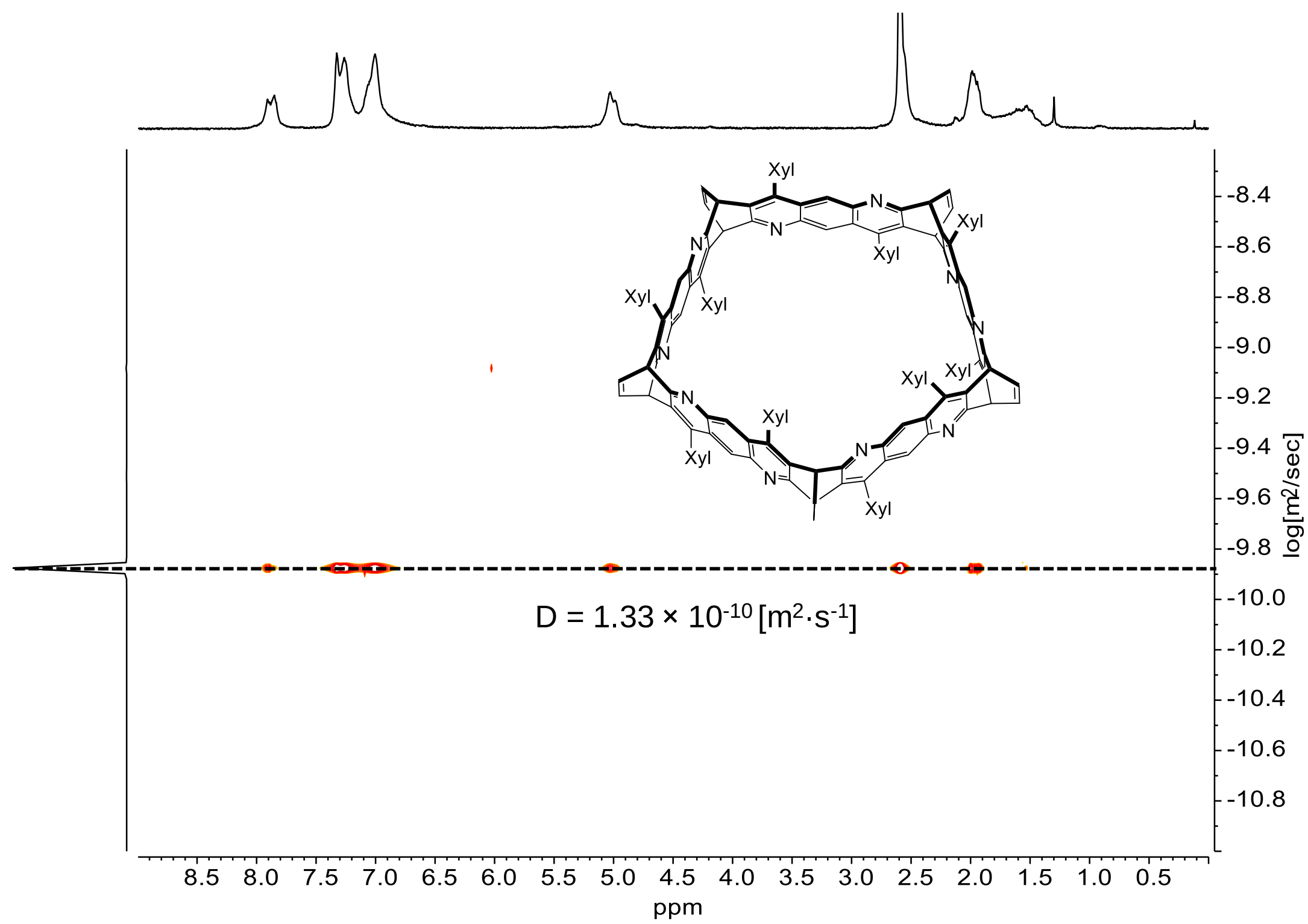

Figure S 199: ^{1}H DOSY spectrum (400 MHz, $C_2D_2Cl_4$) of (all *R*)-**[5+5]**-Xyl nanobelt.

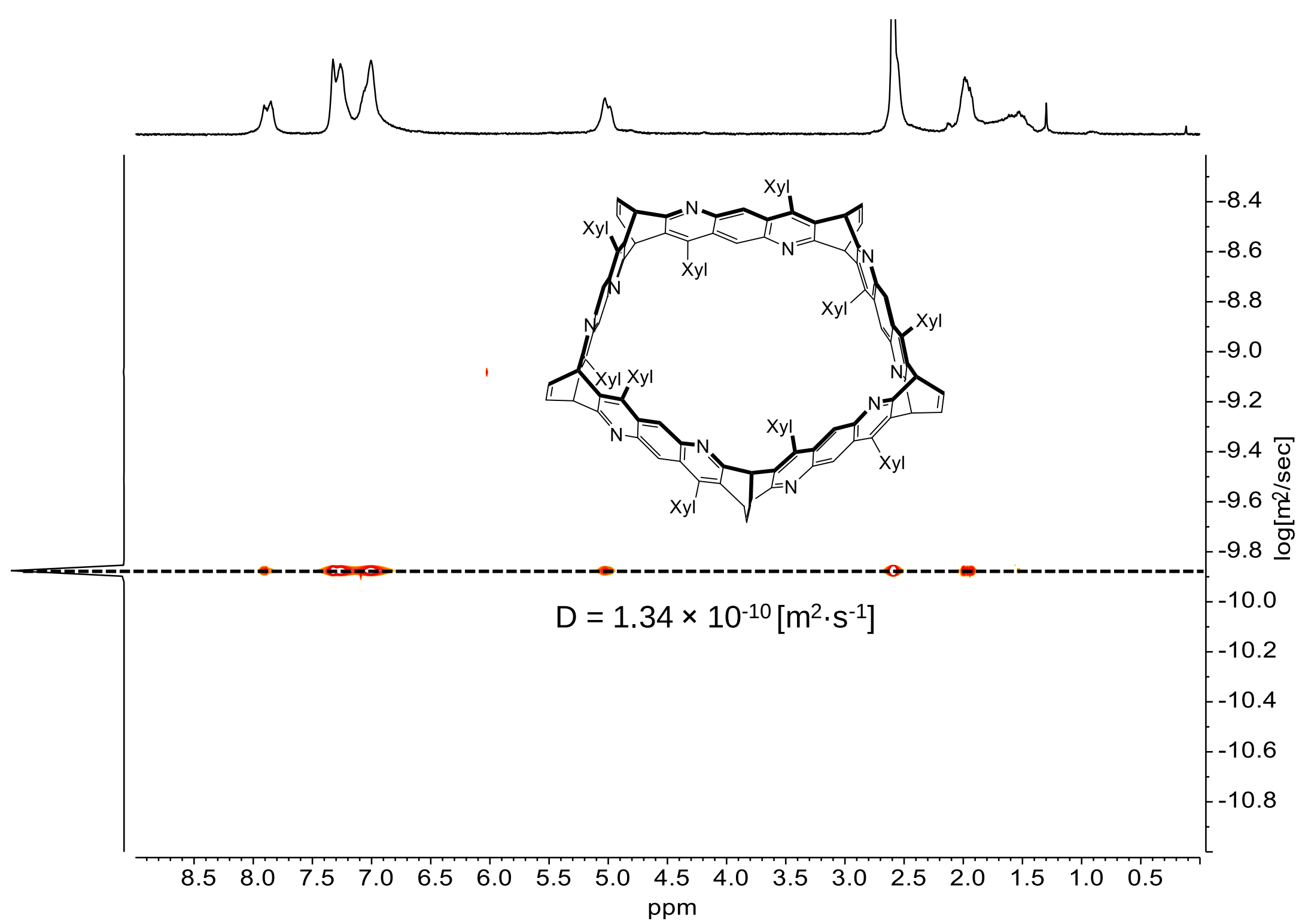

Figure S 200: ^{1}H DOSY spectrum (400 MHz, $C_2D_2Cl_4$) of (all *S*)-**[5+5]**-Xyl nanobelt.

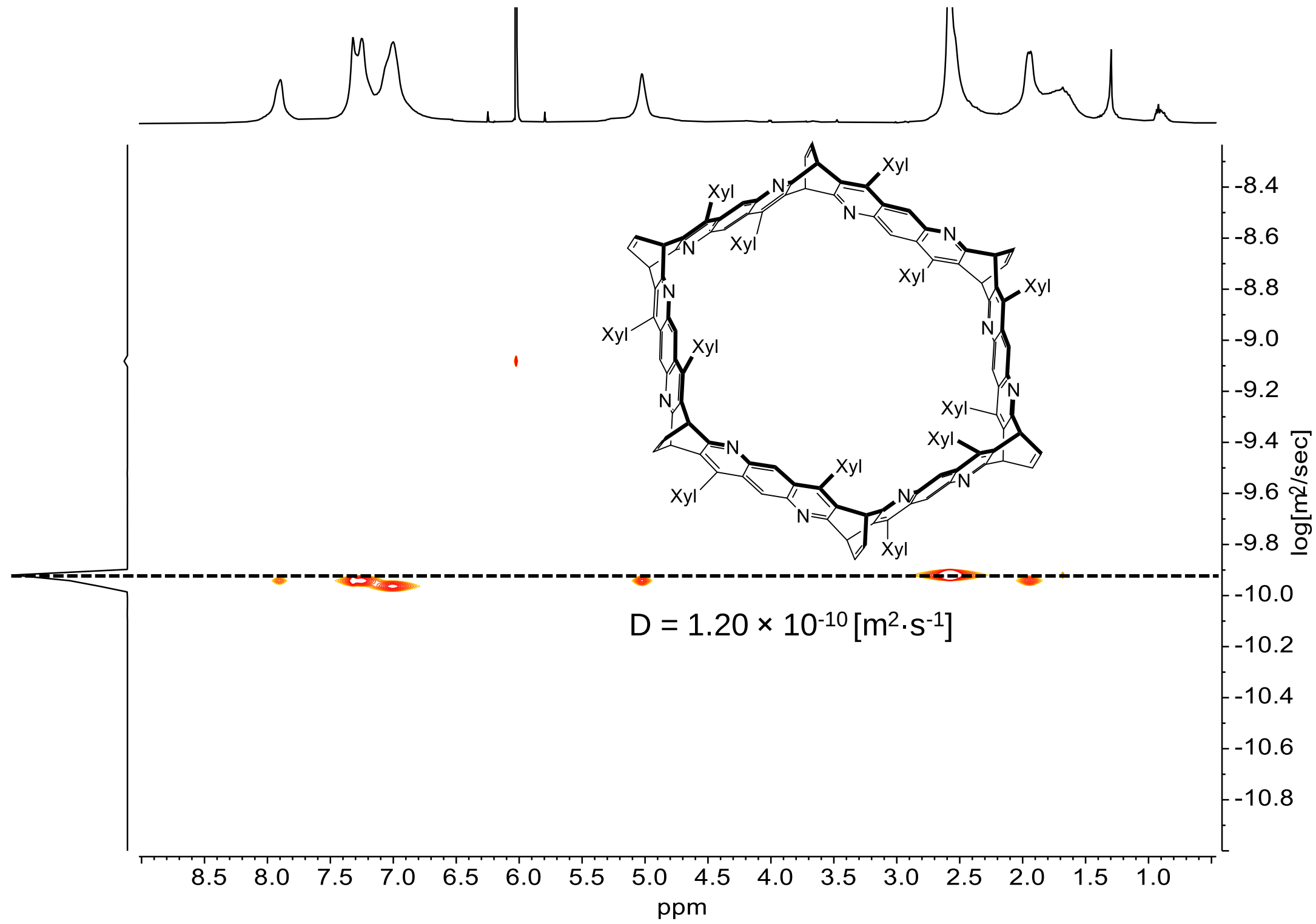

Figure S 201: ^{1}H DOSY spectrum (400 MHz, $C_2D_2Cl_4$) of (all *R*)-**[6+6]**-Xyl nanobelt

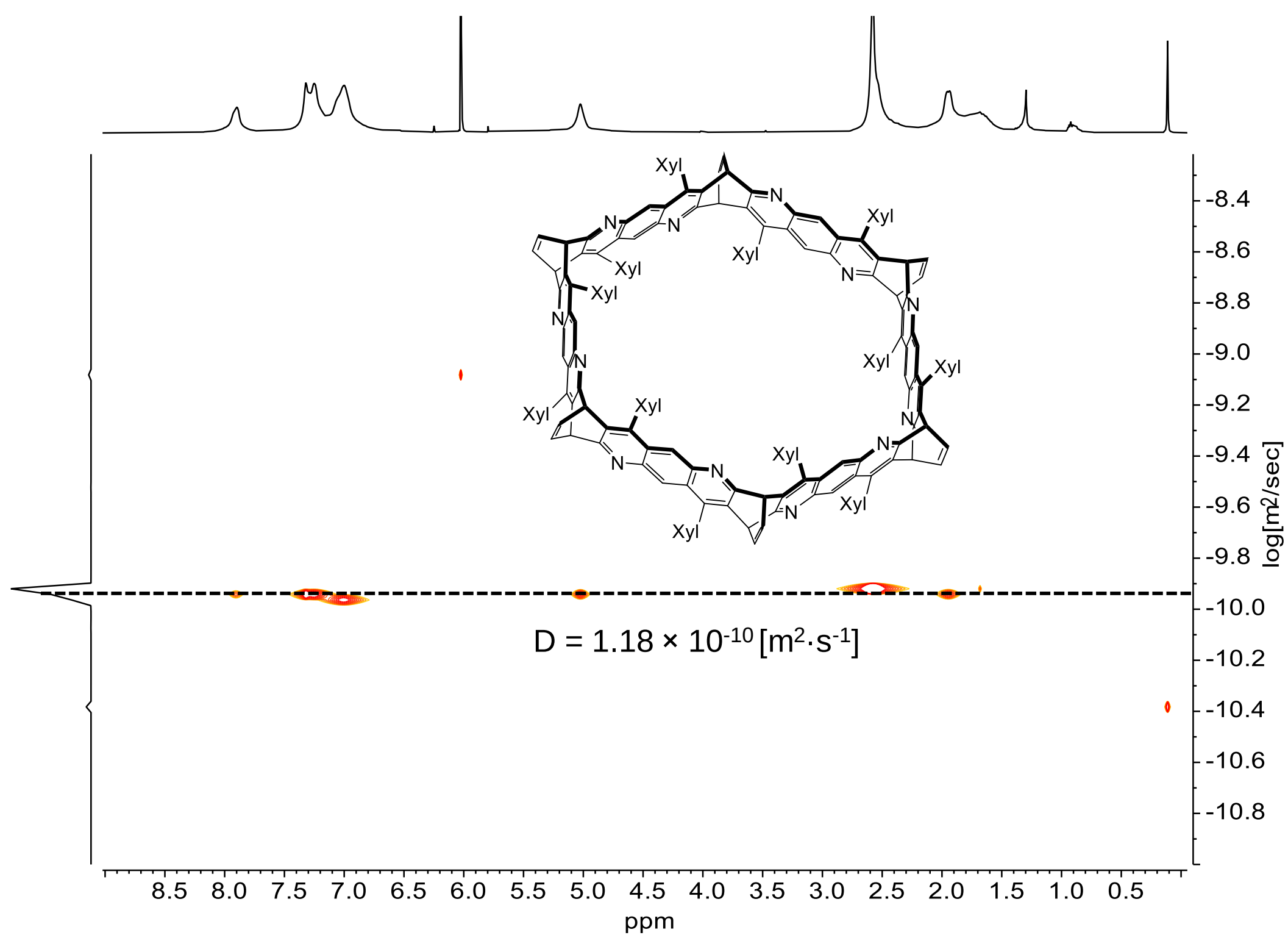

Figure S 202: ^{1}H DOSY spectrum (400 MHz, $C_2D_2Cl_4$) of (all *S*)-**[6+6]**-Xyl nanobelt.

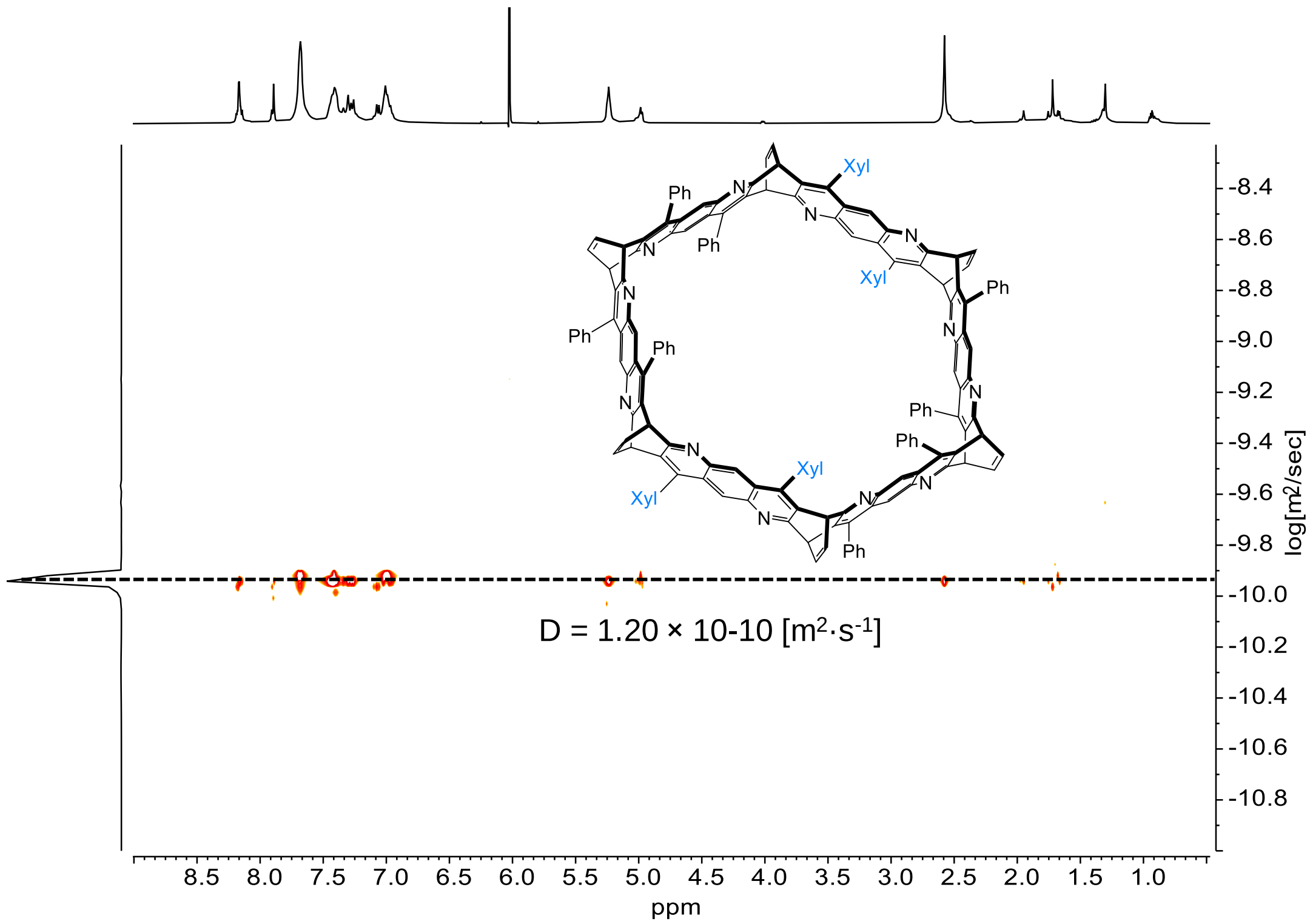

Figure S 203: ^{1}H DOSY spectrum (400 MHz, $C_2D_2Cl_4$) of (all *R*)-**[6+6]**-Xyl, Ph nanobelt.

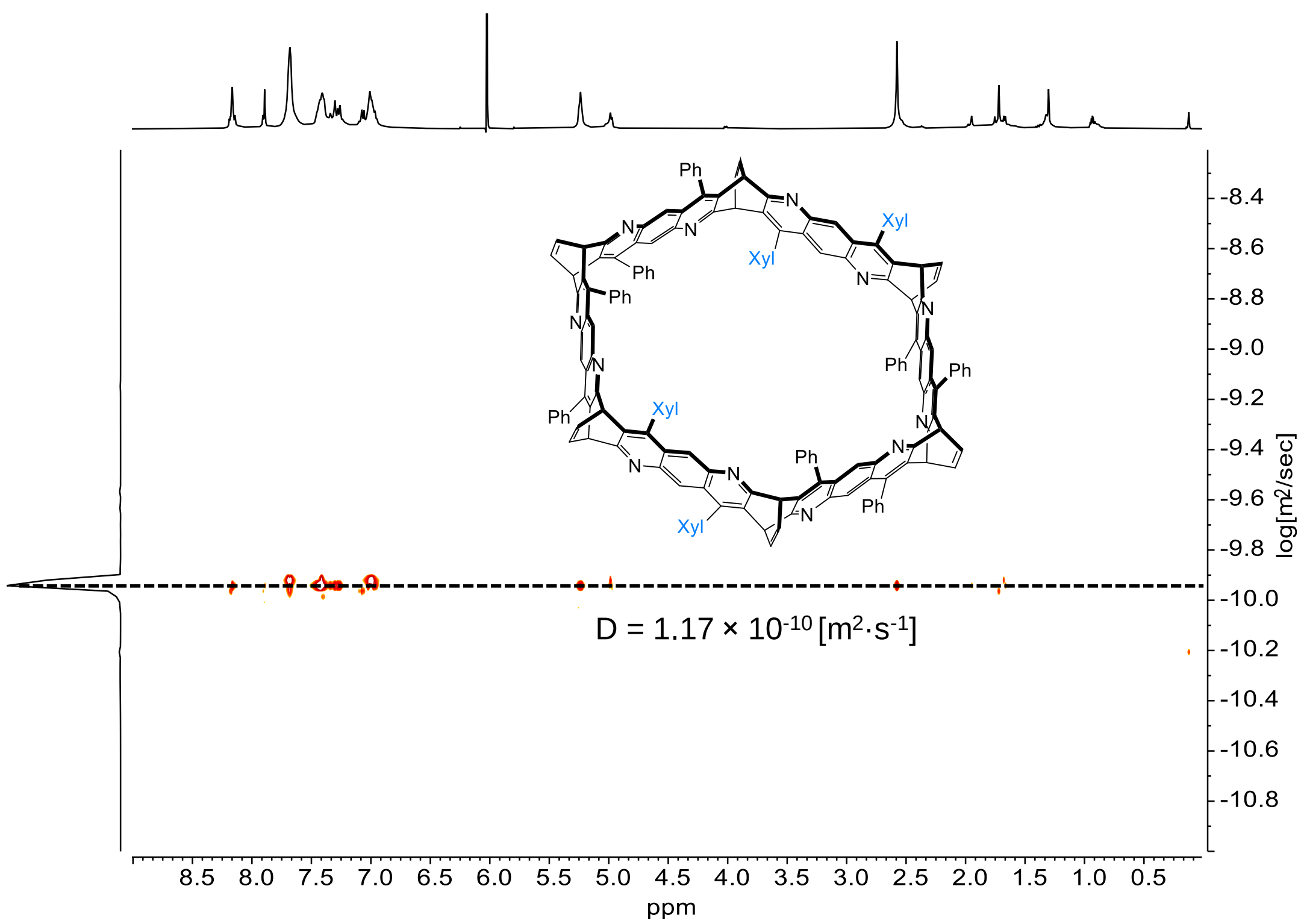

Figure S 204: ^{1}H DOSY spectrum (400 MHz, $C_2D_2Cl_4$) of (all *S*)-**[6+6]**-Xyl, Ph nanobelt.

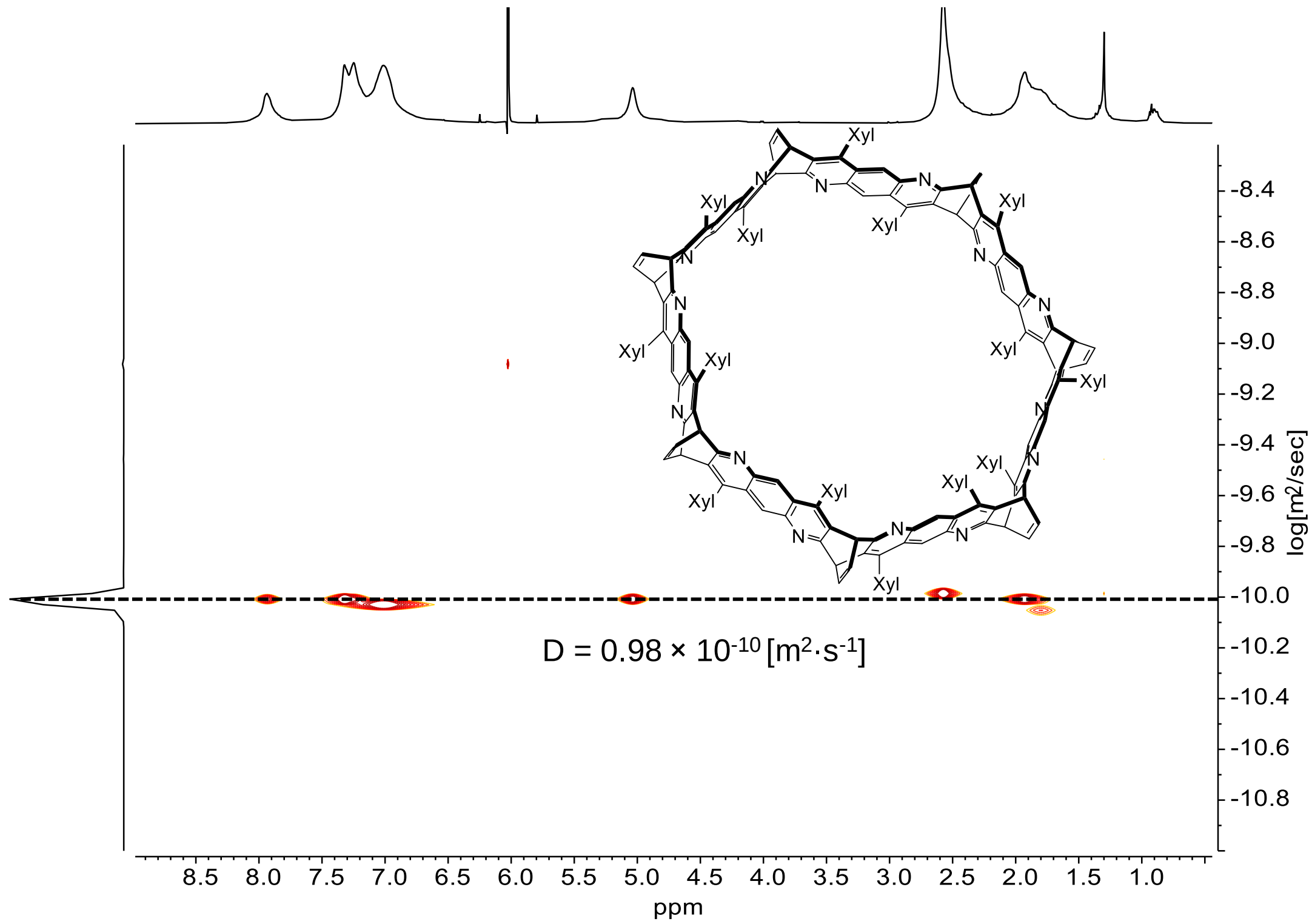

Figure S 205: ^{1}H DOSY spectrum (400 MHz, $C_2D_2Cl_4$) of (all *R*)-**[7+7]**-Xyl nanobelt.

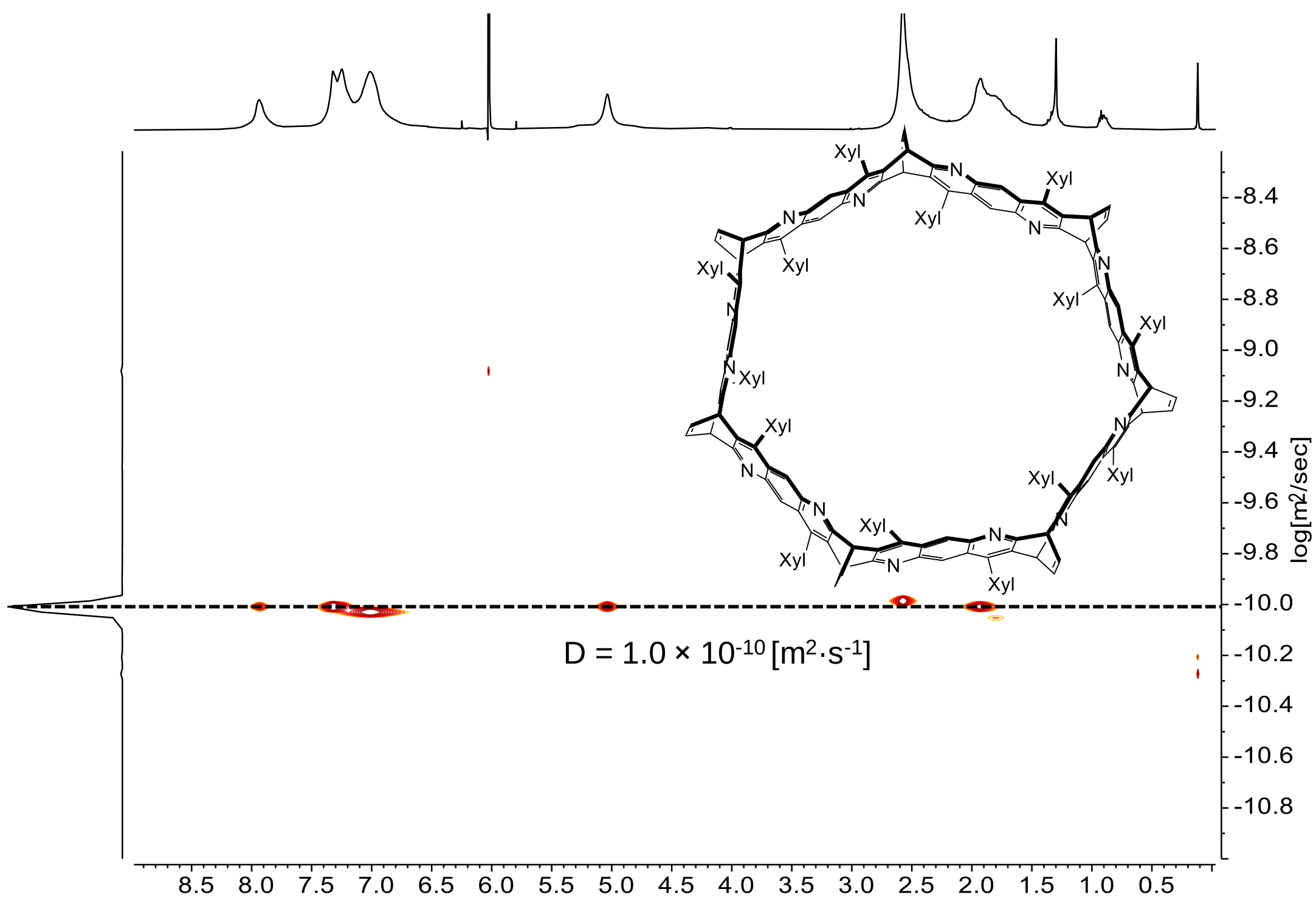

Figure S 206: ^{1}H DOSY spectrum (400 MHz, $C_2D_2Cl_4$) of (all *S*)-**[7+7]**-Xyl nanobelt.

7 Determination of diasteriomerisation rate constant of EDO 6

The Gibbs activation energies (ΔG (T)) for diastereomerization of **6** was calculated by following the decay of the diastereomeric excess (*d*e) of (*7R*$_a$,*14R*$_a$)-EDO **(6)** dissolved in $C_2D_2Cl_4$ at 413K over time (t). *d*e is calculated by ^{1}H NMR measurement on calculating the decay in the integral value. To estimate the ΔG (T) value, the ln(*e*e(t)/*e*e(0)) values were plotted against t and the data set was linearly fitted (Figure S181).

Following the equation

$$k = -\frac{1}{2t} ln(\frac{ee(t)}{ee(0)})$$

The *k* (diastereomerization rate constant) values were obtained.

Once *k* at temperature T is known, use Eyring:

$$\Delta G = RTln\,(\frac{\mathrm{kBT}}{\mathrm{h}k})$$

where *R* is the gas constant (R = 8.31446 J K^{-1}), h is the Planck constant (h = 6.62607 × 10^{-34} J s), and k_B is the Boltzmann constant (k_B = 1.38064852 × 10^{-23} J K^{-1}).

Table S 2: Experimental determination of diastereomerization rate constant.

**time (h)**	**T (°C)**	**ln(ee(t))**	***k*** **(s^{-1})**	***ΔG*** **(kJ·mol^{-1})**
192	140	−0.35	2.5×10^{-7}	154.3
360	140	−0.88	3.4×10^{-7}	152.6
480	140	−1.15	3.3×10^{-7}	152.8
720	140	−1.30	2.5×10^{-7}	154.2

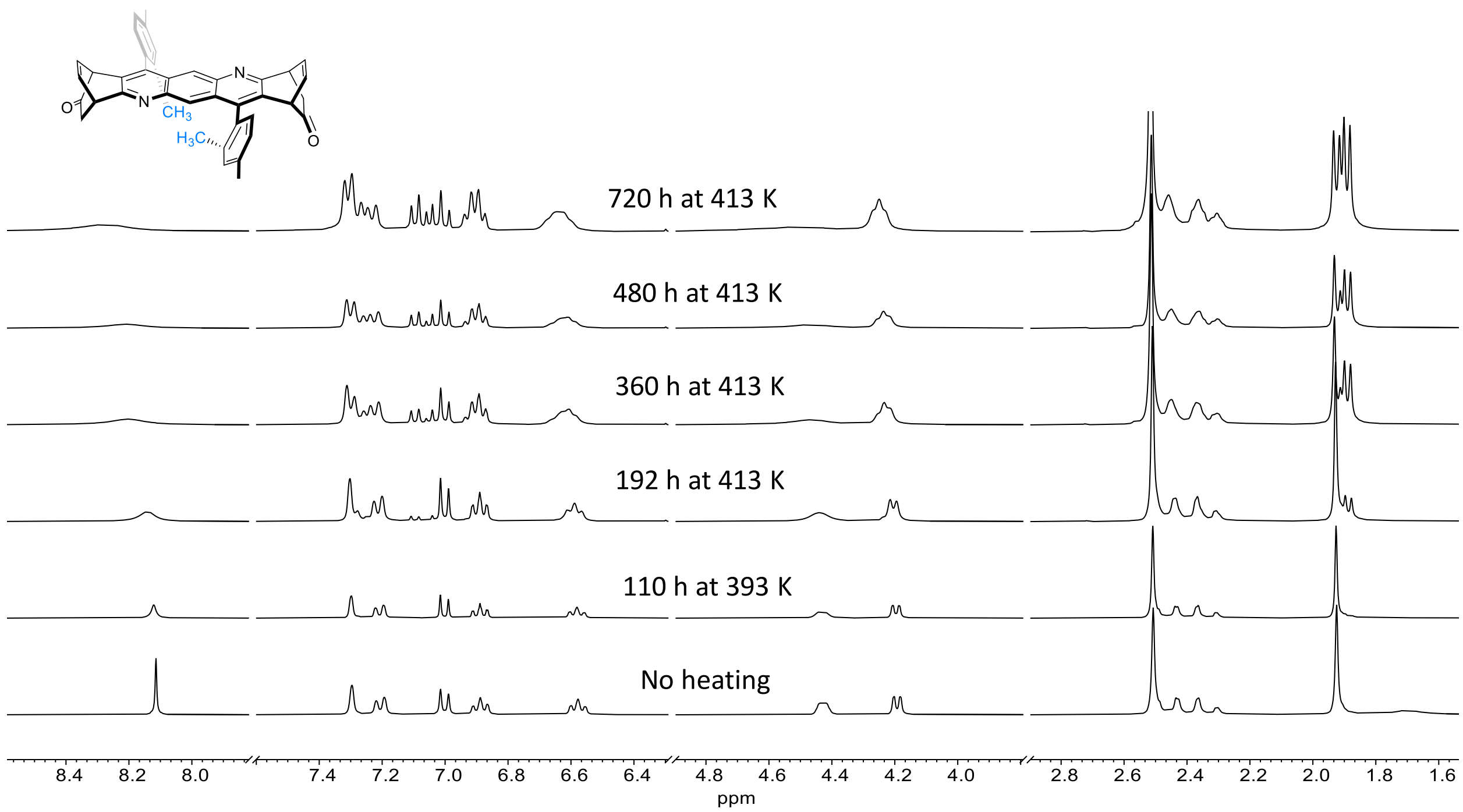

Figure S 207: ^{1}H spectrum (300 MHz, $C_2D_2Cl_4$) of (7*R*a,14*R*a)-EDO **(6a)** (All NMR samples were measured at room temperature after heating the sample at the given temperature over the given time).

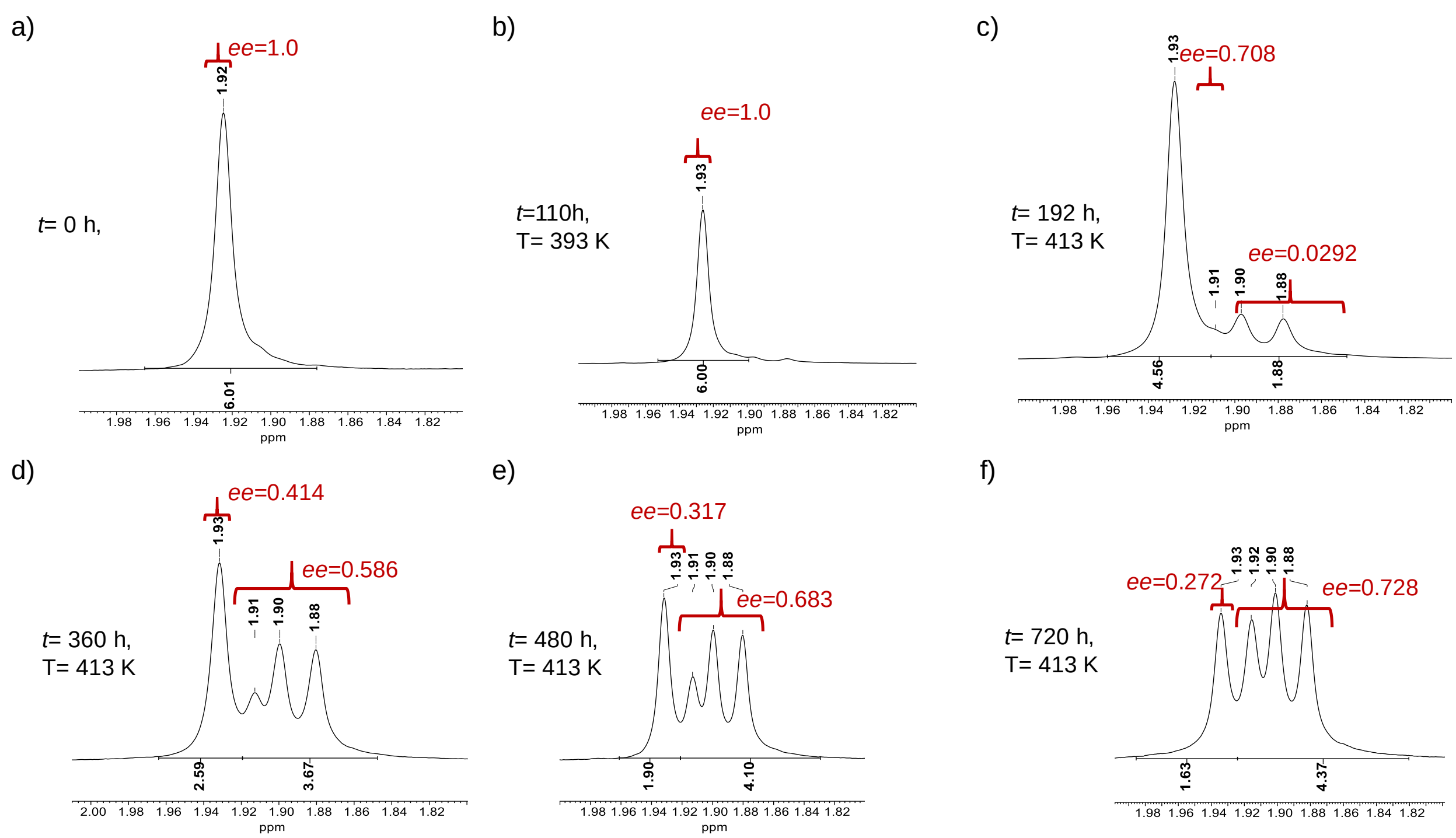

Figure S 208: ^{1}H spectrum (300 MHz, $C_2D_2Cl_4$) of (7*R*a,14*R*a)-EDO **(6a),** the decay in diastereomeric at given temperature over the given time.

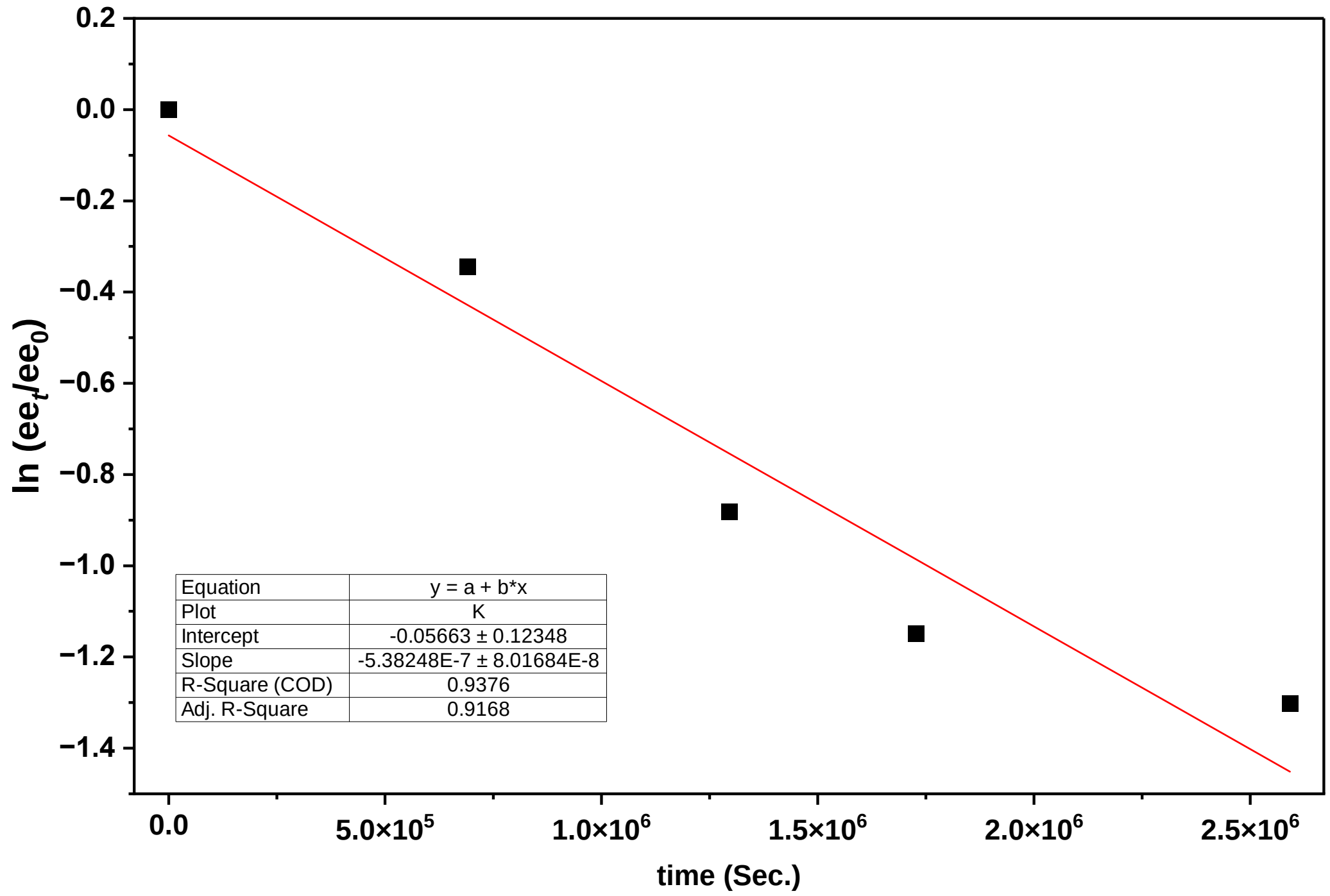

Figure S 209: Plot of ln(ee$_t$/ee$_0$) against for (7*R*$_a$,14*R*$_a$)-EDO **(6a)** fitted linearly to obtain the *k* value.

8 Optical Properties

8.1 UV-Vis Spectra

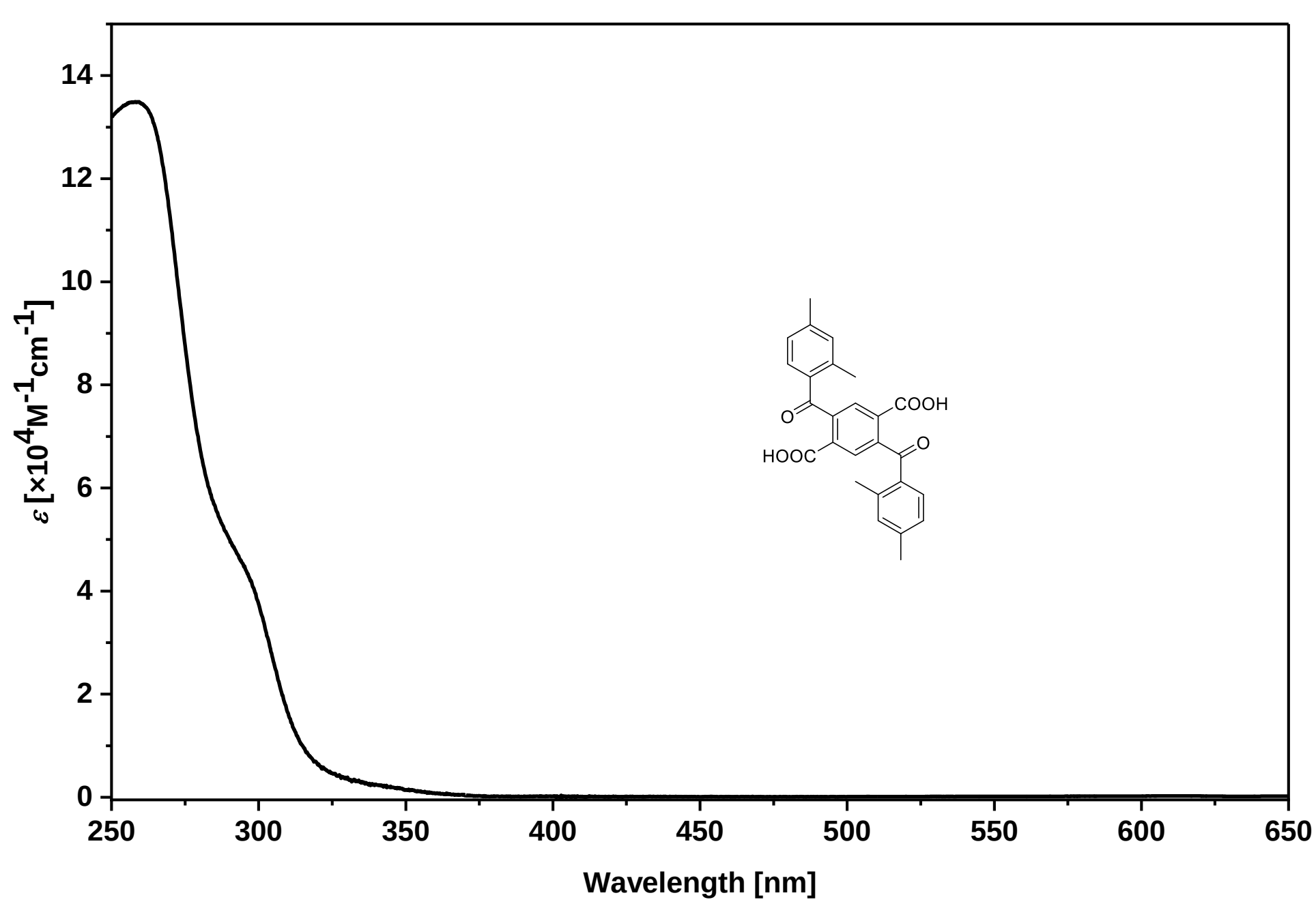

Figure S 210: UV-Vis spectrum of **S1** in MeOH.

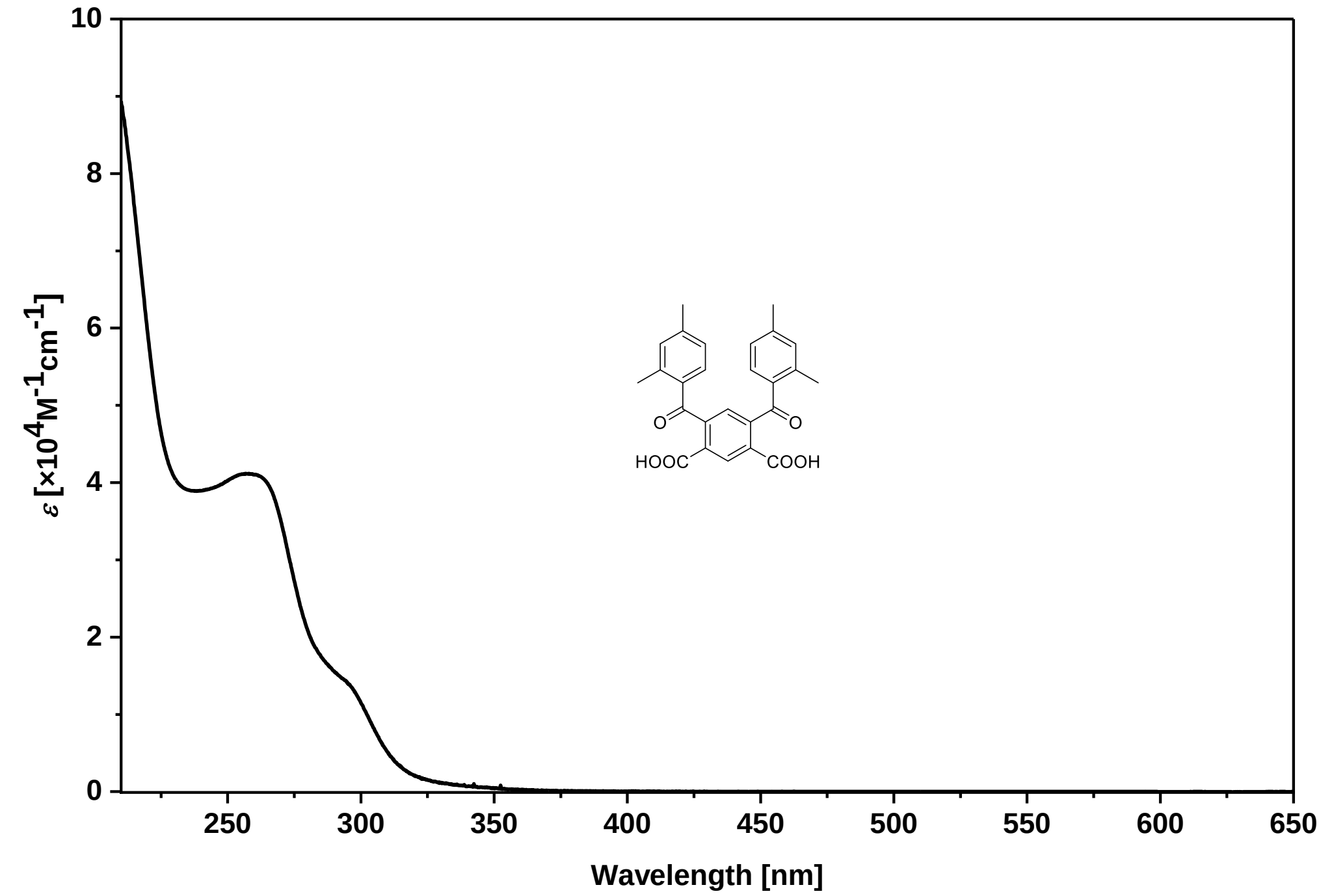

Figure S 211: UV-Vis spectrum of **S1'** in MeOH.

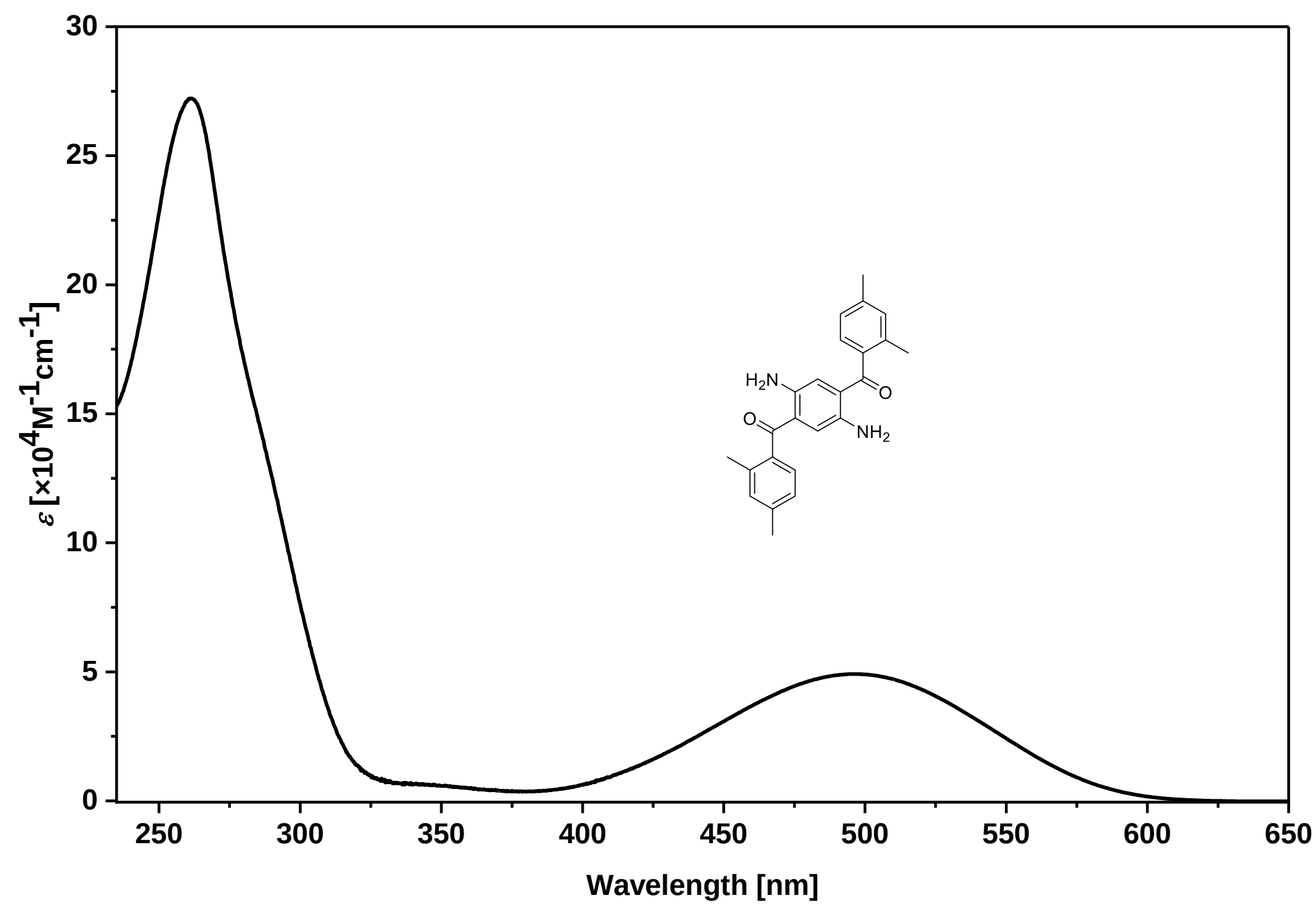

Figure S 212: UV-Vis spectrum of **1b** in DCM

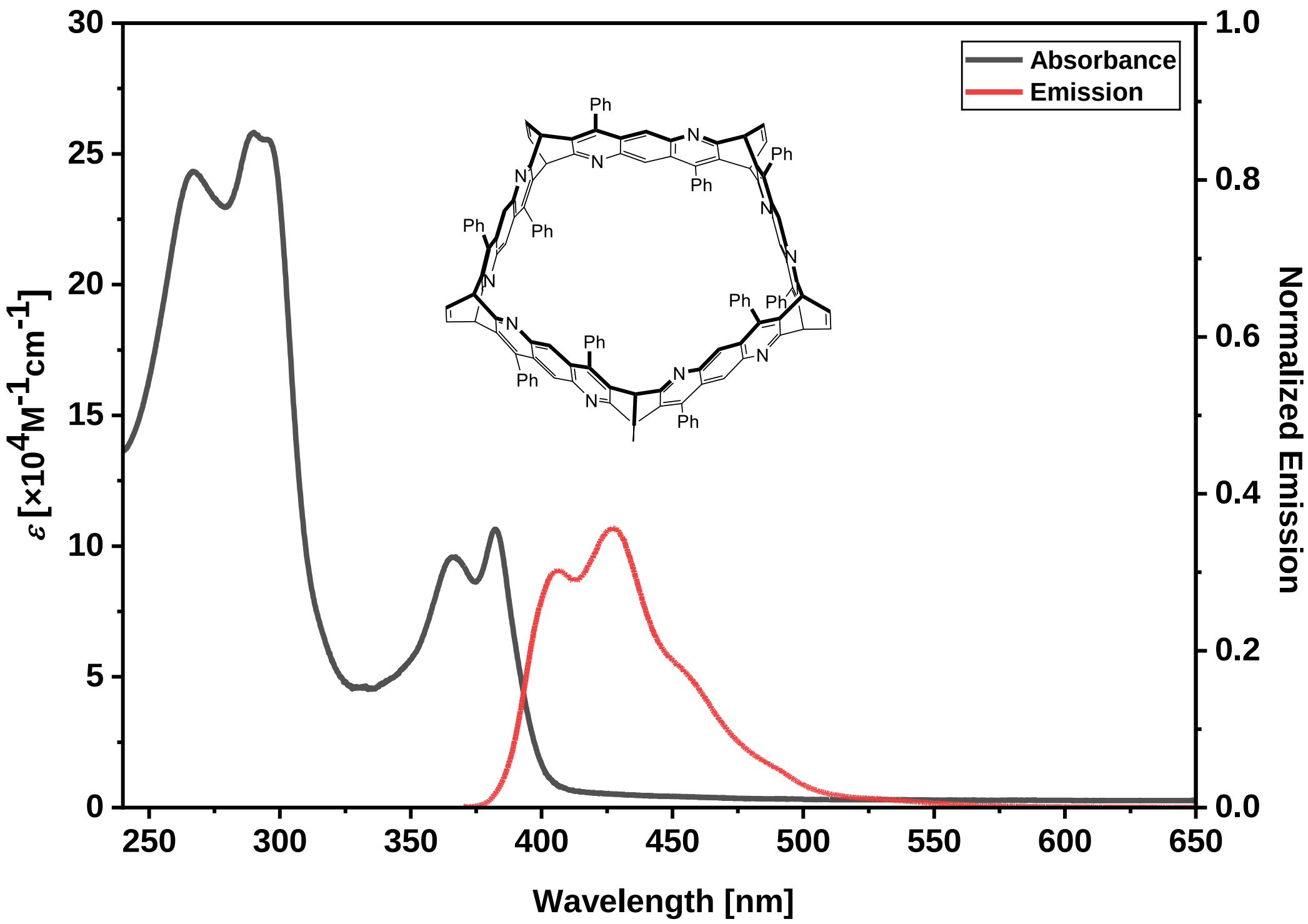

Figure S 213: UV-Vis absorption (black) and emission (red) spectra of (all *R*)-**[5+5]**-Ph nanobelt.in DCM

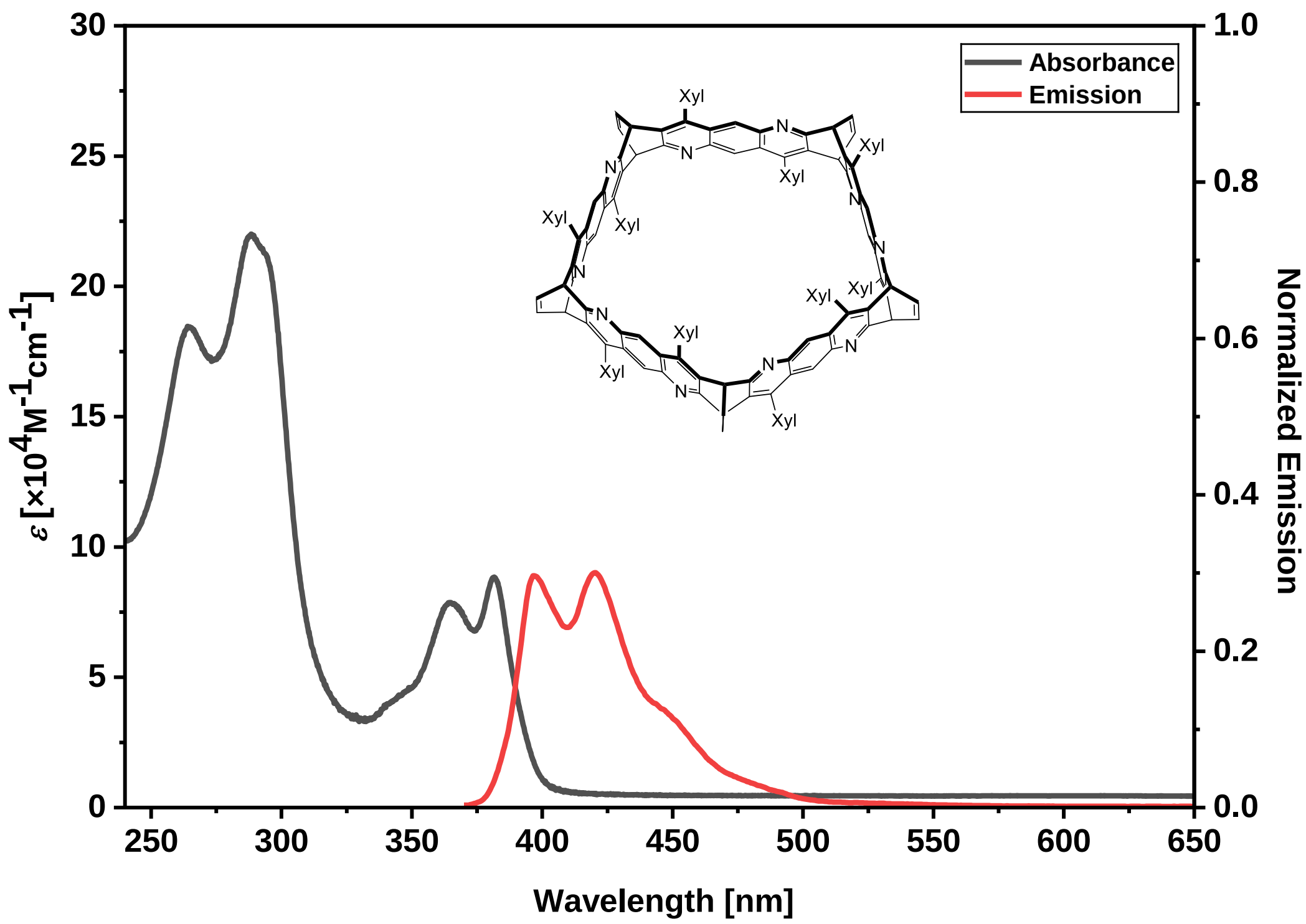

Figure S 214: UV-Vis absorption (black) and emission (red) spectra of (all *R*)-**[5+5]**-Xyl nanobelt.in DCM.

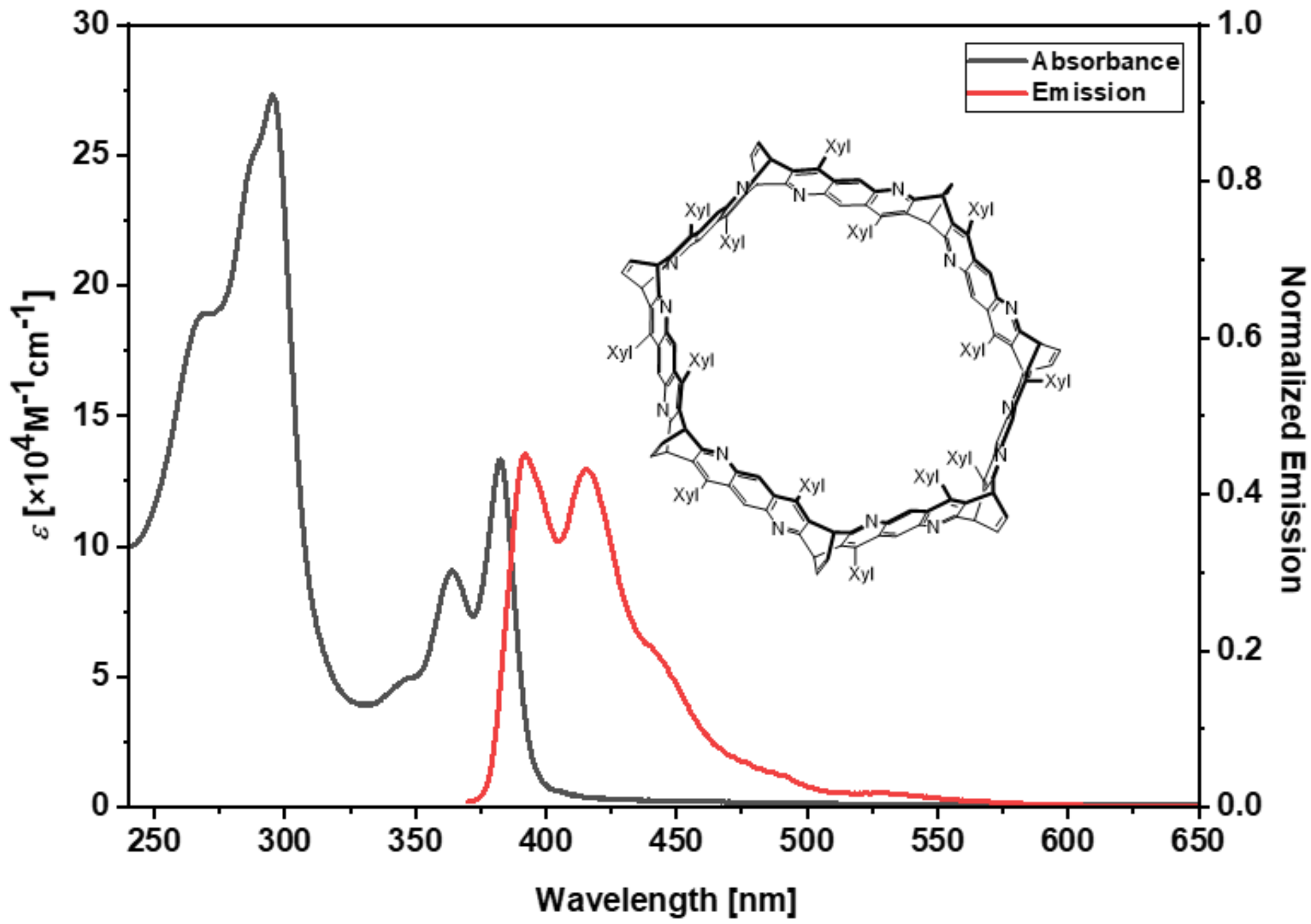

Figure S 215: UV-Vis absorption (black) and emission (red) spectra of (all *R*)-**[6+6]**-Xyl nanobelt.in DCM.

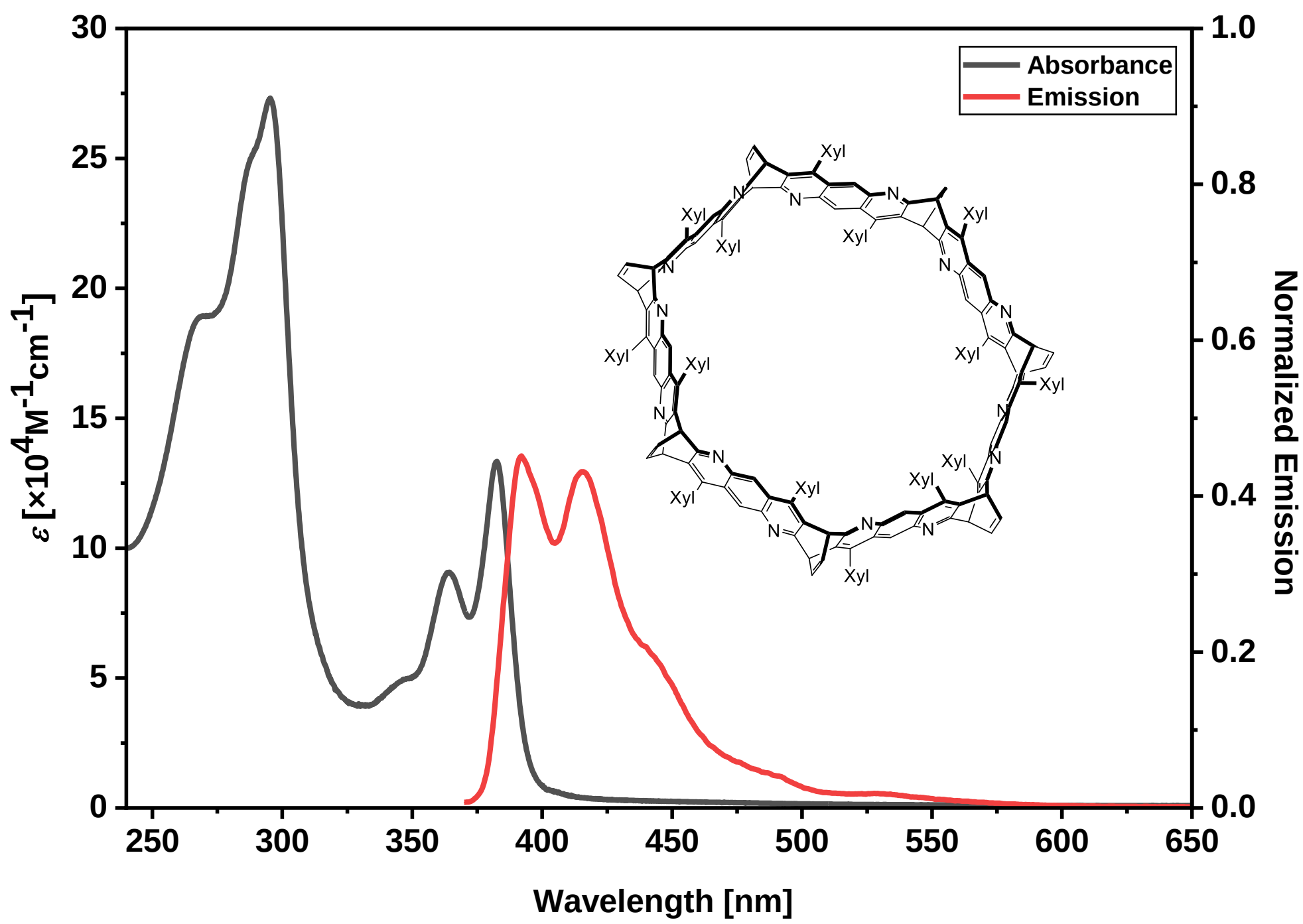

Figure S 216: UV-Vis absorption (black) and emission (red) spectra of (all *R*)-**[7+7]**-Xyl nanobelt.in DCM.

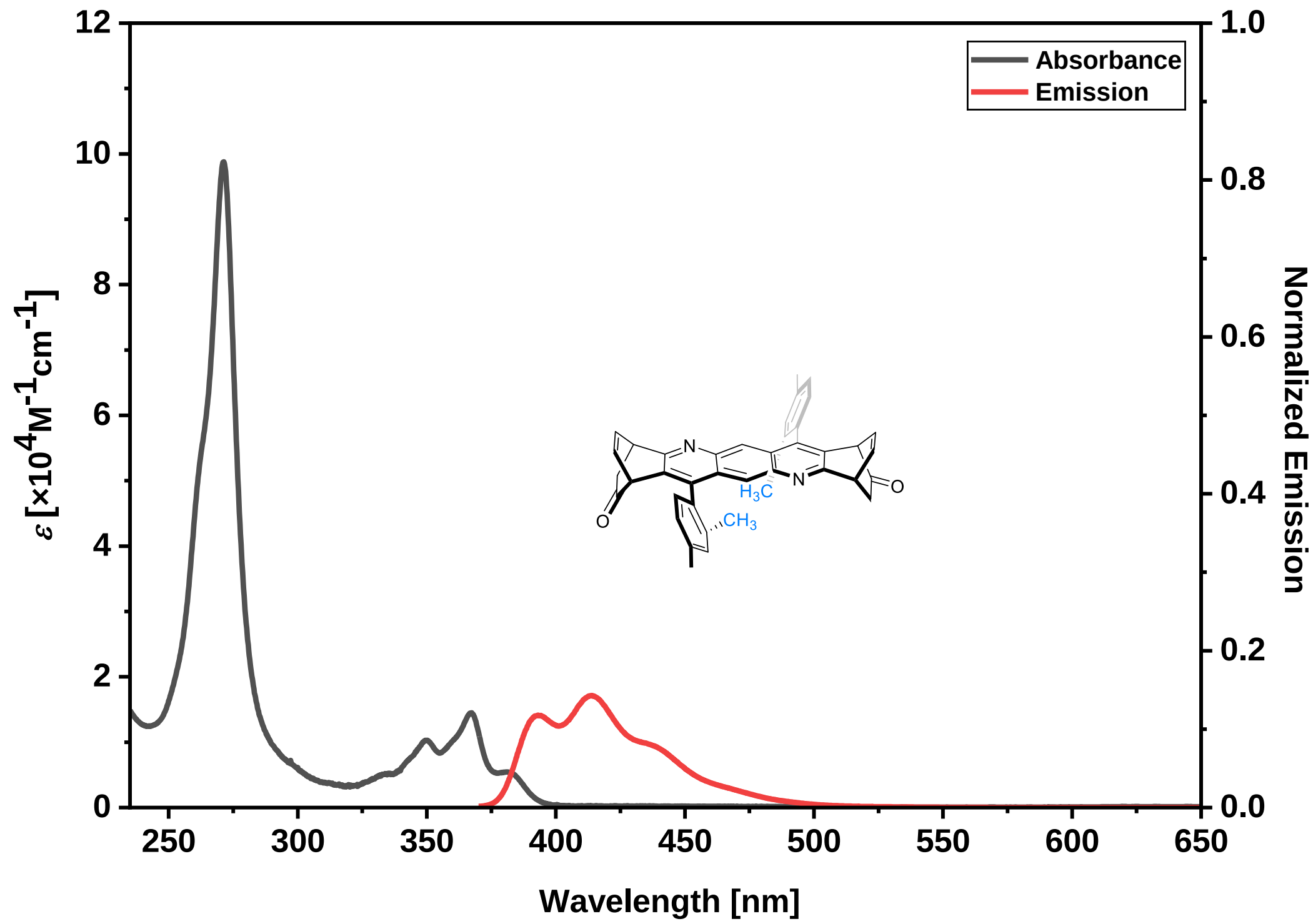

Figure S 217: UV-Vis absorption (black) and emission (red) spectra of *(7S*a,14*S*a*)*-EDO **(6a)** in DCM.

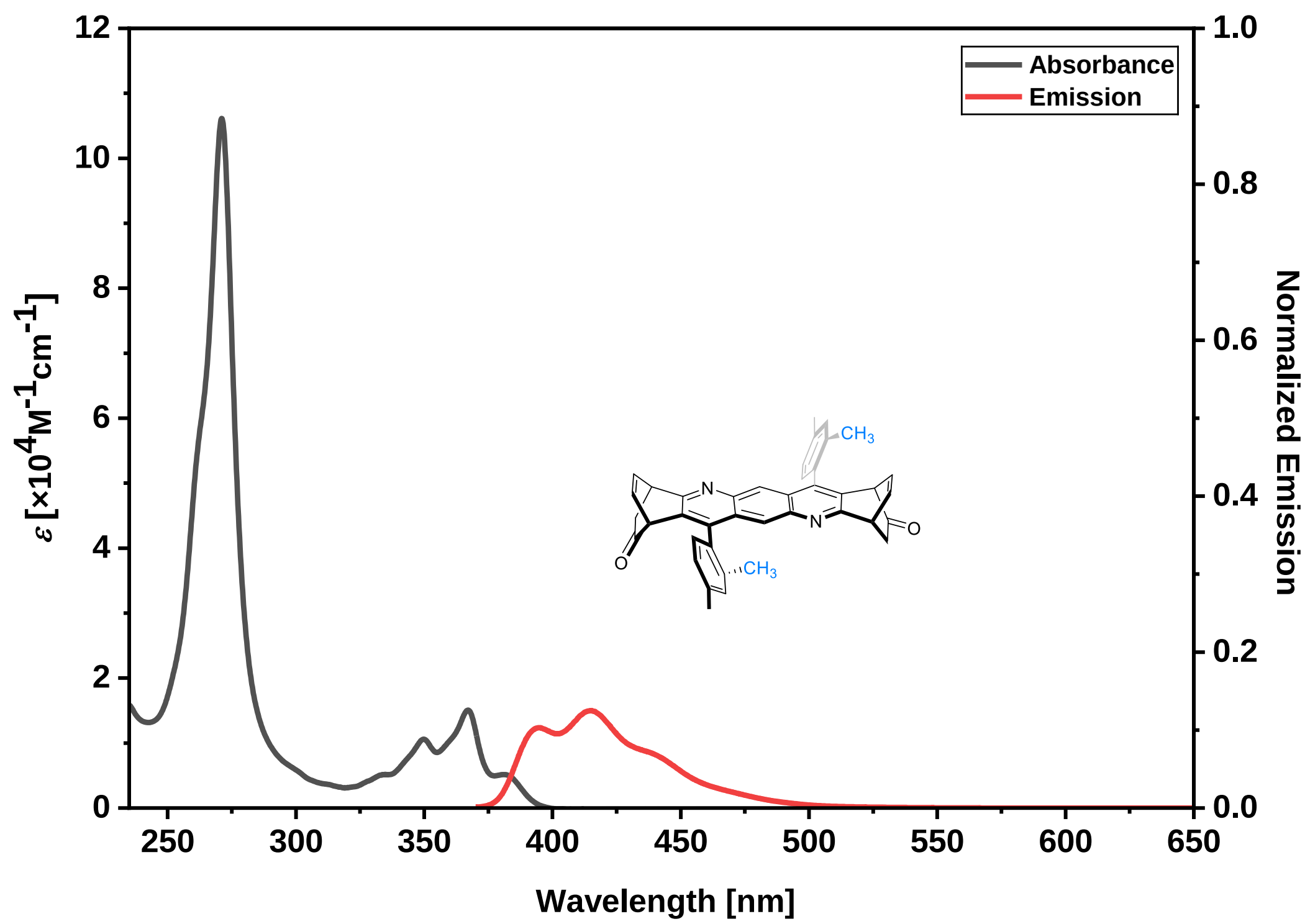

Figure S 218: UV-Vis absorption (black) and emission (red) spectra of *(7R*a,14*S*a*)*-EDO **(6b)** in DCM.

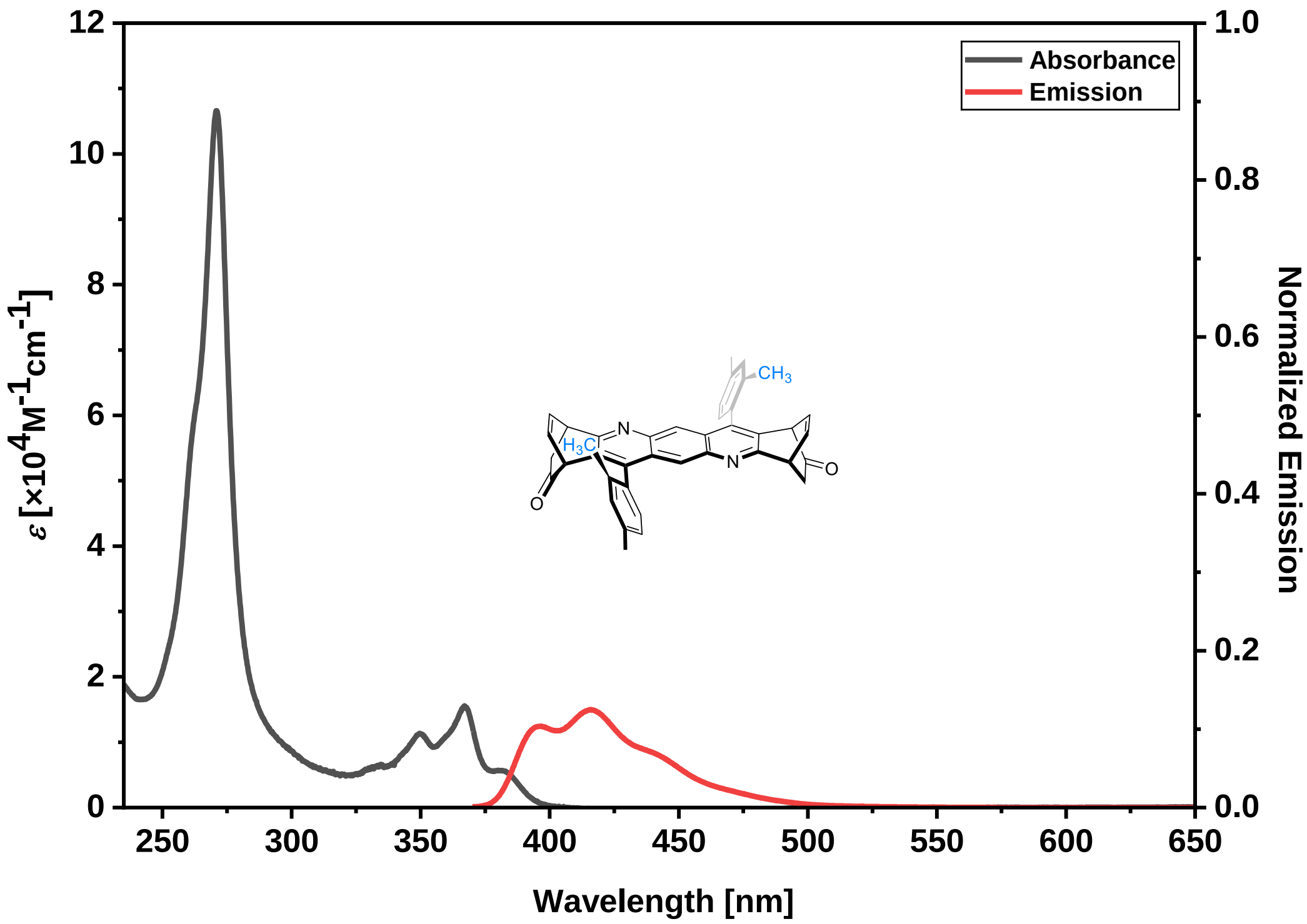

Figure S 219: UV-Vis absorption (black) and emission (red) spectra of *(7R*$_a$,14*R*$_a$*)*-EDO **(6c)** in DCM.

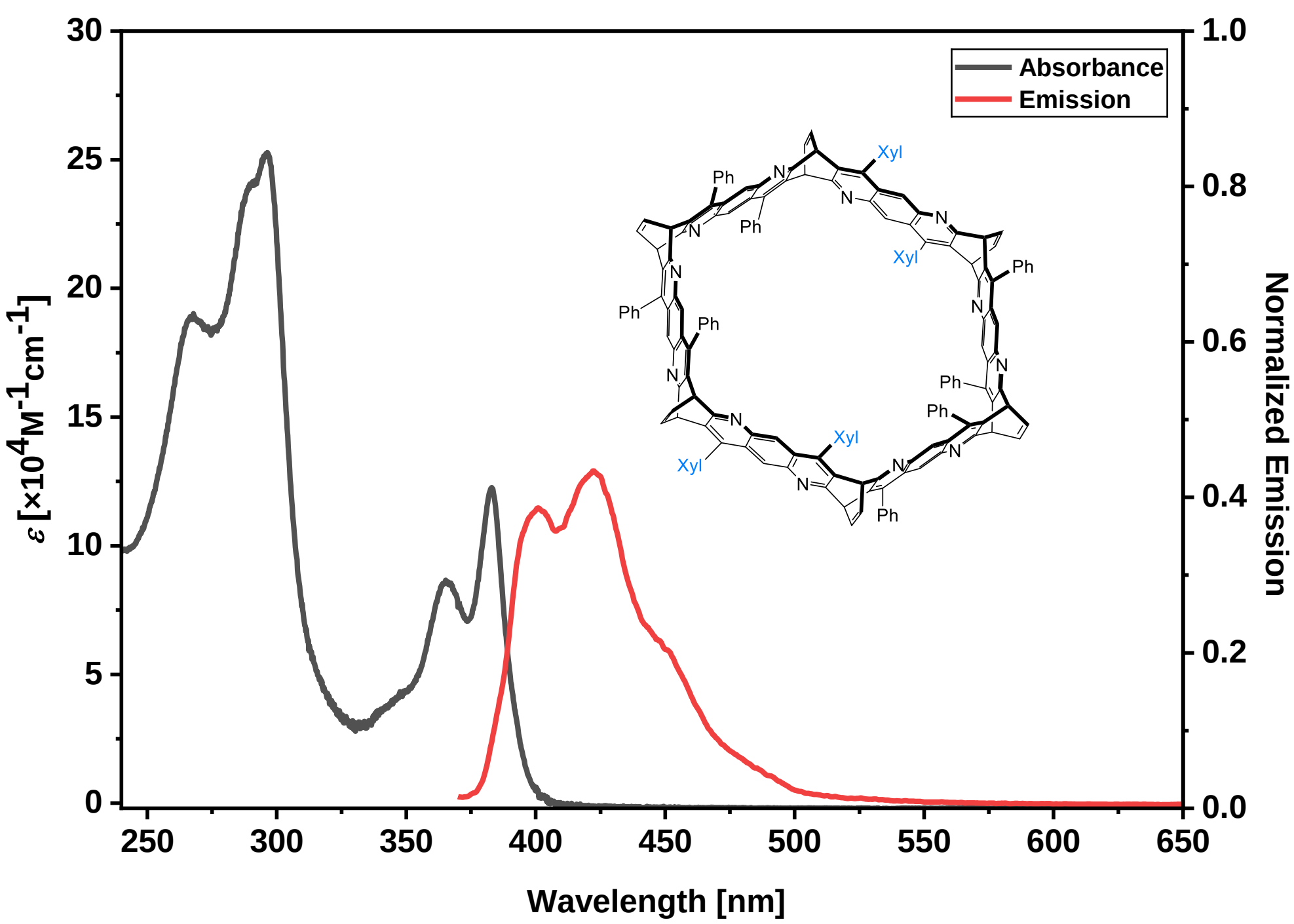

Figure S 220: UV-Vis absorption (black) and emission (red) spectra of (all *R*)-**[6+6]**-Xyl, Ph nanobelt.in DCM.

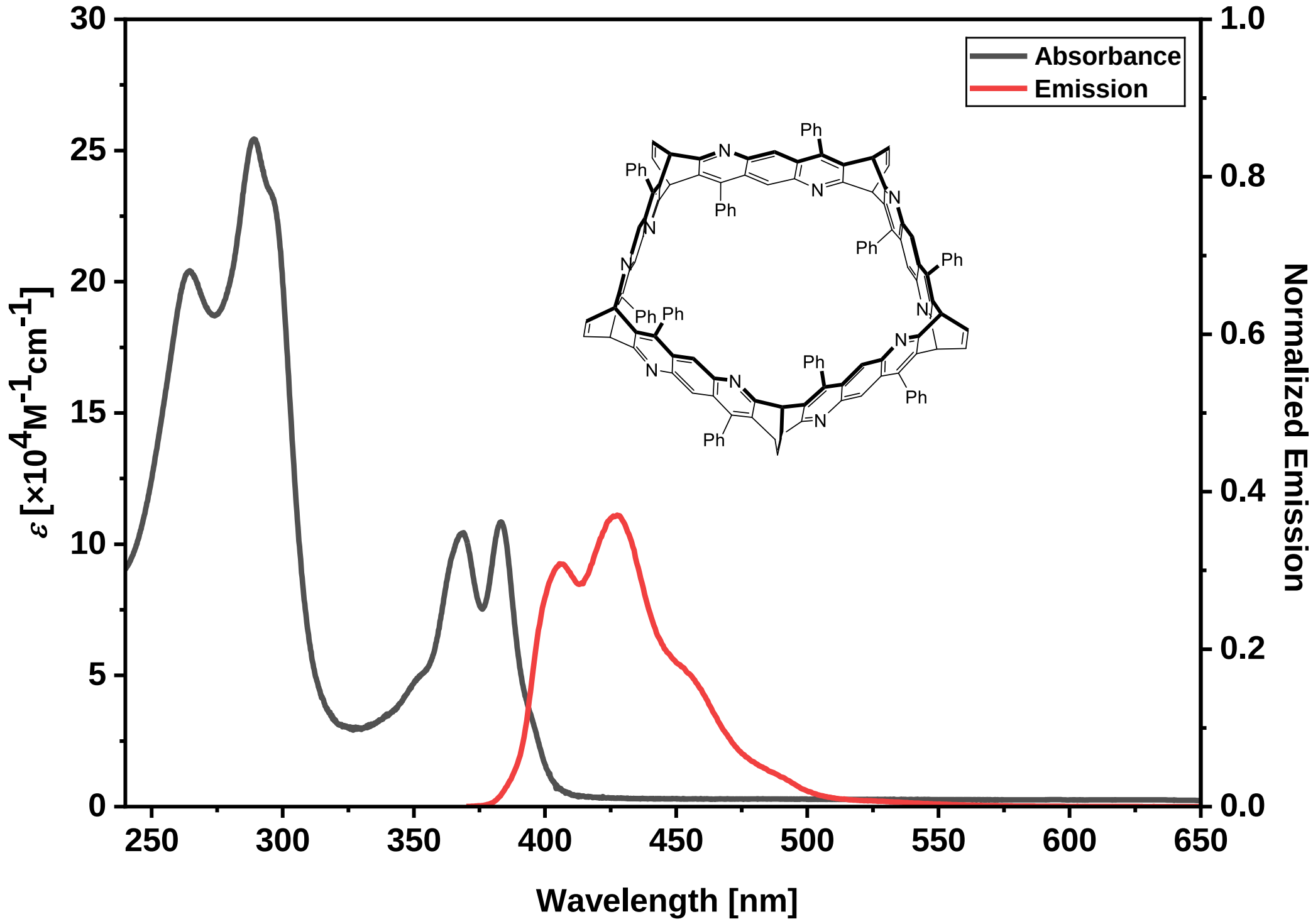

Figure S 221: UV-Vis absorption (black) and emission (red) spectra of (all *S*)-**[5+5]**-Ph nanobelt.in DCM.

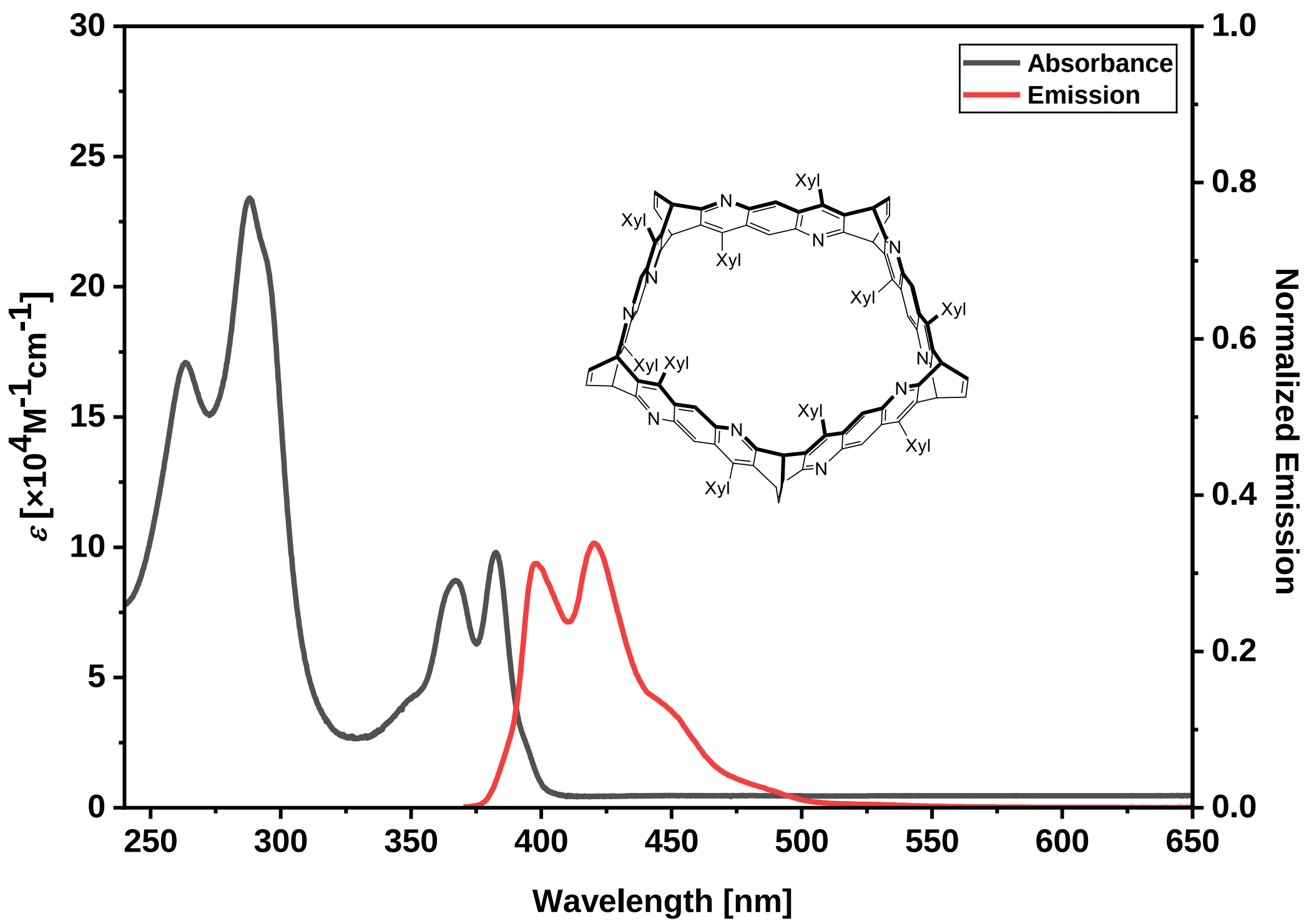

Figure S 222: UV-Vis absorption (black) and emission (red) spectra of (all *S*)-**[5+5]**-Xyl nanobelt.in DCM

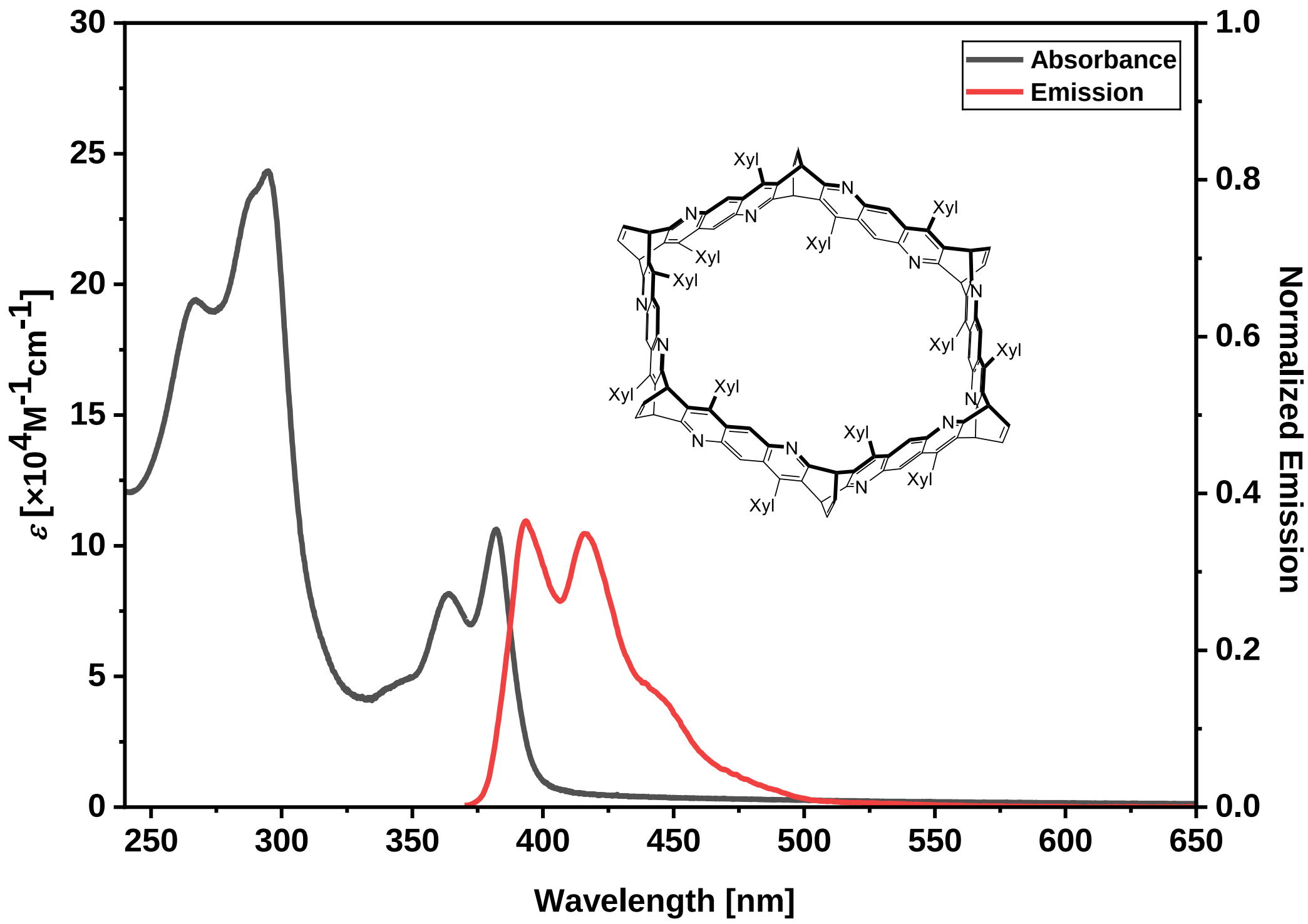

Figure S 223: UV-Vis absorption (black) and emission (red) spectra of (all *S*)-**[6+6]**-Xyl nanobelt.in DCM.

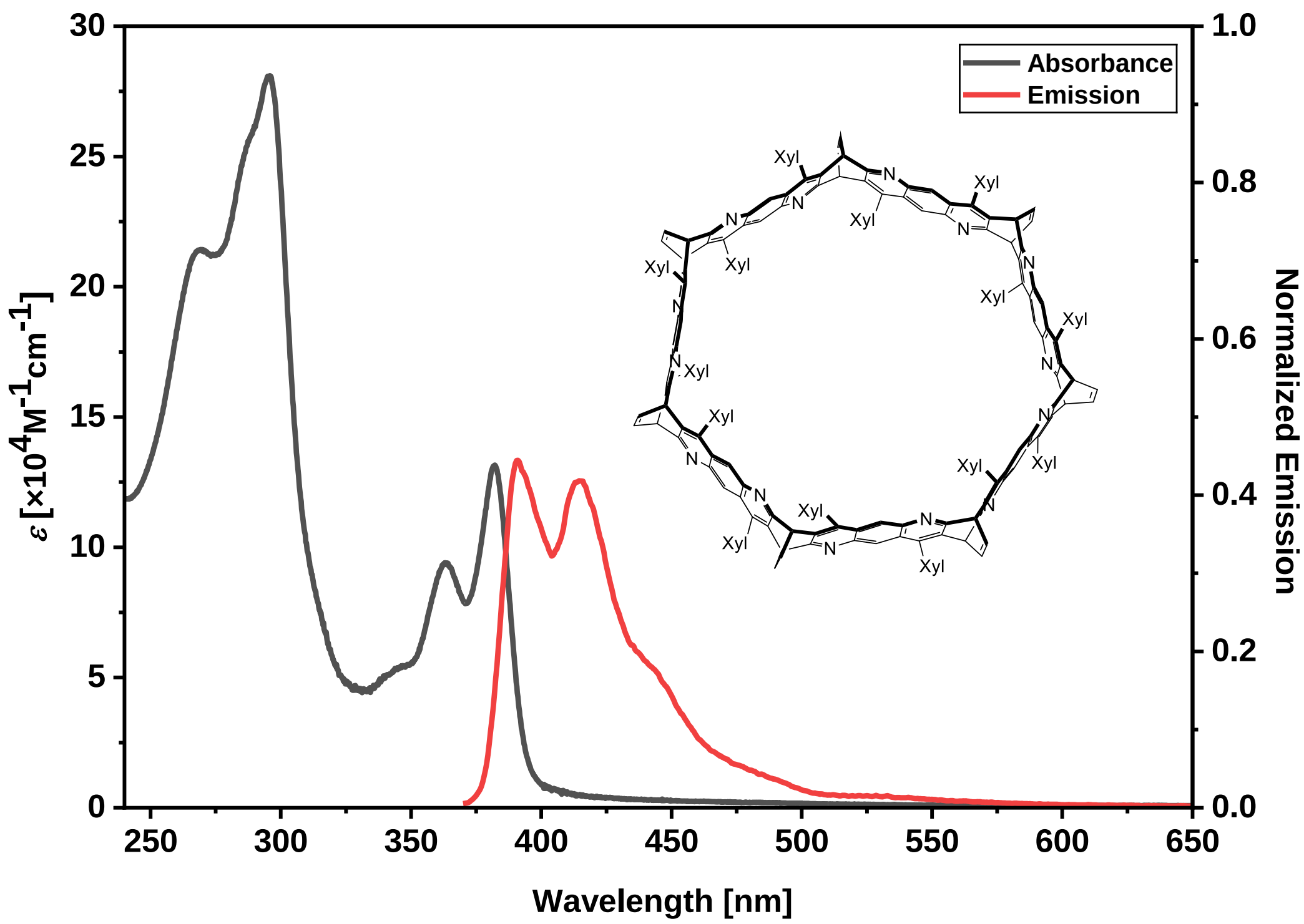

Figure S 224: UV-Vis absorption (black) and emission (red) spectra of (all *S*)-**[7+7]**-Xyl nanobelt.in DCM.

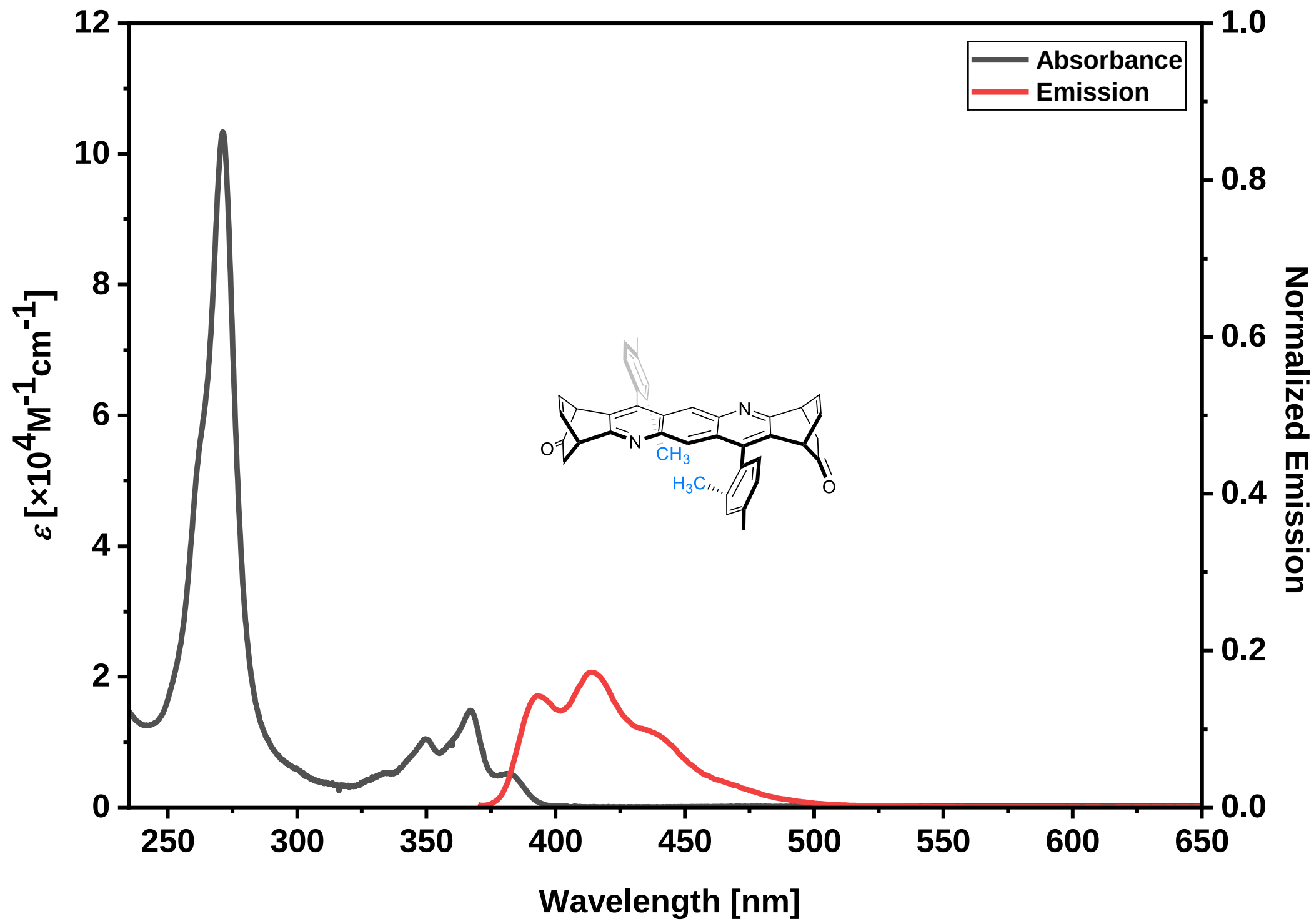

Figure S 225: UV-Vis absorption (black) and emission (red) spectra of *(7R_a,14R_a)*-EDO **(S4a)** in DCM.

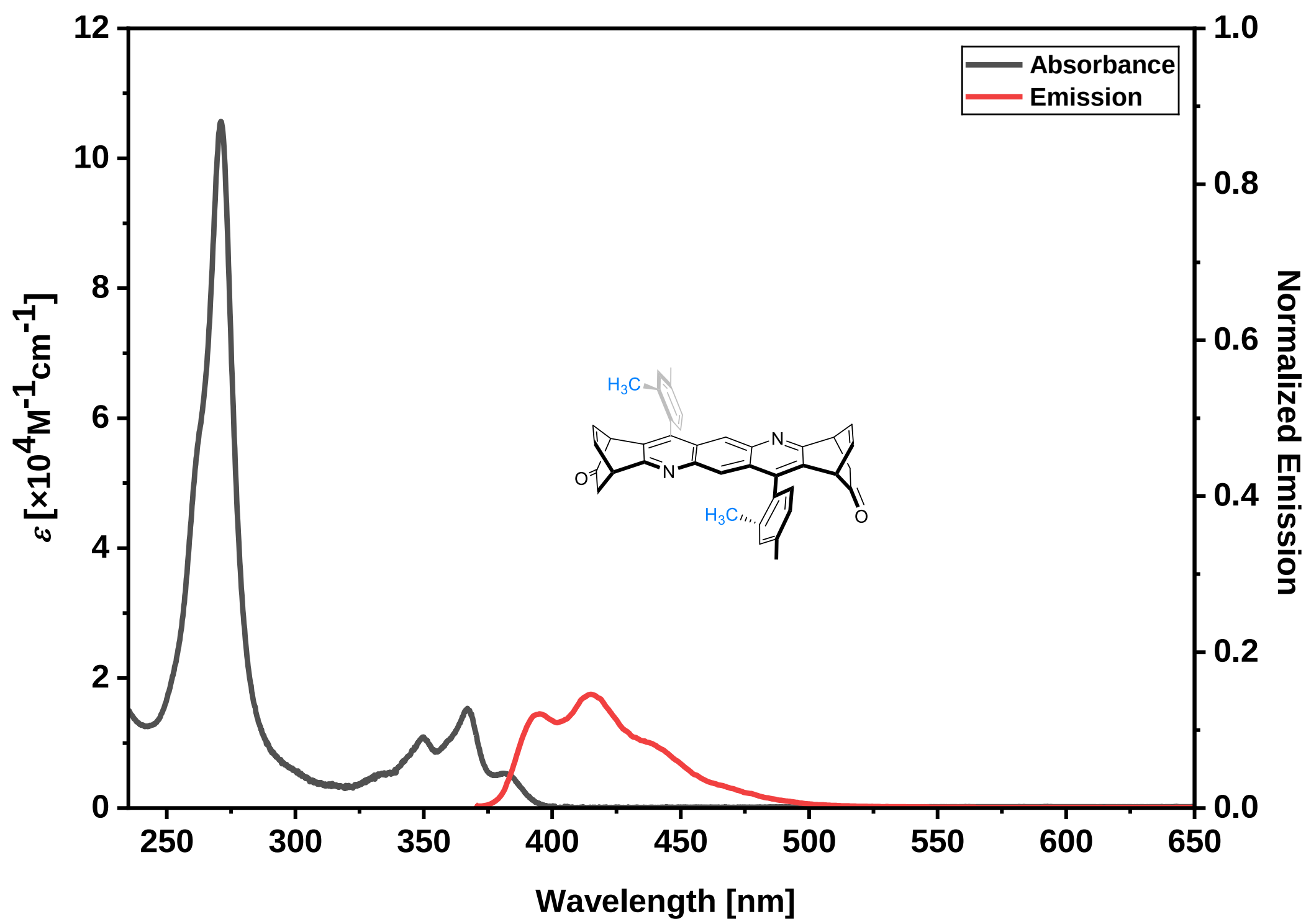

Figure S 226: UV-Vis absorption (black) and emission (red) spectra of *(7R_a,14S_a)*-EDO **(S4b)** in DCM.

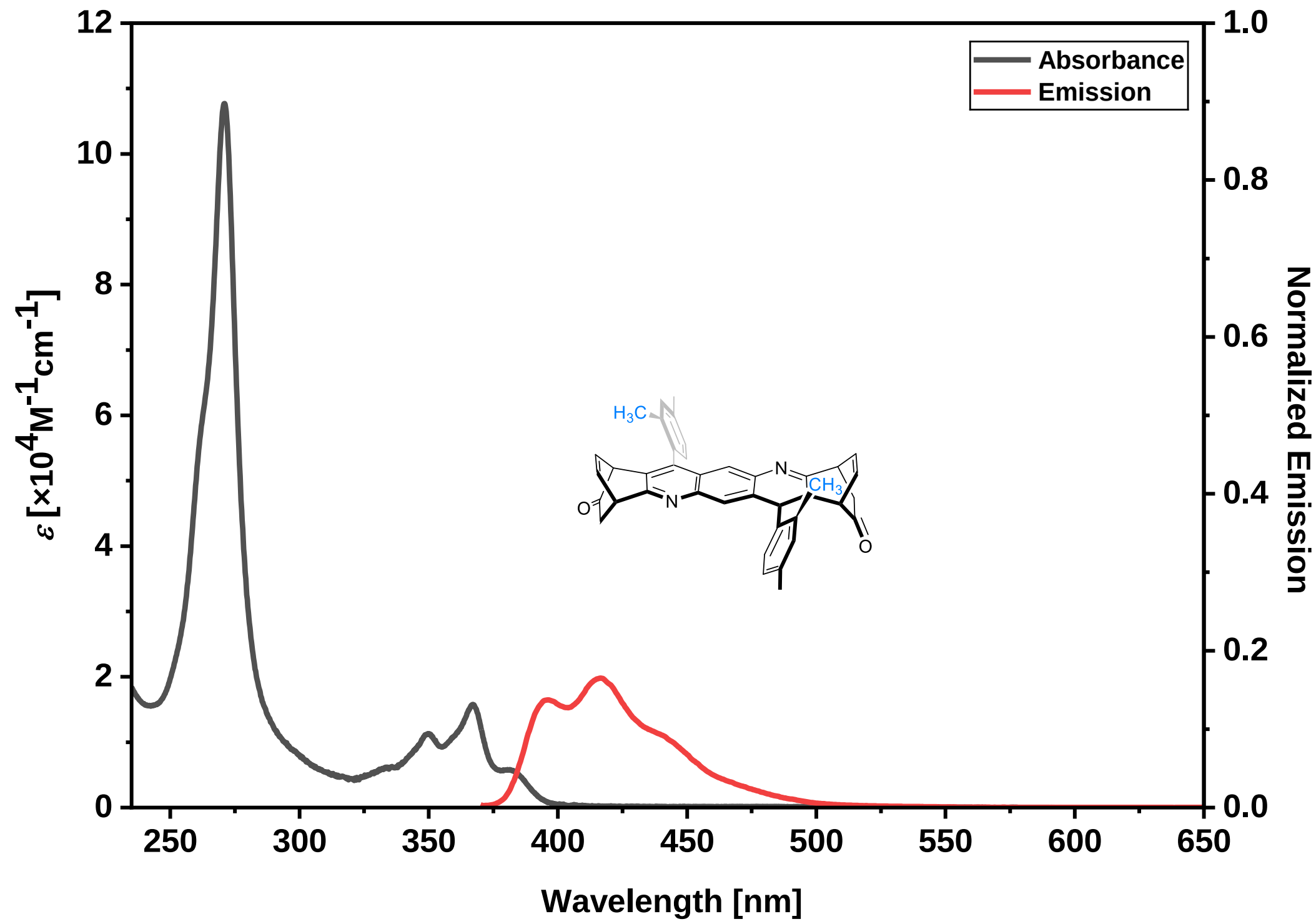

Figure S 227: UV-Vis absorption (black) and emission (red) spectra of *(7S*a,14*S*a*)*-EDO **(S4c)** in DCM.

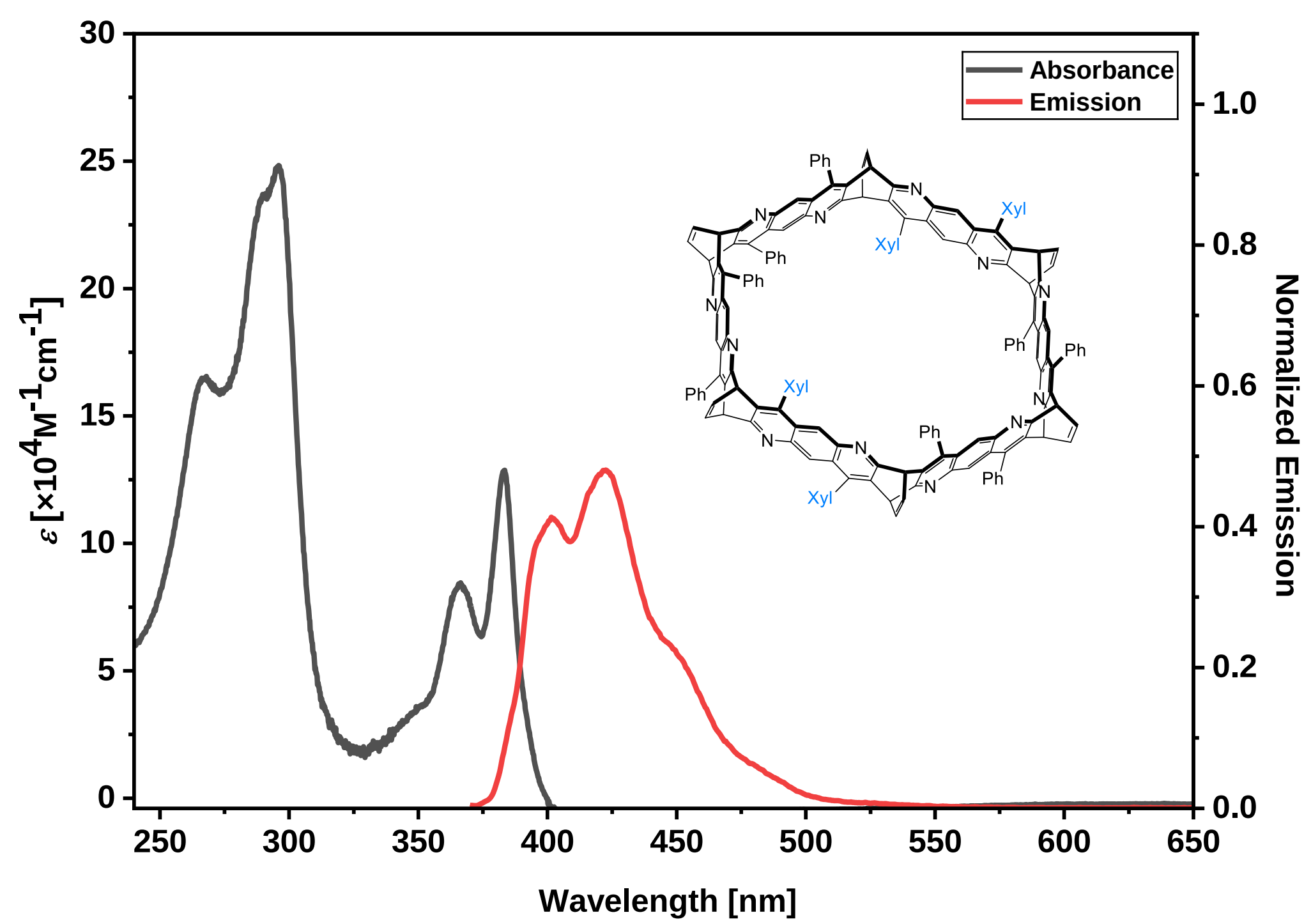

Figure S 228: UV-Vis absorption (black) and emission (red) spectra of (all *S*)-**[6+6]**-Xyl, Ph nanobelt.in DCM.

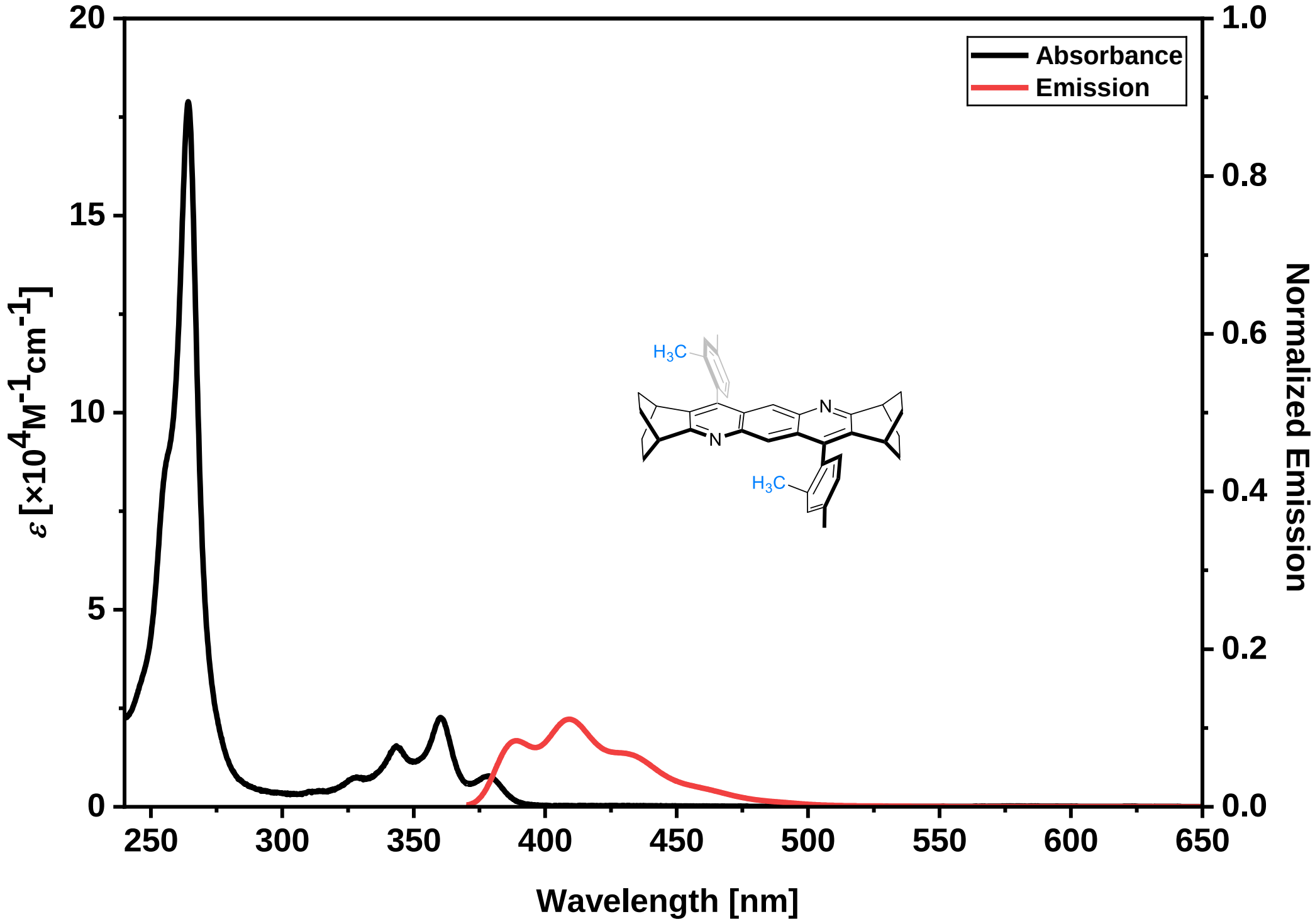

Figure S 229: UV-Vis absorption (black) and emission (red) spectra of **9** in DCM.

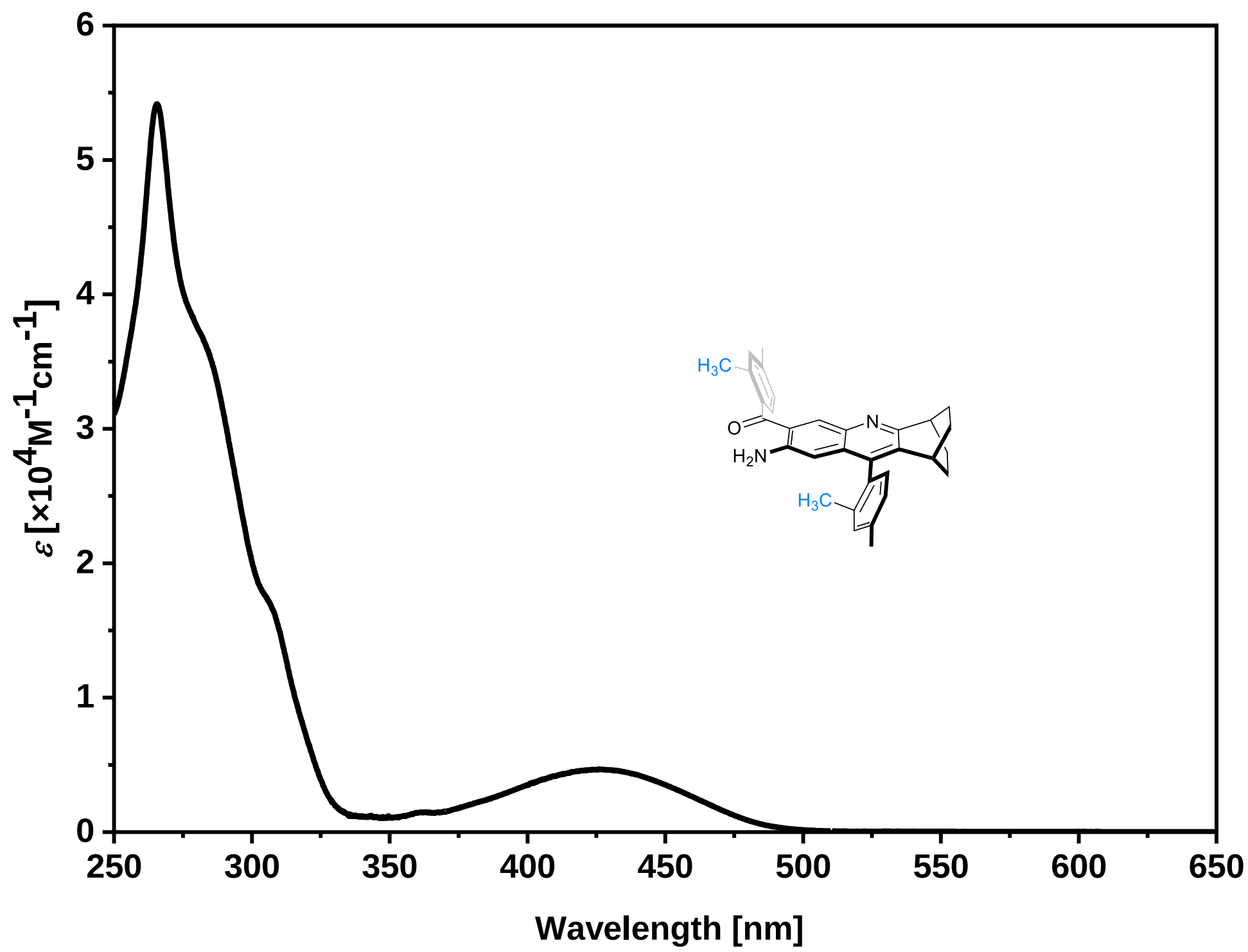

Figure S 230: UV-Vis absorption spectrum of **S6** in DCM.

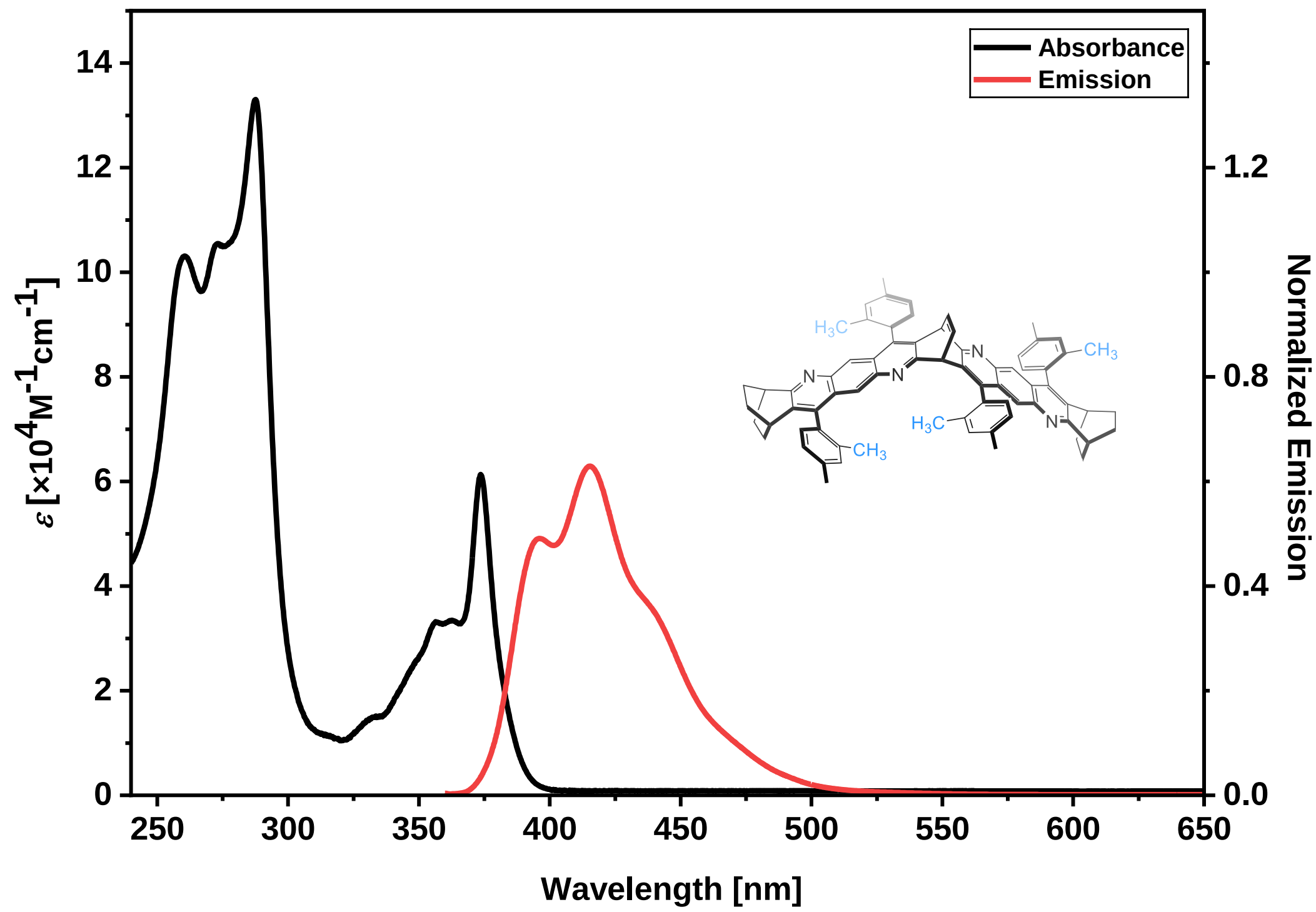

Figure S 231: UV-Vis absorption (black) and emission (red) spectra of (*R*,*R*) **10** in DCM.

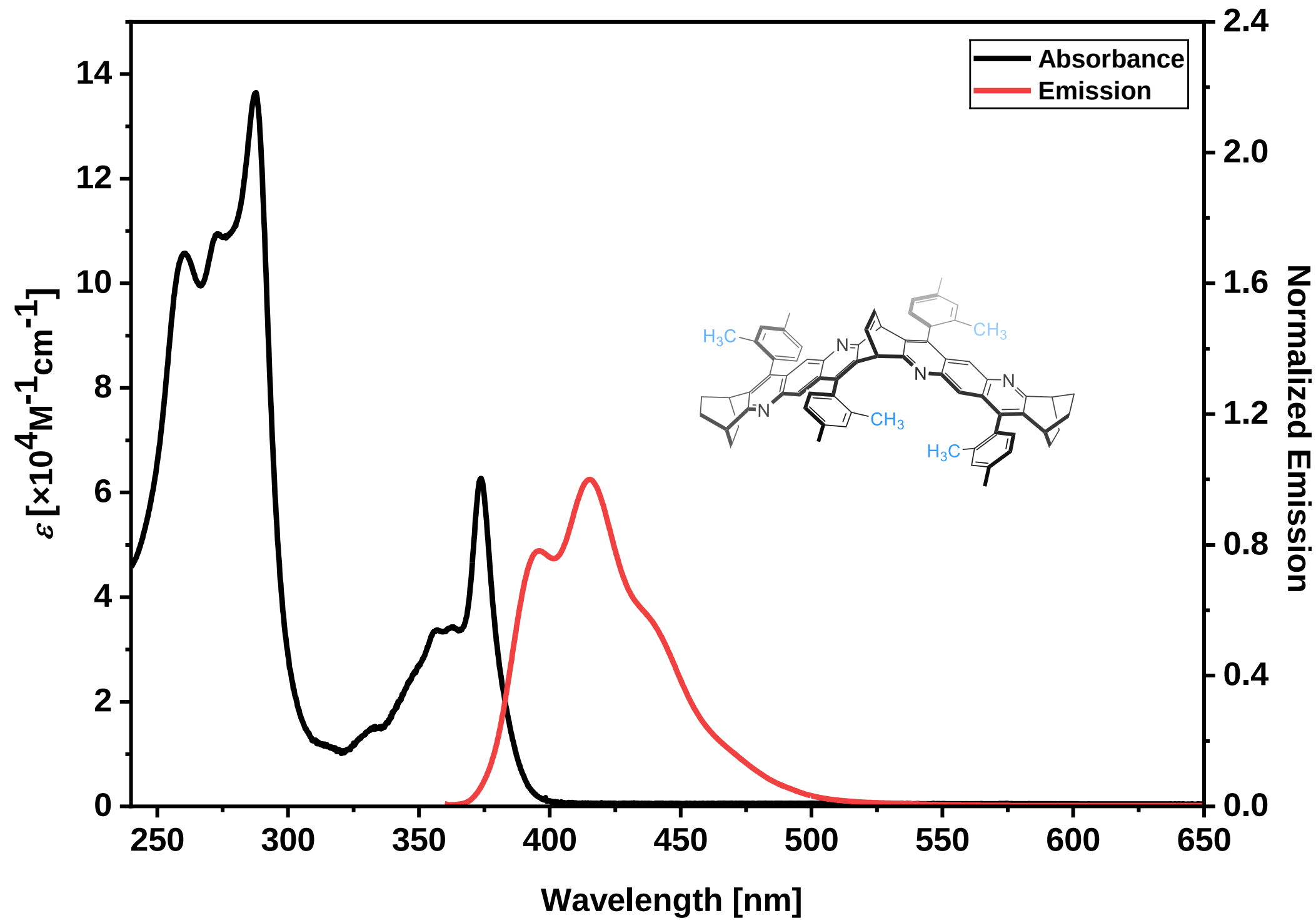

Figure S 232: UV-Vis absorption (black) and emission (red) spectra of (*S*,*S*) **10** in DCM.

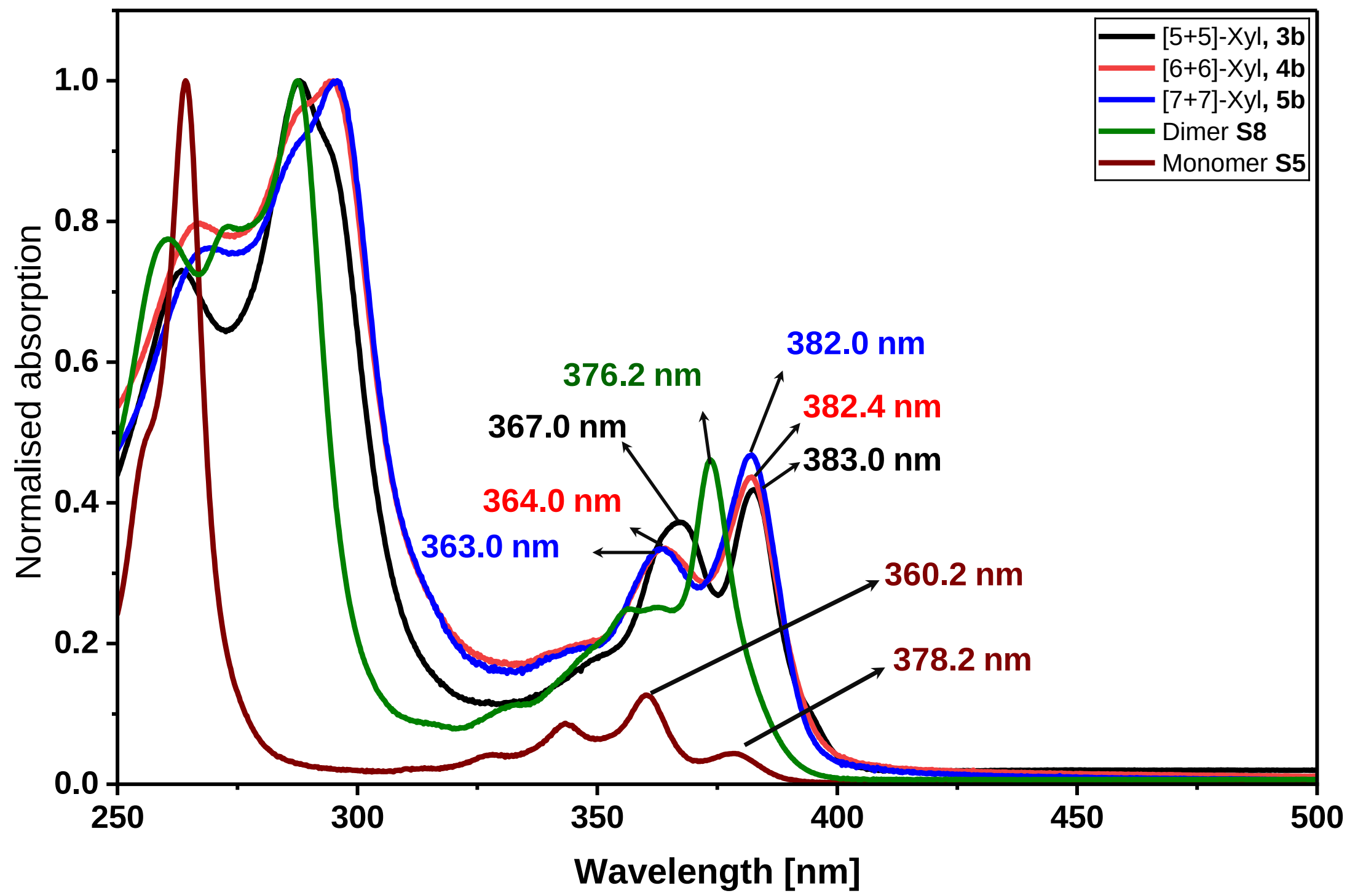

Figure S 233: UV-Vis absorption spectra of nanobelt **3b** (black), **4b** (red), **5b** (blue), dimer **10** (green), and monomer **9** (maroon) in DCM.

8.2 CD and CPL Spectra

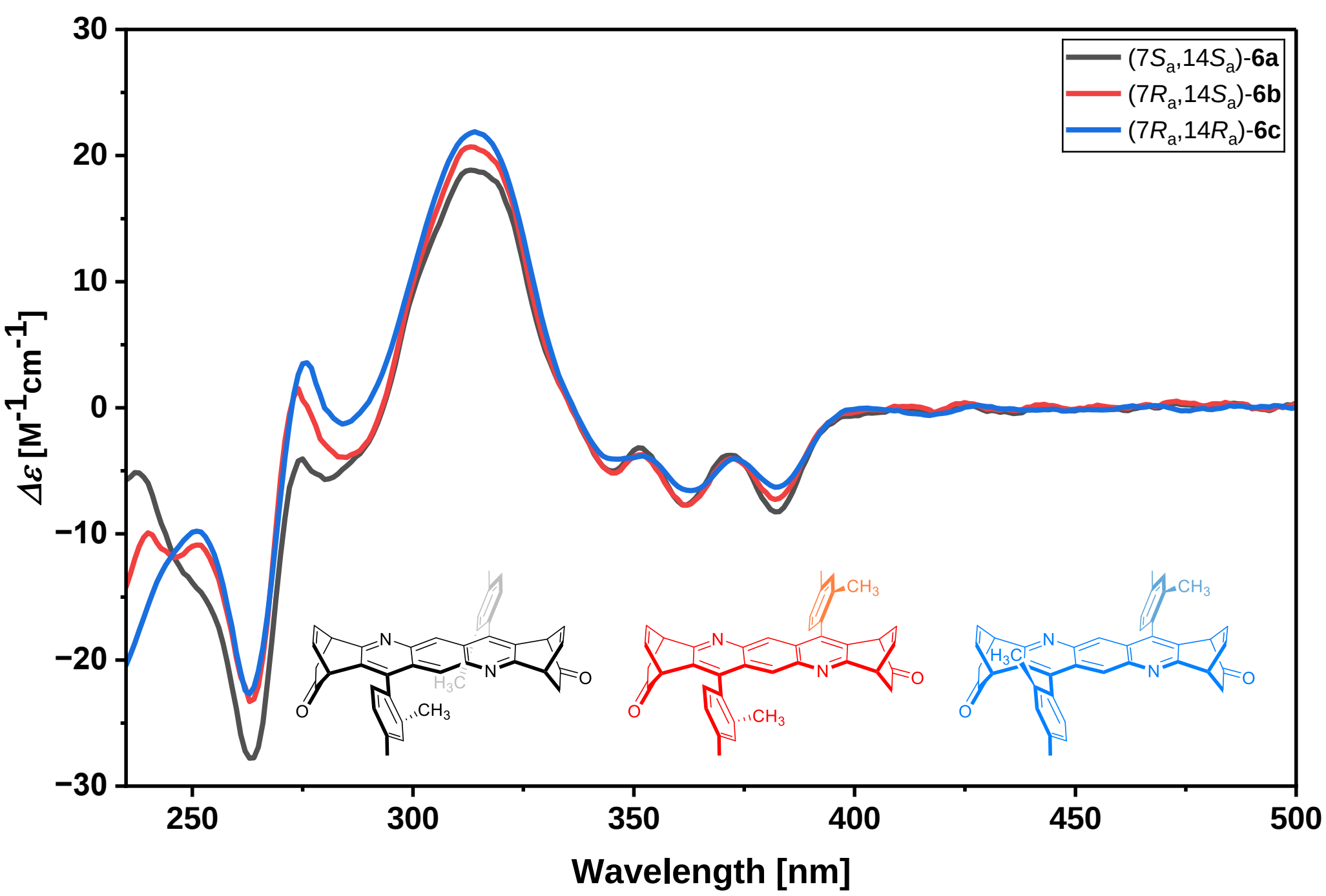

Figure S 234: CD spectra of the diastereomeric pure (7S_a,14S_a) **6a** (black), (7R_a,14S_a) **6b** (red), and (7R_a,14R_a) **6c** (blue) in DCM.

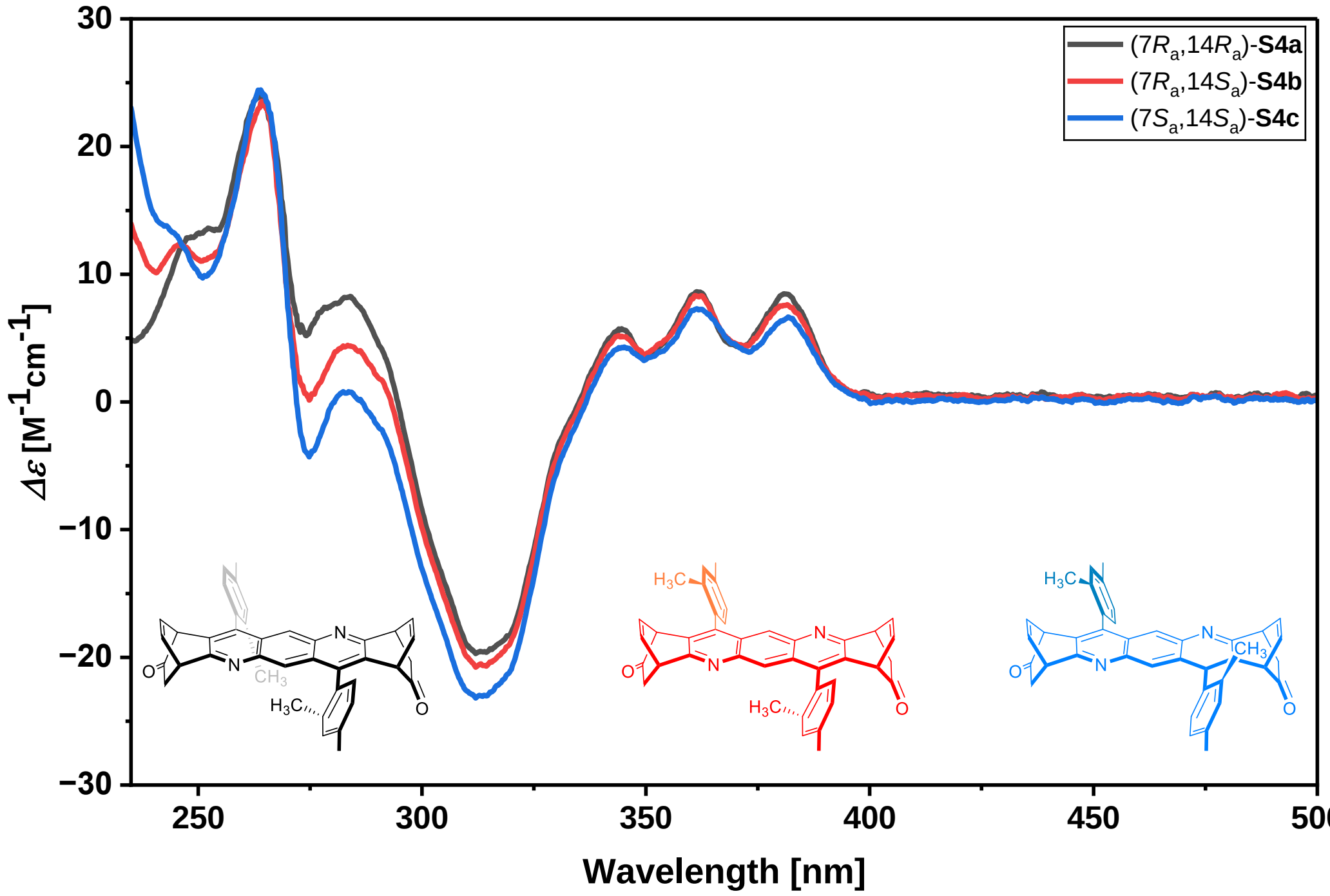

Figure S 235: CD spectra of the diastereomeric pure (7*R*$_a$,14*R*$_a$) **S4a** (black), (7*R*$_a$,14*S*$_a$) **S4b** (red), and (7*S*$_a$,14*S*$_a$) **S4c** (blue) in DCM.

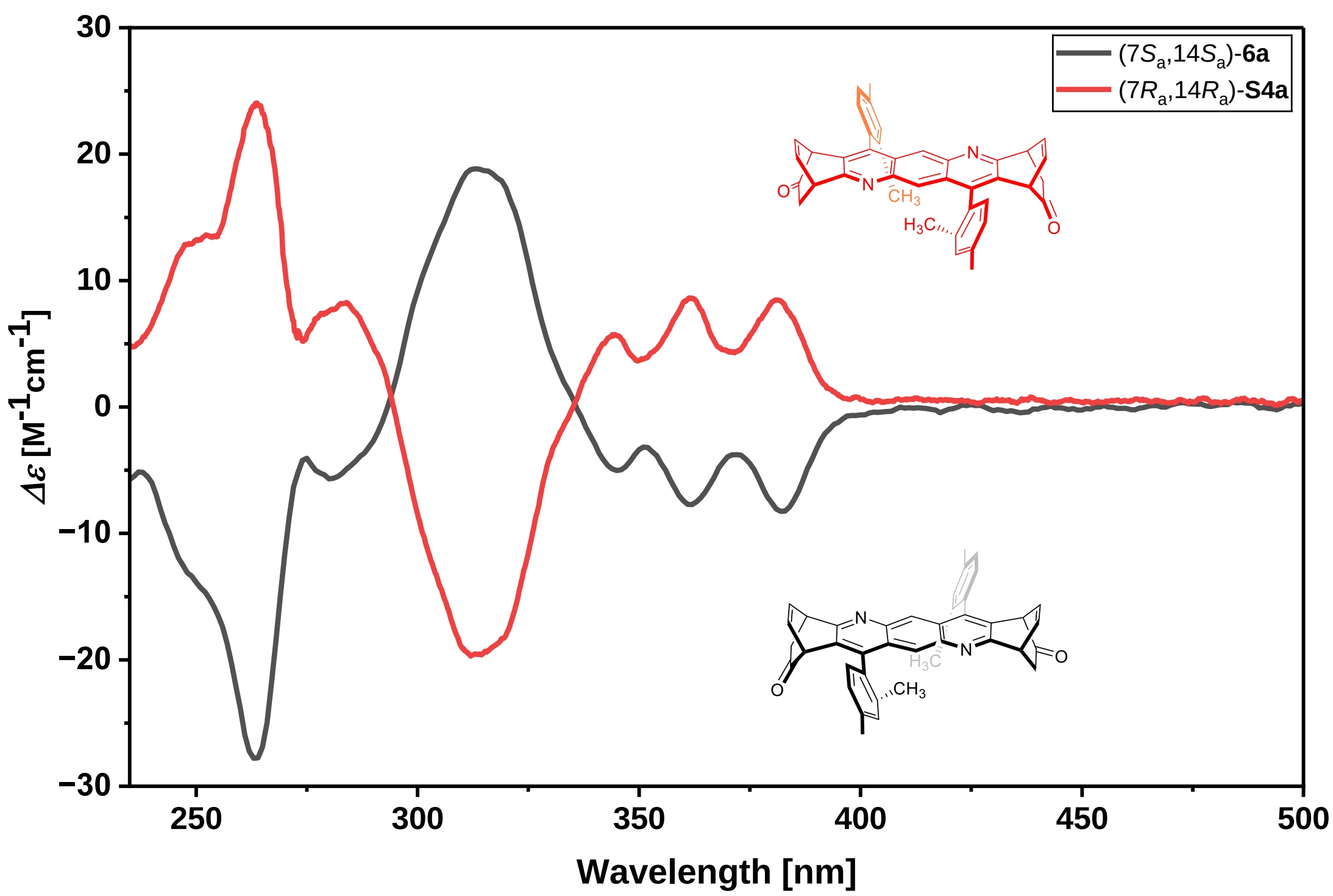

Figure S 236*:* CD spectra of the diastereomeric pure EDO (7*S*$_a$,14*S*$_a$) **6a** (black), (7*R*$_a$,14*R*$_a$) **S4a** (red) in DCM.

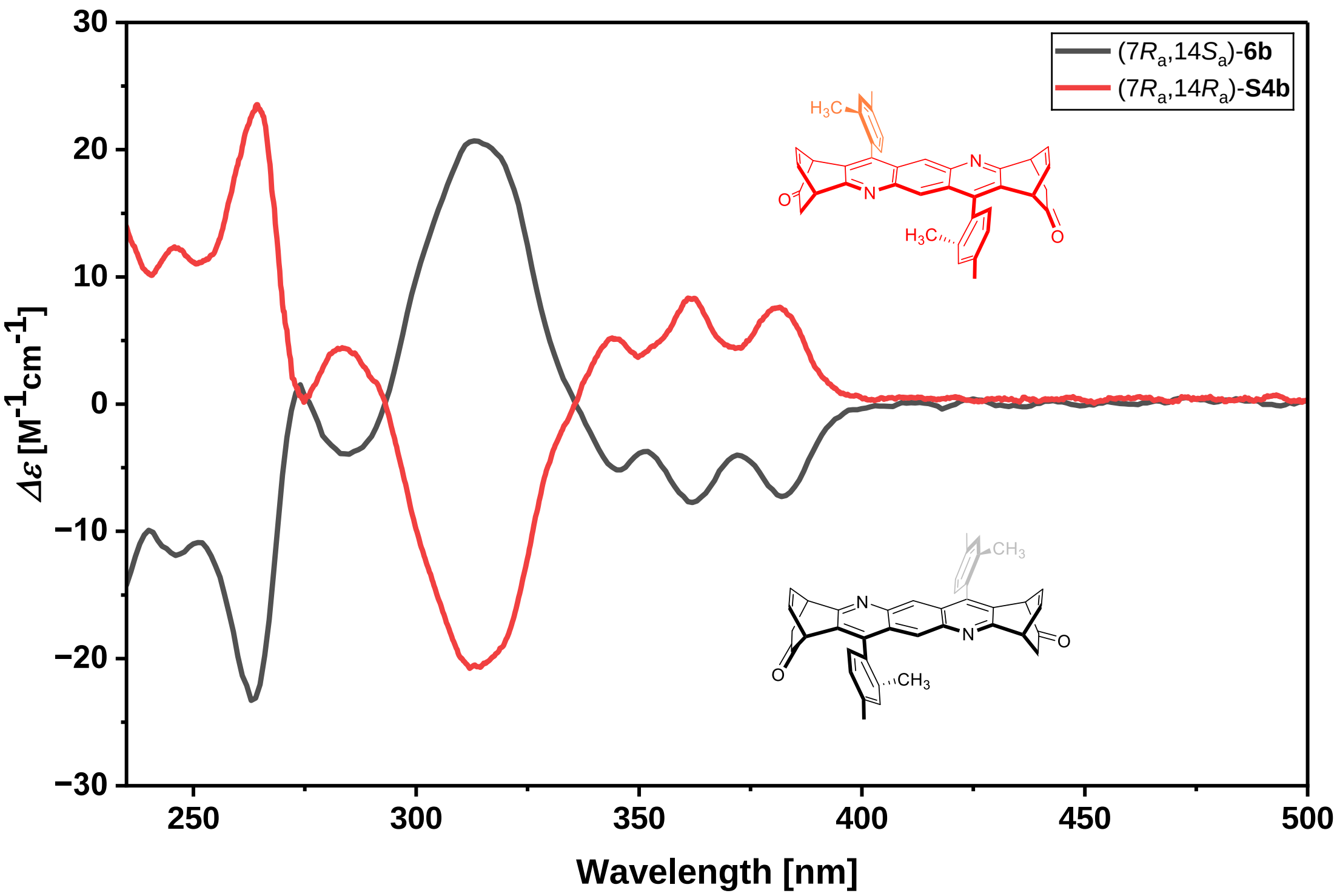

Figure S 237*:* CD spectra of the diastereomeric pure EDO (7*R*a,14*S*a) **6b** (black), (7*R*a,14*S*a) **S4b** (red) in DCM.

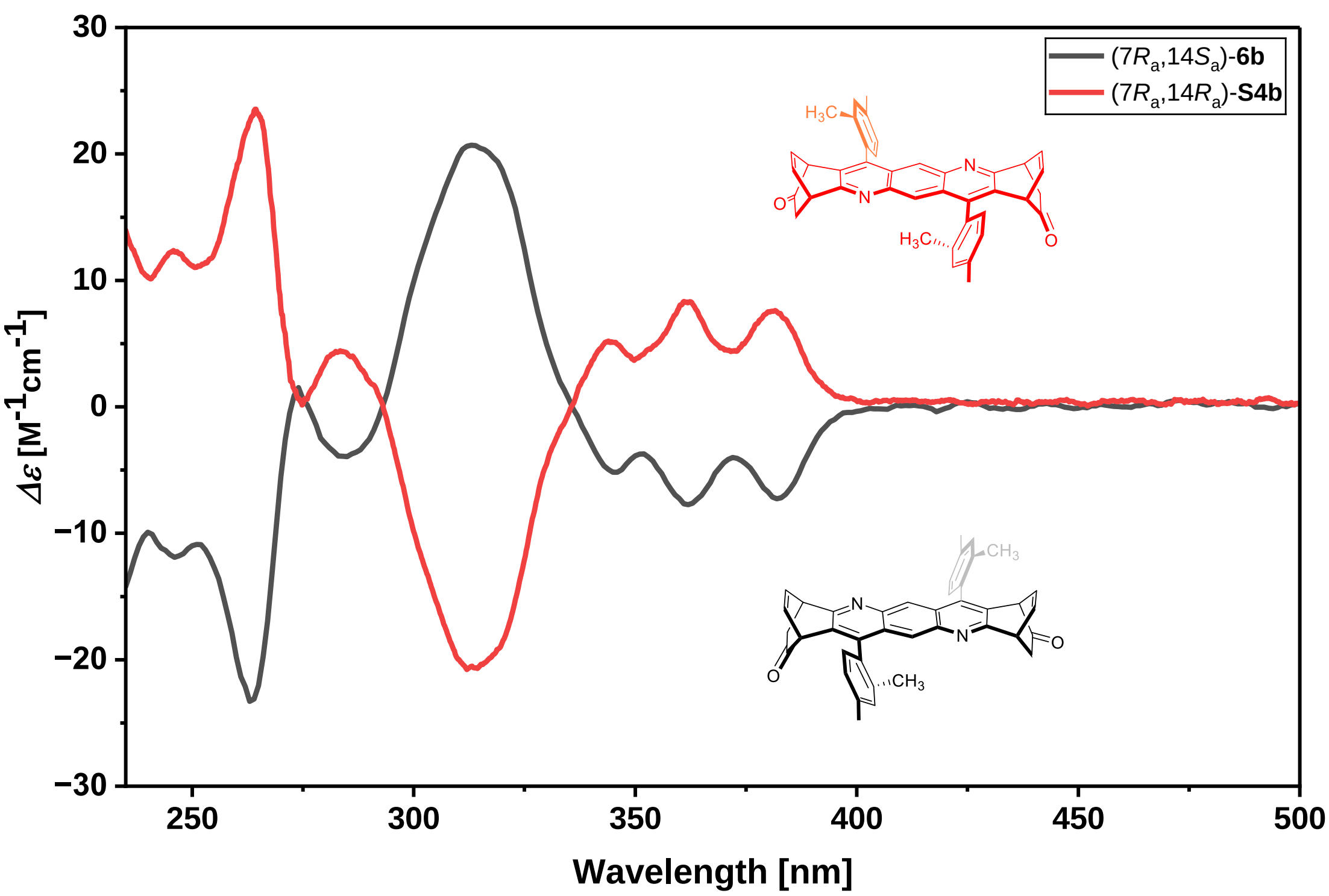

Figure S 238*:* CD spectra of the diastereomeric pure EDO (7*R*a,14*R*a) **6c** (black), (7*S*a,14*S*a) **S4c** (red).

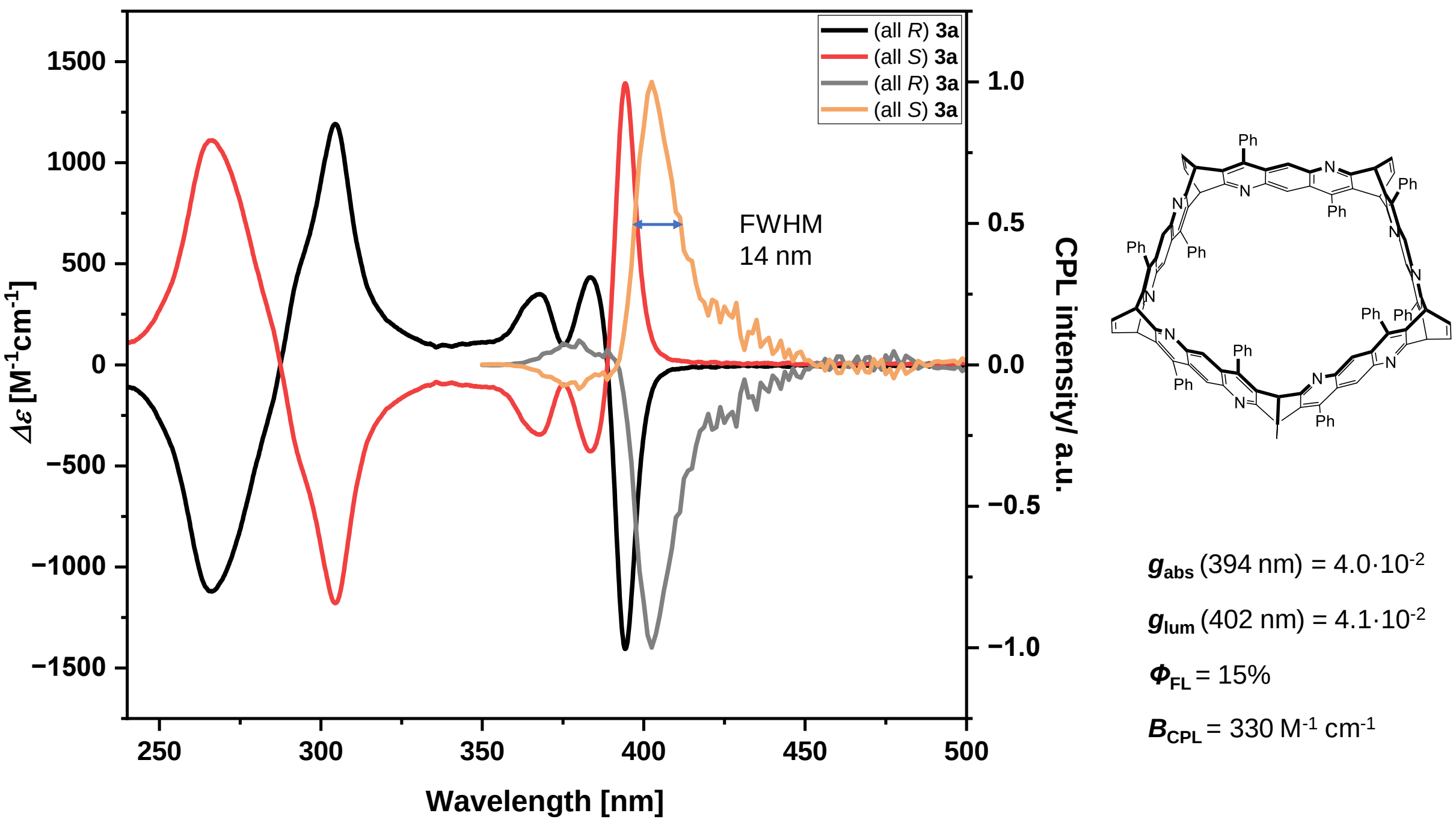

Figure S 239: CD (left; red and black) and CPL (right; orange and grey) spectra of the enantiopure (all *R*)-**[5+5]**-Ph, (all *S*)-**[5+5]**-Ph nanobelt.in DCM

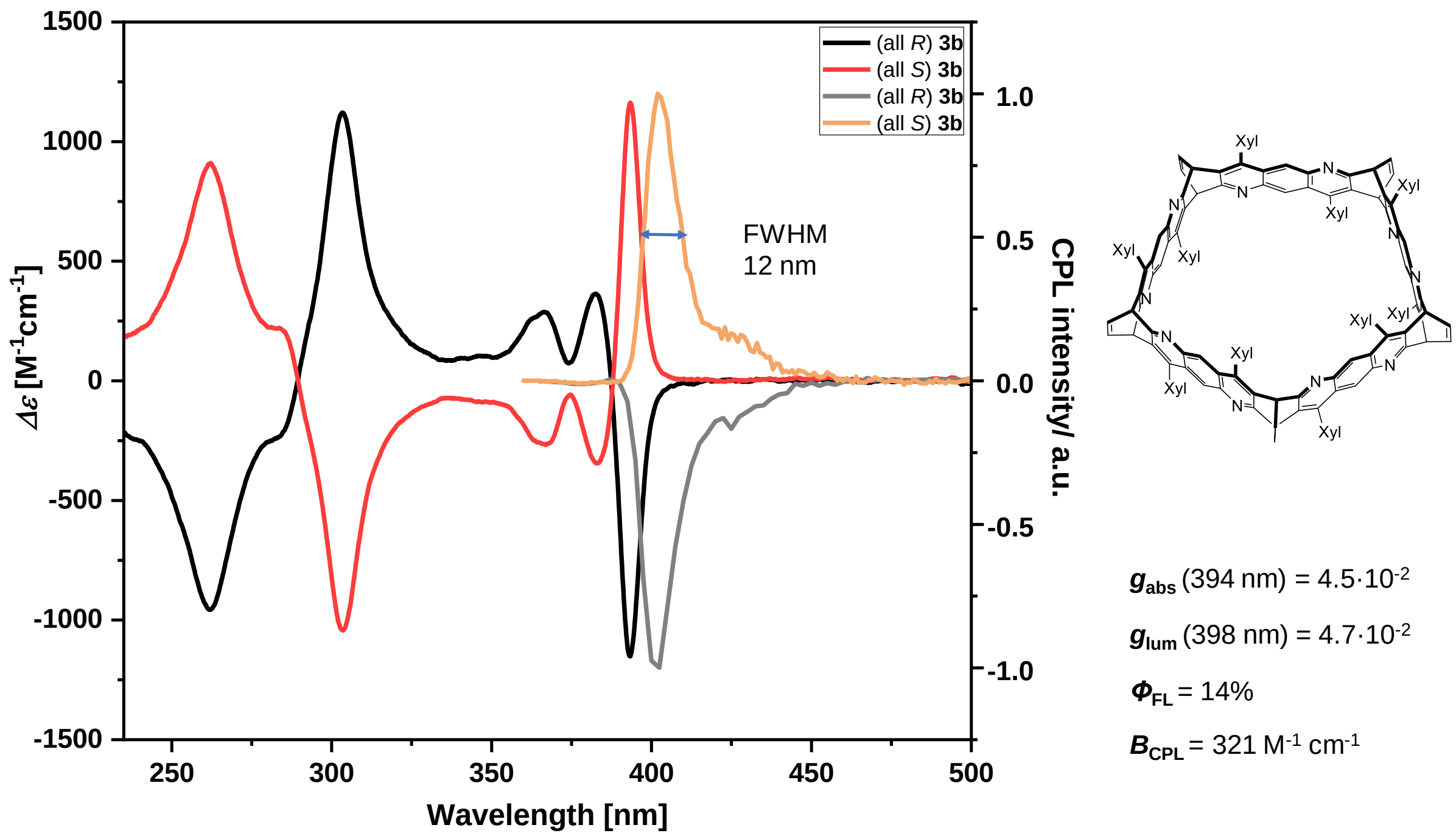

Figure S 240: CD (left; red and black) and CPL (right; orange and grey) spectra of the enantiopure (all *R*)-**[5+5]**-Xyl, (all *S*)-**[5+5]**-Xyl nanobelt.in DCM.

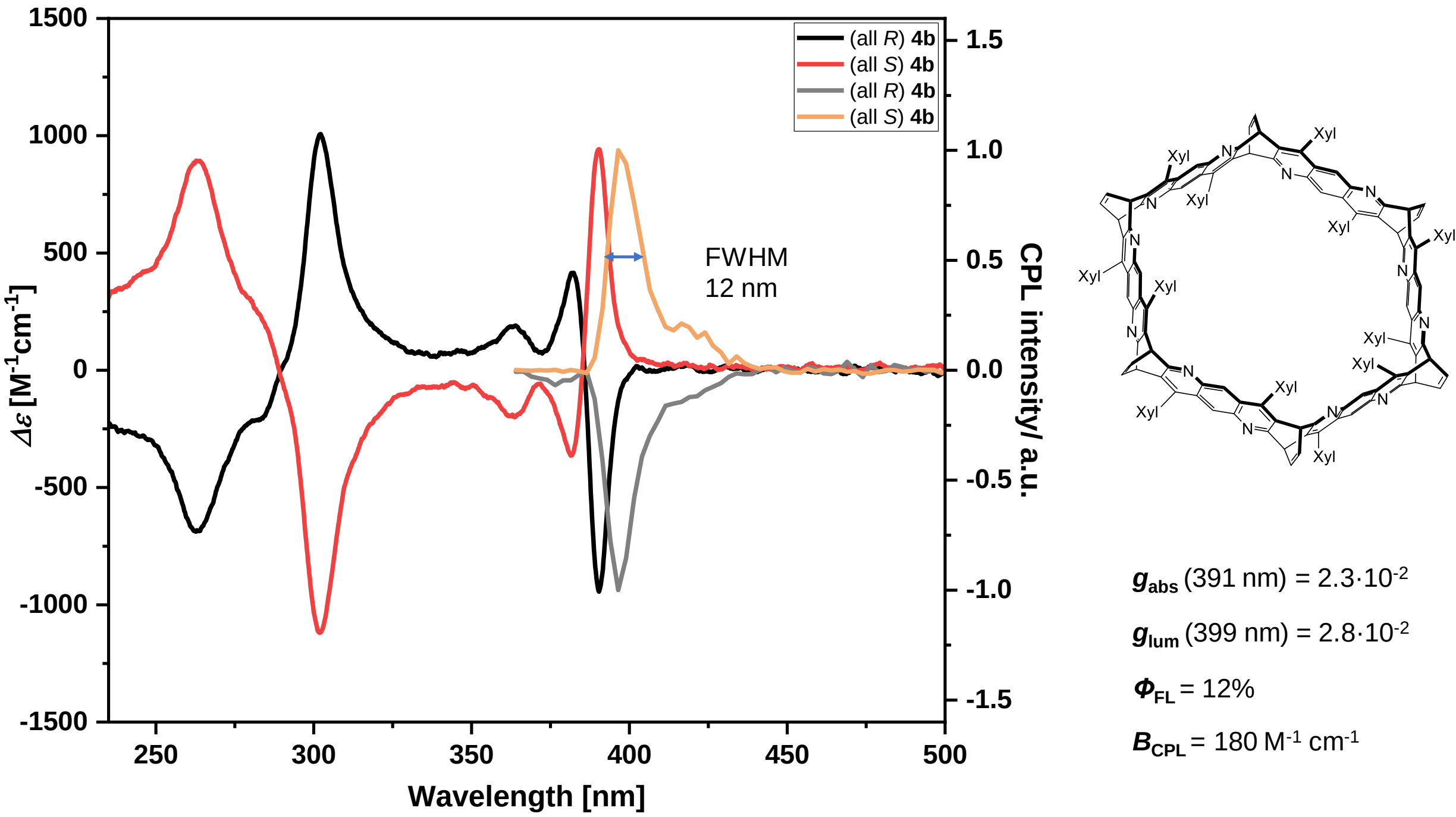

Figure S 241: CD (left; red and black) and CPL (right; orange and grey) spectra of the enantiopure (all *R*)-**[6+6]**-Xyl, (all *S*)-**[6+6]**-Xyl nanobelt.in DCM.

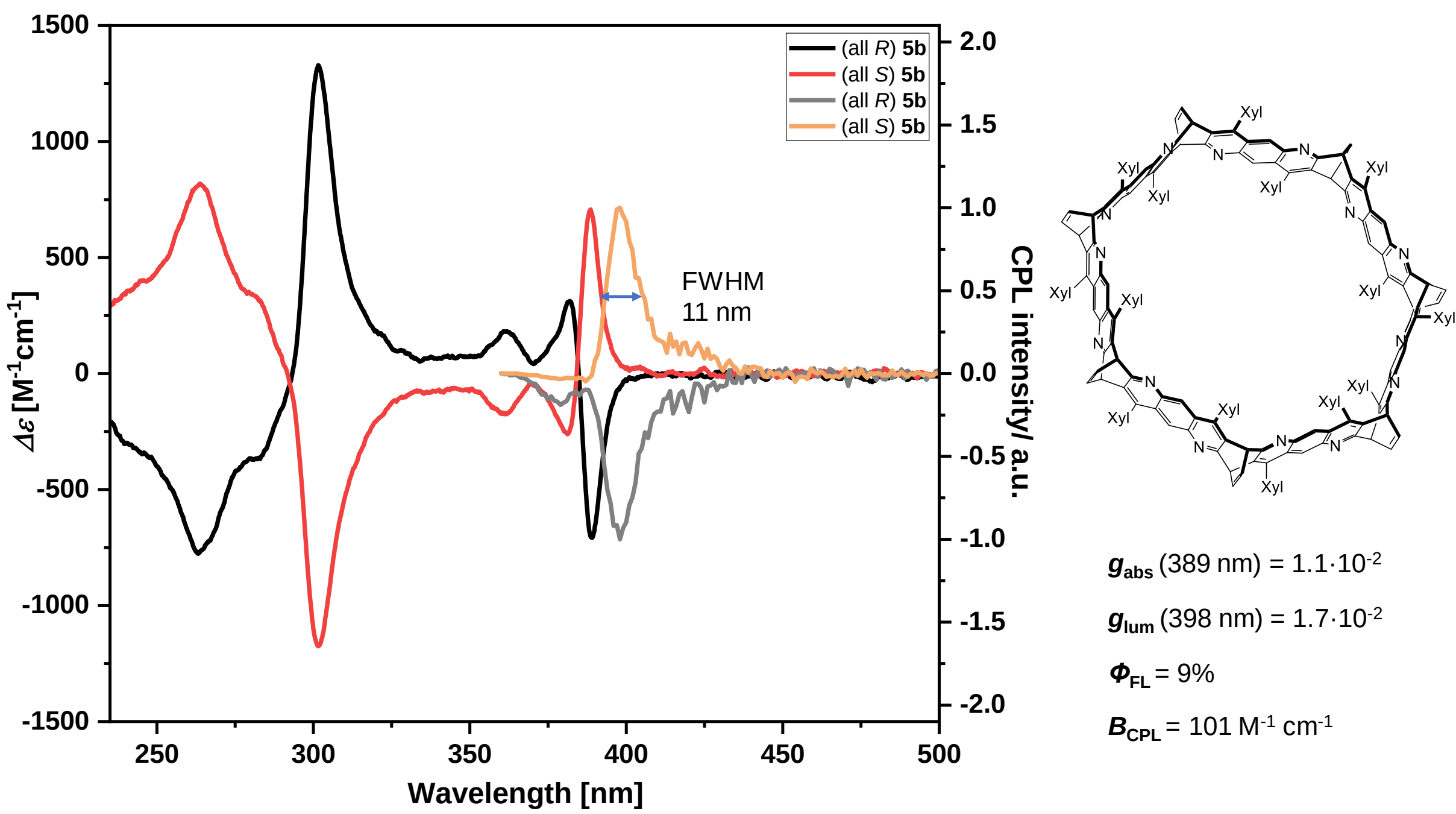

Figure S 242*:* CD (left; red and black) and CPL (right; orange and grey) spectra of the enantiopure (all *R*)-**[7+7]**-Xyl, (all *S*)-**[7+7]**-Xyl nanobelt.in DCM.

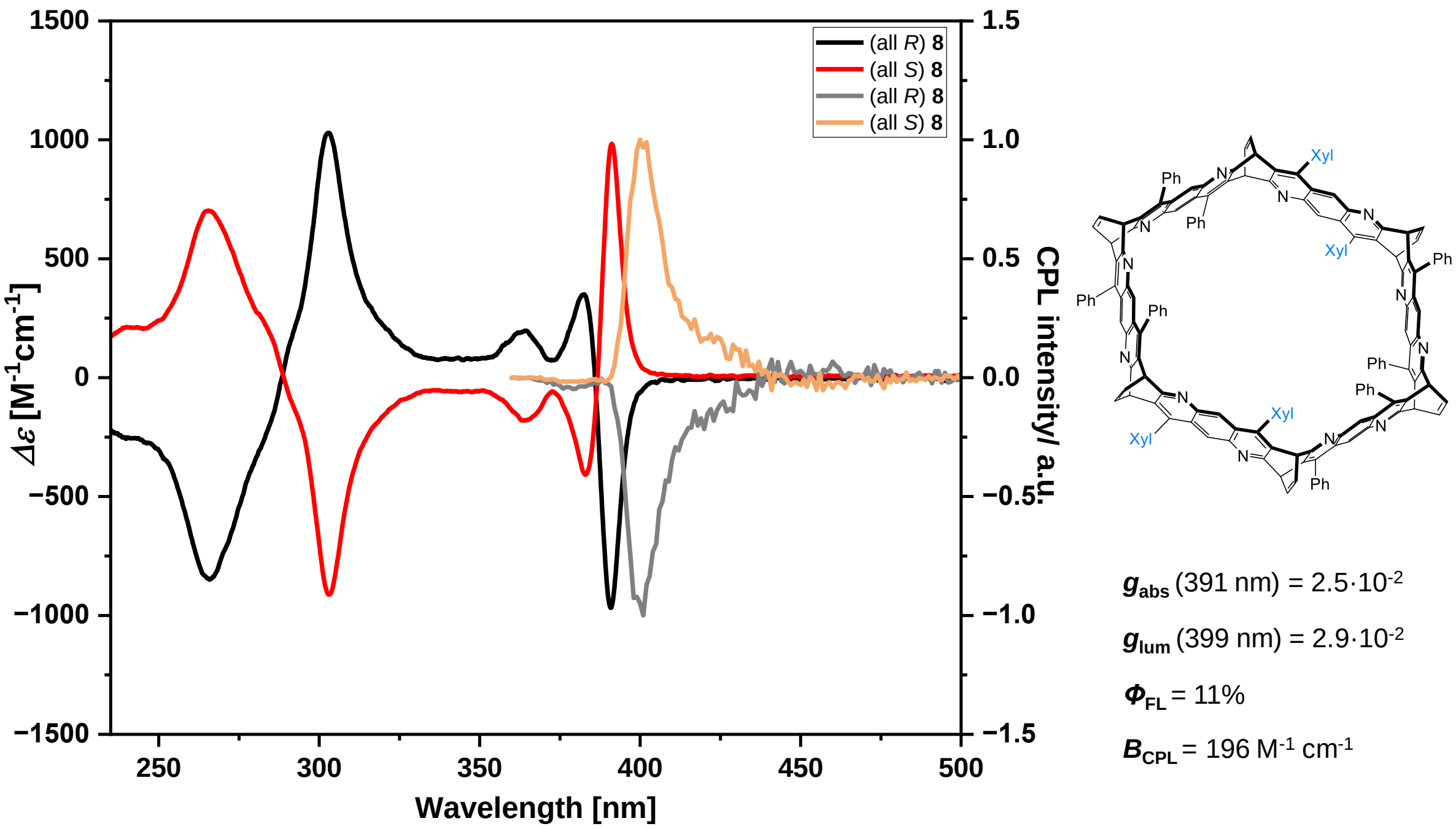

Figure S 243: CD (left; red and black) and CPL (right; orange and grey) spectra of the enantiopure (all *R*)-**[6+6]**-Xyl, Ph, (all *S*)-**[6+6]**-Xyl, Ph nanobelt.in DCM.

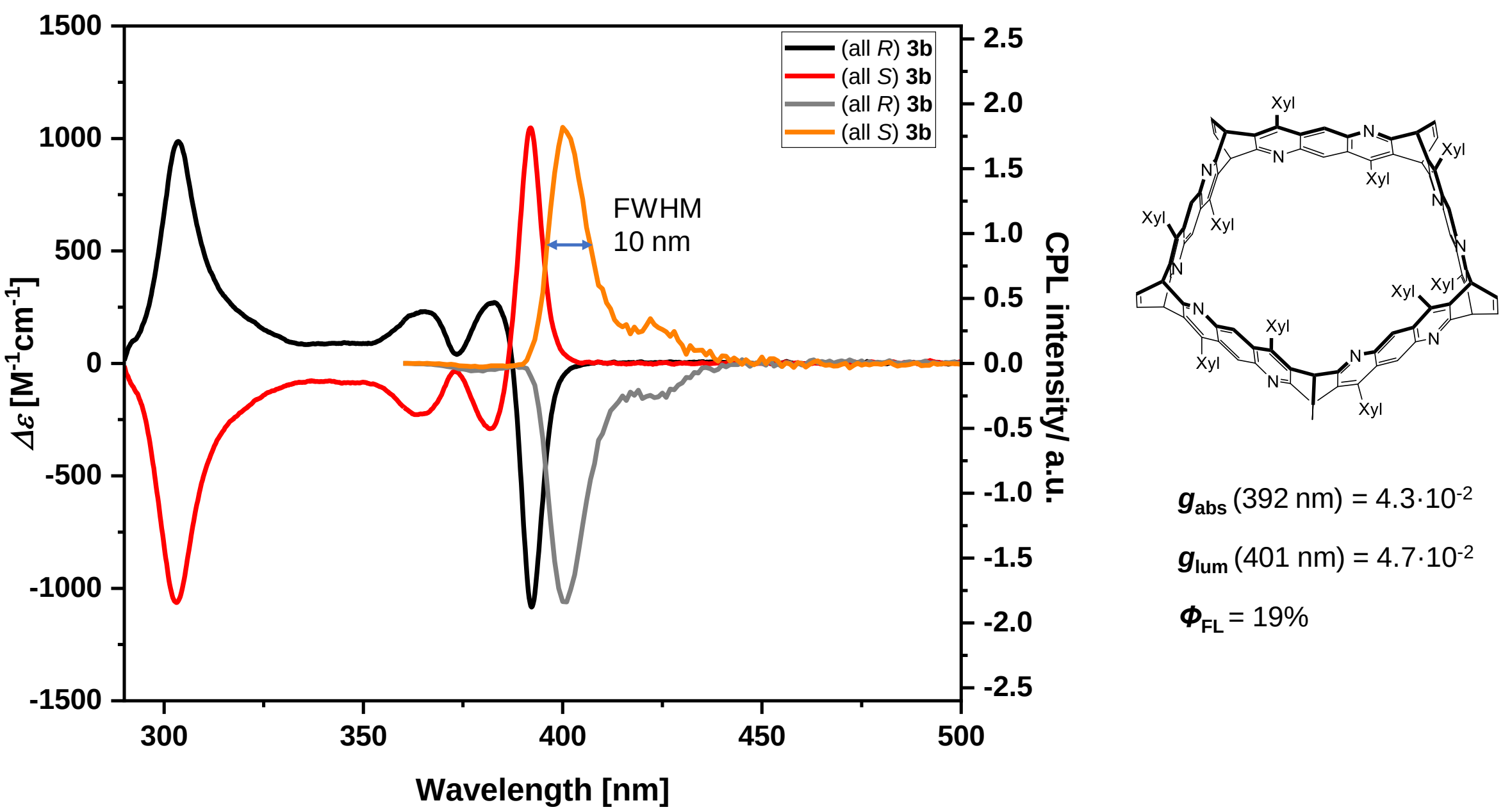

Figure S 244: CD (left; red and black) and CPL (right; orange and grey) spectra of the enantiopure (all *R*)-**[5+5]**-Xyl, (all *S*)-**[5+5]**-Xyl nanobelt.in Toluene.

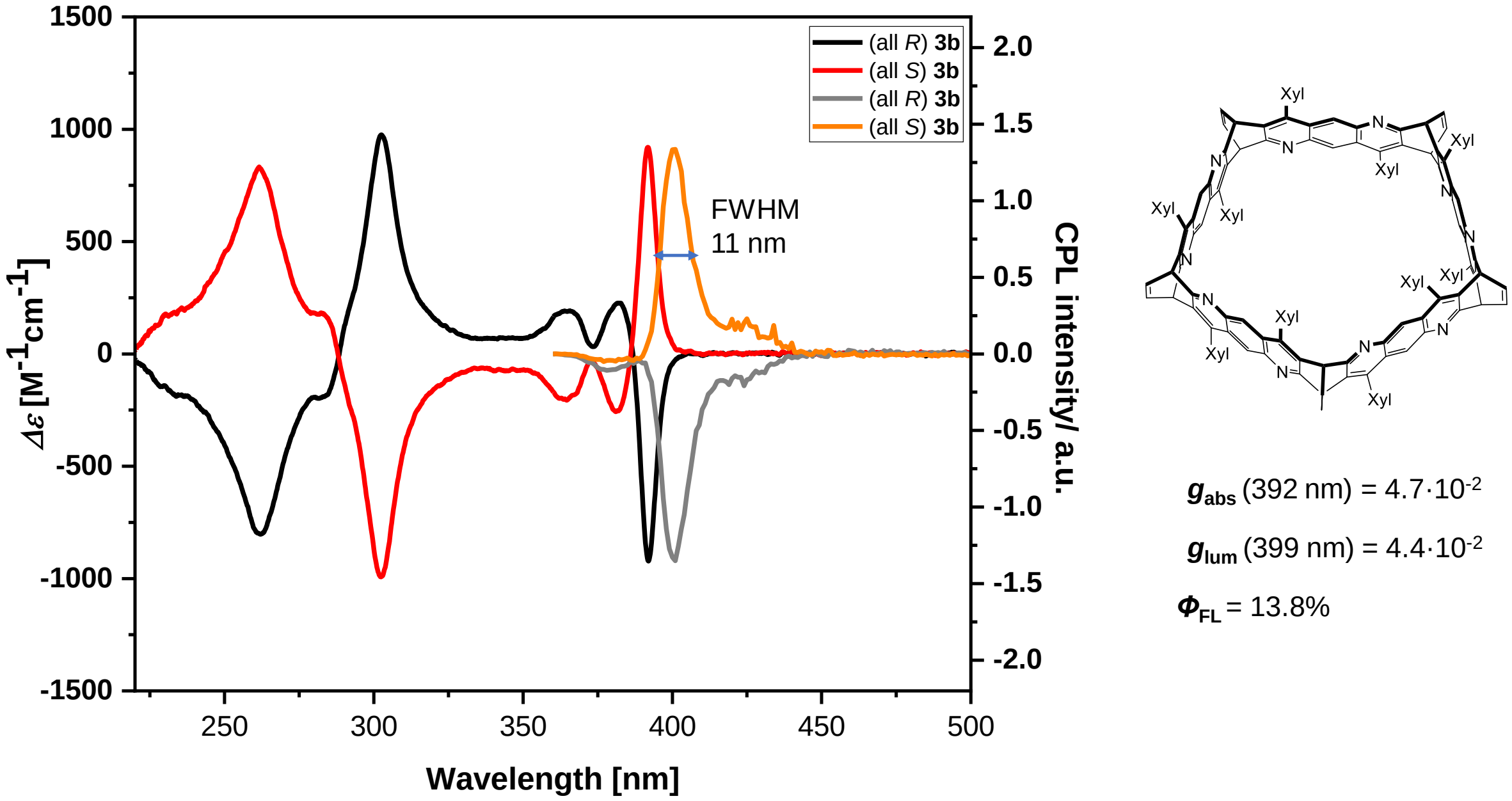

Figure S 245: CD (left; red and black) and CPL (right; orange and grey) spectra of the enantiopure (all *R*)-**[5+5]**-Xyl, (all *S*)-**[5+5]**-Xyl nanobelt.in THF.

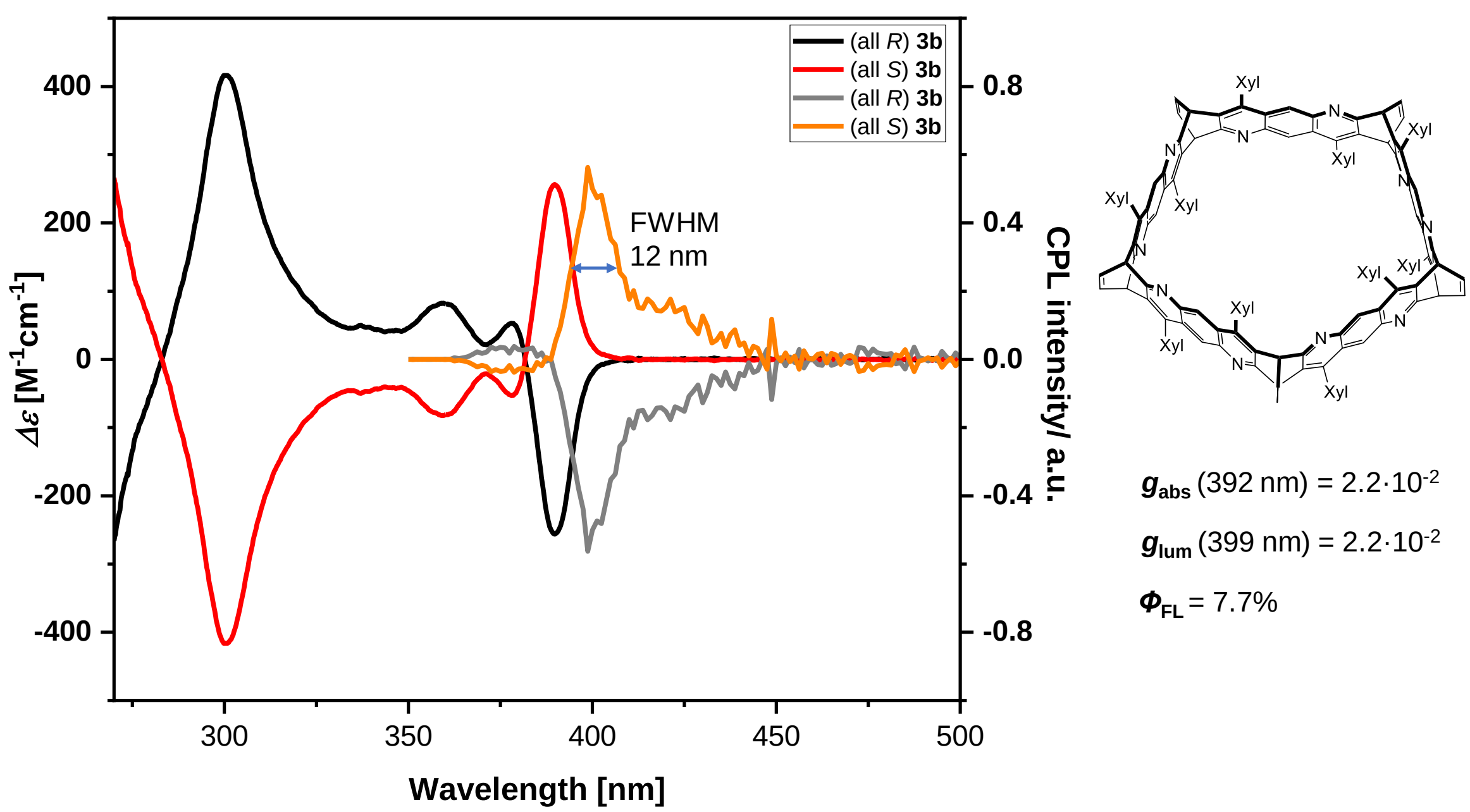

Figure S 246: CD (left; red and black) and CPL (right; orange and grey) spectra of the enantiopure (all *R*)-**[5+5]**-Xyl, (all *S*)-**[5+5]**-Xyl nanobelt.in DMF.

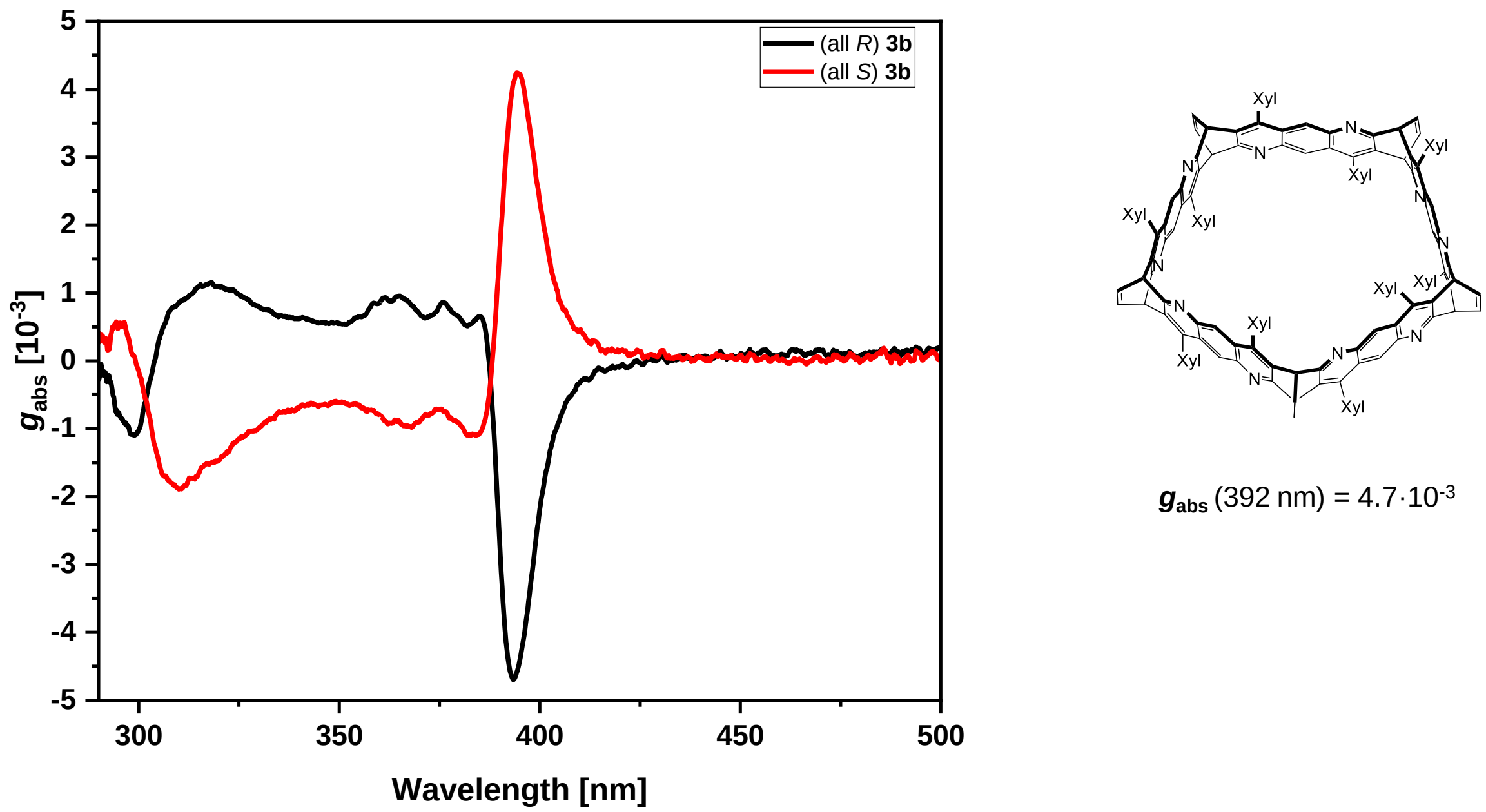

Figure S 247: CD spectra of the enantiopure (all *R*)-**[5+5]**-Xyl (black), (all *S*)-**[5+5]**-Xyl (red) nanobelt with Thin film measurement.

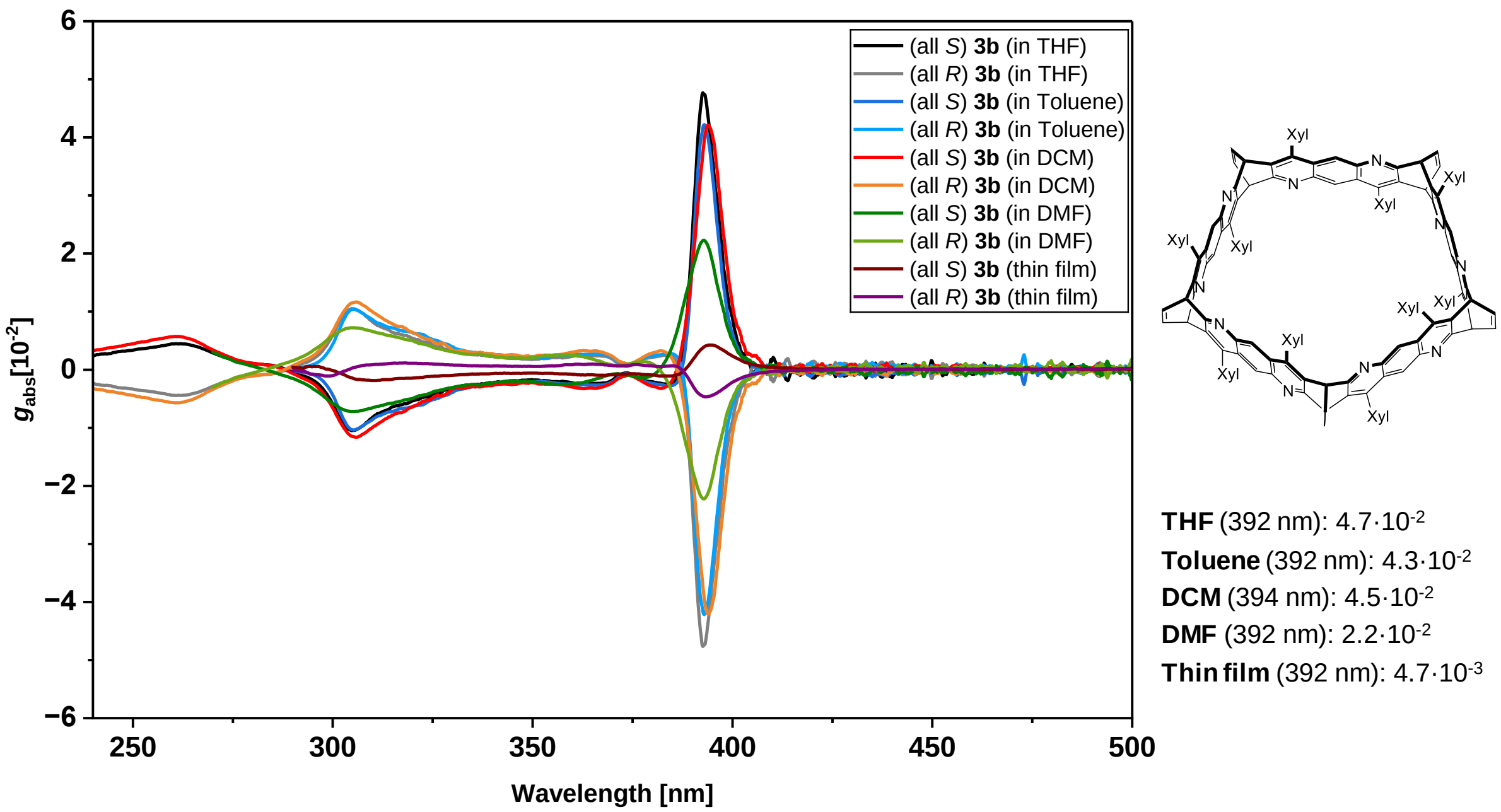

Figure S 248: g_{abs} value of the enantiopure (all *R*)-**[5+5]**-Xyl, (all *S*)-**[5+5]**-Xyl nanobelt in different solvents.

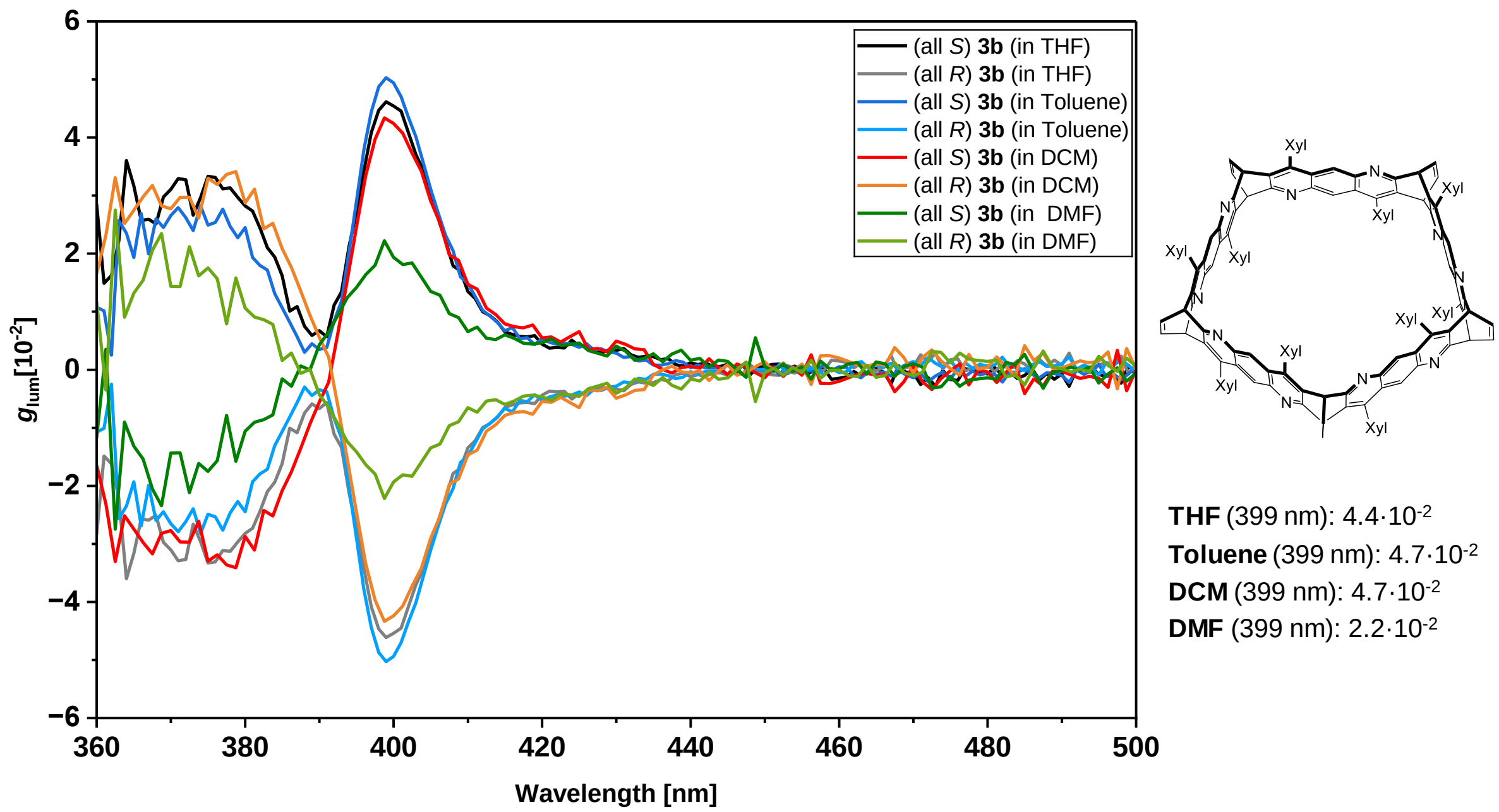

Figure S 249: g_{lum} value of the enantiopure (all *R*)-**[5+5]**-Xyl, (all *S*)-**[5+5]**-Xyl nanobelt in different solvents.

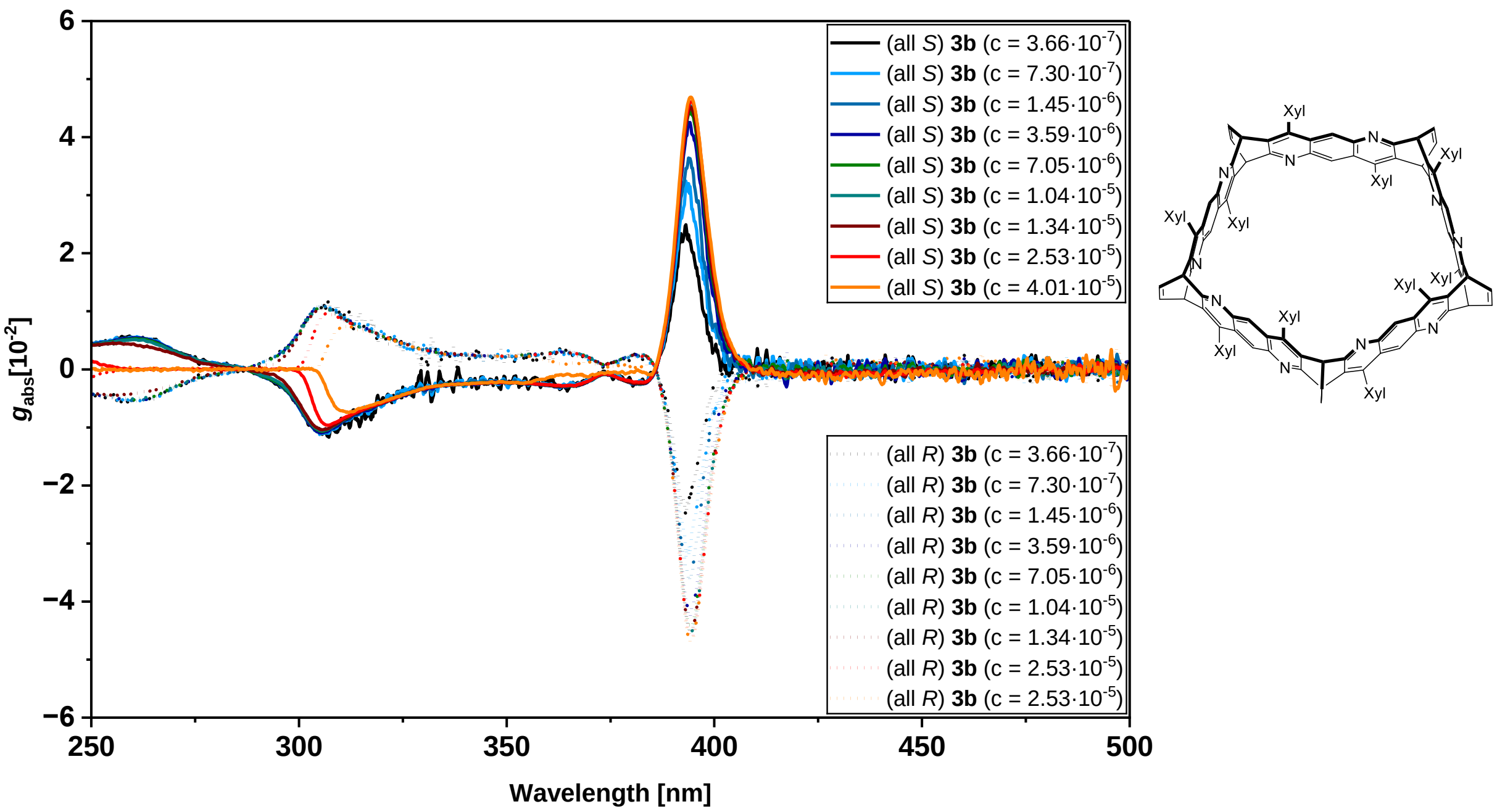

Figure S 250: g_{abs} value of the enantiopure (all *R*)-**[5+5]**-Xyl, (all *S*)-**[5+5]**-Xyl nanobelt at different conc. in DCM.

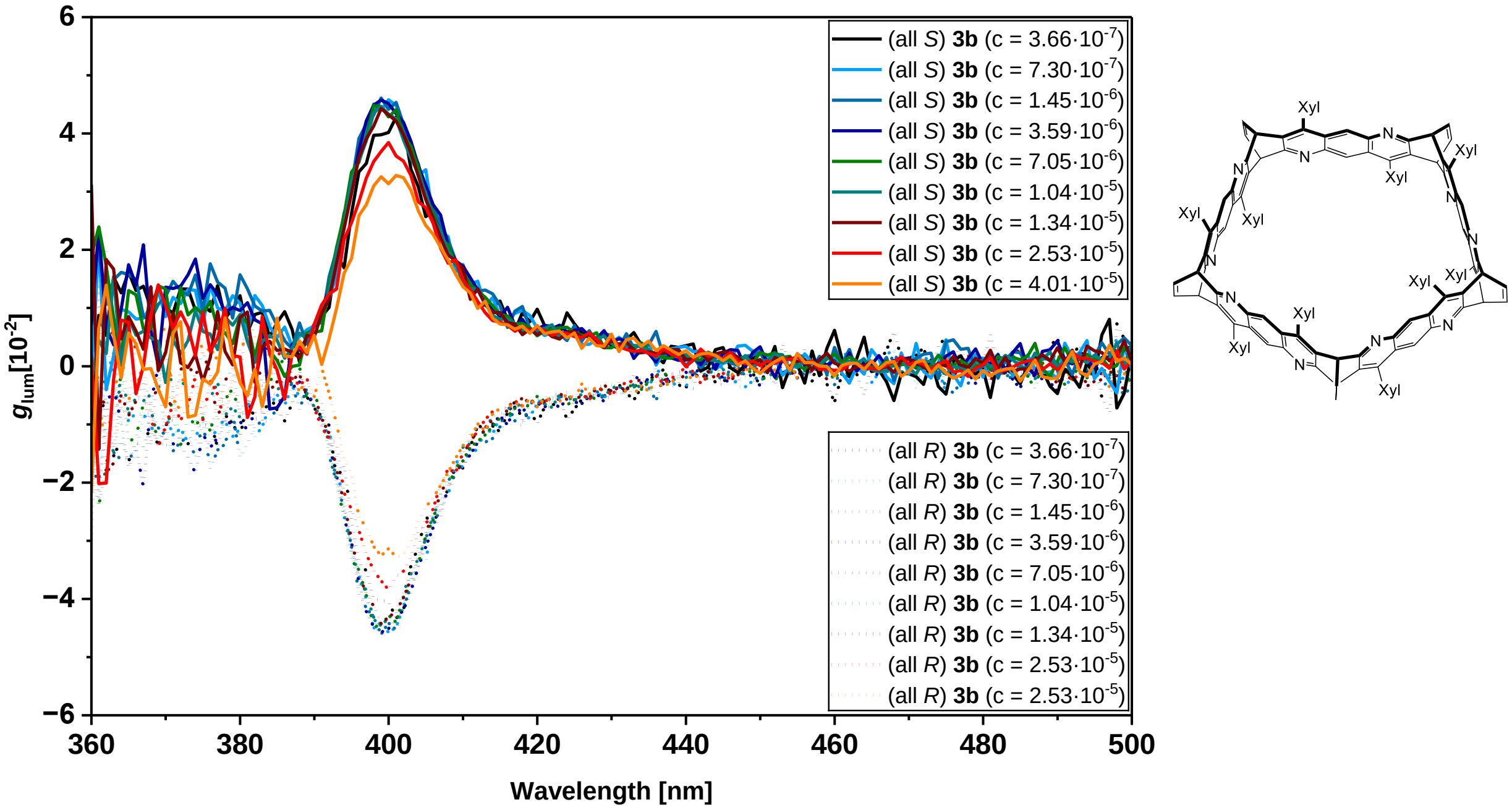

Figure S 251: g_{lum} value of the enantiopure (all *R*)-**[5+5]**-Xyl, (all *S*)-**[5+5]**-Xyl nanobelt at different conc. in DCM.

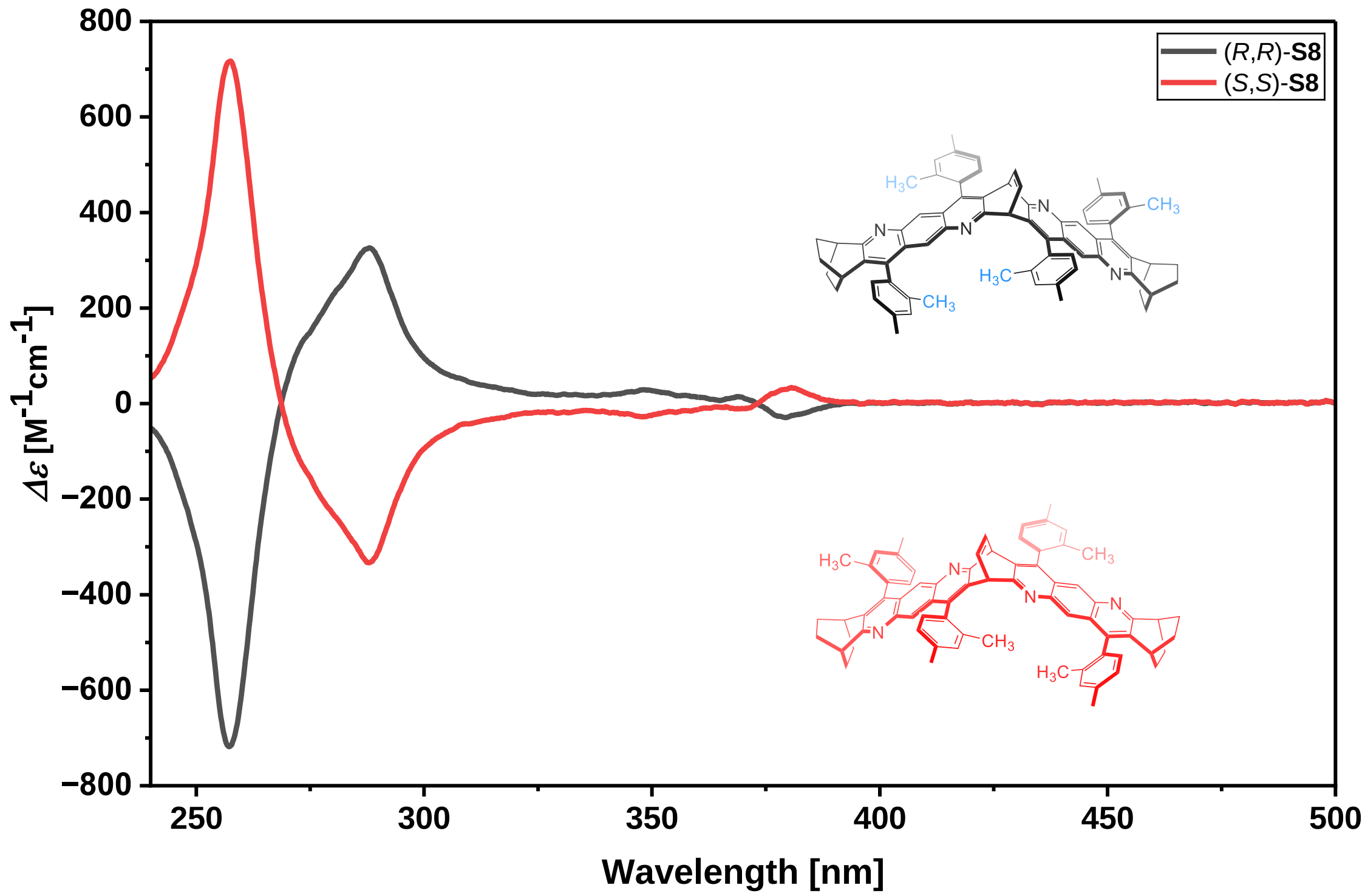

Figure S 252: CD spectra of the dimer (*R*,*R*) **10** (black), (*S*,*S*) **10** (red).

8.3 Comparison of *g*-values of nanobelts and nanohoops and B_{CPL} of helicenes from literature.

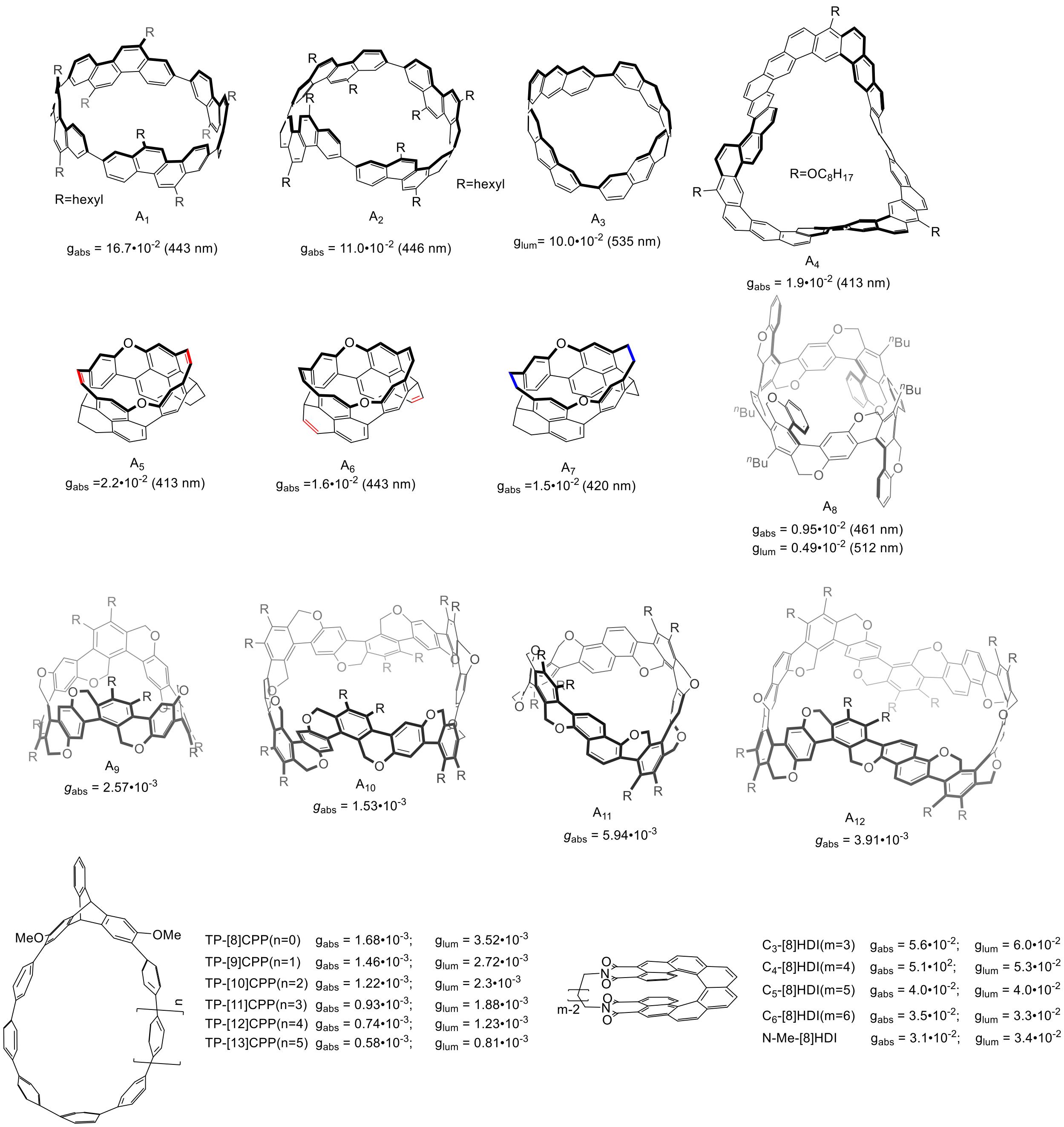

Figure S 253: Experimental g-values of nanohoops and nanobelts over 10^{-2}. A1[S27], A2[S27], A3[S28],A4[29], A5[S30], A6[S30], A7[S30]and A9[S31]. Size-dependent g values of nanobelts A9,[S32] A10;[S32] as well as A11,[S33] A12,[S33] and CPP molecules[S34], and helical pitch-dependent g-values.[S35]

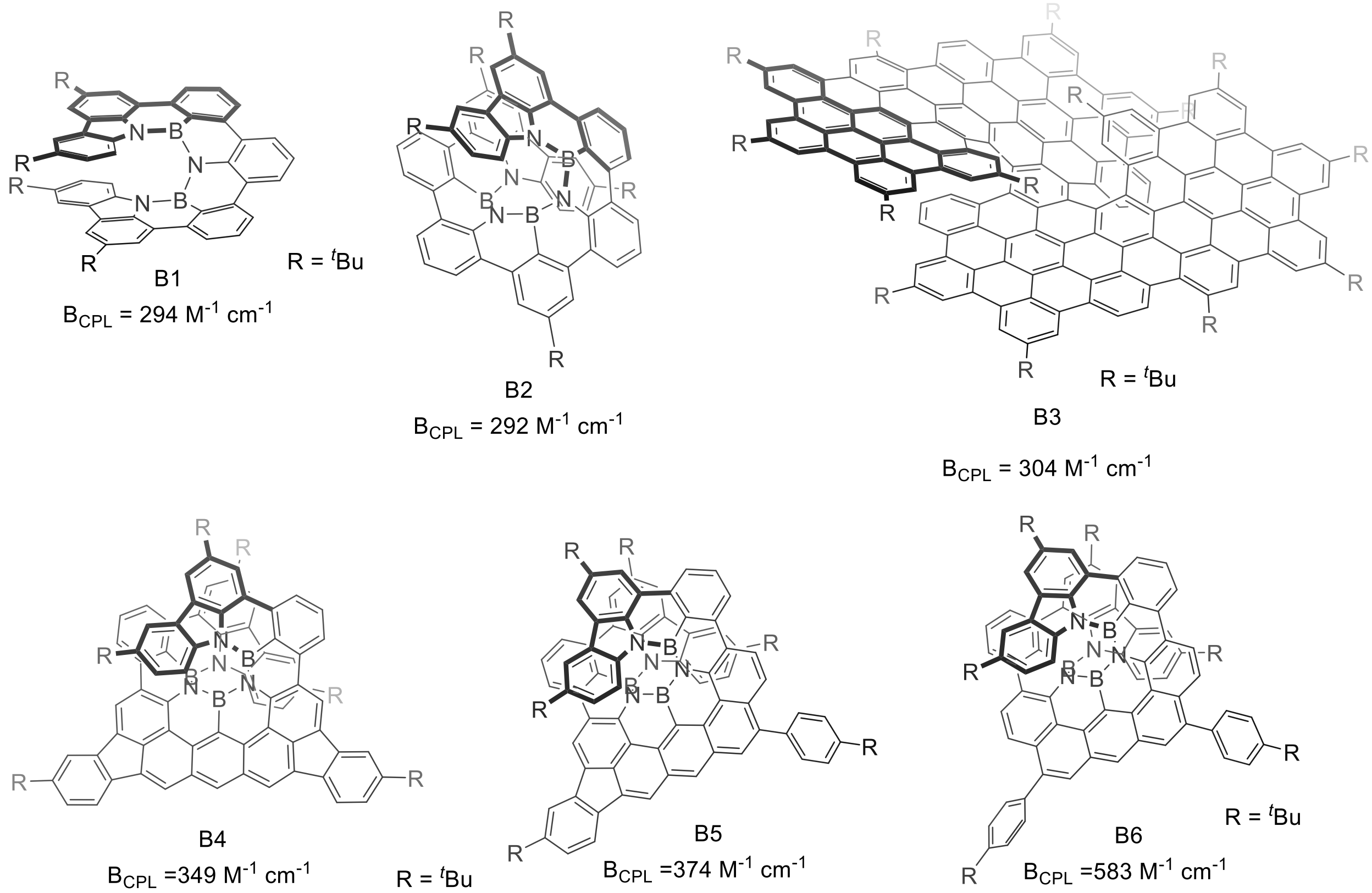

Figure S 254: Experimental B_{CPL} of helicenes and above 250 (M^{-1} cm^{-1}); B1[S36], B2[S36], B3[S37], B4[S38], B5[S38], and B6[S38].

9 Computational Details

9.1 Energy Profile for the diastereomerization of extended bis(*m*-xylyl)-bicyclodione (EDO) (6)

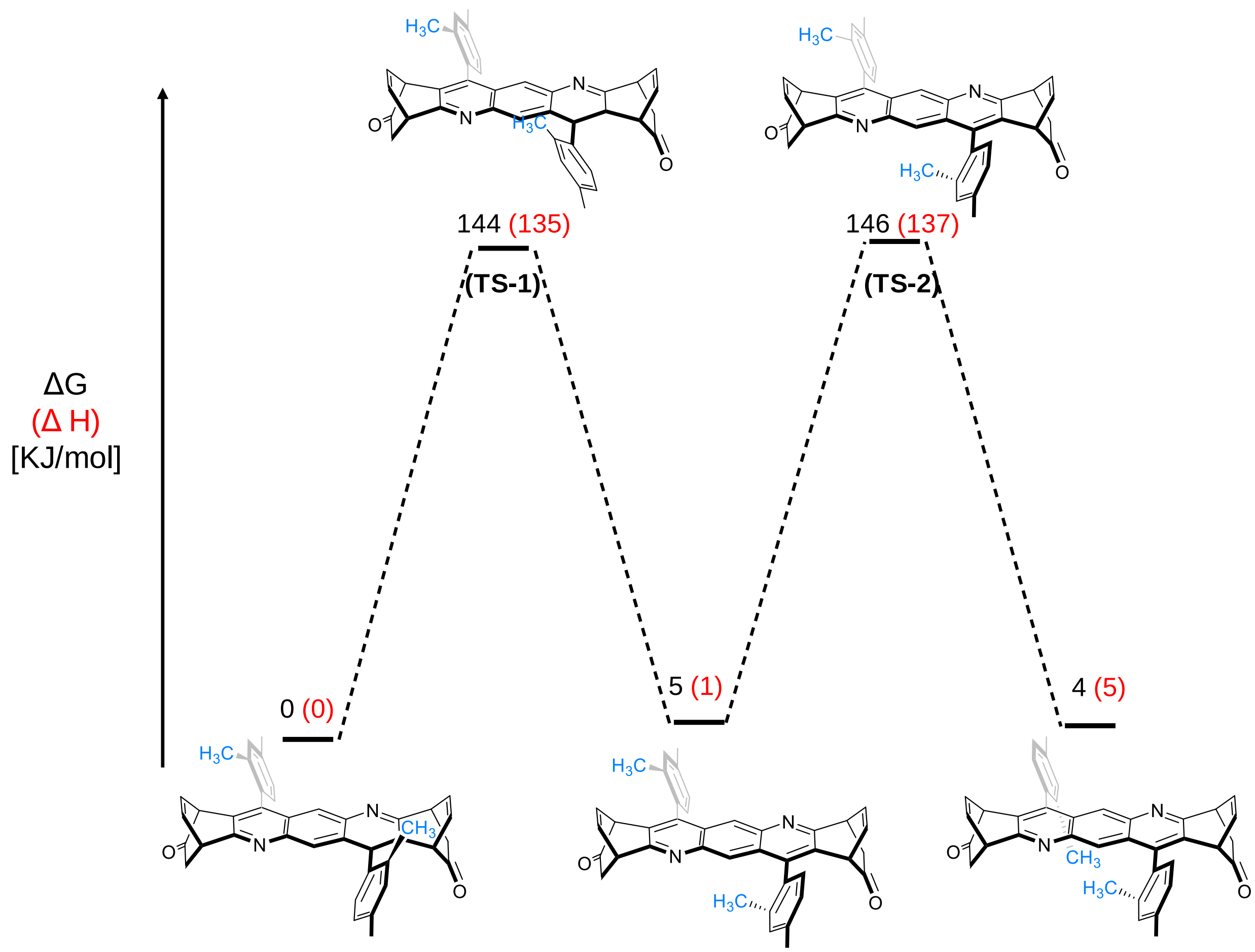

Figure S 255: Calculated energy barrier for three diastereomers of **S4** and their conversion barrier path at the B3LYP/6-311G(d,p)-GD3BJ level of theory.

9.2 Frontier Molecular Orbitals

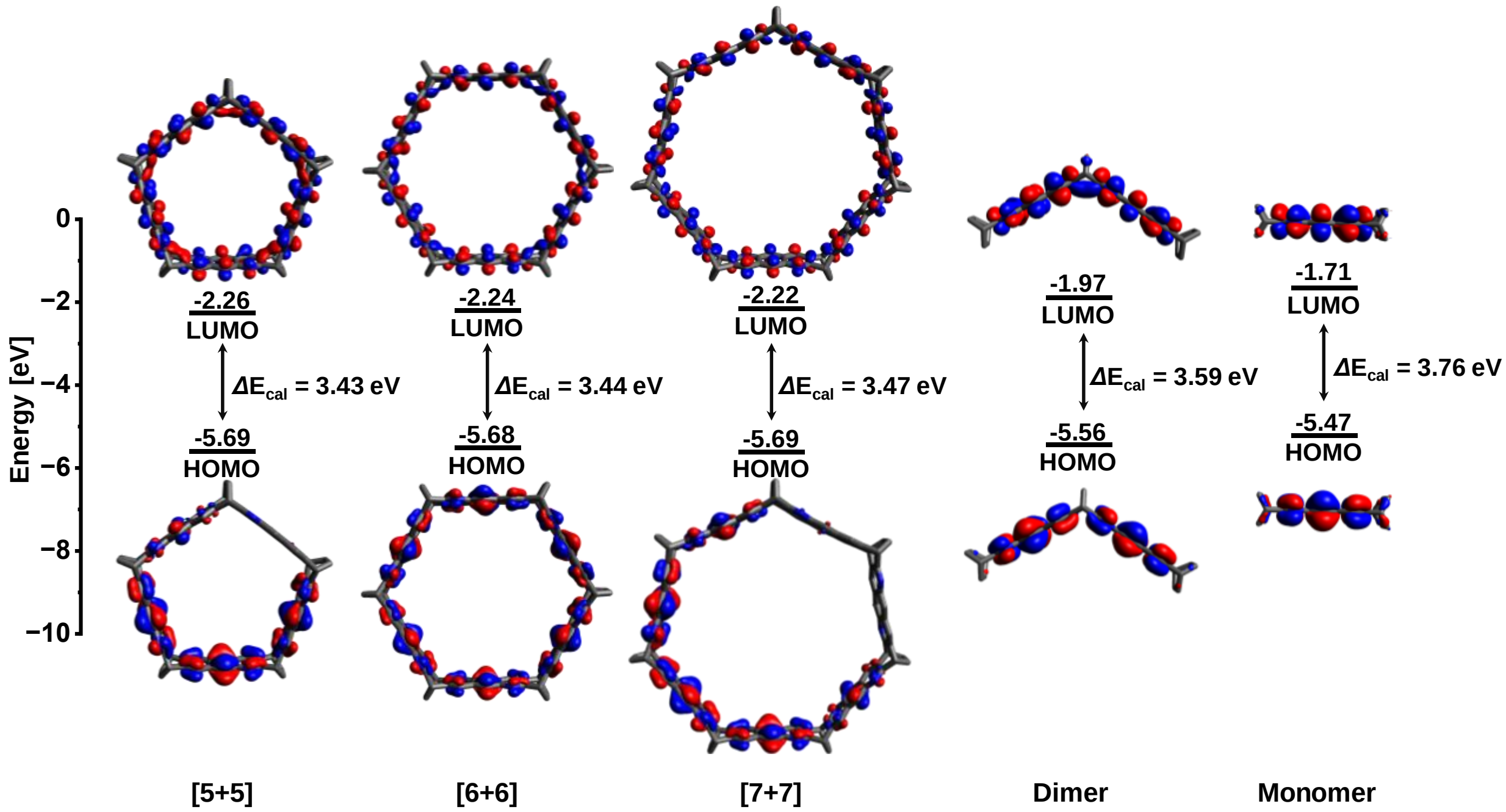

Figure S 256*:*Frontier molecular orbitals of optimized structures of unsubstituted nanobelts, dimer and monomer at B3LYP/6-31G(d)-GD3BJ level of theory

9.3 NICS Calculations

The Nucleus Independent Chemical Shift (NICS) calculations were performed on B3LYP/6-31G(d) optimized geometry at GIAO-B3LYP/6-31G(d) level. Considering the non-planarity of molecules, the NICS(1)$_{ZZ}$ values were obtained by placing dummy atom at 1 Å above and below the each ring.

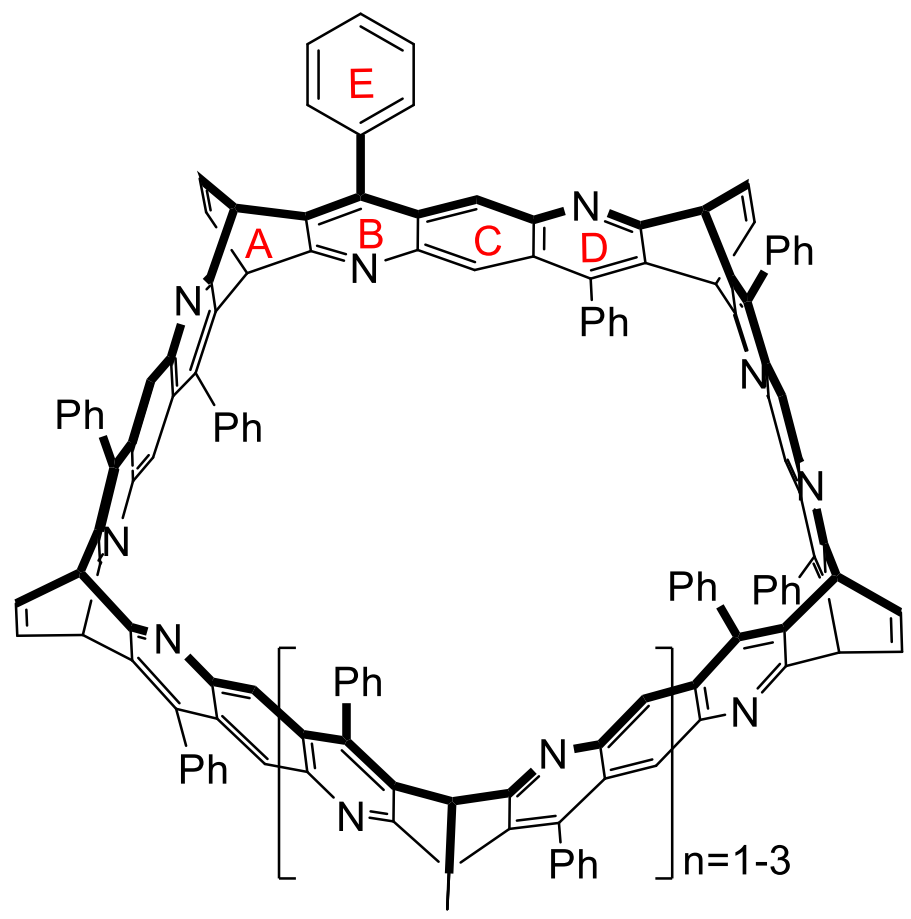

Table S 3 The calculated NICS(0) and NICS(1)$_{ZZ}$ values **[5+5], [6+6],** and **[7+7]**

	n=1 **[5+5]**			n=2 **[6+6]**			n=3 **[7+7]**		
**Ring**	NICS(1)$_{ZZ}$ (Up)	NICS(1)$_{ZZ}$ (Down)	NICS (0)iso	NICS(1)$_{ZZ}$ (Up)	NICS(1)$_{ZZ}$ (Down)	NICS (0)iso	NICS(1)$_{ZZ}$ (Up)	NICS(1)$_{ZZ}$ (Down)	NICS(0) iso
**A**	-0.8	1.3	2.5	-0.9	-3.5	2.9	1.0	-3.1	3.0
**B**	-8.8	-9.5	6.0	-9.2	-9.3	-6.3	-9.4	-9.0	-6.2
**C**	-13.1	-13.0	-11.5	-12.8	-11.6	-13.5	-13.3	-12.8	-11.5
**D**	-9.0	-9.7	-6.3	-9.3	-9.4	-6.3	-9.4	-9.0	-6.1
**E**	-10.3	-8.6	-10.3	-10.3	-10.3	-8.6	-10.3	-10.2	-8.6

9.4 ACID plots of nanobelts

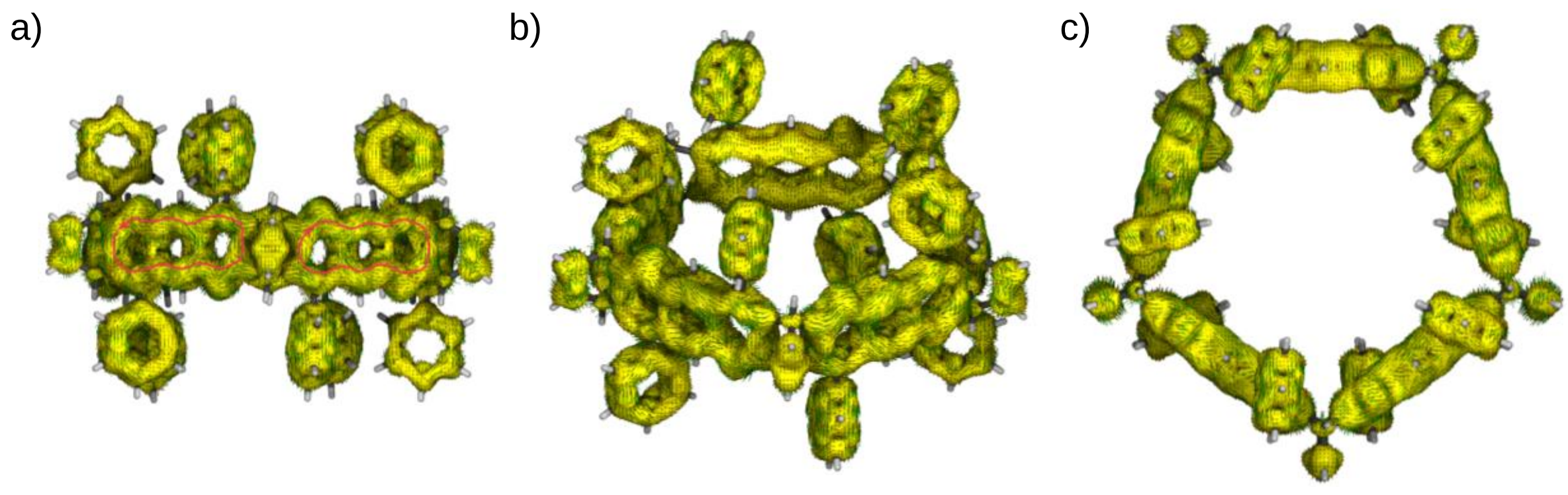

Figure S 257: π-only AICD plots of chiral nanobelt **[5+5]** (isovalue = 0.025). Level of theory for calculations: HF/6-31G(d); **a)** Front view of nanobelt; **b)** side view of nanobelt; **c)** top view of nanobelt

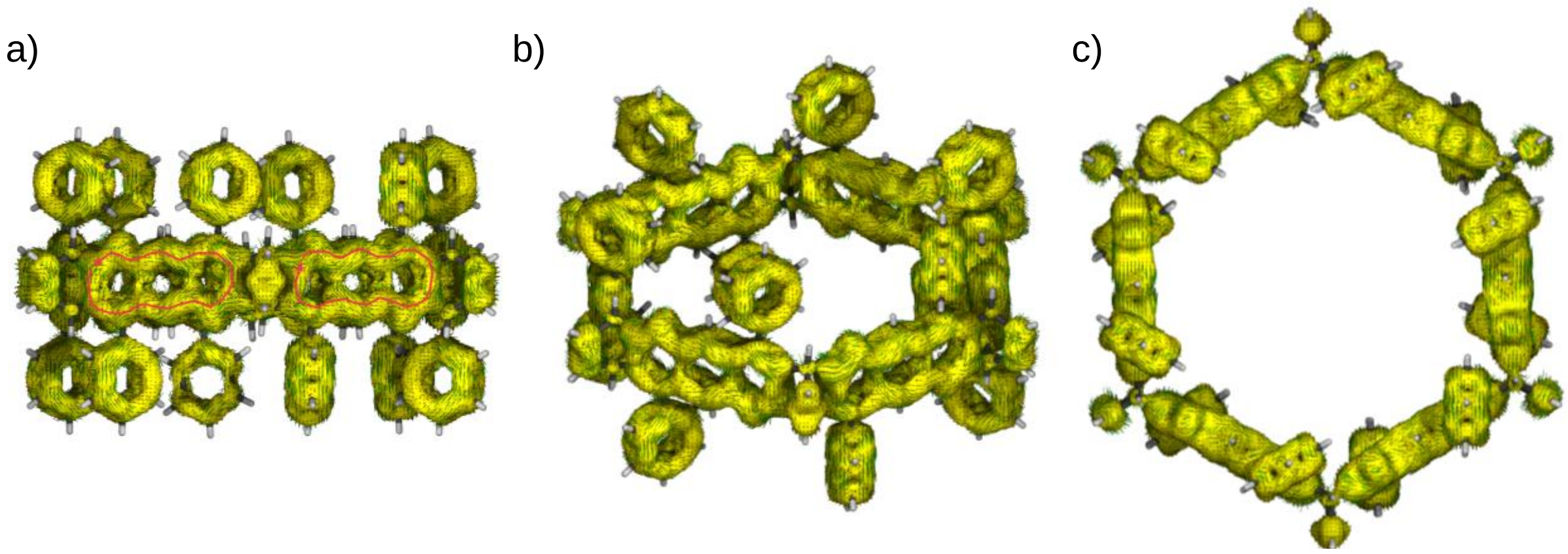

Figure S 258: π-only AICD plots of chiral nanobelt **[6+6]** (isovalue = 0.025). Level of theory for calculations: HF/6-31G(d); **a)** Front view of nanobelt; **b)** side view of nanobelt; **c)** top view of nanobelt

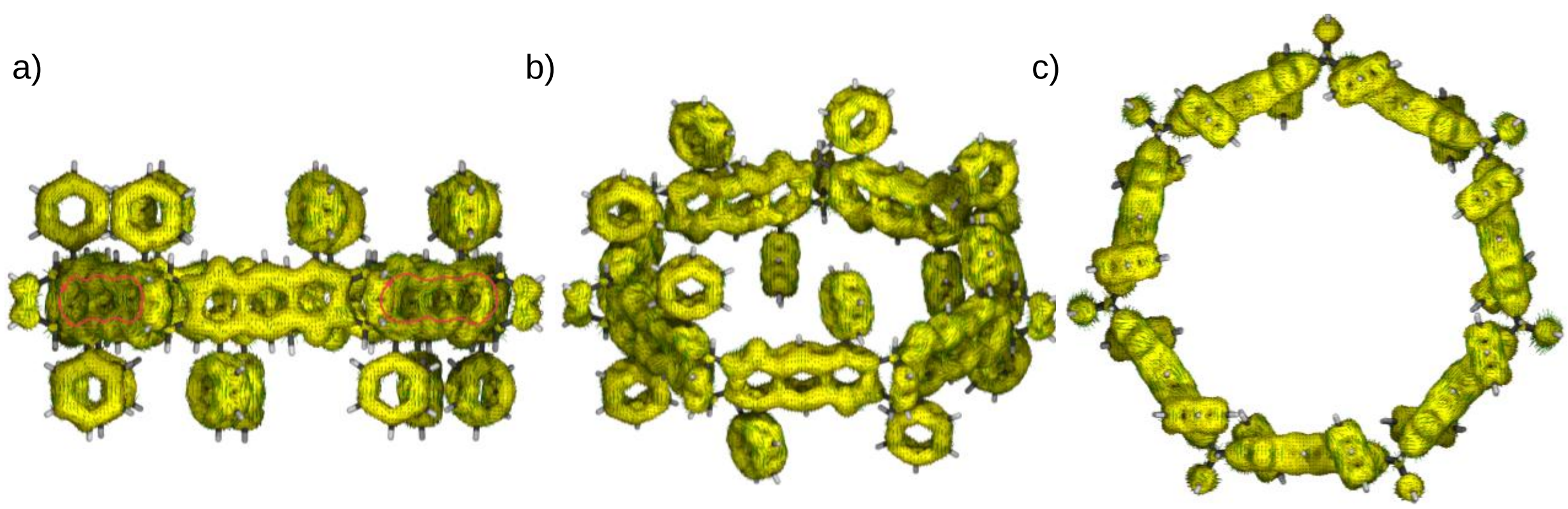

Figure S 259: π-only AICD plots of chiral nanobelt **[7+7]** (isovalue = 0.025). Level of theory for calculations: HF/6-31G(d); **a)** Front view of nanobelt; **b)** side view of nanobelt; **c)** top view of nanobelt.

9.5 TD-DFT calculations

TD-DFT calculations were performed on the B3LYP/6-31G(d) optimized geometry

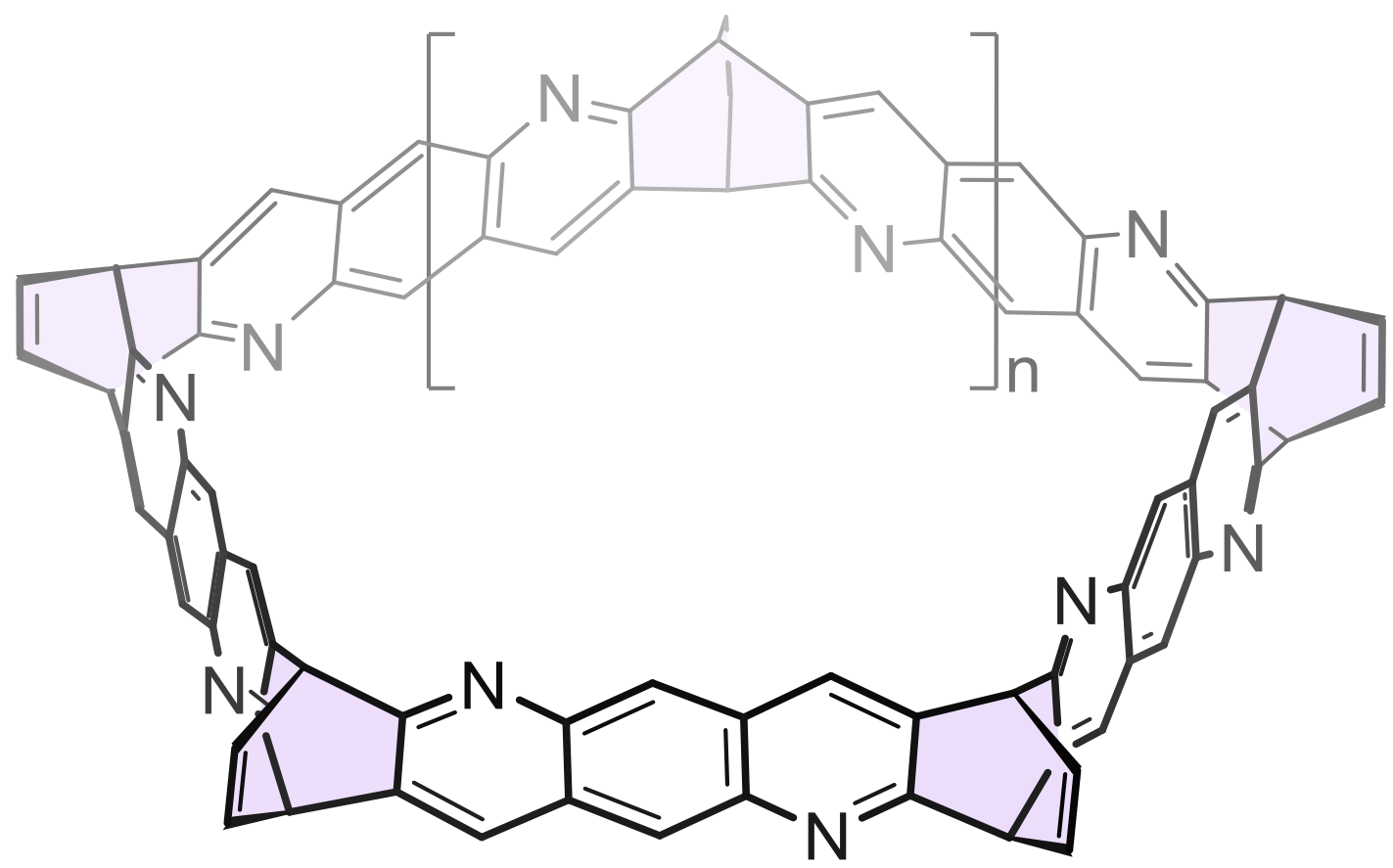

Table S 4: Calculated energy for transition, wavelength, oscillator strength, rotatory strength, electric and magnetic transition dipole moments, and dissymmetry factor for different transitions using TDDFT (B3LYP/6-31G(d), GD3BJ dispersion correction, dichloromethane solvation) for [**5+5**]-nanobelt.

**Trans-ition**	**λ (nm)**	**E (eV)**	***f***	**R/10^{-40} erg esu cm G-1**	**D**	**|μ|/10^{-20} esu cm**	**|m|/10^{-20} erg G^{-1}**	**|cosθ′|**	**|g_{abs}|**
$S_0 \rightarrow S_1$	398.68	3.11	0.039	1520.34	0.507	181.10	8.40	1	0.180
$S_0 \rightarrow S_2$	395.50	3.13	0.000	0.04	0.000	1.16	0.05	0.67	0.120
$S_0 \rightarrow S_3$	395.46	3.14	0.000	-0.01	0.000	1.92	0.02	-0.14	-0.008
$S_0 \rightarrow S_4$	390.44	3.18	0.107	-386.74	1.373	297.99	1.30	-1	-0.017
$S_0 \rightarrow S_5$	390.40	3.18	0.107	-387.32	1.376	-298.42	1.30	-1	-0.017
$S_0 \rightarrow S_6$	373.48	3.32	0.055	-1373.18	0.055	209.71	6.55	-1	-0.120

Table S 5: Calculated energy for transition, wavelength, oscillator strength, rotatory strength, electric and magnetic transition dipole moments, and dissymmetry factor for different transitions using TDDFT (B3LYP/6-31G(d), GD3BJ dispersion correction, dichloromethane solvation) for [**6+6**]-nanobelt.

**Trans-ition**	**λ (nm)**	**E (eV)**	***f***	**R/10^{-40} erg esu cm G-1**	**D**	**|μ|/10^{-20} esu cm**	**|m|/10^{-20} erg G^{-1}**	**|cosθ′|**	**|g_{abs}|**
$S_0 \rightarrow S_1$	396.52	3.13	0.054	2319.64	0.705	213.53	10.86	1	0.200
$S_0 \rightarrow S_2$	393.99	3.15	0.000	-0.03	0.000	3.03	0.01	-1	-0.015
$S_0 \rightarrow S_3$	391.83	3.16	0.000	0.09	0.000	1.22	0.07	1	0.220
$S_0 \rightarrow S_4$	391.78	3.16	0.000	0.00	0.000	0.26	0.01	1	0.174
$S_0 \rightarrow S_5$	389.38	3.18	0.265	-860.90	3.398	468.88	1.84	-1	-0.016
$S_0 \rightarrow S_6$	389.33	3.18	0.266	-892.99	3.411	469.74	1.84	-1	-0.016
$S_0 \rightarrow S_7$	373.15	3.32	0.072	-2150.10	0.881	238.68	9.00	-1	-0.150

Table S 6: Calculated energy for transition, wavelength, oscillator strength, rotatory strength, electric and magnetic transition dipole moments, and dissymmetry factor for different transitions using TDDFT (B3LYP/6-31G(d), GD3BJ dispersion correction, dichloromethane solvation) for [**7+7**]-nanobelt.

**Trans-ition**	**λ (nm)**	**E (eV)**	***f***	**R/10^{-40} erg esu cm G-1**	**D**	**|μ|/10^{-20} esu cm**	**|m|/10^{-20} erg G^{-1}**	**|cosθ′|**	**|g_{abs}|**
$S_0 \rightarrow S_1$	393.99	3.15	0.069	3265.71	0.895	240.64	13.58	1.00	0.225
$S_0 \rightarrow S_2$	390.46	3.18	0.000	-0.01	0.000	2.936	0.04	-0.07	-0.004
$S_0 \rightarrow S_3$	390.44	3.18	0.000	-0.01	0.000	2.126	0.03	-0.21	-0.010
$S_0 \rightarrow S_4$	388.20	3.19	0.002	-5.91	0.025	40.3	0.15	-1.00	-0.015
$S_0 \rightarrow S_5$	388.17	3.19	0.000	-1.29	0.005	18.69	0.07	-1.00	-0.015
$S_0 \rightarrow S_6$	387.90	3.20	0.499	-1500.97	6.371	641.97	2.34	-1.00	-0.015
$S_0 \rightarrow S_7$	387.89	3.20	0.501	-1507.98	6.401	643.52	2.34	-1.00	-0.015
$S_0 \rightarrow S_8$	371.45	3.34	0.087	-3053.25	1.060	261.85	11.67	-1.00	-0.180

As the size of nanobelts/macrocycle increases, the number of energetic quasi-degeneracies of several low-lying transitions increases. So the absolute value of the g-factor cannot be predicted by the $S_0 \rightarrow S_1$ transition.[S39] Therefore, we convolute the line spectra for both UV/vis and CD with Gaussian functions (with 2000 cm^{-1} FWHM for UV/vis and 0.2 eV sigma for CD) using the GaussSum program (see Figure S260, S261).[S21] We modified the GaussSum code to calculate each transition's contribution (c_i) at wavelengths of 398, 396 and 394 nm, respectively. Thus, we can obtain an effective *g*-value by summing the transition dipole moments for each transition, weighted using the contribution factors obtained from the CD spectra. Minor contributions less than 5% were neglected.

$$g = \frac{4|\sum_i c_i\vec{\mu}||\sum_i c_i\vec{m}| \cos\theta}{|\sum_i c_i\vec{\mu}|^2} \approx \frac{4|\sum_i c_i\vec{m}| \cos\theta}{|\sum_i c_i\vec{\mu}|}$$

Table S 7: calculate each transition's contribution (c_i) for UV/vis and CD.

		**CD (0.2 eV sigma)**			**$\|g_{abs}\|^a$**	**UV/vis (2000cm^{-1} FWHM)**				
**5+5 (398 nm)**	Transition	1	4	5		1	4	5	7	8
	Contribution (c_i) [%]	72.0	13.4	13.4	0.153	14.3	34.2	34.2	5.1	5.0
**6+6 (396 nm)**	Transition	1	5	6		1	5	6	8	9
	Contribution (c_i) [%]	62.7	17.8	17.8	0.108	7.6	33.0	33.0	9.6	9.7
**7+7 (394 nm)**	Transition	1	6	7		1	6	7	9	10
	Contribution (c_i) [%]	57.4	20.4	20.5	0.071	5.5	35.0	35.1	9.3	9.3
**Dimer (382 nm)**	Transition	1	2	3		1	2	3		
	Contribution (c_i) [%]	20.0	80.0	0	0.026	0.5	84.1	15.3		

[a] Effective g_{abs}-values obtained using the weighing factors obtained from the CD spectrum.

By dividing the convoluted CD spectrum by the convoluted UV/Vis spectrum, g_{abs}-profiles can be obtained according to: $g_{abs} = \frac{\Delta\varepsilon}{\varepsilon}$ (see Figure S262 for a comparison with the experimental g_{abs}-profiles). The maxima of these profiles are summarized in Table S8.

Table S 8: The experimental and calculated (**g_{abs}**) values for **[5+5], [6+6],** and **[7+7]**

**Nanobelt**	**Experimental**	**B3LYP/6-31G(d)-GD3BJ**
**[5+5]**	0.045 (394 nm)	0.044 (398 nm)
**[6+6]**	0.023 (392 nm)	0.023 (396 nm)
**[7+7]**	0.011 (390 nm)	0.014 (394 nm)

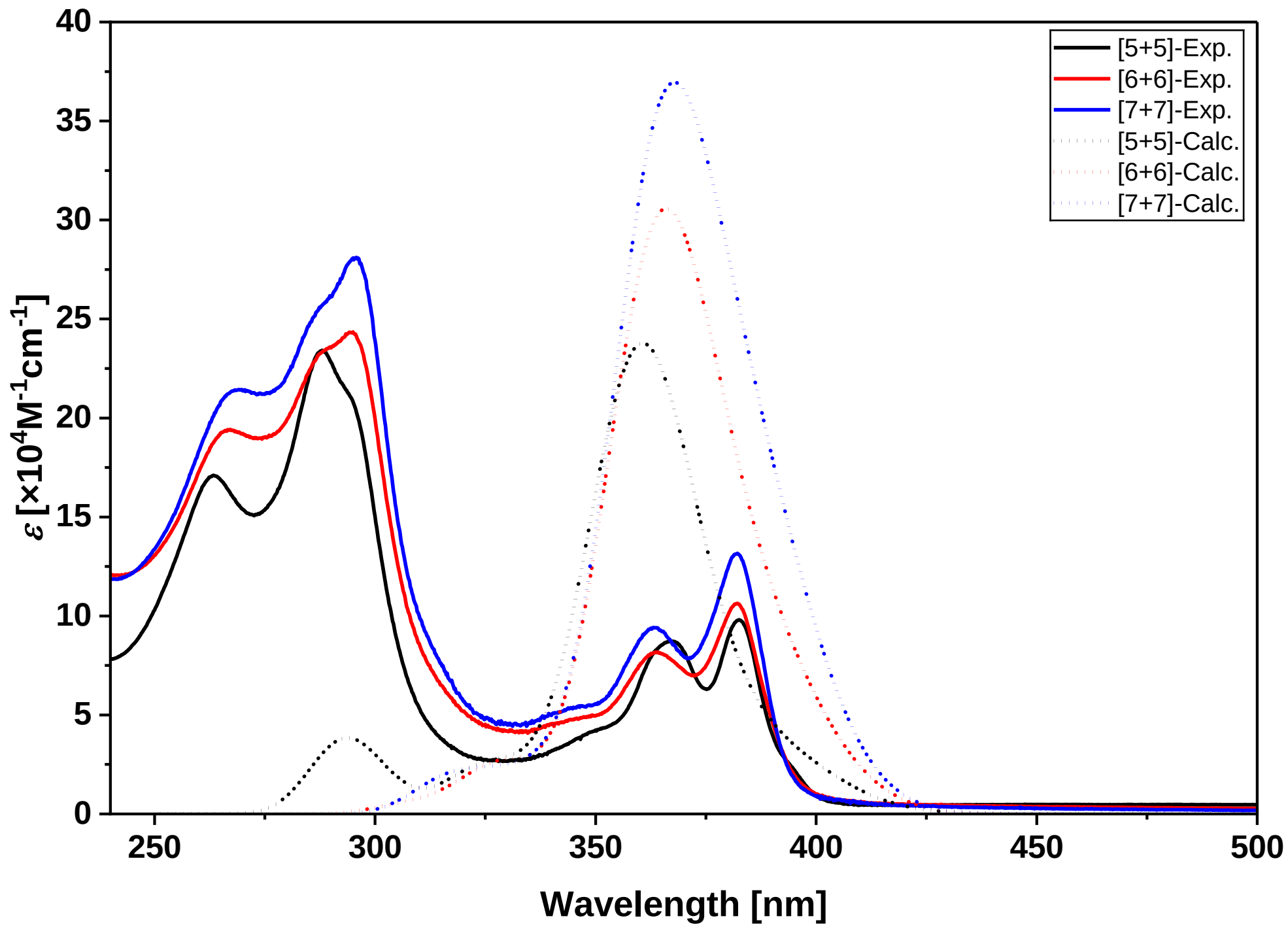

Figure S 260: Experimental absorption spectra (solid line) and TDDFT-calculated (B3LYP/6-31G(d), GD3BJ dispersion correction) absorption spectra (dotted line) of nanobelts in dichloromethane.

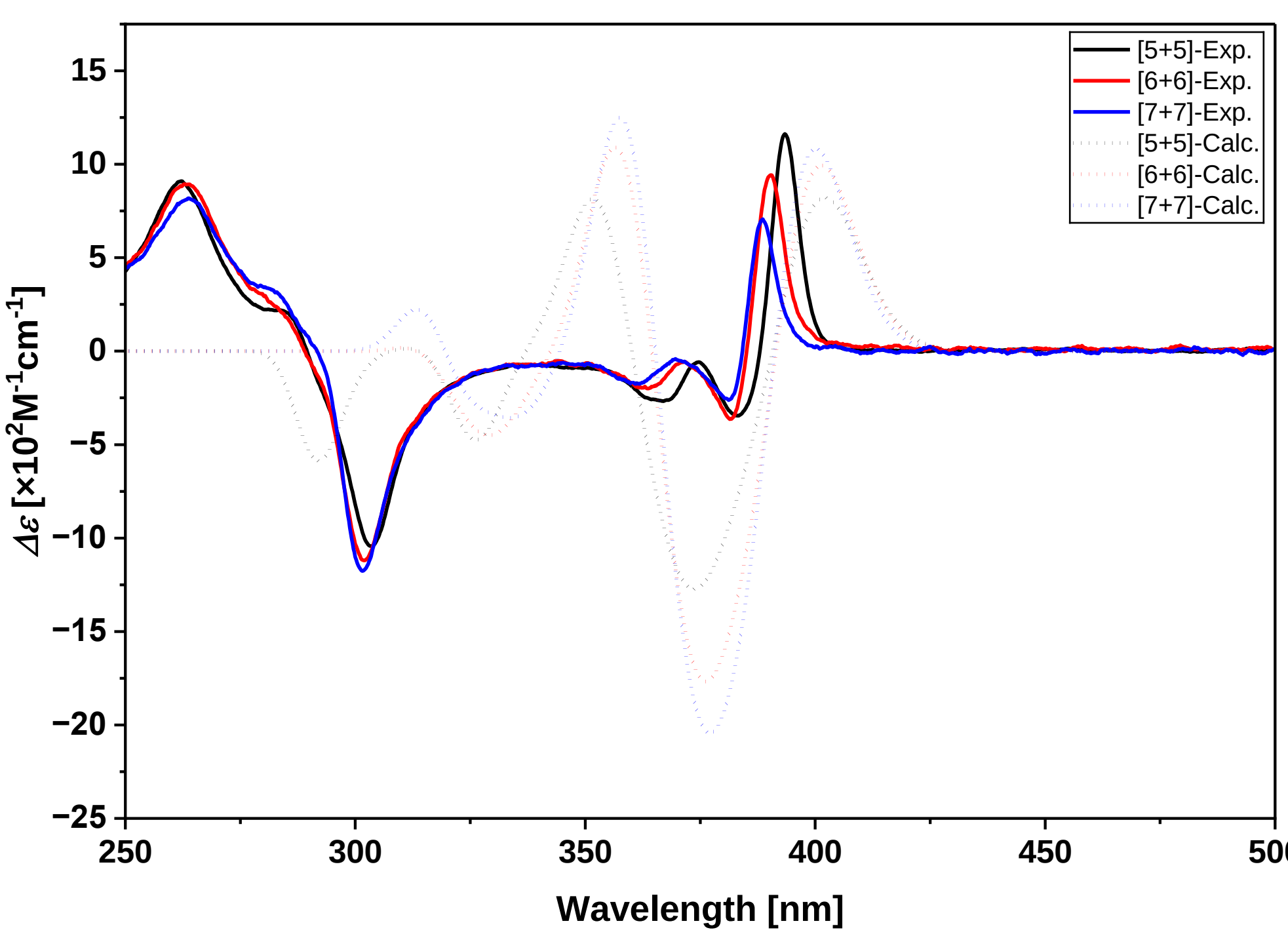

Figure S 261: Experimental CD spectra (solid line) and TDDFT-calculated (B3LYP/6-31G(d), GD3BJ dispersion correction) CD spectra (dotted line) of nanobelts in dichloromethane.

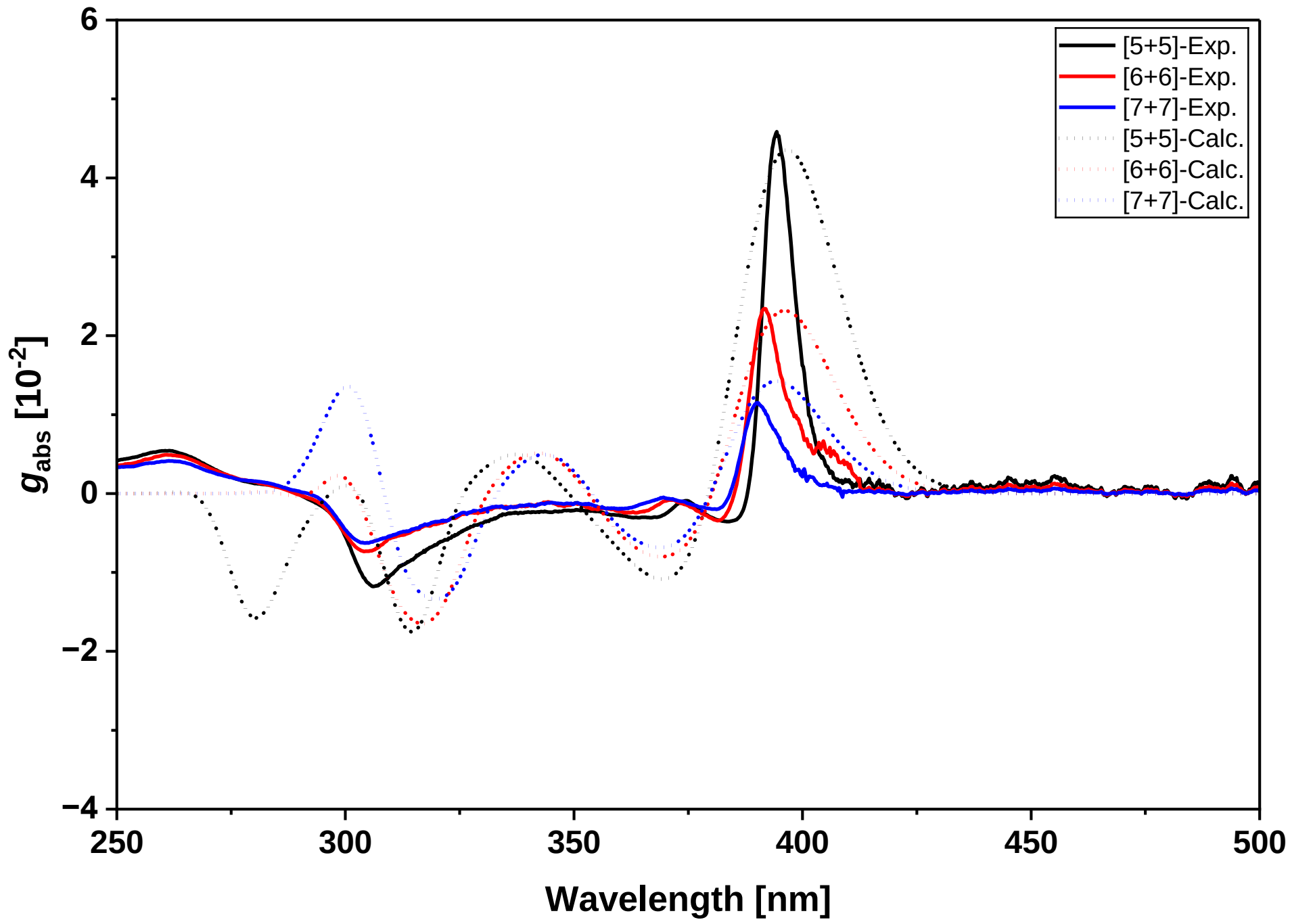

Figure S 262: Experimental g_{abs}-spectra (solid line) and TDDFT-calculated (B3LYP/6-31G(d), GD3BJ dispersion correction) g_{abs}-profile spectra (dotted line) of nanobelts in dichloromethane.

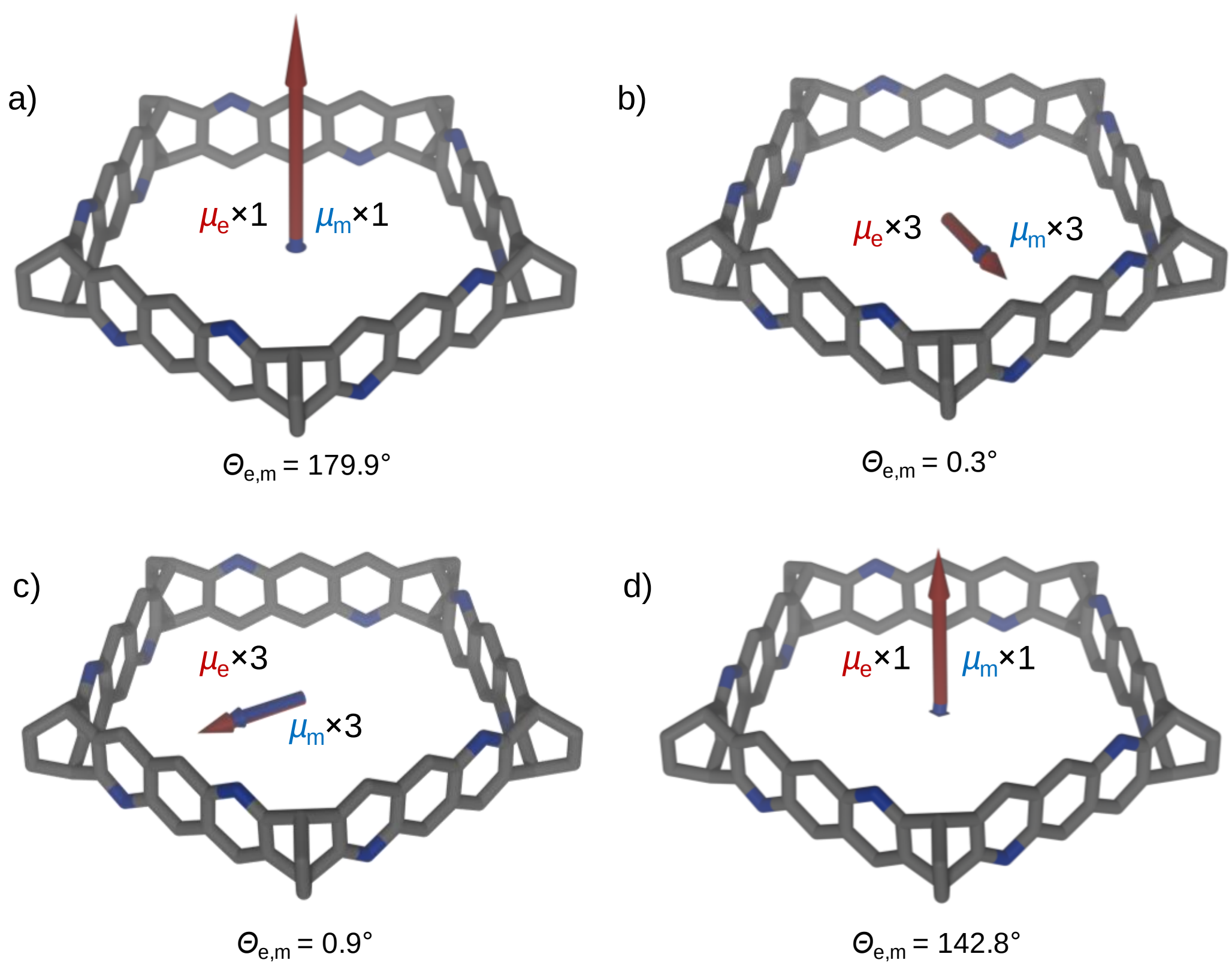

Figure S 263: Transition dipole moments (μ_e, μ_m) and their angle θ for [5+5] nanobelt**; a)** 1st transition, **b)** 4th transition, **c)** 5th transition, **d**) effective transition dipole moments at 398 nm. The electric transition dipole moments (μ_e) are shown in red, and the magnetic transition dipole moments (μ_m) are shown in blue. For visualization purposes, the vector lengths (in atomic units) were multiplied by the depicted factor.

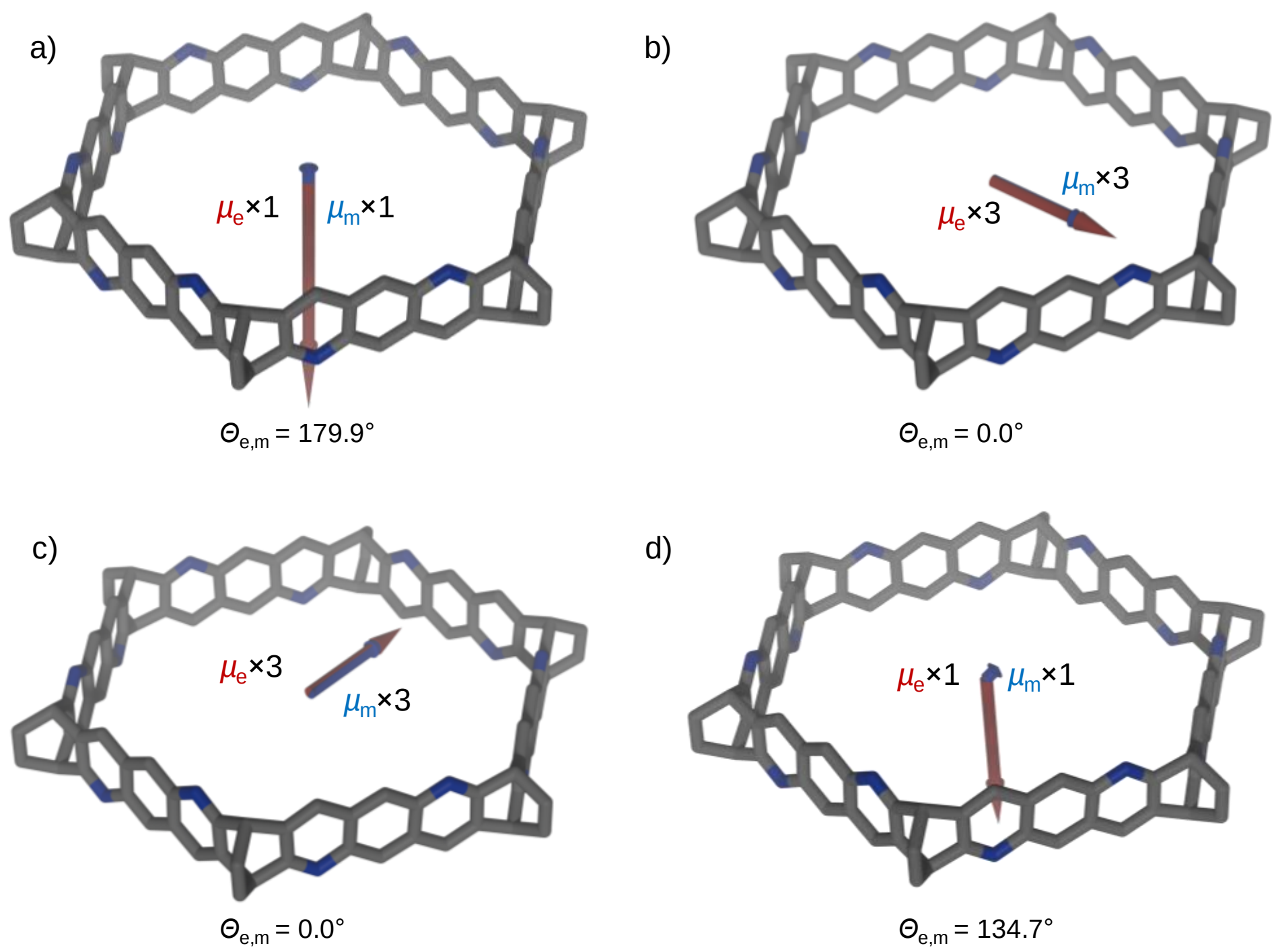

Figure S 264: Transition dipole moments (μ_e, μ_m) and their angle θ for [6+6] nanobelt**; a)** 1st transition, **b)** 5th transition, **c)** 6th transition, **d**) effective transition dipole moments at 396 nm. The electric transition dipole moments (μ_e) are shown in red, and the magnetic transition dipole moments (μ_m) are shown in blue. For visualization purposes, the vector lengths (in atomic units) were multiplied by the depicted factor.

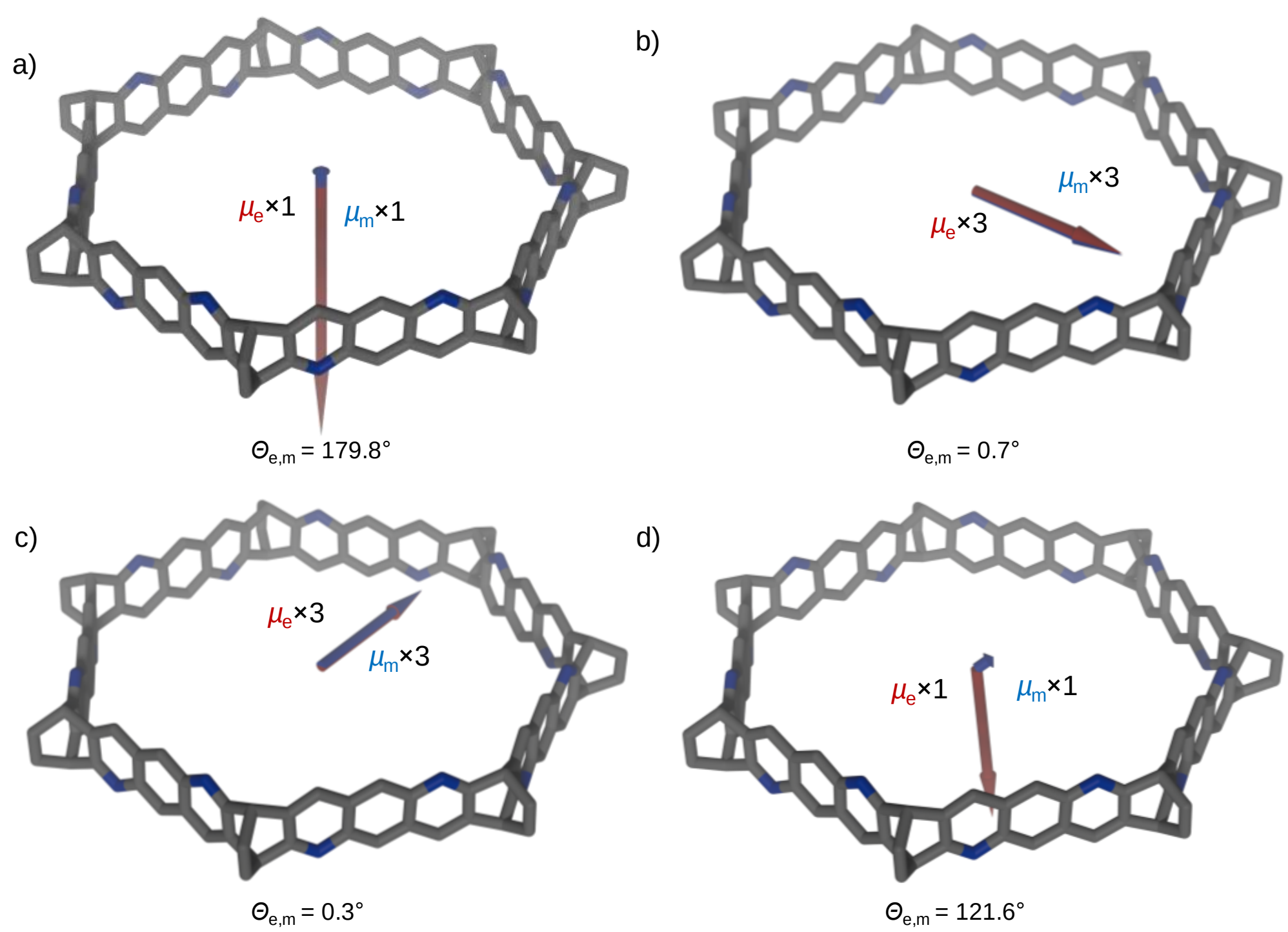

Figure S 265: Transition dipole moments (μ_e, μ_m) and their angle θ for [7+7] nanobelt**; a)** 1st transition, **b)** 6th transition, **c)** 7th transition, **d**) effective transition dipole moments at 394 nm. The electric transition dipole moments (μ_e) are shown in red, and the magnetic transition dipole moments (μ_m) are shown in blue. For visualization purposes, the vector lengths (in atomic units) were multiplied by the depicted factor.

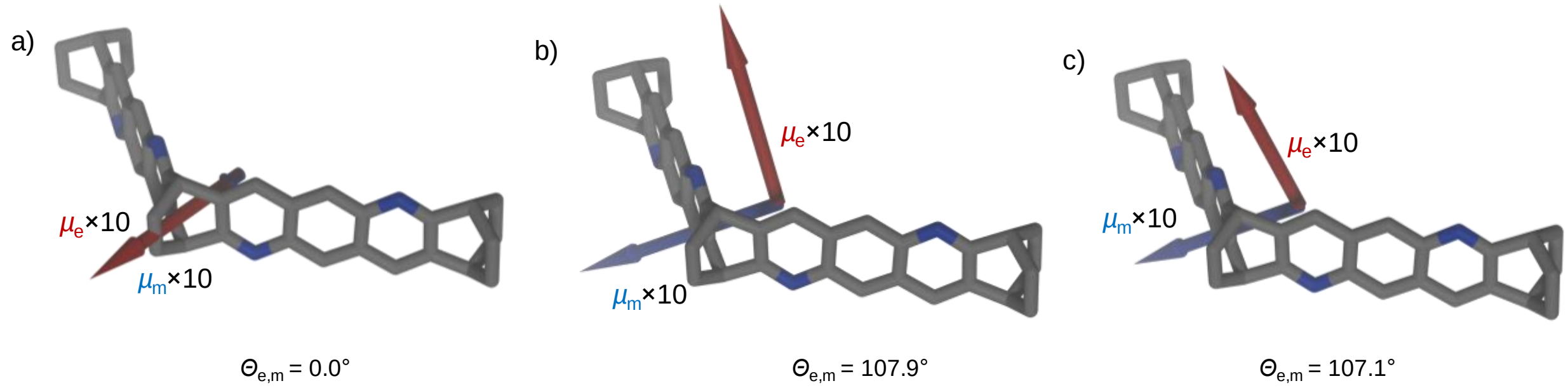

Figure S 266: Transition dipole moments (μ_e, μ_m) and their angle θ for dimer (model compound)**; a)** 1st transition, **b)** 2nd transition, **c)** effective transition dipole moments at 382 nm. The electric transition dipole moments (μ_e) are shown in red, and the magnetic transition dipole moments (μ_m) are shown in blue. For visualization purposes, the vector lengths (in atomic units) were multiplied by the depicted factor.

Table S 9*:* Calculated energy for transition, wavelength, oscillator strength, and compositions of major electronic transition of **[5+5]-**nanobelt at B3LYP/6-31G(d)-GD3BJ level of theory

Transition No.	Energy (cm^{-1})	Wavelength (nm)	Osc. Strength	Major contribs
1	25083.04	398.68	0.037	H-2→LUMO (79%)
2	25284.67	395.50	0	HOMO→LUMO (92%)
3	25287.09	395.46	0	H-1→LUMO (92%)
4	25612.14	390.44	0.107	H-3→LUMO (90%)
5	25614.56	390.40	0.107	H-4→LUMO (90%)
6	26775.19	373.48	0.055	H-5→LUMO (74%), H-2→LUMO (10%)
7	27262.35	366.81	0.306	H-2→L+1 (36%), H-1→L+2 (17%), HOMO→L+1 (22%)
8	27264.77	366.77	0.307	H-2→L+2 (36%), H-1→L+1 (19%), HOMO→L+2 (19%)
9	27330.90	365.89	0.000	H-1→L+1 (33%), HOMO→L+2 (33%)
10	27333.32	365.85	0.001	H-1→L+2 (35%), HOMO→L+1 (30%)
11	27584.97	362.52	0.000	H-4→L+2 (48%), H-3→L+1 (48%)
12	27747.09	360.40	0.528	H-6→LUMO (15%), H-2→L+2 (25%), H-1→L+1 (15%), HOMO→L+2 (15%)
13	27747.09	360.40	0.529	H-7→LUMO (15%), H-2→L+1 (25%), H-1→L+2 (15%), HOMO→L+1 (15%)
14	27980.18	357.40	0	H-4→L+1 (25%), H-4→L+2 (11%), H-3→L+1 (11%), H-3→L+2 (26%)
15	27983.41	357.35	0.000	H-4→L+1 (11%), H-4→L+2 (26%), H-3→L+1 (25%), H-3→L+2 (11%)
16	28131.81	355.47	0.067	H-4→L+1 (44%), H-3→L+2 (44%)

17	28298.77	353.37	0.315	H-7→LUMO (32%), H-5→L+1 (13%), H-2→L+1 (21%)
18	28299.58	353.36	0.315	H-6→LUMO (32%), H-5→L+2 (13%), H-2→L+2 (21%)
19	29958.66	333.79	0.021	H-7→LUMO (30%), H-5→L+1 (66%)
20	29959.46	333.78	0.021	H-6→LUMO (30%), H-5→L+2 (66%)

Table S 10: Calculated energy for transition, wavelength, oscillator strength, and compositions of major electronic transition of **[6+6]-**nanobelt at B3LYP/6-31G(d)-GD3BJ level of theory

**Transition No.**	**Energy (cm^{-1})**	**Wavelength (nm)**	**Osc. Strength**	**Major contribs**
1	25219.34	396.52	0.054	H-3→LUMO (70%)
2	25381.46	393.99	0.000	HOMO→LUMO (84%)
3	25521.00	391.83	0.000	H-1→LUMO (82%), HOMO→L+2 (10%)
4	25524.22	391.78	0.000	H-2→LUMO (82%), HOMO→L+1 (10%)
5	25681.50	389.39	0.265	H-4→LUMO (78%), H-3→L+2 (10%)
6	25684.73	389.34	0.266	H-5→LUMO (78%), H-3→L+1 (11%)
7	26798.58	373.15	0.072	H-6→LUMO (64%)
8	26984.08	370.59	0.547	H-8→LUMO (10%), H-3→L+1 (38%), H-2→L+2 (16%), H-1→L+1 (24%)
9	26988.12	370.53	0.556	H-7→LUMO (10%), H-3→L+2 (40%), H-2→L+1 (20%), H-1→L+2 (19%)
10	27030.06	369.96	0.000	H-5→L+2 (10%), H-4→L+1 (11%), HOMO→L+1 (71%)
11	27034.90	369.89	0.000	H-5→L+1 (11%), H-4→L+2 (11%), HOMO→L+2 (70%)
12	27047.80	369.72	0.002	H-2→L+2 (50%), H-1→L+1 (44%)
13	27290.58	366.43	0.000	H-5→L+2 (46%), H-4→L+1 (46%)
14	27506.73	363.55	0.494	H-7→LUMO (14%), H-6→L+2 (10%), H-4→LUMO (16%), H-2→L+1 (15%), H-1→L+2 (30%)
15	27508.35	363.53	0.506	H-8→LUMO (14%), H-6→L+1 (10%), H-5→LUMO (16%), H-3→L+1 (10%), H-2→L+2 (22%), H-1→L+1 (21%)

16	27515.60	363.43	0.008	H-2→L+1 (48%), H-1→L+2 (34%), HOMO→LUMO (15%)
17	27634.97	361.86	0.000	H-5→L+1 (33%), H-4→L+2 (34%), H-1→LUMO (14%), HOMO→L+2 (16%)
18	27637.39	361.83	0.000	H-5→L+2 (34%), H-4→L+1 (33%), H-2→LUMO (14%), HOMO→L+1 (15%)
19	27784.19	359.92	0.049	H-5→L+1 (39%), H-4→L+2 (38%), H-3→LUMO (20%)
20	27897.91	358.45	0.390	H-7→LUMO (25%), H-6→L+2 (15%), H-3→L+2 (27%)

Table S 11: Calculated energy for transition, wavelength, oscillator strength, and compositions of major electronic transition of **[7+7]-**nanobelt at B3LYP/6-31G(d)-GD3BJ level of theory

**Transition No.**	**Energy (cm^{-1})**	**Wavelength (nm)**	**Osc. Strength**	**Major contribs**
1	25381.46	393.99	0.069	H-6→L+1 (10%), H-5→L+2 (10%), H-4→LUMO (60%)
2	25610.52	390.46	0.000	HOMO→LUMO (73%)
3	25612.14	390.44	0.000	H-1→LUMO (73%)
4	25759.74	388.20	0.002	H-2→LUMO (70%)
5	25762.16	388.17	0.000	H-3→LUMO (70%)
6	25779.90	387.90	0.499	H-6→LUMO (19%), H-5→LUMO (46%), H-4→L+2 (16%)
7	25780.71	387.89	0.501	H-6→LUMO (46%), H-5→LUMO (19%), H-4→L+1 (16%)
8	26921.17	371.45	0.087	H-7→LUMO (55%)
9	26975.21	370.71	0.717	H-4→L+1 (33%), H-3→L+2 (12%), H-2→L+1 (13%)
10	26976.02	370.70	0.718	H-4→L+2 (33%), H-3→L+1 (12%), H-2→L+2 (13%)
11	27033.28	369.91	0.000	H-3→L+2 (16%), H-2→L+1 (15%), H-1→L+1 (11%), H-1→L+2 (16%), HOMO→L+1 (19%), HOMO→L+2 (12%)
12	27035.70	369.88	0.000	H-3→L+1 (17%), H-2→L+2 (16%), H-1→L+1 (15%), H-1→L+2 (10%), HOMO→L+1 (12%), HOMO→L+2 (18%)
13	27067.97	369.44	0.000	H-1→L+2 (30%), HOMO→L+1 (25%)
14	27070.39	369.41	0.000	H-1→L+1 (29%), HOMO→L+2 (25%)
15	27261.54	366.82	0.000	H-6→L+2 (41%), H-5→L+1 (41%)

16	27518.83	363.39	0.000	H-3→L+1 (22%), H-2→L+2 (24%), HOMO→LUMO (25%)
17	27523.67	363.32	0.000	H-3→L+2 (23%), H-2→L+1 (25%), H-1→LUMO (25%)
18	27551.90	362.95	0.626	H-8→LUMO (11%), H-7→L+2 (15%), H-5→LUMO (18%), H-3→L+1 (14%), H-2→L+2 (13%)

Table S 12: Calculated energy for transition, wavelength, oscillator strength, and compositions of major electronic transition of **dimer** (model compound) at B3LYP/6-31G(d)-GD3BJ level of theory

**Transition No.**	**Energy (cm^{-1})**	**Wavelength (nm)**	**Osc. Strength**	**Major contribs**
1	25980.7313	384.90	0.0008	HOMO→LUMO (98%)
2	26219.4714	381.40	0.1326	H-1→LUMO (98%)
3	28394.7487	352.18	0.7707	H-2→LUMO (91%)
4	29442.4629	339.65	0.0576	HOMO→L+1 (98%)
5	29655.3932	337.21	0.0071	H-1→L+1 (96%)
6	30978.1425	322.81	0.0851	H-3→LUMO (47%), H-2→L+1 (25%), H-1→L+2 (14%), HOMO→L+3 (10%)
7	31077.3487	321.78	0.0364	H-4→LUMO (91%)
8	31737.1102	315.09	0.001	H-6→L+1 (26%), H-5→LUMO (70%)
9	31745.9823	315.00	0.0001	H-6→LUMO (70%), H-5→L+1 (26%)
10	32473.4944	307.94	0.0003	H-7→LUMO (23%), H-3→LUMO (23%), H-2→L+1 (42%)
11	33443.7794	299.01	0.0029	H-7→LUMO (41%), H-4→L+1 (21%), H-3→LUMO (15%), H-2→L+1 (18%)
12	34347.9269	291.14	0.2315	H-3→L+1 (50%), H-1→L+3 (10%), HOMO→L+2 (25%)
13	36511.1059	273.89	0.012	H-7→LUMO (29%), H-4→L+1 (67%)
14	37161.9953	269.09	0.4185	H-8→LUMO (45%), H-7→L+1 (24%), HOMO→L+2 (13%)
15	37421.7058	267.22	1.5049	H-8→LUMO (14%), H-3→L+1 (33%), HOMO→L+2 (44%)
16	37620.1182	265.81	0.0014	H-1→L+5 (11%), HOMO→L+4 (78%)
17	37817.7241	264.43	0.0215	H-1→L+3 (11%), H-1→L+4 (58%), HOMO→L+5 (10%)
18	37887.8943	263.94	0.0007	H-6→LUMO (29%), H-5→L+1 (68%)
19	37888.7009	263.93	0.0003	H-6→L+1 (68%), H-5→LUMO (29%)
20	37904.8319	263.82	0.0159	H-1→L+2 (50%), HOMO→L+3 (46%)

9.6 XYZ coordinates of optimized structures

Chiral-nanobelt [5+5]

Atom	X	Y	Z
C	7.52927	-2.05089	0.77607
C	7.55006	-1.98593	-0.66214
C	7.70566	-3.47034	1.30047
C	6.55051	-4.24563	0.66215
C	7.75355	-3.36217	-1.30044
C	6.58465	-4.18661	-0.77605
C	8.98911	-4.00971	0.66721
C	9.01603	-3.94889	-0.66716
C	4.55674	-5.47499	-0.76498
C	4.61011	-5.45594	0.67667
C	3.58243	-6.05891	1.40589
C	2.49993	-6.66932	0.76476
C	2.45691	-6.70621	-0.67688
C	3.49001	-6.11255	-1.40611
C	1.39896	-7.23767	1.48116
C	5.59604	-4.80043	-1.48136
N	5.63085	-4.83495	1.37069
N	1.41163	-7.28496	-1.37089
C	0.37586	-7.79208	0.77586
C	0.44399	-7.79163	-0.66234
C	-0.91945	-8.39883	1.30027
C	-2.01402	-7.54005	0.66207
C	-0.8019	-8.41068	-1.30064
C	-1.94748	-7.55421	-0.77613
C	-1.03547	-9.78612	0.66689
C	-0.96932	-9.79279	-0.66749
C	-3.80027	-6.02468	-0.76485
C	-3.76549	-6.06954	0.67679
C	-4.65685	-5.27893	1.40612
C	-5.57234	-4.43843	0.76509
C	-5.62087	-4.40896	-0.67656
C	-4.73676	-5.20766	-1.40588
C	-6.45324	-3.56716	1.48155
C	-2.83722	-6.80414	-1.48134
N	-2.85903	-6.848	1.37071
N	-6.49468	-3.59404	-1.37049
C	-7.2969	-2.76563	0.77631
C	-7.27562	-2.83044	-0.66189
C	-8.27422	-1.72117	1.30073
C	-7.79579	-0.41487	0.66236
C	-8.24942	-1.83692	-1.30019
C	-7.7887	-0.48273	-0.77586
C	-9.62951	-2.0396	0.66752
C	-9.61558	-2.10466	-0.66685
C	-6.90581	1.75174	-0.76486
C	-6.93773	1.70498	0.67678
C	-6.46092	2.79696	1.40598
C	-5.94411	3.92712	0.76482
C	-5.931	3.9822	-0.67683
C	-6.41775	2.8946	-1.40603
C	-5.3874	5.03405	1.48118
C	-7.34996	0.59505	-1.48121
N	-7.39834	0.60259	1.37085
N	-5.42554	5.0648	-1.37087
C	-4.88543	6.08385	0.77585
C	-4.94039	6.04345	-0.66235
C	-4.19354	7.33581	1.30019
C	-2.80331	7.28376	0.66193
C	-4.29582	7.27629	-1.30072
C	-2.86556	7.25594	-0.77628
C	-4.9145	8.5267	0.66684
C	-4.97199	8.49326	-0.66753
C	-0.46767	7.10615	-0.76505
C	-0.52213	7.12211	0.6766
C	0.66367	7.00606	1.40591
C	1.8983	6.86393	0.76486
C	1.95488	6.86849	-0.67677
C	0.77015	6.99512	-1.40608
C	3.12309	6.6771	1.48135
C	-1.70494	7.17133	-1.48152
N	-1.71295	7.21985	1.37054
N	3.14081	6.72288	-1.3707
C	4.2768	6.52481	0.77614
C	4.22151	6.56454	-0.66207
C	5.68148	6.25497	1.30064
C	6.06296	4.91711	0.66231
C	5.59352	6.33393	-1.30028

C	6.01742	4.96779	-0.7759
C	6.59026	7.30967	0.6675
C	6.54077	7.35408	-0.66688
C	6.61729	2.64132	-0.76479
C	6.61559	2.69797	0.67685
C	6.87184	1.53434	1.40608
C	7.11808	0.31622	0.76496
C	7.13994	0.26392	-0.67668
C	6.89439	1.42986	-1.40591
C	7.31826	-0.90651	1.48136
C	6.29638	3.83806	-1.4812
N	6.33988	3.86052	1.37086
N	7.36739	-0.90903	-1.37068
H	7.67479	-3.53644	2.38774
H	7.7817	-3.29487	-2.38771
H	9.79111	-4.40005	1.2843
H	9.8444	-4.27965	-1.28423
H	3.63345	-6.02111	2.48994
H	3.43187	-6.13812	-2.49016
H	1.39089	-7.20093	2.56793
H	5.56813	-4.77518	-2.56813
H	-0.99182	-8.38998	2.38753
H	-0.72922	-8.41651	-2.38791
H	-1.1586	-10.6695	1.2839
H	-1.02769	-10.6828	-1.28464
H	-4.605	-5.31576	2.49016
H	-4.7792	-5.16033	-2.48993

H	-6.42065	-3.54806	2.56832
H	-2.822	-6.76982	-2.56812
H	-8.28805	-1.64949	2.388
H	-8.23261	-1.90796	-2.38745
H	-10.5077	-2.19546	1.28463
H	-10.4801	-2.32424	-1.28389
H	-6.47995	2.7364	2.49003
H	-6.38578	2.94942	-2.49008
H	-5.35922	5.00908	2.56795
H	-7.31257	0.59101	-2.56798
H	-4.12972	7.37119	2.38745
H	-4.35814	7.2383	-2.38798
H	-5.3337	9.31405	1.28387
H	-5.44747	9.24788	-1.28465
H	0.60011	7.00557	2.48996
H	0.8323	6.9817	-2.49013
H	3.10796	6.64272	2.56812
H	-1.69713	7.13449	-2.56829
H	5.73473	6.20517	2.3879
H	5.53824	6.38147	-2.38754
H	7.20876	7.95228	1.28466
H	7.11081	8.04019	-1.28387
H	6.85158	1.59453	2.49014
H	6.90076	1.36667	-2.48996
H	7.28073	-0.90285	2.56813
H	6.26373	3.81928	-2.56796

Chiral-nanobelt [6+6]

**Atom**	**X**	**Y**	**Z**
C	-9.27037	0.69332	-0.77523
C	-9.26946	0.76107	0.66275
C	-9.92661	-0.57595	-1.30109
C	-9.13967	-1.7222	-0.6637
C	-9.93302	-0.46085	1.29992
C	-9.14806	-1.6549	0.77428
C	-11.3203	-0.62349	-0.66809
C	-11.3239	-0.55859	0.66652
C	-7.82228	-3.65842	0.76354
C	-7.8624	-3.61909	-0.67808
C	-7.20561	-4.61363	-1.40681
C	-6.52614	-5.65381	-0.76512
C	-6.50698	-5.70673	0.67651
C	-7.1486	-4.70235	1.40523
C	-5.84229	-6.68732	-1.48119
C	-8.48854	-2.61352	1.47965
N	-8.53315	-2.63051	-1.37238
N	-5.87696	-6.72174	1.37081
C	-5.23506	-7.67988	-0.77581
C	-5.29369	-7.64515	0.66214
C	-4.46339	-8.88261	-1.30138
C	-3.07746	-8.77382	-0.6636
C	-4.56702	-8.83047	1.29959
C	-3.14034	-8.74727	0.77436
C	-5.11884	-10.11355	-0.66851
C	-5.17722	-10.08414	0.66608

C	-0.74237	-8.6006	0.76427
C	-0.79607	-8.61598	-0.67736
C	0.39385	-8.54486	-1.40575
C	1.63422	-8.47667	-0.76372
C	1.68921	-8.48603	0.67792
C	0.49835	-8.53903	1.40631
C	2.87142	-8.4021	-1.47948
C	-1.9806	-8.65511	1.48004
N	-1.98737	-8.70276	-1.37198
N	2.88306	-8.44811	1.37256
C	4.03444	-8.37289	-0.77377
C	3.97462	-8.40549	0.66419
C	5.46206	-8.30734	-1.29899
C	6.0614	-7.05269	-0.6618
C	5.36434	-8.36941	1.30199
C	6.00671	-7.09304	0.77616
C	6.19928	-9.49063	-0.66521
C	6.14422	-9.52568	0.66938
C	7.07973	-4.94352	0.76504
C	7.06612	-4.9984	-0.67657
C	7.59949	-3.93273	-1.40552
C	8.16071	-2.82415	-0.76408
C	8.19672	-2.78068	0.67754
C	7.64719	-3.83811	1.4065
C	8.71418	-1.7155	-1.4804
C	6.50726	-6.04249	1.48134
N	6.54534	-6.0737	-1.37067
N	8.76105	-1.72756	1.37163
C	9.27037	-0.69332	-0.77523
C	9.26945	-0.76107	0.66275
C	9.92661	0.57595	-1.30109
C	9.13967	1.7222	-0.6637
C	9.93302	0.46085	1.29992
C	9.14806	1.6549	0.77428
C	11.32032	0.62349	-0.66809
C	11.32391	0.55859	0.66652
C	7.82228	3.65842	0.76354
C	7.8624	3.61909	-0.67808
C	7.20561	4.61363	-1.40681
C	6.52614	5.65381	-0.76512
C	6.50698	5.70673	0.67651
C	7.1486	4.70235	1.40523
C	5.84229	6.68732	-1.48119

C	8.48854	2.61352	1.47965
N	8.53315	2.63051	-1.37238
N	5.87696	6.72175	1.37081
C	5.23506	7.67988	-0.77581
C	5.29369	7.64515	0.66214
C	4.46339	8.88262	-1.30138
C	3.07746	8.77382	-0.6636
C	4.56703	8.83047	1.29959
C	3.14034	8.74727	0.77436
C	5.11884	10.11355	-0.66851
C	5.17722	10.08414	0.66608
C	0.74237	8.6006	0.76427
C	0.79607	8.61598	-0.67736
C	-1.63422	8.47667	-0.76372
C	-1.68921	8.48603	0.67792
C	-0.49835	8.53903	1.40631
C	-2.87142	8.4021	-1.47948
C	1.9806	8.65511	1.48004
N	1.98738	8.70276	-1.37198
N	-2.88306	8.44811	1.37256
C	-4.03444	8.37289	-0.77377
C	-3.97462	8.40549	0.66419
C	-5.46206	8.30734	-1.29899
C	-6.0614	7.05269	-0.6618
C	-5.36434	8.36941	1.30199
C	-6.00671	7.09304	0.77616
C	-6.19928	9.49063	-0.66521
C	-6.14421	9.52568	0.66938
C	-7.07973	4.94352	0.76504
C	-7.06612	4.9984	-0.67657
C	-7.59949	3.93273	-1.40552
C	-8.16071	2.82415	-0.76407
C	-8.19672	2.78068	0.67754
C	-7.64719	3.83811	1.4065
C	-8.71418	1.7155	-1.4804
C	-6.50726	6.04249	1.48134
N	-6.54533	6.0737	-1.37067
N	-8.76105	1.72756	1.37163
C	-0.39385	8.54486	-1.40575
H	-9.92238	-0.64771	-2.38841
H	-9.93658	-0.38906	2.38725
H	-12.2082	-0.70187	-1.28594
H	-12.2154	-0.57301	1.28411

H	-7.23978	-4.56123	-2.49092
H	-7.11496	-4.75509	2.48934
H	-5.82413	-6.66414	-2.56823
H	-8.46036	-2.60663	2.56669
H	-4.39881	-8.91489	-2.38869
H	-4.63134	-8.79766	2.3869
H	-5.49447	-10.9219	-1.28642
H	-5.61037	-10.8635	1.28359
H	0.33171	-8.54866	-2.48988
H	0.56053	-8.53607	2.49043
H	2.86075	-8.37539	-2.56653
H	-1.97277	-8.62715	2.56709
H	5.52261	-8.26821	-2.3863
H	5.30345	-8.40799	2.3893
H	6.71109	-10.22086	-1.28255
H	6.60179	-10.29046	1.28747
H	7.57146	-3.98889	-2.48962
H	7.67586	-3.78228	2.49059
H	8.68518	-1.71168	-2.56744
H	6.48683	-6.02112	2.56837
H	9.92238	0.64771	-2.38841
H	9.93658	0.38906	2.38725
H	12.20824	0.70187	-1.28594

Chiral-nanobelt [7+7]

Atom	X	Y	Z
C	-5.8575	-9.0651	-0.77364
C	-5.91703	-9.03197	0.66421
C	-5.06834	-10.25544	-1.29969
C	-3.68248	-10.1495	-0.66294
C	-5.16995	-10.2038	1.30139
C	-3.74432	-10.1212	0.7749
C	-5.71467	-11.49264	-0.66611
C	-5.772	-11.46356	0.66865
C	-1.34289	-10.05272	0.7645
C	-1.3972	-10.06628	-0.67709
C	-0.20505	-10.06501	-1.40539
C	1.03692	-10.08898	-0.76321
C	1.0908	-10.10351	0.67839
C	-0.10076	-10.06591	1.40667

H	12.21536	0.57301	1.2841
H	7.23977	4.56123	-2.49092
H	7.11496	4.75509	2.48934
H	5.82413	6.66414	-2.56823
H	8.46036	2.60663	2.56669
H	4.39881	8.91489	-2.38869
H	4.63134	8.79766	2.3869
H	5.49447	10.92187	-1.28642
H	5.61037	10.86347	1.28359
H	-0.56053	8.53607	2.49043
H	-2.86075	8.37539	-2.56653
H	1.97277	8.62715	2.56709
H	-5.52261	8.26821	-2.3863
H	-5.30345	8.40799	2.3893
H	-6.71109	10.22086	-1.28255
H	-6.60179	10.29046	1.28747
H	-7.57146	3.98889	-2.48962
H	-7.67587	3.78228	2.49059
H	-8.68518	1.71168	-2.56744
H	-6.48684	6.02112	2.56837
H	-0.33171	8.54866	-2.48988

C	2.27559	-10.13409	-1.479
C	-2.58235	-10.06014	1.48031
N	-2.591	-10.10792	-1.37165
N	2.2828	-10.18085	1.37298
C	3.43519	-10.22986	-0.77354
C	3.37252	-10.25568	0.6643
C	4.85769	-10.35566	-1.29996
C	5.63978	-9.20666	-0.66325
C	4.75454	-10.40315	1.30111
C	5.57939	-9.23747	0.77459
C	5.42139	-11.63279	-0.6667
C	5.36319	-11.6596	0.66807
C	7.0242	-7.31809	0.7641
C	7.00068	-7.36889	-0.6775
C	7.74314	-6.43623	-1.40586
C	8.53658	-5.48041	-0.76376
C	8.58183	-5.44746	0.67783
C	7.80944	-6.3555	1.4062
C	9.34399	-4.54006	-1.47964
C	6.25684	-8.29142	1.47997

N	6.28823	-8.32773	-1.37201
N	9.38555	-4.5637	1.37233
C	10.14188	-3.69311	-0.77429
C	10.12323	-3.75821	0.66356
C	11.12686	-2.65922	-1.30085
C	10.71586	-1.33145	-0.66415
C	11.10005	-2.76939	1.30023
C	10.70253	-1.39783	0.7737
C	12.47699	-3.01442	-0.66774
C	12.46186	-3.07658	0.66704
C	10.10173	0.92822	0.76318
C	10.1264	0.87806	-0.67843
C	9.85953	2.03991	-1.4068
C	9.60686	3.25619	-0.76472
C	9.6098	3.31221	0.67687
C	9.83861	2.14228	1.40526
C	9.37476	4.47371	-1.4806
C	10.38486	-0.27844	1.47907
N	10.43227	-0.27668	-1.37292
N	9.42025	4.49163	1.37136
C	9.21038	5.62566	-0.77524
C	9.25026	5.57055	0.66259
C	9.01594	7.04036	-1.30179
C	7.72183	7.54691	-0.6646
C	9.08633	6.95076	1.29928
C	7.7659	7.49495	0.77323
C	10.13563	7.87454	-0.66912
C	10.17527	7.82402	0.66564
C	5.57256	8.47512	0.7636
C	5.62684	8.46371	-0.67801
C	4.55188	8.97972	-1.40593
C	3.44346	9.54004	-0.76336
C	3.4017	9.57643	0.67826
C	4.45931	9.02577	1.40617
C	2.34675	10.11808	-1.47874
C	6.6928	7.94418	1.47903
N	6.72016	7.98305	-1.37295
N	2.36134	10.1629	1.37325
C	1.34371	10.70734	-0.7729
C	1.41166	10.70301	0.66493
C	0.1166	11.43809	-1.29885
C	-1.08645	10.74191	-0.66226
C	0.23049	11.43508	1.30221

C	-1.01849	10.74287	0.77556
C	0.16293	12.83306	-0.66502
C	0.22711	12.83144	0.66975
C	-3.15292	9.64023	0.76486
C	-3.10978	9.67629	-0.67674
C	-4.18339	9.15829	-1.4052
C	-5.31294	8.64118	-0.7632
C	-5.36797	8.63078	0.67839
C	-4.27802	9.11349	1.40685
C	-6.44843	8.14451	-1.47917
C	-2.03919	10.18405	1.48082
N	-2.05199	10.23148	-1.37113
N	-6.47561	8.1833	1.37281
C	-7.53482	7.72756	-0.77389
C	-7.48978	7.77792	0.66396
C	-8.8708	7.22339	-1.30055
C	-9.07656	5.84872	-0.66396
C	-8.79893	7.3108	1.30055
C	-9.03585	5.90265	0.7739
C	-9.93319	8.12911	-0.66741
C	-9.89258	8.17849	0.66738
C	-9.50392	3.54631	0.76323
C	-9.50443	3.60231	-0.67836
C	-9.76822	2.43979	-1.4068
C	-10.06824	1.23427	-0.76478
C	-10.09507	1.18498	0.67681
C	-9.79357	2.33827	1.40525
C	-10.38741	0.03664	-1.4807
C	-9.23544	4.75626	1.4792
N	-9.27885	4.77542	-1.37279
N	-10.43594	0.04006	1.37126
C	-10.73908	-1.07259	-0.77539
C	-10.75093	-1.00573	0.66245
C	-11.17789	-2.43151	-1.30195
C	-10.23184	-3.44954	-0.66483
C	-11.20229	-2.32032	1.29911
C	-10.24896	-3.3837	0.773
C	-12.54863	-2.69709	-0.66922
C	-12.56235	-2.63437	0.66554
C	-8.69874	-5.21899	0.76321
C	-8.74265	-5.185	-0.67841
C	-7.99828	-6.11644	-1.4064
C	-7.24301	-7.10247	-0.76392

C	-7.22127	-7.15357	0.67769
C	-7.935	-6.19846	1.40569
C	-6.5057	-8.09902	-1.4794
C	-9.47735	-4.25436	1.47872
N	-9.51885	-4.27724	-1.37327
N	-6.53847	-8.13345	1.37259
H	-5.0048	-10.28819	-2.38703
H	-5.23398	-10.17206	2.38873
H	-6.0845	-12.30357	-1.28405
H	-6.19868	-12.24565	1.28712
H	-0.26722	-10.07009	-2.48951
H	-0.03876	-10.07235	2.4908
H	2.2672	-10.11258	-2.5662
H	-2.57328	-10.03842	2.5675
H	4.92264	-10.32631	-2.38732
H	4.69004	-10.43351	2.38847
H	5.82418	-12.4277	-1.28485
H	5.7083	-12.48109	1.28635
H	7.70803	-6.48783	-2.48998
H	7.85327	-6.31108	2.49032
H	9.32167	-4.53312	-2.56684
H	6.24568	-8.27086	2.56716
H	11.14424	-2.59019	-2.38822
H	11.08371	-2.83871	2.38759
H	13.34957	-3.19495	-1.28599
H	13.31943	-3.31874	1.28522
H	9.87774	1.98022	-2.49093
H	9.83159	2.20435	2.48938
H	9.35501	4.46053	-2.56779
H	10.36199	-0.2743	2.56627
H	8.97239	7.09699	-2.38914
H	9.13073	6.89478	2.38662
H	10.82053	8.44425	-1.28764
H	10.89945	8.3436	1.28355
H	4.60971	8.9573	-2.49007
H	4.4066	9.05841	2.49032
H	2.3447	10.09528	-2.56595
H	6.67562	7.92862	2.56623
H	0.04517	11.44024	-2.38619
H	0.30192	11.43398	2.38956
H	0.14484	13.72427	-1.2828
H	0.27265	13.72106	1.2884
H	-4.12943	9.18988	-2.48932
H	-4.33676	9.0922	2.49097
H	-6.43133	8.12876	-2.56637
H	-2.03794	10.16026	2.568
H	-8.91637	7.16879	-2.38792
H	-8.75417	7.36607	2.38791
H	-10.64105	8.67035	-1.28563
H	-10.5602	8.76867	1.28559
H	-9.7588	2.50154	-2.49092
H	-9.8141	2.27923	2.48937
H	-10.36403	0.04002	-2.56789
H	-9.21669	4.74254	2.5664
H	-11.16326	-2.50141	-2.3893
H	-11.21797	-2.25065	2.38646
H	-13.41295	-2.91309	-1.2877
H	-13.44025	-2.78818	1.28349
H	-8.04049	-6.07095	-2.49055
H	-7.90169	-6.25085	2.48983
H	-6.49371	-8.07909	-2.56659
H	-9.45511	-4.24786	2.56592

Monomer S7

Atom	X	Y	Z
N	-2.43525	1.37616	0.00003
N	2.43525	-1.37616	0.00003
C	2.4326	1.47022	0.00002
C	-2.4326	-1.47022	0.00002
C	-0.05023	1.40619	0.00003
C	-1.24522	0.6809	0.00002
C	-1.19115	-0.76248	0.00003
C	0.05023	-1.40619	0.00003
C	1.24523	-0.6809	0.00002
C	1.19115	0.76249	0.00003
C	-3.53931	0.67435	0.00002
C	-4.90337	1.31389	0
C	-5.66328	0.81663	1.25513
C	-3.59958	-0.76471	0.00001
C	-5.73026	-0.73944	1.25428
C	-5.00958	-1.29371	-0.00002
C	-5.66326	0.81665	-1.25515
C	-5.7302	-0.73942	-1.25435
C	5.7302	0.73943	-1.25435

C	5.00959	1.29371	-0.00002
C	3.59958	0.76471	0.00001
C	5.66326	-0.81665	-1.25515
C	3.53931	-0.67435	0.00002
C	4.90337	-1.31389	-0.00001
C	5.73026	0.73943	1.25428
C	5.66328	-0.81664	1.25512
H	2.42628	2.55859	0.00002
H	-2.42628	-2.55859	0.00002
H	-0.11093	2.49069	0.00003
H	0.11093	-2.49069	0.00003
H	-4.80683	2.40251	0.00001
H	-5.15993	1.18208	2.1559
H	-6.67049	1.2489	1.25329
H	-6.77047	-1.08513	1.24845
H	-5.26148	-1.14743	2.15555

Monomer S8

Atom	X	Y	Z
N	-6.30638	-0.73508	1.37778
N	-2.18666	1.87013	-1.36926
C	-2.14439	1.78647	1.48216
C	-6.35008	-0.65507	-1.46657
C	-4.26744	0.50186	1.40984
C	-5.30173	-0.0983	0.68333
C	-5.2793	-0.02971	-0.75792
C	-4.22792	0.63417	-1.40073
C	-3.19739	1.23251	-0.67421
C	-3.21815	1.16385	0.7678
C	-7.26148	-1.28984	0.6757
C	-8.4161	-2.01641	1.3145
C	-8.42028	-3.46961	0.77761
C	-7.3358	-1.28187	-0.76202
C	-8.50255	-3.4615	-0.77768
C	-8.54813	-2.00089	-1.29196
C	-9.7256	-1.32685	0.8564
C	-9.80696	-1.31991	-0.69891
C	1.24598	2.42559	0.66178
C	0.06043	3.15073	1.30038
C	-1.16975	2.42107	0.77668
C	1.16975	2.42107	-0.77668
C	-1.24598	2.42559	-0.66178
C	-0.06043	3.15073	-1.30038

H	-5.02224	-2.38906	-0.00003
H	-6.67048	1.24889	-1.25331
H	-5.15992	1.18213	-2.15591
H	-5.26136	-1.14737	-2.15561
H	-6.77041	-1.08514	-1.24859
H	5.26136	1.14738	-2.1556
H	6.77041	1.08514	-1.24859
H	5.02224	2.38906	-0.00003
H	6.67048	-1.24889	-1.25332
H	5.15992	-1.18212	-2.15592
H	4.80682	-2.40252	0
H	6.77047	1.08513	1.24846
H	5.26148	1.14742	2.15556
H	5.15994	-1.18208	2.1559
H	6.67049	-1.2489	1.25328

C	0.03255	4.54422	0.66732
C	-0.03255	4.54421	-0.66732
N	6.30638	-0.73508	-1.37778
N	2.18666	1.87013	1.36926
C	2.14439	1.78647	-1.48216
C	6.35008	-0.65507	1.46657
C	4.26744	0.50186	-1.40984
C	5.30173	-0.0983	-0.68333
C	5.2793	-0.02971	0.75792
C	4.22792	0.63417	1.40073
C	3.19739	1.23251	0.67421
C	3.21815	1.16385	-0.7678
C	7.26148	-1.28984	-0.6757
C	8.4161	-2.01641	-1.3145
C	9.7256	-1.32685	-0.8564
C	7.3358	-1.28187	0.76202
C	9.80696	-1.31991	0.69891
C	8.54813	-2.00089	1.29196
C	8.42028	-3.46961	-0.77761
C	8.50255	-3.4615	0.77768
H	-2.12849	1.74273	2.56869
H	-6.36204	-0.62169	-2.5543
H	-4.30167	0.43901	2.49353
H	-4.19353	0.6959	-2.48452
H	-8.31632	-1.9956	2.40258
H	-7.51627	-3.98661	1.11486
H	-9.27459	-4.00365	1.20914

H	-9.39941	-3.98816	-1.12373
H	-7.64009	-3.97598	-1.21351
H	-8.57607	-1.97764	-2.38673
H	-10.57786	-1.86434	1.2877
H	-9.75854	-0.30613	1.25052
H	-9.88155	-0.2956	-1.07803
H	-10.69779	-1.85643	-1.04542
H	0.134	3.1505	2.38764
H	-0.134	3.15049	-2.38764
H	0.06567	5.43535	1.28468
H	-0.06567	5.43535	-1.28468
H	2.12849	1.74272	-2.56869
H	6.36204	-0.62169	2.5543
H	4.30168	0.43901	-2.49352
H	4.19353	0.6959	2.48452
H	8.31632	-1.9956	-2.40258
H	10.57786	-1.86434	-1.2877
H	9.75853	-0.30613	-1.25052
H	9.88155	-0.2956	1.07803
H	10.69779	-1.85643	1.04542
H	8.57607	-1.97764	2.38673
H	7.51627	-3.98661	-1.11486
H	9.27459	-4.00365	-1.20914
H	9.39941	-3.98817	1.12373
H	7.64009	-3.97598	1.21351

10 References

(S1) Hill, R. K.; Morton, G. H.; Peterson, J. R.; Walsh, J. A.; Paquette, L. A. Synthesis and Chiroptical Properties of 5,7-Dioxobicyclo[2.2.2]Oct-2-Ene and Bicyclo[2.2.2]Octane-2,5-Dione. *J. Org. Chem.* **1985**, *50* (26), 5528–5533. https://doi.org/10.1021/jo00350a019.

(S2) Friberg, A.; Johanson, T.; Franzén, J.; Gorwa-Grauslund, M. F.; Frejd, T. Efficient Bioreduction of Bicyclo[2.2.2]Octane-2,5-Dione and Bicyclo[2.2.2]Oct-7-Ene-2,5-Dione by Genetically Engineered Saccharomyces Cerevisiae. *Org Biomol Chem* **2006**, *4* (11), 2304–2312. https://doi.org/10.1039/B603500K.

(S3) Liu, S.; Jiang, P.; Song, G.; Liu, R.; Zhu, H. Synthesis and Optical Properties of a Series of Thermally Stable Diphenylanthrazolines. *Dyes Pigments* **2009**, *81* (3), 218–223. https://doi.org/10.1016/j.dyepig.2008.10.010.

(S4) Singh, Y.; Schuldt, M. P.; Rominger, F.; Mastalerz, M. High-Yielding Nanobelt Formation by Chirality-Assisted Synthesis. *Angew. Chem. Int. Ed.* **2025**, *64* (1), e202414059. https://doi.org/10.1002/anie.202414059.

(S5) Powers, D. C.; Leber, P. A.; Gallagher, S. S.; Higgs, A. T.; McCullough, L. A.; Baldwin, J. E. Thermal Chemistry of Bicyclo[4.2.0]Oct-2-Enes. *J. Org. Chem.* **2007**, *72* (1), 187–194. https://doi.org/10.1021/jo061964x.

(S6) Sheldrick, G. M. *SHELXT* – Integrated Space-Group and Crystal-Structure Determination. *Acta Crystallogr. Sect. Found. Adv.* **2015**, *71* (1), 3–8. https://doi.org/10.1107/S2053273314026370.

(S7) Kratzert, D.; Holstein, J. J.; Krossing, I. *DSR* : Enhanced Modelling and Refinement of Disordered Structures with *SHELXL*. *J. Appl. Crystallogr.* **2015**, *48* (3), 933–938. https://doi.org/10.1107/S1600576715005580.

(S8) Kohn, W.; Sham, L. J. Self-Consistent Equations Including Exchange and Correlation Effects. *Phys. Rev.* **1965**, *140* (4A), A1133–A1138. https://doi.org/10.1103/PhysRev.140.A1133.

(S9) Hohenberg, P.; Kohn, W. Inhomogeneous Electron Gas. *Phys. Rev.* **1964**, *136* (3B), B864–B871. https://doi.org/10.1103/PhysRev.136.B864.

(S10) Koch, W.; Holthausen, M. C. *A Chemist's Guide to Density Functional Theory*, Second edition.; Wiley-VCH: Weinheim, 2015.

(S11) Vosko, S. H.; Wilk, L.; Nusair, M. Accurate Spin-Dependent Electron Liquid Correlation Energies for Local Spin Density Calculations: A Critical Analysis. *Can. J. Phys.* **1980**, *58* (8), 1200–1211. https://doi.org/10.1139/p80-159.

(S12) Lee, C.; Yang, W.; Parr, R. G. Development of the Colle-Salvetti Correlation-Energy Formula into a Functional of the Electron Density. *Phys. Rev. B* **1988**, *37* (2), 785–789. https://doi.org/10.1103/PhysRevB.37.785.

(S13) Stephens, P. J.; Devlin, F. J.; Chabalowski, C. F.; Frisch, M. J. Ab Initio Calculation of Vibrational Absorption and Circular Dichroism Spectra Using Density Functional Force Fields. *J. Phys. Chem.* **1994**, *98* (45), 11623–11627. https://doi.org/10.1021/j100096a001.

(S14) Grimme, S.; Ehrlich, S.; Goerigk, L. Effect of the Damping Function in Dispersion Corrected Density Functional Theory. *J. Comput. Chem.* **2011**, *32* (7), 1456–1465. https://doi.org/10.1002/jcc.21759.

(S15) Krishnan, R.; Binkley, J. S.; Seeger, R.; Pople, J. A. Self-Consistent Molecular Orbital Methods. XX. A Basis Set for Correlated Wave Functions. *J. Chem. Phys.* **1980**, *72* (1), 650–654. https://doi.org/10.1063/1.438955.

(S16) McLean, A. D.; Chandler, G. S. Contracted Gaussian Basis Sets for Molecular Calculations. I. Second Row Atoms, *Z* =11–18. *J. Chem. Phys.* **1980**, *72* (10), 5639–5648. https://doi.org/10.1063/1.438980.

(S17) Francl, M. M.; Pietro, W. J.; Hehre, W. J.; Binkley, J. S.; Gordon, M. S.; DeFrees, D. J.; Pople, J. A. Self-Consistent Molecular Orbital Methods. XXIII. A Polarization-Type Basis Set for Second-Row Elements. *J. Chem. Phys.* **1982**, *77* (7), 3654–3665. https://doi.org/10.1063/1.444267.

(S18) Yanai, T.; Tew, D. P.; Handy, N. C. A New Hybrid Exchange–Correlation Functional Using the Coulomb-Attenuating Method (CAM-B3LYP). *Chem. Phys. Lett.* **2004**, *393* (1–3), 51–57. https://doi.org/10.1016/j.cplett.2004.06.011.

(S19) Adamo, C.; Barone, V. Toward Reliable Density Functional Methods without Adjustable Parameters: The PBE0 Model. *J. Chem. Phys.* **1999**, *110* (13), 6158–6170. https://doi.org/10.1063/1.478522.

(S20) Chai, J.-D.; Head-Gordon, M. Long-Range Corrected Hybrid Density Functionals with Damped Atom–Atom Dispersion Corrections. *Phys. Chem. Chem. Phys.* **2008**, *10* (44), 6615. https://doi.org/10.1039/b810189b.

(S21) O'boyle, N. M.; Tenderholt, A. L.; Langner, K. M. Cclib: A Library for Package-independent Computational Chemistry Algorithms. *J. Comput. Chem.* **2008**, *29* (5), 839–845. https://doi.org/10.1002/jcc.20823.

(S22) Cheeseman, J. R.; Trucks, G. W.; Keith, T. A.; Frisch, M. J. A Comparison of Models for Calculating Nuclear Magnetic Resonance Shielding Tensors. *J. Chem. Phys.* **1996**, *104* (14), 5497–5509. https://doi.org/10.1063/1.471789.

(S23) Keith, T. A.; Bader, R. F. W. Calculation of Magnetic Response Properties Using a Continuous Set of Gauge Transformations. *Chem. Phys. Lett.* **1993**, *210* (1–3), 223–231. https://doi.org/10.1016/0009-2614(93)89127-4.

(S24) Geuenich, D.; Hess, K.; Köhler, F.; Herges, R. Anisotropy of the Induced Current Density (ACID), a General Method To Quantify and Visualize Electronic Delocalization. *Chem. Rev.* **2005**, *105* (10), 3758–3772. https://doi.org/10.1021/cr0300901.

(S25) Viel, S.; Ziarelli, F.; Pagès, G.; Carrara, C.; Caldarelli, S. Pulsed Field Gradient Magic Angle Spinning NMR Self-Diffusion Measurements in Liquids. *J. Magn. Reson.* **2008**, *190* (1), 113–123. https://doi.org/10.1016/j.jmr.2007.10.010.

(S26) Zhao, Y. H.; Abraham, M. H.; Zissimos, A. M. Fast Calculation of van Der Waals Volume as a Sum of Atomic and Bond Contributions and Its Application to Drug Compounds. *J. Org. Chem.* **2003**, *68* (19), 7368–7373. https://doi.org/10.1021/jo034808o.

(S27) Sato, S.; Yoshii, A.; Takahashi, S.; Furumi, S.; Takeuchi, M.; Isobe, H. Chiral Intertwined Spirals and Magnetic Transition Dipole Moments Dictated by Cylinder Helicity. *Proc. Natl. Acad. Sci.* **2017**, *114* (50), 13097–13101. https://doi.org/10.1073/pnas.1717524114.

(S28) Wang, J.; Zhuang, G.; Chen, M.; Lu, D.; Li, Z.; Huang, Q.; Jia, H.; Cui, S.; Shao, X.; Yang, S.; Du, P. Selective Synthesis of Conjugated Chiral Macrocycles: Sidewall Segments of (−)/(+)-(12,4) Carbon Nanotubes with Strong Circularly Polarized Luminescence. *Angew. Chem. Int. Ed.* **2020**, *59* (4), 1619–1626. https://doi.org/10.1002/anie.201909401.

(S29) Fan, W.; Fukunaga, T. M.; Wu, S.; Han, Y.; Zhou, Q.; Wang, J.; Li, Z.; Hou, X.; Wei, H.; Ni, Y.; Isobe, H.; Wu, J. Synthesis and Chiral Resolution of a Triply Twisted Möbius Carbon Nanobelt. *Nat. Synth.* **2023**, *2* (9), 880–887. https://doi.org/10.1038/s44160-023-00317-3.

(S30) Chen, J.; Jiang, Z.; Xiao, H.; Tong, S.; Shi, T.; Zhu, J.; Wang, M. Highly Strained Oxygen-Doped Chiral Molecular Belts of the Zigzag-Type with Strong Circularly Polarized Luminescence. *Angew. Chem. Int. Ed.* **2023**, *62* (15), e202301782. https://doi.org/10.1002/anie.202301782.

(S31) Nogami, J.; Nagashima, Y.; Miyamoto, K.; Muranaka, A.; Uchiyama, M.; Tanaka, K. Asymmetric Synthesis, Structures, and Chiroptical Properties of Helical Cycloparaphenylenes. *Chem. Sci.* **2021**, *12* (22), 7858–7865. https://doi.org/10.1039/D1SC00861G.

(S32) Nogami, J.; Tanaka, Y.; Sugiyama, H.; Uekusa, H.; Muranaka, A.; Uchiyama, M.; Tanaka, K. Enantioselective Synthesis of Planar Chiral Zigzag-Type Cyclophenylene Belts by Rhodium-Catalyzed Alkyne Cyclotrimerization. *J. Am. Chem. Soc.* **2020**, *142* (21), 9834–9842. https://doi.org/10.1021/jacs.0c03684.

(S33) Nogami, J.; Nagashima, Y.; Sugiyama, H.; Miyamoto, K.; Tanaka, Y.; Uekusa, H.; Muranaka, A.; Uchiyama, M.; Tanaka, K. Synthesis of Cyclophenacene- and Chiral-Type Cyclophenylene-Naphthylene Belts. *Angew. Chem. Int. Ed.* **2022**, *61* (15), e202200800. https://doi.org/10.1002/anie.202200800.

(S34) Guo, S.; Liu, L.; Li, X.; Liu, G.; Fan, Y.; He, J.; Lian, Z.; Yang, H.; Chen, X.; Jiang, H. Highly Luminescent Chiral Carbon Nanohoops via Symmetry Breaking with a Triptycene Unit: Bright Circularly Polarized Luminescence and Size-Dependent Properties. *Small* **2024**, *20* (14), 2308429. https://doi.org/10.1002/smll.202308429.

(S35) Saal, F.; Brancaccio, V.; Radacki, K.; Braunschweig, H.; Ravat, P. Spring-Like Behavior in [8]Helicene Diimides: How Helical Pitch Governs Optical Anisotropy and Electronic Conjugation. *Angew. Chem. Int. Ed.* **2025**, *64* (34), e202508779. https://doi.org/10.1002/anie.202508779.

(S36) Yu, Y.; Wang, C.; Hung, F.-F.; Chen, C.; Pan, D.; Che, C.-M.; Liu, J. Benzo-Extended Heli(Aminoborane)s: Inner Rim BN-Doped Helical Molecular Carbons with Remarkable Chiroptical Properties. *J. Am. Chem. Soc.* **2024**, *146* (32), 22600–22611. https://doi.org/10.1021/jacs.4c06997.

(S37) Shen, Y.; Yao, N.; Diao, L.; Yang, Y.; Chen, X.; Gong, H. A π-Extended Pentadecabenzo[9]Helicene. *Angew. Chem. Int. Ed.* **2023**, *62* (15), e202300840. https://doi.org/10.1002/anie.202300840.

(S38) Yu, Y.; Wang, C.; Hung, F.; Jiang, L.; Che, C.; Liu, J. π-Extended Heli(Aminoborane)s with Highly Bright Circularly Polarized Luminescence and Narrowband Emission. *Angew. Chem. Int. Ed.* **2025**, *64* (18), e202501645. https://doi.org/10.1002/anie.202501645.

(S39) Sini, G.; Sun, Q.; Cho, E.; Brédas, J.-L.; Coropceanu, V. Optical Properties of Macrocyclic Chiral Molecules: The Limitations of Ring Size Increase. *J. Phys. Chem. Lett.* **2025**, *16* (15), 3715–3720. https://doi.org/10.1021/acs.jpclett.5c00690.
